# Regiodivergent metal-catalyzed B(4)- and C(1)-selenylation of *o*-carboranes†

**DOI:** 10.1039/d2sc05590b

**Published:** 2022-12-03

**Authors:** Kyungsup Lee, Jordan L. Harper, Tae Hyeon Kim, Hee Chan Noh, Dongwook Kim, Paul Ha-Yeon Cheong, Phil Ho Lee

**Affiliations:** a Department of Chemistry, Kangwon National University Chuncheon 24341 Republic of Korea phlee@kangwon.ac.kr; b Department of Chemistry, Oregon State University Corvallis Oregon 97331 USA cheongh@oregonstate.edu; c Department of Chemistry, Korea Advanced Institute of Science and Technology (KAIST) Daejeon 34141 Republic of Korea; d Center for Catalytic Hydrocarbon Functionalizations, Institute for Basic Science (IBS) Daejeon 34141 Republic of Korea

## Abstract

Regiodivergent transition metal-catalyzed B(4)- and C(1)-selenylation reactions of *o*-carboranes have been demonstrated. Namely, Ru(ii)-catalysis selectively generated B(4)-selenylated *o*-carboranes from the reaction of *o*-carborane acids with arylselenyl bromides with the release of carbon dioxide. In contrast, Pd(ii) catalysis provided exclusively C(1)-selenylated *o*-carboranes from the decarboxylative reaction of *o*-carborane acids with diaryl diselenides. In contrast to previous milestones in this area, these reactions demonstrate broad substrate scope with excellent yields. Combination of these methods leads to the formation of B(4)–C(1)-diselenylated *o*-carboranes. DFT studies revealed the mechanism of the Ru-process, with initial selenylation of the carborane cluster discovered to be essential for an energetically reasonable decarboxylation. This results in selenylation on the B(4) position prior to the decarboxylation event at C(1). This contrasted with the Pd-process in which the ready decarboxylation at C(1) leads to selenylation at C(1).

## Abstract 2

Regiodivergent transition metal-catalyzed B(4)- and C(1)-selenylation reactions of *o*-carboranes have been demonstrated.

## Graphical abstract

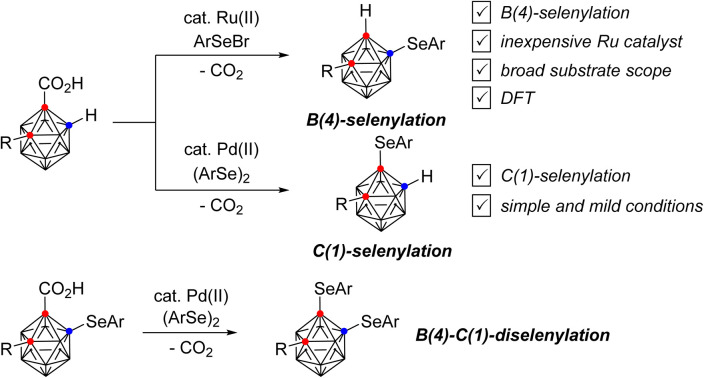

## Introduction

Carboranes, icosahedral boron-carbon molecular clusters,^1^ have recently found a variety of applications ranging from versatile building blocks in the construction of boron neutron capture therapy agents^2^ and ligands in organometallic/coordination chemistry^3^ to the design of supramolecular materials.^4^ Accordingly, the selective introduction of functional groups onto the boron and carbon vertexes of carboranes through B–H and C–H functionalization has received considerable attention.^5^ Of late, a large number of transition metal-catalyzed functionalization reactions of *o*-carboranes have been reported, allowing for the introduction of a wide range of functional groups onto the boron and carbon vertexes.^6^

Organoselenium compounds occupy a significant place in organic synthesis due to their latent bioactivities.^7^ In particular, aryl selenide scaffolds are frequently found in drug candidates displaying a wide range of biological activities^8^ and have versatile applications in materials science.^9^ As a result, the development of synthetic methods for the generation of aryl selenides, as well as methods for their introduction as functional groups, has attracted tremendous research interest.

**Scheme 1:**
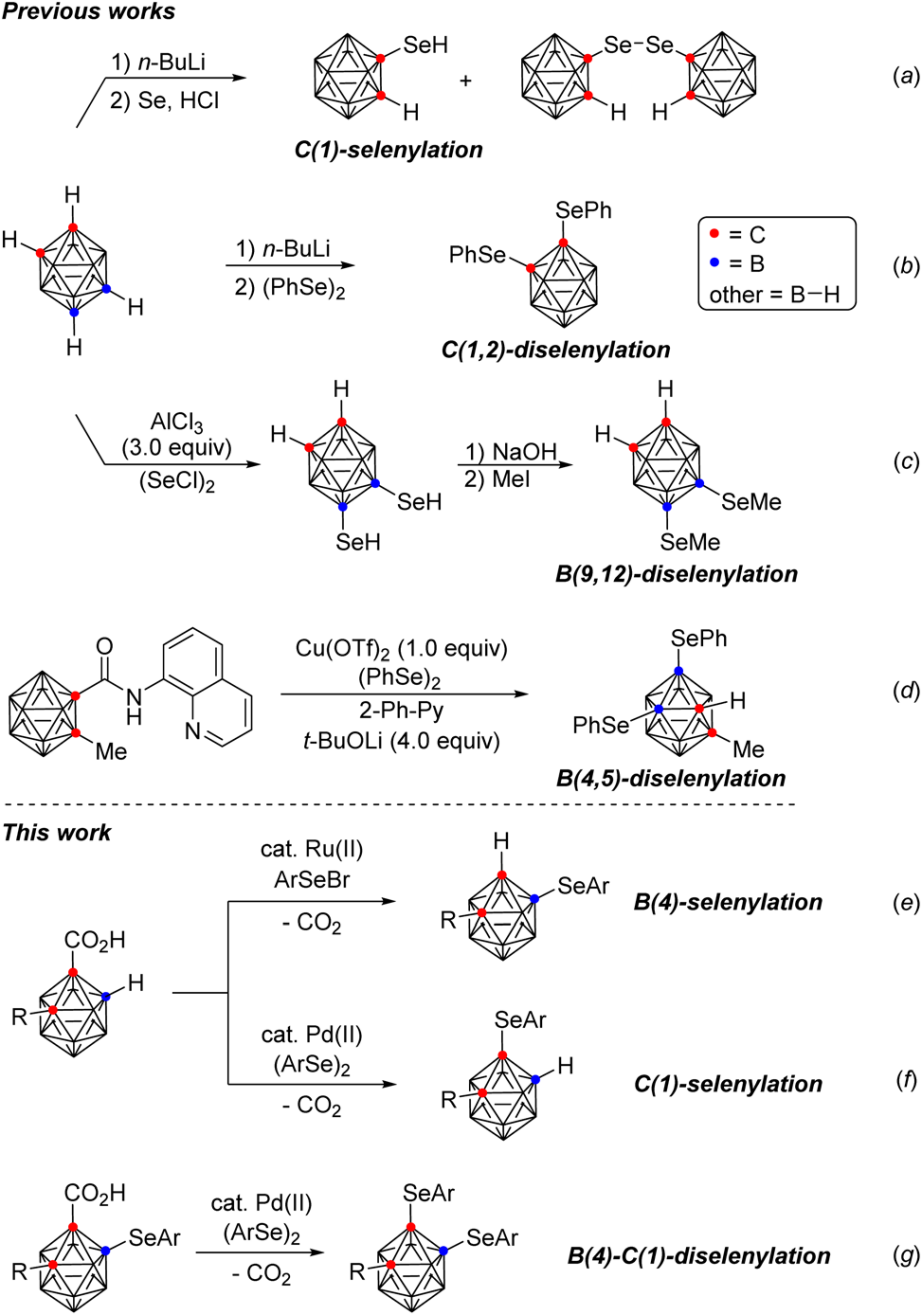
Regioselective selenylation of *o*-carboranes.

In our continuing efforts to develop synthetic methodology for the regioselective B–H functionalization of *o*-carboranes,^10^ we envisioned site selective syntheses of *o*-carboranes bearing organoselenyl groups through transition metal-catalyzed, regioselective B–Se and C–Se bond formations. To date, a limited number of methods for the synthesis of such compounds have been reported (Scheme 1): (a) C(1)-selenylation through the reaction of *o*-carboranes with *n*-BuLi followed by the addition of Se and HCl,^11^ (b) C(1,2)-diselenylation through the treatment of dilithio-*o*-carboranes obtained from *o*-carboranes and *n*-BuLi with diphenyl diselenide,^12^ (c) B(9,12)-diselenylation through the reaction of *o*-carboranes with (SeCl)_2_ in the presence of AlCl_3_ (3.0 equiv.),^13^ and (d) B(4,5)-diselenylation through a traceless, bidentate directing group-guided Cu-mediated reaction of *o*-carboranes with diphenyl diselenide.^14^ These methods have several disadvantages such as harsh reaction conditions including overly basic reaction conditions, excessive use of AlCl_3_ (3.0 equiv.) and Cu(OTf)_2_ (1.0 equiv.)/*t*-BuOLi (4.0 equiv.), as well as limited substrate scope, often restricted to only a single example. Although tremendous progress has been made in the areas of boron cluster and organoselenium chemistries, control of selectivity between B- and C-arylselenyl functionalization in *o*-carboranes remains a significant challenge. Furthermore, direct selenylation of an inert B–H bond is especially challenging due to the strong coordinating properties of organoselenium compounds.^15^ Herein, we demonstrate regiodivergent B(4)- and C(1)-selenylation of *o*-carboranes through the use of Ru(ii) and Pd(ii) catalysts respectively (e and f). Moreover, these methods may also be combined to achieve selective B(4)- and C(1)-diselenyation (g).

## Results and discussion

First, we investigated the reaction of 2-methyl *o*-carborane acid (1a) with phenylselenyl bromide (2a) in the presence of [Ru(*p*-cymene)Cl_2_]_2_ (5.0 mol%), Cu(OAc)_2_·H_2_O (2.0 equiv.), and K_2_CO_3_ (1.5 equiv.) at 70 °C for 12 h in a V-vial (Table 1). Dichloroethane (DCE) gave the desired B(4)-selenylated *o*-carborane (3a) in 13% yield (entry 1). Encouraged by this result, a variety of solvents were examined. Tetrahydrofuran (THF) and methanol disappointingly gave decarboxylated *o*-carborane 4 (entries 2 and 3). However, hexafluoroisopropanol (HFIP) and trifluoroethanol (TFE) selectively provided 3a in 50% and 83% yields respectively (entries 4 and 5). Next, a number of bases, including K_2_HPO_4_, KOAc, and CsOAc, were examined in TFE (entries 5–8) and CsOAc was the choice of base, providing 3a in 85% yield (entry 8). When the reaction temperature was reduced to 50 °C, the yield of 3a dropped to 20% (entry 9), and when the temperature was raised to 90 °C, decarboxylation of 1a occurred, reducing the yield of 3a to 67% (entry 10). The best results were obtained using 1.5 equivalents of 2a at 70 °C and 3a was thus obtained in 91% isolated yield (entry 11).

**Table 1:** Reaction optimization^a^

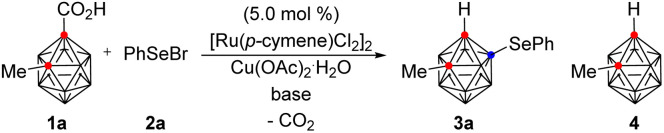				
Entry	Base	Solvent	Temp. (°C)	Yield^b^ (%)
1	K_2_CO_3_	DCE	70	13
2	K_2_CO_3_	THF	70	97^c^
3	K_2_CO_3_	MeOH	70	82^c^
4	K_2_CO_3_	HFIP	70	50
5	K_2_CO_3_	TFE	70	83
6	K_2_HPO_4_	TFE	70	58
7	KOAc	TFE	70	61
8	CsOAc	TFE	70	85
9	CsOAc	TFE	50	20
10	CsOAc	TFE	90	67
11^e^	CsOAc	TFE	70	93(91)^d^
12^f^	CsOAc	TFE	70	78

a Reactions were carried out with 1a (0.1 mmol, 1.0 equiv.) and 2a (2.0 equiv.) in the presence of [Ru(*p*-cymene)Cl_2_]_2_ (5.0 mol%), Cu(OAc)_2_·H_2_O (2.0 equiv.), and base (1.5 equiv.) in solvent (1.0 mL) for 12 h in a V-vial.

b NMR yield using CH_2_Br_2_ as an internal standard.

c NMR yield of decarboxylated *o*-carborane (4) of 1a.

d Isolated yield.

e 2a (1.5 equiv.) was used.

f 2a (1.2 equiv.) was used.

To demonstrate the efficiency and scope of these decarboxylative B(4)-selenylation reactions, we applied this catalytic system to a variety of aryl selenyl bromides 2 in the reaction with 1 (Table 2). When 1a was reacted with 2-methylphenyl and 4-methylphenyl organoselenyl bromides, the desired products 3b and 3c were obtained in 93% and 98% yields, respectively. The structure of 3c was unambiguously confirmed by X-ray crystallography (see the ESI†). The location and identity of the substituents on the aryl ring of the selenyl bromide affect the reaction efficiency. For example, the use of 2-methoxyphenylselenyl bromide provided 3d in 83% yield, while the use of 4-methoxylphenylselenyl bromide afforded 3e in 51% yield. Additionally, when 3-bromophenylselenyl bromide gave the corresponding product 3j in 16% yield, the reaction conditions were modified; gratifyingly, the use of hexafluoroisopropanol (HFIP) instead of TFE increased the yield of 3j to 70%. Other aryl selenyl bromides bearing halo substituents on the phenyl ring were also well tolerated under the modified reaction conditions, giving the corresponding arylselenylated *o*-carboranes 3f–3k in moderate to good yields, varying from 51% to 72%. On the other hand, *n*-pentylselenyl bromide was less reactive, and the reaction with 1a in TFE produced 3l in 35% yield. Nevertheless, to demonstrate the applicability of the present method to larger scale processes, 6.0 mmol of 1a (1.21 g) was treated with 2a (1.5 equiv.) under the optimum reaction conditions, giving 3a in 97% yield.

**Table 2:** Scope of organoselenyl bromides and *o*-carboranes in B(4)-selenylation^a^

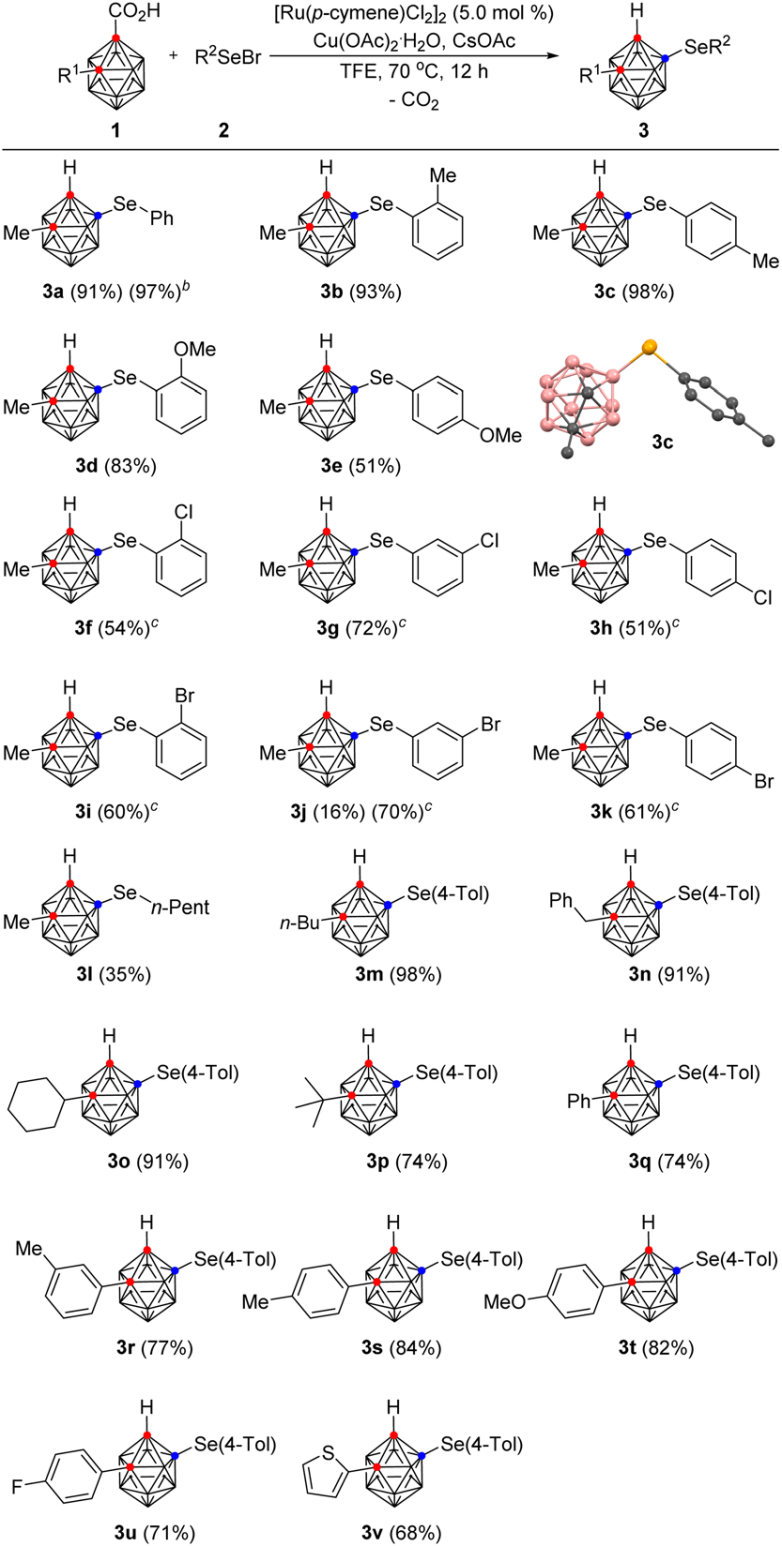

a Reaction conditions: 1 (0.10 mmol, 1.0 equiv.) reacted with 2 (1.5 equiv.) in the presence of [Ru(*p*-cymene)Cl_2_]_2_ (5.0 mol%), Cu(OAc)_2_.H_2_O (2.0 equiv.), and CsOAc (1.5 equiv.) in TFE (1.0 mL) at 70 °C for 12 h in a V-vial.

b Reaction scale is 6.0 mmol of 1a.

c HFIP was used as a solvent.

Stimulated by these results, a wide range of alkyl- and aryl-substituted *o*-carboranes 1 were examined with *p*-tolylselenyl bromide (2c). When *n*-butyl-, benzyl-, and cyclohexyl-substituted *o*-carboranes were reacted with 2c under the optimized reaction conditions, the corresponding selenylated products 3m, 3n, and 3o, were obtained in excellent yields, ranging from 91% to 98%. In addition, *tert*-butyl-substituted *o*-carboranes gave the desired product (3p) in 74% yield, despite the possibility of steric interference. Phenyl-substituted *o*-carboranes were also smoothly converted to the selenylated product (3q) in 74% yield. Electronic modification of the substituents on the aryl ring of 1 did not largely influence the efficiency of the B(4)-selenylation. For example, *o*-carboranes bearing 3- and 4-methyl-, 4-methoxy-, and 4-fluoro-substituted phenyl groups are all amenable to the reaction conditions, providing the desired products (3r–3u) in good yields, varying from 71% to 84%. Thiophen-2-yl-substituted *o*-carboranes are also compatible, giving 3v in 68% yield.

In addition to B(4)-selenylation, we also sought to install selenium substituents at other positions on the *o*-carborane. We thus envisioned that other transition metal catalysts would exhibit selectivity in contrast to that displayed by Ru. Accordingly, a variety of transition metal catalysts and selenylating agents were examined (see the ESI†). To our delight, the reaction of 1a with diphenyl diselenide and PdCl_2_ (5.0 mol%) in DMSO at 60 °C for 6 h provided C(1)-selenylated *o*-carborane 6a regioselectively in 97% yield with the release of carbon dioxide. Phenylselenyl bromide gave an inferior result (52%) compared to diphenyl diselenide. To demonstrate the efficiency and scope of this method, we applied the Pd-catalytic system to a wide range of 2-substituted *o*-carborane acids and diaryl diselenides (Table 3). The presence of various substituents on the aryl rings of the diphenyl diselenides had little effect on either the reaction rate or the product yield. Electron-donating groups such as methyl and methoxy, as well as electron-withdrawing groups such as chloride, bromide, and trifluoromethyl all afforded the corresponding C(1)-selenylated *o*-carboranes in high yields, ranging from 83% to 93%. Di(thiophen-2-yl) diselenide also smoothly underwent C(1)-selenylation to produce 6g in 92% yield. *o*-Carborane acids bearing *n*-butyl, cyclohexyl, and 4-methoxyphenyl groups on the C(2)-position are also amenable to C(1)-selenylation, affording the desired products (6h–6j) in good to excellent yields, varying from 72% to 94%.

**Table 3:** Scope of *o*-carboranes and diaryl diselenides in C(1)-selenylation^a^

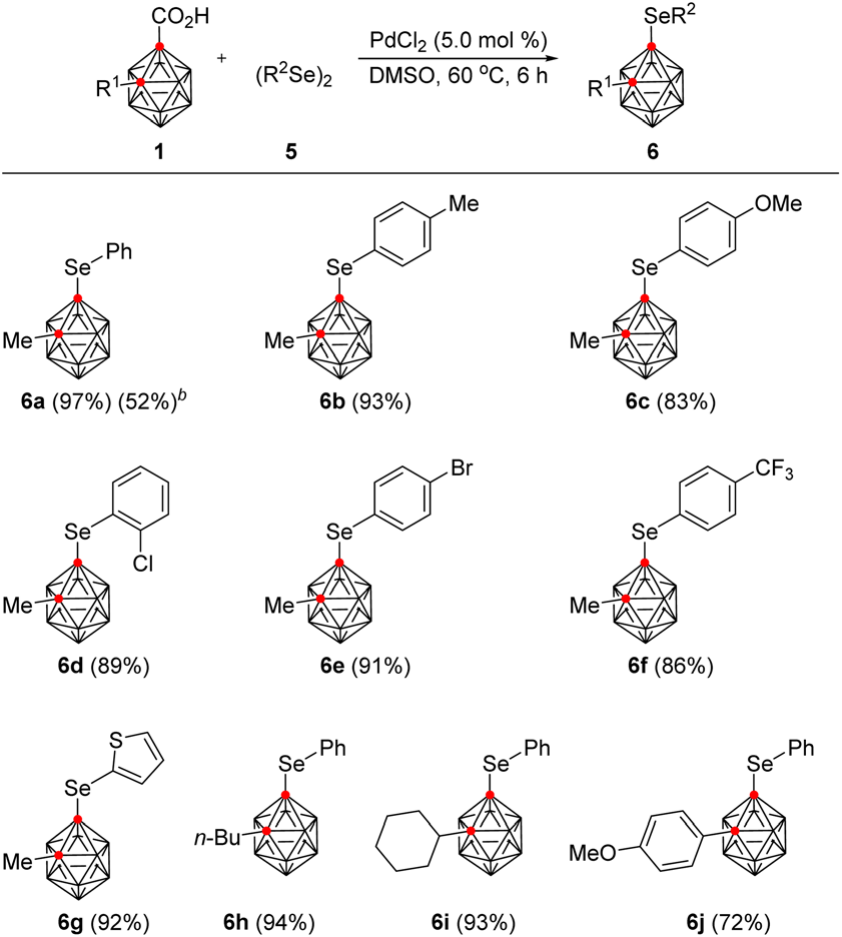

a Reaction conditions: 1 (0.10 mmol, 1.0 equiv.) reacted with 5 (1.2 equiv.) in the presence of PdCl_2_ (5.0 mol%) in DMSO (0.5 mL, 0.20 M) at 60 °C for 6 h in a test tube.

b PhSeBr (2.0 equiv.) was used instead of diphenyl diselenide.

**Scheme 2:**
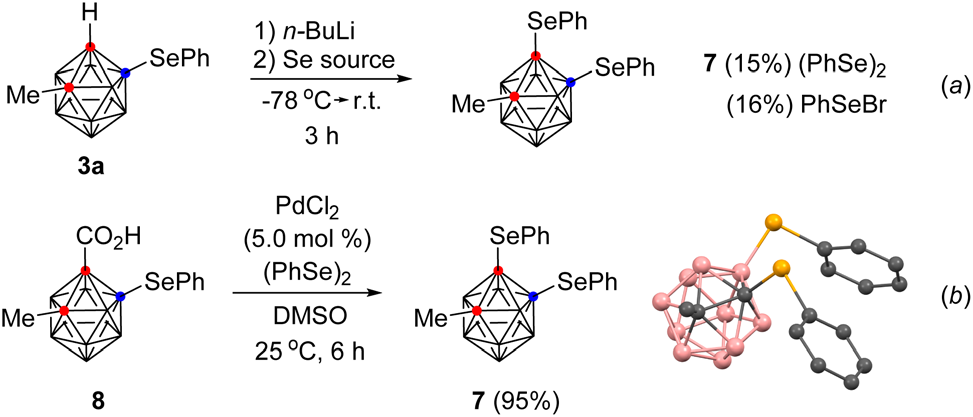
Comparison to efficiency of B(4)–C(1)-diselenylation.

The versatility of the current method was demonstrated in the synthesis of B(4)–C(1)-diselenyl *o*-carborane 7. When an *o*-carboranyl anion was generated *in situ* from 3a and *n*-butyl lithium and then treated with diphenyl diselenide or phenylselenyl bromide, the B(4)–C(1)-diselenyl *o*-carborane was obtained in low yield (Scheme 2, (a)). In contrast, the Pd-catalyzed decarboxylative C(1)-selenylation method provided the desired product (7) in 95% yield from B(4)-phenylselenyl *o*-carborane acid (8) (b). These results indicate that the Pd-catalyzed decarboxylative C(1)-selenylation is higher yielding than the previous approach under mild reaction conditions. A plausible reaction mechanism for Pd-catalyzed decarboxylative C(1)-selenylation is described in the ESI.†

**Scheme 3:**
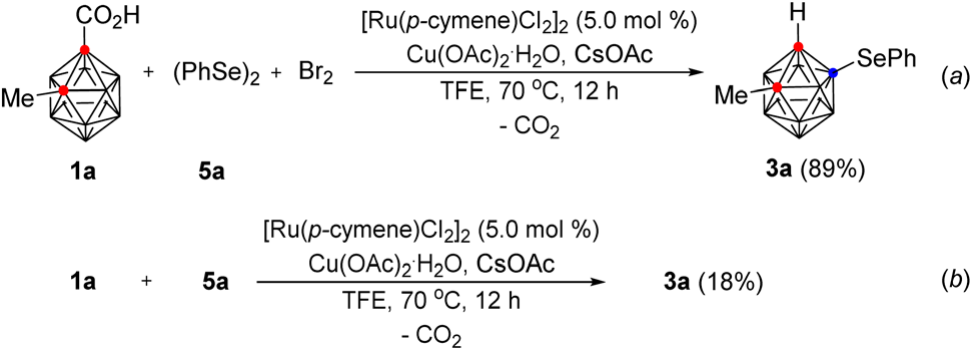
B(4)-Selenylation reaction using diphenyl diselenide and bromine.

Because 1a was smoothly reacted with phenylselenyl bromide in the presence of Ru(ii) to give 3a, we attempted to perform the same selenylation reaction with phenylselenyl bromide generated *in situ* from diphenyl diselenide and bromine in one-pot. This reaction gave 3a in 89% yield (Scheme 3, (a)). However, the reaction of 1a with diphenyl diselenide under the optimum reaction conditions and in the absence of bromine gave 3a in 18% yield (b).

**Fig. 1:**
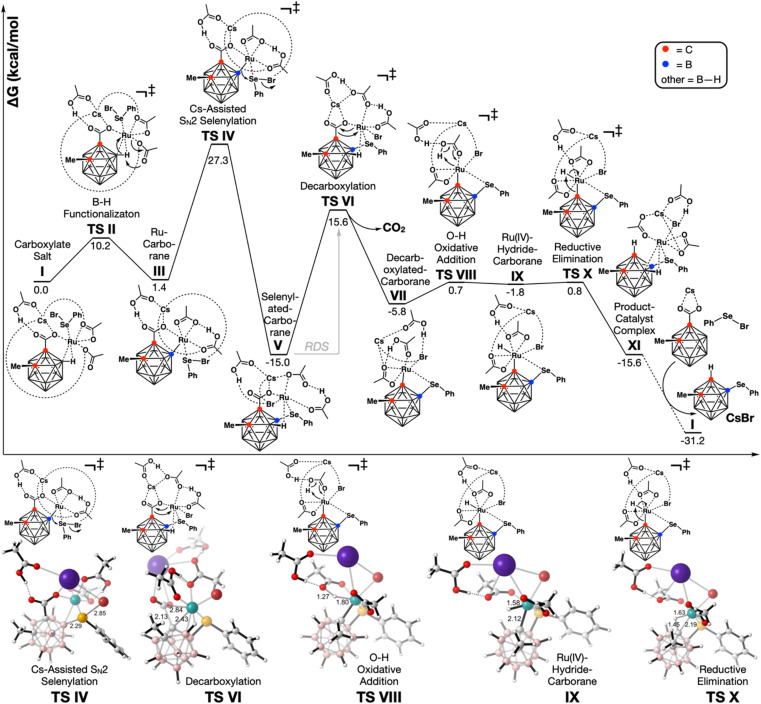
Computed reaction coordinate diagram for the Ru(ii)-catalyzed decarboxylative B(4)-selenylation of *o*-carboranes. Several ligand rearrangement steps have been omitted; non-covalent interactions have been drawn as accurately as possible, while attempting to maintain clarity.

To gain further insight into the more unusual Ru(ii)-catalyzed process, we turned to DFT. All structures were optimized at the PBE^16^/6-31G*^17^ level of theory with the LANL2DZ^18^ effective core potential for Ru, Cs, Br, and Se, in the gas phase, with the D3BJ^19^ empirical dispersion model (Fig. 1). Solvation single point energy refinements were performed with the SMD^20^ implicit solvation model for 2,2,2-trifluoroethanol (TFE) at the PBE-D3BJ/def2-QZVP^21^ level of theory. CsOAc was also assumed to convert 2-methyl *o*-carborane acid 1a into the analogous cesium salt. Furthermore, since it has previously been demonstrated that monometallic systems based on Ru(ii),^22^ Rh(i),^23^ and Pd(ii)^24^ effectively catalyze decarboxylations in the absence of Cu or Ag salts, we assumed that Cu(OAc)_2_ acts solely as a halogen abstractor to generate the active Ru(ii) catalyst.

Extensive exploration of the initial coordination complexes revealed that the Ru(ii) catalyst readily undergoes ligand exchange, losing the *p*-cymene ligand in favor of coordination with the cesium carboxylate salt of *o*-carborane 1a, phenylselenyl bromide 2a, and 2 equivalents of acetate to give complex I.

An agostic interaction between the B(4)–H bond of the *o*-carborane and Ru ostensibly facilitates deprotonation by a carboxylate (B–H functionalization TS II), leading to the Ru–carborane complex III. Deprotonation at B(4) is preferred over deprotonation at B(3) – the position adjacent to both the carboxyl and methyl group – by 1.7 kcal mol^−1^.

The nascent nucleophilic B–Ru bond then attacks the backside of the phenylselenyl bromide (TS IV), displacing bromide to the Ru catalyst, and leading to the irreversible formation of selenylated carborane complex V.

The decarboxylation of this complex (TS VI), which is the rate determining step, has an energy barrier of 30.6 kcal mol^−1^, and leads to the formation of the decarboxylated selenylated carborane–Ru complex VII. To our considerable surprise, we discovered that the direct protonolysis to form the product–catalyst complex XI is energetically disfavored compared to a stepwise process involving the formation (TS VIII) and reductive elimination (TS X) of Ru(iv) hydride carborane complex IX (TS VIII′ Δ*G*^‡^ = 14.3 kcal mol^−1^*vs.*TS X Δ*G*^‡^ = 6.6 kcal mol^−1^, see the ESI†).

We also investigated the possibility of decarboxylation occurring prior to *o*-carborane deprotonation. Extensive exploration of the ligand sphere of TS VI reveals that three interactions are key to achieving a reasonable barrier (see the ESI†): (i) B–H agostic interaction; (ii) Ru⋯Se coordination; and (iii) a Ru–Br bond. The barrier to decarboxylation of I is 53.9 kcal mol^−1^. We hypothesize that this enormous barrier hinges on the fact that the *o*-carborane has not yet been selenylated. Following selenylation of the cluster, the selenium ostensibly functions as a directing group. Thus, the selenium brings the Ru sufficiently close to the cluster that a B–H agostic interaction can form, and such that the Ru is more proximal to the C–C bond that must be broken in the decarboxylation TS. Indeed, in the decarboxylation TS of I, there is no agostic interaction and Ru is more distal to the decarboxylation event.

The B(4) regioselectivity over B(3) occurs naturally because of steric interactions with the carborane C(2)-substituent. The regioselectivity of selenylation for B(4) over C(1) in the Ru-catalyzed process is entirely governed by the need for Ru–selenium coordination for a reasonable decarboxylation barrier. In the absence of initial selenylation, and thus the absence of the directing Se interaction, the decarboxylation barrier is extraordinarily high (Δ*G*^‡^ > 53.9 kcal mol^−1^, see the ESI† for examples). This necessitates the selenylation of B(4) prior to the C(1)-decarboxylation event, which would naturally preclude selenylation on C(1). This is distinctly different from the Pd-process where the decarboxylation is noticeably facile, making the C(1) selenylation process preferable.

Overall, the turnover barrier is 30.6 kcal mol^−1^ (TS VI) and the exothermicity is −15.6 kcal mol^−1^ (XI). Catalyst transfer from the product to another equivalent of the starting material (XI to I) is another −15.6 kcal mol^−1^, indicating that there is no product inhibition.

## Conclusions

In summary, we have developed regiodivergent metal-catalyzed B(4)- and C(1)-selenylation reactions applicable to a wide range of *o*-carboranes. Ru(ii)-catalysis selectively generated B(4)-selenylated *o*-carboranes from *o*-carborane acids and arylselenyl bromides, with the release of carbon dioxide. In contrast, Pd(ii) catalysis exclusively formed C(1)-selenylated *o*-carboranes from the decarboxylative reaction of *o*-carborane acids with diaryl diselenides. The regioselectivity is controlled by the transition metal catalyst. Unlike previous milestones in this area, both transformations show broad substrate scope and high yield. These selenylation reactions are thus a highly efficient way to selectively introduce an organoselenium functionality onto the B(4)- and C(1)-positions of *o*-carboranes.

## Data availability

All of the related experimental and computational data are provided in the ESI.† Crystallographic data for compound 3c and 7 have been deposited in the Cambridge Crystallographic Data Centre under accession number CCDC 1950362 and CCDC 2055987.

## Author contributions

K. L., T. H. K., and H. C. N. performed the experimental work, which was directed by P. H. L. J. L. H. conducted the computational studies, which were directed by P. H.-Y. C. D. K. performed the X-ray crystal structure analysis. The manuscript was written by P. H. L. and P. H.-Y. C. with contributions from all authors.

## Conflicts of interest

There are no conflicts to declare.

## Supplementary Material

SC-014-D2SC05590B-s001
