# Supplementary material for: Regiodivergent metal-catalyzed B(4)- and C(1)-selenylation of o-carboranes
Source: Chem Sci. 2022 Dec 3;14(3):643–9. doi: 10.1039/d2sc05590b (PMC9847680; doi:10.1039/d2sc05590b)

Electronic Supplementary Material (ESI) for Chemical Science.
This journal is © The Royal Society of Chemistry 2022

Supporting Information

Regiodivergent Metal-Catalyzed B(4)- and C(1)-Selenylation of *o*-Carboranes

Kyungsup Lee,‡[a] Jordan L. Harper,‡[b] Tae Hyeon Kim,[a] Hee Chan Noh,[a] Dongwook Kim,[c] Paul Ha-Yeon Cheong,*[b] and Phil Ho Lee*[a]

[a]Department of Chemistry, Kangwon National University, Chuncheon 24341, Republic of Korea

[b]Department of Chemistry, Oregon State University, Corvallis, Oregon 97331, United State

[c]Department of Chemistry, Korea Advanced Institute of Science and Technology (KAIST), Daejeon 34141, Republic of Korea; Center for Catalytic Hydrocarbon Functionalizations, Institute for Basic Science (IBS), Daejeon 34141, Republic of Korea

*Corresponding authors. E-mail: phlee@kangwon.ac.kr and cheongh@oregonstate.edu

Table of Contents

1. Experimental Section……………………………………………………………………….. S3

2. General Procedure of the Ru-Catalyzed B(4)-Selenylation of *o*-Carboranes………………...... S4

3. Synthetic Method for B(4)-Selenylated Carborane on a Large Scale………………………….. S11

4. Reaction Optimization for C(1)-Selenylation of *o*-Carboranes………………………………… S12

5. General Procedure of the Pd-Catalyzed C(1)-Selenylation of *o*-Carboranes…………………. S13

6. Procedure for C(1)-B(4)-Selenylated *o*-Carborane……………………………………………... S17

7. Procedure for One-Pot Synthesis…………………………………………………………………. S19

8. A Proposed Mechanism for Pd-Catalyzed C(1)-Selenylation…………………………………... S20

9. X-Ray Crystallography…………………………………………………………………………… S21

10. References………………………………………………………………………………………… S25

11. Complete Authorship of Gaussian 16……………………………………………………………... S26

12. Complete Authorship of CYLview………………………………………………………………… S26

13. Discussion of DFT Methods and Results ………………………………………………...…... S27

14. Ground State Computed Geometries and Energies…………………………………………… S33

15. Transition State Computed Geometries and Energies……………………………………….. S162

16. Ground State Solvation Single Point Energies………………………………………………... S265

17. Transition State Solvation Single Point Energies…………………………………………….. S374

18. ^{1}H, ^{13}C, ^{11}B and ^{19}F NMR Data………………………………………………………………… S474

1. Experimental Section

Commercial available reagents were used without purification. All reaction mixtures were stirred magnetically and were monitored by thin-layer chromatography using silica gel pre-coated glass plates, which were visualized with UV light, and then developed using a solution of $KMnO_4$. For reactions that require heating, oil bath was used as the source of heating. Flash column chromatography was carried out using silica gel (230-400 mesh). ^{1}H NMR (400 MHz), ^{13}C{^{1}H} NMR (100 MHz), ^{11}B NMR (128 MHz), ^{11}B{^{1}H} NMR (128 MHz), and ^{19}F{^{1}H} NMR (377 MHz) spectra were recorded on NMR spectrometer. Deuterated chloroform was used as the solvent and chemical shift values (δ) are reported in parts per million relative to the residual signals of this solvent [δ 7.26 for ^{1}H (chloroform-*d*), δ 77.2 for ^{13}C{^{1}H} (chloroform-*d*)]. Infrared spectra were recorded on FT-IR spectrometer as either a thin film pressed between two sodium chloride plates or as a solid suspended in a potassium bromide disk. High resolution mass spectra (HRMS) was obtained by electron impact (EI) ionization technique (magnetic sector - electric sector double focusing mass analyzer) from the KBSI (Korea Basic Science Institute Daegu Center) and performed on the Synapt G2-HDMS mass spectrometer (Waters, Manchester, U.K.), which was operated on the MassLynx 4.1 software from the KBSI (Korea Basic Science Institute, Ochang, Center of Research Equipment). Melting points were determined in open capillary tube. Single-crystal X-ray diffraction data were collected on a Bruker D8 QUEST with coated Parabar oil under N_2 gas stream at 173 K. The diffraction data were integrated, scaled, and reduced by using the Bruker APEX3 software. The crystal structures were solved by the SHELX structure solution program and refined by full-matrix least-squares calculations with the SHELXL. See the reference for synthesis of starting material.[1-7]

2. General Procedure of the Ru-Catalyzed B(4)-Selenylation of *o*-Carboranes

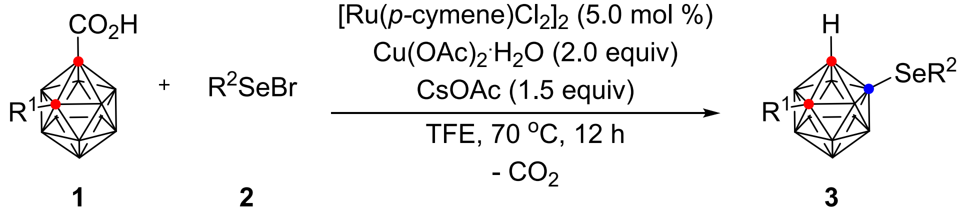

A V-vial equipped with a magnetic stirrer was charged with carboxylated *o*-carborane **1** (0.1 mmol, 1.0 equiv), selenyl bromide **2** (1.5 equiv), [Ru(*p*-cymene)Cl_2]$_2$ (5.0 mol %), Cu(OAc)$_2$·H_2O (2.0 equiv), and CsOAc (1.5 equiv) in TFE (1.0 mL) and stirred at 70 °C for 12 h under air. The reaction mixture was filtered through a pad of silica gel and washed with ethyl acetate. Then, the mixture was concentrated under reduced pressure. The crude product was purified by column chromatography on silica gel to afford product **3**.

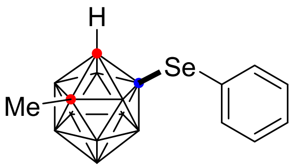

3a: Yield: 28.5 mg (91%); R_f = 0.30 (CH_2Cl_2:Hexane = 1:20); White solid; Melting point: 62-64 °C; ^{1}H NMR (400 MHz, $CDCl_3$) δ 7.61-7.59 (m, 2H), 7.33-7.29 (m, 1H), 7.27-7.23 (m, 2H), 3.76 (s, 1H), 2.01 (s, 3H); ^{13}C{^{1}H} NMR (100 MHz, $CDCl_3$) δ 136.3, 129.2, 128.0, 127.9, 72.7, 64.4, 25.9; ^{11}B{^{1}H} NMR (128 MHz, $CDCl_3$) δ -0.77 (1B), -5.03 (1B), -6.16 (1B), -7.97 (1B), -9.22 (1B), -10.78 (3B), -11.65 (1B), -12.83 (1B); IR (film): 2574, 2360, 2343, 1576, 1474, 1436, 1021, 974, 831 cm^{-1}; HRMS (ESI) *m/z*: $[M]^+$ Calcd for $C_9H_{18}B_{10}Se$ 316.1504; Found 316.1503.

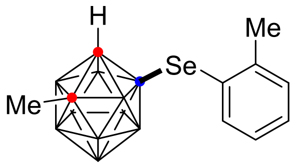

3b: Yield: 30.6 mg (93%); R_f = 0.30 (CH_2Cl_2:Hexane = 1:20); White solid; Melting point: 58-60 °C; ^{1}H NMR (400 MHz, $CDCl_3$) δ 7.63 (d, J = 7.56 Hz, 1H), 7.25-7.20 (m, 2H), 7.09-7.04 (m, 1H), 3,75 (s, 1H), 2.50 (s, 3H), 2.01 (s, 3H); ^{13}C{^{1}H} NMR (100 MHz, $CDCl_3$) δ 142.4, 138.0, 130.1, 129.3, 128.5, 126.5, 72.6, 64.3, 25.9, 24.0; ^{11}B{^{1}H} NMR (128 MHz, $CDCl_3$) δ -0.91 (1B), -5.06 (1B), -6.21 (1B), -8.05 (1B), -9.23 (1B), -10.77 - -12.91 (5B); IR (film): 2575, 2360, 2343, 1465, 1456, 1275, 1261, 943, 831 cm^{-1}; HRMS (EI) *m/z*: $[M]^+$ Calcd for $C_{10}H_{20}B_{10}Se$ 330.1661; Found 330.1658.

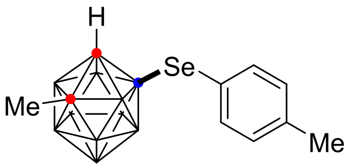

3c: Yield: 32.1 mg (98%); R_f = 0.30 (CH_2Cl_2:Hexane = 1:15); White solid; Melting point: 64-66 °C; ^{1}H NMR (400 MHz, $CDCl_3$) δ 7.46 (d, J = 7.96 Hz, 2H), 7.06 (d, J = 7.84 Hz, 2H), 3.74 (s, 1H), 2.32 (s, 3H), 1.99 (s, 3H); $^{13}C\{^{1}H\}$ NMR (100 MHz, $CDCl_3$) δ 137.8, 136.2, 130.0, 124.3, 72.6, 64.4, 25.9, 21.3; $^{11}B\{^{1}H\}$ NMR (128 MHz, $CDCl_3$) δ -0.84 (1B), -5.02 (1B), -6.11 (1B), -7.98 (1B), -9.24 (1B, -10.75 - -12.90 (5B); IR (film): 2573, 2362, 2345, 1467, 1453, 1245, 1457, 1263, 943 cm^{-1}; HRMS (ESI) *m/z*: [M-H]$^-$ Calcd for $C_{10}H_{19}B_{10}Se$ 329.1583; Found 329.1580.

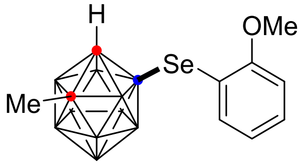

3d: Yield: 28.7 mg (83%); R_f = 0.35 (CH_2Cl_2:Hexane = 1:10); White solid; Melting point: 74-76 °C; ^{1}H NMR (400 MHz, $CDCl_3$) δ 7.65 (dd, J_1 = 7.44 Hz, J_2 = 1.68 Hz, 1H), 7.33-7.29 (m, 1H), 6.89-6.85 (m, 2H), 3.88 (s, 1H), 3.86 (s, 3H), 2.01 (s, 3H); $^{13}C\{^{1}H\}$ NMR (100 MHz, $CDCl_3$) δ 159.7, 138.3, 129.9, 121.4, 116.9, 111.0, 72.5, 64.6, 55.9, 25.9; $^{11}B\{^{1}H\}$ NMR (128 MHz, $CDCl_3$) δ -0.78 (1B), -4.97 (1B), -6.49 (1B), -7.91 (1B), -9.56 (1B), -10.91 - -12.94 (5H); IR (film): 2574, 2361, 2343, 1578, 1473, 1464, 1431, 1288, 1270 cm^{-1}; HRMS (EI) *m/z*: [M]$^+$ Calcd for $C_{10}H_{20}B_{10}OSe$ 346.1610; Found 346.1612.

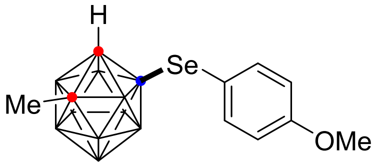

3e: Yield: 17.5 mg (51%); R_f = 0.35 (CH_2Cl_2:Hexane = 1:10); White solid; Melting point: 79-81 °C; ^{1}H NMR (400 MHz, $CDCl_3$) δ 7.48 (d, J = 8.84 Hz, 2H), 6.80 (d, J = 8.84 Hz, 2H), 3.80 (s, 3H), 3.73 (s, 1H), 2.00 (s, 3H); $^{13}C\{^{1}H\}$ NMR (100 MHz, $CDCl_3$) δ 159.6, 137.6, 118.3, 114.9, 72.6, 64.3, 55.4, 25.9; $^{11}B\{^{1}H\}$ NMR (128 MHz, $CDCl_3$) δ -0.86 (1B), -5.04 (1B), -6.06 (1B), -8.00 (1B), -9.25 (1B), -10.81 - -12.95 (5B); IR (film): 2580, 2360, 2343, 1590, 1488, 1457, 1276, 1260, 1244 cm^{-1}; HRMS (ESI) *m/z*: [M-H]$^-$ Calcd for $C_{10}H_{19}B_{10}OSe$ 345.1532; Found 345.1553.

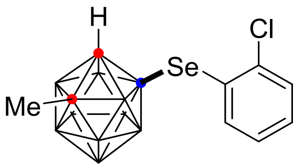

3f: Yield: 18.7 mg (54%); R_f = 0.30 (CH_2Cl_2:Hexane = 1:30); White solid; Melting point: 56-58 °C; ^{1}H NMR (400 MHz, $CDCl_3$) δ 7.603-7.595 (m, 1H), 7.48 (d, J = 7.64 Hz, 1H), 7.30-7.28 (m, 1H), 7.19 (t, J = 7.86 Hz, 1H), 3.81 (s, 1H), 2.03 (s, 3H); $^{13}C\{^{1}H\}$ NMR (100 MHz, $CDCl_3$) δ 135.9, 134.4, 134.3, 130.2, 129.3, 128.2, 72.8, 64.5, 25.9; $^{11}B\{^{1}H\}$ NMR (128 MHz, $CDCl_3$) δ -0.65 (1B), -5.02 (1B), -6.39

(1B), -8.01 (1B), -9.15 (1B), -10.76 - -12.61 (5B); IR (film): 2584, 2361, 2343, 1275, 1263, 763, 749, 703, 680 cm^{-1}; HRMS (EI) *m/z*: $[M]^+$ Calcd for $C_9H_{17}B_{10}ClSe$ 350.1115; Found 350.1116.

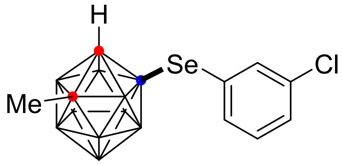

3g: Yield: 25.2 mg (72%); R_f = 0.35 (CH_2Cl_2:Hexane = 1:30); White solid; Melting point: 64-66 °C; ^{1}H NMR (400 MHz, $CDCl_3$) δ 7.76 (dd, J_1 = 7.68 Hz, J_2 = 1.48 Hz, 1H), 7.44 (dd, J_1 = 7.94 Hz, J_2 = 1.18 Hz, 1H), 7.28-7.24 (m, 1H), 7.14 (td, J_1 = 11.29 Hz, J_2 = 1.28 Hz, 1H), 3.97 (s, 1H), 2.03 (s, 3H); ^{13}C{^{1}H} NMR (100 MHz, $CDCl_3$) δ 139.6, 139.1, 129.7, 129.6, 128.6, 127.4, 72.7, 64.5, 25.9; ^{11}B{^{1}H} NMR (128 MHz, $CDCl_3$) δ -0.59 (1B), -4.96 (1B), -6.36 (1B), -8.00 (1B), -9.43 (1B), -10.86 - -12.60 (5B); IR (film): 2582, 2360, 2343, 1566, 1457, 1275, 1261, 832, 763 cm^{-1}; HRMS (ESI) *m/z*: $[M-H]^-$ Calcd for $C_9H_{16}B_{10}ClSe$ 349.1036; Found 349.1063.

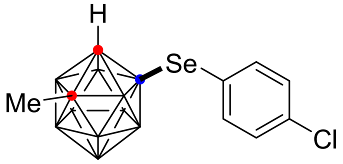

3h: Yield: 17.9 mg (51%); R_f = 0.30 (CH_2Cl_2:Hexane = 1:10); Yellow solid; Melting point: 76-78 °C; ^{1}H NMR (400 MHz, $CDCl_3$) δ 7.51 (d, J = 8.28 Hz, 2H), 7.22 (d, J = 8.32 Hz, 2H), 3.79 (s, 1H), 2.02 (s, 3H); ^{13}C{^{1}H} NMR (100 MHz, $CDCl_3$) δ 137.6, 134.3, 129.4, 126.1, 72.8, 64.4, 25.9; ^{11}B{^{1}H} NMR (128 MHz, $CDCl_3$) δ -0.69 (1B), -5.07 (1B), -6.36 (1B), -8.03 (1B), -9.17 (1B), -10.77 - -12.70 (5B); IR (film): 2576, 2361, 2343, 1472, 1275, 1260, 1088, 1011, 943 cm^{-1}; HRMS (EI) *m/z*: $[M]^+$ Calcd for $C_9H_{17}B_{10}ClSe$ 350.1115; Found 350.1118.

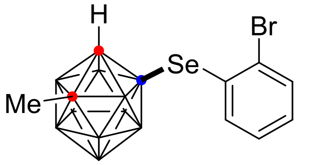

3i: Yield: 23.6 mg (60%); R_f = 0.40 (CH_2Cl_2:Hexane = 1:10); White solid; Melting point: 66-68 °C; ^{1}H NMR (400 MHz, $CDCl_3$) δ 7.79 (dd, J_1 = 7.42 Hz, J_2 = 1.90 Hz, 1H), 7.63 (dd, J_1 = 7.62 Hz, J_2 = 1.70 Hz, 1H), 7.23-2.14 (m, 2H), 4.02 (s, 1H), 2.03 (s, 3H); ^{13}C{^{1}H} NMR (100 MHz, $CDCl_3$) δ 139.0, 133.0, 131.1, 129.8, 128.0, 72.7, 64.4, 26.0; ^{11}B{^{1}H} NMR (128 MHz, $CDCl_3$) δ -0.62 (1B), -4.97 (1B), -6.23 (1B), -8.03 (1B), -9.47 (1B), -10.88 - -12.54(5B); IR (film): 2575, 2360, 2343, 1440, 1426, 1275, 1260, 1012, 943 cm^{-1}; HRMS (EI) *m/z*: $[M]^+$ Calcd for $C_9H_{17}B_{10}SeBr$ 394.0609; Found 394.0614.

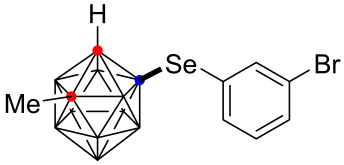

3j: Yield: 27.6 mg (70%); R_f = 0.35 (CH_2Cl_2:Hexane = 1:10); White solid; Melting point: 87-89 °C; ^{1}H NMR (400 MHz, $CDCl_3$) δ 7.76 (t, J = 1.58 Hz,

1H), 7.53 (d, J = 7.72 Hz, 1H), 7.45-7.43 (m, 1H), 7.13 (t, J = 7.86 Hz, 1H), 3.81 (s, 1H), 2.03 (s, 3H); $^{13}C\{^1H\}$ NMR (100 MHz, $CDCl_3$) δ 138.6, 134.9, 131.0, 130.5, 129.7, 122.5, 72.8, 64.5, 25.9; $^{11}B\{^1H\}$ NMR (128 MHz, $CDCl_3$) δ -0.66 (1B), -5.05 (1B), -6.41 (1B), -8.05 (1B), -9.16 (1B), -10.77 - -12.59 (5B); IR (film): 2574, 2360, 2343, 1559, 1541, 1455, 1390, 1275, 1261 cm^{-1}; HRMS (EI) *m/z*: $[M]^+$ Calcd for $C_9H_{17}B_{10}BrSe$ 394.0609; Found 394.0612.

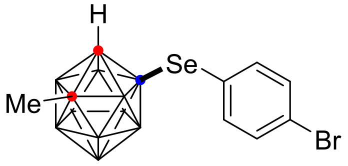

3k: Yield: 23.9 mg (61%); R_f = 0.30 (CH_2Cl_2:Hexane = 1:10); White solid; Melting point: 87-79 °C; ^1H NMR (400 MHz, $CDCl_3$) δ 7.45 (d, J = 8.40 Hz, 2H), 7.37 (d, J = 8.44 Hz, 2H), 3.79 (s, 1H), 2.02 (s, 3H); $^{13}C\{^1H\}$ NMR (100 MHz, $CDCl_3$) δ 137.9, 132.3, 126.8, 122.5, 72.8, 64.4, 25.9; $^{11}B\{^1H\}$ NMR (128 MHz, $CDCl_3$) δ -0.70 (1B), -5.06 (1B), -6.44 (1B), -8.03 (1B), -9.17 (1B), -10.78 - -12.67 (5B); IR (film): 2580, 2360, 2343, 1559, 1465, 1456, 1275, 1260, 1073 cm^{-1}; HRMS (EI) *m/z*: $[M]^+$ Calcd for $C_9H_{17}B_{10}SeBr$ 394.0609; Found 394.0607.

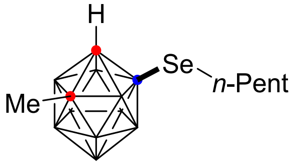

3l: Yield: 10.8 mg (35%); R_f = 0.35 (CH_2Cl_2:Hexane = 1:10); White solid; Melting point: 64-66 °C; ^1H NMR (400 MHz, $CDCl_3$) δ 3.81 (s, 1H), 2.64 (t, J = 7.46 Hz, 2H), 2.06 (s, 3H), 1.68 (quint, J = 7.40 Hz, 2H), 1.37-1.31 (m, 4H), 0.90 (t, J = 7.08 Hz, 3H); $^{13}C\{^1H\}$ NMR (100 MHz, $CDCl_3$) δ 72.4, 64.9, 32.2, 31.8, 26.0, 25.6, 22.3, 14.1; $^{11}B\{^1H\}$ NMR (128 MHz, $CDCl_3$) δ -0.97 (1B), -5.16 (1B), -6.60 (1B), -7.95 (1B), -9.18 (1B), -10.86 - -12.92 (5B); IR (film): 2956, 2926, 2870, 2856, 2575, 1463, 943, 817, 724 cm^{-1}; HRMS (EI) *m/z*: $[M]^+$ Calcd for $C_8H_{24}B_{10}Se$ 310.1974; Found 310.1971.

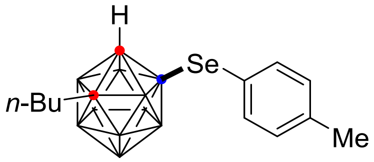

3m: Yield: 37.4 mg (98%); R_f = 0.30 (CH_2Cl_2:Hexane = 1:20); Yellow solid; Melting point: 83-85 °C; ^1H NMR (400 MHz, $CDCl_3$) δ 7.47 (d, J = 8.04 Hz, 2H), 7.06 (d, J = 7.76 Hz, 2H), 3.73 (s, 1H), 2.33 (s, 3H), 2.18-2.14 (m, 2H), 1.41-1.34 (m, 2H), 1.31-1.21 (m, 2H), 0.88 (t, J = 7.24 Hz, 3H); $^{13}C\{^1H\}$ NMR (100 MHz, $CDCl_3$) δ 137.7, 136.2, 130.0, 124.3, 77.8, 63.8, 37.9, 31.4, 22.2, 21.3, 13.7; $^{11}B\{^1H\}$ NMR (128 MHz, $CDCl_3$) δ -1.53 (1B), -4.25 (1B), -6.59 (1B), -8.23 (1B), -9.42 (1B), -11.70 - -13.62 (5B); IR (film): 2582, 2360, 2343, 1275, 1260, 803, 763, 750, 725 cm^{-1}; HRMS (ESI) *m/z*: $[M-H]^-$ Calcd for $C_{13}H_{25}B_{10}Se$ 371.2052; Found 371.2080.

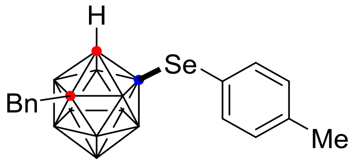

3n: Yield: 36.7 mg (91%); R_f = 0.30 (CH_2Cl_2:Hexane = 1:20); White solid; Melting point: 59-61 °C; ^{1}H NMR (400 MHz, $CDCl_3$) δ 7.36-7.32 (m, 5H), 7.06-7.00 (m, 4H), 3.45 (s, 2H), 3.32 (s, 1H), 2.33 (s, 3H); $^{13}C\{^1H\}$ NMR (100 MHz, $CDCl_3$) δ 137.7, 136.2, 134.2, 130.0, 129.8, 129.2, 128.6, 124.0, 61.8, 43.5, 21.3; $^{11}B\{^1H\}$ NMR (128 MHz, $CDCl_3$) δ -1.64 (1B), -4.08 (1B), -6.57 (1B), -7.77 (1B), -9.07 (1B), -10.84 - -11.96 (5B); IR (film): 2572, 2361, 2343, 1487, 1455, 1437, 1275, 1015, 976 cm^{-1}; HRMS (ESI) *m/z*: $[M-H]^-$ Calcd for $C_{16}H_{23}B_{10}Se$ 405.1896; Found 405.1923.

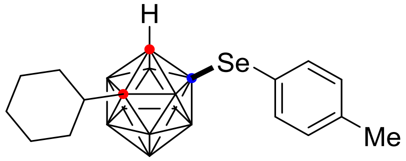

3o: Yield: 36.0 mg (91%); R_f = 0.30 (CH_2Cl_2:Hexane = 1:20); White solid; Melting point: 85-87 °C; ^{1}H NMR (400 MHz, $CDCl_3$) δ 7.47-7.45 (m, 2H), 7.06 (d, J = 7.80 Hz, 2H), 3.83 (s, 1H), 2.32 (s, 3H), 2.08-2.00 (m, 1H), 1.85-1.79 (m, 4H), 1.67-1.63 (m, 1H), 1.21-1.14 (m, 2H), 1.10-1.03 (m, 3H); $^{13}C\{^1H\}$ NMR (100 MHz, $CDCl_3$) δ 137.7, 136.2, 130.0, 124.3, 83.6, 62.2, 43.5, 33.2, 26.4, 25.2, 21.3; $^{11}B\{^1H\}$ NMR (128 MHz, $CDCl_3$) δ -2.32 (1B), -3.35 (1B), -7.13 (1B), -8.37 (1B), -9.50 (1B), -11.79 - -13.83 (5B); IR (film): 2930, 2856, 2574, 2340, 1717, 1698, 1487, 1396, 1275 cm^{-1}; HRMS (EI) *m/z*: $[M]^+$ Calcd for $C_{15}H_{28}B_{10}Se$ 398.2287; Found 398.2283.

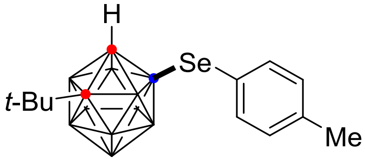

3p: Yield: 27.4 mg (74%); R_f = 0.30 (CH_2Cl_2:Hexane = 1:20); White solid; Melting point: 84-86 °C; ^{1}H NMR (400 MHz, $CDCl_3$) δ 7.47 (d, J = 8.00 Hz, 2H), 7.07 (d, J = 7.76 Hz, 2H), 3.88 (s, 1H), 2.33 (s, 3H), 1.18 (s, 9H); $^{13}C\{^1H\}$ NMR (100 MHz, $CDCl_3$) δ 137.8, 136.3, 130.0, 124.3, 88.4, 64.4, 36.7, 32.0, 21.3; $^{11}B\{^1H\}$ NMR (128 MHz, $CDCl_3$) δ -2.78 (2B), -7.11 (1B), -7.76 (1B), -8.82 (1B), -11.28 (1B), -12.62 - -13.27 (4B); IR (film): 2615, 2570, 2360, 2343, 1487, 1479, 1473, 1457, 1370 cm^{-1}; HRMS (EI) *m/z*: $[M]^+$ Calcd for $C_{13}H_{26}B_{10}Se$ 372.2130; Found 372.2132.

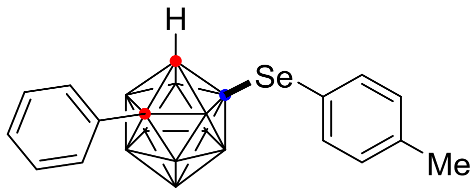

3q: Yield: 28.3 mg (74%); R_f = 0.35 (CH_2Cl_2:Hexane = 1:20); White solid; Melting point: 84-86 °C; ^{1}H NMR (400 MHz, $CDCl_3$) δ 7.49 (d, J = 7.2 Hz, 2H), 7.43-7.37 (m, 3H), 7.33-7.29

(m, 2H), 7.06 (d, J = 7.9 Hz, 2H), 4.13 (s, 1H), 2.32 (s, 3H); $^{13}C\{^1H\}$ NMR (100 MHz, $CDCl_3$) δ 137.8, 136.2, 133.1, 130.2, 130.1, 129.0, 127.6, 124.3, 78.9, 63.0, 21.3; $^{11}B\{^1H\}$ NMR (128 MHz, $CDCl_3$) δ -1.21 (1B), -2.71 (1B), -6.02 (1B), -7.89 (1B), -9.01 (1B), -10.95 - -12.99 (5B); IR (film): 2582, 2360, 2343, 1275, 1260, 803, 763, 750, 690 cm^{-1}; HRMS (ESI) *m/z*: $[M-H]^-$ Calcd for $C_{15}H_{21}B_{10}Se$ 391.1739; Found 391.1753.

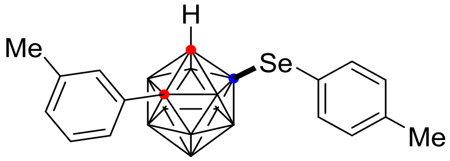

3r: Yield: 31.1 mg (77%); R_f = 0.30 (CH_2Cl_2:Hexane = 1:20); Yellow solid; Melting point: 92-94 °C; ^1H NMR (400 MHz, $CDCl_3$) δ 7.50 (d, J = 7.8 Hz, 2H), 7.22-7.20 (m, 4H), 7.08 (d, J = 7.7 Hz, 2H), 4.12 (s, 1H), 2.35 (s, 3H), 2.34 (s, 3H); $^{13}C\{^1H\}$ NMR (100 MHz, $CDCl_3$) δ 138.9, 137.8, 136.3, 133.1, 130.9, 130.1, 128.9, 128.2, 124.7, 124.3, 79.0, 62.9, 21.5, 21.3; $^{11}B\{^1H\}$ NMR (128 MHz, $CDCl_3$) δ -1.4 (1B), -2.8 (1B), -6.1 (1B), -7.9 (1B), -9.0 (1B), -10.9 - -13.0 (5B); IR (film): 2575, 2360, 2343, 1487, 1275, 1261, 803, 764, 750 cm^{-1}; HRMS (EI) *m/z*: $[M]^+$ Calcd for $C_{16}H_{24}B_{10}Se$ 406.1974; Found 406.1976.

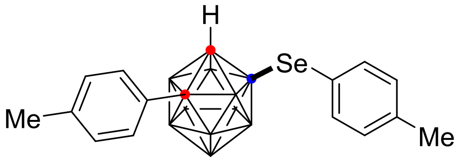

3s: Yield: 33.9 mg (84%); R_f = 0.35 (CH_2Cl_2:Hexane = 1:20); White solid; Melting point: 78-80 °C; ^1H NMR (400 MHz, $CDCl_3$) δ 7.48 (d, J = 8.0 Hz, 2H), 7.29 (d, J = 8.4 Hz, 2H), 7.10 (d, J = 8.1 Hz, 2H), 7.06 (d, J = 7.8 Hz, 2H), 4.08 (s, 1H), 2.325 (s, 3H), 2.319 (s, 3H); $^{13}C\{^1H\}$ NMR (100 MHz, $CDCl_3$) δ 140.5, 137.8, 136.2, 130.3, 130.1, 129.6, 127.6, 124.3, 79.1, 63.2, 21.3, 21.1; $^{11}B\{^1H\}$ NMR (128 MHz, $CDCl_3$) δ -1.25 (1B), -2.95 (1B), -6.08 (1B), -7.98 (1B), -9.10 (1B), -10.96 - -12.97 (5B); IR (film): 2582, 2360, 2343, 1540, 1456, 1275, 1260, 831, 763 cm^{-1}; HRMS (EI) *m/z*: $[M]^+$ Calcd for $C_{16}H_{24}B_{10}Se$ 406.1974; Found 406.1971.

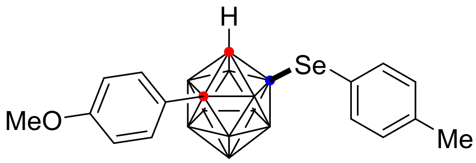

3t: Yield: 34.4 mg (82%); R_f = 0.35 (CH_2Cl_2:Hexane = 1:20); White solid; Melting point: 89-91 °C; ^1H NMR (400 MHz, $CDCl_3$) δ 7.48 (d, J = 7.9 Hz, 2H), 7.35 (d, J = 8.9 Hz, 2H), 7.06 (d, J = 7.8 Hz, 2H), 6.80 (d, J = 8.9 Hz, 2H), 4.04 (s, 1H), 3.79 (s, 3H), 2.32 (s, 3H); $^{13}C\{^1H\}$ NMR (100 MHz, $CDCl_3$) δ 161.0, 137.8, 136.2, 130.1, 129.4, 125.2, 124.3, 114.2, 79.3, 63.8, 55.6, 21.3; $^{11}B\{^1H\}$ NMR (128 MHz, $CDCl_3$) δ -1.12 (1B), -3.09 (1B), -6.02 (1B), -8.08 (1B), -9.21 (1B), -11.06 - -12.74

(5B); IR (film): 2571, 2360, 2343, 1608, 1559, 1540, 1509, 1487, 1439 cm^{-1}; HRMS (EI) *m/z*: $[M]^+$ Calcd for $C_{16}H_{24}B_{10}OSe$ 422.1923; Found 422.1920.

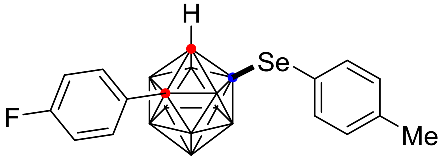

3u: Yield: 29.0 mg (71%); R_f = 0.30 (CH_2Cl_2:Hexane = 1:10); White solid; Melting point: 92-94 °C; ^1H NMR (400 MHz, $CDCl_3$) δ 7.48 (d, *J* = 8.0 Hz, 2H), 7.44-7.39 (m, 2H), 7.07 (d, *J* = 7.8 Hz, 2H), 7.03-6.98 (m, 2H), 4.07 (s, 1H), 2.32 (s, 3H); $^{13}C\{^1H\}$ NMR (100 MHz, $CDCl_3$) δ 163.7 (d, *J* = 252.2 Hz), 137.9, 136.2, 130.1, 129.9 (d, *J* = 8.7 Hz), 129.1 (d, *J* = 3.4 Hz), 124.2, 116.1 (d, *J* = 22.0 Hz), 78.2, 63.4, 21.3; $^{11}B\{^1H\}$ NMR (128 MHz, $CDCl_3$) δ -1.01 (1B), -2.75 (1B), -5.92 (1B), -7.88 (1B), -8.99 (1B), -11.06 - -12.88 (5B); ^{19}F NMR (376 MHz, $CDCl_3$) δ -110.2; IR (film): 2575, 2360, 2343, 1540, 1508, 1487, 1275, 1241, 1167 cm^{-1}; HRMS (ESI) *m/z*: $[M-H]^-$ Calcd for $C_{15}H_{20}B_{10}FSe$ 409.1645; Found 409.1645.

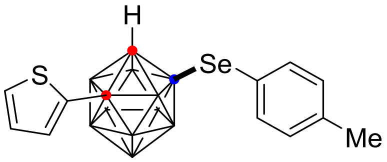

3v: Yield: 26.9 mg (68%); R_f = 0.30 (CH_2Cl_2:Hexane = 1:20); Yellow solid; Melting point: 88-90 °C; ^1H NMR (400 MHz, $CDCl_3$) δ 7.48-7.46 (m, 2H), 7.24 (dd, *J* = 5.2 Hz, *J* = 1.3 Hz, 1H), 7.14 (dd, *J* = 3.7 Hz, *J* = 1.3 Hz, 1H), 7.07 (d, *J* = 7.8 Hz, 2H), 6.89 (dd, *J* = 5.2 Hz, *J* = 3.7 Hz, 1H), 4.02 (s, 1H), 2.33 (s, 3H); $^{13}C\{^1H\}$ NMR (100 MHz, $CDCl_3$) δ 137.9, 136.5, 136.2, 130.2, 130.1, 128.2, 127.4, 124.1, 74.4, 65.8, 21.3; $^{11}B\{^1H\}$ NMR (128 MHz, $CDCl_3$) δ -0.65 (1B), -3.00 (1B), -5.86 (1B), -8.29 (1B), -9.38 (1B), -10.79 - -11.63 (5B); IR (film): 2572, 2360, 2343, 1540, 1507, 1487, 1456, 1428, 1275 cm^{-1}; HRMS (ESI) *m/z*: $[M-H]^-$ Calcd for $C_{13}H_{19}B_{10}SSe$ 397.1303; Found 397.1309.

3. Synthetic Method for B(4)-Selenylated Carborane on a Large Scale

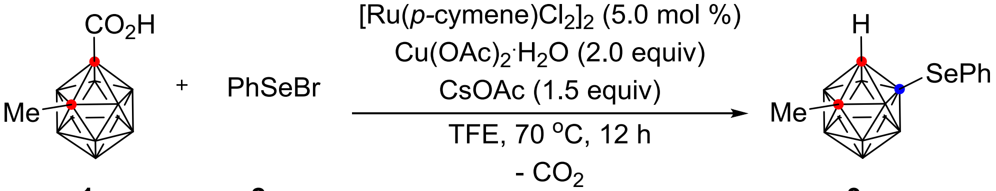

1a **2a** **3a**

A Schlenk flask equipped with a magnetic stirrer was charged with carboxylated *o*-carborane **1a** (1.21 g, 6.0 mmol, 1.0 equiv), arylselenyl bromide **2a** (1.5 equiv), [Ru(*p*-cymene)Cl_2]$_2$ (5.0 mol %), $Cu(OAc)_2 \cdot H_2O$ (2.0 equiv), and CsOAc (1.5 equiv) in TFE (60 mL) and stirred at 70 °C for 12 h under air. The reaction mixture was filtered through a pad of silica gel and washed with ethyl acetate. Then, the mixture was concentrated under reduced pressure. The crude product was purified by column chromatography on silica gel to afford product **3a** (1.84 g, 97%).

4. Reaction Optimization for C(1)-Selenylation of *o*-Carboranes[a]

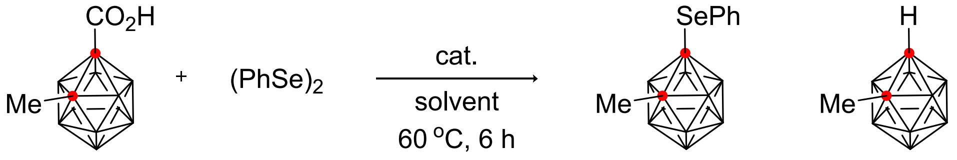

1a **5a** **6a** **4**

entry	cat. (5 mol %)	solvent	yield (%)[a]	
			**6a**	**4**
1	$PdCl_2(NCPh)_2$	DMSO	90	5
2	$Pd(PPh_3)_4$	DMSO	54	26
3	$Pd(OAc)_2$	DMSO	90	5
4	$Pd(TFA)_2$	DMSO	84	0
5	$PdBr_2$	DMSO	93	7
6	$PdCl_2(PPh_3)_2$	DMSO	81	7
**7**	**$PdCl_2$**	**DMSO**	**97**	**3**
8	$PdCl_2$	DMF	17	53
9	$PdCl_2$	toluene	0	0
10	$PdCl_2$	DCE	0	0
11	$PdCl_2$	TFE	0	0
12[b]	$PdCl_2$	DMSO	52	9

[a]Reaction conditions: **1a** (0.1 mmol, 1.0 equiv), **5a** (1.2 equiv), and cat. (5 mol %) in solvent (0.5 mL, 0.20 M) were stirred in a test tube at 60 °C for 6 h. [b]NMR yield using CH_2Br_2 as an internal standard. [b]Phenylselenyl bromide **2a** (2.0 equiv) was used instead of **5a**.

5. General Procedure of the Pd-Catalyzed C(1)-Selenylation of *o*-Carboranes

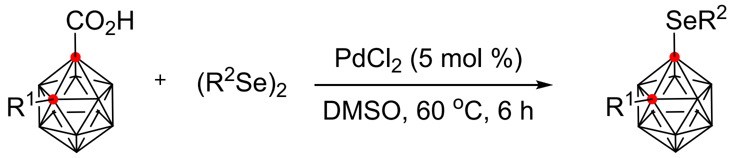

1 **5** **6**

A test tube equipped with a magnetic stirrer was charged with carboxylated *o*-carborane **1** (0.1 mmol, 1.0 equiv), diselenide **5** (1.2 equiv), and $PdCl_2$ (5.0 mol %) in DMSO (0.5 mL) and stirred at 60 °C for 6 h under N_2. After the mixture was cooled to room temperature, saturated sodium thiosulfate solution (5 mL) was added to quench the reaction. The mixture was extracted with dichloromethane (5 mL) three times. The combined organic layers were washed with water and dried over anhydrous $MgSO_4$. After the volatiles were evaporated, the crude product was purified by column chromatography on silica gel to afford product **6**.

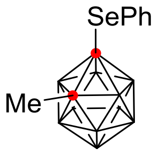

6a: Yield: 30.4 mg (97%); R_f = 0.45 (Hexane); White solid; Melting point: 128-130 °C; ^{1}H NMR (400 MHz, $CDCl_3$) δ 7.67 (t, J = 6.92 Hz, 2H), 7.51 (t, J = 7.44 Hz, 1H), 7.41 (t, J = 7.48 Hz, 2H), 2.18 (s, 3H); ^{13}C{^{1}H} NMR (100 MHz, $CDCl_3$) δ; 138.0, 131.2, 129.8, 127.5, 77.7, 69.3, 25.2;^{11}B{^{1}H} NMR (128 MHz, $CDCl_3$) δ -3.13 (1B), -4.74 (1B), -8.62 (1B), -9.58 (7B); IR (film): 2606, 2595, 2565, 2545, 1439, 742, 727, 689, 503 cm^{-1}; HRMS (EI) *m/z*: $[M]^+$ Calcd for $C_9H_{18}B_{10}Se$ 316.1504; Found 316.1503.

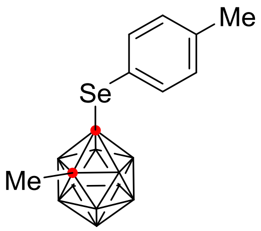

6b: Yield: 30.5 mg (93%); R_f = 0.40 (Hexane); White solid; Melting point: 74-76 °C; ^{1}H NMR (400 MHz, $CDCl_3$) δ 7.53 (d, J = 8.00 Hz, 2H), 7.20 (d, J = 8.08 Hz, 2H), 2.41 (s, 3H), 2.17 (s, 3H); ^{13}C{^{1}H} NMR (100 MHz, $CDCl_3$) δ 141.7, 137.9, 130.6, 124.2, 77.7, 69.6, 25.2, 21.6; ^{11}B{^{1}H} NMR (128 MHz, $CDCl_3$) δ -3.17 (1B), -4.71 (1B), -8.62 (2B), -9.54 (6B); IR (film): 2576, 1487, 1442, 1381, 1181, 1040, 1015, 800, 725 cm^{-1}; HRMS (EI) *m/z*: $[M]^+$ Calcd for $C_{10}H_{20}B_{10}Se$ 330.1661; Found 330.1659.

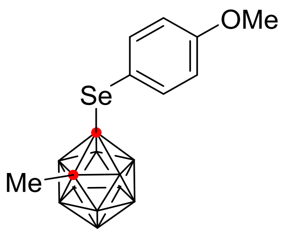

6c: Yield: 28.5 mg (83%); R_f = 0.35 (CH_2Cl_2:Hexane = 1:5); White solid; Melting point: 80-82 °C; ^{1}H NMR (400 MHz, $CDCl_3$) δ 7.56 (d, J = 8.88 Hz, 2H), 6.90 (d, J = 8.84 Hz, 2H), 3.85 (s, 3H), 2.17 (s, 3H); ^{13}C{^{1}H} NMR (100 MHz, $CDCl_3$) δ 161.9, 139.7, 118.4, 115.3, 77.6, 70.0, 55.5, 25.2; ^{11}B{^{1}H} NMR (128 MHz, $CDCl_3$) δ -3.26 (1B), -4.78 (1B), -8.66 (2B), -9.58 (6B); IR (film): 2570, 1585, 1488, 1298, 1290, 1250, 1172, 1027, 826 cm^{-1}; HRMS (EI) *m/z*: $[M]^+$ Calcd for $C_{10}H_{20}B_{10}OSe$ 346.1610; Found 346.1611.

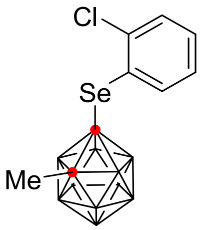

6d: Yield: 31.0 mg (89%); R_f = 0.40 (Hexane); White solid; Melting point: 109-111 °C; ^{1}H NMR (400 MHz, $CDCl_3$) δ 7.82 (d, J = 7.72 Hz, 1H), 7.56 (d, J = 8.00 Hz, 1H), 7.46 (t, J = 7.70 Hz, 1H), 7.31 (t, J = 7.54 Hz, 1H), 2.27 (s, 3H); ^{13}C{^{1}H} NMR (100 MHz, $CDCl_3$) δ 141.5, 140.6, 133.1, 130.4, 127.8, 127.7, 78.7, 69.5, 25.7; ^{11}B{^{1}H} NMR (128 MHz, $CDCl_3$) δ -2.98 (1B), -5.07 (1B), -9.15 (3B), -9.48 (3B), -10.13 (2B); IR (film): 2591, 2566, 1445, 1422, 1099, 1036, 1025, 756, 726 cm^{-1}; HRMS (EI) *m/z*: $[M]^+$ Calcd for $C_9H_{17}B_{10}ClSe$ 350.1115; Found 350.1115.

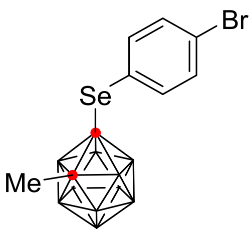

6e: Yield: 35.7 mg (91%); R_f = 0.45 (Hexane); White solid; Melting point: 148-150 °C; ^{1}H NMR (400 MHz, $CDCl_3$) δ 7.56-7.51 (m, 4H), 2.16 (s, 3H); ^{13}C{^{1}H} NMR (100 MHz, $CDCl_3$) δ 139.4, 133.1, 126.5, 126.0, 77.7, 68.8, 25.3; ^{11}B{^{1}H} NMR (128 MHz, $CDCl_3$) δ -3.00 (1B), -4.72 (1B), -8.71 (2B), -9.48 (6B); IR (film): 2592, 2571, 2551, 1465, 1380, 1007, 810, 802, 723 cm^{-1}; HRMS (EI) *m/z*: $[M]^+$ Calcd for $C_9H_{17}B_{10}BrSe$ 394.0609; Found 394.0606.

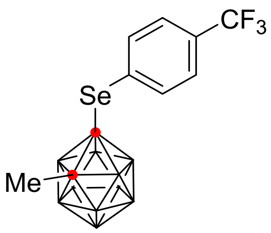

6f: Yield: 32.8 mg (86%); R_f = 0.40 (Hexane); Yellow solid; Melting point: 60-62 °C; ^{1}H NMR (400 MHz, $CDCl_3$) δ 7.81 (d, J = 7.88 Hz, 2H), 7.67 (d, J = 8.00 Hz, 2H), 2.18 (s, 3H); ^{13}C{^{1}H} NMR (100 MHz, $CDCl_3$) δ 138.3, 133.2 (q, J = 33.1 Hz), 131.1 (q, J = 1.3 Hz), 126.6 (q, J = 3.6 Hz), 123.7 (q, J = 272.7 Hz), 77.8, 68.5, 25.3; ^{11}B{^{1}H} NMR (128 MHz, $CDCl_3$) δ -2.83 (1B), -4.65 (1B), -8.71 (2B), -9.42 (6B); ^{19}F NMR (376 MHz, $CDCl_3$) δ -63.03; IR (film): 2577, 1319, 1167, 1129, 1101, 1077, 1055, 1013, 725 cm^{-1}; HRMS (EI) *m/z*: $[M]^+$ Calcd for $C_{10}H_{17}B_{10}F_3Se$ 384.1378; Found 384.1381.

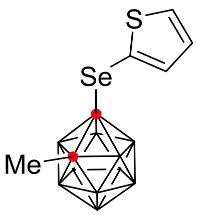

6g: Yield: 29.4 mg (92%); R_f = 0.40 (Hexane); Yellow solid; Melting point: 112-114 °C; ^{1}H NMR (400 MHz, $CDCl_3$) δ 7.60 (dd, J = 5.32 Hz, J = 1.12 Hz, 1H), 7.37 (dd, J = 3.60 Hz, J = 1.16 Hz, 1H), 7.11 (dd, J = 5.32 Hz, J = 3.64 Hz, 1H), 2.18 (s, 3H); ^{13}C{^{1}H} NMR (100 MHz, $CDCl_3$) δ 140.2, 135.0, 128.8, 121.7, 77.5, 70.3, 25.2; ^{11}B{^{1}H} NMR (128 MHz, $CDCl_3$) δ -3.12 (1B), -4.52 (1B), -8.57 (2B), -9.55 (4B), -10.18 (2B); IR (film): 2598, 2570, 2561, 1392, 1383, 1214, 849, 839, 715 cm^{-1}; HRMS (EI) *m/z*: $[M]^+$ Calcd for $C_7H_{16}B_{10}SSe$ 322.1068; Found 322.1071.

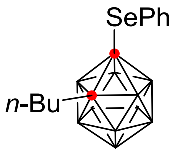

6h: Yield: 33.4 mg (94%); R_f = 0.45 (Hexane); White solid; Melting point: 99-101 °C; ^{1}H NMR (400 MHz, $CDCl_3$) δ 7.65 (d, J = 6.96 Hz, 2H), 7.50 (t, J = 7.44 Hz, 1H), 7.40 (t, J = 7.54 Hz, 2H), 2.39 (t, J = 8.44 Hz, 2H), 1.54-1.46 (m, 2H), 1.44-1.35 (m, 2H), 0.97 (t, J = 7.22 Hz, 3H); ^{13}C{^{1}H} NMR (100 MHz, $CDCl_3$) δ 137.9, 131.2, 129.8, 127.4, 82.7, 71.5, 36.8, 32.0, 22.6, 13.9; ^{11}B{^{1}H} NMR (128 MHz, $CDCl_3$) δ -3.82 (2B), -9.47 (4B), -10.78 (4B); IR (film): 2957, 2568, 1438, 1061, 1021, 737, 727, 687, 672 cm^{-1}; HRMS (EI) *m/z*: $[M]^+$ Calcd for $C_{12}H_{24}B_{10}Se$ 358.1974; Found 358.1974.

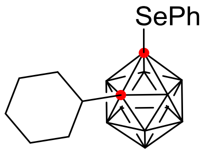

6i: Yield: 35.5 mg (93%); R_f = 0.40 (Hexane); White solid; Melting point: 118-120 °C; ^{1}H NMR (400 MHz, $CDCl_3$) δ 7.65 (d, J = 7.20 Hz, 2H), 7.50 (t, J = 7.40 Hz, 1H), 7.40 (t, J = 7.58 Hz, 2H), 2.35-2.29 (m, 1H), 1.89-1.86 (m, 4H), 1.72 (d, J = 12.80 Hz, 1H), 1.41-1.24 (m, 4H), 1.22-1.10 (m, 1H); ^{13}C{^{1}H} NMR (100 MHz, $CDCl_3$) δ 137.7, 131.1, 129.7, 127.4, 88.7, 74.7, 41.9, 34.6, 27.1, 25.5; ^{11}B{^{1}H} NMR (128 MHz, $CDCl_3$) δ -2.88 (2B), -4.03 (2B), -9.26 (4B), -11.82 (4B); IR (film): 2931, 2855, 2568, 1475, 1449, 1439, 1066, 738, 689 cm^{-1}; HRMS (EI) *m/z*: $[M]^+$ Calcd for $C_{14}H_{26}B_{10}Se$ 384.2130; Found 384.2128.

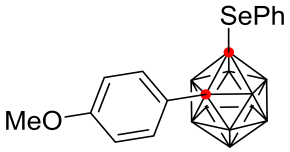

6j: Yield: 27.5 mg (77%); R_f = 0.35 ($CHCl_2$:Hexane = 1:10); White solid; Melting point: 108-110 °C; ^{1}H NMR (400 MHz, $CDCl_3$) δ 7.48 (d, J = 8.96 Hz, 2H), 7.43 (t, J = 7.40 Hz, 1H), 7.27 (t, J = 7.66 Hz, 2H), 7.19 (d, J = 7.00 Hz, 2H), 6.91 (d, J = 8.92 Hz, 2H), 3.88 (s, 3H); ^{13}C{^{1}H} NMR (100 MHz, $CDCl_3$) δ 161.6, 137.8, 133.5, 130.9, 129.4, 127.3, 124.3, 113.8, 87.1, 73.8, 55.6; ^{11}B{^{1}H} NMR (128 MHz, $CDCl_3$) δ -2.62 (2B), -

8.97 (3B), -9.94 (3B), -11.40 (2B); IR (film): 2566, 1510, 1258, 1182, 833, 737, 688, 557, 518cm^{-1}; HRMS (EI) *m/z*: $[M]^+$ Calcd for $C_{15}H_{22}B_{10}OSe$ 408.1766; Found 408.1763.

6. Procedure for C(1)-B(4)-Selenylated *o*-Carborane

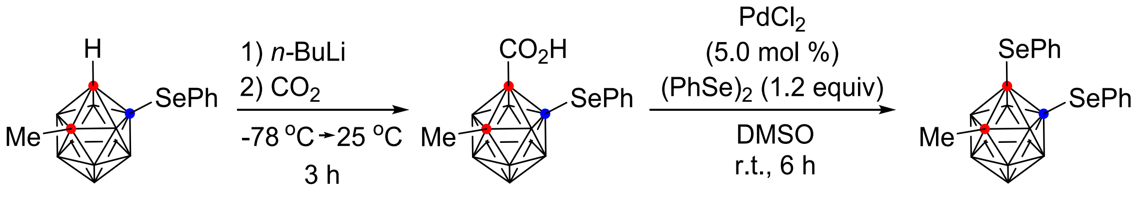

3a **8** **7**

3a (1.0 mmol, 1.0 equiv) in THF (4 mL) was cooled to -78 ^{o}C, to which was slowly added *n*-BuLi (1.1 mmol, 1.6 M in hexane, 0.7 mL). The resulting solution was stirred for 1 h at -78 ^{o}C. After the CO_2 gas was added to the solution for 30 minutes through bubbling, the mixture was warmed to room temperature and stirred for 3 h. After removal of THF and addition of water (10 mL), the solution was extracted with hexane (10 x 3 mL) to recover the residual **3a**. The aqueous layer was acidified with 3 M HCl. Then, the resultant solution was extracted with ether (10 x 3 mL). The ether solutions were combined and dried with anhydrous Mg_2SO_4. After the ether was removed through evaporation, **8** was obtained as white solid (0.77 mmol, 77%).

A test tube equipped with a magnetic stirrer was charged with **8** (0.1 mmol, 1.0 equiv), diselenide **6a** (1.2 equiv), and $PdCl_2$ (5.0 mol %) in DMSO (0.5 mL) and stirred at room temperature for 6 h under N_2. Then, sodium thiosulfate solution (5 mL) was added to quench the reaction. The mixture was extracted with dichloromethane (5 x 3 mL). The combined organic layers were washed with water and dried over anhydrous $MgSO_4$. After the volatiles were evaporated, the crude product was purified by column chromatography on silica gel to afford product **7**.

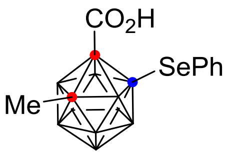

8: Yield: 275.1 mg (77%); R_f = 0.35 (MeOH:$CHCl_2$ = 1:7); White solid; Melting point: 163-165 ^{o}C; ^{1}H NMR (400 MHz, $CDCl_3$) δ 10.38 (s, 1H), 7.59-7.57 (m, 2H), 7.32 (t, J = 7.32 Hz, 1H), 7.27-7.23 (m, 2H), 2.15 (s, 3H); $^{13}C\{^1H\}$ NMR (100 MHz, $CDCl_3$) δ 162.8, 136.4, 129.2, 128.2, 127.4, 78.0, 73.6, 24.7; $^{11}B\{^1H\}$ NMR (128 MHz, $CDCl_3$) δ 0.76 (1B), -3.71 (2B), -7.81 (1B), -10.62 (6B); IR (film): 2583, 1725, 1437, 1407, 1267, 1250, 842, 735, 690 cm^{-1}; HRMS (EI) *m/z*: $[M]^+$ Calcd for $C_{10}H_{18}B_{10}O_2Se$ 360.1403; Found 360.1404.

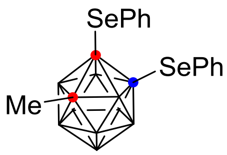

7: Yield: 44.5 mg (95%); R_f = 0.35 ($CHCl_2$:Hexane = 1:10); White solid; Melting point: 73-75 °C; ^{1}H NMR (400 MHz, $CDCl_3$) δ 7.74-7.72 (m, 2H), 7.57-7.55 (m, 2H), 7.50-7.46 (m, 1H), 7.37-7.27 (m, 5H), 2.17 (s, 3H); ^{13}C{^{1}H} NMR (100 MHz, $CDCl_3$) δ 138.3, 136.7, 131.1, 129.7, 129.0, 128.5, 127.8, 127.7, 80.3, 73.1, 25.5; ^{11}B{^{1}H} NMR (128 MHz, $CDCl_3$) δ -1.26 (3B), -3.28 (1B), -9.54 (5B), -11.95 (1B); IR (film): 2579, 1576, 1474, 1437, 1021, 943, 834, 737, 689 cm^{-1}; HRMS (EI) *m/z*: [M]$^+$ Calcd for $C_{15}H_{22}B_{10}Se_2$ 472.0982; Found 472.0985.

7. Procedure for One-Pot Synthesis

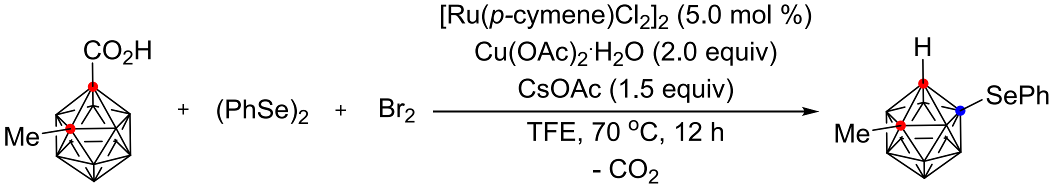

1a **5a** **3a** (89%)

A V-vial equipped with a magnetic stirrer was charged with carboxylated *o*-carborane **1a** (0.1 mmol, 1.0 equiv), diphenyl diselenide **5a** (0.75 equiv), [Ru(*p*-cymene)Cl_2]$_2$ (5.0 mol %), $Cu(OAc)_2 \cdot H_2O$ (2.0 equiv), and CsOAc (1.5 equiv) in TFE (1 mL). After slowly adding Br_2 (0.75 equiv) to the solution, the resulting solution was stirred at 70 °C for 12 h under air. The reaction mixture was filtered through a pad of silica gel and washed with EtOAc. Then, the mixture was concentrated under reduced pressure. The crude product was purified by column chromatography on silica gel to afford product **3a** (0.089 mmol, 89%).

8. A Proposed Mechanism for Pd-Catalyzed C(1)-Selenylation

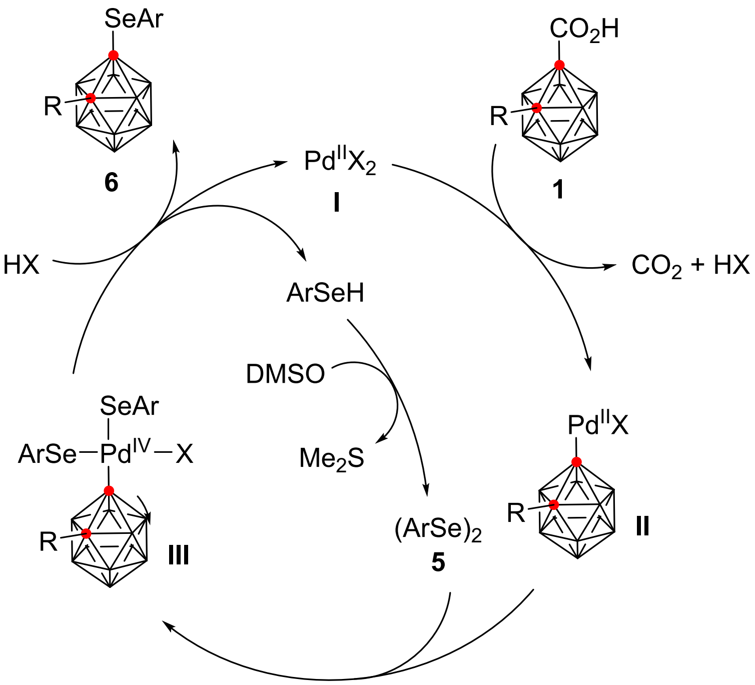

On the basis of previous works about Pd(II)-catalyzed decarboxylative coupling reactions and selenylation reactions, the plausible mechanism was proposed. First, the *ipso* attack through decarboxylation of *o*-carborane acid (**1**) forms carboranyl Pd^{II} species. Next, oxidative addition of diselenide (**5**) produces Pd^{IV} intermediate (**III**) followed by reductive elimination, affording C(1)-selenylation product (**6**). Meanwhile, the catalyst is regenerated through a ligand exchange. ArSeH produced as a by-product is converted to diselenide through proton-coupled electron transfer (PCET) by DMSO and participates in the reaction.[8,9]

9. X-Ray Crystallography

3c (20 mg) was dissolved in ether (1 mL) in 4 mL glass vial and hexane (2 mL) was slowly added to form a separate layer. The 4 mL vial was open cap. Vapor diffusion afforded crystals of the composition **3c** suitable for Xray diffraction within 2 day at room temperature. A block-like specimen of $C_{10}H_{20}B_{10}Se$, approximate dimensions 0.324 mm x 0.081 mm x 0.078 mm, was used for the X-ray crystallographic analysis. The X-ray intensity data were measured. (ellipsoid = 40%, CCDC = 1950362)

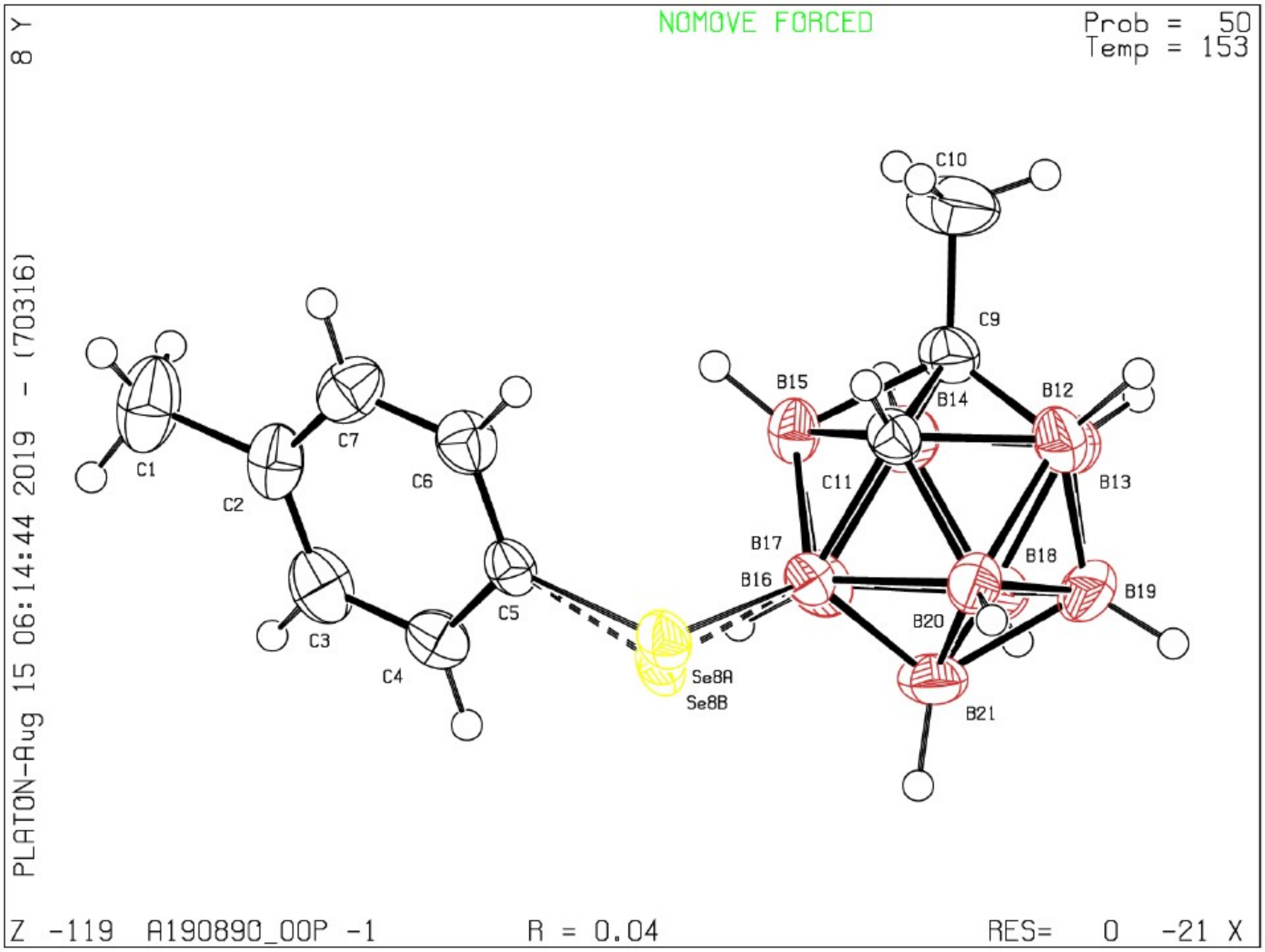

Table S1. Crystal data and structure refinement for 3c.

Empirical formula	$C_{10}H_{20}B_{10}Se$
Formula weight	327.32
Temperature	153(2) K
Wavelength	0.71073 Å
Crystal system	Triclinic
Space group	*P*-1

Unit cell dimensions	a = 7.6309(7) Å α = 102.151(3)°
	b = 9.2669(8) Å β = 105.067(3)°
	c = 12.6674(11) Å γ = 94.783(3)°
Volume	836.50(13) $Å^3$
Z	2
Density (calculated)	1.300 Mg/m^3
Absorption coefficient	2.224 mm^{-1}
F(000)	328
Crystal size	0.223 x 0.212 x 0.077 mm^3
Theta range for data collection	2.794 to 29.166°.
Index ranges	-10<=h<=10, -12<=k<=12, -17<=l<=17
Reflections collected	32855
Independent reflections	4510 [R(int) = 0.0743]
Completeness to theta = 25.242°	99.5 %
Absorption correction	Semi-empirical from equivalents
Max. and min. transmission	0.7415 and 0.6167
Refinement method	Full-matrix least-squares on F^2
Data / restraints / parameters	4510 / 0 / 232
Goodness-of-fit on F^2	1.068
Final R indices [I>2sigma(I)]	R1 = 0.0385, wR2 = 0.0905
R indices (all data)	R1 = 0.0537, wR2 = 0.1003
Largest diff. peak and hole	0.574 and -0.378 $e·Å^{-3}$

7 (20 mg) was dissolved in ether (1 mL) in 4 mL glass vial and hexane (2 mL) was slowly added to form a separate layer. The 4 mL vial was open cap. Vapor diffusion afforded crystals of the composition **7** suitable for Xray diffraction within 2 day at room temperature. A block-like specimen of $C_{10}H_{20}B_{10}Se$, approximate dimensions 0.240 mm x 0.062 mm x 0.053 mm, was used for the X-ray crystallographic analysis. The X-ray intensity data were measured. (ellipsoid = 40%, CCDC = 2055987)

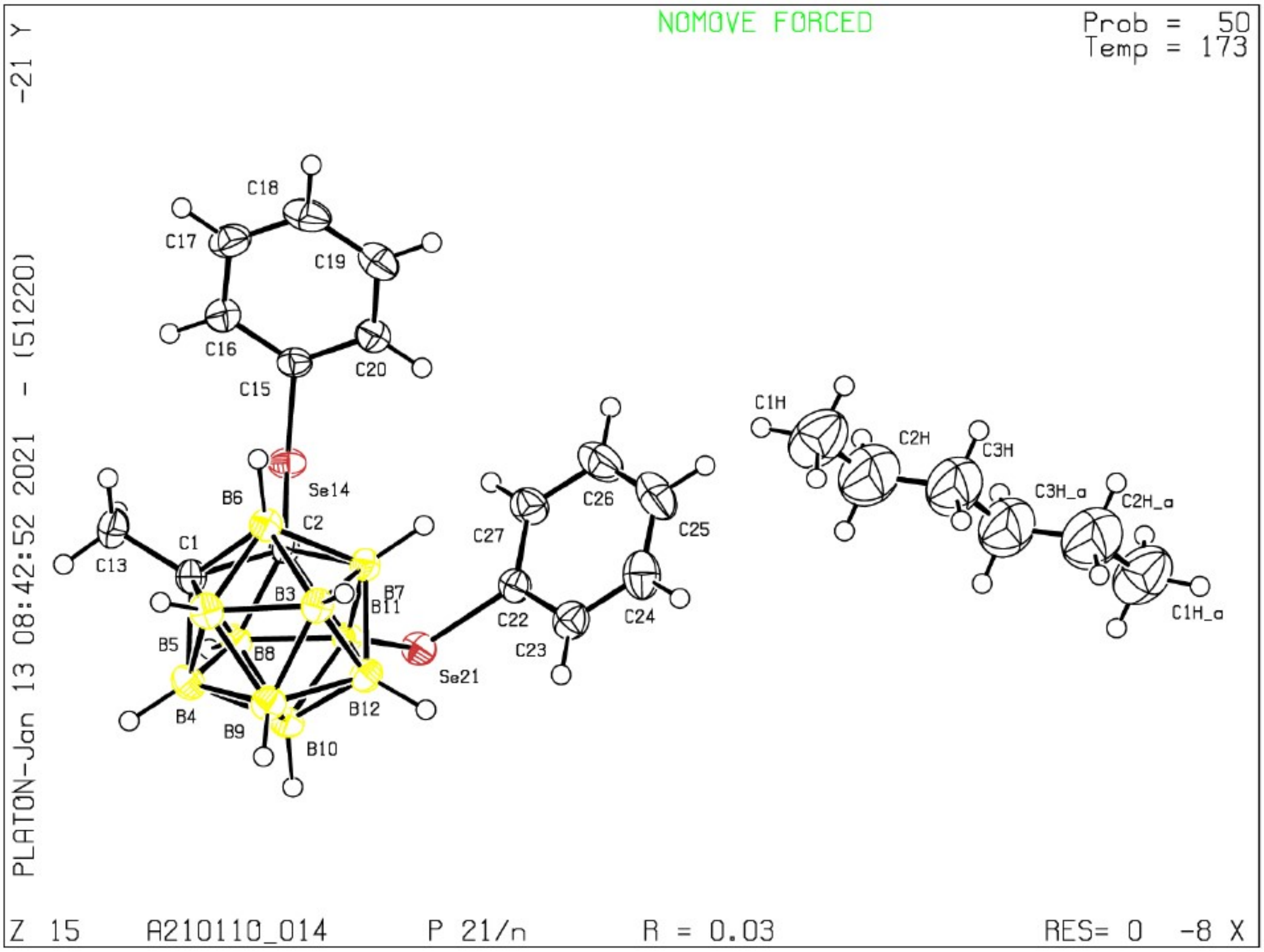

Table S2. Crystal data and structure refinement for 7.

Empirical formula	$C_{18}\ H_{29}\ B_{10}\ Se_2$
Formula weight	511.43
Temperature	173(2) K
Wavelength	0.71073 Å
Crystal system	Monoclinic
Space group	$P2_1/n$

Unit cell dimensions	a = 10.2404(7) Å	α = 90°
	b = 17.7479(12) Å	β = 95.928(2)°
	c = 13.0880(7) Å	γ = 90°
Volume	2366.0(3) $Å^3$	
Z	4	
Density (calculated)	1.436 Mg/m^3	
Absorption coefficient	3.130 mm^{-1}	
F(000)	1020	
Crystal size	0.163 x 0.095 x 0.075 mm^3	
Theta range for data collection	2.663 to 27.512°.	
Index ranges	–13<=h<=13, –23<=k<=22, –16<=l<=16	
Reflections collected	33547	
Independent reflections	5394 [R(int) = 0.0271]	
Completeness to theta = 25.242°	99.5 %	
Absorption correction	Semi-empirical from equivalents	
Max. and min. transmission	0.7456 and 0.5773	
Refinement method	Full-matrix least-squares on F^2	
Data / restraints / parameters	5394 / 0 / 273	
Goodness-of-fit on F^2	1.042	
Final R indices [I>2sigma(I)]	R1 = 0.0251, wR2 = 0.0580	
R indices (all data)	R1 = 0.0331, wR2 = 0.0624	
Largest diff. peak and hole	0.654 and –0.602 $e\cdot Å^{-3}$	

10. References

1. A. Toppino, A. R. Genady, M. E. El-Zaria, J. Reeve, F. Mostofian, J. Kent, and J. F. Valliant, *Inorg. Chem.* 2013, **52**, 8743.

2. C. Tang, and Z. Xie, *Angew. Chem., Int. Ed.* 2015, **54**, 7662.

3. Y. Quan, C. Tang, and Z. Xie, *Chem. Sci.* 2016, **7**, 5838.

4. T. Leng, G. Wu, Y.-B. Zhou, W. Gao, J. Ding, X. Huang, M. Liu, and H. Wu, *Adv. Synth. Catal.* 2018, **360**, 4336.

5. D. Singh, A. M. Deobald, L. R. S. Camargo, G. Tabarelli, O. E. D. Rodrigues, and A. L. Braga, *Org. Lett.* 2010, **12**, 3288.

6. M. Iwaoka, H. Komatsu, T. Katsuda, and S. Tomoda, *J. Am. Chem. Soc.* 2004, **126**, 5309.

7. B. Boduszek, and R. *J.* Gancarz, *Prakt. Chem.* 1996, **338**, 186.

8. A. G. Myers, D. Tanaka, and M. R. Mannion, *J. Am. Chem. Soc.* 2002, **124**, 11250.

9. M. Iwasaki, Y. Ysuchiya, K. Nakajima, and Y. Nishihra, *Org. Lett.* 2014, **16**, 4920.

11. Complete Authorship of Gaussian 16

Gaussian 16, Revision **A.03**, M. J. Frisch, G. W. Trucks, H. B. Schlegel, G. E. Scuseria, M. A. Robb, J. R. Cheeseman, G. Scalmani, V. Barone, G. A. Petersson, H. Nakatsuji, X. Li, M. Caricato, A. V. Marenich, J. Bloino, B. G. Janesko, R. Gomperts, B. Mennucci, H. P. Hratchian, J. V. Ortiz, A. F. Izmaylov, J. L. Sonnenberg, D. Williams-Young, F. Ding, F. Lipparini, F. Egidi, J. Goings, B. Peng, A. Petrone, T. Henderson, D. Ranasinghe, V. G. Zakrzewski, J. Gao, N. Rega, G. Zheng, W. Liang, M. Hada, M. Ehara, K. Toyota, R. Fukuda, J. Hasegawa, M. Ishida, T. Nakajima, Y. Honda, O. Kitao, H. Nakai, T. Vreven, K. Throssell, J. A. Montgomery, Jr., J. E. Peralta, F. Ogliaro, M. J. Bearpark, J. J. Heyd, E. N. Brothers, K. N. Kudin, V. N. Staroverov, T. A. Keith, R. Kobayashi, J. Normand, K. Raghavachari, A. P. Rendell, J. C. Burant, S. S. Iyengar, J. Tomasi, M. Cossi, J. M. Millam, M. Klene, C. Adamo, R. Cammi, J. W. Ochterski, R. L. Martin, K. Morokuma, O. Farkas, J. B. Foresman, and D. J. Fox, Gaussian, Inc., Wallingford CT, 2016.

12. Complete Authorship of CYLview

Legault, C. Y. *CYLview*, 1.0.565 BETA; University of Sherbrooke, Québec, Montreal, Canada, 2012. https://www.cylview.org/

13. Discussion of DFT Methods and Results

As mentioned in the main body of this work, all geometry optimizations were performed at the PBE-D3BJ/6-31G*+LANL2DZ level of theory, with single point solvation energy refinements then performed on the optimized structures at the PBE-D3BJ/def2-QZVP level of theory with the SMD implicit solvation model in 2,2,2-trifluoroethanol (TFE). The Gibbs thermal correction factors from the structures optimized at PBE-D3BJ/6-31G*+LANL2DZ was then added to the single point energy from the PBE-D3BJ/def2-QZVP structures to obtain final corrected energy values. All ground and transition states were verified through frequency calculations – i.e.: zero negative frequencies in ground states and one negative frequency in transition states. All energy values are in kcal/mol, all distances are in Ångstroms.

Ligand exchange is spontaneous, and dissociation of p-cymene is favored by > 22 kcal/mol:

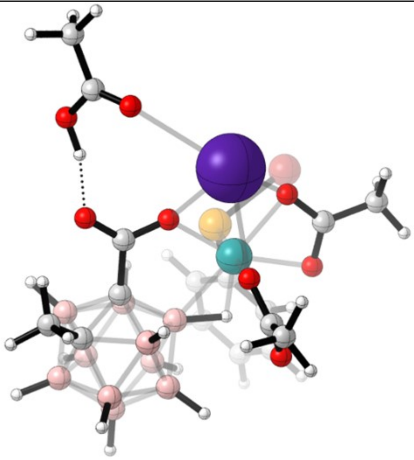<br>ΔG = 0.0 (**I**)<br>010-Cs-Cluster-RuII-1HOAc-2OAc-PhSeBr-01-PBE	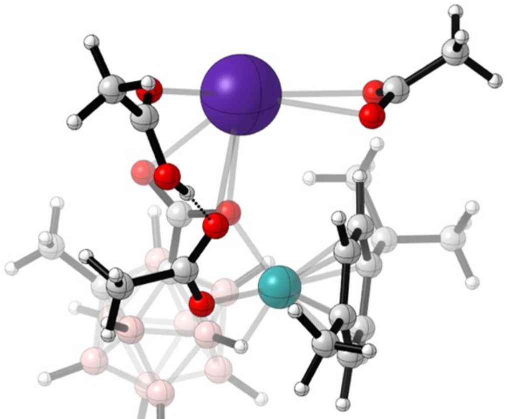<br>ΔG = 22.3 (**I’**)<br>010-Cs-Cluster-RuII-1HOAc-2OAc-Cymene-06-PBE

Deprotonation of **I**, **TS II**, is favored over deprotonation of **I’**, **TS II’**, by 21.3 kcal/mol:

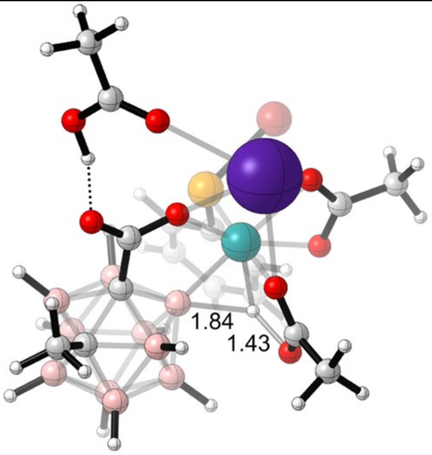<br>ΔG‡ = 10.2 (**TS II**)<br>015-Cs-Cluster-RuII-1HOAc-2OAc-PhSeBr-01-Deprotonation-TS-01-PBE	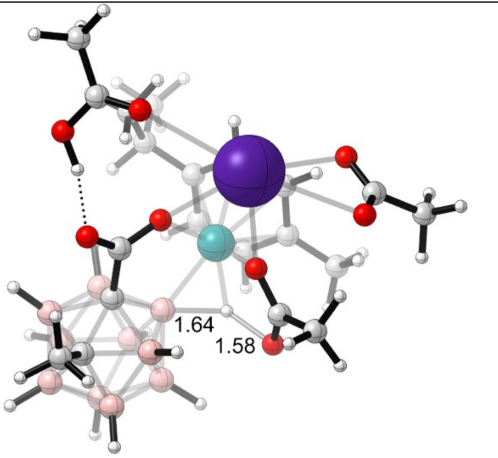<br>ΔG‡ = 31.5 (**TS II’**)<br>015-Cs-Cluster-RuII-1HOAc-2OAc-Cymene-Deprotonation-TS-01-PBE

TS II is preferred by 1.7 kcal/mol over **TS II''**, deprotonation at B(3). **TS II** is also preferred by 43.7 kcal/mol over decarboxylation **TS II'''**.

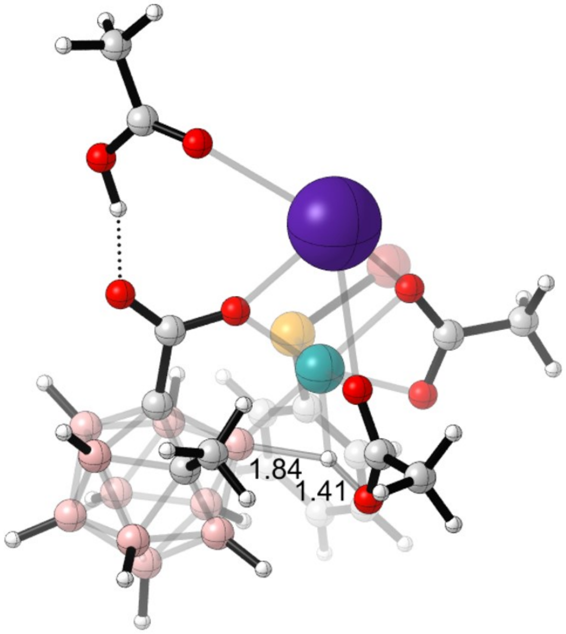

ΔG‡ = 11.9 (**TS II''**)
015-Cs-Cluster-RuII-1HOAc-2OAc-PhSeBr-01-Deprotonation-TS-02-PBE

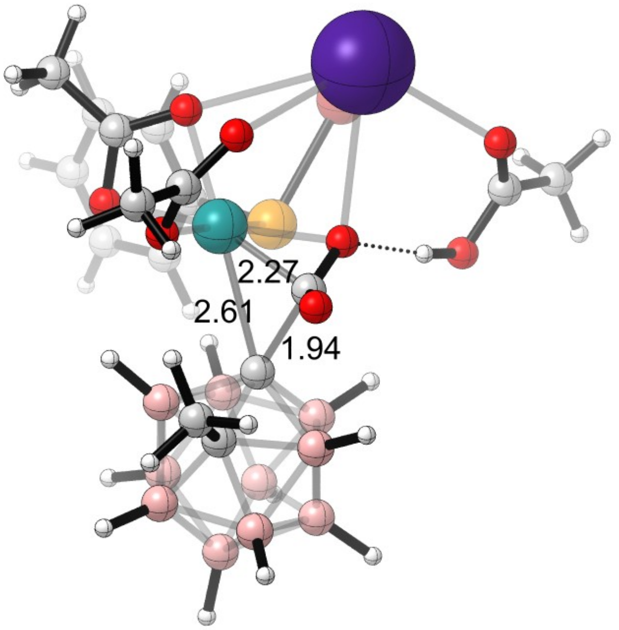

ΔG‡ = 53.9 (**TS II'''**)
017-Cs-Cluster-RuII-1HOAc-2OAc-PhSeBr-Decarboxylation-TS-01-PBE

Ligand rearrangement from **III'** to **III** following deprotonation **TS II** is spontaneous:

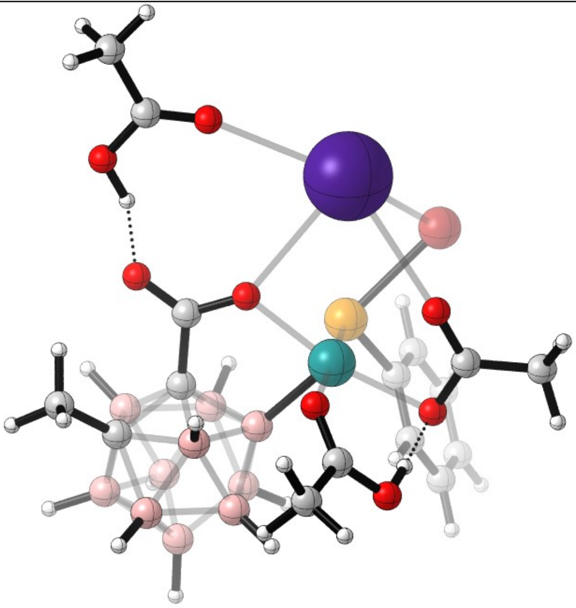

ΔG = 4.7 (**III'**)
020-Cs-Deprotonated-Cluster-RuII-2HOAc-1OAc-PhSeBr-01-PBE

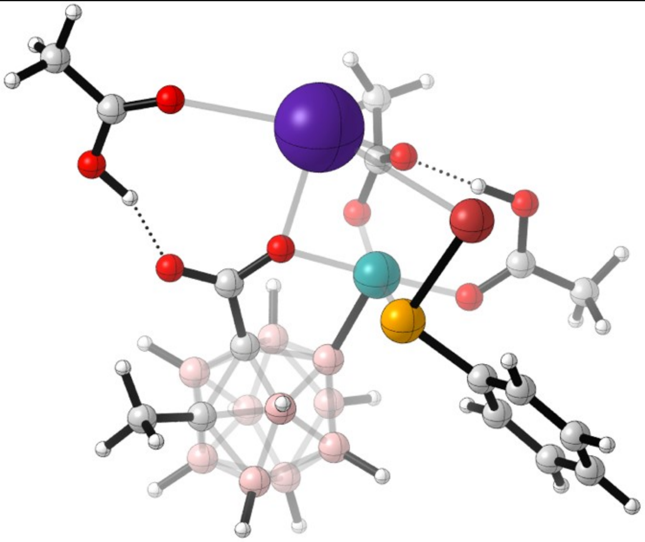

ΔG = 1.4 (**III**)
020-Cs-Deprotonated-Cluster-RuII-2HOAc-1OAc-PhSeBr-04-PBE

Many conformations of the intermediate following selenylation of the *o*-carborane were computed, with ligand rearrangement from **V’** to **V** again found to be spontaneous:

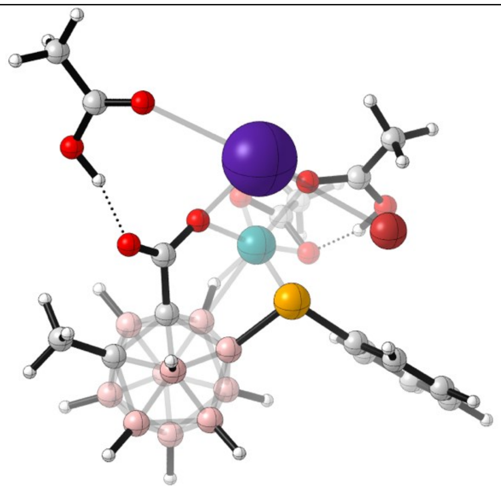<br>ΔG = 0.5 (**V’**)<br>030-Selenylated-Cluster-RuII-2HOAc-1OAc-CsBr-22-PBE	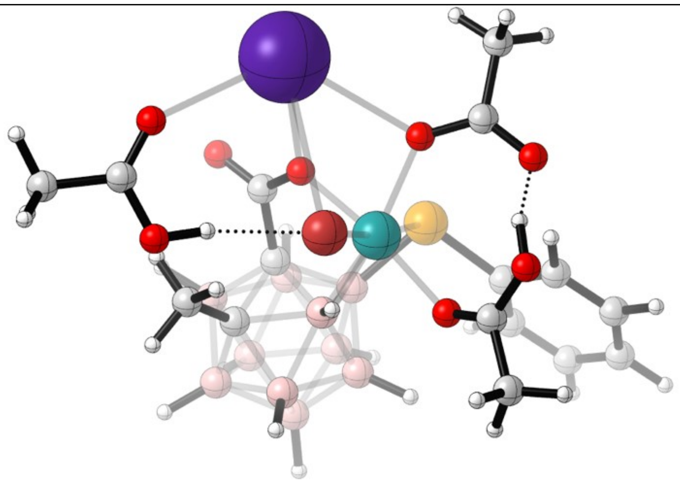<br>ΔG = -15.0 (**V**)<br>030-Selenylated-Cluster-RuII-2HOAc-1OAc-CsBr-06-PBE

As with the previous intermediate, many conformations of decarboxylation **TS VI** were computed. The conformation of these TSs with the lowest energy is 30.6 kcal/mol above **V**. A decarboxylation TS with *p*-cymene ligated to Ru, **TS VI’**, was also located, which lies 57.2 kcal/mol higher in energy than **V**, making it energetically unreasonable.

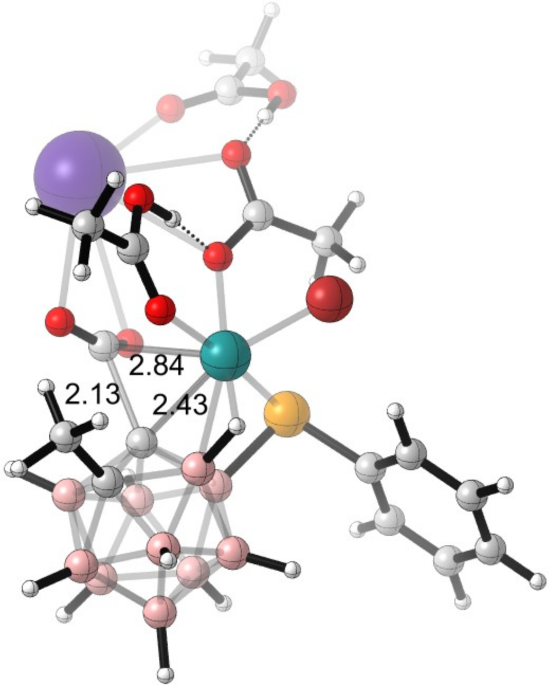<br><br>ΔG‡ = 15.6 (**TS VI**)<br>035-Selenylated-Cluster-RuII-2HOAc-1OAc-CsBr-Decarboxylation-TS-14-PBE	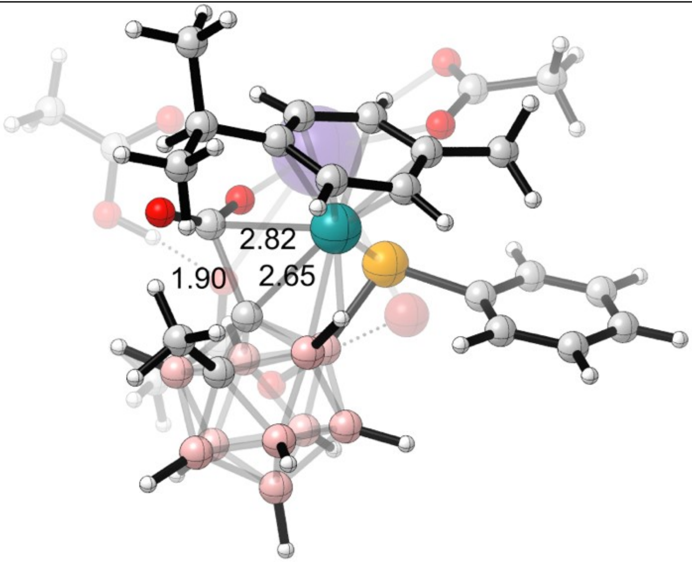<br><br>ΔG‡ = 42.2 (**TS VI’**)<br>035-Selenylated-Cluster-RuII-1OAc-2HOAc-Cymene-CsBr-Decarboxylation-TS-01

As mentioned in the main manuscript, several interactions in the decarboxylation transition state are necessary in order to make the process energetically reasonable. The absence of a Ru—Br interaction raises the barrier to decarboxylation to at least 37.8 kcal/mol; the absence of an agostic interaction between Ru and a B—H bond raises the barrier to decarboxylation to at least 43.3 kcal/mol. The absence of an agostic interaction and a Ru—Se interaction raises the barrier to decarboxylation to at least 65.0 kcal/mol; and the absence of a Ru—Br interaction, agostic interaction between Ru and a B—H bond, and Ru—Se interaction raises the barrier to decarboxylation to at least 74.9 kcal/mol.

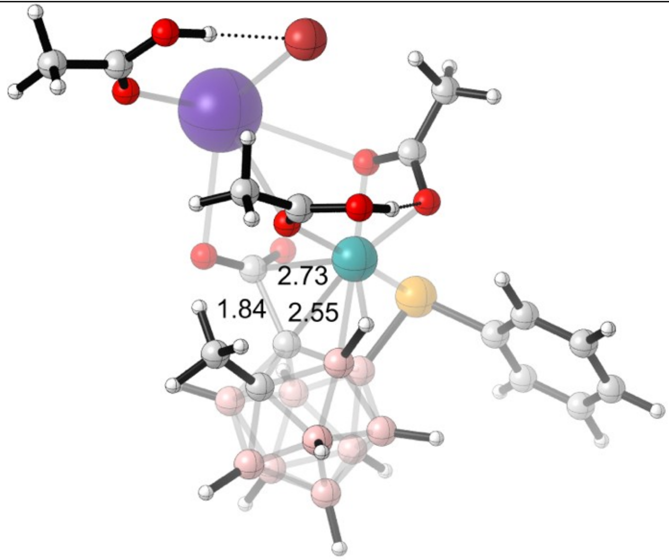<br>$\Delta G^{\ddagger}$ = 22.8<br>035-Selenylated-Cluster-RuII-2HOAc-1OAc-CsBr-Decarboxylation-TS-19-PBE<br>(No Ru—Br interaction)	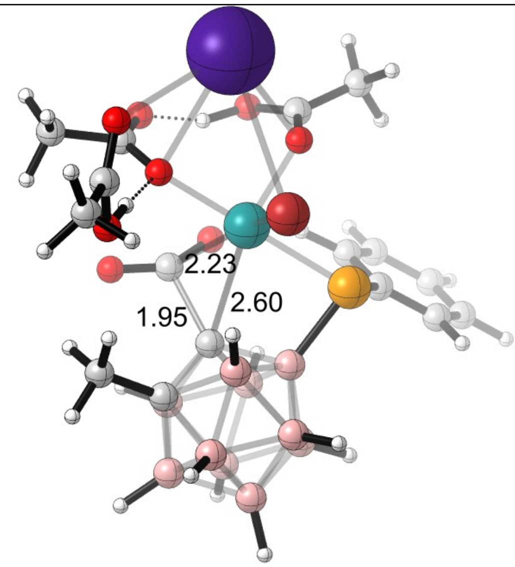<br>$\Delta G^{\ddagger}$ = 28.3<br>035-Selenylated-Cluster-RuII-2HOAc-1OAc-CsBr-Decarboxylation-TS-24-PBE<br>(No Ru / B—H agostic interaction)
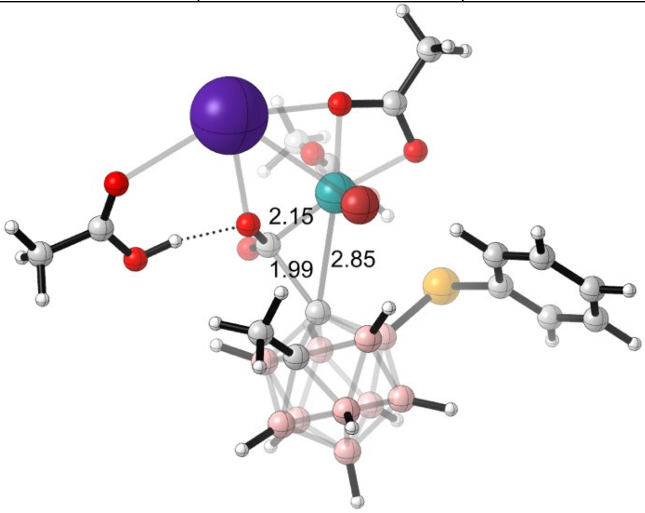<br>$\Delta G^{\ddagger}$ = 50.0<br>035-Selenylated-Cluster-RuII-2HOAc-1OAc-CsBr-Decarboxylation-TS-34-PBE<br>(No Ru / B—H agostic interaction; no Ru—Se interaction)	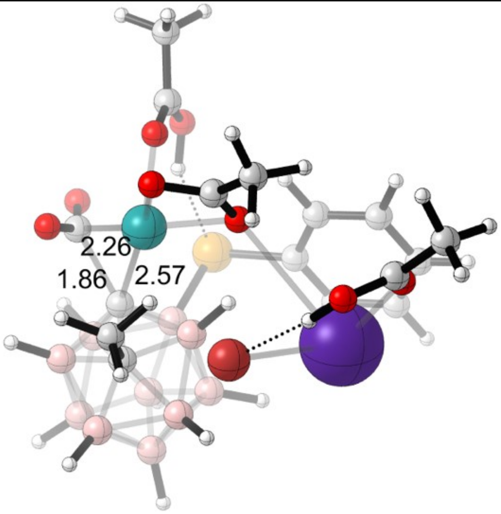<br>$\Delta G^{\ddagger}$ = 59.9<br>035-Selenylated-Cluster-RuII-2HOAc-1OAc-CsBr-Decarboxylation-TS-04-PBE<br>(No Ru / B—H agostic interaction; no Ru—Se interaction; no Ru—Br interaction)

We also examined whether an equivalent of CsBr is necessary in the coordination sphere for decarboxylation to occur. Two decarboxylation TSs were located with CsBr absent. They lie 41.7 kcal/mol and 43.5 kcal/mol, **TS VI''** and **TS VI'''** respectively, above **V**, making them energetically unreasonable.

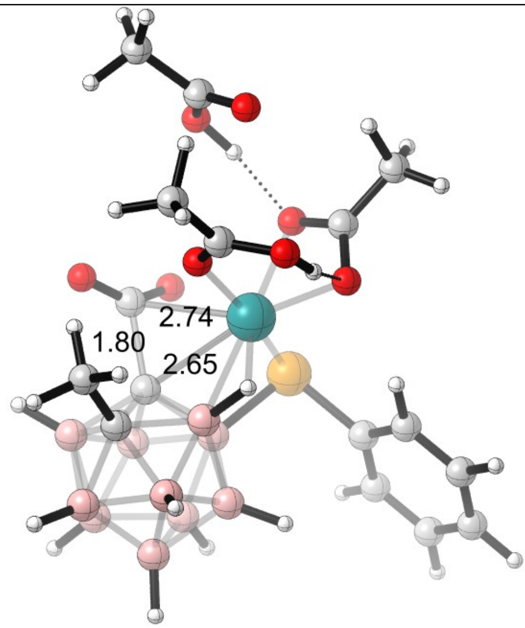	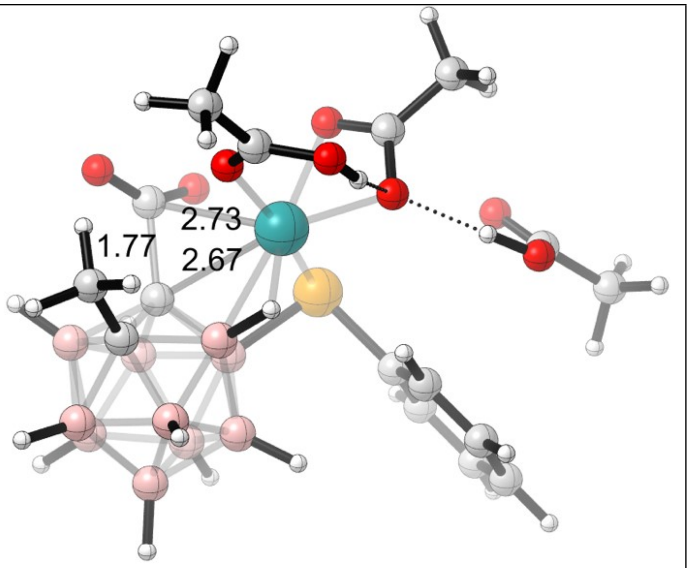
ΔG‡ = 26.7 (**TS VI''**)<br>035-Selenylated-Cluster-RuII-2HOAc-1OAc-Decarboxylation-TS-01-SP-TFE-PBE	ΔG‡ = 28.5 (**TS VI'''**)<br>035-Selenylated-Cluster-RuII-2HOAc-1OAc-Decarboxylation-TS-02-SP-TFE-PBE

Following decarboxylation, we examined whether CO_2 would remain in the coordination sphere of Ru (**VII'**), or whether dissociation – i.e.: bubbling out of solution – was more favorable (**VII**). Dissociation is favored by 2.0 kcal/mol. Decarboxylation is also endothermic by 9.2 kcal/mol.

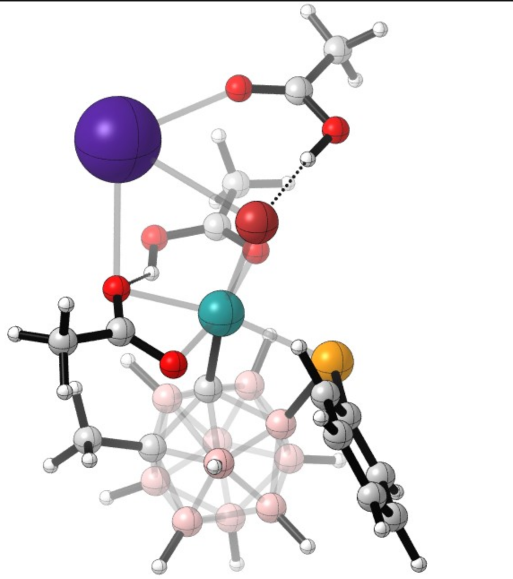	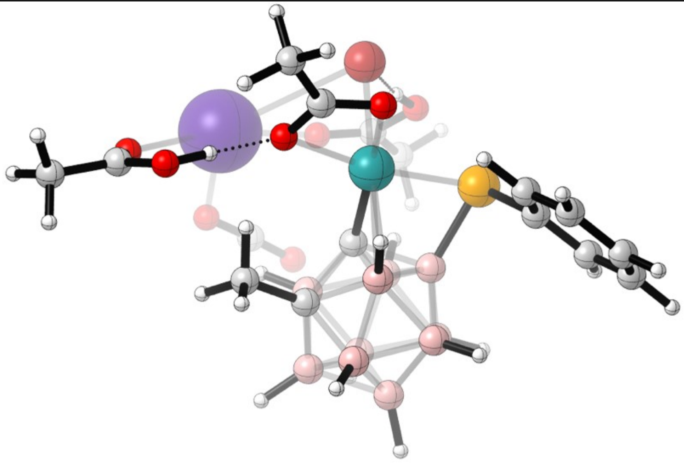
ΔG = -5.8 (**VII**)<br>040-Decarboxylated-Cluster-RuII-2HOAc-1OAc-CsBr-04-PBE	ΔG = -3.8 (**VII'**)<br>040-Decarboxylated-Cluster-RuII-2HOAc-1OAc-CsBr-CO2-01-PBE

As discussed in the main manuscript, we found two pathways for the decarboxylated cluster to undergo protonolysis and arrive at the product-catalyst complex. The stepwise process through a Ru(IV)-hydride species is shown in the main manuscript; below is **TS VIII'**, which leads directly from the decarboxylated cluster to the product-catalyst complex **XI**. The pathway shown in the main manuscript is more favorable by 13.5 kcal/mol. The difference between **TS VIII'** and **TS X** is also represented graphically in the reaction coordinate below.

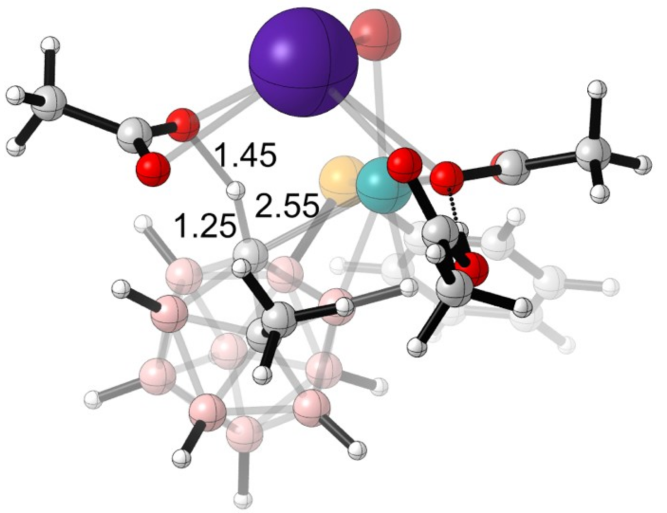	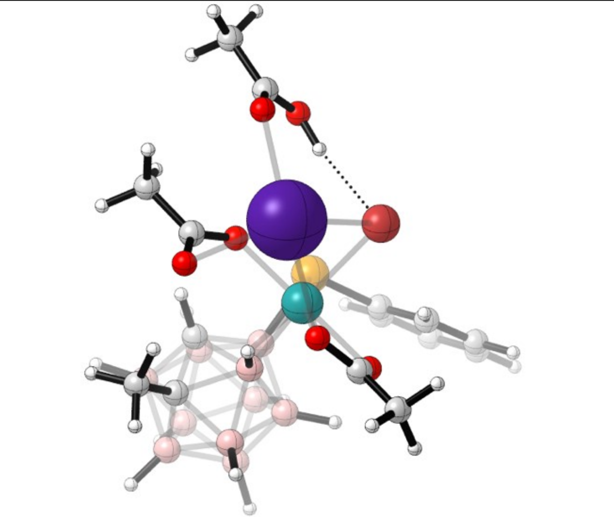
ΔG‡ = 14.3 (**TS VIII'**)<br>047-Decarboxylated-Cluster-RuII-2HOAc-1OAc-CsBr-01-Protonolysis-TS-01-PBE	ΔG‡ = -15.6 (**XI**)<br>050-Product-Catalyst-Complex-1HOAc-2OAc-CsBr-01-PBE

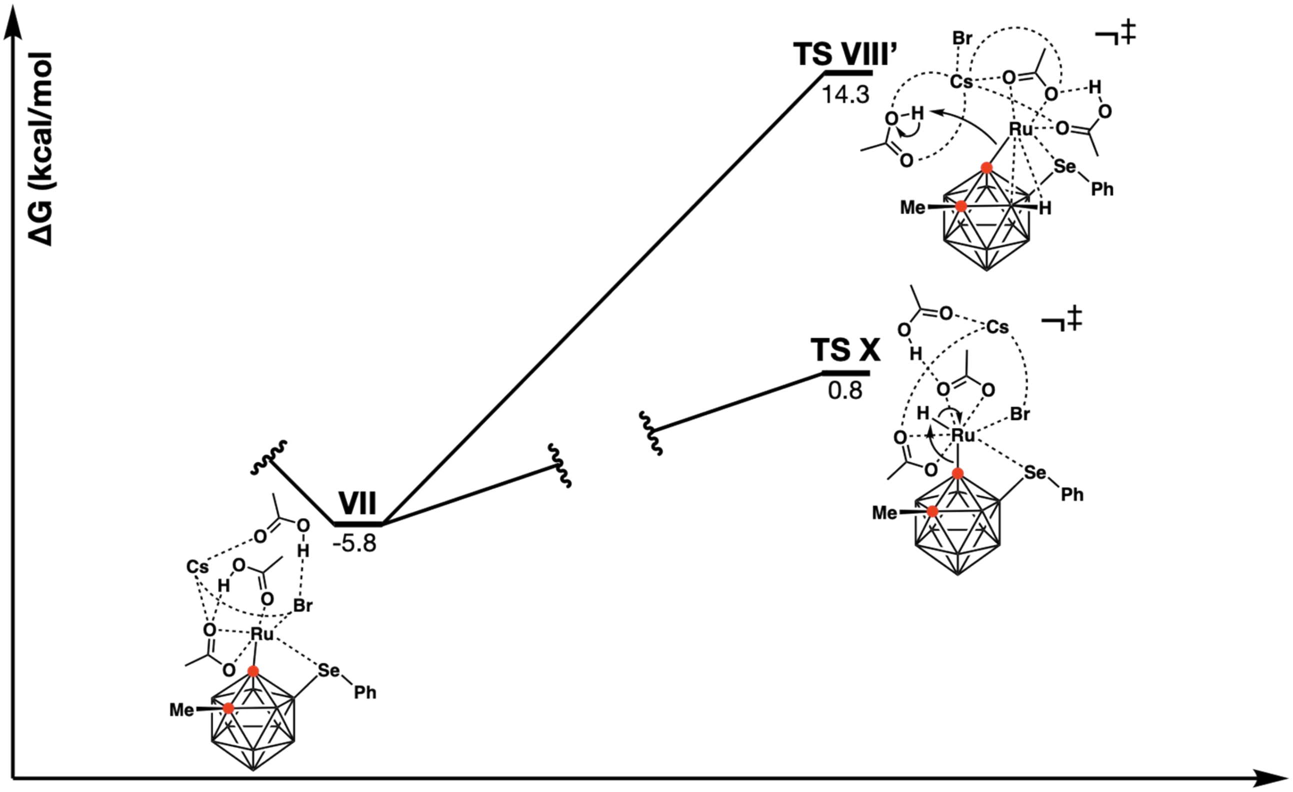

14. Ground State Computed Geometries and Energies

Supporting Information: 000-2-OAc-Active-RuII-Catalyst-02-PBE.log

----

Using Gaussian 16: ES64L-G16RevA.03 25-Dec-2016

==

# pbepbe/gen/auto pseudo=read gfprint gfinput empiricaldispersion=gd3bj
scf=(direct,vshift=200,tight,maxcycle=300,yqc) opt=(gdiis,maxcycle=250)
freq=noraman
#N Geom=AllCheck Guess=TCheck SCRF=Check GenChk RPBEPBE/Gen/Auto Freq

----

Pointgroup= C1 Stoichiometry= C14H20O4Ru C1[X(C14H20O4Ru)] #Atoms= 39
Charge = 0 Multiplicity = 1

SCF Energy= -939.436325741 Predicted Change= -1.959306D-08

==

Optimization completed on the basis of negligible forces. {Found 2 times}

Item	Max Val.		Criteria	Pass?	RMS Val.		Criteria	Pass?
Force	0.00000	||	0.00045	[YES]	0.00000	||	0.00030	[YES]
Displ	0.00666	||	0.00180	[NO]	0.00666	||	0.00180	[YES]

Atomic Type	Coordinates (Angstroms) X	Y	Z
Ru	-0.081126	-0.395828	-0.065082
C	1.140184	-1.155615	-1.742901
C	0.683814	-2.253743	-0.928072
C	1.819118	-0.050232	-1.143687
H	0.851057	-1.108307	-2.795756
C	1.011144	-2.260597	0.470602
C	2.077676	0.012823	0.260271
H	1.990854	0.840060	-1.753981
C	1.632384	-1.113859	1.050348
H	0.615683	-3.050208	1.115183
H	1.701656	-1.046150	2.141467
C	-0.169204	-3.344010	-1.516623
H	0.449646	-4.228184	-1.756230
H	-0.954025	-3.648582	-0.805647
H	-0.660705	-3.003936	-2.441729
C	2.714627	1.213367	0.936255
H	2.275410	1.266208	1.951528
C	2.408799	2.540546	0.224407
H	1.329604	2.636372	0.015957
H	2.731674	3.387011	0.853673
H	2.955142	2.620531	-0.733076
C	4.233650	0.984804	1.085205

H	4.697441	1.824712	1.631084
H	4.453535	0.053515	1.635413
H	4.714657	0.915421	0.093206
O	-2.106247	-1.122445	0.014593
C	-2.282246	-0.541547	1.150236
O	-1.251365	0.015013	1.686864
C	-3.620494	-0.518989	1.821374
H	-3.504736	-0.465672	2.914655
H	-4.140291	0.386929	1.468288
H	-4.209406	-1.404360	1.536781
O	-0.677081	1.415451	-0.863774
C	-1.822913	2.068016	-0.730178
O	-2.837024	1.709283	-0.126468
C	-1.755014	3.423110	-1.444906
H	-1.028611	4.079072	-0.933556
H	-1.408626	3.298998	-2.484396
H	-2.745811	3.900129	-1.432961

Statistical Thermodynamic Analysis
Temperature= 298.150 Kelvin Pressure= 1.00000 Atm
==
SCF Energy= -939.436325741 Predicted Change= -1.959306D-08
Zero-point correction (ZPE)= -939.1262 0.31009
Internal Energy (U)= -939.1036 0.33265
Enthalpy (H)= -939.1027 0.33360
Gibbs Free Energy (G)= -939.1787 0.25753
Entropy (S)= 0.00025512

Frequencies -- 21.4401 36.4655 47.7272
Supporting Information: 000-AcO-Anion-PBE.log

Using Gaussian 16: ES64L-G16RevA.03 25-Dec-2016
==
# pbepbe/gen/auto pseudo=read gfprint gfinput empiricaldispersion=gd3bj
scf=(direct,vshift=200,tight,maxcycle=300,yqc) opt=(gdiis,maxcycle=250)
freq=noraman
#N Geom=AllCheck Guess=TCheck SCRF=Check GenChk RPBEPBE/Gen/Auto Freq
----
Pointgroup= C1 Stoichiometry= C2H3O2(1-) C1[X(C2H3O2)] #Atoms= 7
Charge = -1 Multiplicity = 1

SCF Energy= -228.241497255 Predicted Change= -1.048928D-08
==
Optimization completed. {Found 2 times}
Item Max Val. Criteria Pass? RMS Val. Criteria Pass?
Force 0.00005 || 0.00045 [YES] 0.00001 || 0.00030 [YES]

Displ 0.00020 || 0.00180 [YES] 0.00020 || 0.00180 [YES]

Atomic Coordinates (Angstroms)

Type	X	Y	Z
C	-0.222666	0.002262	0.000013
O	-0.818150	-1.115389	-0.000023
O	-0.693773	1.179056	-0.000002
C	1.360196	-0.060573	0.000010
H	1.763454	0.469779	-0.885918
H	1.763432	0.469468	0.886135
H	1.743318	-1.098710	-0.000156

Statistical Thermodynamic Analysis
Temperature= 298.150 Kelvin Pressure= 1.00000 Atm

===

SCF Energy= -228.241497255 Predicted Change= -1.048928D-08
Zero-point correction (ZPE)= -228.1946 0.04684
Internal Energy (U)= -228.1901 0.05137
Enthalpy (H)= -228.1891 0.05232
Gibbs Free Energy (G)= -228.2223 0.01914
Entropy (S)= 0.00011128

Frequencies -- 35.9432 399.1219 582.5225
Supporting Information: 000-AcOH-PBE.log

Using Gaussian 16: ES64L-G16RevA.03 25-Dec-2016

===

# pbepbe/gen/auto pseudo=read gfprint gfinput empiricaldispersion=gd3bj
scf=(direct,vshift=200,tight,maxcycle=300,yqc) opt=(gdiis,maxcycle=250)
freq=noraman
#N Geom=AllCheck Guess=TCheck SCRF=Check GenChk RPBEPBE/Gen/Auto Freq

---

Pointgroup= C1 Stoichiometry= C2H4O2 C1[X(C2H4O2)] #Atoms= 8
Charge = 0 Multiplicity = 1

SCF Energy= -228.825029296 Predicted Change= -3.590149D-07

===

Optimization completed. {Found 1 times}

Item	Max Val.	Criteria	Pass?	RMS Val.	Criteria	Pass?
Force	0.00019 ||	0.00045	[YES]	0.00007 ||	0.00030	[YES]
Displ	0.00187 ||	0.00180	[NO]	0.00187 ||	0.00180	[YES]

Atomic Coordinates (Angstroms)
Type X Y Z

C	-0.092605	0.125863	0.000543
O	-0.662480	1.205397	-0.000172
O	-0.768875	-1.063921	-0.000129
H	-1.720213	-0.805566	-0.000451
C	1.401929	-0.098087	-0.000007
H	1.699020	-0.681786	0.887515
H	1.698400	-0.682274	-0.887419
H	1.917693	0.871163	-0.000456

Statistical Thermodynamic Analysis
Temperature= 298.150 Kelvin   Pressure= 1.00000 Atm

===

SCF Energy= -228.825029296 Predicted Change= -3.590149D-07
Zero-point correction (ZPE)= -228.7646 0.06037
Internal Energy (U)= -228.7600 0.06501
Enthalpy (H)= -228.7590 0.06595
Gibbs Free Energy (G)= -228.7920 0.03302
Entropy (S)= 0.00011046

Frequencies -- 62.1976 407.9943 530.9545
Supporting Information: 000-Borane-Cluster-SePh-Added-PBE.log

----

Using Gaussian 16: ES64L-G16RevA.03 25-Dec-2016

===

# pbepbe/gen/auto pseudo=read gfprint gfinput empiricaldispersion=gd3bj
scf=(direct,vshift=200,tight,maxcycle=300,yqc) opt=(gdiis,maxcycle=250)
freq=noraman
#N Geom=AllCheck Guess=TCheck SCRF=Check GenChk RPBEPBE/Gen/Auto Freq

----

Pointgroup= C1 Stoichiometry= C9H18B10Se C1[X(C9H18B10Se)] #Atoms= 38
Charge = 0 Multiplicity = 1

SCF Energy= -3000.23200617 Predicted Change= -4.438154D-09

===

Optimization completed on the basis of negligible forces. {Found 2 times}

Item	Max Val.	Criteria	Pass?	RMS Val.	Criteria	Pass?
Force	0.00000 ||	0.00045	[YES]	0.00000 ||	0.00030	[YES]
Displ	0.00551 ||	0.00180	[NO]	0.00551 ||	0.00180	[YES]

Atomic Type	Coordinates (Angstroms) X	Y	Z
B	0.863297	1.014871	-1.403455
B	2.626625	1.146173	-1.637360
H	3.121079	1.610787	-2.623576
H	0.065996	1.384948	-2.215551

B	3.388947	-0.222551	-0.795312
B	3.375129	1.392546	-0.042879
B	0.535234	-0.435995	-0.423671
B	0.530706	1.176279	0.340027
H	-0.457222	1.568953	0.882385
H	4.405343	1.940571	0.215596
H	4.431630	-0.757049	-1.032145
C	1.369202	-0.116091	1.053890
C	2.994824	-0.005176	0.857567
B	1.842222	-0.461945	-1.639100
B	2.105639	-1.190024	-0.049718
H	2.229693	-2.327693	0.288427
H	1.761012	-1.162256	-2.605464
B	1.808194	2.163399	-0.414222
H	1.706821	3.352190	-0.506033
B	2.082569	1.430713	1.169300
H	2.191512	1.909649	2.256647
H	1.024615	-0.568797	1.986378
Se	-1.038654	-1.648791	-0.404017
C	-2.418746	-0.350983	-0.013576
C	-3.044965	-0.363081	1.243276
C	-2.823654	0.573124	-0.991467
C	-4.066707	0.555874	1.524837
H	-2.725475	-1.087189	1.999024
C	-3.835052	1.498558	-0.699625
H	-2.339352	0.569105	-1.971788
C	-4.459297	1.490937	0.556681
H	-4.550960	0.543209	2.506921
H	-4.139828	2.223634	-1.461368
H	-5.252628	2.211709	0.779659
C	3.894561	-0.524851	1.968708
H	3.556840	-1.512758	2.319498
H	4.918258	-0.631155	1.579208
H	3.913107	0.173851	2.818939

Statistical Thermodynamic Analysis
Temperature= 298.150 Kelvin Pressure= 1.00000 Atm

==

SCF Energy= -3000.23200617 Predicted Change= -4.438154D-09
Zero-point correction (ZPE)= -2999.9510 0.28095
Internal Energy (U)= -2999.9340 0.29797
Enthalpy (H)= -2999.9330 0.29892
Gibbs Free Energy (G)= -2999.9966 0.23534
Entropy (S)= 0.00021324

Frequencies -- 20.4197 28.8974 34.5974

Supporting Information: 000-CO2-PBE.log

Using Gaussian 16: ES64L-G16RevA.03 25-Dec-2016

==

# pbepbe/gen/auto pseudo=read gfprint gfinput empiricaldispersion=gd3bj
scf=(direct,vshift=200,tight,maxcycle=300,yqc) opt=(gdiis,maxcycle=250)
freq=noraman
#N Geom=AllCheck Guess=TCheck SCRF=Check GenChk RPBEPBE/Gen/Auto Freq

---

Pointgroup= D*H Stoichiometry= CO2 D*H[O(C),C*(O.O)] #Atoms= 3
Charge = 0 Multiplicity = 1

SCF Energy= -188.391714252 Predicted Change= -2.915502D-08

==

Optimization completed. {Found 2 times}

Item	Max Val.	Criteria	Pass?	RMS Val.	Criteria	Pass?
Force	0.00017 ||	0.00045	[YES]	0.00012 ||	0.00030	[YES]
Displ	0.00016 ||	0.00180	[YES]	0.00016 ||	0.00180	[YES]

Atomic Type	Coordinates (Angstroms) X	Y	Z
C	0.000000	0.000000	0.000000
O	0.000000	0.000000	1.180809
O	0.000000	0.000000	-1.180809

Statistical Thermodynamic Analysis
Temperature= 298.150 Kelvin Pressure= 1.00000 Atm

==

SCF Energy= -188.391714252 Predicted Change= -2.915502D-08
Zero-point correction (ZPE)= -188.3804 0.01126
Internal Energy (U)= -188.3777 0.01393
Enthalpy (H)= -188.3768 0.01488
Gibbs Free Energy (G)= -188.4011 -0.00948
Entropy (S)= 8.171e-05

Frequencies -- 618.6575 618.6575 1321.4468

Supporting Information: 000-Cs-Borane-Cluster-PBE.log

Using Gaussian 16: ES64L-G16RevA.03 25-Dec-2016

==

# pbepbe/gen/auto pseudo=read gfprint gfinput empiricaldispersion=gd3bj
scf=(direct,vshift=200,tight,maxcycle=300,yqc) opt=(gdiis,maxcycle=250)
freq=noraman
#N Geom=AllCheck Guess=TCheck SCRF=Check GenChk RPBEPBE/Gen/Auto Freq

---

Pointgroup= C1   Stoichiometry= C4H13B10CsO2   C1[X(C4H13B10CsO2)] #Atoms= 30
Charge = 0   Multiplicity = 1

SCF Energy= -578.622601335   Predicted Change= -2.302869D-10

===

Optimization completed.   {Found 2 times}

Item	Max Val.	Criteria	Pass?	RMS Val.	Criteria	Pass?
Force	0.00000 ||	0.00045	[YES]	0.00000 ||	0.00030	[YES]
Displ	0.00016 ||	0.00180	[YES]	0.00016 ||	0.00180	[YES]

Atomic Type	Coordinates (Angstroms) X	Y	Z
B	3.328452	-1.859205	-0.280660
B	4.427015	-0.457392	-0.267171
H	5.614347	-0.551066	-0.401867
H	3.707731	-2.986839	-0.428144
B	3.726109	0.768097	0.816745
B	3.549429	0.927739	-0.942422
B	1.965146	-1.499709	0.799761
B	1.768628	-1.326033	-0.956484
H	1.288234	-2.266116	1.411477
H	0.953182	-1.975543	-1.544390
H	3.988346	1.889052	-1.504430
H	4.286786	1.618126	1.445978
C	1.195201	-0.130207	0.114029
C	2.233682	1.198381	0.116576
B	3.597316	-0.953866	1.235782
B	2.203001	0.120389	1.470443
H	1.697948	0.527393	2.473038
H	4.170785	-1.406253	2.185795
B	3.286643	-0.685740	-1.628915
H	3.630743	-0.948947	-2.746614
B	1.910871	0.399656	-1.365026
H	1.210294	0.958488	-2.150330
C	-0.346916	0.066545	0.273194
O	-0.923774	-0.843049	0.926287
O	-0.858391	1.076027	-0.299753
Cs	-3.602291	-0.091757	-0.074799
C	1.659376	2.592368	0.311480
H	0.593558	2.585923	0.035463
H	1.774178	2.910661	1.358998
H	2.206088	3.295751	-0.335248

Statistical Thermodynamic Analysis
Temperature= 298.150 Kelvin   Pressure= 1.00000 Atm

==
SCF Energy=   -578.622601335   Predicted Change= -2.302869D-10
Zero-point correction (ZPE)=   -578.4200   0.20257
Internal Energy (U)=   -578.4052   0.21734
Enthalpy (H)=   -578.4043   0.21828
Gibbs Free Energy (G)=   -578.4635   0.15902
Entropy (S)=   0.00019879

Frequencies --   13.5185   35.1296   68.6644
Supporting Information: 000-CsBr-PBE.log

Using Gaussian 16: ES64L-G16RevA.03 25-Dec-2016
==
# pbepbe/gen/auto pseudo=read gfprint gfinput empiricaldispersion=gd3bj
scf=(direct,vshift=200,tight,maxcycle=300,yqc) opt=(gdiis,maxcycle=250)
freq=noraman
#N Geom=AllCheck Guess=TCheck SCRF=Check GenChk RPBEPBE/Gen/Auto Freq
----
Pointgroup= C*V   Stoichiometry= BrCs   C*V[C*(BrCs)] #Atoms= 2
Charge = 0   Multiplicity = 1

SCF Energy= -2590.95045051   Predicted Change= -1.748887D-08
==
Optimization completed.   {Found   2   times}

Item	Max Val.		Criteria	Pass?	RMS Val.		Criteria	Pass?
Force	0.00003	||	0.00045	[YES]	0.00003	||	0.00030	[YES]
Displ	0.00051	||	0.00180	[YES]	0.00051	||	0.00180	[YES]

Atomic Type	Coordinates (Angstroms) X	Y	Z
Cs	0.000000	0.000000	1.277784
Br	0.000000	0.000000	-2.007946

Statistical Thermodynamic Analysis
Temperature= 298.150 Kelvin   Pressure= 1.00000 Atm
==
SCF Energy=   -2590.95045051   Predicted Change= -1.748887D-08
Zero-point correction (ZPE)=   -2590.9501   0.00030
Internal Energy (U)=   -2590.9471   0.00333
Enthalpy (H)=   -2590.9461   0.00428
Gibbs Free Energy (G)=   -2590.9767   -0.02628
Entropy (S)=   0.0001025

Frequencies --   132.3951
Supporting Information: 000-CsOAc-PBE.log

Using Gaussian 16: ES64L-G16RevA.03 25-Dec-2016
==
# pbepbe/gen/auto pseudo=read gfprint gfinput empiricaldispersion=gd3bj
scf=(direct,vshift=200,tight,maxcycle=300,yqc) opt=(gdiis,maxcycle=250)
freq=noraman
#N Geom=AllCheck Guess=TCheck SCRF=Check GenChk RPBEPBE/Gen/Auto Freq
----
Pointgroup= C1 Stoichiometry= C2H3CsO2 C1[X(C2H3CsO2)] #Atoms= 8
Charge = 0 Multiplicity = 1

SCF Energy= -248.154409254 Predicted Change= -7.599348D-10
==
Optimization completed. {Found 2 times}

Item	Max Val.		Criteria	Pass?	RMS Val.		Criteria	Pass?
Force	0.00000	||	0.00045	[YES]	0.00000	||	0.00030	[YES]
Displ	0.00043	||	0.00180	[YES]	0.00043	||	0.00180	[YES]

Atomic Type	Coordinates (Angstroms) X	Y	Z
C	2.011445	0.010334	-0.000038
O	1.438623	1.149667	-0.000031
O	1.442812	-1.132656	-0.000028
C	3.556698	-0.004141	-0.000009
H	3.923363	-0.551865	0.885966
H	3.923422	-0.552601	-0.885500
H	3.970998	1.015871	-0.000391
Cs	-1.241420	-0.001539	0.000012

Statistical Thermodynamic Analysis
Temperature= 298.150 Kelvin Pressure= 1.00000 Atm
==
SCF Energy= -248.154409254 Predicted Change= -7.599348D-10
Zero-point correction (ZPE)= -248.1059 0.04850
Internal Energy (U)= -248.0993 0.05506
Enthalpy (H)= -248.0984 0.05600
Gibbs Free Energy (G)= -248.1401 0.01429
Entropy (S)= 0.0001399

Frequencies -- 21.2782 58.6163 150.8599
Supporting Information: 000-p-Cymene-PBE.log

Using Gaussian 16: ES64L-G16RevA.03 25-Dec-2016
==
pbepbe/gen/auto pseudo=read gfprint gfinput empiricaldispersion=gd3bj

scf=(direct,vshift=200,tight,maxcycle=300,yqc) opt=(gdiis,maxcycle=250)
freq=noraman
#N Geom=AllCheck Guess=TCheck SCRF=Check GenChk RPBEPBE/Gen/Auto Freq

---

Pointgroup= C1 Stoichiometry= C10H14 C1[X(C10H14)] #Atoms= 24
Charge = 0 Multiplicity = 1

SCF Energy= -388.993122238 Predicted Change= -9.159088D-08

===

Optimization completed. {Found 1 times}

Item	Max Val.	Criteria	Pass?	RMS Val.	Criteria	Pass?
Force	0.00008 ||	0.00045	[YES]	0.00001 ||	0.00030	[YES]
Displ	0.04122 ||	0.00180	[NO]	0.04122 ||	0.00180	[NO]

Atomic Type	Coordinates (Angstroms) X	Y	Z
C	0.029697	-1.047787	-0.006383
C	-1.361354	-1.190802	-0.011888
C	-2.210103	-0.065942	-0.006650
C	-1.611201	1.204934	-0.001181
C	-0.216244	1.347561	0.004540
C	0.630698	0.227054	0.002476
H	0.659640	-1.944957	-0.012749
H	-1.801602	-2.195258	-0.022065
H	-2.246713	2.098416	-0.002495
H	0.224606	2.351764	0.006915
C	-3.712911	-0.227667	0.007524
H	-4.073726	-0.560876	0.998646
H	-4.222775	0.721591	-0.227209
H	-4.046223	-0.982815	-0.726113
C	2.145128	0.389072	0.005016
H	2.358713	1.475671	0.013541
C	2.776966	-0.222955	1.270475
H	3.868992	-0.057855	1.281119
H	2.349339	0.222112	2.184735
H	2.601696	-1.312720	1.313118
C	2.779258	-0.203468	-1.268569
H	2.353350	0.255770	-2.176601
H	3.871339	-0.038456	-1.274688
H	2.603752	-1.292390	-1.328318

Statistical Thermodynamic Analysis
Temperature= 298.150 Kelvin Pressure= 1.00000 Atm

===

SCF Energy= -388.993122238 Predicted Change= -9.159088D-08

Zero-point correction (ZPE)= -388.7852 0.20790
Internal Energy (U)= -388.7743 0.21873
Enthalpy (H)= -388.7734 0.21967
Gibbs Free Energy (G)= -388.8227 0.17037
Entropy (S)= 0.00016537

Frequencies -- 18.2418 42.3259 91.8253
Supporting Information: 000-PhSeBr-PBE.log

Using Gaussian 16: ES64L-G16RevA.03 25-Dec-2016

===

# pbepbe/gen/auto pseudo=read gfprint gfinput empiricaldispersion=gd3bj
scf=(direct,vshift=200,tight,maxcycle=300,yqc) opt=(gdiis,maxcycle=250)
freq=noraman
#N Geom=AllCheck Guess=TCheck SCRF=Check GenChk RPBEPBE/Gen/Auto Freq

---

Pointgroup= C1 Stoichiometry= C6H5BrSe C1[X(C6H5BrSe)] #Atoms= 13
Charge = 0 Multiplicity = 1

SCF Energy= -5200.92124785 Predicted Change= -1.923489D-09

===

Optimization completed. {Found 2 times}

Item	Max Val.	Criteria	Pass?	RMS Val.	Criteria	Pass?
Force	0.00002 ||	0.00045	[YES]	0.00000 ||	0.00030	[YES]
Displ	0.00052 ||	0.00180	[YES]	0.00052 ||	0.00180	[YES]

Atomic Type	Coordinates (Angstroms) X	Y	Z
C	2.966913	-0.421152	1.217493
C	1.664301	0.093091	1.221257
C	1.013424	0.351837	-0.000022
C	1.664279	0.092908	-1.221272
C	2.966891	-0.421336	-1.217454
C	3.616824	-0.677619	0.000032
H	3.475134	-0.622986	2.165697
H	1.142659	0.293784	2.161369
H	1.142623	0.293463	-2.161406
H	3.475093	-0.623312	-2.165638
H	4.635076	-1.080067	0.000054
Se	-0.742022	1.119907	-0.000058
Br	-2.057075	-0.869832	0.000049

Statistical Thermodynamic Analysis
Temperature= 298.150 Kelvin Pressure= 1.00000 Atm

===

SCF Energy= -5200.92124785 Predicted Change= -1.923489D-09
Zero-point correction (ZPE)= -5200.8317 0.08953
Internal Energy (U)= -5200.8237 0.09751
Enthalpy (H)= -5200.8227 0.09845
Gibbs Free Energy (G)= -5200.8681 0.05308
Entropy (S)= 0.00015217

Frequencies -- 19.6233 58.3299 188.1155
Supporting Information: 000-TriFluoroEthanol-PBE.log

Using Gaussian 16: ES64L-G16RevA.03 25-Dec-2016

===

# pbepbe/gen/auto pseudo=read gfprint gfinput empiricaldispersion=gd3bj
scf=(direct,vshift=200,tight,maxcycle=300,yqc) opt=(gdiis,maxcycle=250)
freq=noraman
#N Geom=AllCheck Guess=TCheck SCRF=Check GenChk RPBEPBE/Gen/Auto Freq

---

Pointgroup= C1 Stoichiometry= C2H3F3O C1[X(C2H3F3O)] #Atoms= 9
Charge = 0 Multiplicity = 1

SCF Energy= -452.297436102 Predicted Change= -2.961567D-07

===

Optimization completed. {Found 2 times}

Item	Max Val.	Criteria	Pass?	RMS Val.	Criteria	Pass?
Force	0.00023 ||	0.00045	[YES]	0.00006 ||	0.00030	[YES]
Displ	0.00074 ||	0.00180	[YES]	0.00074 ||	0.00180	[YES]

Atomic Type	Coordinates (Angstroms) X	Y	Z
C	0.422977	0.004486	-0.000002
C	-0.909331	0.744832	-0.000025
H	-0.917399	1.396480	-0.898471
H	-0.917399	1.396528	0.898384
O	-1.942796	-0.220569	-0.000001
H	-2.788634	0.267706	0.000115
F	0.563617	-0.779347	-1.095393
F	0.563635	-0.779245	1.095461
F	1.437629	0.915029	-0.000052

Statistical Thermodynamic Analysis
Temperature= 298.150 Kelvin Pressure= 1.00000 Atm

===

SCF Energy= -452.297436102 Predicted Change= -2.961567D-07
Zero-point correction (ZPE)= -452.2418 0.05558
Internal Energy (U)= -452.2356 0.06179

Enthalpy (H)= -452.2347 0.06273
Gibbs Free Energy (G)= -452.2720 0.02540
Entropy (S)= 0.00012521

Frequencies -- 99.8306 159.9175 218.2707
Supporting Information: 010-Cs-Cluster-RuII-1HOAc-2OAc-Cymene-01-PBE.log

---

Using Gaussian 16: ES64L-G16RevA.03 25-Dec-2016

===

# pbepbe/gen/auto pseudo=read gfprint gfinput empiricaldispersion=gd3bj
scf=(direct,vshift=200,tight,maxcycle=300,yqc) opt=(gdiis,maxcycle=250)
freq=noraman
#N Geom=AllCheck Guess=TCheck SCRF=Check GenChk RPBEPBE/Gen/Auto Freq

---

Pointgroup= C1 Stoichiometry= C20H37B10CsO8Ru C1[X(C20H37B10CsO8Ru)] #Atoms= 77
Charge = 0 Multiplicity = 1

SCF Energy= -1746.95073058 Predicted Change= -2.287821D-08

===

Optimization completed. {Found 1 times}

Item	Max Val.	Criteria	Pass?	RMS Val.	Criteria	Pass?
Force	0.00000 ||	0.00045	[YES]	0.00000 ||	0.00030	[YES]
Displ	0.01010 ||	0.00180	[NO]	0.01010 ||	0.00180	[YES]

Atomic Type	Coordinates (Angstroms) X	Y	Z
B	-4.539369	0.348195	1.446107
B	-5.944265	-0.274605	0.548191
H	-7.065884	0.081214	0.765916
H	-4.613126	1.169233	2.316396
B	-5.590411	-1.950730	0.081312
B	-5.415689	-0.650352	-1.111421
B	-3.324997	-0.948776	1.531836
B	-3.163710	0.337670	0.332320
H	-2.488726	-1.139231	2.360593
H	-2.410943	1.332334	0.277854
H	-6.058594	-0.657378	-2.118771
H	-6.354666	-2.847104	-0.118752
C	-2.898029	-1.266340	-0.102652
C	-4.230110	-1.868224	-0.954836
B	-5.046401	-1.353664	1.659354
B	-3.971340	-2.369146	0.673014
H	-3.575959	-3.478170	0.850192
H	-5.497837	-1.783648	2.680550
B	-4.763366	0.779799	-0.281572

H	-4.999181	1.893387	-0.651559
B	-3.691417	-0.258469	-1.251410
H	-3.126871	-0.039439	-2.276085
C	-1.466570	-1.776328	-0.389638
O	-0.522850	-0.990510	0.021490
O	-1.277058	-2.880161	-0.916503
Cs	2.299428	-2.173509	-0.839302
C	-4.031814	-2.894302	-2.059135
H	-4.127834	-2.412377	-3.043706
H	-3.037762	-3.355064	-1.967394
H	-4.806783	-3.669819	-1.964065
Ru	-0.675279	1.034065	0.555042
C	-0.766451	1.464487	2.743197
C	-0.941483	2.674988	1.983399
C	0.364504	0.629486	2.528198
H	-1.566002	1.136202	3.413929
C	0.006396	3.108454	1.002456
H	-1.873635	3.234217	2.108580
C	1.330431	1.047110	1.534291
C	1.152172	2.250155	0.800029
H	2.204944	0.395823	1.316205
H	1.870886	2.460279	0.004268
C	-0.234994	4.380551	0.194165
H	-0.163171	5.215587	0.921704
C	-1.646474	4.411057	-0.427561
H	-2.445901	4.335317	0.328693
H	-1.795844	5.359896	-0.970487
H	-1.760759	3.573107	-1.135522
C	0.828064	4.616379	-0.889673
H	0.625049	5.570585	-1.403575
H	1.848404	4.669080	-0.474517
H	0.806660	3.815227	-1.648385
C	0.541129	-0.672590	3.253754
H	-0.427379	-1.171915	3.422865
H	1.234979	-1.350587	2.721530
H	0.991447	-0.469972	4.244073
O	-0.847298	1.330407	-1.520104
C	0.128175	1.064578	-2.340640
O	1.236365	0.577849	-2.018960
C	-0.173354	1.426402	-3.790745
H	0.195124	0.629496	-4.456098
H	-1.246784	1.597888	-3.957428
H	0.376575	2.349510	-4.048179
O	4.466455	-0.239451	-2.039521
O	3.891075	-0.428407	1.351878
C	3.942858	-1.498772	2.060366

C	4.550261	0.846237	-1.466174
O	3.567831	1.782051	-1.538547
H	2.781228	1.340167	-1.982372
O	3.037460	-2.386332	2.122095
C	5.223869	-1.695242	2.890960
H	6.113730	-1.622196	2.241151
H	5.307619	-0.883065	3.635078
H	5.221366	-2.665245	3.411185
C	5.678253	1.255896	-0.560402
H	5.413309	0.832656	0.431803
H	6.619984	0.801777	-0.902012
H	5.774094	2.349312	-0.483291

Statistical Thermodynamic Analysis
Temperature= 298.150 Kelvin Pressure= 1.00000 Atm
==
SCF Energy= -1746.95073058 Predicted Change= -2.287821D-08
Zero-point correction (ZPE)= -1746.3750 0.57565
Internal Energy (U)= -1746.3299 0.62075
Enthalpy (H)= -1746.3290 0.62169
Gibbs Free Energy (G)= -1746.4563 0.49442
Entropy (S)= 0.00042686

Frequencies -- 18.3566 27.5977 29.4452
Supporting Information: 010-Cs-Cluster-RuII-1HOAc-2OAc-Cymene-02-PBE.log
----
Using Gaussian 16: ES64L-G16RevA.03 25-Dec-2016
==
# pbepbe/gen/auto pseudo=read gfprint gfinput empiricaldispersion=gd3bj
scf=(direct,vshift=200,tight,maxcycle=300,yqc) opt=(gdiis,maxcycle=250)
freq=noraman
#N Geom=AllCheck Guess=TCheck SCRF=Check GenChk RPBEPBE/Gen/Auto Freq
----
Pointgroup= C1 Stoichiometry= C20H37B10CsO8Ru C1[X(C20H37B10CsO8Ru)] #Atoms= 77
Charge = 0 Multiplicity = 1

SCF Energy= -1746.89386123 Predicted Change= -5.870618D-08
==
Optimization completed on the basis of negligible forces. {Found 2 times}

Item	Max Val.	Criteria	Pass?	RMS Val.	Criteria	Pass?
Force	0.00000 ||	0.00045	[YES]	0.00000 ||	0.00030	[YES]
Displ	0.02232 ||	0.00180	[NO]	0.02232 ||	0.00180	[YES]

Atomic Coordinates (Angstroms)
Type X Y Z

B	4.638560	-1.362407	0.140892
B	6.001465	-0.270834	-0.111745
H	7.140613	-0.634148	-0.139510
H	4.706981	-2.546591	0.306452
B	5.578385	1.309252	0.593858
B	5.450723	1.056251	-1.166487
B	3.361114	-0.441952	0.966315
B	3.244456	-0.685990	-0.726919
H	2.440246	-0.845659	1.726706
H	2.227916	-1.243917	-1.259716
H	6.116583	1.718592	-1.904128
H	6.328054	2.137461	1.016138
C	2.970578	0.880950	-0.062356
C	4.226658	1.878494	-0.292802
B	5.055755	-0.186197	1.420148
B	3.958247	1.203655	1.299252
H	3.517228	1.938313	2.127535
H	5.493658	-0.495698	2.489298
B	4.853244	-0.600706	-1.461055
H	5.147432	-1.204742	-2.450146
B	3.749525	0.787983	-1.584908
H	3.186953	1.273291	-2.515789
C	1.517041	1.350947	-0.013660
O	0.663917	0.380659	0.124347
O	1.226026	2.554794	-0.097961
Cs	-2.114785	1.050642	-1.786847
C	4.006523	3.370545	-0.494238
H	3.361999	3.559285	-1.363604
H	3.530589	3.820644	0.387434
H	4.988358	3.839464	-0.659512
Ru	1.389795	-1.537895	0.327850
C	0.313693	-1.463235	2.621793
C	-0.250731	-2.504209	1.824618
C	-0.352190	-0.221276	2.797398
H	1.201179	-1.687306	3.225130
C	-1.555988	-2.351583	1.254925
H	0.213272	-3.497779	1.856247
C	-1.588912	-0.067203	2.166982
C	-2.181251	-1.113929	1.427524
H	-2.130462	0.883299	2.226306
H	-3.146403	-0.928143	0.945388
C	-2.264357	-3.473534	0.497971
H	-2.230184	-3.197298	-0.573921
C	-3.756157	-3.565030	0.887179
H	-4.310301	-2.656829	0.596606
H	-4.221923	-4.420600	0.367137

H	-3.872004	-3.722789	1.974302
C	-1.573539	-4.838822	0.632463
H	-2.137931	-5.600690	0.068375
H	-0.546679	-4.815862	0.230567
H	-1.535340	-5.169731	1.686882
C	0.275008	0.886546	3.606416
H	1.361331	0.959960	3.412067
H	-0.183430	1.857999	3.359291
H	0.155314	0.718548	4.693227
O	0.277203	-2.619626	-0.967874
C	-0.037699	-2.198742	-2.180644
O	0.152322	-1.048506	-2.608400
C	-0.723065	-3.272917	-3.017021
H	-0.551919	-4.281500	-2.613042
H	-1.811728	-3.082645	-3.024409
H	-0.365368	-3.210257	-4.056768
O	-1.882317	3.934039	-0.611364
O	-4.631177	-0.397111	-0.567007
C	-4.903086	0.569782	0.221916
C	-1.961620	3.912520	0.617862
O	-0.947017	3.460101	1.397841
H	-0.216182	3.164031	0.779465
O	-4.067813	1.464517	0.585132
C	-6.317121	0.639529	0.818935
H	-7.021494	0.008233	0.255177
H	-6.285413	0.280830	1.864029
H	-6.676198	1.682012	0.844824
C	-3.180734	4.298782	1.408219
H	-3.695970	5.133690	0.910086
H	-3.847325	3.410236	1.388803
H	-2.934102	4.556458	2.448883

Statistical Thermodynamic Analysis
Temperature= 298.150 Kelvin Pressure= 1.00000 Atm

==

SCF Energy= -1746.89386123 Predicted Change= -5.870618D-08
Zero-point correction (ZPE)= -1746.3196 0.57425
Internal Energy (U)= -1746.2737 0.62011
Enthalpy (H)= -1746.2728 0.62105
Gibbs Free Energy (G)= -1746.4025 0.49127
Entropy (S)= 0.00043531

Frequencies -- 20.8071 23.5760 26.5670
Supporting Information: 010-Cs-Cluster-RuII-1HOAc-2OAc-Cymene-03-PBE.log

----

Using Gaussian 16: ES64L-G16RevA.03 25-Dec-2016

==

# pbepbe/gen/auto pseudo=read gfprint gfinput empiricaldispersion=gd3bj
scf=(direct,vshift=200,tight,maxcycle=300,yqc) opt=(gdiis,maxcycle=250)
freq=noraman
#N Geom=AllCheck Guess=TCheck SCRF=Check GenChk RPBEPBE/Gen/Auto Freq

----

Pointgroup= C1 Stoichiometry= C20H37B10CsO8Ru C1[X(C20H37B10CsO8Ru)] #Atoms= 77
Charge = 0 Multiplicity = 1

SCF Energy= -1746.96160651 Predicted Change= -3.464752D-07

==

Optimization completed. {Found 1 times}

Item	Max Val.		Criteria	Pass?	RMS Val.		Criteria	Pass?
Force	0.00003	||	0.00045	[YES]	0.00000	||	0.00030	[YES]
Displ	0.02701	||	0.00180	[NO]	0.02701	||	0.00180	[NO]

Atomic Type	Coordinates (Angstroms) X	Y	Z
B	-4.898935	0.583372	1.159569
B	-6.218991	-0.351619	0.413965
H	-7.371074	-0.041477	0.507399
H	-5.067775	1.586686	1.793523
B	-5.729248	-2.062012	0.393261
B	-5.596182	-1.085243	-1.082661
B	-3.605621	-0.553595	1.597882
B	-3.478864	0.397814	0.118078
H	-2.786429	-0.466719	2.458380
H	-2.742423	1.368981	-0.130229
H	-6.198933	-1.398318	-2.065957
H	-6.424448	-3.033924	0.404073
C	-3.096523	-1.242752	0.105394
C	-4.330747	-2.129400	-0.591455
B	-5.300200	-1.043809	1.784458
B	-4.119542	-2.186275	1.122309
H	-3.658507	-3.183966	1.580513
H	-5.766688	-1.237197	2.869289
B	-5.077895	0.552644	-0.624866
H	-5.376090	1.516333	-1.268858
B	-3.903719	-0.606817	-1.281306
H	-3.315647	-0.612859	-2.314560
C	-1.633426	-1.721500	-0.007872
O	-0.747235	-0.835466	0.329934
O	-1.382271	-2.889143	-0.326559
Cs	2.159029	-2.001993	-0.153045
C	-4.013905	-3.379200	-1.400512

H	-3.271128	-3.166031	-2.181482
H	-3.616236	-4.175215	-0.757977
H	-4.948421	-3.717326	-1.874457
Ru	-0.967378	1.244653	0.193512
C	-1.359084	2.903896	1.562799
C	-0.855785	3.460294	0.334080
C	-0.619935	1.950721	2.337999
H	-2.365109	3.185540	1.893407
C	0.373429	2.986170	-0.214107
H	-1.480328	4.153718	-0.232806
C	0.597306	1.469282	1.763076
C	1.083999	1.963305	0.513355
H	1.159431	0.633809	2.224480
H	1.998290	1.513313	0.114865
C	0.931680	3.470846	-1.536088
H	1.408110	2.590572	-2.003680
C	2.033693	4.512928	-1.239806
H	2.818071	4.097348	-0.584974
H	2.508091	4.833150	-2.183101
H	1.609512	5.406931	-0.748695
C	-0.128509	4.022271	-2.498836
H	0.335087	4.237764	-3.476195
H	-0.940126	3.291850	-2.652588
H	-0.564502	4.969239	-2.131455
C	-1.128321	1.430352	3.653128
H	-2.225002	1.509997	3.724644
H	-0.837184	0.377968	3.799342
H	-0.688552	2.016229	4.481434
O	-1.120447	0.821555	-1.845529
C	-0.120583	0.308971	-2.530293
O	1.033487	0.111353	-2.114177
C	-0.536156	-0.076160	-3.949660
H	-0.991479	-1.083022	-3.926835
H	-1.288220	0.619104	-4.353819
H	0.348293	-0.108839	-4.603229
O	5.216281	-1.876760	-0.535018
O	2.644471	-0.579395	2.752813
C	3.711586	-0.174448	2.217084
C	6.055010	-0.973352	-0.678740
O	5.947424	0.251870	-0.183428
H	5.035280	0.340527	0.374647
O	3.761275	0.436402	1.073204
C	5.050094	-0.465932	2.903137
H	5.707821	0.418571	2.873298
H	4.892471	-0.788202	3.942652
H	5.567019	-1.272865	2.352004

C	7.346193	-1.174416	-1.456093
H	7.383390	-2.189736	-1.875238
H	7.421137	-0.429583	-2.266331
H	8.212383	-1.014365	-0.791478

Statistical Thermodynamic Analysis
Temperature= 298.150 Kelvin   Pressure= 1.00000 Atm

===

SCF Energy=   -1746.96160651   Predicted Change= -3.464752D-07
Zero-point correction (ZPE)=   -1746.3873   0.57430
Internal Energy (U)=   -1746.3418   0.61978
Enthalpy (H)=   -1746.3408   0.62073
Gibbs Free Energy (G)=   -1746.4709   0.49068
Entropy (S)=   0.00043618

Frequencies --   13.3262   20.7610   21.5179
Supporting Information: 010-Cs-Cluster-RuII-1HOAc-2OAc-Cymene-04-PBE.log

----

Using Gaussian 16: ES64L-G16RevA.03 25-Dec-2016

===

# pbepbe/gen/auto pseudo=read gfprint gfinput empiricaldispersion=gd3bj
scf=(direct,vshift=200,tight,maxcycle=300,yqc) opt=(gdiis,maxcycle=250)
freq=noraman
#N Geom=AllCheck Guess=TCheck SCRF=Check GenChk RPBEPBE/Gen/Auto Freq

----

Pointgroup= C1 Stoichiometry= C20H37B10CsO8Ru C1[X(C20H37B10CsO8Ru)] #Atoms= 77
Charge = 0   Multiplicity = 1

SCF Energy= -1746.90705088   Predicted Change= -7.555846D-08

===

Optimization completed.   {Found   1   times}

Item	Max Val.	Criteria	Pass?	RMS Val.	Criteria	Pass?
Force	0.00000 ||	0.00045	[YES]	0.00000 ||	0.00030	[YES]
Displ	0.00583 ||	0.00180	[NO]	0.00583 ||	0.00180	[YES]

Atomic Type	Coordinates (Angstroms) X	Y	Z
B	4.182291	-0.632552	0.143613
B	4.626433	-2.230457	0.799019
H	5.647106	-2.425521	1.392486
H	4.858618	0.351614	0.260848
B	3.929546	-3.470878	-0.269501
B	3.123662	-3.075643	1.260959
B	3.219736	-0.900599	-1.329308
B	2.417001	-0.536951	0.200099

H	3.092880	-0.197687	-2.287495
H	1.870638	0.498335	0.690782
H	2.973698	-3.910655	2.101186
H	4.328280	-4.579179	-0.467203
C	1.826013	-1.755783	-0.791746
C	2.227929	-3.261421	-0.188715
B	4.585261	-1.973391	-0.970305
B	3.064676	-2.654898	-1.576399
H	2.796088	-3.136743	-2.630530
H	5.557897	-1.978448	-1.667428
B	3.274000	-1.323811	1.528799
H	3.296181	-0.840956	2.625259
B	1.756550	-2.011220	0.912952
H	0.677533	-2.077333	1.400859
C	0.515336	-1.560365	-1.570536
O	-0.017578	-0.369637	-1.455733
O	0.050781	-2.455399	-2.272432
Cs	-3.100963	-1.271061	-0.974075
C	1.260535	-4.419676	-0.359833
H	0.277597	-4.175530	0.096667
H	1.116728	-4.648712	-1.424445
H	1.699428	-5.293828	0.146656
Ru	0.712452	1.254805	-0.375407
C	1.081276	2.273120	-2.285179
C	2.188084	2.523126	-1.433852
C	-0.271676	2.617117	-1.892947
H	1.241750	1.728135	-3.220856
C	1.999945	3.097450	-0.121052
H	3.187020	2.190034	-1.730060
C	-0.463430	3.144924	-0.580473
C	0.663432	3.391281	0.285012
H	-1.494586	3.182078	-0.132007
H	0.464982	3.760001	1.295887
C	3.215455	3.373095	0.748273
H	4.055749	2.796491	0.318687
C	3.069985	2.950761	2.217308
H	2.981821	1.854225	2.315876
H	3.971328	3.247528	2.779171
H	2.209475	3.433978	2.712782
C	3.550124	4.877330	0.627294
H	4.490645	5.097991	1.159462
H	3.671086	5.182800	-0.425839
H	2.749703	5.493794	1.072657
C	-1.444567	2.304809	-2.766840
H	-2.339603	2.100128	-2.134960
H	-1.670099	3.182660	-3.401800

H	-1.226110	1.450859	-3.429950
O	-0.757192	0.484642	0.980399
C	-0.839944	0.784206	2.201428
O	0.157564	1.400233	2.841016
C	-2.050711	0.503940	3.004896
H	-2.788531	1.264480	2.678561
H	-2.450029	-0.511228	2.764619
H	-1.842839	0.623605	4.077431
O	-1.426069	-3.288182	0.664809
O	-2.915508	2.888911	0.949123
C	-3.837959	2.242312	0.347826
C	-1.917340	-2.938952	1.789278
O	-2.941314	-2.194617	1.947879
O	-3.762222	1.692723	-0.799227
C	-5.163557	2.059192	1.115437
H	-5.152815	1.082221	1.636683
H	-5.295357	2.845664	1.875218
H	-6.020867	2.055063	0.422586
C	-1.199737	-3.408739	3.070752
H	-1.934849	-3.737073	3.824059
H	-0.482420	-4.219774	2.866564
H	-0.645156	-2.559543	3.514509
H	0.873481	1.563718	2.170845

Statistical Thermodynamic Analysis
Temperature= 298.150 Kelvin Pressure= 1.00000 Atm
==
SCF Energy= -1746.90705088 Predicted Change= -7.555846D-08
Zero-point correction (ZPE)= -1746.3323 0.57465
Internal Energy (U)= -1746.2879 0.61911
Enthalpy (H)= -1746.2869 0.62006
Gibbs Free Energy (G)= -1746.4108 0.49618
Entropy (S)= 0.00041547

Frequencies -- 16.7394 26.6341 30.4243
Supporting Information: 010-Cs-Cluster-RuII-1HOAc-2OAc-Cymene-05-PBE.log
----
Using Gaussian 16: ES64L-G16RevA.03 25-Dec-2016
==
# pbepbe/gen/auto pseudo=read gfprint gfinput empiricaldispersion=gd3bj
scf=(direct,vshift=200,tight,maxcycle=300,yqc) opt=(gdiis,maxcycle=250)
freq=noraman
#N Geom=AllCheck Guess=TCheck SCRF=Check GenChk RPBEPBE/Gen/Auto Freq
----
Pointgroup= C1 Stoichiometry= C20H37B10CsO8Ru C1[X(C20H37B10CsO8Ru)] #Atoms= 77
Charge = 0 Multiplicity = 1

SCF Energy= -1746.96435053 Predicted Change= -4.106986D-09
==

Optimization completed on the basis of negligible forces. {Found 2 times}

Item	Max Val.	Criteria	Pass?	RMS Val.	Criteria	Pass?
Force	0.00000 ||	0.00045	[YES]	0.00000 ||	0.00030	[YES]
Displ	0.00200 ||	0.00180	[NO]	0.00200 ||	0.00180	[YES]

Atomic Type	Coordinates (Angstroms) X	Y	Z
B	4.517919	-1.261727	0.025084
B	5.845256	-0.125966	-0.199907
H	6.996229	-0.452430	-0.241162
H	4.616544	-2.445795	0.157661
B	5.378224	1.417550	0.552439
B	5.255447	1.211605	-1.215532
B	3.207955	-0.412125	0.880852
B	3.090760	-0.607738	-0.814213
H	2.309483	-0.874749	1.659235
H	2.085244	-1.196172	-1.340438
H	5.902618	1.914702	-1.933604
H	6.105433	2.256410	0.995343
C	2.784822	0.947183	-0.115585
C	4.016372	1.976128	-0.317698
B	4.899352	-0.113572	1.335214
B	3.759353	1.244002	1.257026
H	3.309607	1.954534	2.103064
H	5.354904	-0.437193	2.393656
B	4.698347	-0.451973	-1.553860
H	5.011457	-1.017322	-2.560996
B	3.559767	0.905115	-1.635819
H	2.987677	1.409687	-2.552313
C	1.320288	1.386491	-0.075530
O	0.496424	0.420340	0.113111
O	1.021506	2.594816	-0.245117
Cs	-1.906084	1.305274	-1.628489
C	3.773224	3.470259	-0.471606
H	3.172284	3.679682	-1.366931
H	3.240737	3.874761	0.399904
H	4.751812	3.965776	-0.563927
Ru	1.305057	-1.545160	0.264040
O	-0.046320	-2.526378	-0.941163
C	-0.381054	-2.087599	-2.124128
O	-0.114787	-0.966267	-2.608597
C	-1.157592	-3.117124	-2.949748

H	-0.528840	-3.427351	-3.802445
H	-1.424059	-4.008798	-2.363024
H	-2.068668	-2.656939	-3.369622
O	-0.411124	4.173523	1.364017
C	-1.661963	4.424568	0.958567
O	-2.180565	3.924183	-0.044294
C	-2.385212	5.365288	1.897935
H	-2.648767	4.817343	2.820062
H	-1.736354	6.205834	2.190605
H	-3.305269	5.734379	1.424346
C	-3.440768	-2.078252	-0.254422
C	-4.632826	-1.576632	-0.816033
C	-2.912399	-1.555656	0.935379
H	-2.903091	-2.890545	-0.754974
C	-5.275988	-0.516916	-0.144454
C	-3.567793	-0.509306	1.617983
H	-1.971177	-1.965646	1.316127
C	-4.754509	0.000324	1.051739
H	-6.208092	-0.105991	-0.552460
H	-5.282374	0.816145	1.561731
C	-5.215593	-2.176270	-2.076703
H	-5.863520	-1.458415	-2.609010
H	-4.425010	-2.509866	-2.771496
H	-5.834574	-3.063916	-1.847649
C	-3.001509	0.113742	2.889010
H	-3.854571	0.566017	3.433101
C	-2.326985	-0.904892	3.823390
H	-2.021369	-0.410105	4.761912
H	-3.015500	-1.729158	4.078226
H	-1.423512	-1.341569	3.364356
C	-2.026141	1.253799	2.525009
H	-1.155909	0.861470	1.969680
H	-2.525347	2.010102	1.893958
H	-1.651130	1.759188	3.432824
H	0.025015	3.523221	0.720565
O	0.206933	-2.456140	1.831479
C	0.858307	-3.564539	1.663214
O	1.805620	-3.562863	0.802193
C	0.483373	-4.796824	2.435849
H	0.166823	-4.526377	3.455502
H	-0.366652	-5.294044	1.935764
H	1.330292	-5.498095	2.469090

Statistical Thermodynamic Analysis

Temperature= 298.150 Kelvin   Pressure= 1.00000 Atm

===

SCF Energy=   -1746.96435053   Predicted Change= -4.106986D-09
Zero-point correction (ZPE)=   -1746.3893   0.57504
Internal Energy (U)=   -1746.3437   0.62064
Enthalpy (H)=   -1746.3427   0.62159
Gibbs Free Energy (G)=   -1746.4728   0.49147
Entropy (S)=   0.0004364

Frequencies --   16.5173   17.5178   25.5017

Supporting Information: 010-Cs-Cluster-RuII-1HOAc-2OAc-Cymene-06-PBE.log

----

Using Gaussian 16: ES64L-G16RevA.03 25-Dec-2016

==

# pbepbe/gen/auto pseudo=read gfprint gfinput empiricaldispersion=gd3bj
scf=(direct,vshift=200,tight,maxcycle=300,yqc) opt=(gdiis,maxcycle=250)
freq=noraman
#N Geom=AllCheck Guess=TCheck SCRF=Check GenChk RPBEPBE/Gen/Auto Freq

----

Pointgroup= C1   Stoichiometry= C20H37B10CsO8Ru   C1[X(C20H37B10CsO8Ru)] #Atoms= 77
Charge = 0   Multiplicity = 1

SCF Energy= -1746.95261800   Predicted Change= -1.362768D-08

==

Optimization completed.   {Found   1   times}

Item	Max Val.	Criteria	Pass?	RMS Val.	Criteria	Pass?
Force	0.00002 ||	0.00045	[YES]	0.00000 ||	0.00030	[YES]
Displ	0.01154 ||	0.00180	[NO]	0.01154 ||	0.00180	[YES]

Atomic Type	Coordinates (Angstroms) X	Y	Z
B	4.798717	0.410434	0.417353
B	5.886681	-0.944303	0.028143
H	7.038732	-0.967933	0.351156
H	5.135232	1.381474	1.033987
B	5.354254	-1.641494	-1.519903
B	4.920002	-2.437579	0.006190
B	3.604405	0.543830	-0.891852
B	3.175914	-0.268395	0.613047
H	3.005744	1.504797	-1.260178
H	2.433245	0.147850	1.518504
H	5.274440	-3.558580	0.220305
H	6.004717	-2.227777	-2.332971
C	2.749232	-0.949832	-0.867853
C	3.768609	-2.221759	-1.242828
B	5.281429	0.116549	-1.279983
B	3.947191	-0.724966	-2.086676

H	3.571953	-0.689213	-3.215971
H	5.975299	0.863356	-1.906474
B	4.568103	-1.180719	1.213748
H	4.741554	-1.357612	2.384564
B	3.242145	-2.026189	0.389108
H	2.435190	-2.794933	0.803482
C	1.282486	-0.979787	-1.351254
O	0.487694	-0.148795	-0.746250
O	0.952912	-1.712542	-2.288808
Cs	-2.540818	-0.399510	-1.646137
C	3.242209	-3.426760	-2.009562
H	2.335530	-3.830703	-1.538692
H	2.999299	-3.160046	-3.046133
H	4.027787	-4.198175	-1.999646
Ru	0.753972	0.713817	1.143870
C	-1.159096	1.681557	1.647858
C	-0.595995	2.382351	0.542722
C	-0.411015	1.498953	2.871256
H	-2.158043	1.256210	1.514003
C	0.732138	2.920602	0.585717
H	-1.193944	2.417790	-0.394349
C	0.915952	2.018943	2.926733
C	1.484695	2.680549	1.779215
H	1.544059	1.810572	3.797789
H	2.546086	2.952326	1.800248
C	1.314884	3.708494	-0.570528
H	2.414244	3.579781	-0.536411
C	0.998506	5.203474	-0.337081
H	1.387395	5.559801	0.632738
H	1.451792	5.813746	-1.137001
H	-0.092738	5.370461	-0.356937
C	0.796695	3.251371	-1.943905
H	1.326631	3.803893	-2.738617
H	0.970139	2.172896	-2.096226
H	-0.286465	3.437105	-2.053419
C	-0.996998	0.712771	4.008770
H	-0.212591	0.237735	4.619366
H	-1.581908	1.388974	4.659266
H	-1.678257	-0.062425	3.625906
O	0.483050	-1.272807	1.776903
C	-0.644285	-1.908596	1.692904
O	-1.742389	-1.394681	1.350154
C	-0.535466	-3.395425	2.008234
H	0.307981	-3.598036	2.684282
H	-1.476186	-3.773940	2.436184
H	-0.353610	-3.937335	1.062176

O	-3.929029	-2.990311	-0.629903
O	-3.785053	1.928211	-0.008955
C	-3.539755	2.752506	-0.943647
C	-4.333596	-3.394931	0.462488
O	-3.823914	-3.012198	1.642808
H	-3.046320	-2.365288	1.475256
O	-2.507365	2.729518	-1.709650
C	-4.537233	3.902994	-1.174039
H	-5.526886	3.664245	-0.753735
H	-4.159302	4.810439	-0.667371
H	-4.621657	4.142757	-2.246954
C	-5.475950	-4.375920	0.628690
H	-6.309067	-3.889646	1.164676
H	-5.821001	-4.719933	-0.355785
H	-5.156391	-5.235844	1.240620

Statistical Thermodynamic Analysis
Temperature= 298.150 Kelvin Pressure= 1.00000 Atm

==

SCF Energy= -1746.95261800 Predicted Change= -1.362768D-08
Zero-point correction (ZPE)= -1746.3775 0.57506
Internal Energy (U)= -1746.3319 0.62068
Enthalpy (H)= -1746.3309 0.62163
Gibbs Free Energy (G)= -1746.4612 0.49138
Entropy (S)= 0.00043684

Frequencies -- 15.9346 21.5597 25.9965

Supporting Information: 010-Cs-Cluster-RuII-1HOAc-2OAc-PhSeBr-01-PBE.log

----

Using Gaussian 16: ES64L-G16RevA.03 25-Dec-2016

==

# pbepbe/gen/auto pseudo=read gfprint gfinput empiricaldispersion=gd3bj
scf=(direct,vshift=200,tight,maxcycle=300,yqc) opt=(gdiis,maxcycle=250)
freq=noraman
#N Geom=AllCheck Guess=TCheck SCRF=Check GenChk RPBEPBE/Gen/Auto Freq

----

Pointgroup= C1 Stoichiometry= C16H28B10BrCsO8RuSe C1[X(C16H28B10BrCsO8RuSe)]
#Atoms= 66
Charge = 0 Multiplicity = 1

SCF Energy= -6558.92848090 Predicted Change= -1.806434D-08

==

Optimization completed on the basis of negligible forces. {Found 2 times}

Item	Max Val.	Criteria	Pass?	RMS Val.	Criteria	Pass?
Force	0.00000 ||	0.00045	[YES]	0.00000 ||	0.00030	[YES]
Displ	0.01282 ||	0.00180	[NO]	0.01282 ||	0.00180	[YES]

Atomic Type	Coordinates (Angstroms) X	Y	Z
B	-2.494280	2.742256	0.069825
B	-2.220141	4.431736	0.556775
H	-3.099611	5.149866	0.937045
H	-3.575361	2.232955	0.085763
B	-0.879952	5.075467	-0.413827
B	-0.588030	4.541955	1.254057
B	-1.313622	2.353357	-1.192523
B	-1.031656	1.783272	0.480918
H	-1.392095	1.574752	-2.098618
H	-1.447573	0.682414	1.170300
H	-0.170289	5.309918	2.070045
H	-0.657803	6.207450	-0.727965
C	0.208301	2.529027	-0.454619
C	0.478278	4.120204	-0.005647
B	-2.045065	3.965544	-1.160046
B	-0.314405	3.796470	-1.497958
H	0.308806	3.964405	-2.499295
H	-2.785162	4.322678	-2.030736
B	-1.586927	3.102042	1.568639
H	-1.995375	2.862370	2.663556
B	0.135553	2.925091	1.209468
H	1.063820	2.606676	1.883868
C	1.348555	1.627476	-0.915160
O	1.204801	0.389259	-0.586075
O	2.309350	2.094511	-1.565903
Cs	3.399303	-1.239994	0.923791
C	1.886920	4.690747	-0.052871
H	2.345213	4.658242	0.947270
H	2.505681	4.120386	-0.760307
H	1.830371	5.739215	-0.382691
Ru	-0.331270	-0.417580	0.616415
O	0.923304	0.019778	2.249562
O	-1.339152	-1.866860	1.804810
C	0.341051	0.377940	3.386910
C	-0.452983	-2.757796	1.540736
O	-0.866580	0.564114	3.557098
O	0.542262	-2.411769	0.790655
C	-0.600172	-4.161090	2.047493
H	0.385018	-4.630357	2.194911
H	-1.179197	-4.173393	2.983050
H	-1.149250	-4.738533	1.281706
C	1.352484	0.600641	4.516159

H	1.890772	1.550125	4.342464
H	0.830051	0.664428	5.481550
H	2.103627	-0.207853	4.554781
Se	-1.563437	-0.965158	-1.338650
Br	-1.891879	-3.455616	-1.434895
C	-3.409424	-0.533105	-0.972072
C	-4.169368	-0.000193	-2.025583
C	-3.950809	-0.687029	0.314725
C	-5.486435	0.410661	-1.778824
H	-3.727955	0.109879	-3.020997
C	-5.273164	-0.288738	0.543485
H	-3.339245	-1.105851	1.118657
C	-6.036644	0.266133	-0.496827
H	-6.080407	0.846027	-2.588584
H	-5.703297	-0.398329	1.544026
H	-7.064821	0.590089	-0.306076
O	4.978091	-0.302653	-1.455634
C	4.938398	0.029747	-2.646151
O	4.027546	0.849304	-3.171222
C	5.930839	-0.453242	-3.684203
H	6.647231	-1.149381	-3.226984
H	5.399390	-0.948999	-4.513777
H	6.470533	0.406431	-4.116163
H	3.394444	1.189830	-2.450430

Statistical Thermodynamic Analysis
Temperature= 298.150 Kelvin   Pressure= 1.00000 Atm

==

SCF Energy=   -6558.92848090   Predicted Change= -1.806434D-08
Zero-point correction (ZPE)=   -6558.4720   0.45646
Internal Energy (U)=   -6558.4292   0.49924
Enthalpy (H)=   -6558.4282   0.50018
Gibbs Free Energy (G)=   -6558.5530   0.37543
Entropy (S)=   0.00041843

Frequencies --   9.9255   23.8952   29.2757
Supporting Information: 010-Cs-Cluster-RuII-1HOAc-2OAc-PhSeBr-02-PBE.log

----

Using Gaussian 16: ES64L-G16RevA.03 25-Dec-2016

==

# pbepbe/gen/auto pseudo=read gfprint gfinput empiricaldispersion=gd3bj
scf=(direct,vshift=200,tight,maxcycle=300,yqc) opt=(gdiis,maxcycle=250)
freq=noraman
#N Geom=AllCheck Guess=TCheck SCRF=Check GenChk RPBEPBE/Gen/Auto Freq

----

Pointgroup= C1 Stoichiometry= C16H28B10BrCsO8RuSe C1[X(C16H28B10BrCsO8RuSe)]
#Atoms= 66
Charge = 0 Multiplicity = 1

SCF Energy= -6558.91673976 Predicted Change= -3.083340D-08
===
Optimization completed. {Found 1 times}

Item	Max Val.	Criteria	Pass?	RMS Val.	Criteria	Pass?
Force	0.00001 ||	0.00045	[YES]	0.00000 ||	0.00030	[YES]
Displ	0.01343 ||	0.00180	[NO]	0.01343 ||	0.00180	[YES]

Atomic Type	Coordinates (Angstroms) X	Y	Z
B	-1.110401	4.382980	-0.283720
B	-2.705250	4.832761	-0.937442
H	-2.938757	5.906924	-1.411121
H	-0.163089	5.114536	-0.283714
B	-3.942384	3.958919	-0.005054
B	-3.454949	3.366533	-1.606080
B	-1.362767	3.237892	1.056146
B	-0.900680	2.643183	-0.547992
H	-0.677227	3.028105	2.007507
H	0.192382	2.248228	-0.986173
H	-4.263598	3.268485	-2.481243
H	-5.079209	4.266342	0.200479
C	-2.136418	1.880608	0.337967
C	-3.640138	2.301149	-0.281140
B	-2.499753	4.587008	0.823000
B	-3.119445	2.979166	1.221656
H	-3.638963	2.564912	2.209961
H	-2.581142	5.468133	1.628535
B	-1.710415	3.618175	-1.789130
H	-1.204197	3.792027	-2.859100
B	-2.337416	2.009909	-1.373273
H	-2.354459	0.995434	-1.999480
C	-1.941747	0.493733	0.970530
O	-0.744153	0.035009	0.969209
O	-2.917373	-0.116006	1.461449
Cs	-1.528888	-2.734898	-0.225679
C	-4.788256	1.303734	-0.286091
H	-4.451892	0.302652	-0.591300
H	-5.259439	1.231389	0.703184
H	-5.535471	1.658816	-1.012568
Ru	0.909767	0.876153	-0.055517
O	0.178417	-0.328943	-1.543618

O	2.614940	1.947395	-0.795326
C	0.976933	-0.638069	-2.567097
C	2.681270	2.662348	0.270636
O	2.136768	-0.261143	-2.729763
O	1.791699	2.455057	1.180731
C	3.791878	3.653776	0.465297
H	3.531252	4.377276	1.251518
H	4.703118	3.105004	0.765286
H	4.007444	4.173067	-0.481481
C	0.306146	-1.601428	-3.548049
H	-0.791947	-1.495700	-3.557514
H	0.701687	-1.434245	-4.560988
H	0.573723	-2.633727	-3.252520
Se	2.145214	-0.725844	1.084569
Br	2.122539	-3.063053	-0.163496
C	4.004756	-0.347402	0.768228
C	4.823363	-0.202732	1.901669
C	4.507041	-0.171531	-0.532434
C	6.170134	0.148675	1.730350
H	4.411081	-0.352820	2.904788
C	5.858060	0.160976	-0.685982
H	3.836384	-0.273203	-1.392347
C	6.685747	0.327623	0.437802
H	6.814933	0.273008	2.606163
H	6.263488	0.302830	-1.692842
H	7.739506	0.594220	0.304887
O	-4.593201	-2.559080	0.101213
C	-5.482361	-2.215088	0.885864
O	-5.356118	-1.207510	1.762936
C	-6.843357	-2.869219	0.967400
H	-6.887047	-3.732539	0.289953
H	-7.053354	-3.186011	2.002435
H	-7.623759	-2.140215	0.689467
H	-4.447342	-0.792494	1.645506

Statistical Thermodynamic Analysis
Temperature= 298.150 Kelvin   Pressure= 1.00000 Atm

===

SCF Energy=   -6558.91673976   Predicted Change= -3.083340D-08
Zero-point correction (ZPE)=   -6558.4592   0.45753
Internal Energy (U)=   -6558.4163   0.50040
Enthalpy (H)=   -6558.4153   0.50134
Gibbs Free Energy (G)=   -6558.5406   0.37606
Entropy (S)=   0.00042018

Frequencies --   16.8757   21.4586   25.8467

Supporting Information: 010-Cs-Cluster-RuII-1HOAc-2OAc-PhSeBr-03-PBE.log

----

Using Gaussian 16: ES64L-G16RevA.03 25-Dec-2016

==

# pbepbe/gen/auto pseudo=read gfprint gfinput empiricaldispersion=gd3bj
scf=(direct,vshift=200,tight,maxcycle=300,yqc) opt=(gdiis,maxcycle=250)
freq=noraman
#N Geom=AllCheck Guess=TCheck SCRF=Check GenChk RPBEPBE/Gen/Auto Freq

----

Pointgroup= C1 Stoichiometry= C16H28B10BrCsO8RuSe C1[X(C16H28B10BrCsO8RuSe)]
#Atoms= 66
Charge = 0 Multiplicity = 1

SCF Energy= -6558.92475121 Predicted Change= -2.416181D-08

==

Optimization completed on the basis of negligible forces. {Found 2 times}

Item	Max Val.	Criteria	Pass?	RMS Val.	Criteria	Pass?
Force	0.00000 ||	0.00045	[YES]	0.00000 ||	0.00030	[YES]
Displ	0.00935 ||	0.00180	[NO]	0.00935 ||	0.00180	[YES]

Atomic Type	Coordinates (Angstroms) X	Y	Z
B	0.777785	4.302696	0.784090
B	0.001790	5.481607	-0.305649
H	0.469948	6.557206	-0.544653
H	1.818444	4.505745	1.340208
B	-1.759723	5.261879	-0.183910
B	-0.873538	4.574351	-1.560363
B	-0.505114	3.361672	1.577657
B	0.381177	2.665228	0.199212
H	-0.502958	2.817011	2.636684
H	1.455530	1.930219	0.090840
H	-1.141339	4.906907	-2.677107
H	-2.625220	6.065121	-0.370196
C	-1.324513	2.574099	0.293347
C	-2.061912	3.669877	-0.726455
B	-0.749689	5.095868	1.270660
B	-2.072011	3.961245	0.977109
H	-3.104402	3.794879	1.546805
H	-0.830461	5.875523	2.175130
B	0.694920	3.978668	-0.974161
H	1.658904	3.943875	-1.683502
B	-0.632977	2.848520	-1.254158
H	-0.774862	1.995501	-2.076128
C	-1.972555	1.213269	0.539013

O	-1.144134	0.252612	0.749368
O	-3.219946	1.105144	0.552913
Cs	-2.098496	-2.583283	1.310242
C	-3.373079	3.308125	-1.408587
H	-3.294297	2.357469	-1.955742
H	-4.185837	3.219152	-0.675585
H	-3.612572	4.107787	-2.126269
Ru	0.982232	0.445312	0.803601
O	0.799566	-1.360606	1.767763
O	3.045365	0.706618	1.279961
C	1.800207	-2.202897	1.983848
C	2.757816	1.168837	2.445396
O	2.979233	-2.077579	1.646558
O	1.513371	1.309906	2.744463
C	3.846816	1.488572	3.428616
H	3.495293	2.228376	4.162960
H	4.128909	0.562700	3.960647
H	4.738070	1.861163	2.900647
C	1.330964	-3.478397	2.697549
H	0.974981	-4.194990	1.932982
H	2.179882	-3.943440	3.219746
H	0.520363	-3.281539	3.420449
Se	0.906833	-0.387018	-1.385992
C	2.715816	-0.498536	-2.028577
C	2.935476	-0.092715	-3.357499
C	3.767968	-0.931639	-1.204813
C	4.240948	-0.099464	-3.865489
H	2.095476	0.228815	-3.981829
C	5.064145	-0.943921	-1.733924
H	3.567876	-1.250815	-0.175278
C	5.303000	-0.526410	-3.053900
H	4.425992	0.223214	-4.895005
H	5.894803	-1.281865	-1.105981
H	6.322205	-0.537530	-3.454225
O	-4.417061	-2.043152	-0.578191
C	-4.830635	-1.278394	-1.452987
O	-4.651818	0.050578	-1.431425
C	-5.592327	-1.726676	-2.680988
H	-5.788780	-2.805905	-2.627415
H	-6.541012	-1.171459	-2.766620
H	-5.002561	-1.502381	-3.586335
H	-4.129619	0.307094	-0.603735
Br	0.455871	-2.941189	-1.375160

Statistical Thermodynamic Analysis
Temperature= 298.150 Kelvin Pressure= 1.00000 Atm

==
SCF Energy= -6558.92475121 Predicted Change= -2.416181D-08
Zero-point correction (ZPE)= -6558.4678 0.45689
Internal Energy (U)= -6558.4249 0.49981
Enthalpy (H)= -6558.4239 0.50075
Gibbs Free Energy (G)= -6558.5511 0.37361
Entropy (S)= 0.00042642

Frequencies -- 7.0223 10.5995 23.1443
Supporting Information: 010-Cs-Cluster-RuII-1HOAc-2OAc-PhSeBr-Cymene-01-PBE.log
----
Using Gaussian 16: ES64L-G16RevA.03 25-Dec-2016
==
# pbepbe/gen/auto pseudo=read gfprint gfinput empiricaldispersion=gd3bj
scf=(direct,vshift=200,tight,maxcycle=300,yqc) opt=(gdiis,maxcycle=250)
freq=noraman
#N Geom=AllCheck Guess=TCheck SCRF=Check GenChk RPBEPBE/Gen/Auto Freq
----
Pointgroup= C1 Stoichiometry= C26H42B10BrCsO8RuSe C1[X(C26H42B10BrCsO8RuSe)]
#Atoms= 90
Charge = 0 Multiplicity = 1

SCF Energy= -6947.90972047 Predicted Change= -1.794739D-08
==
Optimization completed. {Found 1 times}

Item	Max Val.		Criteria	Pass?	RMS Val.		Criteria	Pass?
Force	0.00001	||	0.00045	[YES]	0.00000	||	0.00030	[YES]
Displ	0.01065	||	0.00180	[NO]	0.01065	||	0.00180	[YES]

Atomic Type	Coordinates (Angstroms) X	Y	Z
B	4.540950	2.054065	-0.192525
B	6.006580	1.355777	0.542104
H	6.799059	2.008726	1.156873
H	4.259377	3.216970	-0.116017
B	6.530622	-0.007448	-0.469760
B	5.634838	-0.318198	1.027126
B	4.176474	1.119210	-1.658876
B	3.274531	0.802613	-0.156563
H	3.643500	1.458712	-2.670453
H	2.114807	1.000139	0.403476
H	6.116981	-0.968431	1.905968
H	7.630755	-0.450197	-0.615465
C	3.860484	-0.465084	-1.100068
C	5.248172	-1.142347	-0.419343

B	5.864499	1.446765	-1.236586
B	5.397464	-0.162686	-1.822455
H	5.626345	-0.709244	-2.854836
H	6.534929	2.159680	-1.925204
B	4.403416	0.951981	1.211056
H	4.027317	1.308663	2.288051
B	3.951306	-0.656784	0.601432
H	3.291123	-1.512923	1.090237
C	2.857392	-1.310724	-1.907197
O	1.645177	-0.844352	-1.899492
O	3.230908	-2.304118	-2.537822
Cs	-3.641335	-1.933520	-0.327374
C	5.500371	-2.634043	-0.563551
H	4.893603	-3.034777	-1.389139
H	6.566802	-2.795728	-0.781841
H	5.241615	-3.153769	0.371021
Ru	0.940681	0.836393	-0.851359
C	0.758431	1.804700	-2.853696
C	1.280612	2.772290	-1.962013
C	-0.504741	1.142822	-2.596787
H	1.352665	1.496332	-3.719797
C	0.602212	3.094450	-0.729755
H	2.258086	3.219630	-2.161059
C	-1.181074	1.482151	-1.393191
C	-0.623314	2.424850	-0.463846
H	-2.159936	1.024141	-1.172310
H	-1.146506	2.570936	0.484952
C	1.152520	4.168866	0.190629
H	2.179807	4.397175	-0.148660
C	1.224569	3.746587	1.665468
H	1.981407	2.957694	1.806704
H	1.517210	4.609269	2.287758
H	0.254321	3.373661	2.038102
C	0.290342	5.438579	0.007983
H	0.719928	6.271669	0.589626
H	0.239507	5.747485	-1.050159
H	-0.740394	5.265727	0.363472
C	-1.019311	0.070691	-3.514499
H	-2.088714	-0.141366	-3.326900
H	-0.894962	0.377196	-4.567639
H	-0.430543	-0.853786	-3.366327
O	-5.750357	-0.216552	1.073032
O	-4.101321	1.056750	-1.305413
C	-4.765037	0.513643	-2.282257
C	-5.670350	0.950087	1.486989
O	-5.232246	1.980175	0.773001

O	-4.309538	-0.405243	-3.009309
C	-6.200738	1.009421	-2.473163
H	-6.797903	0.721426	-1.588631
H	-6.223230	2.110867	-2.532825
H	-6.652902	0.574841	-3.376695
C	-6.034118	1.339081	2.908267
H	-6.697658	0.582927	3.352176
H	-5.103310	1.388216	3.501984
H	-6.503145	2.334974	2.941408
Se	-0.072965	-0.870805	0.807838
Br	-1.856929	0.674419	2.118578
C	0.996809	-0.948519	2.440275
C	1.322787	-2.205081	2.972656
C	1.356339	0.229034	3.106220
C	2.045911	-2.271922	4.172309
H	1.043342	-3.116360	2.441072
C	2.078617	0.150234	4.303387
H	1.056366	1.196403	2.700624
C	2.430251	-1.098591	4.836088
H	2.308546	-3.250928	4.586029
H	2.368636	1.072096	4.817387
H	2.998571	-1.156919	5.769665
H	-4.841253	1.625522	-0.143248
O	1.058416	-2.509998	0.170374
C	0.347582	-3.238175	-0.685011
O	-0.815842	-2.986800	-1.034189
C	1.130460	-4.420524	-1.227108
H	1.779589	-4.046890	-2.039059
H	1.779513	-4.860190	-0.453413
H	0.437491	-5.174803	-1.627258

Statistical Thermodynamic Analysis
Temperature= 298.150 Kelvin Pressure= 1.00000 Atm

==

SCF Energy= -6947.90972047 Predicted Change= -1.794739D-08
Zero-point correction (ZPE)= -6947.2441 0.66554
Internal Energy (U)= -6947.1893 0.72032
Enthalpy (H)= -6947.1884 0.72127
Gibbs Free Energy (G)= -6947.3387 0.57096
Entropy (S)= 0.00050413

Frequencies -- 10.0784 12.9925 16.3346
Supporting Information: 010-Cs-Cluster-RuII-1HOAc-2OAc-PhSeBr-Cymene-02-PBE.log

----

Using Gaussian 16: ES64L-G16RevA.03 25-Dec-2016

==

# pbepbe/gen/auto pseudo=read gfprint gfinput empiricaldispersion=gd3bj
scf=(direct,vshift=200,tight,maxcycle=300,yqc) opt=(gdiis,maxcycle=250)
freq=noraman
#N Geom=AllCheck Guess=TCheck SCRF=Check GenChk RPBEPBE/Gen/Auto Freq
----
Pointgroup= C1 Stoichiometry= C26H42B10BrCsO8RuSe C1[X(C26H42B10BrCsO8RuSe)]
#Atoms= 90
Charge = 0 Multiplicity = 1

SCF Energy= -6947.90762627 Predicted Change= -1.019408D-07
==
Optimization completed. {Found 1 times}

Item	Max Val.	Criteria	Pass?	RMS Val.	Criteria	Pass?
Force	0.00002 ||	0.00045	[YES]	0.00000 ||	0.00030	[YES]
Displ	0.00516 ||	0.00180	[NO]	0.00516 ||	0.00180	[YES]

Atomic Type	Coordinates (Angstroms) X	Y	Z
B	4.433302	-0.736140	-1.872177
B	5.935723	-1.142441	-1.004287
H	7.023431	-0.874207	-1.424790
H	4.434976	-0.179141	-2.934063
B	5.652716	-2.641694	-0.088279
B	5.598037	-1.082482	0.741716
B	3.240755	-2.001178	-1.493814
B	3.166803	-0.427876	-0.650735
H	2.339918	-2.438285	-2.135082
H	2.398500	0.597920	-0.369721
H	6.341345	-0.836425	1.644369
H	6.440471	-3.475730	0.245017
C	2.989123	-1.870644	0.185859
C	4.404068	-2.277212	1.028229
B	4.936172	-2.448529	-1.699066
B	3.982625	-3.162800	-0.377795
H	3.573383	-4.271460	-0.230826
H	5.271477	-3.125509	-2.626867
B	4.839855	0.107310	-0.350329
H	5.108769	1.273858	-0.292492
B	3.906024	-0.638713	0.960759
H	3.474001	-0.141972	1.945224
C	1.558345	-2.208021	0.658853
O	0.631068	-1.560998	0.006760
O	1.346208	-3.067710	1.518675
Cs	-2.411726	-2.066336	1.198209
C	4.298659	-2.998395	2.361285

H	4.483330	-2.298468	3.189781
H	3.293241	-3.433505	2.465244
H	5.050228	-3.801538	2.394832
Ru	0.892561	0.228652	-1.139392
C	1.256888	-0.278973	-3.355258
C	1.645961	1.060613	-3.030286
C	-0.047994	-0.757826	-3.049797
H	2.007553	-0.964368	-3.754058
C	0.709353	2.029557	-2.539348
H	2.691339	1.351387	-3.175535
C	-0.959931	0.162306	-2.416720
C	-0.598935	1.534655	-2.247421
H	-1.948854	-0.210695	-2.095400
H	-1.327456	2.187074	-1.757431
C	1.123373	3.484626	-2.369763
H	2.033946	3.490345	-1.739293
C	0.058519	4.351958	-1.685674
H	-0.274385	3.931313	-0.724692
H	0.469381	5.356544	-1.492067
H	-0.830470	4.471324	-2.330512
C	1.495134	4.071849	-3.749541
H	1.834026	5.115429	-3.634941
H	2.303574	3.504207	-4.241335
H	0.618602	4.070699	-4.421272
C	-0.453950	-2.175180	-3.296973
H	-1.139105	-2.562302	-2.513758
H	-1.030488	-2.213268	-4.241354
H	0.419903	-2.837328	-3.405464
O	-5.241045	-0.926604	0.854019
O	-3.585606	-1.273353	-1.833987
C	-3.772686	-2.556461	-1.834423
C	-5.654309	0.063648	0.230754
O	-5.429945	0.297076	-1.056474
O	-2.861401	-3.400815	-1.623962
C	-5.214770	-3.030106	-2.037335
H	-5.799697	-2.763350	-1.137969
H	-5.678511	-2.510660	-2.892827
H	-5.255625	-4.118906	-2.188646
C	-6.448499	1.173234	0.894520
H	-7.205970	1.586782	0.210447
H	-6.917899	0.806111	1.818736
H	-5.746799	1.987917	1.150519
Se	-0.132357	1.038038	1.068184
Br	-2.538681	2.095242	0.387331
C	0.650368	2.779768	1.453359
C	-0.132701	3.730308	2.126506

C	1.990108	3.049687	1.135038
C	0.432622	4.967303	2.465458
H	-1.181416	3.514522	2.346839
C	2.545262	4.289081	1.479743
H	2.600180	2.301802	0.623712
C	1.766759	5.251415	2.139960
H	-0.179704	5.712541	2.982806
H	3.589179	4.500055	1.226186
H	2.200156	6.222318	2.400097
H	-4.732981	-0.408472	-1.422094
O	1.313885	0.280062	2.399800
C	0.716168	-0.478071	3.317681
O	-0.485769	-0.767214	3.341332
C	1.688851	-1.008820	4.362924
H	1.909761	-2.059949	4.111054
H	2.628444	-0.437531	4.389151
H	1.202497	-0.990696	5.350382

Statistical Thermodynamic Analysis
Temperature= 298.150 Kelvin Pressure= 1.00000 Atm

===

SCF Energy= -6947.90762627 Predicted Change= -1.019408D-07
Zero-point correction (ZPE)= -6947.2424 0.66519
Internal Energy (U)= -6947.1877 0.71983
Enthalpy (H)= -6947.1868 0.72078
Gibbs Free Energy (G)= -6947.3350 0.57255
Entropy (S)= 0.00049714

Frequencies -- 13.7543 20.9856 24.8857
Supporting Information: 010-Cs-Cluster-RuII-1HOAc-2OAc-PhSeBr-Cymene-03-PBE.log

----

Using Gaussian 16: ES64L-G16RevA.03 25-Dec-2016

===

# pbepbe/gen/auto pseudo=read gfprint gfinput empiricaldispersion=gd3bj
scf=(direct,vshift=200,tight,maxcycle=300,yqc) opt=(gdiis,maxcycle=250)
freq=noraman
#N Geom=AllCheck Guess=TCheck SCRF=Check GenChk RPBEPBE/Gen/Auto Freq

----

Pointgroup= C1 Stoichiometry= C26H42B10BrCsO8RuSe C1[X(C26H42B10BrCsO8RuSe)]
#Atoms= 90
Charge = 0 Multiplicity = 1

SCF Energy= -6947.88504099 Predicted Change= -9.041422D-09

===

Optimization completed. {Found 1 times}
Item Max Val. Criteria Pass? RMS Val. Criteria Pass?

Force 0.00001 || 0.00045 [YES] 0.00000 || 0.00030 [YES]
Displ 0.00278 || 0.00180 [NO] 0.00278 || 0.00180 [YES]

Atomic Type	Coordinates (Angstroms) X	Y	Z
B	-3.299352	3.200193	0.335294
B	-2.689297	4.639283	1.194483
H	-3.388042	5.330497	1.877398
H	-4.440903	2.834550	0.387386
B	-1.368122	5.338463	0.228088
B	-0.991700	4.334575	1.640300
B	-2.350748	3.033905	-1.159439
B	-1.967092	2.027905	0.256792
H	-2.664478	2.587142	-2.217714
H	-2.009067	0.796833	0.632339
H	-0.372357	4.782854	2.557736
H	-1.006074	6.475930	0.182804
C	-0.744190	2.777410	-0.632523
C	-0.151221	4.134732	0.158730
B	-2.787363	4.649564	-0.587511
B	-1.158335	4.344337	-1.219225
H	-0.641708	4.698271	-2.231434
H	-3.550123	5.327040	-1.213250
B	-2.179665	3.011141	1.718542
H	-2.507266	2.516227	2.757138
B	-0.547533	2.725045	1.072248
H	0.364883	2.095724	1.486977
C	0.185041	1.894359	-1.478949
O	-0.115771	0.622420	-1.479162
O	1.128076	2.384942	-2.104211
Cs	3.176970	-0.195943	-1.932606
C	1.322956	4.478219	0.045585
H	1.961286	3.600623	0.274256
H	1.559422	4.837402	-0.965779
H	1.535451	5.278248	0.772416
Ru	-1.922926	-0.256139	-0.772068
C	-3.002180	-0.024231	-2.751805
C	-3.934239	0.203898	-1.711683
C	-2.216169	-1.235460	-2.801510
H	-2.811193	0.762598	-3.487165
C	-4.130458	-0.753573	-0.648575
H	-4.453666	1.164733	-1.654872
C	-2.414438	-2.194555	-1.764122
C	-3.364284	-1.951840	-0.706586
H	-1.702481	-3.034791	-1.658305

H	-3.411241	-2.668267	0.117955
C	-5.146423	-0.489419	0.450439
H	-5.517195	0.542355	0.305351
C	-4.553164	-0.580616	1.866090
H	-3.826034	0.230325	2.036097
H	-5.355791	-0.480665	2.616499
H	-4.039823	-1.542148	2.039631
C	-6.335918	-1.457509	0.271070
H	-7.131123	-1.217057	0.996663
H	-6.764837	-1.391755	-0.743527
H	-6.021905	-2.502059	0.442983
C	-1.149793	-1.438532	-3.830985
H	-0.236944	-1.827042	-3.328652
H	-1.482561	-2.187727	-4.572775
H	-0.918845	-0.499268	-4.358439
O	4.004684	-1.982643	0.423280
O	-0.137704	-3.835873	-0.851086
C	0.972296	-3.540259	-1.416234
C	4.408790	-1.744443	1.574179
O	4.962983	-0.605754	1.959033
O	1.185483	-2.458383	-2.058350
C	2.147203	-4.498304	-1.224975
H	2.862869	-4.019844	-0.529492
H	1.817944	-5.461875	-0.807297
H	2.673578	-4.661892	-2.181175
C	4.241906	-2.738332	2.709411
H	4.111554	-3.753433	2.307590
H	5.095307	-2.699692	3.403958
H	3.334280	-2.468070	3.279632
Se	-0.241740	-1.544874	0.599088
Br	-1.463337	-3.150134	1.966122
C	0.198649	-0.436916	2.135283
C	1.555671	-0.092206	2.209521
C	-0.720381	0.001865	3.102051
C	2.002156	0.725472	3.259258
H	2.260517	-0.416397	1.442193
C	-0.263293	0.806211	4.150434
H	-1.771451	-0.292617	3.039336
C	1.094730	1.169931	4.228081
H	3.058891	1.001658	3.289202
H	-0.970561	1.159513	4.907684
H	1.439677	1.804612	5.050671
H	5.023292	0.098686	1.146597
O	3.159486	1.923669	0.523998
C	4.379877	2.109783	0.245416
O	5.282526	1.189742	0.206555

C	4.839817	3.528740	-0.117427
H	4.239199	3.917506	-0.959163
H	4.661454	4.201017	0.740290
H	5.907750	3.554056	-0.380034

Statistical Thermodynamic Analysis
Temperature= 298.150 Kelvin   Pressure= 1.00000 Atm

==

SCF Energy=   -6947.88504099   Predicted Change= -9.041422D-09
Zero-point correction (ZPE)=   -6947.2209   0.66410
Internal Energy (U)=   -6947.1661   0.71887
Enthalpy (H)=   -6947.1652   0.71982
Gibbs Free Energy (G)=   -6947.3133   0.57174
Entropy (S)=   0.00049666

Frequencies --   15.0019   18.2863   26.6024

Supporting Information: 020-Cs-Deprotonated-Cluster-RuII-2HOAc-1OAc-PhSeBr-01-PBE.log

----

Using Gaussian 16: ES64L-G16RevA.03 25-Dec-2016

==

# pbepbe/gen/auto pseudo=read gfprint gfinput empiricaldispersion=gd3bj
scf=(direct,vshift=200,tight,maxcycle=300,yqc) opt=(gdiis,maxcycle=250)
freq=noraman
#N Geom=AllCheck Guess=TCheck SCRF=Check GenChk RPBEPBE/Gen/Auto Freq

----

Pointgroup= C1   Stoichiometry= C16H28B10BrCsO8RuSe   C1[X(C16H28B10BrCsO8RuSe)]
#Atoms= 66
Charge = 0   Multiplicity = 1

SCF Energy= -6558.94070675   Predicted Change= -1.554570D-08

==

Optimization completed.   {Found   1   times}

Item	Max Val.	Criteria	Pass?	RMS Val.	Criteria	Pass?
Force	0.00001 ||	0.00045	[YES]	0.00000 ||	0.00030	[YES]
Displ	0.00640 ||	0.00180	[NO]	0.00640 ||	0.00180	[YES]

Atomic Type	Coordinates (Angstroms) X	Y	Z
B	-2.121145	3.016177	-0.813358
B	-1.824845	4.749717	-0.532526
H	-2.706349	5.551766	-0.409184
H	-3.227427	2.570413	-0.918718
B	-0.310810	5.193952	-1.357117
B	-0.315706	4.904009	0.394067
B	-0.779254	2.404639	-1.797510

B	-0.821310	2.060749	-0.030022
H	-0.779375	1.499634	-2.583414
H	-1.991682	0.556709	3.012867
H	0.011316	5.767258	1.155816
H	0.016172	6.260794	-1.787710
C	0.606023	2.630312	-0.854906
C	0.908183	4.267361	-0.609800
B	-1.408377	4.027102	-2.115465
B	0.344018	3.742625	-2.141027
H	1.120889	3.743009	-3.045051
H	-1.987070	4.277014	-3.133608
B	-1.435529	3.556090	0.745668
H	-2.023479	3.517844	1.790718
B	0.307568	3.274156	0.681744
H	1.107490	3.043159	1.533814
C	1.739056	1.600886	-0.868814
O	1.472791	0.515172	-0.216023
O	2.810147	1.844539	-1.464076
Cs	2.355350	-2.513846	0.536958
C	2.344591	4.754128	-0.512559
H	2.623413	4.914777	0.539886
H	3.025121	4.015910	-0.962584
H	2.437033	5.707113	-1.055459
Ru	-0.451070	0.140886	0.737420
O	0.302641	1.111097	2.771958
O	-2.154367	-0.677645	1.953000
C	-0.480589	1.560211	3.634928
C	-1.568040	-1.772448	2.386578
O	-1.764361	1.245921	3.730017
O	-0.342409	-1.983594	2.180324
C	-2.443631	-2.788981	3.078907
H	-1.857159	-3.376046	3.801669
H	-3.302660	-2.310028	3.572299
H	-2.829312	-3.479116	2.306448
C	-0.023411	2.574828	4.648826
H	-0.180125	3.577992	4.211487
H	-0.607379	2.508617	5.578346
H	1.050791	2.451807	4.846473
Se	-1.088200	-0.825347	-1.227687
Br	-0.870917	-3.489194	-1.024791
C	-3.014582	-0.896461	-1.411932
C	-3.532451	-1.601231	-2.513619
C	-3.858169	-0.181031	-0.548892
C	-4.911975	-1.592915	-2.745383
H	-2.859338	-2.163984	-3.167829
C	-5.237920	-0.174196	-0.795279

H	-3.433001	0.360716	0.297111
C	-5.764891	-0.878199	-1.887751
H	-5.322470	-2.142735	-3.598414
H	-5.900958	0.387820	-0.129725
H	-6.843565	-0.869416	-2.075111
O	4.802946	-1.196040	-0.605472
C	5.587640	-0.379685	-1.101468
O	5.248867	0.832295	-1.535907
C	7.066782	-0.653176	-1.292811
H	7.328763	-1.631818	-0.867720
H	7.313136	-0.638398	-2.368242
H	7.665949	0.139433	-0.814594
H	4.252166	1.009450	-1.418662

Statistical Thermodynamic Analysis
Temperature= 298.150 Kelvin   Pressure= 1.00000 Atm

===

SCF Energy=   -6558.94070675   Predicted Change= -1.554570D-08
Zero-point correction (ZPE)=   -6558.4794   0.46123
Internal Energy (U)=   -6558.4364   0.50427
Enthalpy (H)=   -6558.4354   0.50521
Gibbs Free Energy (G)=   -6558.5635   0.37714
Entropy (S)=   0.00042956

Frequencies --   8.3966   11.3215   20.9720
Supporting Information: 020-Cs-Deprotonated-Cluster-RuII-2HOAc-1OAc-PhSeBr-02-PBE.log

----

Using Gaussian 16: ES64L-G16RevA.03 25-Dec-2016

===

# pbepbe/gen/auto pseudo=read gfprint gfinput empiricaldispersion=gd3bj
scf=(direct,vshift=200,tight,maxcycle=300,yqc) opt=(gdiis,maxcycle=250)
freq=noraman
#N Geom=AllCheck Guess=TCheck SCRF=Check GenChk RPBEPBE/Gen/Auto Freq

----

Pointgroup= C1   Stoichiometry= C16H28B10BrCsO8RuSe   C1[X(C16H28B10BrCsO8RuSe)]
#Atoms= 66
Charge = 0   Multiplicity = 1

SCF Energy= -6558.92675234   Predicted Change= -2.585521D-07

===

Optimization completed.   {Found   1   times}

Item	Max Val.	Criteria	Pass?	RMS Val.	Criteria	Pass?
Force	0.00008 ||	0.00045	[YES]	0.00000 ||	0.00030	[YES]
Displ	0.00782 ||	0.00180	[NO]	0.00782 ||	0.00180	[YES]

Atomic   Coordinates (Angstroms)

Type	X	Y	Z
B	1.941861	2.628736	1.665209
B	1.945352	4.285037	1.016696
H	2.921475	4.980469	0.993778
H	2.919311	2.114161	2.134769
B	0.340008	4.997089	1.312397
B	0.747448	4.360915	-0.295300
B	0.334002	2.319792	2.347823
B	0.773200	1.639512	0.744742
H	0.021658	1.574708	3.228363
H	-0.284391	-2.325135	-0.667625
H	0.731948	5.086038	-1.247557
H	0.051861	6.151459	1.439575
C	-0.737019	2.476512	1.032621
C	-0.755747	4.029541	0.436008
B	1.061900	3.927903	2.533062
B	-0.656262	3.788782	2.142023
H	-1.633440	4.034197	2.781092
H	1.393209	4.341791	3.607483
B	1.741439	2.893692	-0.093783
H	2.556246	2.617238	-0.923795
B	0.028451	2.751052	-0.460702
H	-0.551110	2.400284	-1.443808
C	-1.961815	1.572150	0.931594
O	-1.669127	0.318373	0.894474
O	-3.129182	2.034570	0.864262
Cs	-3.571422	-1.970437	0.312834
C	-2.036558	4.623024	-0.130193
H	-2.343961	4.105240	-1.050370
H	-2.856632	4.554107	0.597093
H	-1.843985	5.681108	-0.365638
Ru	0.343961	-0.387331	0.633262
O	0.085081	-0.144164	-1.483318
O	0.318521	-1.040742	2.651805
C	-0.434415	-1.085125	-2.121158
C	-0.273666	-2.164227	2.461675
O	-0.684746	-2.291046	-1.594613
O	-0.684892	-2.516917	1.295273
C	-0.448581	-3.090996	3.644085
H	0.344585	-3.859223	3.613114
H	-0.364648	-2.537728	4.590893
H	-1.416553	-3.616243	3.591315
C	-0.874598	-0.929859	-3.545628
H	-0.578503	-1.806435	-4.143504
H	-1.977186	-0.851694	-3.560905

H	-0.442495	-0.010542	-3.964658
Se	2.562320	-1.009360	0.628948
Br	2.686572	-3.311318	-0.560064
C	3.582861	-0.061231	-0.720184
C	4.642379	0.725410	-0.243124
C	3.272481	-0.098928	-2.086872
C	5.375769	1.512534	-1.142797
H	4.875638	0.742943	0.826082
C	4.020132	0.677290	-2.979400
H	2.465319	-0.741840	-2.442222
C	5.064748	1.490255	-2.508820
H	6.190272	2.142587	-0.771659
H	3.785298	0.650576	-4.048770
H	5.639233	2.103216	-3.210793
O	-3.764848	0.012976	-2.001264
C	-3.910779	1.171705	-2.411369
O	-3.957412	2.250815	-1.628856
C	-4.026490	1.528471	-3.877742
H	-4.343678	0.650363	-4.457904
H	-4.725003	2.365696	-4.029054
H	-3.035979	1.855574	-4.242200
H	-3.739855	2.015424	-0.659598

Statistical Thermodynamic Analysis
Temperature= 298.150 Kelvin Pressure= 1.00000 Atm

==

SCF Energy= -6558.92675234 Predicted Change= -2.585521D-07
Zero-point correction (ZPE)= -6558.4680 0.45871
Internal Energy (U)= -6558.4253 0.50139
Enthalpy (H)= -6558.4244 0.50233
Gibbs Free Energy (G)= -6558.5483 0.37844
Entropy (S)= 0.00041553

Frequencies -- 12.3980 19.2920 25.6906

Supporting Information: 020-Cs-Deprotonated-Cluster-RuII-2HOAc-1OAc-PhSeBr-03-PBE.log

----

Using Gaussian 16: ES64L-G16RevA.03 25-Dec-2016

==

# pbepbe/gen/auto pseudo=read gfprint gfinput empiricaldispersion=gd3bj
scf=(direct,vshift=200,tight,maxcycle=300,yqc) opt=(gdiis,maxcycle=250)
freq=noraman
#N Geom=AllCheck Guess=TCheck SCRF=Check GenChk RPBEPBE/Gen/Auto Freq

----

Pointgroup= C1 Stoichiometry= C16H28B10BrCsO8RuSe C1[X(C16H28B10BrCsO8RuSe)]
#Atoms= 66
Charge = 0 Multiplicity = 1

SCF Energy= -6558.94375431 Predicted Change= -1.220192D-07
===

Optimization completed. {Found 1 times}

Item	Max Val.	Criteria	Pass?	RMS Val.	Criteria	Pass?
Force	0.00001 ||	0.00045	[YES]	0.00000 ||	0.00030	[YES]
Displ	0.02520 ||	0.00180	[NO]	0.02520 ||	0.00180	[NO]

Atomic Type	Coordinates (Angstroms) X	Y	Z
B	1.795558	3.443613	0.833531
B	2.060102	4.675643	-0.431557
H	3.005703	5.411377	-0.443250
H	2.543562	3.299958	1.760518
B	0.470266	5.237061	-0.999759
B	1.380236	4.049748	-1.951955
B	0.040558	3.258087	1.028552
B	0.976829	2.024156	0.106487
H	-0.585080	2.949695	1.994610
H	1.324353	1.009858	3.282344
H	1.713641	4.287816	-3.075819
H	0.179966	6.295019	-1.476159
C	-0.509437	2.702351	-0.475497
C	-0.290529	3.862624	-1.670435
B	0.707287	4.869105	0.719258
B	-0.781248	4.361729	-0.102593
H	-1.909708	4.722106	0.029108
H	0.660002	5.725669	1.554949
B	2.207505	2.940112	-0.828207
H	3.253831	2.458727	-1.153824
B	0.711152	2.444620	-1.619076
H	0.506314	1.642380	-2.481201
C	-1.672544	1.710163	-0.500154
O	-1.371818	0.530390	-0.058598
O	-2.798158	2.065749	-0.907678
Cs	-2.626726	-2.306551	0.072616
C	-1.297450	3.976223	-2.803389
H	-0.912464	3.482656	-3.708647
H	-2.250361	3.512032	-2.509313
H	-1.467127	5.041100	-3.024096
Ru	0.527380	0.082697	0.777670
O	2.355382	-0.430419	1.697477
O	-0.203957	0.785907	2.800449
C	2.873617	0.019927	2.747075
C	-0.724973	-0.357988	3.123985

O	2.292152	0.874260	3.574076
O	-0.677649	-1.323847	2.302896
C	-1.376531	-0.491966	4.480538
H	-1.787480	-1.501915	4.623121
H	-0.632842	-0.284491	5.269307
H	-2.176458	0.260301	4.586146
C	4.258173	-0.406931	3.147825
H	4.825990	0.459401	3.523073
H	4.197423	-1.145223	3.966858
H	4.768387	-0.863502	2.288232
Se	1.179811	-0.855364	-1.212315
C	3.100333	-1.179447	-1.282122
C	3.593287	-2.246411	-2.053572
C	3.985297	-0.265345	-0.685683
C	4.976060	-2.408266	-2.204395
H	2.898494	-2.958044	-2.507921
C	5.367341	-0.431094	-0.851689
H	3.594017	0.574167	-0.112309
C	5.866162	-1.504400	-1.603760
H	5.358213	-3.246879	-2.795596
H	6.053450	0.288867	-0.392907
H	6.946275	-1.633908	-1.727286
O	-5.154944	-0.730824	-0.346597
C	-5.855847	0.260793	-0.577137
O	-5.380514	1.481074	-0.824152
C	-7.370302	0.226510	-0.611843
H	-7.729790	-0.789834	-0.400772
H	-7.780670	0.931706	0.130420
H	-7.732393	0.554141	-1.600928
H	-4.365190	1.487825	-0.800041
Br	0.536871	-3.514434	-1.158461

Statistical Thermodynamic Analysis

Temperature= 298.150 Kelvin Pressure= 1.00000 Atm

===

SCF Energy= -6558.94375431 Predicted Change= -1.220192D-07

Zero-point correction (ZPE)= -6558.4844 0.45929

Internal Energy (U)= -6558.4413 0.50239

Enthalpy (H)= -6558.4404 0.50334

Gibbs Free Energy (G)= -6558.5685 0.37518

Entropy (S)= 0.00042984

Frequencies -- 6.1004 11.4633 20.7690

Supporting Information: 020-Cs-Deprotonated-Cluster-RuII-2HOAc-1OAc-PhSeBr-04-PBE.log

----

Using Gaussian 16: ES64L-G16RevA.03 25-Dec-2016

==

# pbepbe/gen/auto pseudo=read gfprint gfinput empiricaldispersion=gd3bj
scf=(direct,vshift=200,tight,maxcycle=300,yqc) opt=(gdiis,maxcycle=250)
freq=noraman
#N Geom=AllCheck Guess=TCheck SCRF=Check GenChk RPBEPBE/Gen/Auto Freq

----

Pointgroup= C1 Stoichiometry= C16H28B10BrCsO8RuSe C1[X(C16H28B10BrCsO8RuSe)]
#Atoms= 66
Charge = 0 Multiplicity = 1

SCF Energy= -6558.94364945 Predicted Change= -2.325057D-08

==

Optimization completed. {Found 1 times}

Item	Max Val.	Criteria	Pass?	RMS Val.	Criteria	Pass?
Force	0.00000 ||	0.00045	[YES]	0.00000 ||	0.00030	[YES]
Displ	0.00532 ||	0.00180	[NO]	0.00532 ||	0.00180	[YES]

Atomic Type	Coordinates (Angstroms) X	Y	Z
B	1.238269	3.647035	1.358433
B	1.334829	5.056569	0.266948
H	2.162564	5.915955	0.376723
H	1.987500	3.490740	2.280878
B	-0.312918	5.454758	-0.272957
B	0.771314	4.542904	-1.341180
B	-0.474361	3.186145	1.475560
B	0.640937	2.237607	0.431789
H	-1.055612	2.658721	2.372780
H	1.161150	-2.099042	2.141253
H	1.078873	4.971575	-2.414920
H	-0.746706	6.513920	-0.620422
C	-0.922724	2.764212	-0.107667
C	-0.858284	4.086439	-1.136573
B	-0.045428	4.903557	1.392284
B	-1.435140	4.301976	0.468452
H	-2.605559	4.478962	0.608486
H	-0.226570	5.630630	2.326539
B	1.738694	3.426254	-0.341757
H	2.846502	3.139960	-0.693693
B	0.339115	2.829581	-1.233928
H	0.264703	2.118188	-2.192911
C	-1.927388	1.628785	-0.301312
O	-1.463509	0.454067	-0.012422
O	-3.087363	1.870111	-0.696025
Cs	-2.233622	-2.526325	-0.370860

C	-1.857100	4.204709	-2.276411
H	-1.402711	3.868482	-3.220848
H	-2.748729	3.597311	-2.062367
H	-2.153666	5.259451	-2.381221
Ru	0.462320	0.194908	0.840920
O	2.314798	-0.024636	1.844097
O	-0.502622	0.369440	2.841281
C	2.794905	-1.093055	2.297311
C	-0.767244	-0.859910	2.998956
O	2.142417	-2.236731	2.391401
O	-0.429665	-1.725225	2.087175
C	-1.473692	-1.337955	4.246519
H	-0.819352	-2.036877	4.796021
H	-1.738198	-0.491025	4.895060
H	-2.386300	-1.895864	3.974151
C	4.230397	-1.134635	2.739246
H	4.427643	-2.004777	3.380699
H	4.866194	-1.195459	1.837240
H	4.483696	-0.199218	3.260571
Se	1.297734	-0.424649	-1.199053
C	3.242707	-0.390980	-1.281834
C	3.891417	-1.209427	-2.224293
C	3.972548	0.526850	-0.508306
C	5.280190	-1.121811	-2.373278
H	3.312771	-1.928236	-2.811538
C	5.363447	0.610784	-0.671470
H	3.457959	1.166438	0.208820
C	6.019022	-0.213923	-1.596891
H	5.787455	-1.766346	-3.098461
H	5.931178	1.330628	-0.072614
H	7.105078	-0.147318	-1.718320
O	-4.969811	-1.313027	-0.642639
C	-5.820218	-0.417024	-0.691909
O	-5.544782	0.886293	-0.709689
C	-7.311726	-0.680277	-0.739109
H	-7.504996	-1.761591	-0.725455
H	-7.806515	-0.199725	0.121700
H	-7.745277	-0.233338	-1.649551
H	-4.541856	1.048556	-0.682471
Br	1.234376	-3.164904	-1.204372

Statistical Thermodynamic Analysis
Temperature= 298.150 Kelvin Pressure= 1.00000 Atm

==

SCF Energy= -6558.94364945 Predicted Change= -2.325057D-08
Zero-point correction (ZPE)= -6558.4845 0.45907

Internal Energy (U)= -6558.4414 0.50219
Enthalpy (H)= -6558.4405 0.50313
Gibbs Free Energy (G)= -6558.5682 0.37537
Entropy (S)= 0.00042852

Frequencies -- 6.3646 14.5832 21.0485
Supporting Information: 020-Cs-Deprotonated-Cluster-RuII-2HOAc-1OAc-PhSeBr-05-PBE.log

---

Using Gaussian 16: ES64L-G16RevA.03 25-Dec-2016

==

# pbepbe/gen/auto pseudo=read gfprint gfinput empiricaldispersion=gd3bj
scf=(direct,vshift=200,tight,maxcycle=300,yqc) opt=(gdiis,maxcycle=250)
freq=noraman
#N Geom=AllCheck Guess=TCheck SCRF=Check GenChk RPBEPBE/Gen/Auto Freq

---

Pointgroup= C1 Stoichiometry= C16H28B10BrCsO8RuSe C1[X(C16H28B10BrCsO8RuSe)]
#Atoms= 66
Charge = 0 Multiplicity = 1

SCF Energy= -6558.94246362 Predicted Change= -1.304635D-07

==

Optimization completed. {Found 1 times}

Item	Max Val.	Criteria	Pass?	RMS Val.	Criteria	Pass?
Force	0.00008 ||	0.00045	[YES]	0.00000 ||	0.00030	[YES]
Displ	0.02018 ||	0.00180	[NO]	0.02018 ||	0.00180	[NO]

Atomic Type	Coordinates (Angstroms) X	Y	Z
B	2.206341	3.142405	1.043849
B	2.788754	4.283795	-0.200777
H	3.845507	4.844743	-0.134527
H	2.840816	2.897745	2.032578
B	1.374617	5.103732	-0.907856
B	2.138667	3.757059	-1.769727
B	0.438714	3.272983	1.084074
B	1.220374	1.863175	0.254412
H	-0.322874	3.114474	1.988106
H	2.598185	3.909130	-2.863660
H	1.314307	6.186764	-1.412002
C	-0.073434	2.787507	-0.446288
C	0.440601	3.869393	-1.629798
B	1.399348	4.735052	0.827367
B	-0.081189	4.479434	-0.116460
H	-1.136313	5.033259	-0.081421
H	1.434792	5.602352	1.653603

B	2.663685	2.539752	-0.573071
H	3.629435	1.869517	-0.797902
B	1.171951	2.303612	-1.483729
H	0.903259	1.540362	-2.365457
C	-1.385806	2.006298	-0.516845
O	-1.330270	0.845884	0.054079
O	-2.396562	2.504707	-1.055908
Cs	-2.784771	-1.873825	0.589362
C	-0.439773	4.124379	-2.841992
H	-0.045203	3.590889	-3.720046
H	-1.466798	3.786442	-2.637543
H	-0.451332	5.203827	-3.056970
Ru	0.462426	-0.002159	0.853387
O	2.000200	-1.168413	1.827350
O	-0.045372	0.874596	2.810433
C	1.219648	-2.150143	2.211581
C	0.774323	1.110636	3.726607
O	-0.032499	-2.047915	2.073349
O	2.002388	0.619985	3.780794
C	0.428544	2.047133	4.848975
H	0.989129	1.806216	5.763757
H	0.705978	3.067852	4.527091
H	-0.655286	2.032082	5.031061
C	1.865994	-3.404922	2.736136
H	2.865374	-3.200391	3.147906
H	1.224043	-3.885338	3.490172
H	1.972858	-4.101216	1.883909
Se	0.800505	-0.915449	-1.220690
C	2.652759	-1.357449	-1.568643
C	2.937540	-2.030910	-2.770553
C	3.684096	-0.934874	-0.716484
C	4.268704	-2.287858	-3.115414
H	2.118886	-2.364640	-3.415787
C	5.014592	-1.191872	-1.075216
H	3.440446	-0.412514	0.209153
C	5.307945	-1.866880	-2.269007
H	4.496378	-2.815163	-4.047439
H	5.823780	-0.858263	-0.417548
H	6.349098	-2.064711	-2.543960
O	-4.956794	-0.157869	-0.584132
C	-5.518218	0.773136	-1.172652
O	-4.929235	1.915279	-1.520894
C	-6.976550	0.741907	-1.586084
H	-7.435778	-0.208446	-1.281356
H	-7.517871	1.585084	-1.124929
H	-7.060947	0.864829	-2.679058

H -3.950352 1.931875 -1.239393
Br 0.075633 -3.502201 -1.146168
H 2.134860 -0.006997 2.990133

Statistical Thermodynamic Analysis
Temperature= 298.150 Kelvin Pressure= 1.00000 Atm

==

SCF Energy= -6558.94246362 Predicted Change= -1.304635D-07
Zero-point correction (ZPE)= -6558.4831 0.45928
Internal Energy (U)= -6558.4402 0.50222
Enthalpy (H)= -6558.4392 0.50317
Gibbs Free Energy (G)= -6558.5660 0.37639
Entropy (S)= 0.0004252

Frequencies -- 7.5698 12.1513 23.2328
Supporting Information: 030-Selenylated-Cluster-RuII-2HOAc-1OAc-CsBr-01-PBE.log

----

Using Gaussian 16: ES64L-G16RevA.03 25-Dec-2016

==

# pbepbe/gen/auto pseudo=read gfprint gfinput empiricaldispersion=gd3bj
scf=(direct,vshift=200,tight,maxcycle=300,yqc) opt=(gdiis,maxcycle=250)
freq=noraman
#N Geom=AllCheck Guess=TCheck SCRF=Check GenChk RPBEPBE/Gen/Auto Freq

----

Pointgroup= C1 Stoichiometry= C16H28B10BrCsO8RuSe C1[X(C16H28B10BrCsO8RuSe)]
#Atoms= 66
Charge = 0 Multiplicity = 1

SCF Energy= -6558.96441949 Predicted Change= -3.985356D-08

==

Optimization completed. {Found 1 times}

Item	Max Val.		Criteria	Pass?	RMS Val.		Criteria	Pass?
Force	0.00000	||	0.00045	[YES]	0.00000	||	0.00030	[YES]
Displ	0.00820	||	0.00180	[NO]	0.00820	||	0.00180	[YES]

Atomic Type	Coordinates (Angstroms) X	Y	Z
B	-3.698671	1.796026	-1.721184
B	-3.423230	3.441627	-1.080299
H	-4.316886	4.181165	-0.785070
H	-4.787253	1.322623	-1.878723
B	-1.930756	4.064086	-1.813676
B	-1.896356	3.423605	-0.151214
B	-2.362757	1.414471	-2.838756
B	-2.350188	0.805611	-1.170663

H	-2.346855	0.651042	-3.756730
H	-1.579125	4.133219	0.757914
H	-1.623994	5.201372	-2.017333
C	-0.946599	1.500939	-1.871155
C	-0.688925	3.019593	-1.280781
B	-3.030434	3.066273	-2.788881
B	-1.281450	2.824369	-2.899996
H	-0.513567	3.035070	-3.783616
H	-3.622914	3.531137	-3.718819
B	-3.002608	2.037861	-0.080473
H	-3.569929	1.729992	0.925124
B	-1.256505	1.750136	-0.179294
H	-0.388468	1.657572	0.770835
C	0.316662	0.664579	-2.193690
O	0.906346	0.810383	-3.261945
O	0.675713	-0.159815	-1.234333
C	0.747104	3.493663	-1.128514
H	0.775749	4.584924	-1.269227
H	1.135555	3.257224	-0.125448
H	1.390968	3.028366	-1.891545
Ru	-0.171375	-0.062234	0.711390
Se	-2.091557	-1.083130	-0.513643
C	-3.623947	-1.157545	0.678473
C	-4.911712	-1.206613	0.124553
C	-3.419302	-1.233823	2.061655
C	-6.018079	-1.304176	0.979253
H	-5.051948	-1.152463	-0.959319
C	-4.535955	-1.340183	2.904365
H	-2.406767	-1.189765	2.470491
C	-5.831358	-1.371447	2.368114
H	-7.027322	-1.327646	0.555693
H	-4.387056	-1.394178	3.987795
H	-6.697902	-1.448720	3.032607
O	1.470390	0.611401	2.077496
C	0.656022	0.469617	3.087789
C	1.138790	0.716997	4.489293
H	1.771160	-0.130210	4.808596
H	1.754900	1.630804	4.525645
H	0.287049	0.805233	5.178703
O	-0.537845	0.111426	2.839097
Cs	3.562730	-0.845627	-0.131899
Br	0.549068	-2.455279	1.163047
H	0.702735	-1.799060	-1.962786
O	0.507097	-2.634063	-2.481543
C	1.649098	-3.324455	-2.630526
C	1.419709	-4.640946	-3.338474

H	0.879097	-5.325554	-2.661666
H	0.789044	-4.497709	-4.230574
H	2.382978	-5.089377	-3.616979
O	2.746249	-2.937898	-2.223446
O	4.304120	2.014433	0.795065
C	3.764528	3.026473	1.244648
O	2.595564	3.016720	1.911747
C	4.315937	4.426073	1.089003
H	4.276165	4.969384	2.046706
H	5.347172	4.383582	0.713318
H	3.691418	4.987202	0.371356
H	2.253740	2.060254	1.964611

Statistical Thermodynamic Analysis
Temperature= 298.150 Kelvin Pressure= 1.00000 Atm
===
SCF Energy= -6558.96441949 Predicted Change= -3.985356D-08
Zero-point correction (ZPE)= -6558.5058 0.45861
Internal Energy (U)= -6558.4625 0.50188
Enthalpy (H)= -6558.4615 0.50283
Gibbs Free Energy (G)= -6558.5880 0.37633
Entropy (S)= 0.00042426

Frequencies -- 14.6892 19.9020 22.3754
Supporting Information: 030-Selenylated-Cluster-RuII-2HOAc-1OAc-CsBr-02-PBE.log
----
Using Gaussian 16: ES64L-G16RevA.03 25-Dec-2016
===
# pbepbe/gen/auto pseudo=read gfprint gfinput empiricaldispersion=gd3bj
scf=(direct,vshift=200,tight,maxcycle=300,yqc) opt=(gdiis,maxcycle=250)
freq=noraman
#N Geom=AllCheck Guess=TCheck SCRF=Check GenChk RPBEPBE/Gen/Auto Freq
----
Pointgroup= C1 Stoichiometry= C16H28B10BrCsO8RuSe C1[X(C16H28B10BrCsO8RuSe)]
#Atoms= 66
Charge = 0 Multiplicity = 1

SCF Energy= -6558.96961506 Predicted Change= -1.084318D-07
===
Optimization completed. {Found 1 times}

Item	Max Val.	Criteria	Pass?	RMS Val.	Criteria	Pass?
Force	0.00001 ||	0.00045	[YES]	0.00000 ||	0.00030	[YES]
Displ	0.01238 ||	0.00180	[NO]	0.01238 ||	0.00180	[NO]

Atomic Coordinates (Angstroms)
Type X Y Z

B	-3.567129	2.883994	-0.150831
B	-3.795515	3.170460	1.600746
H	-4.850696	3.475178	2.076819
H	-4.453679	2.958240	-0.952565
B	-2.277664	3.838184	2.242576
B	-2.658288	2.115317	2.486033
B	-1.906301	3.375973	-0.569112
B	-2.300370	1.667109	-0.298082
H	-1.471248	3.732230	-1.622503
H	-2.771115	1.680479	3.594911
H	-2.126230	4.550991	3.190348
C	-0.900444	2.366590	0.385236
C	-1.106925	2.616948	1.997172
B	-2.832050	4.322135	0.622914
B	-1.122422	3.975116	0.907319
H	-0.165646	4.682303	0.915477
H	-3.178577	5.446073	0.401820
B	-3.471442	1.525515	1.022935
H	-4.262999	0.630964	1.053387
B	-1.763875	1.133670	1.282384
H	-1.293041	0.125601	1.970156
C	0.527976	1.959294	-0.057939
O	1.339260	2.828844	-0.394514
O	0.758455	0.680992	0.001485
C	0.112606	2.463989	2.891548
H	-0.083258	2.959741	3.854078
H	0.322849	1.397154	3.075403
H	0.994338	2.932463	2.426581
Ru	-0.687382	-0.724876	0.619172
Se	-1.895698	0.058912	-1.444941
C	-3.686296	-0.696988	-1.454267
C	-4.665885	-0.084499	-2.249840
C	-3.958753	-1.861299	-0.725812
C	-5.952326	-0.638693	-2.295417
H	-4.432753	0.823636	-2.814513
C	-5.248726	-2.409943	-0.788252
H	-3.183979	-2.314325	-0.101995
C	-6.243917	-1.801605	-1.566720
H	-6.726468	-0.158562	-2.902652
H	-5.473965	-3.316627	-0.217167
H	-7.249432	-2.232751	-1.605701
O	0.284113	-1.741431	2.324317
C	-0.717291	-2.557390	2.389477
C	-0.700033	-3.721602	3.343715
H	-0.239465	-4.591522	2.842062

H	-0.105587	-3.480983	4.238597
H	-1.726769	-3.996122	3.628950
O	-1.708735	-2.368269	1.602780
Cs	3.124684	-1.059530	1.030585
Br	0.377730	-2.488507	-0.932383
H	1.129611	-0.899034	-2.329195
O	1.276482	-0.066958	-2.886739
C	2.497277	0.409536	-2.672956
C	2.709691	1.781966	-3.251068
H	2.092097	1.936175	-4.147916
H	2.385340	2.501250	-2.474811
H	3.772884	1.955248	-3.471450
O	3.357438	-0.210741	-2.015641
O	5.708777	0.433847	0.316431
C	6.242298	0.997691	-0.643723
O	5.736109	1.009055	-1.883417
C	7.546387	1.761407	-0.554955
H	7.370833	2.825776	-0.787298
H	7.968355	1.668365	0.454860
H	8.262795	1.381551	-1.302204
H	4.842454	0.517427	-1.887018

Statistical Thermodynamic Analysis
Temperature= 298.150 Kelvin Pressure= 1.00000 Atm

==

SCF Energy= -6558.96961506 Predicted Change= -1.084318D-07
Zero-point correction (ZPE)= -6558.5109 0.45863
Internal Energy (U)= -6558.4680 0.50152
Enthalpy (H)= -6558.4671 0.50246
Gibbs Free Energy (G)= -6558.5914 0.37816
Entropy (S)= 0.00041689

Frequencies -- 11.9502 23.8183 29.1424
Supporting Information: 030-Selenylated-Cluster-RuII-2HOAc-1OAc-CsBr-03-PBE.log

----

Using Gaussian 16: ES64L-G16RevA.03 25-Dec-2016

==

# pbepbe/gen/auto pseudo=read gfprint gfinput empiricaldispersion=gd3bj
scf=(direct,vshift=200,tight,maxcycle=300,yqc) opt=(gdiis,maxcycle=250)
freq=noraman
#N Geom=AllCheck Guess=TCheck SCRF=Check GenChk RPBEPBE/Gen/Auto Freq

----

Pointgroup= C1 Stoichiometry= C16H28B10BrCsO8RuSe C1[X(C16H28B10BrCsO8RuSe)]
#Atoms= 66
Charge = 0 Multiplicity = 1

SCF Energy= -6558.95624577 Predicted Change= -1.120939D-07

===

Optimization completed. {Found 1 times}

Item	Max Val.	Criteria	Pass?	RMS Val.	Criteria	Pass?
Force	0.00007 ||	0.00045	[YES]	0.00000 ||	0.00030	[YES]
Displ	0.00768 ||	0.00180	[NO]	0.00768 ||	0.00180	[YES]

Atomic Type	Coordinates (Angstroms) X	Y	Z
B	-4.298532	1.843911	0.120517
B	-4.146308	2.362258	1.825930
H	-5.078014	2.410609	2.576009
H	-5.331943	1.493465	-0.373023
B	-2.843964	3.571225	1.909872
B	-2.523809	1.889339	2.403429
B	-3.083241	2.736918	-0.829609
B	-2.774048	1.069952	-0.306875
H	-3.091947	3.009465	-1.992128
H	-2.183203	1.653961	3.525969
H	-2.709243	4.457288	2.701237
C	-1.575754	2.278466	-0.148449
C	-1.430569	2.748388	1.420218
B	-3.935110	3.554747	0.504828
B	-2.201933	3.809142	0.276366
H	-1.585799	4.766403	-0.068978
H	-4.696448	4.456577	0.306375
B	-3.432855	0.819397	1.318172
H	-3.825346	-0.254579	1.665180
B	-1.692071	1.028597	1.070701
H	-0.711924	0.387944	1.643684
C	-0.257812	2.253692	-0.967591
O	0.101428	3.278038	-1.561244
O	0.384151	1.126721	-0.921519
C	-0.047487	3.144637	1.909439
H	-0.139184	3.575797	2.917938
H	0.609810	2.259948	1.960155
H	0.403637	3.895361	1.242587
Ru	-0.261607	-0.509715	0.261305
Se	-2.159369	-0.513104	-1.391810
C	-3.510898	-1.774133	-0.792599
C	-4.818838	-1.643593	-1.282230
C	-3.151860	-2.822359	0.063631
C	-5.791924	-2.570451	-0.884330
H	-5.079534	-0.819349	-1.953403
C	-4.134542	-3.747796	0.446520

H	-2.129531	-2.897047	0.443267
C	-5.450456	-3.623006	-0.021831
H	-6.818442	-2.467246	-1.250675
H	-3.865369	-4.567269	1.120997
H	-6.212757	-4.346008	0.285901
O	1.404939	-0.841650	1.667040
C	0.777818	-1.862628	2.153166
C	1.403170	-2.724180	3.216713
H	1.835504	-3.616693	2.731575
H	2.201581	-2.175723	3.739199
H	0.636546	-3.059185	3.932544
O	-0.383800	-2.136159	1.687266
Cs	3.376359	0.332995	-0.642910
Br	0.833171	-2.157863	-1.386551
H	2.967387	4.696048	1.724822
O	2.715725	5.158931	0.890996
C	3.188591	4.368670	-0.111485
C	2.839305	4.893669	-1.473178
H	1.896744	4.398351	-1.781695
H	2.680503	5.981581	-1.463489
H	3.630670	4.623000	-2.187994
O	3.791061	3.322624	0.126331
O	4.437135	-2.472528	0.063366
C	4.040327	-3.527676	0.558095
O	2.791233	-4.007145	0.425600
C	4.896519	-4.437716	1.413812
H	4.784955	-5.487220	1.097524
H	5.948710	-4.128333	1.351428
H	4.562106	-4.376225	2.464701
H	2.239906	-3.369437	-0.135003

Statistical Thermodynamic Analysis
Temperature= 298.150 Kelvin   Pressure= 1.00000 Atm

==

SCF Energy=   -6558.95624577   Predicted Change= -1.120939D-07
Zero-point correction (ZPE)=   -6558.4979   0.45832
Internal Energy (U)=   -6558.4545   0.50166
Enthalpy (H)=   -6558.4536   0.50260
Gibbs Free Energy (G)=   -6558.5801   0.37609
Entropy (S)=   0.00042433

Frequencies --   16.2585   20.4378   22.5866
Supporting Information: 030-Selenylated-Cluster-RuII-2HOAc-1OAc-CsBr-04-PBE.log

----

Using Gaussian 16: ES64L-G16RevA.03 25-Dec-2016

==

# pbepbe/gen/auto pseudo=read gfprint gfinput empiricaldispersion=gd3bj
scf=(direct,vshift=200,tight,maxcycle=300,yqc) opt=(gdiis,maxcycle=250)
freq=noraman
#N Geom=AllCheck Guess=TCheck SCRF=Check GenChk RPBEPBE/Gen/Auto Freq

----

Pointgroup= C1 Stoichiometry= C16H28B10BrCsO8RuSe C1[X(C16H28B10BrCsO8RuSe)]
#Atoms= 66
Charge = 0 Multiplicity = 1

SCF Energy= -6558.95173261 Predicted Change= -1.268673D-08

==

Optimization completed. {Found 1 times}

Item	Max Val.	Criteria	Pass?	RMS Val.	Criteria	Pass?
Force	0.00000 ||	0.00045	[YES]	0.00000 ||	0.00030	[YES]
Displ	0.00246 ||	0.00180	[NO]	0.00246 ||	0.00180	[YES]

Atomic Type	Coordinates (Angstroms) X	Y	Z
B	-2.743194	3.272489	0.351234
B	-1.906260	3.542978	1.909751
H	-2.437391	4.056549	2.851590
H	-3.888519	3.561825	0.156397
B	-0.191219	3.842275	1.555912
B	-0.707054	2.232907	2.124810
B	-1.529976	3.401276	-0.949625
B	-2.040473	1.814258	-0.342807
H	-1.687791	3.691563	-2.097612
H	1.653547	-2.626312	1.190985
H	-0.271567	1.798666	3.150740
H	0.595908	4.473964	2.197237
C	-0.388351	2.165801	-0.606184
C	0.380639	2.407469	0.830236
B	-1.434895	4.488246	0.461202
B	0.037007	3.769463	-0.197676
H	0.957998	4.252248	-0.775624
H	-1.611239	5.667744	0.359481
B	-2.288817	1.889605	1.405891
H	-3.090555	1.186068	1.942342
B	-0.840571	1.119065	0.724505
H	-0.332494	-0.032800	1.156563
C	0.454254	1.467803	-1.701830
O	0.262473	0.187857	-1.797278
O	1.281479	2.120646	-2.355481
Cs	3.267415	-0.467939	-1.828098
C	1.830975	1.978238	0.947158

H	1.909896	0.880635	1.009486
H	2.416453	2.342116	0.088588
H	2.261404	2.401872	1.866753
Ru	-0.709325	-0.899073	-0.244274
O	3.353876	-1.009740	1.381836
O	1.070629	-2.293905	-0.269882
C	2.959635	-1.760255	2.304333
C	0.362545	-3.076377	-1.041340
O	2.052786	-2.700173	2.131920
O	-0.718454	-2.622604	-1.527459
C	0.804666	-4.489743	-1.300577
H	0.369663	-4.864178	-2.239099
H	1.903808	-4.569130	-1.322188
H	0.434590	-5.118870	-0.471013
C	3.450907	-1.647025	3.726226
H	2.731668	-1.029505	4.293954
H	3.486471	-2.635913	4.207117
H	4.435389	-1.158791	3.762532
Se	-2.712407	0.165343	-1.291389
C	-4.430132	0.058722	-0.379555
C	-5.427351	0.955934	-0.793249
C	-4.681165	-0.917791	0.590993
C	-6.696522	0.884935	-0.203173
H	-5.213663	1.709990	-1.557516
C	-5.958847	-0.979776	1.167950
H	-3.880879	-1.593709	0.909982
C	-6.963310	-0.083345	0.776472
H	-7.475838	1.589443	-0.511487
H	-6.161147	-1.733797	1.935661
H	-7.955832	-0.136326	1.235626
Br	-1.566926	-2.446498	1.595016
O	5.225477	1.229685	-0.185418
C	5.431127	1.638173	0.960620
O	4.912608	1.066056	2.059524
C	6.267932	2.854101	1.289141
H	6.958900	2.639230	2.120027
H	6.824586	3.177734	0.399285
H	5.606179	3.673935	1.619805
H	4.345410	0.270845	1.774037

Statistical Thermodynamic Analysis
Temperature= 298.150 Kelvin Pressure= 1.00000 Atm

==

SCF Energy= -6558.95173261 Predicted Change= -1.268673D-08
Zero-point correction (ZPE)= -6558.4935 0.45822
Internal Energy (U)= -6558.4505 0.50119

Enthalpy (H)= -6558.4495 0.50213
Gibbs Free Energy (G)= -6558.5745 0.37720
Entropy (S)= 0.00041901

Frequencies -- 19.0574 21.7764 23.8108

Supporting Information: 030-Selenylated-Cluster-RuII-2HOAc-1OAc-CsBr-05-PBE.log

----

Using Gaussian 16: ES64L-G16RevA.03 25-Dec-2016

==

# pbepbe/gen/auto pseudo=read gfprint gfinput empiricaldispersion=gd3bj
scf=(direct,vshift=200,tight,maxcycle=300,yqc) opt=(gdiis,maxcycle=250)
freq=noraman
#N Geom=AllCheck Guess=TCheck SCRF=Check GenChk RPBEPBE/Gen/Auto Freq

----

Pointgroup= C1 Stoichiometry= C16H28B10BrCsO8RuSe C1[X(C16H28B10BrCsO8RuSe)]
#Atoms= 66
Charge = 0 Multiplicity = 1

SCF Energy= -6558.96475785 Predicted Change= -2.169064D-07

==

Optimization completed. {Found 1 times}

Item	Max Val.	Criteria	Pass?	RMS Val.	Criteria	Pass?
Force	0.00000 ||	0.00045	[YES]	0.00000 ||	0.00030	[YES]
Displ	0.04514 ||	0.00180	[NO]	0.04514 ||	0.00180	[NO]

Atomic Type	Coordinates (Angstroms) X	Y	Z
B	-3.791929	2.809227	-0.700069
B	-3.414879	3.757988	0.766534
H	-4.254167	4.318088	1.410729
H	-4.904093	2.662552	-1.119556
B	-1.806142	4.476944	0.544055
B	-1.989930	3.018717	1.551287
B	-2.403443	2.943083	-1.810613
B	-2.603614	1.511478	-0.780957
H	-2.382604	2.830795	-2.999261
H	1.833336	0.048266	2.513131
H	-1.689020	3.050174	2.708793
H	-1.370728	5.479799	1.028026
C	-1.081517	2.281657	-0.938716
C	-0.731360	3.156656	0.413887
B	-2.905183	4.355326	-0.845929
B	-1.186955	3.983717	-1.041758
H	-0.324097	4.549572	-1.633688
H	-3.361924	5.335738	-1.358189

B	-3.230239	1.993143	0.802586
H	-3.915966	1.252887	1.442943
B	-1.520042	1.577479	0.595184
H	-0.745439	0.868245	1.357225
C	0.134399	1.600529	-1.626667
O	0.352465	0.377147	-1.237003
O	0.822117	2.233939	-2.431590
Cs	2.891083	-1.285785	-1.189284
C	0.725958	3.267029	0.831827
H	1.007953	2.434732	1.495702
H	1.379950	3.266160	-0.054686
H	0.868547	4.215245	1.372172
Ru	-0.694542	-0.529770	0.348466
O	3.921050	0.296826	1.417537
O	0.848047	-0.990692	1.891674
C	3.519730	1.009735	2.357423
C	-0.073169	-1.554514	2.629665
O	2.349990	0.827886	2.954213
O	-1.265269	-1.540627	2.196673
C	0.299769	-2.207942	3.930968
H	-0.599001	-2.399686	4.534183
H	0.801939	-3.168785	3.720666
H	1.006777	-1.574571	4.492213
C	4.289623	2.187861	2.905495
H	3.849208	3.116865	2.500656
H	4.198887	2.233744	4.001553
H	5.346147	2.141344	2.606544
Se	-2.555752	-0.433357	-1.308600
C	-4.162590	-1.035058	-0.398209
C	-5.411221	-0.505314	-0.755129
C	-4.047583	-2.057251	0.553115
C	-6.563392	-0.988593	-0.119990
H	-5.484540	0.288166	-1.504505
C	-5.210229	-2.537173	1.173736
H	-3.063209	-2.458524	0.809421
C	-6.464345	-2.002997	0.844014
H	-7.539909	-0.568065	-0.381111
H	-5.129226	-3.331474	1.922965
H	-7.366802	-2.377629	1.337728
Br	-0.302193	-2.869200	-0.598769
O	5.868303	-0.418881	-1.248155
C	6.678939	0.283132	-0.640816
O	6.423659	0.852323	0.550018
C	8.068706	0.607848	-1.141233
H	8.822427	0.290095	-0.401363
H	8.248032	0.103997	-2.100399

H	8.177904	1.698553	-1.266171
H	5.480747	0.605437	0.826963

Statistical Thermodynamic Analysis
Temperature= 298.150 Kelvin Pressure= 1.00000 Atm

==

SCF Energy= -6558.96475785 Predicted Change= -2.169064D-07
Zero-point correction (ZPE)= -6558.5064 0.45827
Internal Energy (U)= -6558.4632 0.50152
Enthalpy (H)= -6558.4622 0.50247
Gibbs Free Energy (G)= -6558.5913 0.37342
Entropy (S)= 0.00043284

Frequencies -- 6.3990 12.7251 16.0810
Supporting Information: 030-Selenylated-Cluster-RuII-2HOAc-1OAc-CsBr-06-PBE.log

----

Using Gaussian 16: ES64L-G16RevA.03 25-Dec-2016

==

# pbepbe/gen/auto pseudo=read gfprint gfinput empiricaldispersion=gd3bj
scf=(direct,vshift=200,tight,maxcycle=300,yqc) opt=(gdiis,maxcycle=250)
freq=noraman
#N Geom=AllCheck Guess=TCheck SCRF=Check GenChk RPBEPBE/Gen/Auto Freq

----

Pointgroup= C1 Stoichiometry= C16H28B10BrCsO8RuSe C1[X(C16H28B10BrCsO8RuSe)]
#Atoms= 66
Charge = 0 Multiplicity = 1

SCF Energy= -6558.96649355 Predicted Change= -8.901533D-08

==

Optimization completed on the basis of negligible forces. {Found 1 times}

Item	Max Val.	Criteria	Pass?	RMS Val.	Criteria	Pass?
Force	0.00000 ||	0.00045	[YES]	0.00000 ||	0.00030	[YES]
Displ	0.04171 ||	0.00180	[NO]	0.04171 ||	0.00180	[NO]

Atomic Type	Coordinates (Angstroms) X	Y	Z
B	-2.819266	3.404863	-0.699083
B	-2.366042	4.185563	0.843063
H	-3.138208	4.828428	1.494048
H	-3.919170	3.477871	-1.167839
B	-0.654420	4.652073	0.727247
B	-1.111767	3.183006	1.624008
B	-1.383185	3.387963	-1.749647
B	-1.842701	1.939193	-0.825637
H	-1.326543	3.348421	-2.941667

H	-1.266032	-3.284665	1.165366
H	-0.864691	3.093669	2.790531
H	-0.091947	5.541921	1.293470
C	-0.218830	2.476576	-0.883016
C	0.202888	3.185955	0.542654
B	-1.698876	4.797180	-0.704354
B	-0.055429	4.172377	-0.869232
H	0.910563	4.631261	-1.390348
H	-1.977716	5.867752	-1.160803
B	-2.459464	2.415434	0.761951
H	-3.280308	1.755096	1.325560
B	-0.824556	1.748032	0.590698
H	-0.262435	0.885203	1.409149
C	0.907436	1.672024	-1.582785
O	1.018209	0.443079	-1.168509
O	1.625356	2.213095	-2.427264
Cs	3.287659	-1.479329	-1.419736
C	1.635585	3.011686	1.015127
H	1.785294	2.004760	1.433044
H	2.339793	3.161074	0.181564
H	1.849418	3.755165	1.797327
Ru	-0.257759	-0.380281	0.276173
O	0.210375	-2.092711	-0.916702
O	-1.464277	-3.229930	2.199756
C	-0.394763	-3.222758	-1.026926
C	-1.750574	-2.005520	2.560102
O	-1.278000	-3.692767	-0.255622
O	-1.578970	-0.974073	1.864931
C	-2.322287	-1.862130	3.948252
H	-2.889381	-0.923554	4.023574
H	-2.955261	-2.725129	4.204124
H	-1.486201	-1.822430	4.669376
C	0.026021	-4.082884	-2.216464
H	0.651833	-4.921174	-1.861049
H	-0.869089	-4.525185	-2.681762
H	0.579560	-3.500580	-2.969847
Se	-2.057186	0.032780	-1.464127
C	-3.793645	-0.422571	-0.719354
C	-4.879911	0.459712	-0.806069
C	-3.946825	-1.705525	-0.172936
C	-6.126695	0.062737	-0.302235
H	-4.757409	1.452848	-1.244532
C	-5.200532	-2.091710	0.321155
H	-3.099217	-2.396468	-0.133406
C	-6.289140	-1.209052	0.265372
H	-6.972307	0.756117	-0.355680

H	-5.319906	-3.090467	0.754149
H	-7.264379	-1.513293	0.658816
Br	1.729294	-0.985868	1.861674
O	5.113743	0.282438	0.270316
C	5.104072	1.107061	1.185846
O	4.168167	1.155458	2.148545
H	3.474723	0.440586	1.977876
C	6.133480	2.203460	1.351798
H	6.484150	2.252856	2.395158
H	6.977654	2.030737	0.670625
H	5.670470	3.178002	1.116955

Statistical Thermodynamic Analysis
Temperature= 298.150 Kelvin Pressure= 1.00000 Atm
==
SCF Energy= -6558.96649355 Predicted Change= -8.901533D-08
Zero-point correction (ZPE)= -6558.5086 0.45786
Internal Energy (U)= -6558.4655 0.50092
Enthalpy (H)= -6558.4646 0.50186
Gibbs Free Energy (G)= -6558.5905 0.37591
Entropy (S)= 0.00042245

Frequencies -- 15.2881 20.4216 24.0101
Supporting Information: 030-Selenylated-Cluster-RuII-2HOAc-1OAc-CsBr-07-PBE.log
----
Using Gaussian 16: ES64L-G16RevA.03 25-Dec-2016
==
# pbepbe/gen/auto pseudo=read gfprint gfinput empiricaldispersion=gd3bj
scf=(direct,vshift=200,tight,maxcycle=300,yqc) opt=(gdiis,maxcycle=250)
freq=noraman
#N Geom=AllCheck Guess=TCheck SCRF=Check GenChk RPBEPBE/Gen/Auto Freq
----
Pointgroup= C1 Stoichiometry= C16H28B10BrCsO8RuSe C1[X(C16H28B10BrCsO8RuSe)]
#Atoms= 66
Charge = 0 Multiplicity = 1

SCF Energy= -6558.95586576 Predicted Change= -1.328442D-08
==
Optimization completed on the basis of negligible forces. {Found 2 times}

Item	Max Val.	Criteria	Pass?	RMS Val.	Criteria	Pass?
Force	0.00000 ||	0.00045	[YES]	0.00000 ||	0.00030	[YES]
Displ	0.00270 ||	0.00180	[NO]	0.00270 ||	0.00180	[YES]

Atomic Coordinates (Angstroms)
Type X Y Z

B	5.037882	-0.322035	-0.620867
B	5.385211	-1.310903	0.832058
H	6.375689	-1.173454	1.489258
H	5.759331	0.547458	-1.016865
B	4.669786	-2.921562	0.578480
B	3.828062	-1.732280	1.600639
B	4.114320	-1.337947	-1.757573
B	3.281411	-0.172315	-0.709169
H	4.027160	-1.249984	-2.944861
H	3.617449	-1.975292	2.752618
H	5.019750	-3.963692	1.046866
C	2.689005	-1.760797	-0.908423
C	2.984642	-2.654502	0.440404
B	5.423453	-2.063572	-0.790834
B	3.907774	-2.941269	-1.019050
H	3.650220	-3.929420	-1.629251
H	6.425751	-2.475684	-1.297474
B	4.065405	-0.130543	0.875168
H	4.066281	0.864981	1.536026
B	2.522903	-0.973431	0.648235
H	1.569153	-0.731232	1.541699
C	1.354270	-2.060664	-1.629489
O	1.304702	-2.848181	-2.571462
O	0.339118	-1.421416	-1.112356
C	1.952326	-3.703114	0.824508
H	2.350896	-4.308675	1.652252
H	1.014154	-3.223033	1.146814
H	1.738837	-4.365625	-0.028474
Ru	0.482421	-0.048628	0.435571
O	-0.202971	1.569535	1.744152
C	-1.192137	1.809457	0.921448
O	-1.200878	1.131048	-0.167471
C	-2.286564	2.753975	1.276336
H	-1.935781	3.516185	1.987585
H	-2.715133	3.213640	0.373431
H	-3.090213	2.143572	1.742701
Se	1.918609	1.256985	-1.161215
C	2.845629	2.742858	-0.313854
C	3.892622	3.344968	-1.027474
C	2.453375	3.214770	0.944992
C	4.572586	4.429104	-0.455656
H	4.185043	2.963751	-2.010873
C	3.136764	4.306980	1.500501
H	1.635405	2.727725	1.482796
C	4.194564	4.911347	0.806167
H	5.399325	4.895869	-1.000461

H	2.840844	4.680261	2.486295
H	4.726760	5.759296	1.248580
Br	-4.405373	-0.242412	1.588319
Cs	-2.785172	-1.414051	-1.365179
O	-0.827175	-1.429374	1.464189
C	-1.233945	-1.338507	2.654977
O	-0.972665	-0.319010	3.449775
C	-2.079755	-2.412570	3.261589
H	-3.130354	-2.116600	3.063149
H	-1.879333	-3.381460	2.782097
H	-1.924191	-2.466826	4.349192
H	-0.582001	0.441618	2.906293
O	-4.687218	0.818899	-2.194649
C	-5.141184	1.857259	-1.706271
O	-5.285142	2.090147	-0.396917
C	-5.622595	3.030307	-2.538758
H	-5.326986	2.891608	-3.587896
H	-6.722614	3.098484	-2.476117
H	-5.216626	3.977385	-2.147600
H	-5.006379	1.269375	0.148543

Statistical Thermodynamic Analysis
Temperature= 298.150 Kelvin Pressure= 1.00000 Atm

==

SCF Energy= -6558.95586576 Predicted Change= -1.328442D-08
Zero-point correction (ZPE)= -6558.4975 0.45835
Internal Energy (U)= -6558.4551 0.50072
Enthalpy (H)= -6558.4541 0.50166
Gibbs Free Energy (G)= -6558.5770 0.37882
Entropy (S)= 0.00041203

Frequencies -- 14.5336 19.9806 27.2284
Supporting Information: 030-Selenylated-Cluster-RuII-2HOAc-1OAc-CsBr-08-PBE.log

----

Using Gaussian 16: ES64L-G16RevA.03 25-Dec-2016

==

# pbepbe/gen/auto pseudo=read gfprint gfinput empiricaldispersion=gd3bj
scf=(direct,vshift=200,tight,maxcycle=300,yqc) opt=(gdiis,maxcycle=250)
freq=noraman
#N Geom=AllCheck Guess=TCheck SCRF=Check GenChk RPBEPBE/Gen/Auto Freq

----

Pointgroup= C1 Stoichiometry= C16H28B10BrCsO8RuSe C1[X(C16H28B10BrCsO8RuSe)]
#Atoms= 66
Charge = 0 Multiplicity = 1

SCF Energy= -6558.96583770 Predicted Change= -8.591045D-09

==

Optimization completed. {Found 1 times}

Item	Max Val.		Criteria	Pass?	RMS Val.		Criteria	Pass?
Force	0.00000	||	0.00045	[YES]	0.00000	||	0.00030	[YES]
Displ	0.00216	||	0.00180	[NO]	0.00216	||	0.00180	[YES]

Atomic Type	Coordinates (Angstroms) X	Y	Z
B	-4.505179	1.048096	-0.846524
B	-4.983106	1.781359	0.714143
H	-6.089816	1.692366	1.161484
H	-5.250965	0.407779	-1.530591
B	-4.028294	3.265328	0.930160
B	-3.554375	1.776508	1.786506
B	-3.254454	2.087535	-1.575094
B	-2.803940	0.612548	-0.696804
H	-2.960566	2.215191	-2.725437
H	-3.533364	1.757917	2.982603
H	-4.313170	4.243649	1.555485
C	-1.968301	2.082153	-0.439283
C	-2.400847	2.749389	1.000191
B	-4.613473	2.828615	-0.692112
B	-2.976046	3.458822	-0.481190
H	-2.479408	4.487453	-0.813897
H	-5.436229	3.495508	-1.249298
B	-3.865717	0.404396	0.705489
H	-4.132561	-0.683527	1.121159
B	-2.205934	0.988147	0.906670
H	-1.354143	0.647435	1.834343
C	-0.488032	2.279203	-0.853661
O	-0.159701	3.277263	-1.506650
O	0.315465	1.351974	-0.424452
C	-1.335700	3.517824	1.765184
H	-1.824251	4.162898	2.510700
H	-0.658183	2.820768	2.286277
H	-0.750557	4.151064	1.079850
Ru	-0.322506	-0.244740	0.802097
Se	-1.599746	-0.902987	-1.261548
C	-2.808384	-2.359044	-0.817260
C	-3.889969	-2.614557	-1.673081
C	-2.555919	-3.161618	0.301973
C	-4.750129	-3.682954	-1.385215
H	-4.068783	-1.977943	-2.545243
C	-3.420232	-4.233375	0.572508
H	-1.714585	-2.936057	0.962339

C	-4.515127	-4.493308	-0.264287
H	-5.604827	-3.880453	-2.040235
H	-3.235399	-4.863212	1.448915
H	-5.188060	-5.328010	-0.043146
O	0.891310	0.003533	2.630614
C	0.402351	-1.102944	3.088097
C	0.913917	-1.696141	4.373189
H	1.788590	-2.333280	4.150600
H	1.231830	-0.902527	5.067020
H	0.140181	-2.324748	4.838795
O	-0.503342	-1.695547	2.404909
Cs	3.127425	1.463067	0.893882
Br	1.550352	-1.740036	-0.135958
H	1.562947	-0.746045	-2.135499
O	1.368504	-0.149911	-2.930122
C	2.235296	0.861454	-2.943999
C	1.824389	1.982397	-3.857741
H	1.230407	1.607646	-4.704704
H	1.188075	2.669672	-3.266615
H	2.711025	2.528603	-4.210108
O	3.264694	0.900677	-2.242951
O	5.326738	-0.507245	0.145911
C	5.305234	-1.443182	-0.658037
O	4.650709	-1.413562	-1.827692
H	4.139730	-0.545795	-1.895456
C	5.987504	-2.772732	-0.425427
H	6.736778	-2.676255	0.372447
H	5.225507	-3.510385	-0.116707
H	6.451967	-3.146638	-1.351338

Statistical Thermodynamic Analysis
Temperature= 298.150 Kelvin   Pressure= 1.00000 Atm

==

SCF Energy= -6558.96583770 Predicted Change= -8.591045D-09
Zero-point correction (ZPE)= -6558.5071 0.45866
Internal Energy (U)= -6558.4643 0.50150
Enthalpy (H)= -6558.4633 0.50245
Gibbs Free Energy (G)= -6558.5869 0.37889
Entropy (S)= 0.00041443

Frequencies -- 16.0713 23.6295 26.1425
Supporting Information: 030-Selenylated-Cluster-RuII-2HOAc-1OAc-CsBr-09-PBE.log

----

Using Gaussian 16: ES64L-G16RevA.03 25-Dec-2016

==

pbepbe/gen/auto pseudo=read gfprint gfinput empiricaldispersion=gd3bj

scf=(direct,vshift=200,tight,maxcycle=300,yqc) opt=(gdiis,maxcycle=250)
freq=noraman
#N Geom=AllCheck Guess=TCheck SCRF=Check GenChk RPBEPBE/Gen/Auto Freq
----
Pointgroup= C1 Stoichiometry= C16H28B10BrCsO8RuSe C1[X(C16H28B10BrCsO8RuSe)]
#Atoms= 66
Charge = 0 Multiplicity = 1

SCF Energy= -6558.95009623 Predicted Change= -9.712840D-09
==
Optimization completed. {Found 1 times}

Item	Max Val.	Criteria	Pass?	RMS Val.	Criteria	Pass?
Force	0.00001 ||	0.00045	[YES]	0.00000 ||	0.00030	[YES]
Displ	0.00725 ||	0.00180	[NO]	0.00725 ||	0.00180	[YES]

Atomic Coordinates (Angstroms)

Type	X	Y	Z
B	-3.942638	0.666306	-0.778048
B	-5.309384	-0.309640	-1.337133
H	-6.319526	0.171066	-1.761767
H	-3.935956	1.860725	-0.791459
B	-5.345383	-1.802491	-0.368123
B	-4.673067	-1.827792	-2.014835
B	-3.113044	-0.221386	0.529023
B	-2.471467	-0.251072	-1.108718
H	-2.777046	0.301022	1.667636
H	0.146203	2.777617	1.715851
H	-5.169528	-2.531959	-2.842621
H	-6.287832	-2.489272	-0.108144
C	-2.612564	-1.704546	-0.190348
C	-3.875957	-2.610042	-0.716847
B	-4.897033	-0.264403	0.409301
B	-4.010469	-1.743843	0.793570
H	-3.951269	-2.389540	1.794122
H	-5.596073	0.243460	1.237532
B	-3.787076	-0.304483	-2.284569
H	-3.678770	0.184956	-3.370610
B	-2.907222	-1.785046	-1.893068
H	-2.147024	-2.442353	-2.532205
C	-1.341990	-2.390831	0.359718
O	-0.677474	-1.653579	1.196426
O	-1.018160	-3.523982	-0.021910
Cs	2.202986	-2.311969	0.154888
C	-3.800567	-4.128741	-0.656760
H	-3.017536	-4.509759	-1.324917

H	-3.576857	-4.471183	0.363482
H	-4.779249	-4.528231	-0.963721
Ru	-1.081514	0.389035	1.449731
O	1.036571	0.583721	1.651457
O	-1.360275	0.691352	3.624027
C	1.693949	1.658400	1.552456
C	-1.432976	1.949253	3.456484
O	1.146279	2.860276	1.561789
O	-1.367431	2.405618	2.231208
C	-1.590136	2.908933	4.601397
H	-0.721265	3.588868	4.637521
H	-2.486800	3.531976	4.445222
H	-1.672781	2.362998	5.551579
C	3.168668	1.634829	1.334922
H	3.317540	1.488967	0.239021
H	3.634348	2.580616	1.645283
H	3.628748	0.774412	1.842323
Se	-0.584506	0.436278	-1.014524
C	-0.713339	2.262489	-1.649297
C	0.064972	2.588795	-2.769353
C	-1.466182	3.226879	-0.965457
C	0.053077	3.912263	-3.233242
H	0.706681	1.834163	-3.235275
C	-1.469656	4.543987	-1.445456
H	-2.023119	2.954712	-0.065607
C	-0.717524	4.885002	-2.580080
H	0.661617	4.181304	-4.102305
H	-2.057440	5.305917	-0.923254
H	-0.720051	5.916617	-2.946596
Br	2.992397	0.356262	-2.066799
O	5.025811	-1.316762	0.773954
C	5.949661	-0.623736	0.330039
O	5.858990	0.195056	-0.717836
C	7.343014	-0.615626	0.930029
H	7.363400	-1.228603	1.841646
H	8.066011	-1.015460	0.198454
H	7.654779	0.416926	1.158764
H	4.915205	0.169977	-1.124374

Statistical Thermodynamic Analysis
Temperature= 298.150 Kelvin Pressure= 1.00000 Atm

==

SCF Energy= -6558.95009623 Predicted Change= -9.712840D-09
Zero-point correction (ZPE)= -6558.4916 0.45844
Internal Energy (U)= -6558.4489 0.50111
Enthalpy (H)= -6558.4480 0.50205

Gibbs Free Energy (G)= -6558.5724 0.37762
Entropy (S)= 0.00041736

Frequencies -- 19.0811 21.0685 26.5898
Supporting Information: 030-Selenylated-Cluster-RuII-2HOAc-1OAc-CsBr-10-PBE.log

---

Using Gaussian 16: ES64L-G16RevA.03 25-Dec-2016

==

# pbepbe/gen/auto pseudo=read gfprint gfinput empiricaldispersion=gd3bj
scf=(direct,vshift=200,tight,maxcycle=300,yqc) opt=(gdiis,maxcycle=250)
freq=noraman
#N Geom=AllCheck Guess=TCheck SCRF=Check GenChk RPBEPBE/Gen/Auto Freq

---

Pointgroup= C1 Stoichiometry= C16H28B10BrCsO8RuSe C1[X(C16H28B10BrCsO8RuSe)]
#Atoms= 66
Charge = 0 Multiplicity = 1

SCF Energy= -6558.94963133 Predicted Change= -5.041587D-09

==

Optimization completed on the basis of negligible forces. {Found 2 times}

Item	Max Val.		Criteria	Pass?	RMS Val.		Criteria	Pass?
Force	0.00000	||	0.00045	[YES]	0.00000	||	0.00030	[YES]
Displ	0.00251	||	0.00180	[NO]	0.00251	||	0.00180	[YES]

Atomic Type	Coordinates (Angstroms) X	Y	Z
B	-2.589273	2.003093	1.537749
B	-4.124334	2.875604	1.595616
H	-4.334903	3.762807	2.370562
H	-1.677031	2.248888	2.266747
B	-5.411221	1.738530	1.131406
B	-4.798581	2.923608	-0.048590
B	-2.903631	0.323709	1.023861
B	-2.316265	1.505209	-0.138284
H	-2.346232	-0.658204	1.666731
H	-1.640302	-2.317917	-2.091394
H	-5.547776	3.728729	-0.515288
H	-6.564153	1.765149	1.443487
C	-3.639932	0.476625	-0.531248
C	-5.061670	1.289704	-0.480919
B	-4.048384	1.158658	2.118304
B	-4.670326	0.163024	0.799933
H	-5.255678	-0.875501	0.799735
H	-4.200174	0.809594	3.253094
B	-3.038542	3.089645	0.182741

H	-2.458231	4.106723	-0.062359
B	-3.668160	2.096034	-1.132382
H	-3.617219	2.221827	-2.315226
C	-3.471741	-0.657595	-1.559530
O	-2.601190	-1.551749	-1.151608
O	-4.100314	-0.681318	-2.610930
Cs	2.914276	-1.222373	0.924854
C	-6.190539	0.916374	-1.431173
H	-5.915316	1.127013	-2.472501
H	-6.436218	-0.151919	-1.351756
H	-7.074105	1.510375	-1.151686
Ru	-1.287204	-1.255681	0.459241
O	0.205047	-2.385492	-0.554987
O	-1.639706	-3.014798	1.696020
C	0.188552	-2.831614	-1.739724
C	-0.647536	-2.706031	2.440883
O	-0.841821	-2.780848	-2.547309
O	-0.003234	-1.610949	2.188012
C	-0.218395	-3.603794	3.572926
H	0.410721	-4.422935	3.180312
H	0.357958	-3.037853	4.320601
H	-1.101385	-4.062534	4.044179
C	1.432293	-3.425113	-2.328351
H	1.973170	-4.016055	-1.572366
H	1.197738	-4.044589	-3.205171
H	2.074069	-2.570295	-2.635242
Se	-0.567294	0.785782	-0.858951
C	0.652128	1.845032	0.223828
C	1.453542	2.779822	-0.446579
C	0.787787	1.628340	1.603347
C	2.380782	3.535537	0.286252
H	1.380295	2.888001	-1.531797
C	1.719114	2.390582	2.327379
H	0.177391	0.866393	2.097610
C	2.512589	3.344158	1.669866
H	3.016941	4.255375	-0.237073
H	1.818928	2.239155	3.407856
H	3.239643	3.934117	2.237728
Br	2.669960	0.005665	-2.357559
O	5.101436	0.845742	0.587785
C	5.356003	1.835932	-0.105901
O	4.837436	2.086372	-1.309026
H	4.173021	1.352458	-1.579162
C	6.284767	2.944712	0.351599
H	6.988265	2.557362	1.102532
H	6.827187	3.389674	-0.496440

H 5.680456 3.742514 0.820238

Statistical Thermodynamic Analysis
Temperature= 298.150 Kelvin Pressure= 1.00000 Atm

===

SCF Energy= -6558.94963133 Predicted Change= -5.041587D-09
Zero-point correction (ZPE)= -6558.4912 0.45839
Internal Energy (U)= -6558.4487 0.50084
Enthalpy (H)= -6558.4478 0.50179
Gibbs Free Energy (G)= -6558.5700 0.37962
Entropy (S)= 0.00040975

Frequencies -- 22.3433 23.8369 31.7703
Supporting Information: 030-Selenylated-Cluster-RuII-2HOAc-1OAc-CsBr-11-PBE.log

---

Using Gaussian 16: ES64L-G16RevA.03 25-Dec-2016

===

# pbepbe/gen/auto pseudo=read gfprint gfinput empiricaldispersion=gd3bj
scf=(direct,vshift=200,tight,maxcycle=300,yqc) opt=(gdiis,maxcycle=250)
freq=noraman
#N Geom=AllCheck Guess=TCheck SCRF=Check GenChk RPBEPBE/Gen/Auto Freq

---

Pointgroup= C1 Stoichiometry= C16H28B10BrCsO8RuSe C1[X(C16H28B10BrCsO8RuSe)]
#Atoms= 66
Charge = 0 Multiplicity = 1

SCF Energy= -6558.93509263 Predicted Change= -6.347796D-09

===

Optimization completed on the basis of negligible forces. {Found 2 times}

Item	Max Val.	Criteria	Pass?	RMS Val.	Criteria	Pass?
Force	0.00000 ||	0.00045	[YES]	0.00000 ||	0.00030	[YES]
Displ	0.00344 ||	0.00180	[NO]	0.00344 ||	0.00180	[YES]

Atomic Type	Coordinates (Angstroms) X	Y	Z
B	-3.995850	0.299265	-2.156270
B	-5.331631	1.150488	-1.354601
H	-6.478164	1.016384	-1.671921
H	-4.136968	-0.473016	-3.059767
B	-4.715336	2.720886	-0.799712
B	-4.881542	1.377954	0.350947
B	-2.550721	1.342429	-2.091260
B	-2.740442	0.007626	-0.941266
H	-1.621350	1.364593	-2.836768
H	3.419514	-0.880640	-2.015579

H	-5.596150	1.485760	1.303198
H	-5.318021	3.738697	-0.626945
C	-2.206733	1.556110	-0.422625
C	-3.492512	2.367855	0.332595
B	-4.156575	2.064154	-2.350062
B	-3.006454	2.843667	-1.252274
H	-2.412716	3.870274	-1.359350
H	-4.432131	2.591914	-3.388375
B	-4.435878	-0.122776	-0.473553
H	-4.898546	-1.180075	-0.157893
B	-3.270530	0.677980	0.611290
H	-2.853332	0.351179	1.676470
C	-0.779102	1.917074	0.051725
O	0.224291	1.159696	-0.269708
O	-0.616050	2.971644	0.687124
Cs	3.281156	1.708164	0.728874
C	-3.259410	3.312264	1.502138
H	-2.727397	2.803363	2.316880
H	-2.675643	4.188615	1.195886
H	-4.248536	3.630487	1.867557
Ru	0.640594	-0.641644	-1.089726
O	4.935927	-0.727193	-0.258115
O	2.451812	0.545024	-2.084085
C	4.514918	-1.779868	-0.737799
C	1.669745	0.693474	-3.108470
O	3.701320	-1.825432	-1.816606
O	0.495814	0.188910	-3.069008
C	2.137667	1.443788	-4.327100
H	1.353839	1.458114	-5.097609
H	2.400947	2.479186	-4.049765
H	3.049372	0.970714	-4.730979
C	4.796588	-3.152761	-0.181775
H	3.931928	-3.432019	0.447814
H	4.889523	-3.902030	-0.982921
H	5.701585	-3.130364	0.441118
Se	-1.538185	-1.599410	-1.086272
C	-2.181579	-2.631520	0.450793
C	-2.939397	-3.769506	0.135753
C	-1.910136	-2.293767	1.782902
C	-3.444794	-4.571169	1.169761
H	-3.139734	-4.021287	-0.910795
C	-2.417871	-3.102213	2.809265
H	-1.314793	-1.413074	2.019718
C	-3.183517	-4.237996	2.506337
H	-4.042215	-5.455999	0.927203
H	-2.208022	-2.840272	3.851498

H	-3.575364	-4.865612	3.313301
Br	1.479275	-1.595562	1.135838
O	1.693030	1.750106	3.330992
C	0.481647	1.579088	3.465181
O	-0.179203	0.506769	2.988127
H	0.467711	-0.076360	2.477719
C	-0.437959	2.566724	4.143657
H	0.100330	3.083373	4.951604
H	-0.731499	3.314343	3.385990
H	-1.346262	2.081642	4.531549

Statistical Thermodynamic Analysis
Temperature= 298.150 Kelvin Pressure= 1.00000 Atm

==

SCF Energy= -6558.93509263 Predicted Change= -6.347796D-09
Zero-point correction (ZPE)= -6558.4744 0.46064
Internal Energy (U)= -6558.4316 0.50341
Enthalpy (H)= -6558.4307 0.50435
Gibbs Free Energy (G)= -6558.5546 0.38043
Entropy (S)= 0.00041566

Frequencies -- 17.0413 22.9475 25.5997
Supporting Information: 030-Selenylated-Cluster-RuII-2HOAc-1OAc-CsBr-12-PBE.log

----

Using Gaussian 16: ES64L-G16RevA.03 25-Dec-2016

==

# pbepbe/gen/auto pseudo=read gfprint gfinput empiricaldispersion=gd3bj
scf=(direct,vshift=200,tight,maxcycle=300,yqc) opt=(gdiis,maxcycle=250)
freq=noraman
#N Geom=AllCheck Guess=TCheck SCRF=Check GenChk RPBEPBE/Gen/Auto Freq

----

Pointgroup= C1 Stoichiometry= C16H28B10BrCsO8RuSe C1[X(C16H28B10BrCsO8RuSe)]
#Atoms= 66
Charge = 0 Multiplicity = 1

SCF Energy= -6558.95173259 Predicted Change= -1.941357D-08

==

Optimization completed on the basis of negligible forces. {Found 2 times}

Item	Max Val.	Criteria	Pass?	RMS Val.	Criteria	Pass?
Force	0.00000 ||	0.00045	[YES]	0.00000 ||	0.00030	[YES]
Displ	0.00369 ||	0.00180	[NO]	0.00369 ||	0.00180	[YES]

Atomic Type	Coordinates (Angstroms) X	Y	Z
B	-2.743584	3.272305	0.351423

B	-1.906626	3.542887	1.909909
H	-2.437781	4.056344	2.851797
H	-3.888949	3.561521	0.156649
B	-0.191636	3.842392	1.556000
B	-0.707245	2.232958	2.124875
B	-1.530428	3.401268	-0.949479
B	-2.040700	1.814181	-0.342663
H	-1.688381	3.691583	-2.097440
H	1.653948	-2.626460	1.190654
H	-0.271669	1.798742	3.150777
H	0.595441	4.474140	2.197330
C	-0.388657	2.165944	-0.606146
C	0.380367	2.407663	0.830285
B	-1.435436	4.488222	0.461364
B	0.036537	3.769639	-0.197584
H	0.957434	4.252528	-0.775597
H	-1.611965	5.667694	0.359657
B	-2.288993	1.889456	1.406043
H	-3.090646	1.185846	1.942524
B	-0.840672	1.119097	0.724586
H	-0.332491	-0.032734	1.156615
C	0.453989	1.468081	-1.701851
O	0.262522	0.188078	-1.797127
O	1.280952	2.121071	-2.355679
Cs	3.267486	-0.467756	-1.828018
C	1.830763	1.978615	0.947108
H	1.909799	0.881044	1.009869
H	2.416036	2.342205	0.088276
H	2.261339	2.402675	1.866439
Ru	-0.709252	-0.899004	-0.244258
O	3.353584	-1.009314	1.381695
O	1.070801	-2.293739	-0.269980
C	2.960310	-1.760716	2.303877
C	0.362801	-3.076145	-1.041567
O	2.053661	-2.700810	2.131349
O	-0.718220	-2.622390	-1.527654
C	0.805021	-4.489455	-1.300981
H	0.370103	-4.863778	-2.239587
H	1.904168	-4.568786	-1.322521
H	0.434913	-5.118716	-0.471533
C	3.452625	-1.648491	3.725489
H	2.733442	-1.031979	4.294380
H	3.489214	-2.637809	4.205424
H	4.436822	-1.159660	3.761459
Se	-2.712493	0.165220	-1.291259
C	-4.430187	0.058407	-0.379392

C	-5.427515	0.955482	-0.793120
C	-4.681095	-0.918085	0.591210
C	-6.696671	0.884366	-0.203028
H	-5.213919	1.709526	-1.557426
C	-5.958763	-0.980188	1.168184
H	-3.880731	-1.593908	0.910203
C	-6.963335	-0.083895	0.776670
H	-7.476074	1.588766	-0.511368
H	-6.160968	-1.734193	1.935935
H	-7.955847	-0.136968	1.235836
Br	-1.566678	-2.446742	1.594853
O	5.225205	1.230276	-0.185307
C	5.430739	1.638699	0.960773
O	4.912101	1.066528	2.059603
C	6.267414	2.854683	1.289414
H	6.825301	3.177463	0.400014
H	5.605351	3.674962	1.618364
H	6.957196	2.640427	2.121429
H	4.344965	0.271316	1.774017

Statistical Thermodynamic Analysis
Temperature= 298.150 Kelvin   Pressure= 1.00000 Atm
===
SCF Energy= -6558.95173259   Predicted Change= -1.941357D-08
Zero-point correction (ZPE)=   -6558.4935   0.45822
Internal Energy (U)=   -6558.4505   0.50119
Enthalpy (H)=   -6558.4495   0.50213
Gibbs Free Energy (G)=   -6558.5745   0.37720
Entropy (S)=   0.00041902

Frequencies -- 19.0469   21.7824   23.8104
Supporting Information: 030-Selenylated-Cluster-RuII-2HOAc-1OAc-CsBr-13-PBE.log
----
Using Gaussian 16: ES64L-G16RevA.03 25-Dec-2016
===
# pbepbe/gen/auto pseudo=read gfprint gfinput empiricaldispersion=gd3bj
scf=(direct,vshift=200,tight,maxcycle=300,yqc) opt=(gdiis,maxcycle=250)
freq=noraman
#N Geom=AllCheck Guess=TCheck SCRF=Check GenChk RPBEPBE/Gen/Auto Freq
----
Pointgroup= C1   Stoichiometry= C16H28B10BrCsO8RuSe   C1[X(C16H28B10BrCsO8RuSe)]
#Atoms= 66
Charge = 0   Multiplicity = 1

SCF Energy= -6558.95112921   Predicted Change= -8.075763D-09
===

Optimization completed. {Found 1 times}

Item	Max Val.		Criteria	Pass?	RMS Val.		Criteria	Pass?
Force	0.00001	||	0.00045	[YES]	0.00000	||	0.00030	[YES]
Displ	0.00434	||	0.00180	[NO]	0.00434	||	0.00180	[YES]

Atomic Type	Coordinates (Angstroms) X	Y	Z
B	3.058178	-2.185394	2.043973
B	2.773283	-1.212839	3.518887
H	3.626520	-0.996579	4.329898
H	4.120250	-2.658016	1.758776
B	1.088010	-1.472551	4.017555
B	1.528961	0.014611	3.140036
B	1.542849	-3.033108	1.649302
B	1.984475	-1.533873	0.807293
H	1.382406	-4.044980	1.035846
H	-1.339653	3.292057	0.172521
H	1.367494	1.080490	3.657821
H	0.619362	-1.395259	5.114807
C	0.401967	-1.776026	1.382280
C	0.136009	-0.870853	2.731316
B	2.021242	-2.831745	3.352470
B	0.335920	-2.598140	2.877425
H	-0.644072	-3.232443	3.109068
H	2.316033	-3.767717	4.037465
B	2.759237	-0.414966	1.937986
H	3.582426	0.363311	1.560831
B	1.085250	-0.152954	1.410425
H	0.570026	1.044551	1.096656
C	-0.786650	-1.943906	0.415156
O	-0.915681	-1.022629	-0.475234
O	-1.554932	-2.918652	0.579126
Cs	-3.790856	0.216939	0.003831
C	-1.261532	-0.327804	2.971326
H	-1.440858	0.594701	2.394979
H	-2.016415	-1.089499	2.714826
H	-1.371622	-0.090326	4.040071
Ru	0.404286	0.673260	-0.535968
O	-2.826276	2.492574	1.835861
O	-1.368905	1.973977	-0.913860
C	-2.194662	3.557738	1.872099
C	-1.080209	1.908575	-2.185545
O	-1.396390	3.993696	0.895745
O	-0.202236	1.069189	-2.559791
C	-1.742687	2.837380	-3.163609

H	-1.734425	2.402773	-4.174246
H	-2.771928	3.079932	-2.852852
H	-1.163905	3.778262	-3.184024
C	-2.217445	4.502315	3.053320
H	-1.299379	4.349309	3.648260
H	-2.218190	5.551594	2.719696
H	-3.090595	4.294167	3.687212
Se	2.083709	-1.093507	-1.156517
C	3.984178	-0.664719	-1.193629
C	4.873658	-1.750282	-1.156396
C	4.439977	0.651164	-1.328993
C	6.251394	-1.506014	-1.233609
H	4.497749	-2.773537	-1.056660
C	5.822481	0.878298	-1.410363
H	3.726901	1.481592	-1.335862
C	6.726459	-0.192127	-1.361575
H	6.951831	-2.346462	-1.192965
H	6.188923	1.905670	-1.505362
H	7.803409	-0.004419	-1.421439
Br	1.695859	2.859851	-0.659912
O	-4.573194	-2.425457	-1.202413
C	-4.135740	-3.462939	-1.714539
O	-2.965487	-4.022265	-1.404855
C	-4.877650	-4.250094	-2.774871
H	-5.828198	-3.755216	-3.016269
H	-4.257042	-4.337977	-3.682435
H	-5.070127	-5.275346	-2.416332
H	-2.499103	-3.484160	-0.677490

Statistical Thermodynamic Analysis
Temperature= 298.150 Kelvin   Pressure= 1.00000 Atm

===

SCF Energy=   -6558.95112921   Predicted Change= -8.075763D-09
Zero-point correction (ZPE)=   -6558.4931   0.45794
Internal Energy (U)=   -6558.4499   0.50120
Enthalpy (H)=   -6558.4489   0.50215
Gibbs Free Energy (G)=   -6558.5761   0.37499
Entropy (S)=   0.00042648

Frequencies --   10.7558   19.1374   19.8517
Supporting Information: 030-Selenylated-Cluster-RuII-2HOAc-1OAc-CsBr-14-PBE.log

----

Using Gaussian 16: ES64L-G16RevA.03 25-Dec-2016

===

# pbepbe/gen/auto pseudo=read gfprint gfinput empiricaldispersion=gd3bj
scf=(direct,vshift=200,tight,maxcycle=300,yqc) opt=(gdiis,maxcycle=250)

freq=noraman
#N Geom=AllCheck Guess=TCheck SCRF=Check GenChk RPBEPBE/Gen/Auto Freq
----
Pointgroup= C1 Stoichiometry= C16H28B10BrCsO8RuSe C1[X(C16H28B10BrCsO8RuSe)]
#Atoms= 66
Charge = 0 Multiplicity = 1

SCF Energy= -6558.96744908 Predicted Change= -2.231979D-08
===
Optimization completed. {Found 1 times}

Item	Max Val.		Criteria	Pass?	RMS Val.		Criteria	Pass?
Force	0.00001	||	0.00045	[YES]	0.00000	||	0.00030	[YES]
Displ	0.00373	||	0.00180	[NO]	0.00373	||	0.00180	[YES]

Atomic Type	Coordinates (Angstroms) X	Y	Z
B	-4.465039	1.614535	-0.091003
B	-4.248178	2.525994	1.431858
H	-5.128578	2.677003	2.228694
H	-5.491450	1.081328	-0.401541
B	-3.063912	3.814211	1.126839
B	-2.552800	2.324962	1.960162
B	-3.401840	2.347968	-1.321035
B	-2.909880	0.876485	-0.461408
H	-3.516026	2.341021	-2.509647
H	2.239646	1.113755	2.089873
H	-2.115540	2.379908	3.072224
H	-2.958378	4.868659	1.680124
C	-1.816491	2.172838	-0.688636
C	-1.611639	3.012963	0.718504
B	-4.235391	3.388696	-0.139483
B	-2.547589	3.710737	-0.565340
H	-2.047958	4.602637	-1.173593
H	-5.087578	4.159632	-0.473502
B	-3.428535	0.965982	1.228735
H	-3.694032	-0.022698	1.844976
B	-1.736531	1.249329	0.784188
H	-0.663504	0.813017	1.362170
C	-0.560206	2.048876	-1.592198
O	0.128886	0.960917	-1.400251
O	-0.270954	2.954573	-2.378599
Cs	3.121622	0.574939	-1.777588
C	-0.251018	3.633624	0.989698
H	0.386302	2.947728	1.569044
H	0.252733	3.888591	0.043948

H	-0.393517	4.558686	1.568886
Ru	-0.240177	-0.371636	0.184696
O	3.607026	2.409441	0.651989
O	1.575803	-0.259988	1.458820
C	3.266626	2.727371	1.791820
C	1.072726	-1.163859	2.253271
O	2.495888	1.963054	2.586477
O	-0.076801	-1.625653	1.963201
C	1.849210	-1.647823	3.444674
H	1.170094	-2.078180	4.194985
H	2.547326	-2.431882	3.102426
H	2.434797	-0.824241	3.884209
C	3.635269	4.037393	2.453184
H	2.749912	4.697785	2.470185
H	3.941042	3.876349	3.499128
H	4.437973	4.530205	1.887959
Se	-2.252799	-0.875705	-1.209393
C	-3.444933	-2.043721	-0.214024
C	-4.791516	-2.116930	-0.599613
C	-2.937124	-2.836019	0.822809
C	-5.651237	-2.985265	0.086748
H	-5.169495	-1.492504	-1.415105
C	-3.807388	-3.707225	1.495226
H	-1.886476	-2.754225	1.113029
C	-5.160015	-3.781164	1.132488
H	-6.706865	-3.037824	-0.198572
H	-3.421477	-4.326618	2.311487
H	-5.834052	-4.459454	1.665434
Br	0.867738	-2.265303	-1.142983
O	4.630328	-1.696985	-0.356123
C	4.467144	-2.501398	0.560388
O	3.306192	-3.130017	0.819536
C	5.544540	-2.891447	1.550346
H	5.567316	-3.983813	1.692300
H	6.521135	-2.529515	1.201168
H	5.321793	-2.437757	2.532518
H	2.601621	-2.814187	0.165549

Statistical Thermodynamic Analysis
Temperature= 298.150 Kelvin Pressure= 1.00000 Atm

==

SCF Energy= -6558.96744908 Predicted Change= -2.231979D-08
Zero-point correction (ZPE)= -6558.5091 0.45830
Internal Energy (U)= -6558.4659 0.50151
Enthalpy (H)= -6558.4649 0.50245
Gibbs Free Energy (G)= -6558.5911 0.37629

Entropy (S)= 0.00042314

Frequencies -- 14.4859 22.2027 22.5663

Using Gaussian 16: ES64L-G16RevA.03 25-Dec-2016

==

\# pbepbe/gen/auto pseudo=read gfprint gfinput empiricaldispersion=gd3bj
scf=(direct,vshift=200,tight,maxcycle=300,yqc) opt=(gdiis,maxcycle=250)
freq=noraman
\#N Geom=AllCheck Guess=TCheck SCRF=Check GenChk RPBEPBE/Gen/Auto Freq

---

Pointgroup= C1 Stoichiometry= C16H28B10BrCsO8RuSe C1[X(C16H28B10BrCsO8RuSe)]
\#Atoms= 66
Charge = 0 Multiplicity = 1

SCF Energy= -6558.95132002 Predicted Change= -4.547543D-09

==

Optimization completed. {Found 1 times}

Item	Max Val.	Criteria	Pass?	RMS Val.	Criteria	Pass?
Force	0.00000 ||	0.00045	[YES]	0.00000 ||	0.00030	[YES]
Displ	0.00405 ||	0.00180	[NO]	0.00405 ||	0.00180	[YES]

Atomic Type	Coordinates (Angstroms) X	Y	Z
B	-4.472414	1.612551	0.456915
B	-4.538222	1.468199	2.241360
H	-5.549251	1.234426	2.836857
H	-5.424064	1.467083	-0.254461
B	-3.304601	2.557854	2.921987
B	-2.973221	0.813605	2.802454
B	-3.201903	2.795609	0.057304
B	-2.869512	1.052366	-0.027680
H	-3.085342	3.477420	-0.915485
H	-2.752483	0.176680	3.790100
H	-3.302366	3.090206	3.991617
C	-1.764320	2.126012	0.705098
C	-1.806208	1.967847	2.341969
B	-4.233168	3.061029	1.484946
B	-2.500059	3.385381	1.580687
H	-1.890158	4.401042	1.687209
H	-5.006770	3.971618	1.540765
B	-3.702859	0.224874	1.295358
H	-4.076383	-0.903695	1.175988
B	-1.956876	0.513449	1.366147

H	-1.181352	-0.499707	1.729360
C	-0.381060	2.405746	0.088364
O	-0.063737	3.577526	-0.167941
O	0.343970	1.350560	-0.094027
C	-0.499218	2.154344	3.096692
H	-0.707360	2.138253	4.177019
H	0.208159	1.344632	2.854193
H	-0.037241	3.120483	2.840997
Ru	-0.346307	-0.592036	0.258017
O	-0.368066	-2.757374	0.141521
C	0.525494	-2.721404	-0.815668
O	0.843789	-1.550104	-1.239154
C	1.192105	-3.950547	-1.312672
H	0.613252	-4.849212	-1.054975
H	1.362999	-3.886014	-2.398660
H	2.191448	-3.978790	-0.809080
Se	-2.053750	-0.053362	-1.515464
C	-3.483276	-1.368429	-1.636316
C	-4.625036	-1.012157	-2.369509
C	-3.358516	-2.632028	-1.045479
C	-5.671280	-1.936519	-2.494030
H	-4.704483	-0.020400	-2.825574
C	-4.410058	-3.550214	-1.186989
H	-2.460477	-2.887049	-0.476406
C	-5.563880	-3.205074	-1.905131
H	-6.570954	-1.661380	-3.053513
H	-4.324443	-4.539002	-0.724856
H	-6.382100	-3.924975	-2.006040
Br	4.090130	-2.899057	0.537357
Cs	3.396672	0.313292	-0.715534
O	1.285178	-0.687876	1.667170
C	1.524466	-1.629751	2.476512
O	0.836891	-2.753508	2.521542
C	2.641225	-1.527569	3.461872
H	3.516616	-1.979293	2.943024
H	2.855804	-0.476537	3.703296
H	2.420205	-2.110453	4.368211
H	0.270330	-2.834142	1.688191
O	3.424159	3.301755	-1.305728
C	3.088542	4.443053	-1.634385
O	1.870908	4.955679	-1.432461
C	4.010504	5.431696	-2.317256
H	5.001066	4.981397	-2.467675
H	3.584982	5.735780	-3.288476
H	4.103265	6.345353	-1.706319
H	1.283141	4.278193	-0.968569

Statistical Thermodynamic Analysis
Temperature= 298.150 Kelvin Pressure= 1.00000 Atm
==
SCF Energy= -6558.95132002 Predicted Change= -4.547543D-09
Zero-point correction (ZPE)= -6558.4923 0.45898
Internal Energy (U)= -6558.4500 0.50123
Enthalpy (H)= -6558.4491 0.50218
Gibbs Free Energy (G)= -6558.5726 0.37869
Entropy (S)= 0.00041417

Frequencies -- 8.2549 20.7247 27.6520
Supporting Information: 030-Selenylated-Cluster-RuII-2HOAc-1OAc-CsBr-16-PBE.log
----
Using Gaussian 16: ES64L-G16RevA.03 25-Dec-2016
==
# pbepbe/gen/auto pseudo=read gfprint gfinput empiricaldispersion=gd3bj
scf=(direct,vshift=200,tight,maxcycle=300,yqc) opt=(gdiis,maxcycle=250)
freq=noraman
#N Geom=AllCheck Guess=TCheck SCRF=Check GenChk RPBEPBE/Gen/Auto Freq
----
Pointgroup= C1 Stoichiometry= C16H28B10BrCsO8RuSe C1[X(C16H28B10BrCsO8RuSe)]
#Atoms= 66
Charge = 0 Multiplicity = 1

SCF Energy= -6558.96553487 Predicted Change= -8.608646D-09
==
Optimization completed. {Found 1 times}

Item	Max Val.	Criteria	Pass?	RMS Val.	Criteria	Pass?
Force	0.00000 ||	0.00045	[YES]	0.00000 ||	0.00030	[YES]
Displ	0.00183 ||	0.00180	[NO]	0.00183 ||	0.00180	[YES]

Atomic Type	Coordinates (Angstroms) X	Y	Z
B	4.585548	-0.837760	0.441517
B	4.655736	-0.877442	2.229236
H	5.604096	-0.493167	2.850341
H	5.468144	-0.408087	-0.244441
B	3.713662	-2.282217	2.777694
B	2.996858	-0.653085	2.852768
B	3.594841	-2.219820	-0.093672
B	2.893910	-0.593225	0.011864
H	3.614736	-2.798800	-1.137738
H	2.663143	-0.195967	3.906838
H	3.849448	-2.916419	3.781965

C	2.056889	-1.954215	0.618038
C	2.112598	-1.982271	2.262293
B	4.692814	-2.407277	1.297382
B	3.073048	-3.112161	1.350364
H	2.705822	-4.243702	1.338523
H	5.649062	-3.125315	1.251730
B	3.546305	0.249085	1.426832
H	3.667389	1.437678	1.437253
B	1.908022	-0.421698	1.449048
H	0.839505	0.137884	1.952555
C	0.738482	-2.470757	-0.014179
O	0.617111	-3.667322	-0.295943
O	-0.175702	-1.556403	-0.153939
C	0.901906	-2.537958	2.994372
H	1.188554	-2.782222	4.028166
H	0.089265	-1.792718	3.015489
H	0.541265	-3.454427	2.501514
Ru	0.083870	0.441581	0.455293
Se	1.835852	0.442260	-1.356354
C	2.848547	2.097870	-1.253859
C	4.093760	2.158290	-1.896716
C	2.308160	3.212007	-0.600157
C	4.821745	3.355447	-1.862537
H	4.497889	1.277366	-2.405331
C	3.044157	4.406455	-0.581820
H	1.342364	3.137982	-0.093494
C	4.297103	4.479681	-1.207430
H	5.800536	3.406996	-2.350223
H	2.633215	5.281732	-0.068087
H	4.867529	5.413777	-1.184307
O	-1.543316	0.726499	1.913296
C	-1.207001	1.969201	2.037577
C	-1.999607	2.903870	2.911365
H	-2.668525	3.501298	2.267737
H	-2.605529	2.337600	3.634793
H	-1.323034	3.595208	3.437543
O	-0.193027	2.391205	1.378453
Cs	-3.249151	-1.572109	0.265833
Br	-1.512699	1.210327	-1.428359
H	-0.993264	-0.693190	-2.756029
O	-0.541258	-1.464207	-3.202178
C	-1.311691	-2.556882	-3.026001
C	-0.614460	-3.817882	-3.472622
H	0.081118	-3.625254	-4.303213
H	-0.033081	-4.188687	-2.608394
H	-1.357485	-4.578131	-3.753150

O	-2.434029	-2.522992	-2.519225
O	-4.984658	0.956358	0.463668
C	-4.893465	2.177962	0.577542
O	-3.869844	2.908941	0.096932
H	-3.207783	2.297735	-0.351589
C	-5.904877	3.044651	1.295844
H	-6.138521	3.943701	0.703612
H	-6.818045	2.466922	1.493203
H	-5.478695	3.386913	2.255702

Statistical Thermodynamic Analysis
Temperature= 298.150 Kelvin Pressure= 1.00000 Atm

==

SCF Energy= -6558.96553487 Predicted Change= -8.608646D-09
Zero-point correction (ZPE)= -6558.5070 0.45851
Internal Energy (U)= -6558.4638 0.50164
Enthalpy (H)= -6558.4629 0.50258
Gibbs Free Energy (G)= -6558.5872 0.37829
Entropy (S)= 0.00041686

Frequencies -- 20.4927 23.5762 27.3898

Supporting Information: 030-Selenylated-Cluster-RuII-2HOAc-1OAc-CsBr-17-PBE.log

----

Using Gaussian 16: ES64L-G16RevA.03 25-Dec-2016

==

# pbepbe/gen/auto pseudo=read gfprint gfinput empiricaldispersion=gd3bj
scf=(direct,vshift=200,tight,maxcycle=300,yqc) opt=(gdiis,maxcycle=250)
freq=noraman
#N Geom=AllCheck Guess=TCheck SCRF=Check GenChk RPBEPBE/Gen/Auto Freq

----

Pointgroup= C1 Stoichiometry= C16H28B10BrCsO8RuSe C1[X(C16H28B10BrCsO8RuSe)]
#Atoms= 66
Charge = 0 Multiplicity = 1

SCF Energy= -6558.94203994 Predicted Change= -5.343607D-08

==

Optimization completed on the basis of negligible forces. {Found 2 times}

Item	Max Val.	Criteria	Pass?	RMS Val.	Criteria	Pass?
Force	0.00000 ||	0.00045	[YES]	0.00000 ||	0.00030	[YES]
Displ	0.01401 ||	0.00180	[NO]	0.01401 ||	0.00180	[NO]

Atomic Type	Coordinates (Angstroms) X	Y	Z
B	2.035243	3.141714	-0.549130
B	1.624923	4.677771	-1.323927

H	2.442403	5.510633	-1.587702
H	3.148187	2.836911	-0.243282
B	-0.007160	5.140344	-0.788688
B	0.227166	4.408862	-2.392795
B	0.653857	2.628919	0.454537
B	0.896974	1.926184	-1.138979
H	0.695706	2.228295	1.681480
H	2.394402	-1.148537	2.323540
H	-0.083826	5.020664	-3.370767
H	-0.473263	6.237037	-0.703675
C	-0.664767	2.480037	-0.639037
C	-1.046128	3.908977	-1.366954
B	1.101836	4.357699	0.363687
B	-0.603485	3.896941	0.320264
H	-1.482013	4.051895	1.111494
H	1.534279	4.946413	1.311694
B	1.486692	3.155270	-2.261490
H	2.198538	2.870822	-3.179545
B	-0.219769	2.696581	-2.293815
H	-0.859365	2.097450	-3.100991
C	-1.772615	1.472625	-0.287059
O	-1.456951	0.618125	0.623306
O	-2.867214	1.528641	-0.889587
Cs	-2.284659	-2.285013	-0.610523
C	-2.488680	4.239895	-1.722715
H	-2.865358	3.572203	-2.508419
H	-3.145411	4.146116	-0.846879
H	-2.512646	5.280478	-2.081190
Ru	0.481058	0.541026	1.455894
O	0.142576	-1.552643	1.696461
O	0.248167	0.847017	3.640047
C	1.032238	-2.428211	1.904403
C	1.491434	0.646383	3.810781
O	2.284561	-2.150229	2.214745
O	2.223071	0.433185	2.746010
C	2.134571	0.646017	5.167868
H	2.568169	-0.348442	5.372146
H	2.962376	1.374545	5.188724
H	1.395783	0.893654	5.942829
C	0.729646	-3.876332	1.733009
H	0.797830	-4.071455	0.628685
H	1.458843	-4.504744	2.262914
H	-0.295439	-4.100404	2.065983
Se	1.060667	-0.068357	-0.921480
C	2.974466	-0.351780	-1.055217
C	3.412092	-1.175234	-2.101955

C	3.860308	0.166798	-0.101360
C	4.783281	-1.447960	-2.211940
H	2.686761	-1.642139	-2.775209
C	5.227973	-0.115832	-0.227188
H	3.487036	0.767913	0.731071
C	5.689561	-0.915227	-1.283799
H	5.137042	-2.095495	-3.020334
H	5.930854	0.286272	0.509732
H	6.757971	-1.136613	-1.374281
Br	0.742185	-3.547943	-1.793258
O	-5.037569	-1.143257	0.071287
C	-5.652022	-0.154928	0.480770
O	-5.206756	1.103835	0.374196
C	-7.000478	-0.221666	1.165589
H	-7.343010	-1.263864	1.218800
H	-6.927089	0.201242	2.181868
H	-7.735889	0.388230	0.614544
H	-4.308055	1.103449	-0.090681

Statistical Thermodynamic Analysis
Temperature= 298.150 Kelvin   Pressure= 1.00000 Atm

===

SCF Energy=   -6558.94203994   Predicted Change= -5.343607D-08
Zero-point correction (ZPE)=   -6558.4827   0.45928
Internal Energy (U)=   -6558.4402   0.50175
Enthalpy (H)=   -6558.4393   0.50270
Gibbs Free Energy (G)=   -6558.5635   0.37845
Entropy (S)=   0.00041674

Frequencies --   11.1276   19.1482   26.5648
Supporting Information: 030-Selenylated-Cluster-RuII-2HOAc-1OAc-CsBr-18-PBE.log

----

Using Gaussian 16: ES64L-G16RevA.03 25-Dec-2016

===

# pbepbe/gen/auto pseudo=read gfprint gfinput empiricaldispersion=gd3bj
scf=(direct,vshift=200,tight,maxcycle=300,yqc) opt=(gdiis,maxcycle=250)
freq=noraman
#N Geom=AllCheck Guess=TCheck SCRF=Check GenChk RPBEPBE/Gen/Auto Freq

----

Pointgroup= C1   Stoichiometry= C16H28B10BrCsO8RuSe   C1[X(C16H28B10BrCsO8RuSe)]
#Atoms= 66
Charge = 0   Multiplicity = 1

SCF Energy= -6558.93828024   Predicted Change= -2.985564D-08

===

Optimization completed on the basis of negligible forces.   {Found   2   times}

Item	Max Val.	Criteria	Pass?	RMS Val.	Criteria	Pass?
Force	0.00000 ||	0.00045	[YES]	0.00000 ||	0.00030	[YES]
Displ	0.01213 ||	0.00180	[NO]	0.01213 ||	0.00180	[YES]

Atomic Type	Coordinates (Angstroms) X	Y	Z
B	4.302529	1.660946	0.879711
B	5.035226	2.456255	-0.532540
H	6.218255	2.525501	-0.703640
H	4.927378	1.128371	1.752230
B	3.960302	3.786096	-1.020792
B	3.923810	2.247837	-1.907652
B	2.774704	2.499920	1.255895
B	2.760049	0.968357	0.358893
H	2.211685	2.574247	2.307566
H	-2.090661	-2.144836	1.532882
H	4.181904	2.220126	-3.074600
H	4.243495	4.798606	-1.589706
C	1.789427	2.291936	-0.136118
C	2.484033	3.047733	-1.460102
B	4.189496	3.432896	0.705513
B	2.583052	3.819798	0.085841
H	1.895077	4.770762	0.268472
H	4.740657	4.205734	1.434618
B	4.138817	0.926696	-0.744452
H	4.652743	-0.106738	-1.055213
B	2.518731	1.316050	-1.358923
H	1.788872	0.705568	-2.072635
C	0.253746	2.380626	-0.016997
O	-0.434712	1.377568	0.381991
O	-0.275448	3.491918	-0.267659
Cs	-3.878103	-0.568782	-1.361853
C	1.580282	3.656639	-2.521202
H	0.834111	2.924491	-2.864899
H	1.051558	4.534616	-2.128202
H	2.208193	3.949097	-3.376720
Ru	-0.342524	-0.605295	0.760770
O	-2.801418	-3.486380	-0.200780
O	-2.727158	-0.454280	1.564546
C	-1.673480	-3.547570	0.269968
C	-2.161209	0.034831	2.625218
O	-1.305295	-2.754012	1.341028
O	-0.892994	-0.030459	2.777251
C	-2.985039	0.752268	3.664602
H	-2.490863	0.715949	4.646942

H	-3.064380	1.812044	3.359849
H	-3.999229	0.327356	3.726053
C	-0.548121	-4.418504	-0.207455
H	-0.014695	-3.847833	-0.990104
H	0.163772	-4.644671	0.599787
H	-0.953174	-5.339202	-0.651369
Se	1.963585	-0.640847	1.291682
C	3.077380	-2.013794	0.449920
C	4.154690	-2.477802	1.219984
C	2.825966	-2.530839	-0.828170
C	5.001960	-3.464627	0.694839
H	4.335902	-2.062492	2.216732
C	3.675703	-3.522069	-1.339755
H	1.979061	-2.156148	-1.410294
C	4.762223	-3.988187	-0.583721
H	5.848515	-3.824156	1.288901
H	3.488167	-3.926650	-2.340152
H	5.423195	-4.760061	-0.991298
Br	-0.340052	-1.132604	-1.775092
O	-3.717459	2.395323	-0.735800
C	-3.725485	2.989914	0.346939
O	-2.673524	3.587331	0.903296
H	-1.832972	3.465949	0.329651
C	-4.966060	3.084593	1.220043
H	-4.910996	3.933368	1.917657
H	-5.862300	3.169390	0.586516
H	-5.052210	2.156352	1.813668

Statistical Thermodynamic Analysis
Temperature= 298.150 Kelvin Pressure= 1.00000 Atm

==

SCF Energy= -6558.93828024 Predicted Change= -2.985564D-08
Zero-point correction (ZPE)= -6558.4771 0.46112
Internal Energy (U)= -6558.4346 0.50366
Enthalpy (H)= -6558.4336 0.50460
Gibbs Free Energy (G)= -6558.5578 0.38041
Entropy (S)= 0.00041653

Frequencies -- 14.2784 15.9701 23.4348

Supporting Information: 030-Selenylated-Cluster-RuII-2HOAc-1OAc-CsBr-19-PBE.log

----

Using Gaussian 16: ES64L-G16RevA.03 25-Dec-2016

==

# pbepbe/gen/auto pseudo=read gfprint gfinput empiricaldispersion=gd3bj
scf=(direct,vshift=200,tight,maxcycle=300,yqc) opt=(gdiis,maxcycle=250)
freq=noraman

#N Geom=AllCheck Guess=TCheck SCRF=Check GenChk RPBEPBE/Gen/Auto Freq

----

Pointgroup= C1 Stoichiometry= C16H28B10BrCsO8RuSe C1[X(C16H28B10BrCsO8RuSe)]
#Atoms= 66

Charge = 0 Multiplicity = 1

SCF Energy= -6558.93545803 Predicted Change= -3.798798D-08

==

Optimization completed. {Found 1 times}

Item	Max Val.	Criteria	Pass?	RMS Val.	Criteria	Pass?
Force	0.00000 ||	0.00045	[YES]	0.00000 ||	0.00030	[YES]
Displ	0.00890 ||	0.00180	[NO]	0.00890 ||	0.00180	[YES]

Atomic Coordinates (Angstroms)

Type	X	Y	Z
B	4.736522	1.272676	0.431902
B	5.164240	2.322447	-0.937353
H	6.284309	2.445819	-1.341889
H	5.523988	0.606716	1.041057
B	4.033867	3.694304	-0.941634
B	3.783567	2.337801	-2.058888
B	3.340440	1.995763	1.269428
B	3.102324	0.653841	0.138023
H	3.017709	1.875680	2.413377
H	-2.506688	-2.025084	1.688944
H	3.786285	2.522754	-3.239870
H	4.205414	4.798693	-1.365884
C	2.072083	2.021312	0.108746
C	2.485346	3.016484	-1.185329
B	4.617648	3.044624	0.605601
B	2.920369	3.499409	0.417321
H	2.301136	4.387622	0.909461
H	5.322737	3.688830	1.327112
B	4.213544	0.837837	-1.222755
H	4.626795	-0.113438	-1.816305
B	2.504639	1.298768	-1.399375
H	1.631889	0.806107	-2.040455
C	0.610744	2.085945	0.606098
O	0.134165	1.112971	1.324313
O	-0.027299	3.136390	0.419323
Cs	-2.933950	1.635191	0.481632
C	1.409463	3.793826	-1.929521
H	0.566315	3.139593	-2.194676
H	1.030708	4.625422	-1.322167
H	1.859728	4.182870	-2.856091

Ru	0.120739	-0.901014	1.106122
O	-4.100469	-1.369823	0.125900
O	-1.841337	-0.706988	2.431366
C	-3.445902	-2.427382	0.069894
C	-1.052589	-0.885629	3.452702
O	-2.626560	-2.806546	1.050329
O	0.194119	-1.062767	3.251686
C	-1.612724	-0.882470	4.850596
H	-0.819721	-1.079231	5.585980
H	-2.077334	0.095831	5.063961
H	-2.403801	-1.646947	4.939890
C	-3.453333	-3.371932	-1.099955
H	-2.574815	-3.106302	-1.717481
H	-3.337170	-4.415311	-0.770452
H	-4.364586	-3.248451	-1.702096
Se	2.482735	-1.127498	0.858565
C	3.167718	-2.245560	-0.596342
C	4.444060	-2.793866	-0.396467
C	2.435948	-2.514922	-1.759775
C	5.007132	-3.604609	-1.392055
H	4.998041	-2.575420	0.522375
C	3.007949	-3.332917	-2.745525
H	1.438611	-2.084447	-1.890298
C	4.289775	-3.874087	-2.566926
H	6.006684	-4.026973	-1.246033
H	2.444913	-3.543870	-3.660931
H	4.729971	-4.509029	-3.342703
Br	-0.692517	-0.746000	-1.308326
O	-5.228381	1.321071	-1.551745
C	-5.793100	0.451720	-2.220411
O	-5.704154	-0.862665	-1.969580
H	-5.095538	-1.004550	-1.167615
C	-6.665546	0.742634	-3.422820
H	-6.759673	1.827787	-3.563972
H	-6.222352	0.287147	-4.324834
H	-7.662373	0.290419	-3.288865

Statistical Thermodynamic Analysis
Temperature= 298.150 Kelvin   Pressure= 1.00000 Atm

===

SCF Energy= -6558.93545803 Predicted Change= -3.798798D-08
Zero-point correction (ZPE)= -6558.4750 0.46042
Internal Energy (U)= -6558.4322 0.50324
Enthalpy (H)= -6558.4312 0.50418
Gibbs Free Energy (G)= -6558.5572 0.37823
Entropy (S)= 0.00042245

Frequencies -- 11.5399 17.2284 23.6771
Supporting Information: 030-Selenylated-Cluster-RuII-2HOAc-1OAc-CsBr-20-PBE.log
----
Using Gaussian 16: ES64L-G16RevA.03 25-Dec-2016
==
# pbepbe/gen/auto pseudo=read gfprint gfinput empiricaldispersion=gd3bj
scf=(direct,vshift=200,tight,maxcycle=300,yqc) opt=(gdiis,maxcycle=250)
freq=noraman
#N Geom=AllCheck Guess=TCheck SCRF=Check GenChk RPBEPBE/Gen/Auto Freq
----
Pointgroup= C1 Stoichiometry= C16H28B10BrCsO8RuSe C1[X(C16H28B10BrCsO8RuSe)]
#Atoms= 66
Charge = 0 Multiplicity = 1

SCF Energy= -6558.96645354 Predicted Change= -1.597437D-08
==
Optimization completed. {Found 1 times}

Item	Max Val.	Criteria	Pass?	RMS Val.	Criteria	Pass?
Force	0.00000 ||	0.00045	[YES]	0.00000 ||	0.00030	[YES]
Displ	0.00441 ||	0.00180	[NO]	0.00441 ||	0.00180	[YES]

Atomic Type	Coordinates (Angstroms) X	Y	Z
B	-4.447566	-0.445723	1.330798
B	-5.523635	-1.203255	0.136678
H	-6.701342	-0.991509	0.089022
H	-4.811517	0.334017	2.164141
B	-4.867769	-2.811580	-0.238895
B	-4.584195	-1.458035	-1.351210
B	-3.125792	-1.584286	1.687535
B	-2.860247	-0.239811	0.566706
H	-2.471295	-1.670054	2.683041
H	3.786299	0.596826	0.784445
H	-4.977863	-1.513568	-2.478871
H	-5.453211	-3.784783	-0.613027
C	-2.300401	-1.820326	0.198218
C	-3.330752	-2.535292	-0.929233
B	-4.777578	-2.196016	1.424766
B	-3.398299	-3.049365	0.718195
H	-2.936208	-4.114373	0.980190
H	-5.395274	-2.707284	2.313862
B	-4.321442	0.007847	-0.394331
H	-4.605858	1.090384	-0.814662
B	-2.932620	-0.861461	-1.090396

H	-2.188597	-0.546817	-1.963473
C	-0.822264	-2.297493	0.236771
O	0.023318	-1.574208	0.883536
O	-0.563420	-3.414116	-0.253146
Cs	2.560634	-2.734045	-0.554345
C	-2.782077	-3.485117	-1.984221
H	-1.903431	-3.052163	-2.482461
H	-2.490127	-4.444005	-1.538211
H	-3.575082	-3.645468	-2.731533
Ru	0.605682	0.435139	0.793633
O	4.657795	-0.518836	-1.102157
O	2.642704	-0.352982	1.531143
C	4.743962	0.690151	-0.884044
C	2.230591	-0.050725	2.730291
O	4.308830	1.279026	0.245672
O	1.051102	0.407482	2.871532
C	3.140049	-0.230699	3.916568
H	2.604983	-0.003685	4.849659
H	3.524583	-1.264376	3.948612
H	4.010929	0.441776	3.821155
C	5.321033	1.697576	-1.852321
H	4.500479	2.339150	-2.219264
H	6.048957	2.352444	-1.345539
H	5.795878	1.182007	-2.697847
Se	-1.617036	1.250354	1.122270
C	-2.261448	2.633692	-0.103398
C	-3.185146	3.547157	0.427536
C	-1.813202	2.748893	-1.425853
C	-3.680320	4.578360	-0.383451
H	-3.522128	3.443851	1.464152
C	-2.310609	3.787209	-2.226803
H	-1.092378	2.028340	-1.821811
C	-3.242906	4.699637	-1.710535
H	-4.407228	5.288088	0.025125
H	-1.966864	3.878749	-3.262688
H	-3.628528	5.506543	-2.342254
Br	0.659172	0.107899	-1.805185
O	1.462263	2.363639	0.911725
C	1.962246	3.116410	0.044887
O	1.958517	2.888147	-1.260253
H	1.529021	1.982194	-1.445640
C	2.669631	4.379242	0.448469
H	2.366378	4.672635	1.462824
H	3.756354	4.181236	0.441994
H	2.462884	5.188873	-0.268467

Statistical Thermodynamic Analysis
Temperature= 298.150 Kelvin Pressure= 1.00000 Atm
==
SCF Energy= -6558.96645354 Predicted Change= -1.597437D-08
Zero-point correction (ZPE)= -6558.5068 0.45964
Internal Energy (U)= -6558.4644 0.50205
Enthalpy (H)= -6558.4634 0.50299
Gibbs Free Energy (G)= -6558.5853 0.38108
Entropy (S)= 0.00040889

Frequencies -- 16.7289 25.7838 30.4820
Supporting Information: 030-Selenylated-Cluster-RuII-2HOAc-1OAc-CsBr-21-PBE.log
----
Using Gaussian 16: ES64L-G16RevA.03 25-Dec-2016
==
# pbepbe/gen/auto pseudo=read gfprint gfinput empiricaldispersion=gd3bj
scf=(direct,vshift=200,tight,maxcycle=300,yqc) opt=(gdiis,maxcycle=250)
freq=noraman
#N Geom=AllCheck Guess=TCheck SCRF=Check GenChk RPBEPBE/Gen/Auto Freq
----
Pointgroup= C1 Stoichiometry= C16H28B10BrCsO8RuSe C1[X(C16H28B10BrCsO8RuSe)]
#Atoms= 66
Charge = 0 Multiplicity = 1

SCF Energy= -6558.95930846 Predicted Change= -2.810870D-09
==
Optimization completed. {Found 2 times}

Item	Max Val.		Criteria	Pass?	RMS Val.		Criteria	Pass?
Force	0.00000	||	0.00045	[YES]	0.00000	||	0.00030	[YES]
Displ	0.00111	||	0.00180	[YES]	0.00111	||	0.00180	[YES]

Atomic Type	Coordinates (Angstroms) X	Y	Z
B	-4.352097	-0.797808	1.199254
B	-5.271080	-1.798363	0.054728
H	-6.459432	-1.723624	-0.073110
H	-4.842190	0.028091	1.916148
B	-4.431163	-3.357964	-0.094333
B	-4.222426	-2.117643	-1.345633
B	-2.944359	-1.738085	1.752248
B	-2.753246	-0.512807	0.489000
H	-2.345674	-1.634268	2.780997
H	3.741132	1.082610	0.365870
H	-4.540212	-2.344518	-2.475649
H	-4.890095	-4.425304	-0.376951

C	-2.009397	-2.047622	0.342959
C	-2.890884	-2.994543	-0.737790
B	-4.505726	-2.551671	1.486154
B	-3.005833	-3.323823	0.953310
H	-2.454780	-4.295097	1.363627
H	-5.119884	-3.022067	2.399914
B	-4.170284	-0.531711	-0.560308
H	-4.538172	0.460013	-1.118073
B	-2.660612	-1.316631	-1.083628
H	-1.902394	-1.020485	-1.949636
C	-0.492821	-2.358410	0.508749
O	0.261958	-1.463412	1.045448
O	-0.116758	-3.511482	0.220269
Cs	2.848203	-2.567897	-0.361324
C	-2.180795	-3.988947	-1.644684
H	-1.325033	-3.515438	-2.146649
H	-1.817552	-4.853141	-1.074785
H	-2.903297	-4.319254	-2.407464
Ru	0.613245	0.564574	0.649281
O	4.622856	-0.281848	-1.322845
O	2.761259	0.107153	1.464595
C	4.385212	0.923184	-1.421611
C	2.347694	0.498538	2.618299
O	4.000783	1.692317	-0.385322
O	1.124478	0.891303	2.740298
C	3.248884	0.463844	3.824528
H	2.905264	1.179392	4.586551
H	3.222877	-0.547345	4.270041
H	4.289400	0.680243	3.535383
C	4.435618	1.699418	-2.714194
H	3.405494	1.717128	-3.115662
H	4.755604	2.739988	-2.551091
H	5.093341	1.196006	-3.436609
Se	-1.678254	1.132829	0.936970
C	-2.391431	2.392715	-0.380043
C	-3.560299	3.085898	-0.028417
C	-1.708181	2.677760	-1.569337
C	-4.065554	4.065528	-0.894541
H	-4.074130	2.855252	0.910706
C	-2.217608	3.669787	-2.421998
H	-0.802288	2.119280	-1.825811
C	-3.391990	4.361700	-2.089384
H	-4.982838	4.601966	-0.630328
H	-1.693158	3.894088	-3.357193
H	-3.783724	5.132621	-2.761117
Br	0.745376	0.007931	-1.848643

O	1.117998	2.537239	0.099785
C	1.009566	3.554540	0.819908
O	0.748359	3.520682	2.125760
H	0.744220	2.556274	2.420203
C	1.104450	4.926315	0.218981
H	1.898293	4.936521	-0.542967
H	0.148490	5.148791	-0.289633
H	1.291091	5.690266	0.986825

Statistical Thermodynamic Analysis
Temperature= 298.150 Kelvin Pressure= 1.00000 Atm

==

SCF Energy= -6558.95930846 Predicted Change= -2.810870D-09
Zero-point correction (ZPE)= -6558.4993 0.45997
Internal Energy (U)= -6558.4566 0.50263
Enthalpy (H)= -6558.4557 0.50358
Gibbs Free Energy (G)= -6558.5791 0.38011
Entropy (S)= 0.0004141

Frequencies -- 20.2574 23.2424 29.1499

Supporting Information: 030-Selenylated-Cluster-RuII-2HOAc-1OAc-CsBr-22-PBE.log

----

Using Gaussian 16: ES64L-G16RevA.03 25-Dec-2016

==

# pbepbe/gen/auto pseudo=read gfprint gfinput empiricaldispersion=gd3bj
scf=(direct,vshift=200,tight,maxcycle=300,yqc) opt=(gdiis,maxcycle=250)
freq=noraman
#N Geom=AllCheck Guess=TCheck SCRF=Check GenChk RPBEPBE/Gen/Auto Freq

----

Pointgroup= C1 Stoichiometry= C16H28B10BrCsO8RuSe C1[X(C16H28B10BrCsO8RuSe)]
#Atoms= 66
Charge = 0 Multiplicity = 1

SCF Energy= -6558.94203983 Predicted Change= -3.192984D-08

==

Optimization completed on the basis of negligible forces. {Found 2 times}

Item	Max Val.		Criteria	Pass?	RMS Val.		Criteria	Pass?
Force	0.00000	||	0.00045	[YES]	0.00000	||	0.00030	[YES]
Displ	0.01391	||	0.00180	[NO]	0.01391	||	0.00180	[YES]

Atomic Type	Coordinates (Angstroms) X	Y	Z
B	2.035001	3.142166	-0.547853
B	1.624934	4.678175	-1.322841
H	2.442409	5.511313	-1.585766

H	3.147746	2.837611	-0.241022
B	-0.007754	5.140222	-0.789023
B	0.228235	4.408994	-2.393010
B	0.652840	2.628798	0.454478
B	0.897667	1.926352	-1.138921
H	0.694036	2.228319	1.681576
H	2.392806	-1.148090	2.324953
H	-0.082037	5.020839	-3.371184
H	-0.474296	6.236755	-0.704336
C	-0.664701	2.479698	-0.640360
C	-1.045838	3.908613	-1.368381
B	1.100412	4.357746	0.364268
B	-0.604728	3.896497	0.319219
H	-1.484024	4.050994	1.109687
H	1.531847	4.946426	1.312757
B	1.488031	3.155756	-2.260727
H	2.200806	2.871702	-3.178182
B	-0.218260	2.696553	-2.294660
H	-0.856940	2.097429	-3.102564
C	-1.772506	1.471891	-0.289423
O	-1.457456	0.617705	0.621448
O	-2.866512	1.527308	-0.893096
Cs	-2.284468	-2.286480	-0.611709
C	-2.488193	4.239026	-1.725403
H	-2.863950	3.571191	-2.511425
H	-3.145647	4.145024	-0.850131
H	-2.512228	5.279600	-2.083901
Ru	0.479785	0.541144	1.455757
O	0.141574	-1.552691	1.696116
O	0.245082	0.847213	3.639780
C	1.031354	-2.428055	1.904403
C	1.488228	0.646702	3.811494
O	2.283360	-2.149791	2.215774
O	2.220739	0.433530	2.747322
C	2.130330	0.646639	5.169078
H	2.565387	-0.347206	5.373200
H	2.956949	1.376497	5.190903
H	1.390580	0.892805	5.943589
C	0.729358	-3.876221	1.732290
H	0.798226	-4.070952	0.627935
H	1.458461	-4.504583	2.262385
H	-0.295832	-4.100773	2.064626
Se	1.061441	-0.068159	-0.921127
C	2.975381	-0.351322	-1.053372
C	3.413909	-1.174732	-2.099772
C	3.860437	0.167412	-0.098869

C	4.785221	-1.447255	-2.208731
H	2.689141	-1.641748	-2.773561
C	5.228238	-0.115017	-0.223677
H	3.486462	0.768500	0.733269
C	5.690731	-0.914367	-1.279926
H	5.139681	-2.094747	-3.016855
H	5.930512	0.287211	0.513755
H	6.759242	-1.135589	-1.369612
Br	0.743462	-3.547332	-1.794022
O	-5.035669	-1.143434	0.075106
C	-5.650389	-0.154226	0.482029
O	-5.205859	1.104455	0.371488
H	-4.307327	1.103177	-0.093716
C	-6.998446	-0.219624	1.167768
H	-7.340667	-1.261779	1.223773
H	-6.924594	0.205833	2.182948
H	-7.734329	0.388702	0.615613

Statistical Thermodynamic Analysis
Temperature= 298.150 Kelvin Pressure= 1.00000 Atm
==
SCF Energy= -6558.94203983 Predicted Change= -3.192984D-08
Zero-point correction (ZPE)= -6558.4827 0.45927
Internal Energy (U)= -6558.4402 0.50175
Enthalpy (H)= -6558.4393 0.50269
Gibbs Free Energy (G)= -6558.5635 0.37844
Entropy (S)= 0.00041676

Frequencies -- 11.1103 19.1225 26.5517
Supporting Information: 040-Decarboxylated-Cluster-RuII-2HOAc-1OAc-CsBr-01-PBE.log
----
Using Gaussian 16: ES64L-G16RevA.03 25-Dec-2016
==
# pbepbe/gen/auto pseudo=read gfprint gfinput empiricaldispersion=gd3bj
scf=(direct,vshift=200,tight,maxcycle=300,yqc) opt=(gdiis,maxcycle=250)
freq=noraman
#N Geom=AllCheck Guess=TCheck SCRF=Check GenChk RPBEPBE/Gen/Auto Freq
----
Pointgroup= C1 Stoichiometry= C15H28B10BrCsO6RuSe C1[X(C15H28B10BrCsO6RuSe)]
#Atoms= 63
Charge = 0 Multiplicity = 1

SCF Energy= -6370.54414679 Predicted Change= -6.022079D-08
==
Optimization completed. {Found 1 times}
Item Max Val. Criteria Pass? RMS Val. Criteria Pass?

Force 0.00007 || 0.00045 [YES] 0.00000 || 0.00030 [YES]
Displ 0.01437 || 0.00180 [NO] 0.01437 || 0.00180 [YES]

Atomic Type	Coordinates (Angstroms) X	Y	Z
B	-2.471079	1.873953	2.562875
B	-1.965479	0.965566	4.000487
H	-2.542767	1.049834	5.046724
H	-3.415969	2.610636	2.541362
B	-0.195180	0.794109	3.972268
B	-1.240340	-0.586684	3.493098
B	-0.987078	2.228014	1.598521
B	-2.024891	0.895689	1.183682
H	-0.828219	3.143563	0.849011
H	-1.192807	-1.613326	4.105045
H	0.550825	0.712628	4.904114
C	-0.327843	0.702305	1.156013
C	0.152656	-0.128322	2.632778
B	-0.932165	2.294996	3.368687
B	0.410785	1.542450	2.439378
H	1.537253	1.945999	2.368339
H	-0.765075	3.325354	3.956304
B	-2.637379	0.069775	2.600668
H	-3.692862	-0.493643	2.604505
B	-1.213630	-0.579629	1.714352
H	-1.168796	-1.631734	0.972402
C	1.361560	-1.038939	2.565290
H	1.177505	-1.941355	3.168684
H	1.535166	-1.358178	1.528244
H	2.257509	-0.534364	2.960037
Ru	-0.414216	-0.662014	-0.437950
Se	-2.517972	0.627100	-0.751381
C	-3.956061	-0.668713	-0.535468
C	-5.196338	-0.232957	-0.046281
C	-3.750800	-2.003658	-0.909638
C	-6.241507	-1.156842	0.091544
H	-5.339494	0.813341	0.242435
C	-4.807588	-2.917003	-0.774016
H	-2.776016	-2.322059	-1.292076
C	-6.048118	-2.498197	-0.271676
H	-7.207235	-0.826062	0.487305
H	-4.655115	-3.962398	-1.062170
H	-6.866679	-3.217050	-0.163096
O	1.341158	-2.083676	-0.544816
C	0.695752	-2.808734	-1.418326

C	1.372379	-3.995977	-2.055682
H	2.025113	-3.638308	-2.872202
H	2.002664	-4.534209	-1.329800
H	0.620112	-4.673320	-2.485026
O	-0.482191	-2.468470	-1.737227
Cs	3.169950	0.990912	-0.218711
Br	0.519372	0.471270	-2.523726
H	0.113521	2.622881	-2.014338
O	-0.182294	3.566522	-1.811964
C	0.784472	4.191891	-1.122097
C	0.369802	5.585855	-0.707502
H	-0.235521	6.070601	-1.488749
H	-0.254742	5.511026	0.200950
H	1.259678	6.187092	-0.474224
O	1.870159	3.683018	-0.833355
O	4.454445	-1.582036	0.849034
C	4.310694	-2.788655	1.065702
O	3.216023	-3.483668	0.727109
C	5.344752	-3.653697	1.752770
H	5.632158	-4.493114	1.097525
H	6.229856	-3.054103	2.004370
H	4.917036	-4.093385	2.669724
H	2.555096	-2.868583	0.250043

Statistical Thermodynamic Analysis
Temperature= 298.150 Kelvin Pressure= 1.00000 Atm

===

SCF Energy= -6370.54414679 Predicted Change= -6.022079D-08
Zero-point correction (ZPE)= -6370.1007 0.44334
Internal Energy (U)= -6370.0600 0.48412
Enthalpy (H)= -6370.0590 0.48506
Gibbs Free Energy (G)= -6370.1804 0.36367
Entropy (S)= 0.00040713

Frequencies -- 9.9210 25.8005 31.8357
Supporting Information: 040-Decarboxylated-Cluster-RuII-2HOAc-1OAc-CsBr-02-PBE.log

----

Using Gaussian 16: ES64L-G16RevA.03 25-Dec-2016

===

# pbepbe/gen/auto pseudo=read gfprint gfinput empiricaldispersion=gd3bj
scf=(direct,vshift=200,tight,maxcycle=300,yqc) opt=(gdiis,maxcycle=250)
freq=noraman
#N Geom=AllCheck Guess=TCheck SCRF=Check GenChk RPBEPBE/Gen/Auto Freq

----

Pointgroup= C1 Stoichiometry= C15H28B10BrCsO6RuSe C1[X(C15H28B10BrCsO6RuSe)]
#Atoms= 63

Charge = 0   Multiplicity = 1

SCF Energy= -6370.54528412   Predicted Change= -6.810581D-09

==

Optimization completed.   {Found   2   times}

Item	Max Val.		Criteria	Pass?	RMS Val.		Criteria	Pass?
Force	0.00000	||	0.00045	[YES]	0.00000	||	0.00030	[YES]
Displ	0.00153	||	0.00180	[YES]	0.00153	||	0.00180	[YES]

Atomic Type	Coordinates (Angstroms) X	Y	Z
B	-1.401571	-3.589004	0.156808
B	-0.994068	-3.984645	-1.524022
H	-1.307017	-5.023411	-2.032709
H	-2.017182	-4.317469	0.882735
B	0.570156	-3.235264	-1.918973
B	-0.959264	-2.481361	-2.491126
B	-0.087877	-2.528366	0.791284
B	-1.579591	-1.850294	0.218750
H	0.232428	-2.409352	1.936468
H	-1.158995	-2.361669	-3.664662
H	1.391501	-3.623987	-2.698918
C	-0.086124	-1.118001	-0.201860
C	0.328073	-1.590287	-1.819701
B	0.305026	-3.879358	-0.285476
B	1.116049	-2.287540	-0.479447
H	2.269007	-2.044644	-0.300638
H	0.923001	-4.834167	0.093384
B	-2.179240	-2.677866	-1.203166
H	-3.345649	-2.748020	-1.461778
B	-1.262169	-1.142244	-1.362512
H	-1.738452	0.013802	-1.687592
C	1.117128	-0.619168	-2.680375
H	0.955938	-0.877293	-3.738947
H	0.766953	0.413401	-2.520501
H	2.195785	-0.702012	-2.468107
Ru	-0.935456	0.799318	-0.203959
Se	-2.457885	-0.431361	1.347249
C	-4.205245	-0.385936	0.487404
C	-5.105717	-1.435837	0.720949
C	-4.556412	0.706104	-0.318075
C	-6.371237	-1.400907	0.119060
H	-4.814335	-2.282363	1.351077
C	-5.829530	0.733124	-0.908059
H	-3.838690	1.515089	-0.487501

C	-6.734008	-0.317231	-0.694958
H	-7.072442	-2.225262	0.285135
H	-6.111220	1.581845	-1.540255
H	-7.723432	-0.292621	-1.163125
O	0.100947	2.235399	-1.573045
C	-0.894633	3.057611	-1.464638
C	-0.842536	4.394551	-2.166544
H	-0.373192	5.140976	-1.500331
H	-0.246609	4.327835	-3.090342
H	-1.861029	4.742639	-2.396163
O	-1.892096	2.737789	-0.742508
Cs	2.903337	1.846268	-0.418449
Br	0.093674	2.022688	1.795787
H	1.163889	0.384984	2.761247
O	1.503044	-0.437458	3.261386
C	2.610399	-0.907922	2.710620
C	3.021929	-2.253725	3.250855
H	2.614551	-2.419786	4.258158
H	2.608472	-3.028206	2.579503
H	4.117445	-2.352495	3.249693
O	3.238310	-0.314470	1.808514
O	4.554278	-0.576388	-1.295906
C	4.912441	-1.672794	-0.852421
O	4.836067	-2.013486	0.442467
C	5.438138	-2.796737	-1.713482
H	4.590401	-3.456634	-1.973813
H	5.864082	-2.393074	-2.642771
H	6.180432	-3.403836	-1.173418
H	4.322776	-1.295203	0.943530

Statistical Thermodynamic Analysis
Temperature= 298.150 Kelvin   Pressure= 1.00000 Atm

===

SCF Energy=   -6370.54528412   Predicted Change= -6.810581D-09
Zero-point correction (ZPE)=   -6370.1012   0.44404
Internal Energy (U)=   -6370.0611   0.48415
Enthalpy (H)=   -6370.0601   0.48510
Gibbs Free Energy (G)=   -6370.1779   0.36733
Entropy (S)=   0.00039499

Frequencies --   17.0044   25.2179   29.7674
Supporting Information: 040-Decarboxylated-Cluster-RuII-2HOAc-1OAc-CsBr-03-PBE.log

----

Using Gaussian 16: ES64L-G16RevA.03 25-Dec-2016

===

pbepbe/gen/auto pseudo=read gfprint gfinput empiricaldispersion=gd3bj

scf=(direct,vshift=200,tight,maxcycle=300,yqc) opt=(gdiis,maxcycle=250)
freq=noraman
#N Geom=AllCheck Guess=TCheck SCRF=Check GenChk RPBEPBE/Gen/Auto Freq
----
Pointgroup= C1 Stoichiometry= C15H28B10BrCsO6RuSe C1[X(C15H28B10BrCsO6RuSe)]
#Atoms= 63
Charge = 0 Multiplicity = 1

SCF Energy= -6370.54141654 Predicted Change= -8.276415D-09
==
Optimization completed. {Found 1 times}

Item	Max Val.		Criteria	Pass?	RMS Val.		Criteria	Pass?
Force	0.00000	||	0.00045	[YES]	0.00000	||	0.00030	[YES]
Displ	0.00250	||	0.00180	[NO]	0.00250	||	0.00180	[YES]

Atomic Type	Coordinates (Angstroms) X	Y	Z
B	-3.288389	2.649689	0.277426
B	-3.360819	2.854308	2.037028
H	-4.269535	3.414184	2.580982
H	-4.136626	3.042295	-0.472407
B	-1.703800	3.039242	2.657262
B	-2.558322	1.466103	2.825454
B	-1.547608	2.657257	-0.196372
B	-2.415400	1.160337	-0.008575
H	-1.101622	2.977695	-1.256977
H	-2.768835	1.014406	3.913014
H	-1.344142	3.654301	3.618736
C	-0.826255	1.318773	0.602161
C	-0.934211	1.600125	2.342019
B	-2.123237	3.742293	1.080393
B	-0.561927	2.863330	1.261237
H	0.531174	3.352910	1.300840
H	-2.142169	4.931827	0.939331
B	-3.519865	1.205722	1.348358
H	-4.526356	0.559048	1.373222
B	-1.894960	0.452968	1.521893
H	-1.616605	-0.802196	1.622366
C	0.168976	1.069500	3.239664
H	-0.263645	0.819029	4.221031
H	0.614671	0.152154	2.821694
H	0.939352	1.841716	3.402855
Ru	-0.379207	-0.686585	0.228900
Se	-2.218158	-0.261825	-1.421667
C	-3.662182	-1.453244	-0.887228

C	-4.987028	-1.095289	-1.176912
C	-3.358961	-2.668454	-0.257663
C	-6.026951	-1.959890	-0.807938
H	-5.206047	-0.142151	-1.668839
C	-4.408510	-3.529044	0.098982
H	-2.317393	-2.929211	-0.042595
C	-5.738800	-3.176062	-0.170374
H	-7.064245	-1.678916	-1.017234
H	-4.180400	-4.479634	0.592455
H	-6.553406	-3.849218	0.115875
O	1.231761	-1.408805	1.557390
C	0.953253	-2.639932	1.258569
C	1.755887	-3.765935	1.862882
H	2.407542	-4.193466	1.081572
H	2.373490	-3.403847	2.698667
H	1.080658	-4.564331	2.210414
O	0.011190	-2.878333	0.434605
Cs	2.996900	1.174290	0.771607
Br	1.260896	-0.736214	-1.732996
H	1.020841	1.373599	-2.615966
O	0.788087	2.245239	-3.055728
C	1.424848	3.237453	-2.410012
C	1.027215	4.597759	-2.933750
H	0.087624	4.900259	-2.437010
H	1.805394	5.334658	-2.690254
H	0.839118	4.570610	-4.017790
O	2.214530	3.059407	-1.480540
O	4.708304	-1.272127	0.078569
C	4.591043	-2.455464	-0.240283
O	3.548181	-2.955725	-0.927261
C	5.590387	-3.534619	0.115861
H	5.780088	-4.192288	-0.747130
H	6.526164	-3.076586	0.464131
H	5.175175	-4.164479	0.922776
H	2.894323	-2.216576	-1.124867

Statistical Thermodynamic Analysis
Temperature= 298.150 Kelvin   Pressure= 1.00000 Atm

===

SCF Energy=   -6370.54141654   Predicted Change= -8.276415D-09
Zero-point correction (ZPE)=   -6370.0978   0.44352
Internal Energy (U)=   -6370.0573   0.48410
Enthalpy (H)=   -6370.0563   0.48504
Gibbs Free Energy (G)=   -6370.1761   0.36527
Entropy (S)=   0.00040173

Frequencies -- 14.7364 18.8937 30.9254

Supporting Information: 040-Decarboxylated-Cluster-RuII-2HOAc-1OAc-CsBr-04-PBE.log

----

Using Gaussian 16: ES64L-G16RevA.03 25-Dec-2016

==

# pbepbe/gen/auto pseudo=read gfprint gfinput empiricaldispersion=gd3bj
scf=(direct,vshift=200,tight,maxcycle=300,yqc) opt=(gdiis,maxcycle=250)
freq=noraman
#N Geom=AllCheck Guess=TCheck SCRF=Check GenChk RPBEPBE/Gen/Auto Freq

----

Pointgroup= C1 Stoichiometry= C15H28B10BrCsO6RuSe C1[X(C15H28B10BrCsO6RuSe)]
#Atoms= 63
Charge = 0 Multiplicity = 1

SCF Energy= -6370.54860748 Predicted Change= -3.409426D-08

==

Optimization completed. {Found 1 times}

Item	Max Val.		Criteria	Pass?	RMS Val.		Criteria	Pass?
Force	0.00001	||	0.00045	[YES]	0.00000	||	0.00030	[YES]
Displ	0.00365	||	0.00180	[NO]	0.00365	||	0.00180	[YES]

Atomic Type	Coordinates (Angstroms) X	Y	Z
B	3.576699	-2.072925	-1.939057
B	4.493347	-2.996939	-0.721971
H	5.523400	-3.561091	-0.962685
H	3.915246	-1.950112	-3.082546
B	3.328303	-3.689599	0.426846
B	4.231949	-2.227512	0.860480
B	1.852823	-2.222743	-1.518267
B	2.734982	-0.777639	-1.065309
H	0.956480	-2.167635	-2.305271
H	4.956361	-2.221961	1.813448
H	3.443799	-4.680968	1.087924
C	1.635255	-1.462315	0.025928
C	2.544482	-2.354322	1.161577
B	2.912551	-3.609794	-1.290553
B	1.687656	-3.185692	-0.053086
H	0.718819	-3.811861	0.263448
H	2.784058	-4.602055	-1.950909
B	4.384176	-1.213448	-0.595624
H	5.317819	-0.476327	-0.740284
B	3.149857	-0.851743	0.635410
H	3.125048	0.076579	1.378969
C	2.016201	-2.463739	2.582392

H	2.371719	-1.620750	3.193649
H	0.915934	-2.462646	2.576529
H	2.367492	-3.405094	3.032648
Ru	0.140390	-0.033687	0.199796
Se	1.693903	0.911978	-1.534369
C	2.764181	2.243239	-0.608061
C	4.118272	2.394762	-0.944763
C	2.159277	3.073835	0.345127
C	4.880937	3.377226	-0.298822
H	4.578601	1.741105	-1.691423
C	2.931666	4.058362	0.978502
H	1.101828	2.941150	0.592478
C	4.289704	4.209849	0.663178
H	5.941206	3.488175	-0.548838
H	2.464518	4.706689	1.727473
H	4.888605	4.975979	1.166489
O	-0.891055	-0.721687	2.091287
C	-0.059239	0.027997	2.743826
C	-0.183943	0.197067	4.236818
H	-0.899864	1.008730	4.461580
H	-0.551646	-0.731204	4.702965
H	0.788542	0.477858	4.668649
O	0.853799	0.635741	2.093922
Cs	-3.850998	-0.325234	1.405267
O	-0.905668	-0.747594	-1.524613
C	-1.579536	-1.796791	-1.435237
O	-1.669985	-2.467123	-0.277825
H	-0.988993	-2.000742	0.324264
C	-2.334688	-2.383609	-2.586069
H	-2.073882	-3.448617	-2.700922
H	-3.420434	-2.306045	-2.404230
H	-2.081309	-1.833792	-3.502942
Br	-1.519691	2.079032	0.231462
O	-4.265468	0.048955	-1.576926
C	-3.912384	0.690305	-2.576717
O	-2.930690	1.588694	-2.598165
H	-2.494210	1.661557	-1.681213
C	-4.545698	0.512879	-3.941491
H	-5.483581	-0.052548	-3.851930
H	-4.727386	1.489518	-4.417381
H	-3.850049	-0.041327	-4.596182

Statistical Thermodynamic Analysis
Temperature= 298.150 Kelvin   Pressure= 1.00000 Atm

==

SCF Energy=   -6370.54860748   Predicted Change= -3.409426D-08

Zero-point correction (ZPE)= -6370.1036 0.44495
Internal Energy (U)= -6370.0639 0.48467
Enthalpy (H)= -6370.0629 0.48561
Gibbs Free Energy (G)= -6370.1790 0.36956
Entropy (S)= 0.00038923

Frequencies -- 19.7426 25.7618 35.4021

Supporting Information: 040-Decarboxylated-Cluster-RuII-2HOAc-1OAc-CsBr-05-PBE.log

----

Using Gaussian 16: ES64L-G16RevA.03 25-Dec-2016

===

# pbepbe/gen/auto pseudo=read gfprint gfinput empiricaldispersion=gd3bj
scf=(direct,vshift=200,tight,maxcycle=300,yqc) opt=(gdiis,maxcycle=250)
freq=noraman
#N Geom=AllCheck Guess=TCheck SCRF=Check GenChk RPBEPBE/Gen/Auto Freq

----

Pointgroup= C1 Stoichiometry= C15H28B10BrCsO6RuSe C1[X(C15H28B10BrCsO6RuSe)]
#Atoms= 63
Charge = 0 Multiplicity = 1

SCF Energy= -6370.54528410 Predicted Change= -5.058582D-09

===

Optimization completed. {Found 1 times}

Item	Max Val.		Criteria	Pass?	RMS Val.		Criteria	Pass?
Force	0.00000	||	0.00045	[YES]	0.00000	||	0.00030	[YES]
Displ	0.00217	||	0.00180	[NO]	0.00217	||	0.00180	[YES]

Atomic Type	Coordinates (Angstroms) X	Y	Z
B	1.401679	3.589049	0.156842
B	0.994177	3.984705	-1.524004
H	1.307182	5.023472	-2.032656
H	2.017281	4.317536	0.882754
B	-0.570081	3.235386	-1.918929
B	0.959306	2.481425	-2.491101
B	0.087938	2.528444	0.791298
B	1.579640	1.850343	0.218771
H	-0.232344	2.409429	1.936488
H	1.159048	2.361748	-3.664636
H	-1.391391	3.624157	-2.698888
C	0.086140	1.118105	-0.201881
C	-0.328041	1.590401	-1.819654
B	-0.304921	3.879471	-0.285439
B	-1.115998	2.287688	-0.479414
H	-2.268962	2.044837	-0.300583

H	-0.922849	4.834305	0.093433
B	2.179315	2.677902	-1.203141
H	3.345725	2.748023	-1.461756
B	1.262222	1.142301	-1.362490
H	1.738472	-0.013764	-1.687587
C	-1.117107	0.619301	-2.680351
H	-0.956264	0.877735	-3.738900
H	-0.766670	-0.413231	-2.520815
H	-2.195725	0.701850	-2.467777
Ru	0.935401	-0.799272	-0.204031
Se	2.457809	0.431308	1.347242
C	4.205204	0.385848	0.487466
C	5.105706	1.435708	0.721075
C	4.556360	-0.706183	-0.318032
C	6.371248	1.400747	0.119233
H	4.814332	2.282228	1.351215
C	5.829499	-0.733235	-0.907967
H	3.838609	-1.515132	-0.487514
C	6.734009	0.317080	-0.694801
H	7.072477	2.225071	0.285360
H	6.111181	-1.581946	-1.540179
H	7.723449	0.292446	-1.162932
O	-0.100990	-2.235349	-1.573213
C	0.894576	-3.057557	-1.464712
C	0.842535	-4.394553	-2.166519
H	0.374995	-5.141386	-1.499497
H	0.245121	-4.328415	-3.089389
H	1.860985	-4.741747	-2.397717
O	1.892015	-2.737727	-0.742547
Cs	-2.903461	-1.846307	-0.418424
Br	-0.093697	-2.022828	1.795626
H	-1.163821	-0.385104	2.761136
O	-1.502876	0.437392	3.261248
C	-2.610264	0.907868	2.710555
C	-3.021682	2.253740	3.250703
H	-2.613760	2.420120	4.257730
H	-2.608782	3.028127	2.578901
H	-4.117220	2.352315	3.250149
O	-3.238311	0.314363	1.808580
O	-4.553950	0.576661	-1.295901
C	-4.912263	1.672957	-0.852276
O	-4.836028	2.013454	0.442679
C	-5.437997	2.796991	-1.713193
H	-4.590293	3.456984	-1.973387
H	-5.863864	2.393439	-2.642567
H	-6.180362	3.403950	-1.173074

H -4.322737 1.295136 0.943675

Statistical Thermodynamic Analysis
Temperature= 298.150 Kelvin Pressure= 1.00000 Atm

===

SCF Energy= -6370.54528410 Predicted Change= -5.058582D-09
Zero-point correction (ZPE)= -6370.1012 0.44404
Internal Energy (U)= -6370.0611 0.48415
Enthalpy (H)= -6370.0601 0.48510
Gibbs Free Energy (G)= -6370.1779 0.36733
Entropy (S)= 0.00039499

Frequencies -- 17.0107 25.2162 29.7648
Supporting Information: 040-Decarboxylated-Cluster-RuII-2HOAc-1OAc-CsBr-06-PBE.log

----

Using Gaussian 16: ES64L-G16RevA.03 25-Dec-2016

===

# pbepbe/gen/auto pseudo=read gfprint gfinput empiricaldispersion=gd3bj
scf=(direct,vshift=200,tight,maxcycle=300,yqc) opt=(gdiis,maxcycle=250)
freq=noraman
#N Geom=AllCheck Guess=TCheck SCRF=Check GenChk RPBEPBE/Gen/Auto Freq

----

Pointgroup= C1 Stoichiometry= C15H28B10BrCsO6RuSe C1[X(C15H28B10BrCsO6RuSe)]
#Atoms= 63
Charge = 0 Multiplicity = 1

SCF Energy= -6370.53723903 Predicted Change= -1.043960D-08

===

Optimization completed on the basis of negligible forces. {Found 2 times}

Item	Max Val.	Criteria	Pass?	RMS Val.	Criteria	Pass?
Force	0.00000 ||	0.00045	[YES]	0.00000 ||	0.00030	[YES]
Displ	0.00263 ||	0.00180	[NO]	0.00263 ||	0.00180	[YES]

Atomic Type	Coordinates (Angstroms) X	Y	Z
B	-2.894681	3.248292	-1.195656
B	-3.271305	4.159860	0.289153
H	-4.037842	5.081078	0.319034
H	-3.372661	3.489597	-2.268300
B	-1.821145	4.155455	1.314544
B	-3.149358	3.037494	1.666041
B	-1.204135	2.700067	-1.057651
B	-2.511211	1.591074	-0.691211
H	-0.473892	2.498036	-1.983551
H	-3.721751	3.084566	2.716188

H	-1.487794	4.966421	2.129558
C	-1.124174	1.611721	0.283462
C	-1.514207	2.508227	1.686863
B	-1.644763	4.292275	-0.442089
B	-0.547935	3.229052	0.489028
H	0.608864	3.410931	0.732877
H	-1.224873	5.292101	-0.953243
B	-3.818090	2.463400	0.119146
H	-4.967634	2.139310	0.024673
B	-2.684225	1.451432	1.046988
H	-2.932487	0.440970	1.622762
C	-0.830145	2.125737	2.988926
H	-1.420790	1.371229	3.529428
H	0.169367	1.714305	2.780865
H	-0.721019	3.018713	3.623799
Ru	-0.276908	-0.280831	-0.040230
Se	-2.260574	-0.219242	-1.588454
C	-3.665299	-1.230760	-0.703606
C	-4.999430	-0.820864	-0.852774
C	-3.338860	-2.384373	0.021919
C	-6.018597	-1.565635	-0.243832
H	-5.240582	0.079987	-1.424705
C	-4.369525	-3.124064	0.620416
H	-2.292970	-2.692540	0.118676
C	-5.705498	-2.717061	0.494003
H	-7.059276	-1.240952	-0.346647
H	-4.119736	-4.024117	1.192308
H	-6.503965	-3.296425	0.969240
O	1.161253	-0.449645	1.682375
C	0.183631	-0.941397	2.373329
C	0.405476	-1.473694	3.765371
H	0.663219	-2.547083	3.713014
H	1.230650	-0.937180	4.260160
H	-0.517157	-1.379896	4.358434
O	-0.974315	-0.983786	1.837510
Cs	3.541139	-1.773676	0.266034
O	0.781604	0.240387	-1.840160
C	1.722774	1.053791	-1.741456
O	2.022598	1.584505	-0.544223
H	1.241881	1.239202	0.068025
C	2.585768	1.482787	-2.885027
H	2.460153	2.566328	-3.057082
H	3.649260	1.301932	-2.648857
H	2.298513	0.931981	-3.790860
Br	0.328142	-2.794486	-0.639361
O	5.165359	0.764084	-0.386314

C	5.384955	1.956051	-0.146212
O	4.419723	2.881909	-0.038545
H	3.536878	2.430953	-0.180422
C	6.760543	2.546958	0.057259
H	6.828150	3.008026	1.057086
H	7.525873	1.766656	-0.049386
H	6.937494	3.349683	-0.678133

Statistical Thermodynamic Analysis
Temperature= 298.150 Kelvin Pressure= 1.00000 Atm

===

SCF Energy= -6370.53723903 Predicted Change= -1.043960D-08
Zero-point correction (ZPE)= -6370.0927 0.44451
Internal Energy (U)= -6370.0529 0.48432
Enthalpy (H)= -6370.0519 0.48526
Gibbs Free Energy (G)= -6370.1693 0.36791
Entropy (S)= 0.00039361

Frequencies -- 16.8486 21.9128 27.2874

Supporting Information: 040-Decarboxylated-Cluster-RuII-2HOAc-1OAc-CsBr-CO2-01-PBE.log

----

Using Gaussian 16: ES64L-G16RevA.03 25-Dec-2016

===

# pbepbe/gen/auto pseudo=read gfprint gfinput empiricaldispersion=gd3bj
scf=(direct,vshift=200,tight,maxcycle=300,yqc) opt=(gdiis,maxcycle=250)
freq=noraman
#N Geom=AllCheck Guess=TCheck SCRF=Check GenChk RPBEPBE/Gen/Auto Freq

----

Pointgroup= C1 Stoichiometry= C16H28B10BrCsO8RuSe C1[X(C16H28B10BrCsO8RuSe)]
#Atoms= 66
Charge = 0 Multiplicity = 1

SCF Energy= -6558.94964695 Predicted Change= -2.569899D-06

===

Optimization completed. {Found 1 times}

Item	Max Val.	Criteria	Pass?	RMS Val.	Criteria	Pass?
Force	0.00003 ||	0.00045	[YES]	0.00000 ||	0.00030	[YES]
Displ	0.08529 ||	0.00180	[NO]	0.08529 ||	0.00180	[NO]

Atomic Type	Coordinates (Angstroms) X	Y	Z
B	1.973588	-2.537506	2.246071
B	1.601694	-1.771107	3.802737
H	2.077953	-2.152734	4.833502
H	2.722988	-3.464795	2.125727

B	-0.075168	-1.183591	3.761565
B	1.284511	-0.030703	3.544087
B	0.511674	-2.373875	1.201315
B	1.851976	-1.278231	1.038535
H	0.188680	-3.101869	0.313175
H	1.437611	0.870114	4.315914
H	-0.841271	-1.075398	4.674146
C	0.251451	-0.686354	1.000549
C	-0.118714	-0.005768	2.586384
B	0.331695	-2.702379	2.933240
B	-0.738366	-1.514148	2.111520
H	-1.920668	-1.616719	1.951049
H	-0.106943	-3.742797	3.333285
B	2.547675	-0.852671	2.588280
H	3.702049	-0.563207	2.711067
B	1.368385	0.244982	1.789262
H	1.620941	1.381406	1.235384
C	-3.306707	-3.855748	0.865795
O	-4.074205	-2.955145	0.818110
O	-2.572048	-4.771014	0.961914
C	-1.090429	1.155414	2.633438
H	-0.677671	1.968930	3.249754
H	-1.251782	1.552501	1.622033
H	-2.051016	0.836684	3.067896
Ru	0.755440	0.863226	-0.329583
Se	2.515557	-0.830354	-0.810591
C	4.201231	0.025889	-0.342546
C	5.272450	-0.772633	0.084430
C	4.337671	1.414282	-0.476235
C	6.494499	-0.165499	0.405067
H	5.148249	-1.855775	0.184395
C	5.568811	2.008866	-0.159561
H	3.489884	2.018645	-0.813842
C	6.643437	1.224333	0.283539
H	7.329623	-0.781622	0.753849
H	5.683604	3.093312	-0.259209
H	7.599374	1.695302	0.534460
O	-0.593164	2.661230	-0.208695
C	0.253259	3.347512	-0.925367
C	-0.085803	4.753514	-1.350912
H	-0.706236	4.708228	-2.264015
H	-0.662585	5.279161	-0.573534
H	0.833821	5.308543	-1.586939
O	1.335350	2.790469	-1.280615
Cs	-3.143628	-0.065445	-0.553525
Br	-0.283483	0.376398	-2.610116

H	-0.256200	-1.847436	-2.542742
O	-0.116452	-2.848919	-2.562638
C	-1.145917	-3.457108	-1.961506
C	-0.956185	-4.954558	-1.885242
H	-0.338129	-5.321821	-2.717627
H	-0.439057	-5.192670	-0.938783
H	-1.933729	-5.457003	-1.871014
O	-2.125595	-2.862863	-1.495629
O	-3.860054	2.580594	0.821357
C	-3.518473	3.675745	1.277470
O	-2.278791	4.173395	1.177932
C	-4.449911	4.609794	2.019330
H	-4.520449	5.573047	1.486151
H	-5.447041	4.157447	2.105383
H	-4.046675	4.826516	3.022828
H	-1.694857	3.527707	0.642604

Statistical Thermodynamic Analysis
Temperature= 298.150 Kelvin   Pressure= 1.00000 Atm

==

SCF Energy=   -6558.94964695   Predicted Change= -2.569899D-06
Zero-point correction (ZPE)=   -6558.4942   0.45539
Internal Energy (U)=   -6558.4492   0.50044
Enthalpy (H)=   -6558.4482   0.50138
Gibbs Free Energy (G)=   -6558.5827   0.36692
Entropy (S)=   0.000451

Frequencies --   1.0832   20.1892   28.9152

Supporting Information: 040-Decarboxylated-Cluster-RuII-2HOAc-1OAc-CsBr-CO2-02-PBE.log

----

Using Gaussian 16: ES64L-G16RevA.03 25-Dec-2016

==

\# pbepbe/gen/auto pseudo=read gfprint gfinput empiricaldispersion=gd3bj
scf=(direct,vshift=200,tight,maxcycle=300,yqc) opt=(gdiis,maxcycle=250)
freq=noraman
\#N Geom=AllCheck Guess=TCheck SCRF=Check GenChk RPBEPBE/Gen/Auto Freq

----

Pointgroup= C1   Stoichiometry= C16H28B10BrCsO8RuSe   C1[X(C16H28B10BrCsO8RuSe)]
\#Atoms= 66
Charge = 0   Multiplicity = 1

SCF Energy= -6558.94916602   Predicted Change= -4.632672D-08

==

Optimization completed.   {Found   1   times}

Item	Max Val.	Criteria	Pass?	RMS Val.	Criteria	Pass?
Force	0.00010 ||	0.00045	[YES]	0.00000 ||	0.00030	[YES]

Displ 0.00335 || 0.00180 [NO] 0.00335 || 0.00180 [YES]

Atomic Type	Coordinates (Angstroms) X	Y	Z
B	-0.831615	-3.537421	-0.255915
B	-0.576213	-3.708823	-2.002869
H	-0.713561	-4.754975	-2.570148
H	-1.163542	-4.440415	0.458473
B	0.715473	-2.590640	-2.501745
B	-1.007150	-2.178097	-2.814175
B	0.288352	-2.249242	0.327887
B	-1.383595	-1.897183	0.000529
H	0.730790	-2.147988	1.432784
H	-1.388029	-2.023524	-3.937627
H	1.494130	-2.723886	-3.400063
C	-0.159957	-0.810528	-0.499177
C	0.128013	-1.061837	-2.217602
B	0.819759	-3.387901	-0.919607
B	1.222786	-1.636259	-1.045823
H	2.311159	-1.140360	-0.985474
H	1.666029	-4.210006	-0.712924
B	-1.966689	-2.737936	-1.420269
H	-3.112567	-3.058157	-1.547306
B	-1.436222	-1.023318	-1.531077
H	-2.181806	0.016500	-1.689551
C	4.231631	-3.440961	-0.148372
O	4.529673	-3.167367	-1.257987
O	3.942978	-3.738222	0.956563
C	0.542549	0.123505	-3.070649
H	0.191409	-0.043779	-4.101000
H	0.082467	1.054718	-2.701780
H	1.641265	0.211932	-3.102455
Ru	-1.398187	0.852937	-0.217259
Se	-2.424728	-0.813475	1.342268
C	-4.225088	-1.109899	0.662133
C	-4.850081	-2.343292	0.897563
C	-4.887256	-0.078006	-0.017218
C	-6.152415	-2.553557	0.423458
H	-4.318042	-3.138730	1.429298
C	-6.193955	-0.298103	-0.479373
H	-4.381267	0.877836	-0.188253
C	-6.824679	-1.532145	-0.264596
H	-6.639800	-3.519647	0.590509
H	-6.717843	0.502298	-1.012378
H	-7.842054	-1.699448	-0.632898

O	-0.779674	2.565067	-1.484012
C	-1.904243	3.164905	-1.242226
C	-2.150195	4.545905	-1.799842
H	-1.768650	5.299540	-1.087569
H	-1.625966	4.678431	-2.759264
H	-3.229575	4.717340	-1.928364
O	-2.770973	2.588699	-0.509680
Cs	2.193492	2.265282	-0.503505
Br	-0.517019	2.136851	1.810631
H	0.702690	0.604055	2.754631
O	1.169659	-0.127788	3.290634
C	2.347930	-0.414877	2.761229
C	3.050444	-1.563308	3.437481
H	2.639185	-1.749405	4.439274
H	2.911433	-2.467906	2.819807
H	4.132278	-1.366129	3.490443
O	2.823773	0.193241	1.779253
O	4.924461	0.899521	-0.734449
C	5.525259	-0.082922	-0.292676
O	5.104087	-0.799630	0.764008
C	6.799334	-0.635418	-0.888630
H	6.558280	-1.554650	-1.450292
H	7.242266	0.100602	-1.573419
H	7.516196	-0.908715	-0.098040
H	4.237627	-0.397753	1.118216

Statistical Thermodynamic Analysis
Temperature= 298.150 Kelvin   Pressure= 1.00000 Atm

===

SCF Energy=   -6558.94916602   Predicted Change= -4.632672D-08
Zero-point correction (ZPE)=   -6558.4931   0.45605
Internal Energy (U)=   -6558.4487   0.50046
Enthalpy (H)=   -6558.4477   0.50140
Gibbs Free Energy (G)=   -6558.5769   0.37218
Entropy (S)=   0.00043341

Frequencies --   10.9828   17.5998   29.4845
Supporting Information: 040-Decarboxylated-Cluster-RuII-2HOAc-1OAc-CsBr-CO2-03-PBE.log

----

Using Gaussian 16: ES64L-G16RevA.03 25-Dec-2016

===

# pbepbe/gen/auto pseudo=read gfprint gfinput empiricaldispersion=gd3bj
scf=(direct,vshift=200,tight,maxcycle=300,yqc) opt=(gdiis,maxcycle=250)
freq=noraman
#N Geom=AllCheck Guess=TCheck SCRF=Check GenChk RPBEPBE/Gen/Auto Freq

----

Pointgroup= C1 Stoichiometry= C16H28B10BrCsO8RuSe C1[X(C16H28B10BrCsO8RuSe)]
#Atoms= 66
Charge = 0 Multiplicity = 1

SCF Energy= -6558.94690447 Predicted Change= -3.571082D-08

===

Optimization completed. {Found 1 times}

Item	Max Val.	Criteria	Pass?	RMS Val.	Criteria	Pass?
Force	0.00000 ||	0.00045	[YES]	0.00000 ||	0.00030	[YES]
Displ	0.00442 ||	0.00180	[NO]	0.00442 ||	0.00180	[YES]

Atomic Type	Coordinates (Angstroms) X	Y	Z
B	-2.637357	-3.214929	-0.245035
B	-2.622154	-3.464676	-2.000421
H	-3.335962	-4.260291	-2.541267
H	-3.359744	-3.808677	0.504279
B	-0.967208	-3.204708	-2.597848
B	-2.212575	-1.925516	-2.811326
B	-0.967808	-2.740584	0.247643
B	-2.205598	-1.539512	0.016207
H	-0.471618	-2.909638	1.320617
H	-2.519387	-1.568589	-3.911074
H	-0.437493	-3.716727	-3.540840
C	-0.623284	-1.272105	-0.573556
C	-0.621687	-1.604967	-2.306187
B	-1.206286	-3.965865	-1.010065
B	0.059099	-2.699944	-1.194042
H	1.243905	-2.865029	-1.221631
H	-0.903183	-5.112598	-0.841747
B	-3.233810	-1.908684	-1.351264
H	-4.377371	-1.560231	-1.401220
B	-1.871332	-0.746289	-1.523630
H	-1.942275	0.535444	-1.650443
C	3.516662	-4.119696	-0.236900
O	3.989742	-3.251530	-0.888989
O	3.073078	-5.026708	0.368670
C	0.315693	-0.813741	-3.200213
H	-0.147696	-0.708360	-4.193749
H	0.488431	0.197419	-2.797227
H	1.269462	-1.351649	-3.331513
Ru	-0.745189	0.787630	-0.242618
Se	-2.425292	-0.091078	1.398926
C	-4.130993	0.650222	0.823830
C	-5.312199	-0.050462	1.108465

C	-4.161004	1.889926	0.170248
C	-6.542685	0.490222	0.710138
H	-5.270444	-1.017751	1.619382
C	-5.399860	2.424358	-0.215712
H	-3.226850	2.420997	-0.040435
C	-6.587400	1.726682	0.048500
H	-7.467089	-0.059330	0.915542
H	-5.432113	3.391661	-0.728003
H	-7.550224	2.146355	-0.260518
O	0.628805	1.896421	-1.570138
C	0.019890	3.009955	-1.303309
C	0.495567	4.300749	-1.923433
H	1.003541	4.898589	-1.147175
H	1.193861	4.104915	-2.751138
H	-0.366296	4.883845	-2.285653
O	-0.965593	2.997292	-0.495514
Cs	3.037357	-0.085719	-0.694655
Br	0.787893	1.327309	1.732541
H	1.098299	-0.727895	2.664773
O	1.092247	-1.601789	3.161322
C	1.920190	-2.461381	2.551426
C	1.869762	-3.836887	3.172176
H	1.133373	-4.440652	2.612267
H	2.847626	-4.329682	3.078062
H	1.551393	-3.790709	4.223644
O	2.620450	-2.167225	1.575958
O	3.994522	2.756102	-0.020448
C	3.550590	3.869127	0.261226
O	2.391201	4.079768	0.911171
C	4.227099	5.172417	-0.104266
H	4.206037	5.875279	0.743572
H	5.261660	4.980222	-0.419684
H	3.678502	5.647227	-0.937224
H	1.960454	3.193870	1.115250

Statistical Thermodynamic Analysis
Temperature= 298.150 Kelvin   Pressure= 1.00000 Atm

==

SCF Energy=   -6558.94690447   Predicted Change= -3.571082D-08
Zero-point correction (ZPE)=   -6558.4911   0.45580
Internal Energy (U)=   -6558.4463   0.50055
Enthalpy (H)=   -6558.4454   0.50149
Gibbs Free Energy (G)=   -6558.5757   0.37110
Entropy (S)=   0.00043734

Frequencies --   13.8950   19.2279   22.4719

Supporting Information: 042-Decarboxylated-Cluster-RuIV-H-1HOAc-2OAc-CsBr-01-PBE.log

---

Using Gaussian 16: ES64L-G16RevA.03 25-Dec-2016

==

# pbepbe/gen/auto pseudo=read gfprint gfinput empiricaldispersion=gd3bj
scf=(direct,vshift=200,tight,maxcycle=300,yqc) opt=(gdiis,maxcycle=250)
freq=noraman
#N Geom=AllCheck Guess=TCheck SCRF=Check GenChk RPBEPBE/Gen/Auto Freq

---

Pointgroup= C1 Stoichiometry= C15H28B10BrCsO6RuSe C1[X(C15H28B10BrCsO6RuSe)]
#Atoms= 63
Charge = 0 Multiplicity = 1

SCF Energy= -6370.54013916 Predicted Change= -7.457943D-08

==

Optimization completed. {Found 1 times}

Item	Max Val.	Criteria	Pass?	RMS Val.	Criteria	Pass?
Force	0.00000 ||	0.00045	[YES]	0.00000 ||	0.00030	[YES]
Displ	0.01218 ||	0.00180	[NO]	0.01218 ||	0.00180	[YES]

Atomic Type	Coordinates (Angstroms) X	Y	Z
B	-2.582025	3.438632	-1.028543
B	-3.107835	4.220289	0.483951
H	-3.826863	5.178376	0.513141
H	-2.900728	3.809170	-2.122685
B	-1.805145	4.025004	1.679852
B	-3.226102	2.966145	1.741852
B	-0.953941	2.777701	-0.734922
B	-2.351124	1.722455	-0.653236
H	-0.119588	2.595667	-1.567927
H	-3.928637	2.948150	2.709967
H	-1.549977	4.728588	2.613174
C	-1.135944	1.580262	0.500305
C	-1.633466	2.333861	1.913369
B	-1.394384	4.324883	-0.016545
B	-0.475776	3.117359	0.926381
H	0.650275	3.166685	1.318016
H	-0.864449	5.341296	-0.363888
B	-3.714453	2.588873	0.067354
H	-4.856270	2.350646	-0.203939
B	-2.776506	1.421770	1.023761
H	-3.139252	0.373875	1.450092
C	-1.137583	1.779243	3.238635
H	-1.817354	0.999666	3.611999

H	-0.132391	1.348636	3.110701
H	-1.081774	2.594293	3.976345
Ru	-0.250180	-0.314207	0.151754
Se	-2.025999	0.002192	-1.691606
C	-3.595932	-0.988027	-1.112024
C	-4.860038	-0.459809	-1.421260
C	-3.467202	-2.222596	-0.462268
C	-6.009982	-1.170523	-1.053242
H	-4.948299	0.503453	-1.932065
C	-4.627318	-2.923216	-0.100231
H	-2.475777	-2.627119	-0.238102
C	-5.895754	-2.401432	-0.390102
H	-6.996455	-0.755810	-1.284359
H	-4.531420	-3.885099	0.414243
H	-6.796213	-2.952831	-0.100709
O	0.883218	-0.910806	1.963111
C	-0.217252	-1.340806	2.482524
C	-0.223913	-2.112603	3.771655
H	-0.127421	-3.189395	3.544327
H	0.620585	-1.811213	4.410693
H	-1.176822	-1.960443	4.301255
O	-1.299800	-1.107591	1.842077
Cs	3.449845	-1.592041	0.386949
O	0.920160	0.168711	-1.558531
C	1.936135	0.954595	-1.470622
O	2.378515	1.419146	-0.371337
H	0.707754	0.893542	0.500878
C	2.628393	1.293537	-2.777403
H	2.596600	2.386227	-2.927420
H	3.690903	0.999288	-2.722544
H	2.141639	0.790393	-3.623953
Br	0.173609	-2.796580	-0.568840
O	5.460122	0.519243	-0.542644
C	5.656877	1.742476	-0.530793
O	4.690369	2.658200	-0.466309
H	3.775151	2.182969	-0.431974
C	7.035947	2.366197	-0.594662
H	7.201864	3.007516	0.287314
H	7.804313	1.582373	-0.639290
H	7.112688	3.016527	-1.482405

Statistical Thermodynamic Analysis
Temperature= 298.150 Kelvin   Pressure= 1.00000 Atm

==

SCF Energy=   -6370.54013916   Predicted Change= -7.457943D-08
Zero-point correction (ZPE)=   -6370.0970   0.44313

Internal Energy (U)= -6370.0575 0.48259
Enthalpy (H)= -6370.0566 0.48353
Gibbs Free Energy (G)= -6370.1725 0.36754
Entropy (S)= 0.00038906

Frequencies -- 17.3281 24.0723 28.6137
Supporting Information: 050-Product-Catalyst-Complex-1HOAc-2OAc-CsBr-01-PBE.log
----
Using Gaussian 16: ES64L-G16RevA.03 25-Dec-2016
==
# pbepbe/gen/auto pseudo=read gfprint gfinput empiricaldispersion=gd3bj
scf=(direct,vshift=200,tight,maxcycle=300,yqc) opt=(gdiis,maxcycle=250)
freq=noraman
#N Geom=AllCheck Guess=TCheck SCRF=Check GenChk RPBEPBE/Gen/Auto Freq
----
Pointgroup= C1 Stoichiometry= C15H28B10BrCsO6RuSe C1[X(C15H28B10BrCsO6RuSe)]
#Atoms= 63
Charge = 0 Multiplicity = 1

SCF Energy= -6370.56267826 Predicted Change= -3.877580D-08
==
Optimization completed. {Found 1 times}

Item	Max Val.		Criteria	Pass?	RMS Val.		Criteria	Pass?
Force	0.00001	||	0.00045	[YES]	0.00000	||	0.00030	[YES]
Displ	0.00209	||	0.00180	[NO]	0.00209	||	0.00180	[YES]

Atomic Type	Coordinates (Angstroms) X	Y	Z
B	4.366333	-1.196360	-0.477459
B	4.446305	-2.176232	1.017144
H	5.390331	-2.161050	1.753740
H	5.235216	-0.447926	-0.821097
B	3.541663	-3.681696	0.731343
B	2.782253	-2.352454	1.647688
B	3.413369	-2.109914	-1.671066
B	2.661199	-0.812923	-0.730890
H	3.437926	-2.047685	-2.864729
H	2.451872	-2.545343	2.781207
H	3.705565	-4.744359	1.254097
C	1.886768	-2.297219	-0.919746
C	1.933717	-3.198767	0.432575
B	4.521627	-2.979744	-0.583675
B	2.923720	-3.645645	-0.928016
H	2.581042	-4.608750	-1.543442
H	5.498453	-3.538119	-0.992396

B	3.306905	-0.818676	0.924392
H	3.410480	0.192325	1.551150
B	1.671319	-1.422410	0.595396
H	0.428793	-1.501587	1.070816
C	0.753116	-4.103140	0.751664
H	0.862516	-4.471240	1.783367
H	-0.205982	-3.561318	0.672765
H	0.732676	-4.971028	0.074529
Ru	0.015824	0.034449	0.522813
Se	1.535404	0.729031	-1.373387
C	2.715577	2.138532	-0.733857
C	3.930381	2.337655	-1.407865
C	2.332853	2.960982	0.332423
C	4.785443	3.366461	-0.990441
H	4.212914	1.688470	-2.242563
C	3.195882	3.990626	0.736268
H	1.383162	2.788177	0.843062
C	4.418930	4.193806	0.081528
H	5.739580	3.518530	-1.505408
H	2.906063	4.634429	1.573286
H	5.088472	4.997047	0.405928
O	-1.111830	-0.151996	2.447846
C	-0.217037	0.587216	3.010350
C	-0.348959	1.002013	4.453534
H	-0.921414	1.945198	4.513840
H	-0.884203	0.231822	5.030538
H	0.646379	1.179343	4.888342
O	0.774893	0.981352	2.305728
Cs	-3.864437	-0.198657	1.159563
Br	-1.307123	2.196889	0.044244
H	0.963211	-2.347159	-1.503225
O	-0.884666	-0.940976	-1.145459
C	-1.696238	-1.953328	-1.009457
C	-2.151683	-2.565957	-2.334825
H	-1.603941	-2.144127	-3.191512
H	-2.018142	-3.660312	-2.301769
H	-3.229806	-2.368549	-2.465927
O	-2.109275	-2.424793	0.075575
O	-4.116644	0.250973	-1.860182
C	-3.514839	0.592641	-2.883045
O	-2.370944	1.284237	-2.898625
H	-2.066835	1.454450	-1.952024
C	-3.979545	0.244685	-4.282055
H	-3.389329	-0.612630	-4.651678
H	-5.041519	-0.037490	-4.264577
H	-3.812040	1.083758	-4.975108

Statistical Thermodynamic Analysis
Temperature= 298.150 Kelvin   Pressure= 1.00000 Atm
==

SCF Energy=   -6370.56267826   Predicted Change= -3.877580D-08
Zero-point correction (ZPE)=   -6370.1180   0.44461
Internal Energy (U)=   -6370.0780   0.48462
Enthalpy (H)=   -6370.0771   0.48556
Gibbs Free Energy (G)=   -6370.1941   0.36854
Entropy (S)=   0.0003925

Frequencies --   16.0132   24.5962   34.3603
Supporting Information: 050-Product-Catalyst-Complex-1HOAc-2OAc-CsBr-02-PBE.log
----
Using Gaussian 16: ES64L-G16RevA.03 25-Dec-2016
==
# pbepbe/gen/auto pseudo=read gfprint gfinput empiricaldispersion=gd3bj
scf=(direct,vshift=200,tight,maxcycle=300,yqc) opt=(gdiis,maxcycle=250)
freq=noraman
#N Geom=AllCheck Guess=TCheck SCRF=Check GenChk RPBEPBE/Gen/Auto Freq
----
Pointgroup= C1   Stoichiometry=   C15H28B10BrCsO6RuSe   C1[X(C15H28B10BrCsO6RuSe)]
#Atoms= 63
Charge = 0   Multiplicity = 1

SCF Energy= -6370.56046471   Predicted Change= -4.286254D-09
==
Optimization completed.   {Found   1   times}

Item	Max Val.		Criteria	Pass?	RMS Val.		Criteria	Pass?
Force	0.00000	||	0.00045	[YES]	0.00000	||	0.00030	[YES]
Displ	0.00183	||	0.00180	[NO]	0.00183	||	0.00180	[YES]

Atomic Type	Coordinates (Angstroms) X	Y	Z
B	-4.128164	-1.978511	0.150918
B	-3.891097	-2.964944	-1.326117
H	-4.764943	-3.159419	-2.121371
H	-5.161877	-1.439217	0.422453
B	-2.694885	-4.232612	-0.956658
B	-2.194318	-2.775778	-1.851545
B	-3.078242	-2.657971	1.417830
B	-2.567201	-1.227787	0.501481
H	-3.196821	-2.592245	2.605638
H	-1.752839	-2.892638	-2.956135
H	-2.587332	-5.308050	-1.467462

C	-1.508145	-2.509679	0.771089
C	-1.259848	-3.396675	-0.563372
B	-3.892892	-3.756483	0.280465
B	-2.213434	-4.052433	0.737966
H	-1.707392	-4.912432	1.392668
H	-4.749660	-4.513784	0.634568
B	-3.084798	-1.391430	-1.190404
H	-3.369108	-0.446977	-1.862899
B	-1.384371	-1.621956	-0.746198
H	-0.178252	-1.396135	-1.255249
Cs	3.345594	2.067972	0.367139
C	0.131199	-3.976769	-0.771592
H	0.891239	-3.339934	-0.289661
H	0.196170	-4.995506	-0.360234
H	0.349714	-4.016406	-1.849673
Ru	-0.039303	0.117533	-0.545118
O	0.956948	-1.029573	0.959764
O	-0.897664	1.440397	-2.005956
C	1.445991	-0.479907	2.045123
C	-0.354271	2.467167	-1.465768
O	1.495262	0.741032	2.289014
O	0.383109	2.286391	-0.422113
C	-0.633532	3.848425	-1.996316
H	-0.739662	3.821123	-3.091696
H	-1.587934	4.205009	-1.567533
H	0.161701	4.551905	-1.706018
C	1.997795	-1.490684	3.052947
H	3.075828	-1.301958	3.194000
H	1.506414	-1.338610	4.028734
H	1.853625	-2.531876	2.724249
Se	-1.788416	0.473072	1.253531
C	-3.210513	1.735370	0.821498
C	-3.883548	2.295106	1.919338
C	-3.543247	2.106787	-0.489819
C	-4.907701	3.227830	1.701881
H	-3.610655	1.999726	2.937564
C	-4.568434	3.042526	-0.694587
H	-3.001354	1.678196	-1.334787
C	-5.251221	3.602869	0.395169
H	-5.436582	3.659961	2.557667
H	-4.835171	3.329107	-1.717473
H	-6.051097	4.331105	0.226504
H	-0.614045	-2.351706	1.383690
Br	1.973219	-0.153723	-2.160567
O	4.529675	-0.768438	0.702868
C	4.318926	-1.965416	0.492141

O	3.463643	-2.435767	-0.425725
H	3.008474	-1.657888	-0.886780
C	4.986086	-3.084900	1.264005
H	5.290008	-3.901229	0.589873
H	5.854363	-2.694870	1.812929
H	4.264312	-3.504420	1.986842

Statistical Thermodynamic Analysis
Temperature= 298.150 Kelvin Pressure= 1.00000 Atm

===

SCF Energy= -6370.56046471 Predicted Change= -4.286254D-09
Zero-point correction (ZPE)= -6370.1155 0.44491
Internal Energy (U)= -6370.0756 0.48478
Enthalpy (H)= -6370.0747 0.48573
Gibbs Free Energy (G)= -6370.1914 0.36902
Entropy (S)= 0.00039145

Frequencies -- 14.5182 22.9621 26.2596
Supporting Information: 050-Product-Catalyst-Complex-1HOAc-2OAc-CsBr-03-PBE.log

----

Using Gaussian 16: ES64L-G16RevA.03 25-Dec-2016

===

# pbepbe/gen/auto pseudo=read gfprint gfinput empiricaldispersion=gd3bj
scf=(direct,vshift=200,tight,maxcycle=300,yqc) opt=(gdiis,maxcycle=250)
freq=noraman
#N Geom=AllCheck Guess=TCheck SCRF=Check GenChk RPBEPBE/Gen/Auto Freq

----

Pointgroup= C1 Stoichiometry= C15H28B10BrCsO6RuSe C1[X(C15H28B10BrCsO6RuSe)]
#Atoms= 63
Charge = 0 Multiplicity = 1

SCF Energy= -6370.54071817 Predicted Change= -1.192741D-08

===

Optimization completed on the basis of negligible forces. {Found 2 times}

Item	Max Val.	Criteria	Pass?	RMS Val.	Criteria	Pass?
Force	0.00000 ||	0.00045	[YES]	0.00000 ||	0.00030	[YES]
Displ	0.00713 ||	0.00180	[NO]	0.00713 ||	0.00180	[YES]

Atomic Type	Coordinates (Angstroms) X	Y	Z
B	-2.720228	3.215484	-1.230469
B	-3.168217	4.213480	0.176235
H	-4.021560	5.052488	0.137009
H	-3.233976	3.314221	-2.307515
B	-1.713654	4.438184	1.175243

B	-2.931170	3.233721	1.640779
B	-0.989513	2.833959	-1.091648
B	-2.202343	1.625639	-0.623310
H	-0.221200	2.584201	-1.968971
H	-3.490657	3.317633	2.693853
H	-1.450191	5.338197	1.916630
C	-0.854477	1.951557	0.366258
C	-1.260975	2.866035	1.680262
B	-1.573079	4.436231	-0.598991
B	-0.365355	3.594816	0.388313
H	0.785964	3.824174	0.588815
H	-1.259236	5.419932	-1.204151
B	-3.556446	2.463708	0.161136
H	-4.669261	2.028837	0.111777
B	-2.349999	1.632560	1.157617
H	-2.452458	0.663026	1.836115
C	-0.505782	2.596209	2.973027
H	-1.210194	2.292430	3.761898
H	0.229060	1.787262	2.826701
H	0.026908	3.502914	3.297015
Ru	-0.231684	-0.568843	0.095232
Se	-2.067291	-0.181024	-1.510235
C	-3.694146	-0.990901	-0.801096
C	-4.934098	-0.537481	-1.276961
C	-3.609039	-2.050174	0.110943
C	-6.110256	-1.137294	-0.806533
H	-4.981897	0.282829	-2.000407
C	-4.793874	-2.647893	0.567034
H	-2.631453	-2.395426	0.457669
C	-6.040966	-2.192362	0.115861
H	-7.080984	-0.779231	-1.164822
H	-4.736030	-3.475942	1.281364
H	-6.961012	-2.661559	0.479524
O	1.073669	-0.917454	1.881397
C	0.029380	-1.305625	2.531090
C	0.141783	-1.831966	3.939213
H	0.297243	-2.925378	3.907981
H	0.999179	-1.374243	4.457097
H	-0.788891	-1.638796	4.494461
O	-1.108128	-1.246997	1.945491
Cs	3.415634	-1.705283	0.098267
O	0.951403	0.014647	-1.578866
C	1.856331	0.916739	-1.431809
O	2.159521	1.436749	-0.306982
H	0.088317	1.335724	0.522395
C	2.604054	1.332954	-2.687851

H	2.510313	2.423969	-2.823544
H	3.679235	1.110105	-2.567883
H	2.210408	0.815124	-3.573635
Br	0.059602	-2.962875	-0.530643
O	5.285020	0.657557	-0.433140
C	5.408968	1.879700	-0.261122
O	4.394550	2.734414	-0.168428
H	3.492671	2.216842	-0.251530
C	6.755670	2.566066	-0.141317
H	6.821834	3.104367	0.819204
H	7.565946	1.827712	-0.214447
H	6.864274	3.320061	-0.939269

Statistical Thermodynamic Analysis
Temperature= 298.150 Kelvin Pressure= 1.00000 Atm

==

SCF Energy= -6370.54071817 Predicted Change= -1.192741D-08
Zero-point correction (ZPE)= -6370.0964 0.44428
Internal Energy (U)= -6370.0564 0.48425
Enthalpy (H)= -6370.0555 0.48520
Gibbs Free Energy (G)= -6370.1736 0.36705
Entropy (S)= 0.00039629

Frequencies -- 16.4652 24.9773 30.9576

15. Transition State Computed Geometries and Energies

Supporting Information: 015-Cs-Cluster-RuII-1HOAc-2OAc-Cymene-Deprotonation-TS-01-PBE.log

---

Using Gaussian 16: ES64L-G16RevA.03 25-Dec-2016

===

# pbepbe/gen/auto pseudo=read gfprint gfinput empiricaldispersion=gd3bj
scf=(direct,vshift=200,tight,maxcycle=300,yqc)
opt=(gdiis,maxcycle=250,calcfc,ts,noeigentest) freq=noraman
#N Geom=AllCheck Guess=TCheck SCRF=Check GenChk RPBEPBE/Gen/Auto Freq

---

Pointgroup= C1 Stoichiometry= C20H37B10CsO8Ru C1[X(C20H37B10CsO8Ru)] #Atoms= 77
Charge = 0 Multiplicity = 1

SCF Energy= -1746.94503242 Predicted Change= -9.925762D-10

===

Optimization completed. {Found 2 times}

Item	Max Val.	Criteria	Pass?	RMS Val.	Criteria	Pass?
Force	0.00000 ||	0.00045	[YES]	0.00000 ||	0.00030	[YES]
Displ	0.00075 ||	0.00180	[YES]	0.00075 ||	0.00180	[YES]

Atomic Type	Coordinates (Angstroms) X	Y	Z
B	3.922466	-1.387748	0.668509
B	5.118181	-1.502447	-0.647739
H	6.055653	-2.246344	-0.617191
H	3.999721	-2.041797	1.672243
B	5.253351	0.096865	-1.412936
B	4.274610	-1.166813	-2.180316
B	3.335320	0.282397	0.706500
B	2.320753	-1.003226	-0.039482
H	2.929059	0.925948	1.626182
H	1.144240	-2.138721	-0.118537
H	4.535964	-1.554358	-3.280427
H	6.183267	0.570775	-1.995511
C	2.586541	0.513813	-0.798827
C	3.701058	0.435795	-2.048367
B	5.040589	-0.019500	0.345397
B	4.154075	1.204427	-0.578678
H	4.230116	2.393568	-0.582639
H	5.898013	0.320112	1.108837
B	3.455070	-2.097756	-0.901116
H	3.200966	-3.253832	-1.058392
B	2.571752	-0.856517	-1.808112
H	1.695211	-0.935024	-2.612368

C	1.372102	1.437252	-0.820799
O	0.375168	1.004450	-0.120566
O	1.392668	2.525227	-1.433123
Cs	-2.760274	1.026163	-1.313155
C	3.519681	1.309607	-3.279294
H	3.115420	0.714599	-4.112080
H	2.836449	2.142031	-3.055991
H	4.499159	1.715218	-3.574850
Ru	0.313571	-0.904124	0.818083
O	-0.697966	-1.319576	-0.969991
C	-0.399143	-2.456213	-1.535953
O	0.542741	-3.192000	-1.130944
C	-1.245886	-2.829586	-2.730264
H	-0.935741	-2.210146	-3.591745
H	-1.088732	-3.886524	-2.987985
H	-2.313889	-2.612572	-2.524570
C	1.128332	-1.077314	2.926961
C	0.716007	-2.351713	2.418115
C	0.224483	0.018422	2.936215
H	2.172918	-0.922663	3.211421
C	-0.632097	-2.573211	1.979314
H	1.452587	-3.155139	2.320927
C	-1.145834	-0.221340	2.524929
C	-1.578753	-1.487425	2.096019
H	-1.852839	0.612296	2.504236
H	-2.603831	-1.595865	1.682492
C	0.666478	1.366329	3.479264
H	1.755675	1.443051	3.307069
C	-0.007397	2.572151	2.806541
H	0.450704	3.505103	3.176039
H	0.108698	2.541093	1.712926
H	-1.085317	2.621671	3.046065
C	0.423885	1.373683	5.005631
H	0.954623	0.546271	5.506530
H	0.777287	2.324888	5.439368
H	-0.653083	1.275205	5.230820
C	-1.078902	-3.920013	1.480046
H	-1.446725	-4.524504	2.330047
H	-1.908162	-3.815973	0.764070
H	-0.255630	-4.461594	0.991032
O	-4.086311	-1.674774	-1.770359
C	-4.168943	-1.985297	-0.540020
O	-3.994910	-1.193712	0.451338
C	-4.508591	-3.459744	-0.229520
H	-5.484995	-3.712987	-0.678224
H	-3.767836	-4.129920	-0.703534

H	-4.549394	-3.659551	0.853570
O	-1.896215	3.944695	-0.837839
C	-1.144343	4.695039	-0.212361
O	0.190696	4.579401	-0.192129
H	0.474042	3.778823	-0.745147
C	-1.621252	5.846974	0.647816
H	-1.054381	6.763469	0.417181
H	-2.695717	6.013551	0.491094
H	-1.437912	5.610997	1.710980

Statistical Thermodynamic Analysis
Temperature= 298.150 Kelvin Pressure= 1.00000 Atm

==

SCF Energy= -1746.94503242 Predicted Change= -9.925762D-10
Zero-point correction (ZPE)= -1746.3728 0.57219
Internal Energy (U)= -1746.3282 0.61677
Enthalpy (H)= -1746.3273 0.61772
Gibbs Free Energy (G)= -1746.4522 0.49278
Entropy (S)= 0.00041904

Frequencies -- -557.2219 16.9587 22.3071
Supporting Information: 015-Cs-Cluster-RuII-1HOAc-2OAc-PhSeBr-01-Deprotonation-TS-01-PBE.log

----

Using Gaussian 16: ES64L-G16RevA.03 25-Dec-2016

==

# pbepbe/gen/auto pseudo=read gfprint gfinput empiricaldispersion=gd3bj
scf=(direct,vshift=200,tight,maxcycle=300,yqc)
opt=(gdiis,maxcycle=250,calcfc,ts,noeigentest) freq=noraman
#N Geom=AllCheck Guess=TCheck SCRF=Check GenChk RPBEPBE/Gen/Auto Freq

----

Pointgroup= C1 Stoichiometry= C16H28B10BrCsO8RuSe C1[X(C16H28B10BrCsO8RuSe)]
#Atoms= 66
Charge = 0 Multiplicity = 1

SCF Energy= -6558.91955355 Predicted Change= -1.359603D-09

==

Optimization completed. {Found 2 times}

Item	Max Val.		Criteria	Pass?	RMS Val.		Criteria	Pass?
Force	0.00000	||	0.00045	[YES]	0.00000	||	0.00030	[YES]
Displ	0.00141	||	0.00180	[YES]	0.00141	||	0.00180	[YES]

Atomic Type	Coordinates (Angstroms) X	Y	Z
B	-2.509267	2.598895	0.037309

B	-2.345755	4.287195	0.587699
H	-3.275917	4.940807	0.965503
H	-3.575225	2.061270	-0.007009
B	-1.013946	5.044214	-0.311245
B	-0.745907	4.459345	1.343207
B	-1.267844	2.334168	-1.193693
B	-1.018154	1.668626	0.471710
H	-1.275713	1.604075	-2.145022
H	-1.201768	0.455533	1.845135
H	-0.400166	5.213004	2.205897
H	-0.848765	6.199466	-0.573218
C	0.204151	2.564514	-0.401164
C	0.380540	4.150621	0.105851
B	-2.090194	3.897572	-1.134385
B	-0.343639	3.840931	-1.420506
H	0.302921	4.076975	-2.393586
H	-2.823820	4.243211	-2.015770
B	-1.676591	2.956303	1.568127
H	-2.119308	2.696246	2.645569
B	0.059435	2.882635	1.257654
H	0.985912	2.599297	1.955378
C	1.394164	1.718916	-0.827927
O	1.282960	0.473619	-0.498135
O	2.361521	2.218139	-1.443435
Cs	3.372226	-1.342680	0.891687
C	1.761025	4.787342	0.121966
H	2.175921	4.774158	1.141384
H	2.436795	4.247527	-0.557435
H	1.673132	5.832664	-0.211288
Ru	-0.286771	-0.387670	0.617646
O	0.920323	-0.036518	2.389835
O	-1.382732	-1.865341	1.705916
C	0.166261	0.291093	3.385768
C	-0.518305	-2.804588	1.513279
O	-1.076845	0.524712	3.271573
O	0.564411	-2.536734	0.884809
C	-0.833006	-4.196555	1.989283
H	0.089678	-4.737880	2.250030
H	-1.522586	-4.170917	2.846465
H	-1.325549	-4.730412	1.156441
C	0.811347	0.456571	4.744913
H	1.033318	1.527217	4.901280
H	0.117807	0.138906	5.537837
H	1.752888	-0.109417	4.813572
Se	-1.484730	-0.855833	-1.353390
Br	-1.705396	-3.350293	-1.597027

C	-3.365823	-0.549062	-1.045525
C	-4.111557	-0.048711	-2.124076
C	-3.947818	-0.760306	0.213956
C	-5.462383	0.270469	-1.929550
H	-3.635147	0.109211	-3.096631
C	-5.302283	-0.454173	0.389230
H	-3.341179	-1.149768	1.035384
C	-6.055844	0.067834	-0.675178
H	-6.048410	0.680888	-2.757921
H	-5.767644	-0.608894	1.367973
H	-7.110343	0.320809	-0.524709
O	5.047581	-0.192563	-1.319801
C	5.071521	0.228306	-2.482706
O	4.189276	1.084774	-2.995261
C	6.121407	-0.177375	-3.497667
H	6.814609	-0.905400	-3.054491
H	5.637904	-0.610746	-4.389224
H	6.680409	0.711649	-3.835358
H	3.512354	1.374513	-2.290297

Statistical Thermodynamic Analysis
Temperature= 298.150 Kelvin Pressure= 1.00000 Atm

===

SCF Energy= -6558.91955355 Predicted Change= -1.359603D-09
Zero-point correction (ZPE)= -6558.4655 0.45401
Internal Energy (U)= -6558.4233 0.49622
Enthalpy (H)= -6558.4223 0.49716
Gibbs Free Energy (G)= -6558.5453 0.37417
Entropy (S)= 0.00041251

Frequencies -- -712.1389 9.4685 22.0204
Supporting Information: 015-Cs-Cluster-RuII-1HOAc-2OAc-PhSeBr-01-Deprotonation-TS-02-PBE.log

----

Using Gaussian 16: ES64L-G16RevA.03 25-Dec-2016

===

# pbepbe/gen/auto pseudo=read gfprint gfinput empiricaldispersion=gd3bj
scf=(direct,vshift=200,tight,maxcycle=300,yqc)
opt=(gdiis,maxcycle=250,calcfc,ts,noeigentest) freq=noraman
#N Geom=AllCheck Guess=TCheck SCRF=Check GenChk RPBEPBE/Gen/Auto Freq

----

Pointgroup= C1 Stoichiometry= C16H28B10BrCsO8RuSe C1[X(C16H28B10BrCsO8RuSe)] #Atoms= 66
Charge = 0 Multiplicity = 1

SCF Energy= -6558.91508094 Predicted Change= -2.725523D-09

==

Optimization completed. {Found 2 times}

Item	Max Val.	Criteria	Pass?	RMS Val.	Criteria	Pass?
Force	0.00000 ||	0.00045	[YES]	0.00000 ||	0.00030	[YES]
Displ	0.00079 ||	0.00180	[YES]	0.00079 ||	0.00180	[YES]

Atomic Type	Coordinates (Angstroms) X	Y	Z
B	-2.653916	2.454012	0.160895
B	-2.613271	4.171203	0.650808
H	-3.577540	4.720988	1.101508
H	-3.655190	1.806452	0.230289
B	-1.422506	5.034533	-0.369645
B	-0.992290	4.521898	1.275205
B	-1.474280	2.280049	-1.143442
B	-1.084280	1.649569	0.506133
H	-1.477606	1.507908	-2.062932
H	-1.140364	0.461385	1.909723
H	-0.686751	5.221119	2.197066
H	-1.514305	6.194004	-0.655013
C	0.020110	2.678572	-0.465542
B	-2.446345	3.750149	-1.068358
B	-0.719436	3.847683	-1.488821
H	-0.219609	4.032359	-2.557217
H	-3.287031	3.964624	-1.894740
B	-1.746300	2.937307	1.599726
H	-1.970496	2.654630	2.736354
C	1.263815	1.889014	-0.864932
O	1.283980	0.657286	-0.446637
O	2.164386	2.435916	-1.527413
Cs	3.441305	-1.229612	0.710985
Ru	-0.235971	-0.337958	0.621961
O	0.950520	-0.195521	2.450566
O	-1.280819	-1.895145	1.655612
C	0.181630	0.077934	3.448739
C	-0.390314	-2.799661	1.417849
O	-1.029432	0.445201	3.314130
O	0.666909	-2.480823	0.771419
C	-0.644944	-4.212901	1.867481
H	0.302143	-4.725535	2.096726
H	-1.316637	-4.232565	2.738903
H	-1.134024	-4.746826	1.032814
C	0.771027	0.009841	4.840690
H	1.294554	0.958640	5.057004
H	-0.022013	-0.124923	5.589886

H	1.506066	-0.807043	4.912199
Se	-1.399597	-0.811188	-1.389258
Br	-1.473573	-3.296442	-1.684522
C	-3.302975	-0.640407	-1.098222
C	-4.065450	-0.124504	-2.156786
C	-3.886571	-0.982424	0.130782
C	-5.439483	0.078484	-1.969602
H	-3.587531	0.137553	-3.105655
C	-5.263990	-0.794470	0.295897
H	-3.264576	-1.378996	0.937305
C	-6.037171	-0.257061	-0.746094
H	-6.040558	0.501728	-2.780422
H	-5.732400	-1.053028	1.250963
H	-7.110415	-0.095885	-0.602425
O	5.060928	0.257378	-1.320429
C	5.127247	0.765982	-2.446783
O	4.200568	1.562662	-2.973195
C	6.294411	0.554446	-3.390357
H	6.986290	-0.191808	-2.976118
H	5.932664	0.231294	-4.380317
H	6.828504	1.508743	-3.538285
H	3.448651	1.742916	-2.307973
C	-0.124946	3.064660	1.111978
C	1.043932	2.828774	2.056308
H	0.653435	2.776045	3.083760
H	1.553866	1.882687	1.832584
H	1.760527	3.661204	1.994641
B	0.166105	4.338406	-0.051910
H	1.260999	4.809664	-0.051832

Statistical Thermodynamic Analysis
Temperature= 298.150 Kelvin Pressure= 1.00000 Atm

==

SCF Energy= -6558.91508094 Predicted Change= -2.725523D-09
Zero-point correction (ZPE)= -6558.4611 0.45391
Internal Energy (U)= -6558.4191 0.49596
Enthalpy (H)= -6558.4181 0.49690
Gibbs Free Energy (G)= -6558.5403 0.37474
Entropy (S)= 0.00040974

Frequencies -- -729.8574 9.4700 22.7124
Supporting Information: 017-Cs-Cluster-RuII-1HOAc-2OAc-PhSeBr-Decarboxylation-TS-01-PBE.log

----

Using Gaussian 16: ES64L-G16RevA.03 25-Dec-2016

==

# pbepbe/gen/auto pseudo=read gfprint gfinput empiricaldispersion=gd3bj
scf=(direct,vshift=200,tight,maxcycle=300,yqc)
opt=(gdiis,maxcycle=250,calcfc,ts,noeigentest) freq=noraman
#N Geom=AllCheck Guess=TCheck SCRF=Check GenChk RPBEPBE/Gen/Auto Freq

---

Pointgroup= C1 Stoichiometry= C16H28B10BrCsO8RuSe C1[X(C16H28B10BrCsO8RuSe)]
#Atoms= 66
Charge = 0 Multiplicity = 1

SCF Energy= -6558.85345247 Predicted Change= -1.407139D-08

===

Optimization completed on the basis of negligible forces. {Found 2 times}

Item	Max Val.	Criteria	Pass?	RMS Val.	Criteria	Pass?
Force	0.00000 ||	0.00045	[YES]	0.00000 ||	0.00030	[YES]
Displ	0.00637 ||	0.00180	[NO]	0.00637 ||	0.00180	[YES]

Atomic Type	Coordinates (Angstroms) X	Y	Z
B	3.934936	1.189778	2.286253
B	5.460820	0.490985	1.707102
H	6.537900	0.854390	2.086101
H	3.864888	2.076184	3.090254
B	5.218706	-0.015999	0.021862
B	5.159685	-1.215395	1.318470
B	2.780596	1.107711	0.957182
B	2.683331	-0.089084	2.243981
H	1.938975	1.932358	0.766438
H	1.715550	-0.176236	2.941668
H	5.923705	-2.135775	1.328376
H	6.029043	-0.116014	-0.851907
C	2.515656	-0.579049	0.605618
C	3.992932	-1.214024	0.059526
B	4.462360	1.471061	0.597078
B	3.545730	0.356420	-0.457390
H	3.222334	0.508774	-1.593040
H	4.772274	2.548108	0.173753
B	4.358249	-0.480110	2.725066
H	4.613253	-0.829099	3.842066
B	3.463673	-1.575223	1.668890
H	3.089621	-2.675270	1.917351
C	4.040977	-2.297370	-1.009836
H	3.918154	-3.295413	-0.570557
H	3.250081	-2.130874	-1.753710
H	5.023686	-2.231157	-1.502415
Ru	0.388069	-0.046203	-0.806198

C	0.550407	-2.289795	-2.778319
O	-0.567967	-2.670724	-2.384149
O	1.200510	-1.235407	-2.345034
C	1.323595	-3.078339	-3.831045
H	2.004400	-2.433399	-4.407450
H	1.931897	-3.841388	-3.312482
H	0.624156	-3.598750	-4.503137
Cs	-2.793528	-2.285416	-0.458193
O	0.605397	1.385210	-2.340734
C	-0.577509	1.133211	-2.759360
C	-1.140457	1.751763	-4.000672
H	-2.231281	1.614493	-4.040876
H	-0.888522	2.823844	-4.022666
H	-0.684118	1.273797	-4.885034
O	-1.264623	0.292803	-2.045673
C	1.108663	-1.884945	0.315174
O	1.540001	-2.969797	-0.001017
O	-0.015483	-1.475050	0.790933
Se	-0.390424	1.475773	0.866331
Br	-2.902345	1.177471	1.124276
C	-0.526697	3.219531	0.018497
C	0.405682	4.192229	0.413243
C	-1.499213	3.507200	-0.951259
C	0.386518	5.451527	-0.204034
H	1.146110	3.972089	1.187735
C	-1.517899	4.773712	-1.548579
H	-2.245408	2.752938	-1.213926
C	-0.570583	5.743752	-1.184955
H	1.119057	6.207165	0.096232
H	-2.281159	5.004073	-2.299525
H	-0.587427	6.730965	-1.657239
O	-2.669226	-2.186029	2.622560
C	-2.165864	-1.413373	3.438902
O	-0.907295	-0.942407	3.346439
H	-0.515393	-1.258343	2.482939
C	-2.888038	-0.844603	4.636862
H	-3.767409	-1.459570	4.873074
H	-2.218392	-0.770443	5.507590
H	-3.220421	0.177527	4.381152

Statistical Thermodynamic Analysis
Temperature= 298.150 Kelvin   Pressure= 1.00000 Atm

==

SCF Energy=   -6558.85345247   Predicted Change= -1.407139D-08
Zero-point correction (ZPE)=   -6558.3978   0.45560
Internal Energy (U)=   -6558.3554   0.49803

Enthalpy (H)= -6558.3544 0.49897
Gibbs Free Energy (G)= -6558.4768 0.37659
Entropy (S)= 0.00041046

Frequencies -- -215.7661 17.4723 19.8521
Supporting Information: 025-Cs-Deprotonated-Cluster-RuII-2HOAc-1OAc-PhSeBr-03-Selenylation-TS-02-PBE.log

---

Using Gaussian 16: ES64L-G16RevA.03 25-Dec-2016

===

# pbepbe/gen/auto pseudo=read gfprint gfinput empiricaldispersion=gd3bj
scf=(direct,vshift=200,tight,maxcycle=300,yqc)
opt=(gdiis,maxcycle=250,calcfc,ts,noeigentest) freq=noraman
#N Geom=AllCheck Guess=TCheck SCRF=Check GenChk RPBEPBE/Gen/Auto Freq

---

Pointgroup= C1 Stoichiometry= C16H28B10BrCsO8RuSe C1[X(C16H28B10BrCsO8RuSe)]
#Atoms= 66
Charge = 0 Multiplicity = 1

SCF Energy= -6558.90417916 Predicted Change= -4.349955D-09

===

Optimization completed on the basis of negligible forces. {Found 2 times}

Item	Max Val.	Criteria	Pass?	RMS Val.	Criteria	Pass?
Force	0.00000 ||	0.00045	[YES]	0.00000 ||	0.00030	[YES]
Displ	0.00231 ||	0.00180	[NO]	0.00231 ||	0.00180	[YES]

Atomic Coordinates (Angstroms)

Type	X	Y	Z
B	2.399852	2.955556	0.533545
B	2.453331	4.421041	-0.482496
H	3.444117	5.075468	-0.636024
H	3.345996	2.535893	1.135900
B	0.860215	5.194440	-0.414851
B	1.298177	4.190130	-1.815680
B	0.759478	2.821386	1.236457
B	1.212026	1.826077	-0.173952
H	0.393346	2.446857	2.306756
H	1.695466	-2.141385	2.235435
H	1.327373	4.655481	-2.916726
H	0.587173	6.350150	-0.555612
C	-0.286978	2.689850	-0.132594
C	-0.241738	4.070983	-1.074763
B	1.525423	4.427617	1.040589
B	-0.189445	4.221478	0.634033
H	-1.170998	4.619846	1.179611

H	1.829164	5.067583	2.005576
B	2.256144	2.807313	-1.232096
H	3.080414	2.295672	-1.932443
B	0.527772	2.610023	-1.623663
H	-0.012762	2.043026	-2.523912
C	-1.583186	1.870222	-0.005742
O	-1.431747	0.654868	0.394979
O	-2.671264	2.410643	-0.304204
Cs	-2.669360	-1.810001	-0.950797
C	-1.479917	4.545571	-1.819748
H	-1.848233	3.771328	-2.507700
H	-2.288221	4.798468	-1.121726
H	-1.200142	5.437555	-2.401637
Ru	0.416062	-0.068968	1.165503
O	-0.591979	-1.876080	1.646611
O	0.319907	0.414062	3.402392
C	-0.001238	-2.961152	1.902305
C	1.474176	-0.083486	3.531242
O	1.273745	-3.070425	2.216003
O	2.058979	-0.586033	2.464139
C	2.219949	-0.104560	4.837438
H	2.592067	-1.121255	5.048383
H	3.099537	0.559203	4.767430
H	1.569547	0.238167	5.654507
C	-0.730450	-4.271319	1.800345
H	-0.544204	-4.667576	0.784564
H	-0.341087	-4.996639	2.530203
H	-1.810892	-4.126171	1.949600
Se	1.181727	-0.304273	-1.010231
C	3.097970	-0.635975	-1.091544
C	3.602266	-1.127704	-2.306226
C	3.947699	-0.338597	-0.017875
C	4.981325	-1.327541	-2.442275
H	2.916991	-1.371957	-3.123336
C	5.327711	-0.536941	-0.169182
H	3.533630	0.035875	0.920547
C	5.844886	-1.029421	-1.376222
H	5.381986	-1.716640	-3.383933
H	5.999911	-0.301811	0.662618
H	6.923394	-1.180954	-1.488381
Br	0.629853	-3.033132	-1.634495
O	-5.167093	-0.188123	-0.492881
C	-5.720240	0.711892	0.149513
O	-5.129672	1.846918	0.523756
H	-4.170315	1.883225	0.183009
C	-7.165131	0.650056	0.602554

H	-7.608002	-0.315212	0.321455
H	-7.226700	0.791311	1.694537
H	-7.738501	1.470792	0.139069

Statistical Thermodynamic Analysis
Temperature= 298.150 Kelvin   Pressure= 1.00000 Atm

===

SCF Energy=   -6558.90417916   Predicted Change= -4.349955D-09
Zero-point correction (ZPE)=   -6558.4460   0.45814
Internal Energy (U)=   -6558.4037   0.50046
Enthalpy (H)=   -6558.4027   0.50140
Gibbs Free Energy (G)=   -6558.5270   0.37712
Entropy (S)=   0.00041684

Frequencies -- -156.4100   10.0622   19.4796
Supporting Information: 035-Selenylated-Cluster-RuII-1OAc-2HOAc-Cymene-CsBr-Decarboxylation-TS-01.log

----

Using Gaussian 16: ES64L-G16RevA.03 25-Dec-2016

===

# pbepbe/gen/auto pseudo=read gfprint gfinput empiricaldispersion=gd3bj
scf=(direct,vshift=200,tight,maxcycle=300,yqc)
opt=(gdiis,maxcycle=250,calcfc,ts,noeigentest) freq=noraman
#N Geom=AllCheck Guess=TCheck SCRF=Check GenChk RPBEPBE/Gen/Auto Freq

----

Pointgroup= C1   Stoichiometry= C26H42B10BrCsO8RuSe   C1[X(C26H42B10BrCsO8RuSe)]
#Atoms= 90
Charge = 0   Multiplicity = 1

SCF Energy= -6947.87503268   Predicted Change= -4.048755D-09

===

Optimization completed.   {Found 1 times}

Item	Max Val.	Criteria	Pass?	RMS Val.	Criteria	Pass?
Force	0.00000 ||	0.00045	[YES]	0.00000 ||	0.00030	[YES]
Displ	0.00461 ||	0.00180	[NO]	0.00461 ||	0.00180	[YES]

Atomic Type	Coordinates (Angstroms) X	Y	Z
B	0.253632	-0.562985	3.577026
B	-0.874411	-1.583527	4.509625
H	-0.984513	-1.504379	5.698816
H	0.960993	0.260828	4.071694
B	-1.016686	-3.155884	3.692363
B	-2.302535	-1.939459	3.500898
B	0.773333	-1.505919	2.150565

B	-0.493341	-0.303724	1.997125
H	1.781110	-1.420102	1.525055
H	-3.426027	-2.170043	3.840711
H	-1.264865	-4.221257	4.173802
C	-0.657113	-1.855489	1.246457
C	-1.699456	-2.828998	2.172423
B	0.550090	-2.318160	3.714775
B	-0.010471	-3.111386	2.221985
H	0.408954	-4.087757	1.685287
H	1.482139	-2.781819	4.307319
B	-1.522871	-0.332694	3.426839
H	-2.100167	0.640494	3.809324
B	-2.022201	-1.164139	1.932144
H	-3.066821	-0.824709	1.288278
C	-0.113628	-2.290285	-0.520510
O	-0.541095	-3.391612	-0.838884
O	0.645036	-1.405600	-0.904593
C	-2.572706	-3.838554	1.450320
H	-2.639453	-4.753301	2.058801
H	-3.586798	-3.435043	1.306260
H	-2.127438	-4.081438	0.474606
Ru	-2.324004	-0.553723	-0.357666
Se	-0.509059	1.040371	0.493945
C	-1.514646	2.552514	1.191097
C	-0.960010	3.814418	0.931055
C	-2.773388	2.427476	1.794314
C	-1.674439	4.962357	1.301179
H	0.019285	3.890573	0.454223
C	-3.474317	3.583136	2.169200
H	-3.216344	1.443322	1.956790
C	-2.927197	4.850841	1.921604
H	-1.242910	5.948250	1.101581
H	-4.452536	3.487567	2.651914
H	-3.478921	5.751198	2.210003
C	-4.247715	0.153312	-1.078404
C	-4.307181	-1.267314	-1.342982
C	-3.261821	1.001123	-1.686132
H	-5.008525	0.609425	-0.436742
C	-3.305080	-1.902128	-2.106983
H	-5.117594	-1.854407	-0.903549
C	-2.177204	0.342066	-2.366101
C	-2.180925	-1.077025	-2.500915
H	-1.300896	0.951870	-2.642812
H	-1.302414	-1.563078	-2.935482
C	-3.320068	-3.370448	-2.483193
H	-2.342440	-3.781464	-2.169806

C	-3.413683	-3.486196	-4.023002
H	-2.594312	-2.946672	-4.526925
H	-3.355812	-4.546296	-4.322897
H	-4.371146	-3.076643	-4.391146
C	-4.438400	-4.180871	-1.817142
H	-4.394514	-4.123210	-0.717153
H	-5.437237	-3.838855	-2.144359
H	-4.346446	-5.242699	-2.099019
C	-3.294630	2.494959	-1.582384
H	-3.855341	2.889336	-2.452148
H	-3.796796	2.839560	-0.665278
H	-2.261088	2.885150	-1.632061
Br	2.059127	2.345425	1.674750
Cs	2.760536	0.942054	-1.653619
O	-0.163261	2.474892	-2.071912
C	0.444304	3.427215	-2.696773
O	1.583614	3.340198	-3.226352
C	-0.309271	4.767090	-2.800631
H	-1.187458	4.644350	-3.460638
H	-0.690881	5.070147	-1.809665
H	0.335855	5.558596	-3.211062
O	4.255569	-0.657860	0.840334
C	4.317029	-0.811026	2.075985
C	4.937520	-2.019349	2.737604
H	5.628685	-2.529815	2.051932
H	5.451371	-1.733224	3.667785
H	4.125928	-2.718705	3.008925
O	3.806274	0.044463	2.946470
H	3.287805	0.784375	2.459356
O	4.697992	-1.408051	-2.425403
C	5.221543	-2.410945	-1.938850
O	5.305996	-2.650451	-0.619971
H	4.882527	-1.873302	-0.119050
C	5.844585	-3.523815	-2.756380
H	5.332233	-4.477852	-2.545484
H	5.768979	-3.288308	-3.826532
H	6.902792	-3.655184	-2.474023

Statistical Thermodynamic Analysis
Temperature= 298.150 Kelvin Pressure= 1.00000 Atm

===

SCF Energy= -6947.87503268 Predicted Change= -4.048755D-09
Zero-point correction (ZPE)= -6947.2111 0.66388
Internal Energy (U)= -6947.1554 0.71961
Enthalpy (H)= -6947.1544 0.72055
Gibbs Free Energy (G)= -6947.3079 0.56705

Entropy (S)= 0.00051484

Frequencies -- -193.8182 8.3548 18.3493

Supporting Information: 035-Selenylated-Cluster-RuII-2HOAc-1OAc-CsBr-Decarboxylation-TS-01-PBE.log

----

Using Gaussian 16: ES64L-G16RevA.03 25-Dec-2016

==

# pbepbe/gen/auto pseudo=read gfprint gfinput empiricaldispersion=gd3bj
scf=(direct,vshift=200,tight,maxcycle=300,yqc)
opt=(gdiis,maxcycle=250,calcfc,ts,noeigentest) freq=noraman
#N Geom=AllCheck Guess=TCheck SCRF=Check GenChk RPBEPBE/Gen/Auto Freq

----

Pointgroup= C1 Stoichiometry= C16H28B10BrCsO8RuSe C1[X(C16H28B10BrCsO8RuSe)]
#Atoms= 66
Charge = 0 Multiplicity = 1

SCF Energy= -6558.90929532 Predicted Change= -2.947247D-09

==

Optimization completed. {Found 2 times}

Item	Max Val.	Criteria	Pass?	RMS Val.	Criteria	Pass?
Force	0.00000 ||	0.00045	[YES]	0.00000 ||	0.00030	[YES]
Displ	0.00151 ||	0.00180	[YES]	0.00151 ||	0.00180	[YES]

Atomic Type	Coordinates (Angstroms) X	Y	Z
B	3.685896	0.265776	2.716167
B	3.557392	2.021565	2.998867
H	4.480054	2.684969	3.376890
H	4.699057	-0.354377	2.873042
B	1.902118	2.374212	3.550854
B	2.362321	2.700720	1.863269
B	2.092656	-0.460280	3.070647
B	2.554177	-0.115902	1.418615
H	1.859295	-1.570701	3.433226
H	2.303384	3.808477	1.415327
H	1.533910	3.265566	4.257663
C	0.981810	0.276878	1.963406
C	0.883994	1.947651	2.262953
B	2.696230	0.876520	4.072745
B	0.981373	0.843979	3.577052
H	0.008599	0.681781	4.246016
H	2.979876	0.701852	5.222885
B	3.459620	1.398280	1.337178
H	4.281722	1.587865	0.490030

B	1.733580	1.351579	0.893858
H	1.406210	1.710672	-0.317631
C	-0.521107	-0.848067	1.801849
O	-1.562231	-0.204189	1.950322
O	-0.111421	-1.995475	1.717976
C	-0.441370	2.648782	2.019127
H	-0.658027	3.311388	2.871333
H	-0.399151	3.252349	1.100407
H	-1.239074	1.896817	1.930555
Ru	0.526345	0.232063	-0.593079
Se	2.378843	-1.368959	-0.162692
C	3.942332	-0.751835	-1.144277
C	5.211615	-1.128107	-0.680079
C	3.791409	0.007525	-2.311238
C	6.349283	-0.712166	-1.384742
H	5.312853	-1.719426	0.235702
C	4.938143	0.410894	-3.012304
H	2.793459	0.290106	-2.656831
C	6.213884	0.056958	-2.550401
H	7.343200	-0.988487	-1.018303
H	4.829360	1.009107	-3.923041
H	7.104713	0.380330	-3.098327
O	-1.094848	1.622683	-1.358034
C	-0.495415	1.593820	-2.502540
C	-1.029156	2.329033	-3.698580
H	-1.810888	1.714681	-4.180152
H	-1.483212	3.284052	-3.389244
H	-0.227638	2.504946	-4.430819
O	0.574148	0.892306	-2.585559
Cs	-3.791110	-0.273520	-0.121237
Br	-0.800156	-1.715624	-1.604174
H	-1.287778	-3.402524	-0.056162
O	-1.539473	-4.266434	0.385080
C	-2.812058	-4.194021	0.795562
C	-3.215816	-5.427271	1.575621
H	-2.828685	-6.340068	1.095890
H	-2.772357	-5.373663	2.585488
H	-4.309942	-5.472596	1.665922
O	-3.562925	-3.238678	0.584560
O	-4.125615	2.809647	-0.291189
C	-3.509456	3.874293	-0.358699
O	-2.225553	3.969693	-0.750862
C	-4.093482	5.222069	0.000436
H	-3.911436	5.949187	-0.807781
H	-5.170733	5.125794	0.191379
H	-3.593013	5.611461	0.903977

H -1.878251 3.037417 -0.947477

Statistical Thermodynamic Analysis
Temperature= 298.150 Kelvin Pressure= 1.00000 Atm

==========

SCF Energy= -6558.90929532 Predicted Change= -2.947247D-09
Zero-point correction (ZPE)= -6558.4536 0.45568
Internal Energy (U)= -6558.4099 0.49936
Enthalpy (H)= -6558.4089 0.50030
Gibbs Free Energy (G)= -6558.5371 0.37218
Entropy (S)= 0.00042971

Frequencies -- -165.5708 14.0699 17.9993

Supporting Information: 035-Selenylated-Cluster-RuII-2HOAc-1OAc-CsBr-Decarboxylation-TS-02-PBE.log

---

Using Gaussian 16: ES64L-G16RevA.03 25-Dec-2016

==========

# pbepbe/gen/auto pseudo=read gfprint gfinput empiricaldispersion=gd3bj
scf=(direct,vshift=200,tight,maxcycle=300,yqc)
opt=(gdiis,maxcycle=250,calcfc,ts,noeigentest) freq=noraman
#N Geom=AllCheck Guess=TCheck SCRF=Check GenChk RPBEPBE/Gen/Auto Freq

---

Pointgroup= C1 Stoichiometry= C16H28B10BrCsO8RuSe C1[X(C16H28B10BrCsO8RuSe)]
#Atoms= 66
Charge = 0 Multiplicity = 1

SCF Energy= -6558.91105398 Predicted Change= -3.669981D-09

==========

Optimization completed. {Found 1 times}

Item	Max Val.	Criteria	Pass?	RMS Val.	Criteria	Pass?
Force	0.00002 ||	0.00045	[YES]	0.00000 ||	0.00030	[YES]
Displ	0.00531 ||	0.00180	[NO]	0.00531 ||	0.00180	[YES]

Atomic Type	Coordinates (Angstroms) X	Y	Z
B	3.467043	3.118517	0.218910
B	3.674430	3.451474	-1.521147
H	4.640870	4.003234	-1.963969
H	4.280308	3.410974	1.048941
B	2.064023	3.743007	-2.225644
B	2.863028	2.166008	-2.452431
B	1.716794	3.174639	0.575866
B	2.514348	1.639335	0.331606
H	1.190454	3.439428	1.611652

H	3.121743	1.770769	-3.551675
H	1.789740	4.417074	-3.174424
C	0.993669	1.936254	-0.406057
C	1.209980	2.290062	-2.036554
B	2.420626	4.322365	-0.585286
B	0.849404	3.560603	-0.932644
H	-0.233020	4.056128	-0.985396
H	2.465070	5.494784	-0.345491
B	3.728328	1.771304	-0.944191
H	4.703606	1.079540	-0.953058
B	2.135573	1.050514	-1.279950
H	2.094239	-0.181016	-1.716177
C	-0.726664	1.518738	0.325281
O	-1.562703	1.524177	-0.574933
O	-0.603012	1.483073	1.537776
C	0.140916	1.856788	-3.027251
H	0.414557	2.243214	-4.021293
H	0.079871	0.757307	-3.068768
H	-0.839476	2.251727	-2.730340
Ru	0.974929	-0.651971	-0.478722
Se	2.335960	0.001250	1.507134
C	4.135983	-0.691593	1.248794
C	5.195998	-0.062847	1.918831
C	4.350515	-1.818545	0.445480
C	6.497021	-0.558644	1.759140
H	5.011500	0.819367	2.540251
C	5.656805	-2.310284	0.300488
H	3.510595	-2.293088	-0.069201
C	6.728455	-1.681995	0.950926
H	7.330845	-0.062515	2.266255
H	5.834209	-3.188143	-0.329632
H	7.745990	-2.066751	0.828391
O	-0.161042	-1.568779	-2.197792
C	0.590348	-2.599865	-2.061561
C	0.414464	-3.839959	-2.894372
H	-0.279388	-4.528279	-2.379059
H	-0.006649	-3.584433	-3.878785
H	1.377026	-4.359932	-3.014928
O	1.505252	-2.563647	-1.153898
Cs	-3.183151	-0.964332	-1.187111
Br	-0.554046	-1.995124	1.104744
H	-1.613670	-0.766816	2.612696
O	-2.054421	-0.245709	3.360009
C	-3.172099	0.306323	2.917370
C	-3.741491	1.325386	3.872130
H	-3.530085	1.047586	4.915397

H	-3.235752	2.287150	3.672021
H	-4.821569	1.455719	3.713607
O	-3.685047	0.030966	1.814102
O	-6.002654	0.138116	-0.656258
C	-6.564128	0.870881	0.163067
O	-6.040395	1.211204	1.347923
C	-7.926255	1.494604	-0.056293
H	-7.845397	2.594373	-0.023571
H	-8.331579	1.179439	-1.027264
H	-8.613994	1.197349	0.753166
H	-5.131561	0.761796	1.459534

Statistical Thermodynamic Analysis
Temperature= 298.150 Kelvin Pressure= 1.00000 Atm
===
SCF Energy= -6558.91105398 Predicted Change= -3.669981D-09
Zero-point correction (ZPE)= -6558.4552 0.45577
Internal Energy (U)= -6558.4118 0.49919
Enthalpy (H)= -6558.4109 0.50014
Gibbs Free Energy (G)= -6558.5390 0.37199
Entropy (S)= 0.00042981

Frequencies -- -150.0928 5.5177 16.2006

Supporting Information: 035-Selenylated-Cluster-RuII-2HOAc-1OAc-CsBr-Decarboxylation-TS-03-PBE.log

----
Using Gaussian 16: ES64L-G16RevA.03 25-Dec-2016
===
# pbepbe/gen/auto pseudo=read gfprint gfinput empiricaldispersion=gd3bj
scf=(direct,vshift=200,tight,maxcycle=300,yqc)
opt=(gdiis,maxcycle=250,calcfc,ts,noeigentest) freq=noraman
#N Geom=AllCheck Guess=TCheck SCRF=Check GenChk RPBEPBE/Gen/Auto Freq

----
Pointgroup= C1 Stoichiometry= C16H28B10BrCsO8RuSe C1[X(C16H28B10BrCsO8RuSe)] #Atoms= 66
Charge = 0 Multiplicity = 1

SCF Energy= -6558.91268230 Predicted Change= -5.009962D-09
===
Optimization completed on the basis of negligible forces. {Found 2 times}

Item	Max Val.	Criteria	Pass?	RMS Val.	Criteria	Pass?
Force	0.00000 ||	0.00045	[YES]	0.00000 ||	0.00030	[YES]
Displ	0.00563 ||	0.00180	[NO]	0.00563 ||	0.00180	[YES]

Atomic Coordinates (Angstroms)
Type X Y Z

B	-4.326776	-2.070493	0.148985
B	-4.626096	-2.254871	-1.597616
H	-5.724301	-2.392164	-2.055222
H	-5.197001	-2.058051	0.972464
B	-3.236339	-3.112542	-2.311706
B	-3.382767	-1.343644	-2.494474
B	-2.726982	-2.787094	0.504714
B	-2.892502	-1.060492	0.306138
H	-2.350265	-3.257851	1.532397
H	-3.464117	-0.853772	-3.582823
H	-3.227309	-3.817557	-3.277300
C	-1.581425	-1.886721	-0.444889
C	-1.902828	-2.096443	-2.088083
B	-3.799261	-3.556399	-0.686933
B	-2.053381	-3.433209	-1.013022
H	-1.240172	-4.301445	-1.082465
H	-4.282994	-4.631844	-0.479692
B	-4.049576	-0.691182	-0.974642
H	-4.692029	0.317300	-0.963208
B	-2.299237	-0.621340	-1.293788
H	-1.745610	0.504156	-1.641278
C	0.218447	-2.252023	0.248961
O	0.970104	-2.504098	-0.678993
O	0.097883	-2.324676	1.465337
C	-0.746371	-2.074142	-3.074858
H	-1.164897	-2.146272	-4.090943
H	-0.176466	-1.136486	-2.982729
H	-0.067631	-2.918502	-2.896411
Ru	-0.545204	0.361697	-0.368858
Se	-2.122250	0.321437	1.560227
C	-3.446387	1.716485	1.261111
C	-4.698949	1.594178	1.880408
C	-3.128657	2.832249	0.476233
C	-5.658628	2.597116	1.687070
H	-4.931495	0.713945	2.488355
C	-4.095450	3.833555	0.297566
H	-2.144868	2.908720	0.004250
C	-5.358035	3.716548	0.896391
H	-6.644042	2.499988	2.154054
H	-3.857148	4.707721	-0.317409
H	-6.109968	4.498144	0.747602
O	0.900972	0.740899	-2.046619
C	0.696060	1.995272	-1.869336
C	1.335522	3.039793	-2.742050
H	1.196601	4.038718	-2.303819

H	2.409924	2.822451	-2.852927
H	0.875617	3.015094	-3.745528
O	-0.108602	2.344844	-0.924239
Cs	3.529714	-0.847676	-1.061302
Br	1.323383	0.718826	1.360441
H	1.507989	-2.335325	2.499259
O	2.151670	-2.360782	3.271235
C	3.408140	-2.293580	2.818738
C	4.407835	-2.243269	3.956285
H	4.367042	-1.246554	4.429874
H	4.155277	-2.983472	4.732116
H	5.422462	-2.420213	3.573351
O	3.722921	-2.258220	1.626603
O	4.107995	2.189730	-0.865765
C	3.793766	3.280855	-0.380772
O	2.756966	3.476362	0.443535
C	4.520882	4.575081	-0.681014
H	4.726460	5.129608	0.248593
H	5.456462	4.364156	-1.216708
H	3.879293	5.219367	-1.307692
H	2.297770	2.594003	0.634377

Statistical Thermodynamic Analysis
Temperature= 298.150 Kelvin   Pressure= 1.00000 Atm

==

SCF Energy=   -6558.91268230   Predicted Change= -5.009962D-09
Zero-point correction (ZPE)=   -6558.4569   0.45576
Internal Energy (U)=   -6558.4135   0.49914
Enthalpy (H)=   -6558.4125   0.50008
Gibbs Free Energy (G)=   -6558.5387   0.37398
Entropy (S)=   0.00042295

Frequencies --   -128.7249   15.7388   23.9921

Supporting Information: 035-Selenylated-Cluster-RuII-2HOAc-1OAc-CsBr-Decarboxylation-TS-04-PBE.log

----

Using Gaussian 16: ES64L-G16RevA.03 25-Dec-2016

==

# pbepbe/gen/auto pseudo=read gfprint gfinput empiricaldispersion=gd3bj
scf=(direct,vshift=200,tight,maxcycle=300,yqc)
opt=(gdiis,maxcycle=250,calcfc,ts,noeigentest) freq=noraman
#N Geom=AllCheck Guess=TCheck SCRF=Check GenChk RPBEPBE/Gen/Auto Freq

----

Pointgroup= C1   Stoichiometry= C16H28B10BrCsO8RuSe   C1[X(C16H28B10BrCsO8RuSe)]
#Atoms= 66
Charge = 0   Multiplicity = 1

SCF Energy= -6558.84109336   Predicted Change= -2.048597D-09

==

Optimization completed on the basis of negligible forces.   {Found 2 times}

Item	Max Val.	Criteria	Pass?	RMS Val.	Criteria	Pass?
Force	0.00000 ||	0.00045	[YES]	0.00000 ||	0.00030	[YES]
Displ	0.00393 ||	0.00180	[NO]	0.00393 ||	0.00180	[YES]

Atomic Type	Coordinates (Angstroms) X	Y	Z
B	3.568731	1.393327	2.542030
B	2.308487	1.194416	3.785833
H	2.233300	1.895157	4.753940
H	4.420469	2.233232	2.583070
B	1.883732	-0.530474	3.868596
B	0.838805	0.598154	2.982066
B	3.918161	-0.204484	1.846438
B	2.868150	0.906769	0.971328
H	4.940151	-0.582470	1.365775
H	2.897684	-0.284244	-2.133768
H	-0.306521	0.739504	3.300941
H	1.454119	-1.146866	4.797238
C	2.435785	-0.760237	1.130870
C	1.272600	-0.919349	2.327805
B	3.575024	-0.050753	3.587663
B	2.877833	-1.398137	2.670087
H	3.113229	-2.561549	2.748913
H	4.424452	-0.279072	4.398946
B	1.876452	1.787175	2.159863
H	1.480035	2.895674	1.932306
B	1.199516	0.435602	1.250102
H	0.361697	0.506937	0.411881
C	2.923061	-2.118911	-0.046296
O	2.298276	-3.192469	0.242073
O	3.894886	-1.787181	-0.705429
C	0.260165	-2.015280	2.069858
H	-0.569020	-1.962236	2.798879
H	-0.417291	-1.808697	1.163160
H	0.704282	-3.018652	2.078783
Ru	0.703238	-1.899251	-0.383541
O	1.233388	-2.135398	-2.370159
O	-1.033680	-0.863849	-1.117931
C	2.008435	-1.651664	-3.228992
C	-1.604795	-2.003901	-1.324656
O	2.829457	-0.631616	-3.081375

O	-0.890063	-3.047639	-1.060026
C	-3.020935	-2.125191	-1.777960
H	-3.647074	-2.192441	-0.868769
H	-3.322646	-1.229641	-2.342226
H	-3.159216	-3.037431	-2.377762
C	2.055332	-2.270935	-4.598744
H	2.151282	-1.491133	-5.370136
H	2.949866	-2.915944	-4.661220
H	1.160853	-2.885135	-4.768166
Se	3.388423	1.588489	-0.831660
C	1.821945	2.648624	-1.256868
C	1.674511	3.935715	-0.709073
C	0.873297	2.171351	-2.179533
C	0.578487	4.733914	-1.071557
H	2.422365	4.310232	-0.004651
C	-0.213033	2.979202	-2.554104
H	0.981766	1.172574	-2.609344
C	-0.364489	4.261530	-2.000284
H	0.476708	5.737490	-0.645128
H	-0.935878	2.605115	-3.287775
H	-1.201006	4.898745	-2.307842
Br	-3.188971	-1.255843	1.894260
Cs	-2.263658	1.834675	0.550108
O	-4.832006	0.975616	-0.823647
C	-5.749063	0.150279	-0.737589
O	-5.785251	-0.867678	0.124236
H	-4.934873	-0.891680	0.702252
C	-6.976782	0.175099	-1.627429
H	-7.892800	0.175315	-1.013796
H	-6.954996	1.062385	-2.275003
H	-7.006197	-0.737095	-2.247901

Statistical Thermodynamic Analysis
Temperature= 298.150 Kelvin Pressure= 1.00000 Atm
==
SCF Energy= -6558.84109336 Predicted Change= -2.048597D-09
Zero-point correction (ZPE)= -6558.3856 0.45547
Internal Energy (U)= -6558.3434 0.49760
Enthalpy (H)= -6558.3425 0.49855
Gibbs Free Energy (G)= -6558.4656 0.37541
Entropy (S)= 0.00041301

Frequencies -- -210.9647 14.5792 20.0208
Supporting Information: 035-Selenylated-Cluster-RuII-2HOAc-1OAc-CsBr-Decarboxylation-TS-05-PBE.log
----

Using Gaussian 16: ES64L-G16RevA.03 25-Dec-2016
==
pbepbe/gen/auto pseudo=read gfprint gfinput empiricaldispersion=gd3bj scf=(direct,vshift=200,tight,maxcycle=300,yqc) opt=(gdiis,maxcycle=250,calcfc,ts,noeigentest) freq=noraman
#N Geom=AllCheck Guess=TCheck SCRF=Check GenChk RPBEPBE/Gen/Auto Freq
----
Pointgroup= C1 Stoichiometry= C16H28B10BrCsO8RuSe C1[X(C16H28B10BrCsO8RuSe)]
#Atoms= 66
Charge = 0 Multiplicity = 1

SCF Energy= -6558.90440666 Predicted Change= -2.490406D-09
==
Optimization completed. {Found 2 times}

Item	Max Val.		Criteria	Pass?	RMS Val.		Criteria	Pass?
Force	0.00000	||	0.00045	[YES]	0.00000	||	0.00030	[YES]
Displ	0.00121	||	0.00180	[YES]	0.00121	||	0.00180	[YES]

Atomic Type	Coordinates (Angstroms) X	Y	Z
B	4.811910	-1.003244	-1.238047
B	5.307053	-1.942827	0.192929
H	6.449552	-2.046222	0.534564
H	5.579955	-0.404600	-1.935385
B	4.183388	-3.309513	0.373730
B	4.001569	-1.873392	1.410241
B	3.351415	-1.777896	-1.922623
B	3.205320	-0.375359	-0.889805
H	2.971089	-1.761971	-3.051816
H	4.086025	-1.960983	2.599872
H	4.396442	-4.379139	0.861958
C	2.151912	-1.712714	-0.671765
C	2.659611	-2.628332	0.671478
B	4.657056	-2.780267	-1.250952
B	2.957075	-3.206834	-0.919188
H	2.325079	-4.140817	-1.300474
H	5.309123	-3.490765	-1.959903
B	4.385802	-0.441156	0.419000
H	4.818691	0.556035	0.915522
B	2.692671	-0.902878	0.713267
H	1.987004	-0.205666	1.602243
C	0.491746	-1.866240	-1.444214
O	-0.148212	-2.804953	-0.965344
O	0.416433	-1.073323	-2.384932
C	1.618758	-3.363656	1.498111

H	1.954725	-4.401700	1.643723
H	1.505358	-2.884513	2.481704
H	0.653753	-3.360409	0.972275
Ru	0.748430	0.083366	0.429414
O	-0.082049	1.760480	1.375167
C	-1.071710	1.832413	0.512527
O	-1.020024	0.978692	-0.435583
C	-2.183787	2.799719	0.695707
H	-1.861242	3.663275	1.296169
H	-2.578431	3.121145	-0.280123
H	-2.994252	2.253683	1.228627
Se	2.125630	1.285307	-1.287423
C	3.282546	2.559959	-0.378538
C	4.466053	2.953006	-1.021241
C	2.918660	3.097338	0.863005
C	5.310711	3.879167	-0.394298
H	4.738483	2.522991	-1.990370
C	3.768596	4.029579	1.477071
H	1.987159	2.784294	1.342824
C	4.963601	4.417183	0.853940
H	6.243506	4.177295	-0.883440
H	3.493902	4.450042	2.450002
H	5.625960	5.139778	1.340896
Br	-4.192924	-0.040901	1.763806
Cs	-2.976069	-1.368990	-1.313350
O	-0.527238	-1.171941	1.626297
C	-0.942719	-0.952185	2.792784
O	-0.727597	0.174640	3.454362
C	-1.740449	-1.976188	3.534056
H	-2.804813	-1.738502	3.331912
H	-1.514439	-2.984540	3.159491
H	-1.562508	-1.901627	4.617279
H	-0.382585	0.862037	2.807050
O	-4.980357	0.848094	-1.957929
C	-5.552936	1.818800	-1.454637
O	-5.640328	2.060677	-0.142376
H	-5.193646	1.309518	0.389794
C	-6.262994	2.883317	-2.268914
H	-6.019752	2.764835	-3.333861
H	-7.353836	2.784089	-2.131375
H	-5.983402	3.890791	-1.920406

Statistical Thermodynamic Analysis
Temperature= 298.150 Kelvin Pressure= 1.00000 Atm

===

SCF Energy= -6558.90440666 Predicted Change= -2.490406D-09

Zero-point correction (ZPE)= -6558.4483 0.45600
Internal Energy (U)= -6558.4059 0.49843
Enthalpy (H)= -6558.4050 0.49938
Gibbs Free Energy (G)= -6558.5285 0.37581
Entropy (S)= 0.00041444

Frequencies -- -129.1327 13.4114 16.7066

Supporting Information: 035-Selenylated-Cluster-RuII-2HOAc-1OAc-CsBr-Decarboxylation-TS-06-PBE.log

---

Using Gaussian 16: ES64L-G16RevA.03 25-Dec-2016

==

# pbepbe/gen/auto pseudo=read gfprint gfinput empiricaldispersion=gd3bj
scf=(direct,vshift=200,tight,maxcycle=300,yqc)
opt=(gdiis,maxcycle=250,calcfc,ts,noeigentest) freq=noraman
#N Geom=AllCheck Guess=TCheck SCRF=Check GenChk RPBEPBE/Gen/Auto Freq

---

Pointgroup= C1 Stoichiometry= C16H28B10BrCsO8RuSe C1[X(C16H28B10BrCsO8RuSe)]
#Atoms= 66
Charge = 0 Multiplicity = 1

SCF Energy= -6558.89069422 Predicted Change= -3.267381D-07

==

Optimization completed. {Found 1 times}

Item	Max Val.	Criteria	Pass?	RMS Val.	Criteria	Pass?
Force	0.00002 ||	0.00045	[YES]	0.00000 ||	0.00030	[YES]
Displ	0.02500 ||	0.00180	[NO]	0.02500 ||	0.00180	[NO]

Atomic Type	Coordinates (Angstroms) X	Y	Z
B	-1.394611	3.474575	-1.486666
B	-1.786839	4.735944	-0.291166
H	-2.415308	5.719161	-0.561046
H	-1.727217	3.516342	-2.635956
B	-0.470815	4.777142	0.899728
B	-1.985939	3.952631	1.289243
B	0.161483	2.742722	-1.021192
B	-1.369887	1.913820	-0.642416
H	0.959587	2.229362	-1.740374
H	-2.648688	4.300519	2.222294
H	-0.091837	5.689713	1.573537
C	-0.059190	2.033600	0.540638
C	-0.465447	3.228338	1.641096
B	-0.098917	4.492702	-0.814024
B	0.732265	3.563114	0.432609

H	1.862206	3.636804	0.788597
H	0.516194	5.288953	-1.462032
B	-2.564338	3.133812	-0.172730
H	-3.731806	2.961184	-0.367021
B	-1.712740	2.215638	1.090713
H	-2.145265	1.431749	1.870086
C	1.221198	0.895614	1.139856
O	0.900063	0.088459	2.103734
O	2.363549	1.298106	0.841054
C	0.000790	3.039203	3.077414
H	-0.633175	3.645061	3.742507
H	-0.074829	1.980287	3.365796
H	1.046770	3.363313	3.186981
Ru	-0.232481	-0.463837	0.306062
O	-1.460329	-3.158954	-0.919224
C	-0.309167	-3.295268	-0.433759
O	0.246686	-2.466750	0.407589
C	0.561185	-4.473271	-0.828330
H	-0.053280	-5.295798	-1.221576
H	1.250018	-4.127120	-1.620266
H	1.164690	-4.826391	0.024184
Se	-1.721815	0.124567	-1.574365
C	-3.557966	-0.356017	-1.134940
C	-4.088383	-1.368795	-1.953644
C	-4.322561	0.178263	-0.087290
C	-5.381082	-1.852477	-1.714527
H	-3.482702	-1.792014	-2.761123
C	-5.621584	-0.301611	0.133895
H	-3.915444	0.954605	0.559206
C	-6.152533	-1.318291	-0.672719
H	-5.785173	-2.647867	-2.348886
H	-6.220452	0.127751	0.944629
H	-7.165235	-1.691984	-0.491436
O	-1.724985	-0.746873	1.813298
C	-2.638724	-1.604927	1.914503
O	-2.934201	-2.549133	1.057509
C	-3.544316	-1.571150	3.120434
H	-3.161954	-0.862549	3.867231
H	-4.551294	-1.259136	2.792500
H	-3.636760	-2.579102	3.556040
H	-2.314285	-2.587458	0.214549
Cs	3.384536	-1.693993	1.157875
Br	1.455473	-0.594059	-1.663664
O	5.095740	0.168728	-0.556890
C	4.923468	1.121142	-1.324557
O	3.880187	1.955505	-1.283912

H	3.235661	1.656989	-0.567273
C	5.873054	1.467215	-2.453430
H	5.423115	1.150335	-3.410911
H	6.032310	2.555645	-2.512134
H	6.829553	0.944313	-2.314936

Statistical Thermodynamic Analysis
Temperature= 298.150 Kelvin Pressure= 1.00000 Atm

==

SCF Energy= -6558.89069422 Predicted Change= -3.267381D-07
Zero-point correction (ZPE)= -6558.4336 0.45700
Internal Energy (U)= -6558.3915 0.49911
Enthalpy (H)= -6558.3906 0.50005
Gibbs Free Energy (G)= -6558.5108 0.37985
Entropy (S)= 0.00040317

Frequencies -- -194.9754 22.0992 25.5341

Supporting Information: 035-Selenylated-Cluster-RuII-2HOAc-1OAc-CsBr-Decarboxylation-TS-08-PBE.log

----

Using Gaussian 16: ES64L-G16RevA.03 25-Dec-2016

==

# pbepbe/gen/auto pseudo=read gfprint gfinput empiricaldispersion=gd3bj
scf=(direct,vshift=200,tight,maxcycle=300,yqc)
opt=(gdiis,maxcycle=250,calcfc,ts,noeigentest) freq=noraman
#N Geom=AllCheck Guess=TCheck SCRF=Check GenChk RPBEPBE/Gen/Auto Freq

----

Pointgroup= C1 Stoichiometry= C16H28B10BrCsO8RuSe C1[X(C16H28B10BrCsO8RuSe)]
#Atoms= 66
Charge = 0 Multiplicity = 1

SCF Energy= -6558.90750774 Predicted Change= -6.402026D-09

==

Optimization completed. {Found 1 times}

Item	Max Val.	Criteria	Pass?	RMS Val.	Criteria	Pass?
Force	0.00000 ||	0.00045	[YES]	0.00000 ||	0.00030	[YES]
Displ	0.00767 ||	0.00180	[NO]	0.00767 ||	0.00180	[YES]

Atomic Coordinates (Angstroms)

Type	X	Y	Z
B	4.733151	1.981321	0.788101
B	4.857236	2.756030	-0.815017
H	5.905165	3.065708	-1.303924
H	5.682319	1.709391	1.465991
B	3.414066	3.770352	-1.054309

B	3.516041	2.174621	-1.842713
B	3.187343	2.498822	1.523059
B	3.307041	0.948860	0.724319
H	2.914929	2.583787	2.680010
H	3.480535	2.081178	-3.034561
H	3.315506	4.759292	-1.718340
C	1.945373	1.948729	0.440575
C	2.094970	2.705018	-1.059797
B	4.140859	3.650630	0.561204
B	2.369587	3.606507	0.382106
H	1.555191	4.416635	0.696469
H	4.652430	4.603560	1.073980
B	4.330968	1.064349	-0.707194
H	4.959416	0.127086	-1.101650
B	2.558480	1.051391	-0.860213
H	2.089634	-0.006439	-1.543059
C	0.350799	1.823723	1.391736
O	-0.542189	2.470624	0.842219
O	0.580031	1.226831	2.438990
C	0.835040	3.000821	-1.856791
H	1.070635	3.770886	-2.607268
H	0.474609	2.092367	-2.363678
H	0.050809	3.367985	-1.180284
Ru	0.917837	-0.260726	-0.339043
Se	2.638949	-0.831138	1.418903
C	3.956173	-1.973997	0.556319
C	5.241729	-2.038998	1.114378
C	3.607817	-2.743305	-0.561533
C	6.202258	-2.870829	0.523518
H	5.497230	-1.429940	1.987478
C	4.576589	-3.579496	-1.137654
H	2.600635	-2.679642	-0.983321
C	5.870721	-3.641172	-0.601440
H	7.212089	-2.914154	0.943815
H	4.314730	-4.181507	-2.013911
H	6.623197	-4.290588	-1.059810
O	-0.870929	-0.918015	0.655957
C	-1.185502	-1.615300	1.657137
O	-2.376907	-2.162077	1.745560
C	-0.293266	-1.876869	2.833724
H	0.516541	-2.561697	2.528588
H	0.166539	-0.929229	3.153929
H	-0.863929	-2.338473	3.651073
H	-2.918455	-2.054804	0.860964
O	-0.543493	-0.046046	-1.985263
C	-0.410436	-1.305210	-2.209771

C	-1.248959	-2.044135	-3.200306
H	-2.224309	-2.262252	-2.718484
H	-1.431737	-1.418611	-4.088244
H	-0.768910	-2.991722	-3.486092
O	0.469447	-1.930270	-1.492298
Cs	-3.051467	1.395845	-0.562506
Br	-4.132600	-2.005058	-0.822403
O	-5.790996	1.119542	0.748288
C	-6.671665	0.342363	1.128043
O	-6.671888	-0.976053	0.900859
H	-5.852593	-1.231070	0.360176
C	-7.895293	0.775846	1.909320
H	-7.973780	0.197809	2.845011
H	-7.835694	1.849663	2.132954
H	-8.806604	0.567106	1.323324

Statistical Thermodynamic Analysis
Temperature= 298.150 Kelvin   Pressure= 1.00000 Atm

==

SCF Energy= -6558.90750774 Predicted Change= -6.402026D-09
Zero-point correction (ZPE)= -6558.4519 0.45560
Internal Energy (U)= -6558.4094 0.49807
Enthalpy (H)= -6558.4084 0.49901
Gibbs Free Energy (G)= -6558.5323 0.37511
Entropy (S)= 0.00041557

Frequencies -- -147.3234 7.3705 19.1043

Supporting Information: 035-Selenylated-Cluster-RuII-2HOAc-1OAc-CsBr-Decarboxylation-TS-09-PBE.log

----

Using Gaussian 16: ES64L-G16RevA.03 25-Dec-2016

==

# pbepbe/gen/auto pseudo=read gfprint gfinput empiricaldispersion=gd3bj
scf=(direct,vshift=200,tight,maxcycle=300,yqc)
opt=(gdiis,maxcycle=250,calcfc,ts,noeigentest) freq=noraman
#N Geom=AllCheck Guess=TCheck SCRF=Check GenChk RPBEPBE/Gen/Auto Freq

----

Pointgroup= C1 Stoichiometry= C16H28B10BrCsO8RuSe C1[X(C16H28B10BrCsO8RuSe)]
#Atoms= 66
Charge = 0 Multiplicity = 1

SCF Energy= -6558.91029580 Predicted Change= -1.030565D-08

==

Optimization completed. {Found 1 times}

Item	Max Val.	Criteria	Pass?	RMS Val.	Criteria	Pass?
Force	0.00002 ||	0.00045	[YES]	0.00000 ||	0.00030	[YES]

Displ 0.00225 || 0.00180 [NO] 0.00225 || 0.00180 [YES]

Atomic Type	Coordinates (Angstroms) X	Y	Z
B	2.424037	1.691930	-2.917683
B	4.013770	2.009803	-2.180761
H	4.694247	2.944861	-2.493382
H	1.929377	2.394685	-3.755276
B	4.755052	0.446167	-1.776797
B	3.994623	1.421449	-0.494142
B	2.175798	-0.077542	-2.934485
B	1.460964	0.899397	-1.677421
H	1.508104	-0.706615	-3.697299
H	4.638236	1.814302	0.433246
H	5.918779	0.178045	-1.716711
C	2.214460	-0.576820	-1.270365
C	3.741252	-0.262933	-0.615043
B	3.788711	0.592815	-3.255021
B	3.610726	-0.853236	-2.220634
H	3.953823	-1.974172	-2.438146
H	4.297651	0.488701	-4.334467
B	2.549719	2.194537	-1.190765
H	2.136949	3.234369	-0.770960
B	2.387357	0.718743	-0.195055
H	1.952911	0.934883	1.049403
C	1.319376	-2.326980	-1.156050
O	1.994410	-3.106204	-0.516593
O	0.345599	-2.235533	-1.897338
C	4.300205	-1.214848	0.429504
H	5.342347	-1.453801	0.167009
H	4.275074	-0.744988	1.423231
H	3.704334	-2.137469	0.455160
Ru	0.754087	-0.289682	0.847429
Se	-0.459470	0.811770	-1.055340
C	-0.829121	2.689187	-0.718150
C	-1.427739	3.050788	0.497010
C	-0.575231	3.647474	-1.710548
C	-1.761035	4.394623	0.723913
H	-1.621924	2.288678	1.254709
C	-0.908338	4.987884	-1.470316
H	-0.098986	3.357092	-2.651828
C	-1.501055	5.364002	-0.255743
H	-2.213226	4.682308	1.679235
H	-0.695796	5.740818	-2.236165
H	-1.754264	6.412878	-0.071538

O	1.872602	-1.490555	2.215050
C	1.555577	-1.759720	3.398292
C	2.284576	-2.839697	4.148317
H	3.239990	-3.062061	3.653920
H	2.444492	-2.545729	5.197308
H	1.661592	-3.751701	4.142600
O	0.584219	-1.174239	4.073168
Cs	-2.631842	-2.157787	-2.021852
Br	-1.207445	-1.971679	1.261671
H	0.138728	-0.481458	3.462905
O	-0.128839	0.719575	2.420373
C	0.432008	1.842254	2.901953
O	1.464117	2.366357	2.485246
C	-0.371638	2.397386	4.071425
H	-1.453645	2.326638	3.874601
H	-0.161344	1.798759	4.976136
H	-0.077724	3.439628	4.260792
O	-4.040100	-0.051189	-0.338510
C	-4.042186	0.676570	0.658107
O	-3.250955	0.496603	1.726796
H	-2.631021	-0.288699	1.562320
C	-4.911062	1.906531	0.799752
H	-4.302053	2.794504	0.550338
H	-5.758094	1.850385	0.101934
H	-5.267035	2.025017	1.834939

Statistical Thermodynamic Analysis
Temperature= 298.150 Kelvin Pressure= 1.00000 Atm
==
SCF Energy= -6558.91029580 Predicted Change= -1.030565D-08
Zero-point correction (ZPE)= -6558.4542 0.45603
Internal Energy (U)= -6558.4115 0.49875
Enthalpy (H)= -6558.4105 0.49970
Gibbs Free Energy (G)= -6558.5331 0.37715
Entropy (S)= 0.00041101

Frequencies -- -164.1397 19.0663 24.5340

Supporting Information: 035-Selenylated-Cluster-RuII-2HOAc-1OAc-CsBr-Decarboxylation-TS-10-PBE.log

----
Using Gaussian 16: ES64L-G16RevA.03 25-Dec-2016
==
# pbepbe/gen/auto pseudo=read gfprint gfinput empiricaldispersion=gd3bj
scf=(direct,vshift=200,tight,maxcycle=300,yqc)
opt=(gdiis,maxcycle=250,calcfc,ts,noeigentest) freq=noraman
#N Geom=AllCheck Guess=TCheck SCRF=Check GenChk RPBEPBE/Gen/Auto Freq

----
Pointgroup= C1 Stoichiometry= C16H28B10BrCsO8RuSe C1[X(C16H28B10BrCsO8RuSe)]
#Atoms= 66
Charge = 0 Multiplicity = 1

SCF Energy= -6558.90323200 Predicted Change= -6.413915D-09
==
Optimization completed. {Found 1 times}

Item	Max Val.	Criteria	Pass?	RMS Val.	Criteria	Pass?
Force	0.00001 ||	0.00045	[YES]	0.00000 ||	0.00030	[YES]
Displ	0.00373 ||	0.00180	[NO]	0.00373 ||	0.00180	[YES]

Atomic Type	Coordinates (Angstroms) X	Y	Z
B	-2.736319	3.353925	-1.175540
B	-2.212665	4.365166	0.192806
H	-2.887286	5.251653	0.633518
H	-3.794561	3.478171	-1.726787
B	-0.446815	4.532009	0.110073
B	-1.172639	3.387298	1.273105
B	-1.270706	2.860829	-2.078592
B	-2.001793	1.778593	-0.925154
H	-1.197965	2.576993	-3.235050
H	-0.996073	3.523361	2.447069
H	0.223816	5.439293	0.506139
C	-0.303205	1.959248	-0.944187
C	0.175719	2.970838	0.313053
B	-1.383328	4.495538	-1.392058
B	0.146984	3.579408	-1.283905
H	1.188011	3.802298	-1.821378
H	-1.446250	5.461058	-2.098167
B	-2.592506	2.653328	0.484521
H	-3.519562	2.269108	1.132919
B	-1.044257	1.769778	0.547627
H	-0.951883	0.770634	1.443930
C	0.994656	0.828677	-2.167189
O	2.129342	1.078357	-1.804921
O	0.276240	0.324411	-2.996724
C	1.543736	2.759197	0.933909
H	2.031371	3.737679	1.063372
H	1.453203	2.267655	1.911816
H	2.160301	2.133230	0.274885
Ru	-0.397353	-0.271606	0.203667
Se	-2.383974	-0.153272	-1.370296
C	-4.012678	-0.520796	-0.382774

C	-4.184758	-1.820166	0.122544
C	-5.042975	0.425838	-0.277221
C	-5.380159	-2.157743	0.772097
H	-3.389408	-2.562155	0.012241
C	-6.232222	0.079739	0.379734
H	-4.920534	1.426764	-0.698572
C	-6.401536	-1.206525	0.911308
H	-5.512001	-3.170736	1.167610
H	-7.029557	0.824003	0.473463
H	-7.331677	-1.470652	1.424238
O	0.307674	-1.936407	-1.037743
C	-0.161282	-3.104522	-1.040728
O	-0.977126	-3.597013	-0.139942
C	0.210680	-4.067527	-2.142914
H	-0.708004	-4.465240	-2.605308
H	0.811005	-3.561564	-2.912399
H	0.760369	-4.929823	-1.727066
H	-1.138764	-2.869059	0.626472
Cs	3.605430	-1.489253	-1.115630
Br	1.682295	-0.630197	1.753300
O	-1.239121	-1.701403	1.423193
C	-1.831402	-1.390381	2.586419
O	-1.915523	-0.250352	3.033781
C	-2.435617	-2.602469	3.279260
H	-2.435509	-2.435429	4.366238
H	-3.483280	-2.708560	2.946128
H	-1.896157	-3.532768	3.040860
O	5.316433	0.288863	0.641171
C	5.240523	1.182737	1.487959
O	4.165379	1.405210	2.257962
H	3.435414	0.747937	2.019123
C	6.347576	2.173537	1.774562
H	7.257069	1.888371	1.228761
H	6.029348	3.182040	1.458282
H	6.549709	2.222622	2.856907

Statistical Thermodynamic Analysis
Temperature= 298.150 Kelvin Pressure= 1.00000 Atm

===

SCF Energy= -6558.90323200 Predicted Change= -6.413915D-09
Zero-point correction (ZPE)= -6558.4483 0.45484
Internal Energy (U)= -6558.4053 0.49789
Enthalpy (H)= -6558.4043 0.49883
Gibbs Free Energy (G)= -6558.5290 0.37421
Entropy (S)= 0.00041799

Frequencies -- -135.8774 18.6156 22.9113
Supporting Information: 035-Selenylated-Cluster-RuII-2HOAc-1OAc-CsBr-Decarboxylation-TS-11-PBE.log
----
Using Gaussian 16: ES64L-G16RevA.03 25-Dec-2016
==
# pbepbe/gen/auto pseudo=read gfprint gfinput empiricaldispersion=gd3bj
scf=(direct,vshift=200,tight,maxcycle=300,yqc)
opt=(gdiis,maxcycle=250,calcfc,ts,noeigentest) freq=noraman
#N Geom=AllCheck Guess=TCheck SCRF=Check GenChk RPBEPBE/Gen/Auto Freq
----
Pointgroup= C1 Stoichiometry= C16H28B10BrCsO8RuSe C1[X(C16H28B10BrCsO8RuSe)]
#Atoms= 66
Charge = 0 Multiplicity = 1

SCF Energy= -6558.91268230 Predicted Change= -3.992762D-10
==
Optimization completed. {Found 2 times}

Item	Max Val.	Criteria	Pass?	RMS Val.	Criteria	Pass?
Force	0.00000 ||	0.00045	[YES]	0.00000 ||	0.00030	[YES]
Displ	0.00113 ||	0.00180	[YES]	0.00113 ||	0.00180	[YES]

Atomic Type	X	Y	Z
B	-4.326796	-2.070462	0.149035
B	-4.626111	-2.254882	-1.597564
H	-5.724315	-2.392179	-2.055170
H	-5.197025	-2.057994	0.972510
B	-3.236360	-3.112582	-2.311626
B	-3.382769	-1.343688	-2.494440
B	-2.727010	-2.787065	0.504786
B	-2.892516	-1.060466	0.306167
H	-2.350301	-3.257800	1.532482
H	-3.464116	-0.853842	-3.582800
H	-3.227334	-3.817620	-3.277203
C	-1.581439	-1.886724	-0.444838
C	-1.902841	-2.096485	-2.088025
B	-3.799289	-3.556394	-0.686845
B	-2.053408	-3.433223	-1.012932
H	-1.240203	-4.301466	-1.082349
H	-4.283030	-4.631830	-0.479577
B	-4.049583	-0.691183	-0.974627
H	-4.692031	0.317301	-0.963222
B	-2.299248	-0.621349	-1.293767
H	-1.745626	0.504127	-1.641302

C	0.218397	-2.252036	0.249090
O	0.970080	-2.504141	-0.678834
O	0.097815	-2.324622	1.465468
C	-0.746380	-2.074224	-3.074795
H	-1.164883	-2.146627	-4.090869
H	-0.176606	-1.136470	-2.982850
H	-0.067521	-2.918448	-2.896158
Ru	-0.545214	0.361697	-0.368882
Se	-2.122248	0.321500	1.560211
C	-3.446381	1.716542	1.261055
C	-4.698948	1.594254	1.880345
C	-3.128641	2.832288	0.476155
C	-5.658623	2.597190	1.686978
H	-4.931502	0.714034	2.488309
C	-4.095430	3.833593	0.297459
H	-2.144850	2.908744	0.004177
C	-5.358020	3.716604	0.896278
H	-6.644040	2.500075	2.153957
H	-3.857121	4.707745	-0.317533
H	-6.109948	4.498199	0.747466
O	0.900964	0.740846	-2.046646
C	0.696063	1.995224	-1.869397
C	1.335521	3.039716	-2.742148
H	1.196731	4.038640	-2.303875
H	2.409892	2.822294	-2.853156
H	0.875501	3.015086	-3.745576
O	-0.108598	2.344825	-0.924312
Cs	3.529741	-0.847816	-1.061296
Br	1.323400	0.718844	1.360381
O	2.756897	3.476314	0.443180
C	3.793842	3.280727	-0.380934
C	4.521078	4.574925	-0.681011
H	3.878609	5.220533	-1.305400
H	4.728873	5.128006	0.248983
H	5.455506	4.364164	-1.218769
O	4.108185	2.189549	-0.865722
H	2.297757	2.593956	0.634144
O	3.722829	-2.258054	1.626752
C	3.408041	-2.293315	2.818887
O	2.151567	-2.360462	3.271380
H	1.507897	-2.335073	2.499395
C	4.407734	-2.242875	3.956430
H	4.154896	-2.982613	4.732609
H	5.422303	-2.420355	3.573587
H	4.367317	-1.245912	4.429534

Statistical Thermodynamic Analysis
Temperature= 298.150 Kelvin Pressure= 1.00000 Atm
==
SCF Energy= -6558.91268230 Predicted Change= -3.992762D-10
Zero-point correction (ZPE)= -6558.4569 0.45575
Internal Energy (U)= -6558.4135 0.49914
Enthalpy (H)= -6558.4125 0.50008
Gibbs Free Energy (G)= -6558.5387 0.37397
Entropy (S)= 0.00042296

Frequencies -- -128.7319 15.7434 23.9825
Supporting Information: 035-Selenylated-Cluster-RuII-2HOAc-1OAc-CsBr-Decarboxylation-TS-12-PBE.log
----
Using Gaussian 16: ES64L-G16RevA.03 25-Dec-2016
==
# pbepbe/gen/auto pseudo=read gfprint gfinput empiricaldispersion=gd3bj
scf=(direct,vshift=200,tight,maxcycle=300,yqc)
opt=(gdiis,maxcycle=250,calcfc,ts,noeigentest) freq=noraman
#N Geom=AllCheck Guess=TCheck SCRF=Check GenChk RPBEPBE/Gen/Auto Freq
----
Pointgroup= C1 Stoichiometry= C16H28B10BrCsO8RuSe C1[X(C16H28B10BrCsO8RuSe)]
#Atoms= 66
Charge = 0 Multiplicity = 1

SCF Energy= -6558.91268229 Predicted Change= -3.012839D-09
==
Optimization completed. {Found 1 times}

Item	Max Val.	Criteria	Pass?	RMS Val.	Criteria	Pass?
Force	0.00000 ||	0.00045	[YES]	0.00000 ||	0.00030	[YES]
Displ	0.00403 ||	0.00180	[NO]	0.00403 ||	0.00180	[YES]

Atomic Type	Coordinates (Angstroms) X	Y	Z
B	-4.326809	-2.070494	0.149014
B	-4.626131	-2.254889	-1.597585
H	-5.724337	-2.392195	-2.055186
H	-5.197032	-2.058053	0.972495
B	-3.236379	-3.112560	-2.311670
B	-3.382808	-1.343659	-2.494461
B	-2.727007	-2.787081	0.504741
B	-2.892545	-1.060485	0.306149
H	-2.350291	-3.257826	1.532430
H	-3.464169	-0.853798	-3.582814
H	-3.227353	-3.817584	-3.277258

C	-1.581457	-1.886702	-0.444875
C	-1.902869	-2.096453	-2.088067
B	-3.799283	-3.556405	-0.686891
B	-2.053402	-3.433202	-1.012992
H	-1.240192	-4.301438	-1.082429
H	-4.283010	-4.631850	-0.479637
B	-4.049620	-0.691192	-0.974630
H	-4.692082	0.317284	-0.963208
B	-2.299281	-0.621351	-1.293778
H	-1.745636	0.504118	-1.641273
C	0.218502	-2.252038	0.249119
O	0.970168	-2.504127	-0.678815
O	0.097852	-2.324685	1.465477
C	-0.746412	-2.074169	-3.074842
H	-1.164885	-2.147041	-4.090895
H	-0.176912	-1.136217	-2.983199
H	-0.067297	-2.918123	-2.895913
Ru	-0.545209	0.361667	-0.368855
Se	-2.122257	0.321448	1.560220
C	-3.446369	1.716514	1.261080
C	-4.698937	1.594235	1.880370
C	-3.128617	2.832264	0.476191
C	-5.658600	2.597185	1.687014
H	-4.931501	0.714013	2.488326
C	-4.095393	3.833583	0.297506
H	-2.144825	2.908714	0.004213
C	-5.357984	3.716603	0.896324
H	-6.644018	2.500077	2.153992
H	-3.857074	4.707738	-0.317478
H	-6.109904	4.498208	0.747520
O	0.900975	0.740858	-2.046608
C	0.696047	1.995233	-1.869363
C	1.335451	3.039739	-2.742139
H	1.196900	4.038627	-2.303705
H	2.409763	2.822181	-2.853441
H	0.875170	3.015314	-3.745454
O	-0.108614	2.344821	-0.924276
Cs	3.529760	-0.847785	-1.061317
Br	1.323402	0.718831	1.360416
H	1.507948	-2.335030	2.499506
O	2.151648	-2.360385	3.271461
C	3.408099	-2.293261	2.818896
C	4.407852	-2.242803	3.956385
H	4.367280	-1.245926	4.429654
H	4.155188	-2.982710	4.732462
H	5.422429	-2.420054	3.573458

O	3.722818	-2.258051	1.626741
O	4.108070	2.189577	-0.865810
C	3.793754	3.280736	-0.380957
O	2.756899	3.476273	0.443278
H	2.297762	2.593906	0.634210
C	4.520877	4.574959	-0.681194
H	4.727436	5.128890	0.248560
H	5.455953	4.364108	-1.217796
H	3.878880	5.219782	-1.306888

Statistical Thermodynamic Analysis
Temperature= 298.150 Kelvin Pressure= 1.00000 Atm

==

SCF Energy= -6558.91268229 Predicted Change= -3.012839D-09
Zero-point correction (ZPE)= -6558.4569 0.45575
Internal Energy (U)= -6558.4135 0.49914
Enthalpy (H)= -6558.4125 0.50008
Gibbs Free Energy (G)= -6558.5387 0.37398
Entropy (S)= 0.00042296

Frequencies -- -128.7297 15.7464 23.9874

Supporting Information: 035-Selenylated-Cluster-RuII-2HOAc-1OAc-CsBr-Decarboxylation-TS-13-PBE.log

----

Using Gaussian 16: ES64L-G16RevA.03 25-Dec-2016

==

# pbepbe/gen/auto pseudo=read gfprint gfinput empiricaldispersion=gd3bj
scf=(direct,vshift=200,tight,maxcycle=300,yqc)
opt=(gdiis,maxcycle=250,calcfc,ts,noeigentest) freq=noraman
#N Geom=AllCheck Guess=TCheck SCRF=Check GenChk RPBEPBE/Gen/Auto Freq

----

Pointgroup= C1 Stoichiometry= C16H28B10BrCsO8RuSe C1[X(C16H28B10BrCsO8RuSe)]
#Atoms= 66
Charge = 0 Multiplicity = 1

SCF Energy= -6558.90378110 Predicted Change= -1.392513D-08

==

Optimization completed. {Found 1 times}

Item	Max Val.		Criteria	Pass?	RMS Val.		Criteria	Pass?
Force	0.00001	||	0.00045	[YES]	0.00000	||	0.00030	[YES]
Displ	0.00641	||	0.00180	[NO]	0.00641	||	0.00180	[YES]

Atomic Type	Coordinates (Angstroms) X	Y	Z
B	-2.538447	3.428194	-0.517459

B	-2.036128	4.125992	1.043110
H	-2.676767	4.962349	1.612939
H	-3.552366	3.726667	-1.081650
B	-0.258457	4.175655	1.081060
B	-1.130028	2.891433	1.965432
B	-1.056057	3.008000	-1.426049
B	-1.916133	1.783166	-0.541031
H	-0.925224	2.946319	-2.609493
H	-1.014287	2.795341	3.152726
H	0.443494	4.942635	1.671623
C	-0.213675	1.841983	-0.435952
C	0.252474	2.568055	1.015544
B	-1.106052	4.487692	-0.444716
B	0.351686	3.459089	-0.443854
H	1.434758	3.713697	-0.873772
H	-1.059019	5.569565	-0.956267
B	-2.541558	2.423993	0.979528
H	-3.536240	1.990098	1.481571
B	-1.061562	1.428572	0.958127
H	-1.137220	0.245857	1.642733
C	1.079631	0.893146	-1.802917
O	2.200688	0.897603	-1.318244
O	0.395274	0.690230	-2.771202
C	1.577625	2.166149	1.640082
H	2.353850	2.900483	1.378931
H	1.470706	2.137962	2.735413
H	1.876631	1.169556	1.289928
Ru	-0.502052	-0.530707	0.277069
Se	-2.469779	-0.002076	-1.290493
C	-4.225736	-0.074537	-0.448702
C	-5.255495	0.641436	-1.079134
C	-4.465225	-0.809024	0.718676
C	-6.539530	0.636153	-0.517439
H	-5.050581	1.214816	-1.988851
C	-5.756810	-0.812629	1.266950
H	-3.652541	-1.367664	1.190912
C	-6.791699	-0.090855	0.655497
H	-7.342012	1.205089	-0.998084
H	-5.948625	-1.382301	2.182288
H	-7.795502	-0.093342	1.092442
Br	-1.402931	-2.675410	1.299914
O	0.404331	-1.767446	-1.296174
C	-0.197490	-2.540359	-2.078739
O	-1.311621	-3.183219	-1.786433
H	-1.520379	-3.022643	-0.796668
C	0.327137	-2.769580	-3.470332

H	0.395952	-1.789257	-3.971687
H	1.345963	-3.189976	-3.421051
H	-0.322227	-3.448745	-4.039145
O	1.311626	-0.896008	1.237520
C	1.656394	-1.117546	2.492762
C	0.649709	-0.842383	3.598135
H	1.119360	-1.020337	4.576038
H	0.286840	0.198935	3.547447
H	-0.229712	-1.496778	3.480010
O	2.809593	-1.517240	2.753918
Cs	3.823991	-1.639033	-0.195664
O	5.363375	1.015536	-0.396403
C	5.210896	2.236616	-0.350517
O	4.059071	2.858798	-0.668193
H	3.382362	2.166291	-0.916367
C	6.276778	3.218740	0.077591
H	5.975409	3.702189	1.022948
H	7.231804	2.695849	0.220702
H	6.386532	4.018043	-0.673455

Statistical Thermodynamic Analysis
Temperature= 298.150 Kelvin Pressure= 1.00000 Atm

==

SCF Energy= -6558.90378110 Predicted Change= -1.392513D-08
Zero-point correction (ZPE)= -6558.4480 0.45569
Internal Energy (U)= -6558.4050 0.49873
Enthalpy (H)= -6558.4041 0.49967
Gibbs Free Energy (G)= -6558.5284 0.37531
Entropy (S)= 0.00041711

Frequencies -- -152.1109 18.5509 22.9365

Supporting Information: 035-Selenylated-Cluster-RuII-2HOAc-1OAc-CsBr-Decarboxylation-TS-14-PBE.log

----

Using Gaussian 16: ES64L-G16RevA.03 25-Dec-2016

==

pbepbe/gen/auto pseudo=read gfprint gfinput empiricaldispersion=gd3bj scf=(direct,vshift=200,tight,maxcycle=300,yqc) opt=(gdiis,maxcycle=250,calcfc,ts,noeigentest) freq=noraman
#N Geom=AllCheck Guess=TCheck SCRF=Check GenChk RPBEPBE/Gen/Auto Freq

----

Pointgroup= C1 Stoichiometry= C16H28B10BrCsO8RuSe C1[X(C16H28B10BrCsO8RuSe)] #Atoms= 66
Charge = 0 Multiplicity = 1

SCF Energy= -6558.91568213 Predicted Change= -4.345869D-08

==

Optimization completed. {Found 1 times}

Item	Max Val.		Criteria	Pass?	RMS Val.		Criteria	Pass?
Force	0.00000	||	0.00045	[YES]	0.00000	||	0.00030	[YES]
Displ	0.10915	||	0.00180	[NO]	0.10915	||	0.00180	[NO]

Atomic Type	Coordinates (Angstroms) X	Y	Z
B	3.665908	-0.906734	-2.581660
B	4.606116	-2.160698	-1.737692
H	5.762790	-2.366835	-1.970675
H	4.127392	-0.178168	-3.413608
B	3.512649	-3.507733	-1.357404
B	3.968784	-2.348194	-0.077117
B	1.967769	-1.474372	-2.679698
B	2.453587	-0.354048	-1.440757
H	1.154505	-1.181659	-3.501287
H	4.548851	-2.721543	0.899787
H	3.785623	-4.665834	-1.238814
C	1.443910	-1.669311	-1.032459
C	2.362559	-2.883634	-0.287498
B	3.303340	-2.627261	-2.879188
B	1.865089	-3.076138	-1.913908
H	1.034329	-3.897833	-2.155252
H	3.502744	-3.168156	-3.928990
B	4.061864	-0.732349	-0.827691
H	4.785419	0.115273	-0.394938
B	2.604834	-1.219325	0.084739
H	2.375101	-0.707652	1.286039
C	-0.679827	-1.715001	-1.135014
O	-1.076507	-2.670568	-0.492852
O	-0.963982	-0.871634	-1.958091
C	1.701778	-3.793274	0.735166
H	0.613021	-3.792139	0.591854
H	2.089384	-4.813620	0.590556
H	1.925168	-3.457761	1.756814
Ru	0.766463	-0.243333	0.816676
Se	1.476908	1.345560	-0.987651
C	3.046058	2.421336	-0.559250
C	3.780245	2.920063	-1.646761
C	3.416184	2.703550	0.760263
C	4.920227	3.697870	-1.403568
H	3.478643	2.684344	-2.672538
C	4.555864	3.490156	0.988675
H	2.822979	2.312769	1.593209

C	5.309057	3.983481	-0.086010
H	5.506051	4.076990	-2.247259
H	4.854416	3.711827	2.018657
H	6.200331	4.591063	0.101240
Br	0.853113	1.240705	2.856476
O	-1.298286	0.340306	0.703306
C	-2.005559	1.441377	0.664075
O	-3.251709	1.355426	0.844014
C	-1.331702	2.755598	0.361653
H	-0.958969	2.751880	-0.676454
H	-2.038144	3.588060	0.489915
H	-0.464602	2.877409	1.032952
O	0.174160	-1.784363	2.217222
C	-0.964523	-2.078553	2.642816
C	-1.141554	-3.189381	3.644028
H	-1.874851	-3.922917	3.268633
H	-0.180066	-3.682996	3.837951
H	-1.540264	-2.774424	4.585451
O	-2.084945	-1.479047	2.279343
Cs	-4.068127	-1.208344	-0.724038
H	-1.832870	-0.692371	1.640293
O	-5.481841	1.391887	-1.437522
C	-5.531550	2.598329	-1.169311
O	-4.813332	3.189208	-0.210648
H	-4.210259	2.490150	0.239803
C	-6.425812	3.579891	-1.898628
H	-5.814748	4.373234	-2.361369
H	-7.007369	3.058591	-2.671047
H	-7.106118	4.073738	-1.184465

Statistical Thermodynamic Analysis
Temperature= 298.150 Kelvin Pressure= 1.00000 Atm

===

SCF Energy= -6558.91568213 Predicted Change= -4.345869D-08
Zero-point correction (ZPE)= -6558.4594 0.45622
Internal Energy (U)= -6558.4167 0.49891
Enthalpy (H)= -6558.4158 0.49985
Gibbs Free Energy (G)= -6558.5416 0.37400
Entropy (S)= 0.00042212

Frequencies -- -82.6742 6.7195 13.6869

Supporting Information: 035-Selenylated-Cluster-RuII-2HOAc-1OAc-CsBr-Decarboxylation-TS-15-PBE.log

----

Using Gaussian 16: ES64L-G16RevA.03 25-Dec-2016

===

# pbepbe/gen/auto pseudo=read gfprint gfinput empiricaldispersion=gd3bj
scf=(direct,vshift=200,tight,maxcycle=300,yqc)
opt=(gdiis,maxcycle=250,calcfc,ts,noeigentest) freq=noraman
#N Geom=AllCheck Guess=TCheck SCRF=Check GenChk RPBEPBE/Gen/Auto Freq

---

Pointgroup= C1 Stoichiometry= C16H28B10BrCsO8RuSe C1[X(C16H28B10BrCsO8RuSe)]
#Atoms= 66
Charge = 0 Multiplicity = 1

SCF Energy= -6558.90490955 Predicted Change= -1.011158D-08

===

Optimization completed on the basis of negligible forces. {Found 2 times}

Item	Max Val.	Criteria	Pass?	RMS Val.	Criteria	Pass?
Force	0.00000 ||	0.00045	[YES]	0.00000 ||	0.00030	[YES]
Displ	0.00548 ||	0.00180	[NO]	0.00548 ||	0.00180	[YES]

Atomic Coordinates (Angstroms)

Type	X	Y	Z
B	-4.731673	1.030896	-0.002053
B	-4.550980	2.186952	1.343919
H	-5.393121	2.343876	2.181070
H	-5.690023	0.325711	-0.143242
B	-3.588518	3.560848	0.761213
B	-2.818397	2.308765	1.765759
B	-3.848293	1.690908	-1.411486
B	-3.110334	0.474849	-0.394970
H	-4.058551	1.490764	-2.567471
H	-2.330759	2.588249	2.821949
H	-3.619022	4.694224	1.141050
C	-2.196230	1.823085	-0.905664
C	-2.061053	2.930128	0.369884
B	-4.744462	2.784131	-0.334386
B	-3.131958	3.253921	-0.937207
H	-2.830850	4.118340	-1.699896
H	-5.712014	3.374211	-0.721692
B	-3.521395	0.742825	1.298723
H	-3.588577	-0.164480	2.074374
B	-1.927496	1.231430	0.661785
H	-0.864483	0.677524	1.255315
C	-1.066752	1.766131	-2.348306
O	-0.285748	2.716082	-2.356461
O	-1.420223	0.814415	-3.044678
C	-0.826304	3.810053	0.468005
H	-0.308619	3.815943	-0.502294
H	-1.146762	4.830770	0.727410

H	-0.144406	3.429020	1.240937
Ru	-0.244717	0.138188	-0.245677
Se	-2.227655	-1.242599	-0.985543
C	-3.015240	-2.388256	0.382092
C	-4.325847	-2.833421	0.146379
C	-2.312580	-2.790681	1.524334
C	-4.945235	-3.675627	1.079583
H	-4.865001	-2.508852	-0.749516
C	-2.939471	-3.641504	2.447247
H	-1.293944	-2.433502	1.694711
C	-4.252661	-4.081936	2.229992
H	-5.972416	-4.012337	0.905787
H	-2.395499	-3.953380	3.345123
H	-4.738496	-4.740511	2.957142
Br	1.081709	-1.444728	1.214688
O	0.991890	-0.551684	-1.862673
C	1.450187	-1.753981	-2.209759
O	2.647321	-1.920125	-2.492778
C	0.442683	-2.880846	-2.264171
H	-0.429471	-2.586105	-2.868926
H	0.915242	-3.777557	-2.689601
H	0.085025	-3.103677	-1.243549
O	1.224311	1.669018	0.113912
C	2.186046	1.986702	-0.638353
C	2.935784	3.269351	-0.373162
H	3.185988	3.759936	-1.326221
H	2.329488	3.940950	0.251112
H	3.873647	3.036347	0.161199
O	2.633111	1.261368	-1.629168
H	1.934433	0.412998	-1.813506
Cs	4.528943	-1.066618	-0.290662
O	3.988086	1.002960	1.905191
C	3.237938	1.534973	2.731521
O	2.027498	1.070248	3.059545
H	1.808025	0.282068	2.471637
C	3.572096	2.811353	3.470420
H	3.225449	2.766870	4.514501
H	4.653891	2.999523	3.428572
H	3.044358	3.651601	2.984789

Statistical Thermodynamic Analysis
Temperature= 298.150 Kelvin Pressure= 1.00000 Atm

===

SCF Energy= -6558.90490955 Predicted Change= -1.011158D-08
Zero-point correction (ZPE)= -6558.4502 0.45461
Internal Energy (U)= -6558.4079 0.49699

Enthalpy (H)= -6558.4069 0.49793
Gibbs Free Energy (G)= -6558.5292 0.37564
Entropy (S)= 0.00041016

Frequencies -- -183.7117 15.7749 20.3383

Supporting Information: 035-Selenylated-Cluster-RuII-2HOAc-1OAc-CsBr-Decarboxylation-TS-16-PBE.log

---

Using Gaussian 16: ES64L-G16RevA.03 25-Dec-2016

===

# pbepbe/gen/auto pseudo=read gfprint gfinput empiricaldispersion=gd3bj
scf=(direct,vshift=200,tight,maxcycle=300,yqc)
opt=(gdiis,maxcycle=250,calcfc,ts,noeigentest) freq=noraman
#N Geom=AllCheck Guess=TCheck SCRF=Check GenChk RPBEPBE/Gen/Auto Freq

---

Pointgroup= C1 Stoichiometry= C16H28B10BrCsO8RuSe C1[X(C16H28B10BrCsO8RuSe)]
#Atoms= 66
Charge = 0 Multiplicity = 1

SCF Energy= -6558.86176014 Predicted Change= -1.104489D-08

===

Optimization completed on the basis of negligible forces. {Found 2 times}

Item	Max Val.		Criteria	Pass?	RMS Val.		Criteria	Pass?
Force	0.00000	||	0.00045	[YES]	0.00000	||	0.00030	[YES]
Displ	0.00732	||	0.00180	[NO]	0.00732	||	0.00180	[YES]

Atomic Type	Coordinates (Angstroms) X	Y	Z
B	-3.509360	2.420243	-1.729161
B	-2.932431	4.032259	-1.263617
H	-3.646719	4.993862	-1.212636
H	-4.644860	2.174486	-2.020774
B	-1.244845	4.174105	-1.784797
B	-1.616558	3.819285	-0.091004
B	-2.170114	1.556548	-2.526532
B	-2.543605	1.214988	-0.840184
H	-2.259889	0.704933	-3.358094
H	-1.806605	-3.017577	-0.135172
H	-1.256748	4.555611	0.781107
H	-0.625099	5.153971	-2.082005
C	-0.907515	1.406699	-1.373152
C	-0.389570	2.970410	-0.927995
B	-2.405912	3.303347	-2.808751
B	-0.771852	2.650530	-2.562483
H	0.123542	2.614273	-3.347248

H	-2.720433	3.719316	-3.887675
B	-3.007373	2.728077	-0.042859
H	-3.759242	2.714999	0.888722
B	-1.356596	2.075291	0.154638
H	-0.869524	1.673032	1.158625
C	0.523751	0.208434	-2.244706
O	1.588349	0.196943	-1.531087
O	0.225027	0.026868	-3.400149
Cs	4.042028	-1.085164	-0.313629
C	1.071070	3.177831	-0.566089
H	1.148449	3.645458	0.426287
H	1.595771	2.212806	-0.546817
H	1.550741	3.833666	-1.309640
Ru	0.205998	-0.932354	-0.350713
O	-0.561531	-2.157907	-1.954515
O	-0.704393	-2.383722	1.053919
C	-1.400970	-3.083312	-1.989217
C	0.390723	-3.058547	1.024312
O	-2.053842	-3.569240	-0.939843
O	1.319082	-2.625038	0.238131
C	0.583870	-4.284453	1.864478
H	0.644139	-3.987967	2.926137
H	-0.286853	-4.952037	1.756320
H	1.504247	-4.815320	1.579911
C	-1.778037	-3.717168	-3.298134
H	-1.910017	-4.804495	-3.183802
H	-2.745429	-3.293354	-3.622626
H	-1.019750	-3.487289	-4.058340
Se	-3.270008	-0.566296	-0.321836
C	-3.653236	-0.244840	1.547174
C	-4.993291	-0.077860	1.933910
C	-2.630924	-0.209188	2.509928
C	-5.310123	0.134590	3.283336
H	-5.780034	-0.098552	1.173981
C	-2.955260	0.015884	3.855344
H	-1.588759	-0.346362	2.212081
C	-4.291617	0.185473	4.245959
H	-6.355786	0.271405	3.578875
H	-2.152914	0.057626	4.600013
H	-4.539344	0.359685	5.298403
Br	1.265735	0.131544	1.738127
O	4.809814	1.621414	0.818962
C	4.407391	2.644741	1.377753
O	3.183452	2.780072	1.911258
H	2.653803	1.938758	1.750410
C	5.225422	3.907427	1.529566

H	6.266607	3.718015	1.235540
H	4.800818	4.697452	0.885872
H	5.179460	4.275958	2.567040

Statistical Thermodynamic Analysis
Temperature= 298.150 Kelvin Pressure= 1.00000 Atm

===

SCF Energy= -6558.86176014 Predicted Change= -1.104489D-08
Zero-point correction (ZPE)= -6558.4046 0.45713
Internal Energy (U)= -6558.3617 0.50004
Enthalpy (H)= -6558.3607 0.50098
Gibbs Free Energy (G)= -6558.4857 0.37601
Entropy (S)= 0.00041917

Frequencies -- -198.3226 15.8659 22.7847
Supporting Information: 035-Selenylated-Cluster-RuII-2HOAc-1OAc-CsBr-Decarboxylation-TS-17-PBE.log

---

Using Gaussian 16: ES64L-G16RevA.03 25-Dec-2016

===

# pbepbe/gen/auto pseudo=read gfprint gfinput empiricaldispersion=gd3bj
scf=(direct,vshift=200,tight,maxcycle=300,yqc)
opt=(gdiis,maxcycle=250,calcfc,ts,noeigentest) freq=noraman
#N Geom=AllCheck Guess=TCheck SCRF=Check GenChk RPBEPBE/Gen/Auto Freq

---

Pointgroup= C1 Stoichiometry= C16H28B10BrCsO8RuSe C1[X(C16H28B10BrCsO8RuSe)]
#Atoms= 66
Charge = 0 Multiplicity = 1

SCF Energy= -6558.86176015 Predicted Change= -1.683616D-09

===

Optimization completed. {Found 1 times}

Item	Max Val.	Criteria	Pass?	RMS Val.	Criteria	Pass?
Force	0.00000 ||	0.00045	[YES]	0.00000 ||	0.00030	[YES]
Displ	0.00303 ||	0.00180	[NO]	0.00303 ||	0.00180	[YES]

Atomic Type	Coordinates (Angstroms) X	Y	Z
B	-3.509299	2.420186	-1.729192
B	-2.932409	4.032225	-1.263682
H	-3.646719	4.993813	-1.212728
H	-4.644792	2.174396	-2.020807
B	-1.244825	4.174097	-1.784853
B	-1.616537	3.819306	-0.091053
B	-2.170025	1.556504	-2.526535

B	-2.543522	1.214978	-0.840184
H	-2.259771	0.704865	-3.358077
H	-1.806564	-3.017707	-0.135372
H	-1.256753	4.555662	0.781045
H	-0.625100	5.153971	-2.082078
C	-0.907437	1.406704	-1.373138
C	-0.389526	2.970444	-0.928015
B	-2.405861	3.303289	-2.808795
B	-0.771786	2.650513	-2.562496
H	0.123615	2.614254	-3.347252
H	-2.720383	3.719226	-3.887732
B	-3.007329	2.728071	-0.042893
H	-3.759205	2.715006	0.888682
B	-1.356538	2.075321	0.154630
H	-0.869473	1.673086	1.158634
C	0.523898	0.208423	-2.244637
O	1.588450	0.196953	-1.530940
O	0.225261	0.026912	-3.400113
Cs	4.042056	-1.085140	-0.313347
C	1.071109	3.177926	-0.566123
H	1.148483	3.645464	0.426296
H	1.595879	2.212934	-0.546964
H	1.550710	3.833862	-1.309630
Ru	0.206052	-0.932413	-0.350743
O	-0.561467	-2.157875	-1.954633
O	-0.704424	-2.383877	1.053728
C	-1.400922	-3.083263	-1.989426
C	0.390694	-3.058700	1.024183
O	-2.053804	-3.569290	-0.940102
O	1.319120	-2.625129	0.238115
C	0.583643	-4.284796	1.864117
H	0.641693	-3.988799	2.926026
H	-0.286225	-4.953208	1.754075
H	1.505011	-4.814637	1.580864
C	-1.778022	-3.716950	-3.298414
H	-1.910556	-4.804210	-3.184116
H	-2.745135	-3.292646	-3.623103
H	-1.019485	-3.487419	-4.058478
Se	-3.269854	-0.566325	-0.321797
C	-3.653121	-0.244840	1.547202
C	-4.993171	-0.077739	1.933905
C	-2.630827	-0.209269	2.509977
C	-5.310011	0.134758	3.283322
H	-5.779899	-0.098372	1.173960
C	-2.955170	0.015845	3.855384
H	-1.588674	-0.346542	2.212143

C	-4.291520	0.185561	4.245966
H	-6.355667	0.271671	3.578838
H	-2.152836	0.057525	4.600069
H	-4.539255	0.359810	5.298402
Br	1.265640	0.131368	1.738225
O	4.809285	1.621978	0.818323
C	4.406899	2.645060	1.377591
O	3.183131	2.780027	1.911582
H	2.653527	1.938716	1.750570
C	5.224725	3.907890	1.529300
H	6.266287	3.718217	1.236767
H	4.800990	4.697186	0.884122
H	5.177362	4.277560	2.566292

Statistical Thermodynamic Analysis
Temperature= 298.150 Kelvin Pressure= 1.00000 Atm

==

SCF Energy= -6558.86176015 Predicted Change= -1.683616D-09
Zero-point correction (ZPE)= -6558.4046 0.45713
Internal Energy (U)= -6558.3617 0.50004
Enthalpy (H)= -6558.3607 0.50098
Gibbs Free Energy (G)= -6558.4857 0.37601
Entropy (S)= 0.00041915

Frequencies -- -198.3219 15.8714 22.7988

Supporting Information: 035-Selenylated-Cluster-RuII-2HOAc-1OAc-CsBr-Decarboxylation-TS-18-PBE.log

----

Using Gaussian 16: ES64L-G16RevA.03 25-Dec-2016

==

# pbepbe/gen/auto pseudo=read gfprint gfinput empiricaldispersion=gd3bj
scf=(direct,vshift=200,tight,maxcycle=300,yqc)
opt=(gdiis,maxcycle=250,calcfc,ts,noeigentest) freq=noraman
#N Geom=AllCheck Guess=TCheck SCRF=Check GenChk RPBEPBE/Gen/Auto Freq

----

Pointgroup= C1 Stoichiometry= C16H28B10BrCsO8RuSe C1[X(C16H28B10BrCsO8RuSe)] #Atoms= 66
Charge = 0 Multiplicity = 1

SCF Energy= -6558.84419888 Predicted Change= -6.955270D-09

==

Optimization completed. {Found 2 times}

Item	Max Val.	Criteria	Pass?	RMS Val.	Criteria	Pass?
Force	0.00001 ||	0.00045	[YES]	0.00000 ||	0.00030	[YES]
Displ	0.00178 ||	0.00180	[YES]	0.00178 ||	0.00180	[YES]

Atomic Type	Coordinates (Angstroms) X	Y	Z
B	-3.370153	1.372961	-2.365632
B	-2.196807	0.976635	-3.646253
H	-2.061101	1.642772	-4.632589
H	-4.103670	2.318812	-2.407968
B	-2.006206	-0.792326	-3.699539
B	-0.790332	0.204052	-2.881034
B	-3.903128	-0.141148	-1.621516
B	-2.687262	0.833983	-0.793534
H	-4.951837	-0.367477	-1.107870
H	0.337211	-0.528434	2.476480
H	0.351218	0.189560	-3.239457
H	-1.701801	-1.480647	-4.627355
C	-2.484260	-0.875591	-0.927996
C	-1.387300	-1.222610	-2.173602
B	-3.605006	-0.084049	-3.373090
B	-3.054619	-1.486826	-2.434436
H	-3.448120	-2.610359	-2.472131
H	-4.509082	-0.217454	-4.145787
B	-1.629542	1.538377	-2.052013
H	-1.077139	2.590360	-1.887732
B	-1.100260	0.133849	-1.130958
H	-0.203520	0.133828	-0.350884
C	-3.195894	-2.167608	0.258635
O	-2.534552	-3.244468	0.130824
O	-4.184430	-1.753433	0.809772
C	-0.513042	-2.434452	-1.913519
H	0.263787	-2.515398	-2.694582
H	0.242930	-2.274003	-1.055791
H	-1.084742	-3.369254	-1.852503
Ru	-0.804025	-2.100716	0.537786
O	-1.636090	-1.822926	2.404354
O	1.080389	-1.199278	1.223203
C	-1.340857	-0.972982	3.281643
C	1.528627	-2.401817	1.472563
O	-0.230864	-0.251543	3.278537
O	0.689378	-3.353370	1.287432
C	2.936265	-2.643372	1.893737
H	3.576131	-2.487335	1.005285
H	3.238468	-1.910660	2.659978
H	3.060788	-3.670315	2.265542
C	-2.292306	-0.696484	4.403636
H	-2.912105	0.169353	4.105506
H	-2.955681	-1.558802	4.555336

H	-1.752189	-0.440528	5.327232
Se	-3.010963	1.543830	1.049965
C	-1.657551	2.927800	1.081088
C	-1.767772	4.061266	0.253893
C	-0.582128	2.831102	1.983765
C	-0.793375	5.068949	0.303800
H	-2.616230	4.146160	-0.431224
C	0.381610	3.852524	2.046207
H	-0.499254	1.957790	2.636968
C	0.284867	4.969351	1.199528
H	-0.888427	5.944351	-0.347391
H	1.204578	3.776019	2.766264
H	1.030474	5.769906	1.255239
Br	2.905466	-1.492657	-1.759185
Cs	2.159477	1.862918	-0.922216
O	4.710155	1.098887	0.525689
C	5.514254	0.171942	0.671143
O	5.452851	-1.001929	0.036879
H	4.630835	-1.043343	-0.581966
C	6.707177	0.242484	1.604800
H	7.641235	0.127643	1.029111
H	6.713572	1.204133	2.136006
H	6.672939	-0.588936	2.328873

Statistical Thermodynamic Analysis
Temperature= 298.150 Kelvin Pressure= 1.00000 Atm

===

SCF Energy= -6558.84419888 Predicted Change= -6.955270D-09
Zero-point correction (ZPE)= -6558.3880 0.45612
Internal Energy (U)= -6558.3461 0.49806
Enthalpy (H)= -6558.3451 0.49901
Gibbs Free Energy (G)= -6558.4679 0.37628
Entropy (S)= 0.00041164

Frequencies -- -184.3370 10.9872 18.8970

Supporting Information: 035-Selenylated-Cluster-RuII-2HOAc-1OAc-CsBr-Decarboxylation-TS-19-PBE.log

----

Using Gaussian 16: ES64L-G16RevA.03 25-Dec-2016

===

# pbepbe/gen/auto pseudo=read gfprint gfinput empiricaldispersion=gd3bj
scf=(direct,vshift=200,tight,maxcycle=300,yqc)
opt=(gdiis,maxcycle=250,calcfc,ts,noeigentest) freq=noraman
#N Geom=AllCheck Guess=TCheck SCRF=Check GenChk RPBEPBE/Gen/Auto Freq

----

Pointgroup= C1 Stoichiometry= C16H28B10BrCsO8RuSe C1[X(C16H28B10BrCsO8RuSe)]
#Atoms= 66
Charge = 0 Multiplicity = 1

SCF Energy= -6558.90447471 Predicted Change= -3.411588D-09

===

Optimization completed on the basis of negligible forces. {Found 2 times}

Item	Max Val.	Criteria	Pass?	RMS Val.	Criteria	Pass?
Force	0.00000 ||	0.00045	[YES]	0.00000 ||	0.00030	[YES]
Displ	0.00522 ||	0.00180	[NO]	0.00522 ||	0.00180	[YES]

Atomic Type	Coordinates (Angstroms) X	Y	Z
B	-4.520848	2.232796	-0.227731
B	-4.335514	2.985541	1.377437
H	-5.269244	3.348247	2.032696
H	-5.582074	2.026516	-0.743344
B	-2.814829	3.907809	1.384104
B	-2.882808	2.310664	2.167658
B	-3.085788	2.667658	-1.204100
B	-3.175666	1.112785	-0.409991
H	-3.002076	2.756940	-2.389504
H	-2.657244	2.190711	3.335922
H	-2.545314	4.879665	2.024996
C	-1.722398	2.020672	-0.351003
C	-1.575365	2.770265	1.169369
B	-3.796732	3.857873	-0.091433
B	-2.023344	3.702556	-0.202453
H	-1.224060	4.467309	-0.642072
H	-4.323417	4.847994	-0.509658
B	-3.934970	1.267240	1.174610
H	-4.545634	0.362971	1.662196
B	-2.164869	1.153437	1.033079
H	-1.548857	0.127563	1.621105
C	-0.332693	1.817308	-1.533251
O	0.669230	2.431037	-1.158588
O	-0.738790	1.209635	-2.524425
C	-0.188736	2.998889	1.745898
H	-0.114347	4.044511	2.081617
H	-0.019660	2.335778	2.607068
H	0.571873	2.807652	0.976083
Ru	-0.773729	-0.297043	0.138055
O	-0.402737	-2.290874	0.653256
C	0.280252	-2.517626	-0.446533
O	0.359506	-1.525445	-1.247184

C	0.926304	-3.834347	-0.688401
H	0.438231	-4.623465	-0.097282
H	0.903297	-4.077269	-1.762064
H	1.988227	-3.736568	-0.371715
Se	-2.819651	-0.684066	-1.262942
C	-4.111059	-1.708288	-0.226027
C	-5.467921	-1.590270	-0.563069
C	-3.690572	-2.568162	0.796915
C	-6.419195	-2.325714	0.156933
H	-5.782217	-0.912675	-1.363355
C	-4.651666	-3.305036	1.505460
H	-2.627602	-2.653133	1.039012
C	-6.012685	-3.182219	1.190967
H	-7.480666	-2.224882	-0.090778
H	-4.331223	-3.974531	2.310427
H	-6.758414	-3.754374	1.751794
Br	3.937806	-2.206429	0.380892
Cs	2.938211	0.393157	-1.830767
O	1.085636	0.260789	1.079999
C	1.590245	-0.264265	2.107006
O	1.103832	-1.345008	2.695028
C	2.789558	0.329390	2.775339
H	3.642691	-0.344497	2.580137
H	3.005542	1.323546	2.361789
H	2.629475	0.379002	3.864790
H	0.450282	-1.779840	2.068129
O	5.086824	1.455886	0.087198
C	5.925557	1.127302	0.931745
O	6.080485	-0.110082	1.414577
H	5.399442	-0.746160	0.987287
C	6.911523	2.097801	1.552755
H	7.944179	1.773912	1.339082
H	6.746702	3.107647	1.152822
H	6.797541	2.103456	2.649931

Statistical Thermodynamic Analysis
Temperature= 298.150 Kelvin Pressure= 1.00000 Atm

===

SCF Energy= -6558.90447471 Predicted Change= -3.411588D-09
Zero-point correction (ZPE)= -6558.4484 0.45601
Internal Energy (U)= -6558.4060 0.49847
Enthalpy (H)= -6558.4050 0.49941
Gibbs Free Energy (G)= -6558.5282 0.37623
Entropy (S)= 0.00041317

Frequencies -- -144.2020 16.6915 20.4495

Supporting Information: 035-Selenylated-Cluster-RuII-2HOAc-1OAc-CsBr-Decarboxylation-TS-20-PBE.log

---

Using Gaussian 16: ES64L-G16RevA.03 25-Dec-2016

===

# pbepbe/gen/auto pseudo=read gfprint gfinput empiricaldispersion=gd3bj
scf=(direct,vshift=200,tight,maxcycle=300,yqc)
opt=(gdiis,maxcycle=250,calcfc,ts,noeigentest) freq=noraman
#N Geom=AllCheck Guess=TCheck SCRF=Check GenChk RPBEPBE/Gen/Auto Freq

---

Pointgroup= C1 Stoichiometry= C16H28B10BrCsO8RuSe C1[X(C16H28B10BrCsO8RuSe)]
#Atoms= 66
Charge = 0 Multiplicity = 1

SCF Energy= -6558.89659906 Predicted Change= -2.108000D-09

===

Optimization completed on the basis of negligible forces. {Found 2 times}

Item	Max Val.	Criteria	Pass?	RMS Val.	Criteria	Pass?
Force	0.00000 ||	0.00045	[YES]	0.00000 ||	0.00030	[YES]
Displ	0.00349 ||	0.00180	[NO]	0.00349 ||	0.00180	[YES]

Atomic Type	Coordinates (Angstroms) X	Y	Z
B	-3.855758	2.734300	0.020494
B	-3.973227	2.924975	1.787592
H	-4.973447	3.293569	2.331805
H	-4.764498	2.942479	-0.731215
B	-2.378638	3.423152	2.399477
B	-2.904646	1.729007	2.573577
B	-2.164854	3.080545	-0.454498
B	-2.708283	1.431632	-0.263965
H	-1.764154	3.486824	-1.500469
H	-3.012465	1.229269	3.654333
H	-2.132607	4.078747	3.367584
C	-1.194388	1.909836	0.382876
C	-1.326862	2.140597	2.064240
B	-2.937967	4.031791	0.831284
B	-1.243777	3.501238	1.020881
H	-0.247670	4.154358	1.027701
H	-3.170951	5.195212	0.675248
B	-3.815686	1.292363	1.101260
H	-4.668512	0.455303	1.126805
B	-2.109291	0.818780	1.278834
H	-1.791798	-0.433956	1.562737
C	0.454766	1.793643	-0.550823

O	1.395393	2.019753	0.228234
O	0.232628	1.710139	-1.747429
C	-0.122043	1.862382	2.946735
H	0.037303	2.728330	3.607341
H	-0.301379	0.971057	3.566043
H	0.772306	1.704080	2.329587
Ru	-0.719745	-0.509283	0.192778
O	-0.711155	-2.596386	0.017259
C	0.208941	-2.577227	-0.920697
O	0.574842	-1.405384	-1.285812
C	0.806520	-3.829136	-1.439592
H	0.137031	-4.685559	-1.270967
H	1.054463	-3.725731	-2.507417
H	1.757987	-3.979119	-0.864459
Se	-2.410829	-0.047051	-1.604968
C	-4.021980	-1.068359	-1.216882
C	-5.242871	-0.589764	-1.715466
C	-3.951862	-2.266413	-0.494449
C	-6.416337	-1.312203	-1.459954
H	-5.281735	0.349165	-2.276918
C	-5.132762	-2.984322	-0.251746
H	-2.988022	-2.627815	-0.125551
C	-6.362574	-2.507968	-0.728202
H	-7.374363	-0.935755	-1.832335
H	-5.087384	-3.919162	0.316378
H	-7.281182	-3.069104	-0.530122
Br	3.580426	-3.297917	0.703421
Cs	3.567339	-0.198458	-0.904422
O	0.903027	-0.680141	1.600485
C	1.068800	-1.601702	2.444544
O	0.310038	-2.683452	2.512027
C	2.169224	-1.538987	3.450786
H	3.012304	-2.099820	2.988814
H	2.469112	-0.497827	3.636443
H	1.877099	-2.045892	4.382692
H	-0.213611	-2.755900	1.657973
O	4.410414	2.763038	-1.181586
C	4.048277	3.920000	-0.959407
O	2.886250	4.238704	-0.364849
H	2.388366	3.386617	-0.172318
C	4.849429	5.148883	-1.327374
H	4.978465	5.799525	-0.446726
H	5.828571	4.851569	-1.726136
H	4.302629	5.735401	-2.085549

Statistical Thermodynamic Analysis

Temperature= 298.150 Kelvin Pressure= 1.00000 Atm

==

SCF Energy= -6558.89659906 Predicted Change= -2.108000D-09
Zero-point correction (ZPE)= -6558.4400 0.45655
Internal Energy (U)= -6558.3977 0.49886
Enthalpy (H)= -6558.3967 0.49981
Gibbs Free Energy (G)= -6558.5203 0.37621
Entropy (S)= 0.00041454

Frequencies -- -151.8279 11.0627 16.4651

Supporting Information: 035-Selenylated-Cluster-RuII-2HOAc-1OAc-CsBr-Decarboxylation-TS-21-PBE.log

----

Using Gaussian 16: ES64L-G16RevA.03 25-Dec-2016

==

# pbepbe/gen/auto pseudo=read gfprint gfinput empiricaldispersion=gd3bj
scf=(direct,vshift=200,tight,maxcycle=300,yqc)
opt=(gdiis,maxcycle=250,calcfc,ts,noeigentest) freq=noraman
#N Geom=AllCheck Guess=TCheck SCRF=Check GenChk RPBEPBE/Gen/Auto Freq

----

Pointgroup= C1 Stoichiometry= C16H28B10BrCsO8RuSe C1[X(C16H28B10BrCsO8RuSe)]
#Atoms= 66
Charge = 0 Multiplicity = 1

SCF Energy= -6558.89631055 Predicted Change= -4.357232D-09

==

Optimization completed. {Found 2 times}

Item	Max Val.		Criteria	Pass?	RMS Val.		Criteria	Pass?
Force	0.00001	||	0.00045	[YES]	0.00000	||	0.00030	[YES]
Displ	0.00175	||	0.00180	[YES]	0.00175	||	0.00180	[YES]

Atomic Type	Coordinates (Angstroms) X	Y	Z
B	-4.324944	-1.853327	0.312555
B	-4.467542	-2.519278	-1.335367
H	-5.523072	-2.720302	-1.862553
H	-5.266802	-1.549990	0.987271
B	-3.097055	-3.620150	-1.607490
B	-3.063332	-1.980285	-2.298745
B	-2.839972	-2.524752	1.046737
B	-2.830501	-0.921492	0.346400
H	-2.602942	-2.700705	2.201028
H	-2.989397	-1.814062	-3.480662
H	-3.049555	-4.574687	-2.324695
C	-1.533745	-2.000106	0.033542

C	-1.700690	-2.660774	-1.526427
B	-3.848859	-3.543807	-0.002985
B	-2.072303	-3.616696	-0.146191
H	-1.324552	-4.499251	0.136238
H	-4.436967	-4.486317	0.442453
B	-3.819027	-0.880930	-1.114620
H	-4.366737	0.121560	-1.465435
B	-2.048234	-0.994192	-1.231041
H	-1.409957	-0.006941	-1.868752
C	-0.003393	-2.011086	0.987497
O	0.889901	-2.670856	0.445604
O	-0.210309	-1.443235	2.063795
C	-0.450877	-3.027034	-2.307872
H	-0.554555	-4.059171	-2.675883
H	-0.327992	-2.352952	-3.168236
H	0.429384	-2.957784	-1.653252
Ru	-0.378411	0.239037	-0.517120
O	0.167822	2.190136	-1.035718
C	1.053783	2.288943	-0.077863
O	1.108996	1.261459	0.697224
C	1.965578	3.446693	0.031645
H	1.566507	4.321695	-0.501628
H	2.171676	3.682792	1.087452
H	2.925657	3.115467	-0.449136
Se	-2.129541	0.765618	1.212799
C	-3.421938	1.992750	0.427692
C	-4.711115	2.029570	0.980138
C	-3.061159	2.841865	-0.626514
C	-5.660068	2.912469	0.447081
H	-4.979459	1.359322	1.803025
C	-4.017240	3.727391	-1.146401
H	-2.049299	2.803376	-1.038834
C	-5.314698	3.760881	-0.615457
H	-6.672171	2.933880	0.863523
H	-3.744046	4.390080	-1.974055
H	-6.058399	4.448965	-1.029444
Br	4.436249	1.442821	-1.505617
Cs	3.561564	-1.007345	0.844761
O	1.216245	-0.481115	-1.774727
C	1.602361	0.021345	-2.863894
O	1.151527	1.168632	-3.346413
C	2.623480	-0.660733	-3.712877
H	3.596442	-0.223583	-3.398057
H	2.622264	-1.744302	-3.526960
H	2.460947	-0.436530	-4.777741
H	0.673128	1.654425	-2.611887

O	2.441610	-0.107789	3.563528
C	1.379535	0.293204	4.037576
O	0.586390	1.194563	3.421748
H	0.935382	1.305154	2.494392
C	0.788780	-0.178543	5.342901
H	1.573453	-0.610159	5.979566
H	0.265490	0.636913	5.865520
H	0.045104	-0.961139	5.108402

Statistical Thermodynamic Analysis
Temperature= 298.150 Kelvin Pressure= 1.00000 Atm

===

SCF Energy= -6558.89631055 Predicted Change= -4.357232D-09
Zero-point correction (ZPE)= -6558.4396 0.45663
Internal Energy (U)= -6558.3975 0.49877
Enthalpy (H)= -6558.3965 0.49972
Gibbs Free Energy (G)= -6558.5178 0.37848
Entropy (S)= 0.00040665

Frequencies -- -130.7953 16.3809 22.4392

Supporting Information: 035-Selenylated-Cluster-RuII-2HOAc-1OAc-CsBr-Decarboxylation-TS-22-PBE.log

----

Using Gaussian 16: ES64L-G16RevA.03 25-Dec-2016

===

# pbepbe/gen/auto pseudo=read gfprint gfinput empiricaldispersion=gd3bj
scf=(direct,vshift=200,tight,maxcycle=300,yqc)
opt=(gdiis,maxcycle=250,calcfc,ts,noeigentest) freq=noraman
#N Geom=AllCheck Guess=TCheck SCRF=Check GenChk RPBEPBE/Gen/Auto Freq

----

Pointgroup= C1 Stoichiometry= C16H28B10BrCsO8RuSe C1[X(C16H28B10BrCsO8RuSe)]
#Atoms= 66
Charge = 0 Multiplicity = 1

SCF Energy= -6558.89069462 Predicted Change= -4.713970D-09

===

Optimization completed on the basis of negligible forces. {Found 2 times}

Item	Max Val.	Criteria	Pass?	RMS Val.	Criteria	Pass?
Force	0.00000 ||	0.00045	[YES]	0.00000 ||	0.00030	[YES]
Displ	0.00302 ||	0.00180	[NO]	0.00302 ||	0.00180	[YES]

Atomic Type	Coordinates (Angstroms) X	Y	Z
B	-1.394963	3.474879	-1.486279
B	-1.785987	4.736276	-0.290372

H	-2.414409	5.719674	-0.559698
H	-1.728447	3.516759	-2.635311
B	-0.469019	4.777098	0.899458
B	-1.984065	3.952877	1.290208
B	0.161303	2.742605	-1.021969
B	-1.369989	1.914073	-0.642166
H	0.958743	2.228951	-1.741681
H	-2.646012	4.301003	2.223735
H	-0.089465	5.689588	1.573048
C	-0.058508	2.033583	0.540237
C	-0.463473	3.228162	1.640697
B	-0.098541	4.492658	-0.814599
B	0.733405	3.562917	0.431249
H	1.863621	3.636329	0.786395
H	0.516270	5.288786	-1.463044
B	-2.563826	3.134365	-0.171517
H	-3.731494	2.962141	-0.364963
B	-1.711710	2.215886	1.091140
H	-2.143887	1.432062	1.870751
C	1.221421	0.895371	1.139848
O	0.899714	0.088368	2.103567
O	2.363993	1.297608	0.841443
C	0.004320	3.038754	3.076461
H	-0.618236	3.657411	3.740536
H	-0.086100	1.982493	3.370143
H	1.055470	3.347387	3.181233
Ru	-0.232606	-0.463727	0.305735
O	-1.461239	-3.158827	-0.919472
C	-0.309891	-3.295122	-0.434464
O	0.246156	-2.466780	0.406931
C	0.560550	-4.472909	-0.829530
H	-0.053545	-5.294013	-1.226281
H	1.251918	-4.125496	-1.618694
H	1.161355	-4.828517	0.023880
Se	-1.722301	0.124998	-1.574282
C	-3.558395	-0.355347	-1.134435
C	-4.089305	-1.367866	-1.953138
C	-4.322524	0.178931	-0.086442
C	-5.382044	-1.851278	-1.713694
H	-3.483979	-1.791084	-2.760885
C	-5.621591	-0.300671	0.135076
H	-3.915005	0.955051	0.560070
C	-6.153036	-1.317083	-0.671548
H	-5.786527	-2.646465	-2.348057
H	-6.220089	0.128687	0.946084
H	-7.165770	-1.690567	-0.490010

O	-1.725125	-0.746639	1.813050
C	-2.638947	-1.604576	1.914442
O	-2.934652	-2.548812	1.057565
C	-3.544212	-1.570866	3.120629
H	-4.552295	-1.262724	2.792488
H	-3.633338	-2.578187	3.558438
H	-3.163607	-0.859645	3.865824
H	-2.314884	-2.587218	0.214507
Cs	3.383755	-1.695041	1.157508
Br	1.455327	-0.594003	-1.663963
O	5.096045	0.168151	-0.555833
C	4.924103	1.120606	-1.323524
O	3.880748	1.954901	-1.283338
H	3.236034	1.656428	-0.566834
C	5.874273	1.467003	-2.451808
H	6.829531	0.941470	-2.314798
H	5.423276	1.153841	-3.410001
H	6.036223	2.555202	-2.507752

Statistical Thermodynamic Analysis
Temperature= 298.150 Kelvin Pressure= 1.00000 Atm

==

SCF Energy= -6558.89069462 Predicted Change= -4.713970D-09
Zero-point correction (ZPE)= -6558.4337 0.45699
Internal Energy (U)= -6558.3915 0.49910
Enthalpy (H)= -6558.3906 0.50005
Gibbs Free Energy (G)= -6558.5109 0.37979
Entropy (S)= 0.00040336

Frequencies -- -194.9731 22.0630 25.5214

Supporting Information: 035-Selenylated-Cluster-RuII-2HOAc-1OAc-CsBr-Decarboxylation-TS-24-PBE.log

----

Using Gaussian 16: ES64L-G16RevA.03 25-Dec-2016

==

# pbepbe/gen/auto pseudo=read gfprint gfinput empiricaldispersion=gd3bj
scf=(direct,vshift=200,tight,maxcycle=300,yqc)
opt=(gdiis,maxcycle=250,calcfc,ts,noeigentest) freq=noraman
#N Geom=AllCheck Guess=TCheck SCRF=Check GenChk RPBEPBE/Gen/Auto Freq

----

Pointgroup= C1 Stoichiometry= C16H28B10BrCsO8RuSe C1[X(C16H28B10BrCsO8RuSe)]
#Atoms= 66
Charge = 0 Multiplicity = 1

SCF Energy= -6558.90032623 Predicted Change= -9.237798D-09

==

Optimization completed on the basis of negligible forces. {Found 2 times}

Item	Max Val.		Criteria	Pass?	RMS Val.		Criteria	Pass?
Force	0.00000	||	0.00045	[YES]	0.00000	||	0.00030	[YES]
Displ	0.00416	||	0.00180	[NO]	0.00416	||	0.00180	[YES]

Atomic Type	Coordinates (Angstroms) X	Y	Z
B	4.175076	1.859726	-0.651308
B	3.977896	3.624811	-0.764551
H	4.793246	4.344994	-1.267080
H	5.124447	1.258271	-1.068305
B	3.043738	4.159261	0.648571
B	2.242626	3.975663	-0.917334
B	3.345803	1.317192	0.840622
B	2.561475	1.154649	-0.716118
H	3.611315	0.357008	1.495049
H	1.712138	4.911712	-1.439571
H	3.056692	5.225942	1.189329
C	1.679251	1.717413	0.639366
C	1.516388	3.378934	0.525092
B	4.232803	2.853429	0.830396
B	2.659640	2.743933	1.640417
H	2.415771	2.815040	2.802033
H	5.224336	2.990560	1.488098
B	2.928034	2.552117	-1.733254
H	2.962678	2.460203	-2.926808
B	1.356010	2.445050	-0.901629
H	0.273748	2.348481	-1.384267
Cs	-3.828426	-1.544050	-0.575691
C	0.259051	4.042421	1.067115
H	-0.620241	3.762464	0.467990
H	0.085165	3.737819	2.107436
H	0.393004	5.133898	1.016238
Ru	0.011204	-0.207969	0.136024
Se	1.950519	-0.658954	-1.338311
C	3.294940	-1.819655	-0.532354
C	4.386400	-2.161741	-1.346134
C	3.153804	-2.373356	0.748825
C	5.353671	-3.056700	-0.866878
H	4.482548	-1.726579	-2.346188
C	4.121752	-3.277272	1.213582
H	2.323936	-2.068232	1.389036
C	5.219369	-3.620361	0.410205
H	6.210988	-3.315069	-1.496973
H	4.019748	-3.702312	2.217810

H	5.973089	-4.322430	0.781061
Br	-1.220885	0.202138	-2.087236
O	-1.777471	-1.063408	2.942949
C	-2.190381	-0.028189	2.353651
O	-1.852933	0.328835	1.147236
C	-3.171546	0.887206	3.056845
H	-2.628689	1.808124	3.332248
H	-3.994523	1.169345	2.379020
H	-3.561050	0.411602	3.967742
O	1.074548	-0.429839	2.023407
C	0.680086	0.780037	2.023444
O	0.152126	1.553544	2.803059
O	-0.540870	-2.220370	-0.071648
C	-0.333314	-3.110699	0.815682
O	-0.392712	-2.927628	2.096392
H	-0.849573	-1.978918	2.375442
C	0.069079	-4.487350	0.355847
H	1.174934	-4.513614	0.327434
H	-0.277215	-5.261389	1.056658
H	-0.295700	-4.677692	-0.664075
O	-4.608878	1.378499	-0.220995
C	-3.958699	2.422872	-0.364048
O	-2.745413	2.621303	0.156957
H	-2.417655	1.746393	0.568299
C	-4.440291	3.610092	-1.166563
H	-3.901427	3.626252	-2.130575
H	-5.519558	3.529053	-1.355959
H	-4.208405	4.553525	-0.647308

Statistical Thermodynamic Analysis
Temperature= 298.150 Kelvin   Pressure= 1.00000 Atm

==

SCF Energy=   -6558.90032623   Predicted Change= -9.237798D-09
Zero-point correction (ZPE)=   -6558.4439   0.45639
Internal Energy (U)=   -6558.4019   0.49840
Enthalpy (H)=   -6558.4009   0.49934
Gibbs Free Energy (G)=   -6558.5214   0.37883
Entropy (S)=   0.00040421

Frequencies --   -231.1627   17.8838   19.7930

Supporting Information: 035-Selenylated-Cluster-RuII-2HOAc-1OAc-CsBr-Decarboxylation-TS-25-PBE.log

----

Using Gaussian 16: ES64L-G16RevA.03 25-Dec-2016

==

pbepbe/gen/auto pseudo=read gfprint gfinput empiricaldispersion=gd3bj

scf=(direct,vshift=200,tight,maxcycle=300,yqc)
opt=(gdiis,maxcycle=250,calcfc,ts,noeigentest) freq=noraman
#N Geom=AllCheck Guess=TCheck SCRF=Check GenChk RPBEPBE/Gen/Auto Freq
----
Pointgroup= C1 Stoichiometry= C16H28B10BrCsO8RuSe C1[X(C16H28B10BrCsO8RuSe)]
#Atoms= 66
Charge = 0 Multiplicity = 1

SCF Energy= -6558.90708000 Predicted Change= -4.492754D-09
==
Optimization completed. {Found 2 times}

Item	Max Val.		Criteria	Pass?	RMS Val.		Criteria	Pass?
Force	0.00001	||	0.00045	[YES]	0.00000	||	0.00030	[YES]
Displ	0.00159	||	0.00180	[YES]	0.00159	||	0.00180	[YES]

Atomic Type	Coordinates (Angstroms) X	Y	Z
B	-4.732807	1.955018	-0.076231
B	-4.397779	2.877776	1.414491
H	-5.264100	3.257551	2.148186
H	-5.834818	1.645026	-0.428185
B	-2.948544	3.873620	1.136665
B	-2.817815	2.360687	2.070922
B	-3.460711	2.363022	-1.264721
B	-3.344903	0.897436	-0.320080
H	-3.528371	2.333683	-2.454132
H	-2.445121	2.377677	3.207234
H	-2.668021	4.917147	1.647453
C	-1.960672	1.885399	-0.530965
C	-1.679116	2.781079	0.870459
B	-4.104559	3.622501	-0.187429
B	-2.355798	3.551905	-0.513807
H	-1.666704	4.308641	-1.122658
H	-4.742854	4.536273	-0.623743
B	-3.919916	1.171040	1.324534
H	-4.408265	0.290752	1.969206
B	-2.177118	1.126646	0.971367
H	-1.541831	0.111945	1.590889
C	-0.705356	1.633947	-1.876212
O	0.299274	2.319206	-1.675817
O	-1.213332	0.935148	-2.748073
C	-0.245671	3.119361	1.245828
H	-0.261824	3.947792	1.970424
H	0.254344	2.248415	1.696891
H	0.307281	3.423902	0.346539

Ru	-0.752015	-0.264000	0.137199
Se	-2.899249	-0.956245	-1.001194
C	-3.909841	-1.979565	0.309388
C	-5.301533	-2.063521	0.153198
C	-3.252428	-2.648048	1.350519
C	-6.049181	-2.809632	1.074070
H	-5.799188	-1.533248	-0.665133
C	-4.012143	-3.399752	2.260138
H	-2.166562	-2.570735	1.457438
C	-5.405795	-3.477840	2.126901
H	-7.137247	-2.865802	0.968013
H	-3.507523	-3.922033	3.079530
H	-5.992823	-4.060239	2.844034
O	0.696993	-1.030141	-1.257309
C	0.720414	-1.846040	-2.215382
O	1.846719	-2.417800	-2.582006
C	-0.468233	-2.234194	-3.042705
H	-1.141936	-2.864508	-2.437184
H	-1.019172	-1.324262	-3.326574
H	-0.149633	-2.798969	-3.929449
H	2.616942	-2.193462	-1.928470
O	1.111768	0.069673	1.264588
C	1.074492	-1.166351	1.615450
C	2.196206	-1.838334	2.334630
H	2.939508	-2.151041	1.572438
H	2.689695	-1.136520	3.023989
H	1.837666	-2.728467	2.872564
O	0.024371	-1.835886	1.253602
Cs	3.108815	1.421687	-0.836333
Br	4.364093	-1.914118	-0.782301
O	5.050844	1.227005	1.523823
C	5.544149	0.395739	2.289188
O	5.602550	-0.921892	2.046236
H	5.208056	-1.120664	1.135356
C	6.170437	0.744262	3.623641
H	7.257065	0.555459	3.582902
H	5.991137	1.802819	3.856017
H	5.761248	0.102690	4.421277

Statistical Thermodynamic Analysis
Temperature= 298.150 Kelvin Pressure= 1.00000 Atm

===

SCF Energy= -6558.90708000 Predicted Change= -4.492754D-09
Zero-point correction (ZPE)= -6558.4513 0.45568
Internal Energy (U)= -6558.4089 0.49817
Enthalpy (H)= -6558.4079 0.49912

Gibbs Free Energy (G)= -6558.5310 0.37602
Entropy (S)= 0.00041286

Frequencies -- -143.3367 18.7626 19.7782
Supporting Information: 035-Selenylated-Cluster-RuII-2HOAc-1OAc-CsBr-Decarboxylation-TS-26-PBE.log
----
Using Gaussian 16: ES64L-G16RevA.03 25-Dec-2016
==
# pbepbe/gen/auto pseudo=read gfprint gfinput empiricaldispersion=gd3bj
scf=(direct,vshift=200,tight,maxcycle=300,yqc)
opt=(gdiis,maxcycle=250,calcfc,ts,noeigentest) freq=noraman
#N Geom=AllCheck Guess=TCheck SCRF=Check GenChk RPBEPBE/Gen/Auto Freq
----
Pointgroup= C1 Stoichiometry= C16H28B10BrCsO8RuSe C1[X(C16H28B10BrCsO8RuSe)]
#Atoms= 66
Charge = 0 Multiplicity = 1

SCF Energy= -6558.90523262 Predicted Change= -5.532735D-09
==
Optimization completed. {Found 1 times}

Item	Max Val.	Criteria	Pass?	RMS Val.	Criteria	Pass?
Force	0.00001 ||	0.00045	[YES]	0.00000 ||	0.00030	[YES]
Displ	0.00500 ||	0.00180	[NO]	0.00500 ||	0.00180	[YES]

Atomic Type	Coordinates (Angstroms) X	Y	Z
B	3.743560	2.812098	0.369447
B	3.455863	3.507429	-1.247541
H	4.293983	4.117203	-1.846495
H	4.787916	2.894643	0.949664
B	1.745091	3.992260	-1.349518
B	2.286946	2.477814	-2.123608
B	2.187966	2.835337	1.253419
B	2.733296	1.374292	0.471084
H	2.015512	2.885063	2.431477
H	2.172558	2.324357	-3.303990
H	1.270508	4.867495	-2.010753
C	1.098594	1.859805	0.309927
C	0.848255	2.565389	-1.204649
B	2.619047	4.186315	0.183464
B	0.949654	3.562683	0.190636
H	-0.044166	4.096292	0.573550
H	2.836969	5.275843	0.628365
B	3.522602	1.741410	-1.062216

H	4.381588	1.038571	-1.506789
B	1.842484	1.160365	-1.028564
H	1.643544	-0.033911	-1.591053
C	-0.304735	1.306972	1.542823
O	-1.400646	1.648822	1.084611
O	0.225976	0.915625	2.562335
C	-0.506001	2.403247	-1.875652
H	-0.549008	3.092566	-2.733022
H	-0.639533	1.371492	-2.234671
H	-1.321097	2.649028	-1.180715
Ru	0.701967	-0.510343	-0.241356
Se	2.708507	-0.470568	1.295349
C	4.179779	-1.246279	0.285692
C	5.488937	-0.907058	0.659016
C	3.929796	-2.153702	-0.752050
C	6.566013	-1.468630	-0.039737
H	5.665642	-0.195755	1.472267
C	5.018122	-2.716032	-1.437088
H	2.902303	-2.407894	-1.028031
C	6.331706	-2.372839	-1.086871
H	7.589915	-1.196252	0.235426
H	4.832690	-3.423860	-2.251581
H	7.175453	-2.810681	-1.629493
O	-0.756438	-1.514872	0.966054
C	-0.708049	-2.339736	1.923030
O	-1.694002	-3.163271	2.156682
C	0.441217	-2.448560	2.884321
H	1.312778	-2.874567	2.357642
H	0.717556	-1.444830	3.241027
H	0.174325	-3.103442	3.724929
H	-2.412566	-3.179824	1.372144
O	-0.898357	-0.851264	-1.730540
C	-0.458855	-2.053209	-1.868508
C	-1.176437	-3.083244	-2.673877
H	-2.018547	-3.459040	-2.050340
H	-1.594580	-2.633704	-3.588496
H	-0.507942	-3.920330	-2.923472
O	0.619555	-2.351326	-1.215455
Cs	-3.613106	0.014531	-0.377916
Br	-3.789383	-3.472304	-0.051778
O	-3.844733	3.136942	-0.382469
C	-3.598023	4.099241	0.348782
O	-2.647124	4.086499	1.300223
H	-2.188728	3.194528	1.269595
C	-4.310963	5.429894	0.280988
H	-5.144593	5.372689	-0.431615

H -4.679462 5.719063 1.278929
H -3.602075 6.212326 -0.040491

Statistical Thermodynamic Analysis
Temperature= 298.150 Kelvin Pressure= 1.00000 Atm

===

SCF Energy= -6558.90523262 Predicted Change= -5.532735D-09
Zero-point correction (ZPE)= -6558.4495 0.45571
Internal Energy (U)= -6558.4073 0.49791
Enthalpy (H)= -6558.4063 0.49886
Gibbs Free Energy (G)= -6558.5281 0.37708
Entropy (S)= 0.00040846

Frequencies -- -142.5516 18.9481 25.2130
Supporting Information: 035-Selenylated-Cluster-RuII-2HOAc-1OAc-CsBr-Decarboxylation-TS-27-PBE.log

---

Using Gaussian 16: ES64L-G16RevA.03 25-Dec-2016

===

# pbepbe/gen/auto pseudo=read gfprint gfinput empiricaldispersion=gd3bj
scf=(direct,vshift=200,tight,maxcycle=300,yqc)
opt=(gdiis,maxcycle=250,calcfc,ts,noeigentest) freq=noraman
#N Geom=AllCheck Guess=TCheck SCRF=Check GenChk RPBEPBE/Gen/Auto Freq

---

Pointgroup= C1 Stoichiometry= C16H28B10BrCsO8RuSe C1[X(C16H28B10BrCsO8RuSe)]
#Atoms= 66
Charge = 0 Multiplicity = 1

SCF Energy= -6558.90224656 Predicted Change= -5.496432D-09

===

Optimization completed. {Found 1 times}

Item	Max Val.	Criteria	Pass?	RMS Val.	Criteria	Pass?
Force	0.00001 ||	0.00045	[YES]	0.00000 ||	0.00030	[YES]
Displ	0.00326 ||	0.00180	[NO]	0.00326 ||	0.00180	[YES]

Atomic Type	Coordinates (Angstroms) X	Y	Z
B	-0.251333	3.417235	1.668285
B	0.768403	4.586924	0.797991
H	1.189708	5.582799	1.313400
H	-0.569305	3.535538	2.819353
B	0.265914	4.594524	-0.906520
B	1.738959	3.712116	-0.420738
B	-1.360108	2.673365	0.479709
B	0.095455	1.846079	0.964396

H	-2.441748	2.221208	0.699549
H	2.803552	4.013952	-0.871715
H	0.336062	5.495795	-1.688966
C	-0.344218	1.883605	-0.693665
C	0.595392	3.040993	-1.495067
B	-0.959358	4.398303	0.357183
B	-1.041168	3.390701	-1.117578
H	-1.831744	3.442349	-2.008941
H	-1.789906	5.240803	0.544160
B	1.426631	2.976166	1.169932
H	2.316339	2.766102	1.939826
B	1.290632	1.994333	-0.318580
H	2.257405	1.144924	-0.591386
C	-1.517114	0.573697	-1.721055
O	-1.321533	0.664975	-2.907026
O	-2.314210	0.162329	-0.871460
C	0.951300	2.817249	-2.955410
H	0.796694	3.759705	-3.503082
H	2.004292	2.513497	-3.045496
H	0.319368	2.030213	-3.386655
Ru	1.085809	-0.148634	-0.790829
Se	0.148150	-0.091991	1.518540
C	1.558839	-0.145056	2.849788
C	2.455247	-1.223638	2.800049
C	1.623697	0.804696	3.879289
C	3.441671	-1.337104	3.790307
H	2.395946	-1.942852	1.977972
C	2.619960	0.683385	4.858541
H	0.916687	1.638895	3.909602
C	3.528667	-0.384165	4.816046
H	4.148324	-2.173333	3.754422
H	2.685337	1.431409	5.655254
H	4.305152	-0.472767	5.582431
O	1.580100	-0.132164	-2.874503
C	2.214197	-1.023313	-3.488126
C	2.229475	-1.037882	-4.991868
H	1.938423	-0.052671	-5.381001
H	3.220920	-1.332010	-5.369574
H	1.496414	-1.786862	-5.340530
O	2.882425	-2.008060	-2.919697
Cs	-2.908189	-2.291605	0.847165
Br	-0.019111	-2.462996	-1.205996
H	2.786373	-1.921380	-1.903955
O	2.759757	-1.315311	-0.396976
C	3.931487	-0.713860	-0.144630
O	4.133242	0.502111	-0.129074

C	5.032076	-1.718667	0.173975
H	5.113615	-1.801900	1.272485
H	4.817346	-2.717546	-0.235344
H	5.994478	-1.345185	-0.207372
O	-5.207213	-0.300219	0.715116
C	-5.520986	0.761435	0.168755
O	-4.738255	1.418722	-0.698955
H	-3.864149	0.927364	-0.785325
C	-6.827839	1.483918	0.407353
H	-7.302809	1.747132	-0.551746
H	-7.500172	0.856211	1.007618
H	-6.631764	2.429748	0.941458

Statistical Thermodynamic Analysis
Temperature= 298.150 Kelvin   Pressure= 1.00000 Atm

==

SCF Energy= -6558.90224656 Predicted Change= -5.496432D-09
Zero-point correction (ZPE)= -6558.4460 0.45623
Internal Energy (U)= -6558.4030 0.49919
Enthalpy (H)= -6558.4021 0.50013
Gibbs Free Energy (G)= -6558.5268 0.37536
Entropy (S)= 0.00041848

Frequencies -- -154.1250 15.6624 22.9805

Supporting Information: 035-Selenylated-Cluster-RuII-2HOAc-1OAc-CsBr-Decarboxylation-TS-28-PBE.log

----

Using Gaussian 16: ES64L-G16RevA.03 25-Dec-2016

==

# pbepbe/gen/auto pseudo=read gfprint gfinput empiricaldispersion=gd3bj
scf=(direct,vshift=200,tight,maxcycle=300,yqc)
opt=(gdiis,maxcycle=250,calcfc,ts,noeigentest) freq=noraman
#N Geom=AllCheck Guess=TCheck SCRF=Check GenChk RPBEPBE/Gen/Auto Freq

----

Pointgroup= C1 Stoichiometry= C16H28B10BrCsO8RuSe C1[X(C16H28B10BrCsO8RuSe)]
#Atoms= 66
Charge = 0 Multiplicity = 1

SCF Energy= -6558.89932882 Predicted Change= -5.114810D-09

==

Optimization completed. {Found 1 times}

Item	Max Val.	Criteria	Pass?	RMS Val.	Criteria	Pass?
Force	0.00001 ||	0.00045	[YES]	0.00000 ||	0.00030	[YES]
Displ	0.00281 ||	0.00180	[NO]	0.00281 ||	0.00180	[YES]

Atomic Coordinates (Angstroms)

Type	X	Y	Z
B	-2.441685	3.668452	-0.693648
B	-2.450563	4.307037	0.963735
H	-3.231945	5.139070	1.327813
H	-3.216245	4.005477	-1.545026
B	-0.779087	4.327404	1.559264
B	-1.894326	3.028171	2.091704
B	-0.743745	3.236229	-1.095341
B	-1.870425	2.017679	-0.575569
H	-0.257797	3.200324	-2.184812
H	-2.167192	2.903272	3.248133
H	-0.298814	5.067728	2.366077
C	-0.283427	2.022273	0.069012
C	-0.286203	2.718294	1.612100
B	-1.090143	4.692413	-0.142857
B	0.285316	3.628566	0.291498
H	1.452656	3.871147	0.231135
H	-0.882446	5.788220	-0.579691
B	-2.929917	2.611232	0.695070
H	-4.036635	2.195162	0.868938
B	-1.503910	1.615962	1.096313
H	-1.583735	0.409092	1.600415
C	1.604646	1.078334	-1.017039
O	2.495287	1.194803	-0.192947
O	1.222944	0.994207	-2.146171
C	0.760084	2.271259	2.615646
H	1.138846	3.162874	3.140077
H	0.336094	1.566389	3.343240
H	1.588453	1.766545	2.101091
Ru	-0.536053	-0.245602	0.338169
Se	-1.973058	0.335656	-1.679523
C	-3.774751	-0.316935	-1.384055
C	-3.970278	-1.707927	-1.396851
C	-4.869274	0.554734	-1.281525
C	-5.266355	-2.225551	-1.266369
H	-3.115622	-2.381109	-1.505754
C	-6.160548	0.026373	-1.142575
H	-4.719066	1.636581	-1.307179
C	-6.362096	-1.360966	-1.127323
H	-5.417592	-3.310490	-1.275397
H	-7.012062	0.707936	-1.048170
H	-7.372302	-1.767901	-1.018005
O	0.634990	-1.540639	-0.987033
C	0.261572	-2.640470	-1.481050
O	-0.766926	-3.342558	-1.088052

C	1.040663	-3.237671	-2.631444
H	0.373181	-3.335736	-3.504490
H	1.887944	-2.592323	-2.905043
H	1.387218	-4.253991	-2.377165
H	-1.222376	-2.851045	-0.214565
Cs	3.695004	-1.686453	0.371015
Br	0.907763	-1.055885	2.356454
O	-1.609251	-1.995471	0.751457
C	-2.551325	-2.051575	1.701874
O	-2.879674	-1.093313	2.396552
C	-3.212481	-3.418006	1.806653
H	-3.642596	-3.538403	2.811245
H	-4.032221	-3.471197	1.068321
H	-2.503661	-4.235566	1.598580
O	5.563415	0.246810	-1.068256
C	5.724530	1.427348	-1.389982
O	4.794636	2.380543	-1.221527
H	3.976938	1.956671	-0.826220
C	6.983154	1.973888	-2.023213
H	7.745961	1.186111	-2.082750
H	6.756812	2.350758	-3.035222
H	7.363167	2.828317	-1.439109

Statistical Thermodynamic Analysis
Temperature= 298.150 Kelvin Pressure= 1.00000 Atm
===
SCF Energy= -6558.89932882 Predicted Change= -5.114810D-09
Zero-point correction (ZPE)= -6558.4449 0.45438
Internal Energy (U)= -6558.4016 0.49764
Enthalpy (H)= -6558.4007 0.49858
Gibbs Free Energy (G)= -6558.5273 0.37194
Entropy (S)= 0.00042477

Frequencies -- -39.6041 9.2968 20.9893
Supporting Information: 035-Selenylated-Cluster-RuII-2HOAc-1OAc-CsBr-Decarboxylation-TS-29-PBE.log
----
Using Gaussian 16: ES64L-G16RevA.03 25-Dec-2016
===
pbepbe/gen/auto pseudo=read gfprint gfinput empiricaldispersion=gd3bj scf=(direct,vshift=200,tight,maxcycle=300,yqc) opt=(gdiis,maxcycle=250,calcfc,ts,noeigentest) freq=noraman
#N Geom=AllCheck Guess=TCheck SCRF=Check GenChk RPBEPBE/Gen/Auto Freq
----
Pointgroup= C1 Stoichiometry= C16H28B10BrCsO8RuSe C1[X(C16H28B10BrCsO8RuSe)] #Atoms= 66

Charge = 0 Multiplicity = 1

SCF Energy= -6558.91187297 Predicted Change= -4.743758D-09

==

Optimization completed. {Found 2 times}

Item	Max Val.	Criteria	Pass?	RMS Val.	Criteria	Pass?
Force	0.00001 ||	0.00045	[YES]	0.00000 ||	0.00030	[YES]
Displ	0.00129 ||	0.00180	[YES]	0.00129 ||	0.00180	[YES]

Atomic Type	Coordinates (Angstroms) X	Y	Z
B	3.676846	-2.903805	-0.285447
B	3.424229	-3.557371	1.353181
H	4.285661	-4.120876	1.965255
H	4.719465	-2.972381	-0.871649
B	1.726904	-4.088538	1.484293
B	2.235442	-2.537365	2.202850
B	2.120092	-3.004171	-1.155534
B	2.623002	-1.497294	-0.430774
H	1.944532	-3.097814	-2.329749
H	2.123841	-2.351293	3.379302
H	1.281154	-4.955722	2.176172
C	1.001082	-2.033011	-0.241284
C	0.790872	-2.692841	1.299957
B	2.599504	-4.307362	-0.047379
B	0.913293	-3.734108	-0.066343
H	-0.069071	-4.310072	-0.419062
H	2.850167	-5.403997	-0.457266
B	3.433230	-1.794849	1.110305
H	4.273674	-1.052888	1.526354
B	1.733095	-1.268209	1.074230
H	1.419628	-0.128547	1.627110
C	-0.494108	-1.642661	-1.412422
O	-1.551625	-1.822680	-0.789370
O	-0.053756	-1.478923	-2.525989
C	-0.557042	-2.544506	1.987251
H	-0.438898	-2.854061	3.037544
H	-0.893249	-1.496938	1.958709
H	-1.313397	-3.181838	1.509270
Ru	0.598138	0.395093	0.175430
Se	2.571501	0.303666	-1.346775
C	4.071196	1.113046	-0.406102
C	5.371574	0.741362	-0.778465
C	3.846217	2.073950	0.587810
C	6.464444	1.325203	-0.123566

H	5.529680	-0.013294	-1.555651
C	4.949289	2.657341	1.229884
H	2.824494	2.351100	0.862296
C	6.254552	2.282932	0.880101
H	7.481521	1.027354	-0.398111
H	4.782530	3.406767	2.010713
H	7.110459	2.737258	1.389444
O	-1.082528	0.813461	1.611498
C	-0.530681	1.948814	1.840077
C	-1.084806	2.918325	2.846978
H	-0.917590	2.528091	3.866175
H	-0.590765	3.895541	2.746538
H	-2.170901	3.027314	2.696977
O	0.547698	2.232922	1.192374
Cs	-3.674230	0.338193	-0.145089
Br	-0.643297	1.676372	-1.676782
O	-1.505935	4.466470	-0.276393
C	-2.734865	4.418857	0.252893
C	-3.172240	5.772447	0.772764
H	-2.551664	6.051073	1.642468
H	-3.019711	6.548626	0.005508
H	-4.227786	5.735465	1.074611
O	-3.422749	3.396860	0.336445
H	-1.248937	3.552670	-0.630349
O	-4.593595	-2.610025	0.176743
C	-4.177696	-3.766668	0.087731
O	-2.951499	-4.092430	-0.359302
H	-2.455541	-3.247191	-0.594186
C	-4.979657	-4.992459	0.465198
H	-5.967371	-4.693464	0.840885
H	-5.095184	-5.650621	-0.412504
H	-4.442898	-5.573265	1.234073

Statistical Thermodynamic Analysis
Temperature= 298.150 Kelvin Pressure= 1.00000 Atm

==

SCF Energy= -6558.91187297 Predicted Change= -4.743758D-09
Zero-point correction (ZPE)= -6558.4561 0.45577
Internal Energy (U)= -6558.4127 0.49917
Enthalpy (H)= -6558.4117 0.50011
Gibbs Free Energy (G)= -6558.5383 0.37355
Entropy (S)= 0.00042448

Frequencies -- -139.7202 16.6235 23.8276

Supporting Information: 035-Selenylated-Cluster-RuII-2HOAc-1OAc-CsBr-Decarboxylation-TS-30-PBE.log

----
Using Gaussian 16: ES64L-G16RevA.03 25-Dec-2016
==
# pbepbe/gen/auto pseudo=read gfprint gfinput empiricaldispersion=gd3bj
scf=(direct,vshift=200,tight,maxcycle=300,yqc)
opt=(gdiis,maxcycle=250,calcfc,ts,noeigentest) freq=noraman
#N Geom=AllCheck Guess=TCheck SCRF=Check GenChk RPBEPBE/Gen/Auto Freq
----
Pointgroup= C1 Stoichiometry= C16H28B10BrCsO8RuSe C1[X(C16H28B10BrCsO8RuSe)]
#Atoms= 66
Charge = 0 Multiplicity = 1

SCF Energy= -6558.90854865 Predicted Change= -5.491708D-09
==
Optimization completed. {Found 2 times}

Item	Max Val.	Criteria	Pass?	RMS Val.	Criteria	Pass?
Force	0.00003 ||	0.00045	[YES]	0.00000 ||	0.00030	[YES]
Displ	0.00137 ||	0.00180	[YES]	0.00137 ||	0.00180	[YES]

Atomic Type	Coordinates (Angstroms) X	Y	Z
B	3.104655	2.882483	1.455357
B	3.006082	4.002038	0.073174
H	3.831350	4.843981	-0.137502
H	4.001884	2.888702	2.249348
B	1.283945	4.321358	-0.262886
B	2.196305	3.175554	-1.281888
B	1.433983	2.483585	1.950772
B	2.336213	1.383550	0.939442
H	1.050466	2.157389	3.030495
H	2.320322	3.377237	-2.454408
H	0.799875	5.303320	-0.743335
C	0.696243	1.732862	0.566475
C	0.618724	2.835288	-0.714204
B	1.829768	4.131948	1.416910
B	0.307714	3.369127	0.892046
H	-0.811826	3.663700	1.176433
H	1.792079	5.054083	2.179618
B	3.316488	2.275703	-0.226433
H	4.342636	1.830838	-0.649571
B	1.765047	1.552058	-0.720126
H	1.739086	0.631033	-1.638858
C	-0.900180	0.793164	1.204644
O	-1.837788	1.066845	0.445709
O	-0.639305	0.319111	2.286078

C	-0.539424	2.744494	-1.694688
H	-0.280352	3.340388	-2.583935
H	-0.708415	1.700684	-2.000246
H	-1.460777	3.145339	-1.250723
Ru	0.823622	-0.450132	-0.606269
Se	2.480491	-0.607910	1.246288
C	4.237753	-0.861370	0.447633
C	5.367117	-0.482840	1.188478
C	4.360911	-1.442378	-0.820717
C	6.641597	-0.666943	0.635335
H	5.252436	-0.027213	2.177322
C	5.643108	-1.628779	-1.359762
H	3.467299	-1.732563	-1.380363
C	6.780591	-1.239440	-0.638082
H	7.526541	-0.358686	1.201375
H	5.748983	-2.078635	-2.352522
H	7.777571	-1.381749	-1.067272
O	-0.537648	-0.651566	-2.394726
C	0.257454	-1.564852	-2.819146
C	-0.011211	-2.336623	-4.083044
H	-0.561256	-3.262065	-3.834025
H	-0.618096	-1.737300	-4.778828
H	0.936406	-2.629845	-4.560388
O	1.299192	-1.838891	-2.112949
Cs	-3.445930	-1.072958	-1.039614
Br	-0.389227	-2.476175	0.407398
H	-1.534883	-2.118956	2.378549
O	-2.029364	-2.159925	3.251350
C	-3.326733	-1.911985	3.029951
C	-4.109132	-1.842244	4.324311
H	-3.805681	-2.648602	5.010639
H	-3.887673	-0.883962	4.826257
H	-5.186331	-1.898036	4.115059
O	-3.826704	-1.742652	1.915365
O	-4.670349	1.771987	-1.078904
C	-4.539104	2.867829	-0.531937
O	-3.553542	3.162835	0.336451
H	-2.960938	2.356637	0.440671
C	-5.459797	4.047558	-0.752627
H	-6.257329	3.773894	-1.456276
H	-5.897817	4.368882	0.207277
H	-4.886962	4.903643	-1.147465

Statistical Thermodynamic Analysis
Temperature= 298.150 Kelvin   Pressure= 1.00000 Atm

==

SCF Energy= -6558.90854865 Predicted Change= -5.491708D-09
Zero-point correction (ZPE)= -6558.4528 0.45572
Internal Energy (U)= -6558.4091 0.49936
Enthalpy (H)= -6558.4082 0.50030
Gibbs Free Energy (G)= -6558.5360 0.37251
Entropy (S)= 0.00042864

Frequencies -- -138.5555 15.8910 18.9911

Supporting Information: 035-Selenylated-Cluster-RuII-2HOAc-1OAc-CsBr-Decarboxylation-TS-31-PBE.log

---

Using Gaussian 16: ES64L-G16RevA.03 25-Dec-2016

===

# pbepbe/gen/auto pseudo=read gfprint gfinput empiricaldispersion=gd3bj
scf=(direct,vshift=200,tight,maxcycle=300,yqc)
opt=(gdiis,maxcycle=250,calcfc,ts,noeigentest) freq=noraman
#N Geom=AllCheck Guess=TCheck SCRF=Check GenChk RPBEPBE/Gen/Auto Freq

---

Pointgroup= C1 Stoichiometry= C16H28B10BrCsO8RuSe C1[X(C16H28B10BrCsO8RuSe)]
#Atoms= 66
Charge = 0 Multiplicity = 1

SCF Energy= -6558.91105389 Predicted Change= -7.692973D-09

===

Optimization completed. {Found 1 times}

Item	Max Val.	Criteria	Pass?	RMS Val.	Criteria	Pass?
Force	0.00000 ||	0.00045	[YES]	0.00000 ||	0.00030	[YES]
Displ	0.00635 ||	0.00180	[NO]	0.00635 ||	0.00180	[YES]

Atomic Type	Coordinates (Angstroms) X	Y	Z
B	3.468097	3.117891	0.219777
B	3.675630	3.451171	-1.520202
H	4.642231	4.002768	-1.962875
H	4.281414	3.409952	1.049896
B	2.065318	3.743277	-2.224692
B	2.863917	2.166121	-2.451787
B	1.717852	3.174400	0.576689
B	2.515005	1.638931	0.332116
H	1.191538	3.439092	1.612513
H	3.122562	1.771058	-3.551111
H	1.791246	4.417617	-3.173339
C	0.994437	1.936428	-0.405535
C	1.210890	2.290516	-2.035933
B	2.422028	4.322193	-0.584195

B	0.850618	3.560928	-0.931766
H	-0.231676	4.056740	-0.984447
H	2.466775	5.494548	-0.344148
B	3.729064	1.770862	-0.943607
H	4.704162	1.078846	-0.952592
B	2.136144	1.050539	-1.279583
H	2.094564	-0.180908	-1.716058
C	-0.725882	1.519081	0.325600
O	-1.561981	1.525096	-0.574570
O	-0.602243	1.482856	1.538089
C	0.141748	1.857729	-3.026760
H	0.415750	2.243932	-4.020790
H	0.080152	0.758277	-3.068211
H	-0.838484	2.253207	-2.730040
Ru	0.975040	-0.651825	-0.478774
Se	2.336142	0.000638	1.507290
C	4.136002	-0.692596	1.248857
C	5.196153	-0.064261	1.919064
C	4.350279	-1.819427	0.445306
C	6.497058	-0.560346	1.759305
H	5.011856	0.817865	2.540670
C	5.656450	-2.311461	0.300247
H	3.510253	-2.293645	-0.069501
C	6.728237	-1.683579	0.950854
H	7.330990	-0.064535	2.266554
H	5.833656	-3.189227	-0.330059
H	7.745680	-2.068561	0.828267
O	-0.161074	-1.567998	-2.198071
C	0.590026	-2.599314	-2.061977
C	0.413852	-3.839213	-2.895017
H	-0.280430	-4.527317	-2.379995
H	-0.006898	-3.583366	-3.879503
H	1.376229	-4.359566	-3.015396
O	1.504878	-2.563509	-1.154253
Cs	-3.182986	-0.962788	-1.187836
Br	-0.554505	-1.994695	1.104330
H	-1.614014	-0.765689	2.612024
O	-2.055302	-0.245468	3.359646
C	-3.173170	0.306299	2.917192
C	-3.743975	1.323483	3.873132
H	-3.532568	1.044558	4.916096
H	-3.239386	2.286148	3.674464
H	-4.824162	1.452740	3.714448
O	-3.685297	0.032178	1.813241
O	-6.003571	0.136652	-0.656095
C	-6.565422	0.869243	0.163127

O	-6.041494	1.210672	1.347584
H	-5.132243	0.762073	1.459046
C	-7.928315	1.491414	-0.055881
H	-7.848834	2.591265	-0.022733
H	-8.333375	1.176072	-1.026905
H	-8.615598	1.192977	0.753538

Statistical Thermodynamic Analysis
Temperature= 298.150 Kelvin Pressure= 1.00000 Atm
==
SCF Energy= -6558.91105389 Predicted Change= -7.692973D-09
Zero-point correction (ZPE)= -6558.4552 0.45577
Internal Energy (U)= -6558.4118 0.49919
Enthalpy (H)= -6558.4109 0.50014
Gibbs Free Energy (G)= -6558.5390 0.37199
Entropy (S)= 0.00042982

Frequencies -- -150.1439 5.5017 16.2118
Supporting Information: 035-Selenylated-Cluster-RuII-2HOAc-1OAc-CsBr-Decarboxylation-TS-32-PBE.log
----
Using Gaussian 16: ES64L-G16RevA.03 25-Dec-2016
==
pbepbe/gen/auto pseudo=read gfprint gfinput empiricaldispersion=gd3bj scf=(direct,vshift=200,tight,maxcycle=300,yqc) opt=(gdiis,maxcycle=250,calcfc,ts,noeigentest) freq=noraman
#N Geom=AllCheck Guess=TCheck SCRF=Check GenChk RPBEPBE/Gen/Auto Freq
----
Pointgroup= C1 Stoichiometry= C16H28B10BrCsO8RuSe C1[X(C16H28B10BrCsO8RuSe)] #Atoms= 66
Charge = 0 Multiplicity = 1

SCF Energy= -6558.91002810 Predicted Change= -8.183686D-09
==
Optimization completed on the basis of negligible forces. {Found 2 times}

Item	Max Val.	Criteria	Pass?	RMS Val.	Criteria	Pass?
Force	0.00000 ||	0.00045	[YES]	0.00000 ||	0.00030	[YES]
Displ	0.00519 ||	0.00180	[NO]	0.00519 ||	0.00180	[YES]

Atomic Type	Coordinates (Angstroms) X	Y	Z
B	4.088346	2.305150	-0.273013
B	3.714051	2.921429	-1.904359
H	4.563270	3.231611	-2.689759
H	5.204250	2.139674	0.130226

B	2.194588	3.838551	-1.810945
B	2.185919	2.180662	-2.463547
B	2.771096	2.820909	0.819488
B	2.776279	1.210415	0.150746
H	2.831700	3.009856	1.994651
H	1.830621	1.973193	-3.587577
H	1.843439	4.753721	-2.495565
C	1.322747	2.096640	0.180363
C	1.002426	2.721796	-1.358497
B	3.338945	3.913119	-0.460537
B	1.591783	3.760727	-0.129089
H	0.839899	4.565986	0.326037
H	3.898066	4.937264	-0.190830
B	3.357536	1.226886	-1.516388
H	3.923760	0.286147	-1.989771
B	1.614111	1.105935	-1.168231
H	1.033959	-0.053040	-1.646245
C	0.055023	2.039828	1.674507
O	-1.000213	2.561516	1.339218
O	0.669780	1.589698	2.624544
C	-0.441764	2.943086	-1.776202
H	-0.583596	4.006446	-2.022243
H	-0.680998	2.342445	-2.665424
H	-1.116321	2.667367	-0.956081
Ru	0.342583	-0.262622	-0.123734
Se	2.540196	-0.511426	1.174465
C	3.937727	-1.515956	0.260063
C	5.258218	-1.263100	0.664112
C	3.654148	-2.460840	-0.733101
C	6.309844	-1.953388	0.046406
H	5.464323	-0.518443	1.439580
C	4.715185	-3.151346	-1.338257
H	2.619359	-2.648438	-1.031767
C	6.039758	-2.898329	-0.954647
H	7.341971	-1.748099	0.348365
H	4.499055	-3.887839	-2.119289
H	6.862987	-3.436252	-1.435670
Br	0.020730	-2.669586	-0.829287
O	-0.764697	-0.730074	1.710558
C	-0.440319	-1.576392	2.576380
O	0.199897	-2.701191	2.316542
H	0.286572	-2.788020	1.302921
C	-0.753285	-1.334167	4.026682
H	-0.334643	-0.349961	4.297462
H	-1.846587	-1.288580	4.170185
H	-0.336606	-2.124037	4.666260

O	-1.557341	0.247171	-0.814112
C	-2.221381	0.126369	-1.921960
C	-1.560045	-0.468574	-3.149017
H	-2.243327	-0.426123	-4.009042
H	-0.630746	0.078158	-3.386855
H	-1.265894	-1.511329	-2.945499
O	-3.419243	0.536038	-1.961364
Cs	-3.695460	1.032271	1.180531
O	-5.725365	-0.828634	-0.164971
C	-6.034663	-1.346844	-1.243533
O	-5.417477	-1.116080	-2.405795
H	-4.643214	-0.463100	-2.263673
C	-7.172577	-2.336688	-1.397464
H	-6.775022	-3.313964	-1.720829
H	-7.703764	-2.451594	-0.442757
H	-7.870326	-1.997618	-2.181106

Statistical Thermodynamic Analysis
Temperature= 298.150 Kelvin Pressure= 1.00000 Atm

==

SCF Energy= -6558.91002810 Predicted Change= -8.183686D-09
Zero-point correction (ZPE)= -6558.4545 0.45545
Internal Energy (U)= -6558.4117 0.49831
Enthalpy (H)= -6558.4107 0.49925
Gibbs Free Energy (G)= -6558.5356 0.37441
Entropy (S)= 0.00041873

Frequencies -- -151.8210 8.3011 20.9316

Supporting Information: 035-Selenylated-Cluster-RuII-2HOAc-1OAc-CsBr-Decarboxylation-TS-33-PBE.log

----

Using Gaussian 16: ES64L-G16RevA.03 25-Dec-2016

==

# pbepbe/gen/auto pseudo=read gfprint gfinput empiricaldispersion=gd3bj
scf=(direct,vshift=200,tight,maxcycle=300,yqc)
opt=(gdiis,maxcycle=250,calcfc,ts,noeigentest) freq=noraman
#N Geom=AllCheck Guess=TCheck SCRF=Check GenChk RPBEPBE/Gen/Auto Freq

----

Pointgroup= C1 Stoichiometry= C16H28B10BrCsO8RuSe C1[X(C16H28B10BrCsO8RuSe)]
#Atoms= 66
Charge = 0 Multiplicity = 1

SCF Energy= -6558.90449288 Predicted Change= -5.548417D-09

==

Optimization completed on the basis of negligible forces. {Found 2 times}
Item Max Val. Criteria Pass? RMS Val. Criteria Pass?

Force 0.00000 || 0.00045 [YES] 0.00000 || 0.00030 [YES]
Displ 0.00513 || 0.00180 [NO] 0.00513 || 0.00180 [YES]

Atomic Type	Coordinates (Angstroms) X	Y	Z
B	-4.844295	0.390441	0.294105
B	-5.316982	-0.599206	-1.113799
H	-6.243465	-0.319993	-1.819626
H	-5.404948	1.401310	0.608842
B	-4.939881	-2.296013	-0.746315
B	-3.852184	-1.360260	-1.799826
B	-4.148355	-0.706628	1.521746
B	-3.100827	0.208643	0.460578
H	-4.119285	-0.561571	2.703973
H	-3.654638	-1.700392	-2.929630
H	-5.481114	-3.276318	-1.165140
C	-2.769990	-1.437806	0.764539
C	-3.253742	-2.374503	-0.565254
B	-5.533907	-1.236575	0.544980
B	-4.196597	-2.370767	0.875363
H	-4.185702	-3.355034	1.546686
H	-6.603313	-1.428740	1.048804
B	-3.790035	0.300882	-1.160793
H	-3.573141	1.232360	-1.877799
B	-2.473705	-0.845577	-0.800457
H	-1.357335	-0.627659	-1.553283
C	-1.573337	-1.974802	2.011062
O	-1.210685	-3.137947	1.853526
O	-1.461707	-1.049264	2.822199
C	-2.504684	-3.658065	-0.880801
H	-1.848703	-3.915004	-0.036374
H	-3.240324	-4.461362	-1.040210
H	-1.892701	-3.532319	-1.784129
Ru	-0.415789	-0.649338	-0.155317
Se	-1.507278	1.346233	0.973405
C	-1.902111	2.852375	-0.201845
C	-2.782874	3.818475	0.309711
C	-1.327787	3.002657	-1.469871
C	-3.104654	4.939178	-0.468091
H	-3.226918	3.687187	1.301891
C	-1.649823	4.132643	-2.236270
H	-0.644843	2.242383	-1.856785
C	-2.537190	5.098621	-1.740903
H	-3.802043	5.686964	-0.076711
H	-1.206663	4.250398	-3.230770

H	-2.788951	5.975118	-2.346616
Br	1.244315	0.434990	-1.719143
O	1.214320	-0.813236	1.307351
C	1.939970	-0.084593	2.185934
O	3.174038	-0.143034	2.174914
C	1.138678	0.732188	3.160852
H	0.234697	0.182109	3.465499
H	1.771459	0.983026	4.024793
H	0.816677	1.666855	2.670990
O	0.254873	-2.513841	-0.941014
C	1.380225	-3.060906	-0.712441
C	1.613605	-4.420267	-1.340497
H	1.261208	-5.193920	-0.635106
H	1.043628	-4.521952	-2.275630
H	2.686314	-4.591321	-1.520561
O	2.316784	-2.573726	0.028533
H	1.820622	-1.592959	0.747095
Cs	4.660018	-0.608222	-0.510599
O	4.042667	2.334393	-0.033009
C	3.193740	2.820841	0.715965
O	1.867581	2.721376	0.502457
H	1.720970	2.122761	-0.293781
C	3.510921	3.541975	2.004095
H	4.491183	4.033940	1.927264
H	2.728913	4.268920	2.268756
H	3.563759	2.781199	2.804011

Statistical Thermodynamic Analysis
Temperature= 298.150 Kelvin   Pressure= 1.00000 Atm

===

SCF Energy=   -6558.90449288   Predicted Change= -5.548417D-09
Zero-point correction (ZPE)=   -6558.4507   0.45376
Internal Energy (U)=   -6558.4082   0.49622
Enthalpy (H)=   -6558.4073   0.49716
Gibbs Free Energy (G)=   -6558.5294   0.37507
Entropy (S)=   0.0004095

Frequencies --   -149.8150   13.1301   26.4009
Supporting Information: 035-Selenylated-Cluster-RuII-2HOAc-1OAc-CsBr-Decarboxylation-TS-34-PBE.log

----

Using Gaussian 16: ES64L-G16RevA.03 25-Dec-2016

===

# pbepbe/gen/auto pseudo=read gfprint gfinput empiricaldispersion=gd3bj
scf=(direct,vshift=200,tight,maxcycle=300,yqc)
opt=(gdiis,maxcycle=250,calcfc,ts,noeigentest) freq=noraman

#N Geom=AllCheck Guess=TCheck SCRF=Check GenChk RPBEPBE/Gen/Auto Freq

---

Pointgroup= C1 Stoichiometry= C16H28B10BrCsO8RuSe C1[X(C16H28B10BrCsO8RuSe)]
#Atoms= 66
Charge = 0 Multiplicity = 1

SCF Energy= -6558.86245536 Predicted Change= -6.271478D-10

===

Optimization completed. {Found 2 times}

Item	Max Val.	Criteria	Pass?	RMS Val.	Criteria	Pass?
Force	0.00000 ||	0.00045	[YES]	0.00000 ||	0.00030	[YES]
Displ	0.00097 ||	0.00180	[YES]	0.00097 ||	0.00180	[YES]

Atomic Type	Coordinates (Angstroms) X	Y	Z
B	3.070736	3.338971	0.508812
B	2.678362	4.258045	-0.957637
H	3.376033	5.131767	-1.389785
H	4.053066	3.514349	1.171067
B	0.912973	4.354161	-1.078365
B	1.801924	3.173257	-2.055666
B	1.544735	2.853121	1.287804
B	2.427240	1.690365	0.305056
H	1.363880	2.655140	2.450366
H	1.717350	-0.541970	2.545597
H	1.739746	3.205181	-3.250067
H	0.239855	5.193337	-1.602312
C	0.701797	1.846401	0.171962
C	0.364712	2.741036	-1.226703
B	1.688894	4.456458	0.517325
B	0.212973	3.498857	0.304817
H	-0.885597	3.746923	0.691111
H	1.648805	5.461560	1.168095
B	3.131340	2.534168	-1.082835
H	4.139646	2.128053	-1.585309
B	1.631267	1.588966	-1.267936
H	1.492426	0.601921	-1.907592
C	-0.890508	1.144755	1.144160
O	-1.792582	0.764589	0.282729
O	-0.942456	1.641869	2.254926
Cs	-3.760233	-1.481329	-0.807050
C	-0.896203	2.447381	-2.025474
H	-0.808030	2.956433	-2.997971
H	-1.001874	1.365894	-2.196403
H	-1.787398	2.826163	-1.504637

Ru	-0.287702	-0.806673	0.472051
O	-0.650279	-1.032639	2.594032
O	0.951347	-2.523844	0.703874
C	0.091491	-0.786617	3.572313
C	-0.080061	-3.279213	0.690324
O	1.381131	-0.534581	3.507008
O	-1.232865	-2.689144	0.596938
C	0.024853	-4.773308	0.737169
H	0.074472	-5.161865	-0.295756
H	0.946363	-5.068880	1.260772
H	-0.856684	-5.208911	1.232554
C	-0.481135	-0.731354	4.959879
H	0.263922	-1.028332	5.712369
H	-0.775155	0.316521	5.150866
H	-1.380530	-1.359898	5.023405
Se	3.323325	0.131605	1.166477
C	4.075285	-0.784721	-0.362476
C	5.466010	-0.690408	-0.538642
C	3.294373	-1.527305	-1.260034
C	6.075308	-1.335983	-1.623870
H	6.062226	-0.100909	0.164177
C	3.911195	-2.159354	-2.349078
H	2.214523	-1.604930	-1.124729
C	5.298426	-2.068866	-2.532764
H	7.158557	-1.255245	-1.762345
H	3.293759	-2.722123	-3.057453
H	5.774264	-2.565279	-3.385076
Br	-0.382183	-1.242031	-2.046126
O	-5.183198	1.179677	-0.447124
C	-4.906083	2.337181	-0.120177
O	-3.708936	2.713304	0.354487
H	-3.096289	1.915211	0.376198
C	-5.867312	3.500372	-0.206978
H	-5.463397	4.269247	-0.887204
H	-6.844069	3.154581	-0.570790
H	-5.977288	3.973635	0.783013

Statistical Thermodynamic Analysis
Temperature= 298.150 Kelvin   Pressure= 1.00000 Atm

==

SCF Energy= -6558.86245536 Predicted Change= -6.271478D-10
Zero-point correction (ZPE)= -6558.4051 0.45727
Internal Energy (U)= -6558.3626 0.49977
Enthalpy (H)= -6558.3617 0.50071
Gibbs Free Energy (G)= -6558.4854 0.37697
Entropy (S)= 0.00041505

Frequencies -- -199.1818 14.6082 21.5594

Supporting Information: 035-Selenylated-Cluster-RuII-2HOAc-1OAc-CsBr-Decarboxylation-TS-35-PBE.log

----
Using Gaussian 16: ES64L-G16RevA.03 25-Dec-2016
==
# pbepbe/gen/auto pseudo=read gfprint gfinput empiricaldispersion=gd3bj
scf=(direct,vshift=200,tight,maxcycle=300,yqc)
opt=(gdiis,maxcycle=250,calcfc,ts,noeigentest) freq=noraman
#N Geom=AllCheck Guess=TCheck SCRF=Check GenChk RPBEPBE/Gen/Auto Freq

----
Pointgroup= C1 Stoichiometry= C16H28B10BrCsO8RuSe C1[X(C16H28B10BrCsO8RuSe)]
#Atoms= 66
Charge = 0 Multiplicity = 1

SCF Energy= -6558.86245535 Predicted Change= -1.595082D-08
==
Optimization completed. {Found 1 times}

Item	Max Val.		Criteria	Pass?	RMS Val.		Criteria	Pass?
Force	0.00000	||	0.00045	[YES]	0.00000	||	0.00030	[YES]
Displ	0.00369	||	0.00180	[NO]	0.00369	||	0.00180	[YES]

Atomic Type	Coordinates (Angstroms) X	Y	Z
B	3.070645	3.339103	0.508536
B	2.678222	4.258100	-0.957947
H	3.375859	5.131825	-1.390144
H	4.052980	3.514536	1.170769
B	0.912828	4.354158	-1.078650
B	1.801800	3.173238	-2.055912
B	1.544670	2.853242	1.287574
B	2.427199	1.690468	0.304863
H	1.363845	2.655304	2.450149
H	1.717239	-0.540965	2.545698
H	1.739598	3.205106	-3.250313
H	0.239676	5.193290	-1.602624
C	0.701742	1.846446	0.171793
C	0.364610	2.741011	-1.226906
B	1.688771	4.456549	0.517023
B	0.212874	3.498896	0.304581
H	-0.885697	3.746951	0.690877
H	1.648664	5.461676	1.167752
B	3.131250	2.534228	-1.083075
H	4.139560	2.128111	-1.585542

B	1.631198	1.588978	-1.268111
H	1.492360	0.601913	-1.907736
C	-0.890518	1.144791	1.144038
O	-1.792598	0.764546	0.282648
O	-0.942473	1.641972	2.254779
Cs	-3.760181	-1.481613	-0.806725
C	-0.896309	2.447285	-2.025646
H	-0.808149	2.956268	-2.998179
H	-1.001973	1.365786	-2.196497
H	-1.787505	2.826097	-1.504830
Ru	-0.287625	-0.806651	0.472037
O	-0.650151	-1.032583	2.594038
O	0.951551	-2.523694	0.703889
C	0.091408	-0.785950	3.572327
C	-0.079787	-3.279160	0.690421
O	1.380934	-0.533326	3.507071
O	-1.232647	-2.689198	0.597020
C	0.025242	-4.773243	0.737413
H	0.073003	-5.162053	-0.295505
H	0.947646	-5.068678	1.259503
H	-0.855446	-5.208733	1.234420
C	-0.481304	-0.730630	4.959853
H	0.263363	-1.028788	5.712276
H	-0.774057	0.317511	5.151322
H	-1.381412	-1.358173	5.023024
Se	3.323348	0.131824	1.166418
C	4.075279	-0.784762	-0.362388
C	5.466071	-0.690897	-0.538273
C	3.294315	-1.527119	-1.260089
C	6.075387	-1.336703	-1.623353
H	6.062332	-0.101567	0.164649
C	3.911159	-2.159394	-2.348988
H	2.214412	-1.604411	-1.125002
C	5.298457	-2.069362	-2.532388
H	7.158690	-1.256318	-1.761605
H	3.293686	-2.721990	-3.057469
H	5.774313	-2.565955	-3.384586
Br	-0.382228	-1.242083	-2.046120
O	-5.183217	1.179431	-0.447309
C	-4.906176	2.336975	-0.120438
O	-3.709044	2.713213	0.354171
H	-3.096347	1.915155	0.375941
C	-5.867516	3.500074	-0.207248
H	-5.463570	4.269116	-0.887263
H	-6.844167	3.154224	-0.571294
H	-5.977760	3.973135	0.782812

Statistical Thermodynamic Analysis
Temperature= 298.150 Kelvin Pressure= 1.00000 Atm
==
SCF Energy= -6558.86245535 Predicted Change= -1.595082D-08
Zero-point correction (ZPE)= -6558.4051 0.45727
Internal Energy (U)= -6558.3626 0.49977
Enthalpy (H)= -6558.3617 0.50071
Gibbs Free Energy (G)= -6558.4854 0.37697
Entropy (S)= 0.00041505

Frequencies -- -199.1866 14.6107 21.5492
Supporting Information: 035-Selenylated-Cluster-RuII-2HOAc-1OAc-Decarboxylation-TS-01-PBE.log
----
Using Gaussian 16: ES64L-G16RevA.03 25-Dec-2016
==
# pbepbe/gen/auto pseudo=read gfprint gfinput empiricaldispersion=gd3bj
scf=(direct,vshift=200,tight,maxcycle=300,yqc)
opt=(gdiis,maxcycle=250,calcfc,ts,noeigentest) freq=noraman
#N Geom=AllCheck Guess=TCheck SCRF=Check GenChk RPBEPBE/Gen/Auto Freq
----
Pointgroup= C1 Stoichiometry= C16H28B10O8RuSe C1[X(C16H28B10O8RuSe)] #Atoms= 64
Charge = 0 Multiplicity = 1

SCF Energy= -3967.88085768 Predicted Change= -2.227383D-08
==
Optimization completed. {Found 1 times}

Item	Max Val.	Criteria	Pass?	RMS Val.	Criteria	Pass?
Force	0.00001 ||	0.00045	[YES]	0.00000 ||	0.00030	[YES]
Displ	0.00203 ||	0.00180	[NO]	0.00203 ||	0.00180	[YES]

Atomic Type	Coordinates (Angstroms) X	Y	Z
B	-3.468060	1.980687	-0.503774
B	-3.391367	2.812325	1.073400
H	-4.369706	3.114526	1.693788
H	-4.493800	1.657857	-1.031750
B	-1.959708	3.865583	1.072171
B	-1.902186	2.300521	1.919276
B	-2.049233	2.504184	-1.466061
B	-2.026112	0.980062	-0.605662
H	-1.940662	2.547173	-2.653178
H	-1.696095	2.250502	3.096583
H	-1.792939	4.879779	1.682148

C	-0.663456	2.026733	-0.554880
C	-0.618236	2.832845	0.926436
B	-2.898615	3.670109	-0.420758
B	-1.119258	3.675289	-0.487595
H	-0.376009	4.485951	-0.942067
H	-3.505515	4.590319	-0.887669
B	-2.835954	1.132473	0.952682
H	-3.377961	0.199623	1.466687
B	-1.058277	1.164104	0.845064
H	-0.394310	0.215487	1.525354
C	0.786586	1.805126	-1.597827
O	1.670361	2.610968	-1.331439
O	0.528494	0.924866	-2.434632
C	0.730860	3.206490	1.517625
H	0.692848	4.256017	1.847114
H	0.957683	2.569227	2.385025
H	1.513478	3.090461	0.753421
Ru	0.434051	-0.294113	0.119726
O	0.901388	-2.198783	0.881294
C	1.647111	-2.504953	-0.151543
O	1.653896	-1.642753	-1.093780
C	2.468643	-3.748049	-0.172865
H	2.125718	-4.463324	0.588656
H	2.437511	-4.204508	-1.174875
H	3.512065	-3.445210	0.034448
Se	-1.523675	-0.842411	-1.338812
C	-2.816517	-1.877923	-0.314244
C	-4.149855	-1.885862	-0.751122
C	-2.417707	-2.629231	0.799281
C	-5.099972	-2.635684	-0.044443
H	-4.449360	-1.293036	-1.621336
C	-3.375336	-3.382704	1.494403
H	-1.373486	-2.612466	1.124174
C	-4.714362	-3.383974	1.077715
H	-6.144535	-2.631232	-0.371862
H	-3.071408	-3.965758	2.370030
H	-5.459086	-3.967572	1.628073
O	2.206230	0.496165	1.046178
C	2.839093	0.060419	2.034100
O	2.505084	-1.032004	2.711462
C	4.070540	0.752194	2.534231
H	4.936432	0.207406	2.118235
H	4.091012	1.791099	2.177381
H	4.125054	0.710163	3.632811
H	1.786485	-1.511896	2.199766
H	3.130882	-0.932521	-1.766051

O	3.952476	-0.402797	-1.971988
C	4.780768	-0.497878	-0.921435
O	4.586969	-1.233702	0.051502
C	5.965967	0.430942	-1.059174
H	6.351166	0.425902	-2.090871
H	5.635407	1.462143	-0.841204
H	6.755733	0.143525	-0.351227

Statistical Thermodynamic Analysis
Temperature= 298.150 Kelvin Pressure= 1.00000 Atm

==

SCF Energy= -3967.88085768 Predicted Change= -2.227383D-08
Zero-point correction (ZPE)= -3967.4245 0.45633
Internal Energy (U)= -3967.3868 0.49397
Enthalpy (H)= -3967.3859 0.49491
Gibbs Free Energy (G)= -3967.4945 0.38635
Entropy (S)= 0.00036413

Frequencies -- -226.7799 15.5863 26.6260

Supporting Information: 035-Selenylated-Cluster-RuII-2HOAc-1OAc-Decarboxylation-TS-02-PBE.log

----

Using Gaussian 16: ES64L-G16RevA.03 25-Dec-2016

==

# pbepbe/gen/auto pseudo=read gfprint gfinput empiricaldispersion=gd3bj
scf=(direct,vshift=200,tight,maxcycle=300,yqc)
opt=(gdiis,maxcycle=250,calcfc,ts,noeigentest) freq=noraman
#N Geom=AllCheck Guess=TCheck SCRF=Check GenChk RPBEPBE/Gen/Auto Freq

----

Pointgroup= C1 Stoichiometry= C16H28B10O8RuSe C1[X(C16H28B10O8RuSe)] #Atoms= 64
Charge = 0 Multiplicity = 1

SCF Energy= -3967.87330539 Predicted Change= -7.553120D-09

==

Optimization completed. {Found 1 times}

Item	Max Val.		Criteria	Pass?	RMS Val.		Criteria	Pass?
Force	0.00000	||	0.00045	[YES]	0.00000	||	0.00030	[YES]
Displ	0.00821	||	0.00180	[NO]	0.00821	||	0.00180	[YES]

Atomic Type	Coordinates (Angstroms) X	Y	Z
B	-1.455715	-3.524407	-0.124593
B	-2.380017	-3.457229	1.402152
H	-2.421483	-4.371772	2.173688
H	-0.814753	-4.476370	-0.466073

B	-3.799660	-2.422720	1.144587
B	-2.378761	-1.770865	1.996265
B	-2.302566	-2.493353	-1.317521
B	-0.905906	-1.881662	-0.449184
H	-2.298811	-2.585073	-2.506449
H	-2.467714	-1.380758	3.123839
H	-4.856441	-2.472110	1.700742
C	-2.313252	-0.906026	-0.641938
C	-3.192754	-0.872783	0.795115
B	-3.239924	-3.479520	-0.167616
B	-3.758593	-1.816936	-0.528490
H	-4.715721	-1.425570	-1.117041
H	-3.911071	-4.396352	-0.544586
B	-0.931498	-2.454543	1.219646
H	0.071230	-2.623606	1.844494
B	-1.464151	-0.802038	0.817681
H	-0.801025	0.208259	1.379821
C	-2.453275	0.334685	-1.893164
O	-3.491404	0.984094	-1.802197
O	-1.488403	0.214192	-2.669152
C	-3.981647	0.377979	1.144485
H	-4.985676	0.078153	1.481395
H	-3.484151	0.930302	1.955281
H	-4.068168	1.018293	0.253868
Ru	-0.458720	0.955236	-0.140004
O	1.215321	2.124422	0.475551
C	1.264004	2.718934	-0.715225
O	0.392718	2.338803	-1.545117
C	2.298747	3.752582	-1.011781
H	3.154396	3.237474	-1.482557
H	1.895491	4.491866	-1.720197
H	2.643430	4.245309	-0.089600
Se	0.728132	-0.954226	-1.228047
C	2.036436	-1.725085	-0.006795
C	2.800914	-2.798993	-0.486829
C	2.236417	-1.229107	1.287747
C	3.757245	-3.394554	0.348479
H	2.644701	-3.174271	-1.503232
C	3.199975	-1.822091	2.115384
H	1.632413	-0.390115	1.645310
C	3.956676	-2.908122	1.649776
H	4.346268	-4.240344	-0.020410
H	3.358286	-1.431438	3.125433
H	4.702921	-3.375005	2.300589
O	-1.802769	2.518255	0.493653
C	-1.610883	3.412125	1.345412

O	-0.456485	3.606251	1.977397
C	-2.705414	4.360476	1.741636
H	-2.415426	5.393125	1.483371
H	-3.635585	4.094148	1.223006
H	-2.851963	4.326240	2.834395
H	0.233042	2.992740	1.570546
H	2.932570	1.664702	0.906140
O	3.876011	1.378359	1.060790
C	4.308007	0.855413	-0.113939
O	3.653893	0.906111	-1.153183
C	5.651684	0.183840	0.025082
H	6.289957	0.708311	0.752673
H	5.483871	-0.842217	0.398558
H	6.142225	0.126177	-0.956464

Statistical Thermodynamic Analysis
Temperature= 298.150 Kelvin Pressure= 1.00000 Atm
==

SCF Energy= -3967.87330539 Predicted Change= -7.553120D-09
Zero-point correction (ZPE)= -3967.4170 0.45625
Internal Energy (U)= -3967.3793 0.49394
Enthalpy (H)= -3967.3784 0.49488
Gibbs Free Energy (G)= -3967.4869 0.38631
Entropy (S)= 0.00036415

Frequencies -- -221.2752 19.4898 29.8058

Supporting Information: 041-Decarboxylated-Cluster-RuII-2HOAc-2OAc-CsBr-H-Migration-TS-01-PBE.log

----
Using Gaussian 16: ES64L-G16RevA.03 25-Dec-2016
==

# pbepbe/gen/auto pseudo=read gfprint gfinput empiricaldispersion=gd3bj
scf=(direct,vshift=200,tight,maxcycle=300,yqc)
opt=(gdiis,maxcycle=250,calcfc,ts,noeigentest) freq=noraman
#N Geom=AllCheck Guess=TCheck SCRF=Check GenChk RPBEPBE/Gen/Auto Freq

----
Pointgroup= C1 Stoichiometry= C15H28B10BrCsO6RuSe C1[X(C15H28B10BrCsO6RuSe)] #Atoms= 63
Charge = 0 Multiplicity = 1

SCF Energy= -6370.53521267 Predicted Change= -7.468775D-10
==

Optimization completed. {Found 2 times}

Item	Max Val.	Criteria	Pass?	RMS Val.	Criteria	Pass?
Force	0.00000 ||	0.00045	[YES]	0.00000 ||	0.00030	[YES]
Displ	0.00101 ||	0.00180	[YES]	0.00101 ||	0.00180	[YES]

Atomic Type	Coordinates (Angstroms) X	Y	Z
B	-2.687612	3.324374	-1.216453
B	-3.068810	4.244589	0.260989
H	-3.794115	5.198776	0.272928
H	-3.120456	3.589444	-2.302412
B	-1.651433	4.169303	1.330829
B	-3.040598	3.113162	1.635476
B	-1.028953	2.700076	-1.028997
B	-2.394511	1.651319	-0.707561
H	-0.284484	2.459018	-1.932904
H	-3.642305	3.182249	2.667583
H	-1.309570	4.960739	2.160946
C	-1.048154	1.611780	0.313793
C	-1.430938	2.508485	1.702565
B	-1.416156	4.307509	-0.418988
B	-0.393956	3.195235	0.537972
H	0.760972	3.313937	0.816520
H	-0.937435	5.289617	-0.911583
B	-3.687691	2.576725	0.064501
H	-4.847117	2.305622	-0.066752
B	-2.633883	1.510579	1.022906
H	-2.944966	0.509456	1.582773
C	-0.799675	2.088259	3.019673
H	-1.436062	1.356274	3.538350
H	0.187129	1.637171	2.833734
H	-0.669747	2.972426	3.662664
Ru	-0.242430	-0.320372	0.024641
Se	-2.181435	-0.160551	-1.608834
C	-3.660815	-1.106615	-0.775732
C	-4.964806	-0.628744	-0.981721
C	-3.425719	-2.273931	-0.037268
C	-6.046035	-1.319459	-0.418324
H	-5.135153	0.282139	-1.563058
C	-4.517927	-2.957380	0.517471
H	-2.403466	-2.638175	0.104191
C	-5.824464	-2.483275	0.333073
H	-7.063131	-0.942396	-0.567060
H	-4.338971	-3.867168	1.100230
H	-6.671226	-3.019796	0.773553
O	1.110326	-0.642580	1.780022
C	0.078576	-1.046868	2.441879
C	0.211728	-1.636598	3.819603
H	0.346614	-2.729896	3.734697

H	1.084606	-1.213558	4.341163
H	-0.704605	-1.453561	4.401516
O	-1.065027	-0.950634	1.877692
Cs	3.482453	-1.746083	0.189691
O	0.834391	0.056168	-1.821434
C	1.775066	0.891299	-1.704060
O	2.025814	1.428313	-0.535638
H	1.007446	0.969846	0.076154
C	2.633297	1.283931	-2.871639
H	2.487852	2.357828	-3.082034
H	3.698762	1.133936	-2.625372
H	2.358892	0.697282	-3.759176
Br	0.210048	-2.865748	-0.513901
O	5.184079	0.750448	-0.386782
C	5.322865	1.957690	-0.154664
O	4.306548	2.825424	-0.083475
H	3.443515	2.324112	-0.238813
C	6.658886	2.626841	0.077657
H	6.674449	3.100548	1.073743
H	7.469336	1.889777	-0.000476
H	6.810012	3.431224	-0.661708

Statistical Thermodynamic Analysis
Temperature= 298.150 Kelvin Pressure= 1.00000 Atm

==

SCF Energy= -6370.53521267 Predicted Change= -7.468775D-10
Zero-point correction (ZPE)= -6370.0943 0.44087
Internal Energy (U)= -6370.0550 0.48013
Enthalpy (H)= -6370.0541 0.48107
Gibbs Free Energy (G)= -6370.1695 0.36567
Entropy (S)= 0.00038706

Frequencies -- -677.0405 17.5687 24.3461

Supporting Information: 045-Decarboxylated-Cluster-RuII-2HOAc-1OAc-CsBr-01-Protonolysis-TS-01-PBE.log

----

Using Gaussian 16: ES64L-G16RevA.03 25-Dec-2016

==

# pbepbe/gen/auto pseudo=read gfprint gfinput empiricaldispersion=gd3bj
scf=(direct,vshift=200,tight,maxcycle=300,yqc)
opt=(gdiis,maxcycle=250,calcfc,ts,noeigentest) freq=noraman
#N Geom=AllCheck Guess=TCheck SCRF=Check GenChk RPBEPBE/Gen/Auto Freq

----

Pointgroup= C1 Stoichiometry= C15H28B10BrCsO6RuSe C1[X(C15H28B10BrCsO6RuSe)]
#Atoms= 63
Charge = 0 Multiplicity = 1

SCF Energy= -6370.53090341 Predicted Change= -2.682175D-07
===
Optimization completed on the basis of negligible forces. {Found 2 times}

Item	Max Val.	Criteria	Pass?	RMS Val.	Criteria	Pass?
Force	0.00000 ||	0.00045	[YES]	0.00000 ||	0.00030	[YES]
Displ	0.04347 ||	0.00180	[NO]	0.04347 ||	0.00180	[NO]

Atomic Type	Coordinates (Angstroms) X	Y	Z
B	3.351462	-1.737307	-2.332931
B	4.340994	-2.843402	-1.344526
H	5.374336	-3.316346	-1.723261
H	3.639858	-1.392190	-3.443254
B	3.242665	-3.776361	-0.302604
B	4.126882	-2.393510	0.364136
B	1.649935	-2.014916	-1.891894
B	2.521268	-0.644905	-1.212173
H	0.700488	-1.829929	-2.586827
H	4.887533	-2.542100	1.275139
H	3.407418	-4.867216	0.159206
C	1.516525	-1.581867	-0.219937
C	2.455874	-2.624756	0.696995
B	2.756668	-3.388423	-1.965132
B	1.578401	-3.264176	-0.638376
H	0.617746	-3.928244	-0.400731
H	2.625017	-4.240577	-2.795773
B	4.195629	-1.120136	-0.882783
H	5.098225	-0.334458	-0.918027
B	3.022313	-1.022927	0.442699
H	3.003162	-0.256960	1.348915
C	1.952447	-2.984341	2.085402
H	2.396515	-2.318602	2.840199
H	0.855996	-2.887029	2.127854
H	2.224865	-4.025527	2.315800
Ru	0.046736	-0.065290	0.380218
Se	1.503873	1.102020	-1.338230
C	2.747642	2.231880	-0.356770
C	4.003067	2.494449	-0.927489
C	2.370967	2.812790	0.861143
C	4.901768	3.333330	-0.254709
H	4.282679	2.038855	-1.882453
C	3.277990	3.654784	1.521720
H	1.386890	2.603105	1.288077
C	4.540862	3.913639	0.970419

H	5.886068	3.530954	-0.691459
H	2.990235	4.107683	2.476273
H	5.244905	4.568434	1.494077
O	-0.929403	-0.888374	2.220745
C	-0.007316	-0.336429	2.934712
C	-0.072258	-0.369050	4.439781
H	-0.660756	0.494889	4.797473
H	-0.563668	-1.291312	4.787768
H	0.939225	-0.291440	4.866611
O	0.951529	0.253700	2.330809
Cs	-3.734077	-0.767448	1.084889
O	-1.089464	-0.315908	-1.377664
C	-1.700257	-1.452130	-1.581310
O	-1.789387	-2.382155	-0.745739
H	0.158516	-1.682595	0.111688
C	-2.361635	-1.562736	-2.951375
H	-2.190493	-2.571971	-3.358132
H	-3.451298	-1.416576	-2.845471
H	-1.970113	-0.803140	-3.644642
Br	-1.319901	2.056081	0.808700
O	-4.321062	0.820495	-1.446390
C	-3.818321	1.528913	-2.324512
O	-2.665284	2.193511	-2.197692
H	-2.258725	1.991379	-1.300332
C	-4.428363	1.716478	-3.697292
H	-3.940369	1.017632	-4.400135
H	-5.502787	1.487338	-3.664148
H	-4.259225	2.737236	-4.072913

Statistical Thermodynamic Analysis
Temperature= 298.150 Kelvin Pressure= 1.00000 Atm

==

SCF Energy= -6370.53090341 Predicted Change= -2.682175D-07
Zero-point correction (ZPE)= -6370.0900 0.44082
Internal Energy (U)= -6370.0505 0.48036
Enthalpy (H)= -6370.0495 0.48131
Gibbs Free Energy (G)= -6370.1653 0.36558
Entropy (S)= 0.00038814

Frequencies -- -355.3830 15.2383 27.9444

Supporting Information: 045-Decarboxylated-Cluster-RuII-2HOAc-1OAc-CsBr-01-Protonolysis-TS-02-PBE.log

----

Using Gaussian 16: ES64L-G16RevA.03 25-Dec-2016

==

pbepbe/gen/auto pseudo=read gfprint gfinput empiricaldispersion=gd3bj

scf=(direct,vshift=200,tight,maxcycle=300,yqc)
opt=(gdiis,maxcycle=250,calcfc,ts,noeigentest) freq=noraman
#N Geom=AllCheck Guess=TCheck SCRF=Check GenChk RPBEPBE/Gen/Auto Freq

---

Pointgroup= C1 Stoichiometry= C15H28B10BrCsO6RuSe C1[X(C15H28B10BrCsO6RuSe)]
#Atoms= 63
Charge = 0 Multiplicity = 1

SCF Energy= -6370.53644955 Predicted Change= -1.007832D-09

===

Optimization completed. {Found 1 times}

Item	Max Val.	Criteria	Pass?	RMS Val.	Criteria	Pass?
Force	0.00000 ||	0.00045	[YES]	0.00000 ||	0.00030	[YES]
Displ	0.00188 ||	0.00180	[NO]	0.00188 ||	0.00180	[YES]

Atomic Type	Coordinates (Angstroms) X	Y	Z
B	-2.575006	3.389651	-1.101432
B	-3.040795	4.238520	0.396023
H	-3.782035	5.179270	0.414554
H	-2.959035	3.697903	-2.193519
B	-1.674717	4.136038	1.530147
B	-3.059768	3.047456	1.719483
B	-0.917475	2.783015	-0.868651
B	-2.287218	1.696983	-0.665915
H	-0.117716	2.574460	-1.727764
H	-3.708180	3.060557	2.724356
H	-1.385278	4.890371	2.411889
C	-1.002569	1.653183	0.437764
C	-1.446477	2.462523	1.837441
B	-1.361500	4.357123	-0.202939
B	-0.362640	3.235779	0.749274
H	0.781101	3.317715	1.074780
H	-0.876917	5.365113	-0.630313
B	-3.624140	2.573535	0.094736
H	-4.769147	2.286665	-0.104777
B	-2.615766	1.476827	1.056669
H	-2.926673	0.447757	1.559677
C	-0.863757	1.965948	3.150538
H	-1.544951	1.245211	3.626159
H	0.105606	1.473622	2.972742
H	-0.707887	2.819189	3.828041
Ru	-0.261770	-0.379958	0.127543
Se	-2.057632	-0.062539	-1.644843
C	-3.659941	-0.958555	-0.997201

C	-4.906916	-0.457816	-1.403801
C	-3.565328	-2.102312	-0.194132
C	-6.076974	-1.101449	-0.978757
H	-4.966129	0.433606	-2.035905
C	-4.744701	-2.739016	0.220576
H	-2.585688	-2.485290	0.104285
C	-5.997374	-2.241609	-0.165377
H	-7.051376	-0.707141	-1.284800
H	-4.676942	-3.630901	0.852278
H	-6.913001	-2.742162	0.165928
O	0.960704	-0.896953	1.915467
C	-0.113084	-1.302610	2.505658
C	-0.051774	-1.980489	3.848137
H	0.070637	-3.068153	3.698127
H	0.805335	-1.610052	4.431975
H	-0.989731	-1.817427	4.400587
O	-1.229165	-1.129142	1.905674
Cs	3.413934	-1.666139	0.244216
O	0.938710	0.138987	-1.541768
C	1.930953	0.951495	-1.416313
O	2.343616	1.402554	-0.301428
H	0.316672	1.074305	0.579978
C	2.635299	1.336144	-2.706587
H	2.593087	2.432253	-2.826490
H	3.700818	1.053196	-2.646153
H	2.166210	0.852066	-3.574239
Br	0.058187	-2.822921	-0.561396
O	5.423691	0.507564	-0.521421
C	5.609583	1.729776	-0.426457
O	4.637612	2.632680	-0.319180
H	3.717160	2.151147	-0.327226
C	6.986815	2.362738	-0.429225
H	7.129960	2.962678	0.485158
H	7.761046	1.586094	-0.495115
H	7.078749	3.054460	-1.283771

Statistical Thermodynamic Analysis
Temperature= 298.150 Kelvin Pressure= 1.00000 Atm

==

SCF Energy= -6370.53644955 Predicted Change= -1.007832D-09
Zero-point correction (ZPE)= -6370.0955 0.44088
Internal Energy (U)= -6370.0562 0.48019
Enthalpy (H)= -6370.0553 0.48113
Gibbs Free Energy (G)= -6370.1707 0.36566
Entropy (S)= 0.0003873

Frequencies -- -412.2274 17.7041 25.5886

Supporting Information: 045-Decarboxylated-Cluster-RuII-2HOAc-1OAc-CsBr-01-Protonolysis-TS-03-PBE.log

----

Using Gaussian 16: ES64L-G16RevA.03 25-Dec-2016

===

# pbepbe/gen/auto pseudo=read gfprint gfinput empiricaldispersion=gd3bj
scf=(direct,vshift=200,tight,maxcycle=300,yqc)
opt=(gdiis,maxcycle=250,calcfc,ts,noeigentest) freq=noraman
#N Geom=AllCheck Guess=TCheck SCRF=Check GenChk RPBEPBE/Gen/Auto Freq

----

Pointgroup= C1 Stoichiometry= C15H28B10BrCsO6RuSe C1[X(C15H28B10BrCsO6RuSe)]
#Atoms= 63
Charge = 0 Multiplicity = 1

SCF Energy= -6370.52952124 Predicted Change= -1.641485D-08

===

Optimization completed. {Found 1 times}

Item	Max Val.	Criteria	Pass?	RMS Val.	Criteria	Pass?
Force	0.00002 ||	0.00045	[YES]	0.00000 ||	0.00030	[YES]
Displ	0.00289 ||	0.00180	[NO]	0.00289 ||	0.00180	[YES]

Atomic Type	Coordinates (Angstroms) X	Y	Z
B	-2.991827	2.714501	-1.663058
B	-3.388084	3.867409	-0.363749
H	-4.257502	4.686795	-0.452973
H	-3.555612	2.671640	-2.719492
B	-1.886682	4.228854	0.520413
B	-3.051561	3.075289	1.193168
B	-1.244170	2.390339	-1.566333
B	-2.388149	1.247048	-0.880763
H	-0.546904	2.056318	-2.473397
H	-3.557967	3.278759	2.257491
H	-1.609856	5.220235	1.129486
C	-0.972424	1.642506	-0.027219
C	-1.362904	2.738683	1.186649
B	-1.838349	4.019704	-1.240803
B	-0.560118	3.317127	-0.222466
H	0.583341	3.628369	-0.114864
H	-1.577642	4.929382	-1.974659
B	-3.737031	2.124801	-0.148566
H	-4.836857	1.656103	-0.083440
B	-2.451778	1.451409	0.872263
H	-2.532048	0.562454	1.655470

C	-0.566757	2.697790	2.480108
H	-0.812692	1.790323	3.050513
H	0.513429	2.723598	2.270526
H	-0.826206	3.579593	3.085858
Ru	-0.021434	-0.339119	0.040895
Se	-2.045271	-0.672198	-1.420872
C	-3.436749	-1.506872	-0.342659
C	-4.769642	-1.325949	-0.743837
C	-3.115265	-2.295492	0.768913
C	-5.796834	-1.929547	-0.005707
H	-5.005910	-0.708201	-1.616094
C	-4.152995	-2.896844	1.496921
H	-2.070951	-2.434354	1.059720
C	-5.489788	-2.714775	1.115441
H	-6.838351	-1.781920	-0.309115
H	-3.907978	-3.511509	2.369440
H	-6.293987	-3.184817	1.690783
O	1.549253	-0.264719	1.636490
C	0.647369	-0.557925	2.529271
C	1.042182	-0.756396	3.967278
H	1.572674	-1.719221	4.072393
H	1.730055	0.043138	4.289906
H	0.150536	-0.771386	4.610524
O	-0.557153	-0.670273	2.140734
Cs	3.667026	-1.447922	-0.442592
O	0.891074	-0.189761	-1.855921
C	1.672772	0.835667	-2.072323
O	2.005339	1.685406	-1.214179
H	0.429266	1.252657	-0.059120
C	2.166039	0.946775	-3.510266
H	1.528785	1.680439	-4.035750
H	3.198443	1.332420	-3.534263
H	2.091382	-0.012439	-4.045562
Br	0.429205	-2.863506	-0.047116
O	4.530178	1.208227	0.747799
C	3.864545	2.203749	1.040747
O	2.733481	2.146390	1.768789
H	2.432817	1.185343	1.807267
C	4.187468	3.607685	0.597803
H	3.875885	4.352096	1.346250
H	5.259324	3.702095	0.373569
H	3.607494	3.787573	-0.325499

Statistical Thermodynamic Analysis
Temperature= 298.150 Kelvin Pressure= 1.00000 Atm

===

SCF Energy=   -6370.52952124   Predicted Change= -1.641485D-08
Zero-point correction (ZPE)=   -6370.0886   0.44090
Internal Energy (U)=   -6370.0493   0.48019
Enthalpy (H)=   -6370.0483   0.48114
Gibbs Free Energy (G)=   -6370.1627   0.36677
Entropy (S)=   0.00038359

Frequencies --   -468.5412   20.4046   30.0919

Supporting Information: 047-Decarboxylated-Cluster-RuII-2HOAc-1OAc-CsBr-01-Protonolysis-TS-01-PBE.log

---

Using Gaussian 16: ES64L-G16RevA.03 25-Dec-2016

===

# pbepbe/gen/auto pseudo=read gfprint gfinput empiricaldispersion=gd3bj
scf=(direct,vshift=200,tight,maxcycle=300,yqc)
opt=(gdiis,maxcycle=250,calcfc,ts,noeigentest) freq=noraman
#N Geom=AllCheck Guess=TCheck SCRF=Check GenChk RPBEPBE/Gen/Auto Freq

---

Pointgroup= C1   Stoichiometry= C15H28B10BrCsO6RuSe   C1[X(C15H28B10BrCsO6RuSe)]
#Atoms= 63
Charge = 0   Multiplicity = 1

SCF Energy= -6370.50508858   Predicted Change= -1.437274D-08

===

Optimization completed.   {Found   2   times}

Item	Max Val.	Criteria	Pass?	RMS Val.	Criteria	Pass?
Force	0.00001 ||	0.00045	[YES]	0.00000 ||	0.00030	[YES]
Displ	0.00137 ||	0.00180	[YES]	0.00137 ||	0.00180	[YES]

Atomic Type	Coordinates (Angstroms) X	Y	Z
B	-3.204153	2.806002	0.853017
B	-2.704067	2.901826	2.563708
H	-3.435006	3.284810	3.431414
H	-4.299834	3.098615	0.466585
B	-0.964844	3.280340	2.608960
B	-1.512227	1.607903	2.886262
B	-1.752519	3.095271	-0.154514
B	-2.310298	1.462663	0.149536
H	-1.694135	3.524239	-1.266590
H	-1.283980	1.057765	3.923365
H	-0.362752	3.859106	3.463794
C	-0.640367	1.840280	0.274150
C	-0.184867	1.962912	1.870122
B	-1.991539	4.013955	1.354399

B	-0.379548	3.405787	0.936424
H	0.609004	3.993085	0.634415
H	-2.193065	5.193827	1.339110
B	-2.900955	1.307933	1.806240
H	-3.752900	0.519962	2.093743
B	-1.283705	0.714631	1.372544
H	-1.012442	-0.552596	1.519134
C	1.238238	1.561794	2.218632
H	1.312463	0.473422	2.365537
H	1.926554	1.887843	1.416208
H	1.525687	2.062628	3.156138
Ru	-0.467206	-0.663969	-0.156703
Se	-2.572153	0.040286	-1.261130
C	-4.048510	-0.896583	-0.404770
C	-5.318882	-0.302821	-0.435557
C	-3.843787	-2.151276	0.182072
C	-6.398738	-0.969880	0.158474
H	-5.459877	0.680405	-0.895675
C	-4.935407	-2.811656	0.766563
H	-2.846135	-2.598152	0.190955
C	-6.208100	-2.223187	0.759678
H	-7.389893	-0.505214	0.153141
H	-4.784849	-3.791173	1.232237
H	-7.053901	-2.741120	1.222939
O	1.326138	-1.745786	0.751664
C	0.632198	-2.831574	0.640365
C	1.179891	-4.170122	1.043257
H	1.847449	-4.541485	0.245420
H	1.771474	-4.078221	1.968485
H	0.363360	-4.893651	1.180646
O	-0.549840	-2.724010	0.158077
Cs	3.489997	-0.106736	-1.091253
Br	0.383181	-1.266343	-2.467527
H	0.265659	1.724697	-0.574665
O	1.118565	2.223683	-1.640310
C	2.125423	2.971291	-1.308200
C	2.362409	4.177720	-2.218602
H	2.173781	3.917421	-3.272867
H	1.638558	4.967392	-1.946607
H	3.379248	4.579572	-2.091899
O	2.888398	2.773356	-0.322705
O	4.593747	-1.235257	1.583371
C	4.155259	-1.459849	2.711768
O	2.858854	-1.730166	2.965998
C	4.989667	-1.451618	3.971921
H	4.822778	-2.374514	4.551170

H	6.052696	-1.348160	3.716436
H	4.681122	-0.607389	4.612562
H	2.353856	-1.704420	2.089650

Statistical Thermodynamic Analysis
Temperature= 298.150 Kelvin   Pressure= 1.00000 Atm

==

SCF Energy=   -6370.50508858   Predicted Change= -1.437274D-08
Zero-point correction (ZPE)=   -6370.0651   0.43989
Internal Energy (U)=   -6370.0253   0.47969
Enthalpy (H)=   -6370.0244   0.48063
Gibbs Free Energy (G)=   -6370.1423   0.36275
Entropy (S)=   0.00039539

Frequencies --   -226.7378   17.9089   22.9436

16. Ground State Solvation Single Point Energies

Supporting Information: 000-2-OAc-Active-RuII-Catalyst-02-SP-TFE-PBE.log

----

Using Gaussian 16: ES64L-G16RevA.03 25-Dec-2016

===

# pbepbe/Def2QZVP/auto empiricaldispersion=gd3bj
scf=(maxcycle=300,direct,vshift=200,tight,yqc) density=current
scrf=(solvent=2,2,2-Trifluoroethanol,smd)

Pointgroup= C1 Stoichiometry= C14H20O4Ru C1[X(C14H20O4Ru)] #Atoms= 39
Charge = 0 Multiplicity = 1

SCF Energy= -940.937630536

===

Optimization incomplete.

Atomic Type	Coordinates (Angstroms) X	Y	Z
Ru	-0.081130	-0.395829	-0.065080
C	1.140179	-1.155621	-1.742900
C	0.683808	-2.253740	-0.928070
C	1.819121	-0.050232	-1.143690
H	0.851059	-1.108311	-2.795760
C	1.011138	-2.260601	0.470600
C	2.077681	0.012818	0.260270
H	1.990852	0.840058	-1.753980
C	1.632379	-1.113862	1.050350
H	0.615676	-3.050210	1.115180
H	1.701659	-1.046152	2.141470
C	-0.169204	-3.344009	-1.516620
H	0.449645	-4.228180	-1.756230
H	-0.954035	-3.648578	-0.805650
H	-0.660704	-3.003938	-2.441730
C	2.714633	1.213367	0.936250
H	2.275413	1.266207	1.951530
C	2.408805	2.540547	0.224410
H	1.329605	2.636369	0.015960
H	2.731676	3.387007	0.853670
H	2.955145	2.620526	-0.733080
C	4.233652	0.984794	1.085200
H	4.697444	1.824704	1.631080
H	4.453531	0.053514	1.635410

H	4.714662	0.915414	0.093210
O	-2.106251	-1.122436	0.014590
C	-2.282250	-0.541546	1.150240
O	-1.251369	0.015013	1.686860
C	-3.620490	-0.518984	1.821370
H	-3.504740	-0.465664	2.914660
H	-4.140288	0.386937	1.468290
H	-4.209411	-1.404353	1.536780
O	-0.677077	1.415452	-0.863770
C	-1.822906	2.068024	-0.730180
O	-2.837016	1.709285	-0.126470
C	-1.755004	3.423113	-1.444910
H	-1.028603	4.079072	-0.933560
H	-1.408624	3.299003	-2.484400
H	-2.745803	3.900135	-1.432960

Supporting Information: 000-AcOH-SP-TFE-PBE.log

Using Gaussian 16: ES64L-G16RevA.03 25-Dec-2016

==

# pbepbe/Def2QZVP/auto empiricaldispersion=gd3bj
scf=(maxcycle=300,direct,vshift=200,tight,yqc) density=current
scrf=(solvent=2,2,2-Trifluoroethanol,smd)

Pointgroup= C1 Stoichiometry= C2H4O2 C1[X(C2H4O2)] #Atoms= 8
Charge = 0 Multiplicity = 1

SCF Energy= -228.954120103

==

Optimization incomplete.

Atomic Type	Coordinates (Angstroms) X	Y	Z
C	-0.092612	0.125859	0.000541
O	-0.662494	1.205393	-0.000169
O	-0.768859	-1.063928	-0.000129
H	-1.720202	-0.805589	-0.000449
C	1.401930	-0.098074	-0.000009
H	1.699027	-0.681771	0.887521
H	1.698407	-0.682251	-0.887419
H	1.917680	0.871181	-0.000459

Supporting Information: 000-Borane-Cluster-SePh-Added-SP-TFE-PBE.log

----

Using Gaussian 16: ES64L-G16RevA.03 25-Dec-2016

==

# pbepbe/Def2QZVP/auto empiricaldispersion=gd3bj
scf=(maxcycle=300,direct,vshift=200,tight,yqc) density=current
scrf=(solvent=2,2,2-Trifluoroethanol,smd)

Pointgroup= C1 Stoichiometry= C9H18B10Se C1[X(C9H18B10Se)] #Atoms= 38
Charge = 0 Multiplicity = 1

SCF Energy= -3003.17688754

==

Optimization incomplete.

Atomic Type	Coordinates (Angstroms) X	Y	Z
B	0.863299	1.014870	-1.403449
B	2.626629	1.146171	-1.637359
H	3.121078	1.610791	-2.623579
H	0.065999	1.384950	-2.215549
B	3.388949	-0.222549	-0.795309
B	3.375129	1.392551	-0.042879
B	0.535229	-0.436000	-0.423669
B	0.530709	1.176280	0.340031
H	-0.457221	1.568950	0.882381
H	4.405338	1.940571	0.215601
H	4.431629	-0.757049	-1.032149
C	1.369199	-0.116090	1.053891
C	2.994819	-0.005179	0.857571
B	1.842219	-0.461940	-1.639099
B	2.105639	-1.190020	-0.049719
H	2.229690	-2.327690	0.288431
H	1.761009	-1.162260	-2.605459
B	1.808188	2.163400	-0.414219
H	1.706818	3.352190	-0.506029
B	2.082569	1.430710	1.169301
H	2.191508	1.909650	2.256651
H	1.024619	-0.568800	1.986381
Se	-1.038651	-1.648791	-0.404019
C	-2.418751	-0.350981	-0.013579
C	-3.044961	-0.363081	1.243281
C	-2.823651	0.573119	-0.991469
C	-4.066711	0.555869	1.524841
H	-2.725471	-1.087191	1.999021
C	-3.835051	1.498559	-0.699629

H	-2.339351	0.569099	-1.971789
C	-4.459301	1.490938	0.556681
H	-4.550961	0.543208	2.506921
H	-4.139832	2.223629	-1.461369
H	-5.252632	2.211708	0.779661
C	3.894559	-0.524849	1.968711
H	3.556839	-1.512759	2.319501
H	4.918259	-0.631159	1.579211
H	3.913109	0.173851	2.818941

Supporting Information: 000-CO2-SP-TFE-PBE.log

Using Gaussian 16: ES64L-G16RevA.03 25-Dec-2016

==

# pbepbe/Def2QZVP/auto empiricaldispersion=gd3bj
scf=(maxcycle=300,direct,vshift=200,tight,yqc) density=current
scrf=(solvent=2,2,2-Trifluoroethanol,smd)

Pointgroup= D*H Stoichiometry= CO2 D*H[O(C),C*(O.O)] #Atoms= 3
Charge = 0 Multiplicity = 1

SCF Energy= -188.480094654

==

Optimization incomplete.

Atomic Type	Coordinates (Angstroms) X	Y	Z
C	0.000000	0.000000	0.000000
O	0.000000	0.000000	1.180810
O	0.000000	0.000000	-1.180810

Supporting Information: 000-Cs-Borane-Cluster-SP-TFE-PBE.log

Using Gaussian 16: ES64L-G16RevA.03 25-Dec-2016

==

# pbepbe/Def2QZVP/auto empiricaldispersion=gd3bj
scf=(maxcycle=300,direct,vshift=200,tight,yqc) density=current
scrf=(solvent=2,2,2-Trifluoroethanol,smd)

Pointgroup= C1 Stoichiometry= C4H13B10CsO2 C1[X(C4H13B10CsO2)] #Atoms= 30
Charge = 0 Multiplicity = 1

SCF Energy= -579.182118816

==

Optimization incomplete.

Atomic Type	Coordinates (Angstroms) X	Y	Z
B	-3.328448	-1.859202	0.280660
B	-4.427009	-0.457392	0.267170
H	-5.614349	-0.551073	0.401870
H	-3.707728	-2.986842	0.428140
B	-3.726110	0.768098	-0.816750
B	-3.549430	0.927738	0.942420
B	-1.965149	-1.499711	-0.799760
B	-1.768629	-1.326031	0.956480
H	-1.288228	-2.266120	-1.411480
H	-0.953178	-1.975540	1.544390
H	-3.988351	1.889048	1.504430
H	-4.286790	1.618128	-1.445980
C	-1.195199	-0.130210	-0.114030
C	-2.233680	1.198379	-0.116580
B	-3.597319	-0.953872	-1.235780
B	-2.203000	0.120389	-1.470440
H	-1.697950	0.527389	-2.473040
H	-4.170789	-1.406252	-2.185800
B	-3.286639	-0.685742	1.628910
H	-3.630739	-0.948952	2.746610
B	-1.910870	0.399659	1.365030
H	-1.210290	0.958490	2.150330
C	0.346921	0.066551	-0.273190
O	0.923771	-0.843049	-0.926290
O	0.858390	1.076031	0.299750
Cs	3.602291	-0.091758	0.074800
C	-1.659381	2.592369	-0.311480
H	-0.593561	2.585920	-0.035460
H	-1.774181	2.910659	-1.359000
H	-2.206091	3.295749	0.335250

Supporting Information: 000-CsBr-SP-TFE-PBE.log

Using Gaussian 16: ES64L-G16RevA.03 25-Dec-2016

==

# pbepbe/Def2QZVP/auto empiricaldispersion=gd3bj
scf=(maxcycle=300,direct,vshift=200,tight,yqc) density=current
scrf=(solvent=2,2,2-Trifluoroethanol,smd)

Pointgroup= C*V Stoichiometry= BrCs C*V[C*(BrCs)] #Atoms= 2
Charge = 0 Multiplicity = 1

SCF Energy= -2594.11767462

==

Optimization incomplete.

Atomic Type	Coordinates (Angstroms) X	Y	Z
Cs	0.000000	0.000000	1.277784
Br	0.000000	0.000000	-2.007946

Supporting Information: 000-CsOAc-SP-TFE-PBE.log

Using Gaussian 16: ES64L-G16RevA.03 25-Dec-2016

==

# pbepbe/Def2QZVP/auto empiricaldispersion=gd3bj
scf=(maxcycle=300,direct,vshift=200,tight,yqc) density=current
scrf=(solvent=2,2,2-Trifluoroethanol,smd)

Pointgroup= C1 Stoichiometry= C2H3CsO2 C1[X(C2H3CsO2)] #Atoms= 8
Charge = 0 Multiplicity = 1

SCF Energy= -248.614395787

==

Optimization incomplete.

Atomic Type	Coordinates (Angstroms) X	Y	Z
C	2.011450	0.010331	-0.000038
O	1.438620	1.149671	-0.000028
O	1.442810	-1.132659	-0.000028
C	3.556700	-0.004139	-0.000008
H	3.923360	-0.551869	0.885972
H	3.923420	-0.552599	-0.885498
H	3.971000	1.015871	-0.000388
Cs	-1.241420	-0.001539	0.000012

Supporting Information: 000-p-Cymene-SP-TFE-PBE.log

Using Gaussian 16: ES64L-G16RevA.03 25-Dec-2016

==

# pbepbe/Def2QZVP/auto empiricaldispersion=gd3bj
scf=(maxcycle=300,direct,vshift=200,tight,yqc) density=current

scrf=(solvent=2,2,2-Trifluoroethanol,smd)

Pointgroup= C1 Stoichiometry= C10H14 C1[X(C10H14)] #Atoms= 24
Charge = 0 Multiplicity = 1

SCF Energy= -389.167789083

===

Optimization incomplete.

Atomic Type	Coordinates (Angstroms) X	Y	Z
C	0.029698	-1.047789	-0.006381
C	-1.361352	-1.190799	-0.011891
C	-2.210102	-0.065939	-0.006651
C	-1.611202	1.204931	-0.001181
C	-0.216242	1.347561	0.004539
C	0.630698	0.227051	0.002479
H	0.659638	-1.944959	-0.012751
H	-1.801602	-2.195259	-0.022071
H	-2.246712	2.098421	-0.002491
H	0.224608	2.351761	0.006919
C	-3.712912	-0.227668	0.007519
H	-4.073732	-0.560878	0.998649
H	-4.222782	0.721592	-0.227211
H	-4.046222	-0.982808	-0.726111
C	2.145128	0.389071	0.005019
H	2.358708	1.475671	0.013539
C	2.776968	-0.222949	1.270479
H	3.868988	-0.057850	1.281119
H	2.349338	0.222111	2.184729
H	2.601698	-1.312719	1.313119
C	2.779258	-0.203469	-1.268571
H	2.353348	0.255771	-2.176601
H	3.871338	-0.038460	-1.274691
H	2.603748	-1.292389	-1.328321

Supporting Information: 000-PhSeBr-SP-TFE-PBE.log

Using Gaussian 16: ES64L-G16RevA.03 25-Dec-2016

===

# pbepbe/Def2QZVP/auto empiricaldispersion=gd3bj
scf=(maxcycle=300,direct,vshift=200,tight,yqc) density=current
scrf=(solvent=2,2,2-Trifluoroethanol,smd)

Pointgroup= C1 Stoichiometry= C6H5BrSe C1[X(C6H5BrSe)] #Atoms= 13
Charge = 0 Multiplicity = 1

SCF Energy= -5206.55528830

===

Optimization incomplete.

Atomic Type	Coordinates (Angstroms) X	Y	Z
C	2.966912	-0.421151	1.217490
C	1.664302	0.093089	1.221260
C	1.013422	0.351839	-0.000020
C	1.664282	0.092909	-1.221270
C	2.966892	-0.421341	-1.217450
C	3.616822	-0.677621	0.000030
H	3.475132	-0.622991	2.165700
H	1.142662	0.293779	2.161370
H	1.142622	0.293459	-2.161410
H	3.475092	-0.623311	-2.165640
H	4.635082	-1.080071	0.000050
Se	-0.742018	1.119909	-0.000060
Br	-2.057078	-0.869831	0.000050

Supporting Information: 010-Cs-Cluster-RuII-1HOAc-2OAc-Cymene-01-SP-TFE-PBE.log

---

Using Gaussian 16: ES64L-G16RevA.03 25-Dec-2016

===

# pbepbe/Def2QZVP/auto empiricaldispersion=gd3bj
scf=(maxcycle=300,direct,vshift=200,tight,yqc) density=current
scrf=(solvent=2,2,2-Trifluoroethanol,smd)

Pointgroup= C1 Stoichiometry= C20H37B10CsO8Ru C1[X(C20H37B10CsO8Ru)] #Atoms= 77
Charge = 0 Multiplicity = 1

SCF Energy= -1749.09203596

===

Optimization incomplete.

Atomic Type	Coordinates (Angstroms) X	Y	Z
B	-4.539370	0.348188	1.446109

B	-5.944260	-0.274602	0.548189
H	-7.065880	0.081208	0.765919
H	-4.613130	1.169228	2.316399
B	-5.590410	-1.950732	0.081309
B	-5.415690	-0.650352	-1.111421
B	-3.325000	-0.948782	1.531839
B	-3.163710	0.337668	0.332319
H	-2.488730	-1.139231	2.360589
H	-2.410940	1.332329	0.277849
H	-6.058590	-0.657382	-2.118771
H	-6.354669	-2.847102	-0.118751
C	-2.898030	-1.266342	-0.102651
C	-4.230110	-1.868222	-0.954841
B	-5.046400	-1.353662	1.659349
B	-3.971340	-2.369152	0.673009
H	-3.575959	-3.478172	0.850189
H	-5.497840	-1.783652	2.680549
B	-4.763370	0.779798	-0.281571
H	-4.999180	1.893388	-0.651561
B	-3.691420	-0.258472	-1.251411
H	-3.126870	-0.039442	-2.276091
C	-1.466570	-1.776331	-0.389641
O	-0.522850	-0.990511	0.021489
O	-1.277059	-2.880161	-0.916501
Cs	2.299430	-2.173510	-0.839301
C	-4.031809	-2.894302	-2.059131
H	-4.127830	-2.412382	-3.043711
H	-3.037759	-3.355062	-1.967391
H	-4.806779	-3.669822	-1.964061
Ru	-0.675280	1.034069	0.555039
C	-0.766450	1.464489	2.743199
C	-0.941481	2.674989	1.983399
C	0.364500	0.629489	2.528199
H	-1.566000	1.136199	3.413929
C	0.006399	3.108449	1.002459
H	-1.873631	3.234219	2.108579
C	1.330430	1.047109	1.534289
C	1.152169	2.250149	0.800029
H	2.204940	0.395820	1.316209
H	1.870889	2.460280	0.004269
C	-0.234991	4.380549	0.194169
H	-0.163171	5.215589	0.921699
C	-1.646471	4.411059	-0.427561
H	-2.445901	4.335319	0.328689
H	-1.795841	5.359899	-0.970491
H	-1.760761	3.573109	-1.135521

C	0.828059	4.616379	-0.889671
H	0.625049	5.570589	-1.403581
H	1.848399	4.669080	-0.474521
H	0.806659	3.815229	-1.648381
C	0.541130	-0.672591	3.253749
H	-0.427380	-1.171921	3.422859
H	1.234980	-1.350591	2.721529
H	0.991450	-0.469971	4.244069
O	-0.847300	1.330409	-1.520101
C	0.128180	1.064579	-2.340641
O	1.236360	0.577849	-2.018961
C	-0.173350	1.426399	-3.790741
H	0.195120	0.629499	-4.456101
H	-1.246780	1.597889	-3.957431
H	0.376569	2.349509	-4.048181
O	4.466450	-0.239450	-2.039521
O	3.891070	-0.428410	1.351879
C	3.942860	-1.498770	2.060369
C	4.550260	0.846240	-1.466171
O	3.567830	1.782050	-1.538551
H	2.781230	1.340170	-1.982371
O	3.037460	-2.386330	2.122089
C	5.223870	-1.695240	2.890959
H	6.113730	-1.622200	2.241149
H	5.307620	-0.883060	3.635079
H	5.221370	-2.665250	3.411189
C	5.678250	1.255900	-0.560401
H	5.413310	0.832660	0.431799
H	6.619980	0.801781	-0.902011
H	5.774089	2.349310	-0.483291

Supporting Information: 010-Cs-Cluster-RuII-1HOAc-2OAc-Cymene-02-SP-TFE-PBE.log

----

Using Gaussian 16: ES64L-G16RevA.03 25-Dec-2016

==

# pbepbe/Def2QZVP/auto empiricaldispersion=gd3bj
scf=(maxcycle=300,direct,vshift=200,tight,yqc) density=current
scrf=(solvent=2,2,2-Trifluoroethanol,smd)

Pointgroup= C1 Stoichiometry= C20H37B10CsO8Ru C1[X(C20H37B10CsO8Ru)] #Atoms= 77
Charge = 0 Multiplicity = 1

SCF Energy= -1749.04649788

==

Optimization incomplete.

Atomic Type	Coordinates (Angstroms) X	Y	Z
B	-4.638560	1.362408	0.140890
B	-6.001460	0.270828	-0.111740
H	-7.140610	0.634148	-0.139510
H	-4.706980	2.546588	0.306450
B	-5.578380	-1.309252	0.593860
B	-5.450720	-1.056252	-1.166490
B	-3.361110	0.441948	0.966320
B	-3.244460	0.685988	-0.726920
H	-2.440250	0.845658	1.726710
H	-2.227920	1.243918	-1.259720
H	-6.116580	-1.718592	-1.904130
H	-6.328050	-2.137462	1.016140
C	-2.970580	-0.880952	-0.062360
C	-4.226660	-1.878492	-0.292800
B	-5.055760	0.186198	1.420150
B	-3.958250	-1.203652	1.299250
H	-3.517230	-1.938312	2.127530
H	-5.493660	0.495698	2.489300
B	-4.853240	0.600708	-1.461050
H	-5.147430	1.204738	-2.450150
B	-3.749530	-0.787982	-1.584910
H	-3.186950	-1.273292	-2.515790
C	-1.517040	-1.350952	-0.013660
O	-0.663920	-0.380661	0.124350
O	-1.226030	-2.554792	-0.097960
Cs	2.114780	-1.050641	-1.786850
C	-4.006520	-3.370542	-0.494240
H	-3.362000	-3.559292	-1.363600
H	-3.530590	-3.820642	0.387430
H	-4.988360	-3.839462	-0.659510
Ru	-1.389790	1.537898	0.327850
C	-0.313690	1.463239	2.621790
C	0.250730	2.504209	1.824620
C	0.352190	0.221279	2.797400
H	-1.201180	1.687308	3.225130
C	1.555990	2.351579	1.254930
H	-0.213270	3.497779	1.856250
C	1.588910	0.067199	2.166980
C	2.181250	1.113929	1.427520
H	2.130460	-0.883301	2.226310
H	3.146400	0.928139	0.945390
C	2.264360	3.473529	0.497970

H	2.230180	3.197299	-0.573920
C	3.756160	3.565029	0.887180
H	4.310300	2.656829	0.596610
H	4.221920	4.420599	0.367140
H	3.872000	3.722789	1.974300
C	1.573539	4.838819	0.632460
H	2.137929	5.600689	0.068380
H	0.546679	4.815859	0.230570
H	1.535339	5.169729	1.686880
C	-0.275010	-0.886551	3.606420
H	-1.361330	-0.959962	3.412070
H	0.183430	-1.858001	3.359290
H	-0.155310	-0.718551	4.693230
O	-0.277200	2.619629	-0.967870
C	0.037700	2.198739	-2.180640
O	-0.152320	1.048509	-2.608400
C	0.723060	3.272919	-3.017020
H	0.551920	4.281499	-2.613040
H	1.811730	3.082639	-3.024410
H	0.365370	3.210259	-4.056770
O	1.882320	-3.934041	-0.611360
O	4.631180	0.397109	-0.567010
C	4.903090	-0.569781	0.221920
C	1.961620	-3.912521	0.617860
O	0.947020	-3.460101	1.397840
H	0.216180	-3.164031	0.779460
O	4.067810	-1.464521	0.585130
C	6.317120	-0.639531	0.818930
H	7.021490	-0.008231	0.255180
H	6.285410	-0.280831	1.864030
H	6.676200	-1.682011	0.844820
C	3.180730	-4.298781	1.408220
H	3.695970	-5.133691	0.910090
H	3.847330	-3.410241	1.388800
H	2.934100	-4.556461	2.448880

Supporting Information: 010-Cs-Cluster-RuII-1HOAc-2OAc-Cymene-03-SP-TFE-PBE.log

----

Using Gaussian 16: ES64L-G16RevA.03 25-Dec-2016

==

# pbepbe/Def2QZVP/auto empiricaldispersion=gd3bj
scf=(maxcycle=300,direct,vshift=200,tight,yqc) density=current
scrf=(solvent=2,2,2-Trifluoroethanol,smd)

Pointgroup= C1 Stoichiometry= C20H37B10CsO8Ru C1[X(C20H37B10CsO8Ru)] #Atoms= 77
Charge = 0 Multiplicity = 1

SCF Energy= -1749.10036154

==

Optimization incomplete.

Atomic Type	Coordinates (Angstroms) X	Y	Z
B	-4.898930	0.583371	1.159570
B	-6.218990	-0.351619	0.413970
H	-7.371070	-0.041479	0.507400
H	-5.067780	1.586691	1.793520
B	-5.729250	-2.062009	0.393260
B	-5.596180	-1.085239	-1.082660
B	-3.605620	-0.553589	1.597880
B	-3.478860	0.397811	0.118080
H	-2.786430	-0.466719	2.458380
H	-2.742420	1.368980	-0.130230
H	-6.198930	-1.398319	-2.065960
H	-6.424450	-3.033919	0.404070
C	-3.096520	-1.242749	0.105390
C	-4.330750	-2.129399	-0.591450
B	-5.300200	-1.043809	1.784460
B	-4.119540	-2.186279	1.122310
H	-3.658511	-3.183969	1.580510
H	-5.766690	-1.237199	2.869290
B	-5.077890	0.552641	-0.624870
H	-5.376090	1.516331	-1.268860
B	-3.903720	-0.606819	-1.281310
H	-3.315650	-0.612859	-2.314560
C	-1.633430	-1.721500	-0.007870
O	-0.747230	-0.835470	0.329930
O	-1.382270	-2.889140	-0.326560
Cs	2.159030	-2.001991	-0.153040
C	-4.013911	-3.379199	-1.400510
H	-3.271131	-3.166029	-2.181480
H	-3.616241	-4.175209	-0.757980
H	-4.948421	-3.717329	-1.874460
Ru	-0.967380	1.244650	0.193510
C	-1.359079	2.903900	1.562800
C	-0.855789	3.460290	0.334080
C	-0.619939	1.950720	2.338000
H	-2.365109	3.185540	1.893410
C	0.373431	2.986170	-0.214110
H	-1.480329	4.153720	-0.232810

C	0.597310	1.469280	1.763080
C	1.084001	1.963310	0.513360
H	1.159430	0.633810	2.224480
H	1.998290	1.513310	0.114860
C	0.931681	3.470850	-1.536090
H	1.408111	2.590570	-2.003680
C	2.033691	4.512929	-1.239810
H	2.818071	4.097349	-0.584970
H	2.508091	4.833149	-2.183100
H	1.609511	5.406930	-0.748690
C	-0.128509	4.022270	-2.498840
H	0.335091	4.237760	-3.476200
H	-0.940129	3.291850	-2.652590
H	-0.564499	4.969240	-2.131450
C	-1.128320	1.430350	3.653130
H	-2.225000	1.510000	3.724640
H	-0.837180	0.377970	3.799340
H	-0.688549	2.016230	4.481430
O	-1.120450	0.821560	-1.845530
C	-0.120580	0.308970	-2.530290
O	1.033490	0.111350	-2.114180
C	-0.536160	-0.076160	-3.949660
H	-0.991480	-1.083020	-3.926840
H	-1.288220	0.619100	-4.353820
H	0.348290	-0.108840	-4.603230
O	5.216280	-1.876761	-0.535020
O	2.644470	-0.579391	2.752810
C	3.711590	-0.174451	2.217080
C	6.055010	-0.973351	-0.678740
O	5.947420	0.251869	-0.183430
H	5.035280	0.340529	0.374650
O	3.761270	0.436399	1.073200
C	5.050090	-0.465931	2.903140
H	5.707820	0.418569	2.873300
H	4.892470	-0.788201	3.942650
H	5.567020	-1.272861	2.352000
C	7.346190	-1.174422	-1.456090
H	7.383390	-2.189742	-1.875240
H	7.421140	-0.429582	-2.266330
H	8.212380	-1.014362	-0.791480

Supporting Information: 010-Cs-Cluster-RuII-1HOAc-2OAc-Cymene-04-SP-TFE-PBE.log

----

Using Gaussian 16: ES64L-G16RevA.03 25-Dec-2016

==

# pbepbe/Def2QZVP/auto empiricaldispersion=gd3bj
scf=(maxcycle=300,direct,vshift=200,tight,yqc) density=current

scrf=(solvent=2,2,2-Trifluoroethanol,smd)

Pointgroup= C1 Stoichiometry= C20H37B10CsO8Ru C1[X(C20H37B10CsO8Ru)] #Atoms= 77

Charge = 0 Multiplicity = 1

SCF Energy= -1749.06886067

==

Optimization incomplete.

Atomic Type	Coordinates (Angstroms) X	Y	Z
B	4.182291	-0.632542	0.143611
B	4.626434	-2.230451	0.799021
H	5.647115	-2.425509	1.392491
H	4.858620	0.351619	0.260851
B	3.929556	-3.470872	-0.269499
B	3.123666	-3.075634	1.260961
B	3.219742	-0.900593	-1.329309
B	2.417001	-0.536945	0.200101
H	3.092881	-0.197684	-2.287489
H	1.870639	0.498334	0.690781
H	2.973707	-3.910654	2.101191
H	4.328288	-4.579171	-0.467199
C	1.826013	-1.755776	-0.791749
C	2.227936	-3.261415	-0.188709
B	4.585264	-1.973381	-0.970299
B	3.064685	-2.654894	-1.576399
H	2.796096	-3.136734	-2.630529
H	5.557904	-1.978439	-1.667429
B	3.274003	-1.323803	1.528801
H	3.296182	-0.840953	2.625261
B	1.756554	-2.011216	0.912951
H	0.677534	-2.077328	1.400861
C	0.515343	-1.560368	-1.570539
O	-0.017579	-0.369639	-1.455729
O	0.050785	-2.455399	-2.272429
Cs	-3.100958	-1.271065	-0.974079
C	1.260538	-4.419677	-0.359829
H	0.277608	-4.175529	0.096671
H	1.116739	-4.648707	-1.424439
H	1.699440	-5.293826	0.146661
Ru	0.712448	1.254802	-0.375409
C	1.081276	2.273123	-2.285179

C	2.188076	2.523135	-1.433849
C	-0.271685	2.617120	-1.892949
H	1.241747	1.728133	-3.220859
C	1.999945	3.097454	-0.121049
H	3.187016	2.190036	-1.730059
C	-0.463435	3.144920	-0.580469
C	0.663424	3.391282	0.285011
H	-1.494596	3.182078	-0.132009
H	0.464973	3.760002	1.295891
C	3.215444	3.373107	0.748271
H	4.055745	2.796498	0.318691
C	3.069975	2.950766	2.217311
H	2.981817	1.854236	2.315881
H	3.971324	3.247538	2.779171
H	2.209464	3.433985	2.712781
C	3.550111	4.877337	0.627291
H	4.490631	5.097999	1.159461
H	3.671081	5.182807	-0.425839
H	2.749690	5.493796	1.072661
C	-1.444574	2.304808	-2.766839
H	-2.339604	2.100127	-2.134959
H	-1.670106	3.182658	-3.401799
H	-1.226112	1.450859	-3.429949
O	-0.757191	0.484639	0.980401
C	-0.839941	0.784209	2.201431
O	0.157558	1.400231	2.841021
C	-2.050711	0.503937	3.004901
H	-2.788532	1.264476	2.678561
H	-2.450029	-0.511234	2.764621
H	-1.842841	0.623597	4.077431
O	-1.426064	-3.288182	0.664811
O	-2.915515	2.888905	0.949121
C	-3.837964	2.242304	0.347831
C	-1.917335	-2.938953	1.789281
O	-2.941306	-2.194625	1.947881
O	-3.762223	1.692714	-0.799229
C	-5.163564	2.059181	1.115441
H	-5.152822	1.082211	1.636681
H	-5.295365	2.845651	1.875221
H	-6.020874	2.055050	0.422591
C	-1.199734	-3.408741	3.070751
H	-1.934843	-3.737073	3.824061
H	-0.482412	-4.219770	2.866561
H	-0.645155	-2.559540	3.514511
H	0.873477	1.563722	2.170841

Supporting Information: 010-Cs-Cluster-RuII-1HOAc-2OAc-Cymene-05-SP-TFE-PBE.log

----
Using Gaussian 16: ES64L-G16RevA.03 25-Dec-2016
==
# pbepbe/Def2QZVP/auto empiricaldispersion=gd3bj
scf=(maxcycle=300,direct,vshift=200,tight,yqc) density=current
scrf=(solvent=2,2,2-Trifluoroethanol,smd)

Pointgroup= C1 Stoichiometry= C20H37B10CsO8Ru C1[X(C20H37B10CsO8Ru)] #Atoms= 77
Charge = 0 Multiplicity = 1

SCF Energy= -1749.09413115
==
Optimization incomplete.

Atomic Type	Coordinates (Angstroms) X	Y	Z
B	4.517918	-1.261734	0.025080
B	5.845259	-0.125975	-0.199910
H	6.996229	-0.452436	-0.241160
H	4.616537	-2.445794	0.157660
B	5.378221	1.417546	0.552440
B	5.255450	1.211606	-1.215530
B	3.207959	-0.412132	0.880850
B	3.090759	-0.607742	-0.814210
H	2.309478	-0.874751	1.659240
H	2.085238	-1.196171	-1.340440
H	5.902621	1.914695	-1.933600
H	6.105431	2.256405	0.995340
C	2.784820	0.947178	-0.115580
C	4.016371	1.976127	-0.317700
B	4.899349	-0.113574	1.335210
B	3.759350	1.243997	1.257030
H	3.309611	1.954528	2.103060
H	5.354899	-0.437194	2.393660
B	4.698349	-0.451974	-1.553860
H	5.011458	-1.017324	-2.561000
B	3.559770	0.905117	-1.635820
H	2.987681	1.409688	-2.552310
C	1.320291	1.386489	-0.075530
O	0.496420	0.420340	0.113110
O	1.021512	2.594820	-0.245120
Cs	-1.906080	1.305273	-1.628490
C	3.773222	3.470257	-0.471610

H	3.172283	3.679678	-1.366930
H	3.240743	3.874758	0.399900
H	4.751813	3.965776	-0.563930
Ru	1.305058	-1.545161	0.264040
O	-0.046323	-2.526379	-0.941160
C	-0.381053	-2.087599	-2.124130
O	-0.114792	-0.966269	-2.608600
C	-1.157594	-3.117118	-2.949750
H	-0.528844	-3.427349	-3.802450
H	-1.424065	-4.008798	-2.363020
H	-2.068673	-2.656937	-3.369620
O	-0.411117	4.173521	1.364020
C	-1.661957	4.424572	0.958570
O	-2.180567	3.924183	-0.044290
C	-2.385206	5.365293	1.897930
H	-2.648766	4.817343	2.820060
H	-1.736345	6.205832	2.190610
H	-3.305265	5.734384	1.424350
C	-3.440773	-2.078246	-0.254420
C	-4.632832	-1.576625	-0.816030
C	-2.912402	-1.555657	0.935380
H	-2.903094	-2.890537	-0.754970
C	-5.275991	-0.516914	-0.144450
C	-3.567791	-0.509306	1.617980
H	-1.971183	-1.965647	1.316130
C	-4.754511	0.000325	1.051740
H	-6.208091	-0.105983	-0.552460
H	-5.282370	0.816156	1.561730
C	-5.215593	-2.176264	-2.076700
H	-5.863522	-1.458414	-2.609010
H	-4.425013	-2.509865	-2.771500
H	-5.834574	-3.063914	-1.847650
C	-3.001511	0.113744	2.889010
H	-3.854570	0.566024	3.433100
C	-2.326992	-0.904887	3.823390
H	-2.021371	-0.410097	4.761910
H	-3.015502	-1.729156	4.078230
H	-1.423512	-1.341568	3.364360
C	-2.026140	1.253803	2.525010
H	-1.155910	0.861472	1.969680
H	-2.525349	2.010103	1.893960
H	-1.651129	1.759192	3.432820
H	0.025013	3.523221	0.720570
O	0.206927	-2.456139	1.831480
C	0.858306	-3.564540	1.663210
O	1.805616	-3.562861	0.802190

C	0.483365	-4.796820	2.435850
H	0.166815	-4.526379	3.455500
H	-0.366656	-5.294039	1.935760
H	1.330284	-5.498101	2.469090

Supporting Information: 010-Cs-Cluster-RuII-1HOAc-2OAc-Cymene-06-SP-TFE-PBE.log

----

Using Gaussian 16: ES64L-G16RevA.03 25-Dec-2016

==

# pbepbe/Def2QZVP/auto empiricaldispersion=gd3bj
scf=(maxcycle=300,direct,vshift=200,tight,yqc) density=current
scrf=(solvent=2,2,2-Trifluoroethanol,smd)

Pointgroup= C1 Stoichiometry= C20H37B10CsO8Ru C1[X(C20H37B10CsO8Ru)] #Atoms= 77
Charge = 0 Multiplicity = 1

SCF Energy= -1749.09762560

==

Optimization incomplete.

Atomic Type	Coordinates (Angstroms) X	Y	Z
B	4.798721	0.410429	0.417350
B	5.886681	-0.944301	0.028140
H	7.038731	-0.967932	0.351160
H	5.135231	1.381469	1.033990
B	5.354251	-1.641491	-1.519900
B	4.920000	-2.437581	0.006190
B	3.604401	0.543829	-0.891850
B	3.175911	-0.268391	0.613050
H	3.005741	1.504799	-1.260180
H	2.433241	0.147849	1.518500
H	5.274440	-3.558581	0.220310
H	6.004720	-2.227781	-2.332970
C	2.749231	-0.949831	-0.867850
C	3.768610	-2.221761	-1.242830
B	5.281431	0.116549	-1.279980
B	3.947191	-0.724971	-2.086680
H	3.571951	-0.689211	-3.215970
H	5.975301	0.863359	-1.906470
B	4.568101	-1.180721	1.213750
H	4.741551	-1.357611	2.384560
B	3.242141	-2.026191	0.389110
H	2.435190	-2.794931	0.803480

C	1.282491	-0.979790	-1.351250
O	0.487691	-0.148800	-0.746250
O	0.952911	-1.712540	-2.288810
Cs	-2.540819	-0.399510	-1.646140
C	3.242210	-3.426761	-2.009560
H	2.335530	-3.830701	-1.538690
H	2.999300	-3.160051	-3.046130
H	4.027790	-4.198171	-1.999650
Ru	0.753971	0.713820	1.143870
C	-1.159099	1.681560	1.647860
C	-0.595999	2.382350	0.542720
C	-0.411019	1.498950	2.871260
H	-2.158039	1.256210	1.514000
C	0.732142	2.920600	0.585720
H	-1.193939	2.417790	-0.394350
C	0.915951	2.018940	2.926730
C	1.484702	2.680550	1.779210
H	1.544061	1.810570	3.797790
H	2.546092	2.952329	1.800250
C	1.314882	3.708490	-0.570530
H	2.414242	3.579779	-0.536410
C	0.998512	5.203470	-0.337080
H	1.387392	5.559800	0.632740
H	1.451792	5.813750	-1.137000
H	-0.092738	5.370460	-0.356940
C	0.796702	3.251370	-1.943900
H	1.326632	3.803890	-2.738620
H	0.970141	2.172900	-2.096230
H	-0.286468	3.437100	-2.053420
C	-0.996999	0.712770	4.008770
H	-0.212589	0.237740	4.619370
H	-1.581909	1.388970	4.659270
H	-1.678259	-0.062430	3.625910
O	0.483051	-1.272810	1.776900
C	-0.644279	-1.908600	1.692900
O	-1.742389	-1.394680	1.350150
C	-0.535470	-3.395420	2.008230
H	0.307980	-3.598040	2.684280
H	-1.476190	-3.773940	2.436180
H	-0.353610	-3.937340	1.062180
O	-3.929030	-2.990309	-0.629900
O	-3.785049	1.928211	-0.008950
C	-3.539748	2.752511	-0.943650
C	-4.333600	-3.394929	0.462490
O	-3.823910	-3.012199	1.642810
H	-3.046320	-2.365289	1.475260

O	-2.507368	2.729520	-1.709650
C	-4.537228	3.902991	-1.174040
H	-5.526888	3.664251	-0.753740
H	-4.159298	4.810441	-0.667370
H	-4.621658	4.142761	-2.246950
C	-5.475950	-4.375919	0.628690
H	-6.309070	-3.889649	1.164680
H	-5.821000	-4.719929	-0.355790
H	-5.156390	-5.235839	1.240620

Supporting Information: 010-Cs-Cluster-RuII-1HOAc-2OAc-PhSeBr-01-SP-TFE-PBE.log

----

Using Gaussian 16: ES64L-G16RevA.03 25-Dec-2016

==

# pbepbe/Def2QZVP/auto empiricaldispersion=gd3bj
scf=(maxcycle=300,direct,vshift=200,tight,yqc) density=current
scrf=(solvent=2,2,2-Trifluoroethanol,smd)

Pointgroup= C1 Stoichiometry= C16H28B10BrCsO8RuSe C1[X(C16H28B10BrCsO8RuSe)]
#Atoms= 66
Charge = 0 Multiplicity = 1

SCF Energy= -6566.52195861

==

Optimization incomplete.

Atomic Type	Coordinates (Angstroms) X	Y	Z
B	-2.494274	2.742264	0.069820
B	-2.220131	4.431744	0.556780
H	-3.099600	5.149876	0.937050
H	-3.575355	2.232966	0.085760
B	-0.879940	5.075472	-0.413830
B	-0.588021	4.541951	1.254060
B	-1.313615	2.353362	-1.192520
B	-1.031656	1.783272	0.480920
H	-1.392096	1.574752	-2.098620
H	-1.447568	0.682413	1.170300
H	-0.170280	5.309920	2.070040
H	-0.657788	6.207451	-0.727960
C	0.208305	2.529030	-0.454620
C	0.478288	4.120199	-0.005650
B	-2.045062	3.965544	-1.160050
B	-0.314392	3.796471	-1.497960

H	0.308818	3.964409	-2.499300
H	-2.785151	4.322685	-2.030740
B	-1.586924	3.102043	1.568640
H	-1.995364	2.862374	2.663560
B	0.135556	2.925090	1.209470
H	1.063826	2.606678	1.883870
C	1.348554	1.627478	-0.915160
O	1.204802	0.389258	-0.586080
O	2.309355	2.094506	-1.565900
Cs	3.399299	-1.239996	0.923790
C	1.886929	4.690747	-0.052870
H	2.345219	4.658236	0.947270
H	2.505688	4.120385	-0.760310
H	1.830381	5.739207	-0.382690
Ru	-0.331270	-0.417579	0.616420
O	0.923301	0.019778	2.249560
O	-1.339153	-1.866858	1.804810
C	0.341052	0.377939	3.386910
C	-0.452984	-2.757799	1.540740
O	-0.866578	0.564112	3.557100
O	0.542256	-2.411771	0.790650
C	-0.600177	-4.161089	2.047490
H	0.385012	-4.630361	2.194910
H	-1.179207	-4.173388	2.983050
H	-1.149258	-4.738528	1.281710
C	1.352482	0.600638	4.516160
H	1.890774	1.550117	4.342460
H	0.830052	0.664428	5.481550
H	2.103630	-0.207854	4.554780
Se	-1.563441	-0.965157	-1.338650
Br	-1.891885	-3.455617	-1.434900
C	-3.409420	-0.533104	-0.972070
C	-4.169369	-0.000183	-2.025580
C	-3.950810	-0.687023	0.314720
C	-5.486438	0.410670	-1.778820
H	-3.727959	0.109887	-3.021000
C	-5.273160	-0.288731	0.543480
H	-3.339251	-1.105844	1.118660
C	-6.036639	0.266141	-0.496830
H	-6.080408	0.846041	-2.588580
H	-5.703300	-0.398320	1.544030
H	-7.064818	0.590103	-0.306080
O	4.978090	-0.302659	-1.455630
C	4.938401	0.029741	-2.646150
O	4.027552	0.849293	-3.171220
C	5.930840	-0.453251	-3.684200

H	6.647229	-1.149392	-3.226980
H	5.399389	-0.949010	-4.513780
H	6.470532	0.406418	-4.116160
H	3.394443	1.189824	-2.450430

Supporting Information: 010-Cs-Cluster-RuII-1HOAc-2OAc-PhSeBr-02-SP-TFE-PBE.log

----

Using Gaussian 16: ES64L-G16RevA.03 25-Dec-2016

==

# pbepbe/Def2QZVP/auto empiricaldispersion=gd3bj
scf=(maxcycle=300,direct,vshift=200,tight,yqc) density=current
scrf=(solvent=2,2,2-Trifluoroethanol,smd)

Pointgroup= C1 Stoichiometry= C16H28B10BrCsO8RuSe C1[X(C16H28B10BrCsO8RuSe)]
#Atoms= 66
Charge = 0 Multiplicity = 1

SCF Energy= -6566.50529252

==

Optimization incomplete.

Atomic Type	Coordinates (Angstroms) X	Y	Z
B	1.110395	4.382981	0.283720
B	2.705245	4.832763	0.937440
H	2.938754	5.906923	1.411120
H	0.163085	5.114540	0.283710
B	3.942376	3.958924	0.005050
B	3.454946	3.366533	1.606080
B	1.362767	3.237891	-1.056150
B	0.900677	2.643181	0.547990
H	0.677227	3.028111	-2.007510
H	-0.192382	2.248230	0.986170
H	4.263597	3.268494	2.481240
H	5.079205	4.266345	-0.200480
C	2.136418	1.880612	-0.337970
C	3.640137	2.301154	0.281140
B	2.499745	4.587012	-0.823000
B	3.119437	2.979173	-1.221660
H	3.638957	2.564914	-2.209960
H	2.581134	5.468132	-1.628540
B	1.710416	3.618172	1.789130
H	1.204196	3.792031	2.859100
B	2.337418	2.009912	1.373270

H	2.354459	0.995432	1.999480
C	1.941749	0.493732	-0.970530
O	0.744150	0.035011	-0.969210
O	2.917370	-0.116007	-1.461450
Cs	1.528893	-2.734899	0.225680
C	4.788258	1.303735	0.286090
H	4.451889	0.302654	0.591300
H	5.259439	1.231395	-0.703180
H	5.535468	1.658825	1.012570
Ru	-0.909771	0.876149	0.055520
O	-0.178420	-0.328940	1.543620
O	-2.614942	1.947387	0.795330
C	-0.976930	-0.638071	2.567100
C	-2.681273	2.662347	-0.270640
O	-2.136770	-0.261142	2.729760
O	-1.791703	2.455058	-1.180730
C	-3.791884	3.653776	-0.465300
H	-3.531255	4.377276	-1.251520
H	-4.703123	3.104995	-0.765290
H	-4.007444	4.173066	0.481480
C	-0.306149	-1.601430	3.548050
H	0.791951	-1.495699	3.557510
H	-0.701689	-1.434241	4.560990
H	-0.573718	-2.633731	3.252520
Se	-2.145209	-0.725842	-1.084570
Br	-2.122537	-3.063052	0.163500
C	-4.004760	-0.347404	-0.768230
C	-4.823360	-0.202735	-1.901670
C	-4.507040	-0.171535	0.532430
C	-6.170130	0.148674	-1.730350
H	-4.411080	-0.352825	-2.904790
C	-5.858060	0.160974	0.685980
H	-3.836380	-0.273204	1.392350
C	-6.685751	0.327613	-0.437800
H	-6.814930	0.273003	-2.606160
H	-6.263491	0.302824	1.692840
H	-7.739511	0.594212	-0.304890
O	4.593202	-2.559075	-0.101210
C	5.482362	-2.215085	-0.885860
O	5.356121	-1.207505	-1.762940
C	6.843363	-2.869213	-0.967400
H	6.887054	-3.732533	-0.289950
H	7.053353	-3.186003	-2.002440
H	7.623762	-2.140202	-0.689470
H	4.447341	-0.792486	-1.645510

Using Gaussian 16: ES64L-G16RevA.03 25-Dec-2016
==
# pbepbe/Def2QZVP/auto empiricaldispersion=gd3bj
scf=(maxcycle=300,direct,vshift=200,tight,yqc) density=current
scrf=(solvent=2,2,2-Trifluoroethanol,smd)

Pointgroup= C1 Stoichiometry= C16H28B10BrCsO8RuSe C1[X(C16H28B10BrCsO8RuSe)]
#Atoms= 66
Charge = 0 Multiplicity = 1

SCF Energy= -6566.51588107
==
Optimization incomplete.

Atomic Type	Coordinates (Angstroms) X	Y	Z
B	-0.777790	4.302699	-0.784090
B	-0.001802	5.481610	0.305650
H	-0.469964	6.557209	0.544650
H	-1.818450	4.505747	-1.340210
B	1.759708	5.261884	0.183910
B	0.873530	4.574352	1.560360
B	0.505102	3.361671	-1.577660
B	-0.381187	2.665229	-0.199210
H	0.502953	2.817011	-2.636680
H	-1.455535	1.930217	-0.090840
H	1.141329	4.906913	2.677110
H	2.625207	6.065126	0.370200
C	1.324504	2.574103	-0.293350
C	2.061901	3.669884	0.726450
B	0.749679	5.095872	-1.270660
B	2.072001	3.961244	-0.977110
H	3.104391	3.794887	-1.546800
H	0.830447	5.875522	-2.175130
B	-0.694929	3.978669	0.974160
H	-1.658909	3.943867	1.683500
B	0.632973	2.848522	1.254160
H	0.774855	1.995502	2.076130
C	1.972556	1.213274	-0.539010
O	1.144128	0.252613	-0.749370
O	3.219947	1.105147	-0.552910
Cs	2.098504	-2.583276	-1.310240

C	3.373072	3.308127	1.408590
H	3.294294	2.357477	1.955740
H	4.185832	3.219159	0.675580
H	3.612561	4.107798	2.126270
Ru	-0.982232	0.445308	-0.803600
O	-0.799568	-1.360611	-1.767760
O	-3.045363	0.706614	-1.279960
C	-1.800207	-2.202903	-1.983850
C	-2.757823	1.168835	-2.445400
O	-2.979227	-2.077586	-1.646560
O	-1.513374	1.309907	-2.744460
C	-3.846824	1.488562	-3.428620
H	-3.495296	2.228373	-4.162960
H	-4.128912	0.562692	-3.960650
H	-4.738075	1.861151	-2.900650
C	-1.330954	-3.478402	-2.697550
H	-0.974973	-4.194992	-1.932980
H	-2.179873	-3.943444	-3.219750
H	-0.520355	-3.281541	-3.420450
Se	-0.906830	-0.387022	1.385990
C	-2.715820	-0.498545	2.028580
C	-2.935481	-0.092726	3.357500
C	-3.767969	-0.931647	1.204810
C	-4.240951	-0.099468	3.865490
H	-2.095482	0.228806	3.981830
C	-5.064139	-0.943930	1.733920
H	-3.567879	-1.250827	0.175280
C	-5.303000	-0.526420	3.053900
H	-4.425992	0.223201	4.895010
H	-5.894799	-1.281882	1.105980
H	-6.322210	-0.537543	3.454230
O	4.417063	-2.043141	0.578190
C	4.830641	-1.278380	1.452990
O	4.651819	0.050590	1.431420
C	5.592332	-1.726668	2.680990
H	5.788785	-2.805898	2.627420
H	6.541011	-1.171447	2.766620
H	5.002562	-1.502370	3.586340
H	4.129618	0.307099	0.603740
Br	-0.455865	-2.941191	1.375160

Supporting Information: 010-Cs-Cluster-RuII-1HOAc-2OAc-PhSeBr-Cymene-01-SP-TFE-PBE.log

----

Using Gaussian 16: ES64L-G16RevA.03 25-Dec-2016

==

# pbepbe/Def2QZVP/auto empiricaldispersion=gd3bj
scf=(maxcycle=300,direct,vshift=200,tight,yqc) density=current

scrf=(solvent=2,2,2-Trifluoroethanol,smd)

Pointgroup= C1 Stoichiometry= C26H42B10BrCsO8RuSe C1[X(C26H42B10BrCsO8RuSe)]
#Atoms= 90
Charge = 0 Multiplicity = 1

SCF Energy= -6955.67036488
==
Optimization incomplete.

Atomic Type	Coordinates (Angstroms) X	Y	Z
B	4.540951	2.054070	-0.192531
B	6.006581	1.355780	0.542100
H	6.799061	2.008730	1.156870
H	4.259381	3.216970	-0.116020
B	6.530621	-0.007450	-0.469761
B	5.634841	-0.318200	1.027130
B	4.176471	1.119210	-1.658881
B	3.274531	0.802610	-0.156560
H	3.643501	1.458710	-2.670451
H	2.114811	1.000140	0.403480
H	6.116981	-0.968430	1.905970
H	7.630751	-0.450200	-0.615471
C	3.860481	-0.465080	-1.100070
C	5.248171	-1.142350	-0.419341
B	5.864501	1.446770	-1.236590
B	5.397461	-0.162690	-1.822451
H	5.626341	-0.709240	-2.854841
H	6.534931	2.159680	-1.925201
B	4.403421	0.951980	1.211059
H	4.027321	1.308660	2.288050
B	3.951311	-0.656780	0.601430
H	3.291121	-1.512920	1.090240
C	2.857391	-1.310720	-1.907201
O	1.645181	-0.844350	-1.899491
O	3.230911	-2.304120	-2.537821
Cs	-3.641339	-1.933519	-0.327371
C	5.500371	-2.634040	-0.563551
H	4.893601	-3.034780	-1.389141
H	6.566801	-2.795730	-0.781841
H	5.241621	-3.153770	0.371020
Ru	0.940681	0.836390	-0.851361

C	0.758431	1.804700	-2.853701
C	1.280611	2.772290	-1.962011
C	-0.504739	1.142820	-2.596791
H	1.352671	1.496330	-3.719801
C	0.602211	3.094450	-0.729761
H	2.258091	3.219630	-2.161061
C	-1.181069	1.482150	-1.393190
C	-0.623309	2.424850	-0.463851
H	-2.159939	1.024141	-1.172310
H	-1.146509	2.570940	0.484950
C	1.152521	4.168870	0.190630
H	2.179811	4.397170	-0.148661
C	1.224571	3.746590	1.665470
H	1.981411	2.957690	1.806700
H	1.517211	4.609270	2.287759
H	0.254321	3.373660	2.038099
C	0.290341	5.438580	0.007980
H	0.719932	6.271670	0.589630
H	0.239511	5.747490	-1.050161
H	-0.740389	5.265730	0.363470
C	-1.019309	0.070690	-3.514501
H	-2.088709	-0.141369	-3.326901
H	-0.894959	0.377200	-4.567641
H	-0.430539	-0.853790	-3.366331
O	-5.750359	-0.216549	1.073030
O	-4.101319	1.056751	-1.305411
C	-4.765039	0.513641	-2.282260
C	-5.670349	0.950091	1.486990
O	-5.232249	1.980181	0.773000
O	-4.309539	-0.405239	-3.009311
C	-6.200739	1.009421	-2.473161
H	-6.797899	0.721431	-1.588630
H	-6.223229	2.110871	-2.532821
H	-6.652899	0.574841	-3.376690
C	-6.034119	1.339081	2.908270
H	-6.697659	0.582931	3.352180
H	-5.103309	1.388221	3.501980
H	-6.503139	2.334971	2.941409
Se	-0.072969	-0.870810	0.807840
Br	-1.856929	0.674420	2.118580
C	0.996811	-0.948520	2.440279
C	1.322791	-2.205080	2.972660
C	1.356341	0.229030	3.106219
C	2.045911	-2.271920	4.172309
H	1.043341	-3.116360	2.441069
C	2.078621	0.150230	4.303390

H	1.056371	1.196400	2.700619
C	2.430251	-1.098590	4.836089
H	2.308551	-3.250930	4.586030
H	2.368641	1.072100	4.817389
H	2.998571	-1.156920	5.769659
H	-4.841249	1.625521	-0.143251
O	1.058421	-2.510000	0.170370
C	0.347581	-3.238180	-0.685011
O	-0.815839	-2.986800	-1.034190
C	1.130461	-4.420520	-1.227110
H	1.779591	-4.046890	-2.039060
H	1.779511	-4.860190	-0.453411
H	0.437491	-5.174800	-1.627260

Supporting Information: 010-Cs-Cluster-RuII-1HOAc-2OAc-PhSeBr-Cymene-02-SP-TFE-PBE.log

----

Using Gaussian 16: ES64L-G16RevA.03 25-Dec-2016

==

# pbepbe/Def2QZVP/auto empiricaldispersion=gd3bj
scf=(maxcycle=300,direct,vshift=200,tight,yqc) density=current
scrf=(solvent=2,2,2-Trifluoroethanol,smd)

Pointgroup= C1 Stoichiometry= C26H42B10BrCsO8RuSe C1[X(C26H42B10BrCsO8RuSe)]
#Atoms= 90
Charge = 0 Multiplicity = 1

SCF Energy= -6955.66460105

==

Optimization incomplete.

Atomic Type	Coordinates (Angstroms) X	Y	Z
B	-4.433300	-0.736142	1.872180
B	-5.935720	-1.142443	1.004290
H	-7.023430	-0.874214	1.424790
H	-4.434981	-0.179142	2.934060
B	-5.652719	-2.641693	0.088280
B	-5.598040	-1.082483	-0.741720
B	-3.240760	-2.001182	1.493810
B	-3.166800	-0.427882	0.650730
H	-2.339919	-2.438291	2.135080
H	-2.398501	0.597919	0.369720
H	-6.341340	-0.836423	-1.644370
H	-6.440469	-3.475733	-0.245020

C	-2.989120	-1.870641	-0.185860
C	-4.404069	-2.277212	-1.028230
B	-4.936169	-2.448533	1.699070
B	-3.982629	-3.162802	0.377790
H	-3.573378	-4.271462	0.230830
H	-5.271479	-3.125513	2.626870
B	-4.839861	0.107308	0.350330
H	-5.108771	1.273857	0.292490
B	-3.906020	-0.638712	-0.960760
H	-3.474001	-0.141972	-1.945220
C	-1.558349	-2.208021	-0.658850
O	-0.631070	-1.561000	-0.006760
O	-1.346209	-3.067710	-1.518670
Cs	2.411730	-2.066338	-1.198210
C	-4.298659	-2.998392	-2.361290
H	-4.483329	-2.298472	-3.189780
H	-3.293239	-3.433502	-2.465240
H	-5.050229	-3.801543	-2.394830
Ru	-0.892561	0.228650	1.139390
C	-1.256891	-0.278970	3.355260
C	-1.645961	1.060609	3.030290
C	0.047990	-0.757830	3.049800
H	-2.007550	-0.964371	3.754060
C	-0.709352	2.029560	2.539350
H	-2.691342	1.351389	3.175530
C	0.959929	0.162311	2.416720
C	0.598928	1.534661	2.247420
H	1.948849	-0.210689	2.095400
H	1.327458	2.187071	1.757430
C	-1.123373	3.484630	2.369760
H	-2.033953	3.490349	1.739290
C	-0.058523	4.351960	1.685670
H	0.274377	3.931310	0.724690
H	-0.469384	5.356540	1.492070
H	0.830467	4.471321	2.330510
C	-1.495133	4.071849	3.749540
H	-1.834034	5.115429	3.634940
H	-2.303573	3.504209	4.241340
H	-0.618603	4.070700	4.421270
C	0.453951	-2.175179	3.296970
H	1.139111	-2.562299	2.513760
H	1.030491	-2.213269	4.241350
H	-0.419899	-2.837330	3.405460
O	5.241040	-0.926597	-0.854020
O	3.585610	-1.273348	1.833990
C	3.772691	-2.556458	1.834420

C	5.654309	0.063654	-0.230750
O	5.429949	0.297083	1.056470
O	2.861401	-3.400818	1.623960
C	5.214771	-3.030107	2.037340
H	5.799701	-2.763346	1.137970
H	5.678511	-2.510656	2.892830
H	5.255632	-4.118907	2.188650
C	6.448499	1.173234	-0.894520
H	7.205968	1.586784	-0.210450
H	6.917899	0.806114	-1.818740
H	5.746798	1.987924	-1.150520
Se	0.132359	1.038040	-1.068180
Br	2.538678	2.095242	-0.387330
C	-0.650372	2.779770	-1.453360
C	0.132697	3.730310	-2.126510
C	-1.990112	3.049689	-1.135040
C	-0.432624	4.967300	-2.465460
H	1.181417	3.514521	-2.346840
C	-2.545263	4.289079	-1.479740
H	-2.600182	2.301799	-0.623710
C	-1.766764	5.251409	-2.139960
H	0.179696	5.712540	-2.982810
H	-3.589183	4.500048	-1.226190
H	-2.200164	6.222319	-2.400100
H	4.732980	-0.408467	1.422090
O	-1.313881	0.280060	-2.399800
C	-0.716170	-0.478070	-3.317680
O	0.485770	-0.767209	-3.341330
C	-1.688850	-1.008821	-4.362920
H	-1.909760	-2.059951	-4.111050
H	-2.628440	-0.437531	-4.389150
H	-1.202500	-0.990700	-5.350380

Supporting Information: 010-Cs-Cluster-RuII-1HOAc-2OAc-PhSeBr-Cymene-03-SP-TFE-PBE.log

----

Using Gaussian 16: ES64L-G16RevA.03 25-Dec-2016

==

# pbepbe/Def2QZVP/auto empiricaldispersion=gd3bj
scf=(maxcycle=300,direct,vshift=200,tight,yqc) density=current
scrf=(solvent=2,2,2-Trifluoroethanol,smd)

Pointgroup= C1 Stoichiometry= C26H42B10BrCsO8RuSe C1[X(C26H42B10BrCsO8RuSe)]
#Atoms= 90
Charge = 0 Multiplicity = 1

SCF Energy= -6955.65514499

==

Optimization incomplete.

Atomic Type	Coordinates (Angstroms) X	Y	Z
B	-3.299344	3.200195	0.335291
B	-2.689291	4.639284	1.194481
H	-3.388030	5.330506	1.877401
H	-4.440895	2.834558	0.387391
B	-1.368110	5.338462	0.228091
B	-0.991692	4.334581	1.640301
B	-2.350744	3.033904	-1.159439
B	-1.967086	2.027913	0.256791
H	-2.664475	2.587144	-2.217709
H	-2.009069	0.796833	0.632341
H	-0.372351	4.782850	2.557741
H	-1.006058	6.475931	0.182801
C	-0.744185	2.777411	-0.632519
C	-0.151212	4.134729	0.158731
B	-2.787351	4.649564	-0.587509
B	-1.158322	4.344341	-1.219229
H	-0.641701	4.698270	-2.231429
H	-3.550110	5.327046	-1.213249
B	-2.179654	3.011143	1.718541
H	-2.507265	2.516234	2.757141
B	-0.547525	2.725050	1.072251
H	0.364884	2.095718	1.486981
C	0.185043	1.894359	-1.478949
O	-0.115769	0.622419	-1.479159
O	1.128084	2.384937	-2.104209
Cs	3.176969	-0.195947	-1.932609
C	1.322968	4.478217	0.045591
H	1.961297	3.600615	0.274261
H	1.559429	4.837396	-0.965779
H	1.535460	5.278246	0.772421
Ru	-1.922931	-0.256137	-0.772069
C	-3.002180	-0.024225	-2.751809
C	-3.934240	0.203907	-1.711679
C	-2.216173	-1.235457	-2.801509
H	-2.811189	0.762604	-3.487169
C	-4.130462	-0.753563	-0.648579
H	-4.453668	1.164738	-1.654869
C	-2.414444	-2.194546	-1.764119
C	-3.364284	-1.951834	-0.706589

H	-1.702486	-3.034788	-1.658299
H	-3.411245	-2.668264	0.117961
C	-5.146421	-0.489411	0.450441
H	-5.517199	0.542370	0.305351
C	-4.553161	-0.580612	1.866091
H	-3.826030	0.230336	2.036101
H	-5.355791	-0.480661	2.616501
H	-4.039823	-1.542143	2.039631
C	-6.335923	-1.457499	0.271071
H	-7.131122	-1.217047	0.996661
H	-6.764843	-1.391748	-0.743529
H	-6.021915	-2.502049	0.442981
C	-1.149793	-1.438529	-3.830989
H	-0.236944	-1.827040	-3.328649
H	-1.482564	-2.187728	-4.572779
H	-0.918851	-0.499269	-4.358439
O	4.004676	-1.982648	0.423281
O	-0.137707	-3.835871	-0.851089
C	0.972293	-3.540263	-1.416229
C	4.408787	-1.744449	1.574181
O	4.962979	-0.605760	1.959031
O	1.185475	-2.458383	-2.058349
C	2.147191	-4.498305	-1.224969
H	2.862862	-4.019846	-0.529489
H	1.817930	-5.461884	-0.807299
H	2.673571	-4.661896	-2.181179
C	4.241905	-2.738339	2.709411
H	4.111543	-3.753439	2.307591
H	5.095305	-2.699700	3.403961
H	3.334275	-2.468077	3.279631
Se	-0.241743	-1.544870	0.599091
Br	-1.463346	-3.150128	1.966121
C	0.198649	-0.436921	2.135281
C	1.555670	-0.092214	2.209521
C	-0.720380	0.001861	3.102051
C	2.002161	0.725465	3.259261
H	2.260519	-0.416405	1.442191
C	-0.263289	0.806210	4.150431
H	-1.771451	-0.292618	3.039341
C	1.094732	1.169927	4.228081
H	3.058892	1.001653	3.289201
H	-0.970558	1.159511	4.907681
H	1.439683	1.804606	5.050671
H	5.023290	0.098680	1.146601
O	3.159493	1.923663	0.524001
C	4.379884	2.109771	0.245421

O	5.282532	1.189729	0.206551
C	4.839827	3.528730	-0.117429
H	4.239207	3.917501	-0.959159
H	4.661458	4.201010	0.740291
H	5.907757	3.554048	-0.380029

Supporting Information: 020-Cs-Deprotonated-Cluster-RuII-2HOAc-1OAc-PhSeBr-01-SP-TFE-PBE.log

----

Using Gaussian 16: ES64L-G16RevA.03 25-Dec-2016

==

# pbepbe/Def2QZVP/auto empiricaldispersion=gd3bj
scf=(maxcycle=300,direct,vshift=200,tight,yqc) density=current
scrf=(solvent=2,2,2-Trifluoroethanol,smd)

Pointgroup= C1 Stoichiometry= C16H28B10BrCsO8RuSe C1[X(C16H28B10BrCsO8RuSe)] #Atoms= 66

Charge = 0 Multiplicity = 1

SCF Energy= -6566.51622542

==

Optimization incomplete.

Atomic Type	Coordinates (Angstroms) X	Y	Z
B	-2.121139	3.016180	-0.813360
B	-1.824849	4.749720	-0.532530
H	-2.706349	5.551770	-0.409180
H	-3.227429	2.570410	-0.918720
B	-0.310809	5.193950	-1.357120
B	-0.315709	4.904010	0.394070
B	-0.779249	2.404640	-1.797510
B	-0.821309	2.060750	-0.030020
H	-0.779380	1.499630	-2.583410
H	-1.991680	0.556710	3.012870
H	0.011321	5.767260	1.155820
H	0.016171	6.260790	-1.787710
C	0.606021	2.630310	-0.854910
C	0.908181	4.267360	-0.609800
B	-1.408379	4.027100	-2.115460
B	0.344021	3.742620	-2.141030
H	1.120891	3.743010	-3.045050
H	-1.987069	4.277010	-3.133610
B	-1.435529	3.556090	0.745670

H	-2.023479	3.517840	1.790720
B	0.307571	3.274160	0.681740
H	1.107491	3.043160	1.533810
C	1.739060	1.600890	-0.868810
O	1.472790	0.515170	-0.216020
O	2.810151	1.844540	-1.464080
Cs	2.355350	-2.513850	0.536960
C	2.344591	4.754130	-0.512560
H	2.623411	4.914780	0.539890
H	3.025121	4.015909	-0.962580
H	2.437031	5.707110	-1.055460
Ru	-0.451070	0.140890	0.737420
O	0.302640	1.111100	2.771960
O	-2.154370	-0.677650	1.953000
C	-0.480590	1.560210	3.634930
C	-1.568040	-1.772450	2.386580
O	-1.764360	1.245920	3.730020
O	-0.342410	-1.983590	2.180320
C	-2.443630	-2.788980	3.078910
H	-1.857160	-3.376050	3.801670
H	-3.302660	-2.310030	3.572300
H	-2.829310	-3.479120	2.306450
C	-0.023409	2.574830	4.648830
H	-0.180129	3.577990	4.211490
H	-0.607379	2.508620	5.578350
H	1.050791	2.451810	4.846470
Se	-1.088200	-0.825350	-1.227690
Br	-0.870920	-3.489190	-1.024790
C	-3.014580	-0.896460	-1.411930
C	-3.532450	-1.601230	-2.513620
C	-3.858170	-0.181030	-0.548890
C	-4.911970	-1.592919	-2.745380
H	-2.859340	-2.163980	-3.167830
C	-5.237920	-0.174199	-0.795280
H	-3.433000	0.360720	0.297110
C	-5.764890	-0.878199	-1.887750
H	-5.322470	-2.142739	-3.598410
H	-5.900960	0.387821	-0.129730
H	-6.843560	-0.869419	-2.075110
O	4.802950	-1.196041	-0.605470
C	5.587640	-0.379681	-1.101470
O	5.248870	0.832299	-1.535910
C	7.066780	-0.653181	-1.292810
H	7.328760	-1.631821	-0.867720
H	7.313140	-0.638401	-2.368240
H	7.665950	0.139429	-0.814590

H 4.252170 1.009449 -1.418660

Supporting Information: 020-Cs-Deprotonated-Cluster-RuII-2HOAc-1OAc-PhSeBr-02-SP-TFE-PBE.log

----

Using Gaussian 16: ES64L-G16RevA.03 25-Dec-2016

==

# pbepbe/Def2QZVP/auto empiricaldispersion=gd3bj
scf=(maxcycle=300,direct,vshift=200,tight,yqc) density=current
scrf=(solvent=2,2,2-Trifluoroethanol,smd)

Pointgroup= C1 Stoichiometry= C16H28B10BrCsO8RuSe C1[X(C16H28B10BrCsO8RuSe)]
#Atoms= 66

Charge = 0 Multiplicity = 1

SCF Energy= -6566.51883030

==

Optimization incomplete.

Atomic Type	Coordinates (Angstroms) X	Y	Z
B	-1.941864	2.628738	-1.665210
B	-1.945357	4.285038	-1.016700
H	-2.921488	4.980466	-0.993780
H	-2.919314	2.114156	-2.134770
B	-0.340018	4.997090	-1.312400
B	-0.747457	4.360920	0.295300
B	-0.334004	2.319790	-2.347820
B	-0.773203	1.639510	-0.744740
H	-0.021663	1.574711	-3.228360
H	0.284393	-2.325129	0.667630
H	-0.731958	5.086040	1.247560
H	-0.051870	6.151461	-1.439580
C	0.737016	2.476512	-1.032620
C	0.755743	4.029542	-0.436010
B	-1.061906	3.927899	-2.533060
B	0.656254	3.788782	-2.142020
H	1.633433	4.034203	-2.781090
H	-1.393217	4.341789	-3.607480
B	-1.741445	2.893688	0.093780
H	-2.556254	2.617237	0.923800
B	-0.028455	2.751051	0.460700
H	0.551106	2.400282	1.443810
C	1.961817	1.572154	-0.931590

O	1.669129	0.318373	-0.894470
O	3.129177	2.034576	-0.864260
Cs	3.571423	-1.970434	-0.312830
C	2.036553	4.623024	0.130190
H	2.343953	4.105244	1.050370
H	2.856623	4.554115	-0.597090
H	1.843971	5.681114	0.365640
Ru	-0.343960	-0.387330	-0.633260
O	-0.085080	-0.144159	1.483320
O	-0.318519	-1.040740	-2.651800
C	0.434411	-1.085119	2.121160
C	0.273673	-2.164229	-2.461680
O	0.684753	-2.291048	1.594610
O	0.684894	-2.516918	-1.295270
C	0.448584	-3.090999	-3.644090
H	-0.344574	-3.859220	-3.613110
H	0.364654	-2.537729	-4.590890
H	1.416555	-3.616237	-3.591310
C	0.874601	-0.929858	3.545630
H	0.578502	-1.806438	4.143500
H	1.977191	-0.851686	3.560900
H	0.442500	-0.010539	3.964660
Se	-2.562319	-1.009363	-0.628950
Br	-2.686565	-3.311323	0.560060
C	-3.582860	-0.061235	0.720180
C	-4.642381	0.725404	0.243120
C	-3.272480	-0.098934	2.086870
C	-5.375773	1.512522	1.142800
H	-4.875641	0.742933	-0.826080
C	-4.020131	0.677285	2.979400
H	-2.465319	-0.741843	2.442220
C	-5.064753	1.490253	2.508820
H	-6.190274	2.142581	0.771660
H	-3.785301	0.650575	4.048770
H	-5.639234	2.103212	3.210790
O	3.764850	0.012987	2.001260
C	3.910778	1.171707	2.411370
O	3.957406	2.250817	1.628860
C	4.026487	1.528477	3.877740
H	4.343679	0.650367	4.457900
H	4.724996	2.365708	4.029050
H	3.035977	1.855575	4.242200
H	3.739847	2.015427	0.659600

Supporting Information: 020-Cs-Deprotonated-Cluster-RuII-2HOAc-1OAc-PhSeBr-03-SP-TFE-PBE.log

----

Using Gaussian 16: ES64L-G16RevA.03 25-Dec-2016
==
# pbepbe/Def2QZVP/auto empiricaldispersion=gd3bj
scf=(maxcycle=300,direct,vshift=200,tight,yqc) density=current
scrf=(solvent=2,2,2-Trifluoroethanol,smd)

Pointgroup= C1 Stoichiometry= C16H28B10BrCsO8RuSe C1[X(C16H28B10BrCsO8RuSe)]
#Atoms= 66
Charge = 0 Multiplicity = 1

SCF Energy= -6566.51922579
==
Optimization incomplete.

Atomic Type	Coordinates (Angstroms) X	Y	Z
B	1.795566	3.443606	0.833529
B	2.060107	4.675636	-0.431561
H	3.005708	5.411375	-0.443251
H	2.543565	3.299955	1.760519
B	0.470278	5.237058	-0.999761
B	1.380246	4.049747	-1.951961
B	0.040565	3.258089	1.028549
B	0.976834	2.024158	0.106489
H	-0.585075	2.949700	1.994609
H	1.324352	1.009857	3.282339
H	1.713647	4.287817	-3.075821
H	0.179980	6.295019	-1.476161
C	-0.509435	2.702350	-0.475501
C	-0.290524	3.862619	-1.670431
B	0.707298	4.869108	0.719259
B	-0.781243	4.361730	-0.102591
H	-1.909703	4.722112	0.029109
H	0.660009	5.725668	1.554949
B	2.207505	2.940106	-0.828211
H	3.253834	2.458724	-1.153821
B	0.711154	2.444618	-1.619081
H	0.506313	1.642378	-2.481201
C	-1.672537	1.710161	-0.500151
O	-1.371819	0.530391	-0.058601
O	-2.798156	2.065753	-0.907681
Cs	-2.626733	-2.306547	0.072619
C	-1.297444	3.976221	-2.803391

H	-0.912454	3.482660	-3.708651
H	-2.250354	3.512032	-2.509311
H	-1.467122	5.041101	-3.024101
Ru	0.527381	0.082698	0.777669
O	2.355380	-0.430424	1.697479
O	-0.203958	0.785909	2.800449
C	2.873621	0.019925	2.747079
C	-0.724970	-0.357990	3.123979
O	2.292152	0.874256	3.574079
O	-0.677651	-1.323850	2.302899
C	-1.376530	-0.491969	4.480539
H	-1.787481	-1.501908	4.623119
H	-0.632840	-0.284490	5.269309
H	-2.176459	0.260302	4.586149
C	4.258170	-0.406937	3.147829
H	4.825991	0.459392	3.523069
H	4.197419	-1.145227	3.966859
H	4.768389	-0.863508	2.288229
Se	1.179809	-0.855363	-1.212321
C	3.100329	-1.179455	-1.282121
C	3.593287	-2.246416	-2.053571
C	3.985300	-0.265347	-0.685681
C	4.976057	-2.408278	-2.204391
H	2.898486	-2.958045	-2.507921
C	5.367340	-0.431099	-0.851691
H	3.594021	0.574164	-0.112311
C	5.866159	-1.504409	-1.603761
H	5.358206	-3.246889	-2.795601
H	6.053451	0.288860	-0.392911
H	6.946268	-1.633921	-1.727291
O	-5.154940	-0.730814	-0.346601
C	-5.855849	0.260797	-0.577141
O	-5.380507	1.481077	-0.824151
C	-7.370299	0.226520	-0.611841
H	-7.729790	-0.789820	-0.400771
H	-7.780668	0.931720	0.130419
H	-7.732389	0.554150	-1.600931
H	-4.365187	1.487825	-0.800041
Br	0.536866	-3.514432	-1.158461

Supporting Information: 020-Cs-Deprotonated-Cluster-RuII-2HOAc-1OAc-PhSeBr-04-SP-TFE-PBE.log

----

Using Gaussian 16: ES64L-G16RevA.03 25-Dec-2016

==

# pbepbe/Def2QZVP/auto empiricaldispersion=gd3bj
scf=(maxcycle=300,direct,vshift=200,tight,yqc) density=current

scrf=(solvent=2,2,2-Trifluoroethanol,smd)

Pointgroup= C1 Stoichiometry= C16H28B10BrCsO8RuSe C1[X(C16H28B10BrCsO8RuSe)]
#Atoms= 66
Charge = 0 Multiplicity = 1

SCF Energy= -6566.51963829
==
Optimization incomplete.

Atomic Type	Coordinates (Angstroms) X	Y	Z
B	1.238264	3.647031	1.358430
B	1.334822	5.056572	0.266950
H	2.162550	5.915963	0.376720
H	1.987494	3.490743	2.280880
B	-0.312929	5.454759	-0.272960
B	0.771302	4.542901	-1.341180
B	-0.474365	3.186139	1.475560
B	0.640936	2.237610	0.431790
H	-1.055615	2.658718	2.372780
H	1.161153	-2.099039	2.141250
H	1.078862	4.971571	-2.414920
H	-0.746721	6.513918	-0.620420
C	-0.922725	2.764208	-0.107670
C	-0.858287	4.086438	-1.136570
B	-0.045438	4.903559	1.392280
B	-1.435147	4.301977	0.468450
H	-2.605567	4.478955	0.608490
H	-0.226579	5.630629	2.326540
B	1.738684	3.426252	-0.341760
H	2.846495	3.139964	-0.693690
B	0.339105	2.829580	-1.233930
H	0.264696	2.118190	-2.192910
C	-1.927393	1.628776	-0.301310
O	-1.463511	0.454067	-0.012420
O	-3.087363	1.870105	-0.696030
Cs	-2.233616	-2.526324	-0.370860
C	-1.857107	4.204707	-2.276410
H	-1.402716	3.868477	-3.220850
H	-2.748736	3.597305	-2.062370
H	-2.153679	5.259446	-2.381220
Ru	0.462319	0.194910	0.840920

O	2.314800	-0.024637	1.844100
O	-0.502621	0.369439	2.841280
C	2.794901	-1.093056	2.297310
C	-0.767239	-0.859912	2.998960
O	2.142423	-2.236727	2.391400
O	-0.429668	-1.725231	2.087170
C	-1.473688	-1.337963	4.246520
H	-0.819347	-2.036882	4.796020
H	-1.738200	-0.491023	4.895060
H	-2.386297	-1.895864	3.974150
C	4.230401	-1.134634	2.739250
H	4.427643	-2.004774	3.380700
H	4.866191	-1.195453	1.837240
H	4.483700	-0.199214	3.260570
Se	1.297730	-0.424649	-1.199050
C	3.242710	-0.390975	-1.281830
C	3.891422	-1.209424	-2.224290
C	3.972549	0.526856	-0.508310
C	5.280191	-1.121802	-2.373280
H	3.312773	-1.928235	-2.811540
C	5.363449	0.610788	-0.671470
H	3.457958	1.166445	0.208820
C	6.019020	-0.213911	-1.596890
H	5.787452	-1.766341	-3.098460
H	5.931178	1.330639	-0.072610
H	7.105080	-0.147309	-1.718320
O	-4.969808	-1.313038	-0.642640
C	-5.820220	-0.417030	-0.691910
O	-5.544782	0.886281	-0.709690
C	-7.311729	-0.680292	-0.739110
H	-7.504998	-1.761602	-0.725450
H	-7.806520	-0.199743	0.121700
H	-7.745280	-0.233353	-1.649550
H	-4.541862	1.048552	-0.682470
Br	1.234385	-3.164899	-1.204370

Supporting Information: 020-Cs-Deprotonated-Cluster-RuII-2HOAc-1OAc-PhSeBr-05-SP-TFE-PBE.log

----

Using Gaussian 16: ES64L-G16RevA.03 25-Dec-2016

==

# pbepbe/Def2QZVP/auto empiricaldispersion=gd3bj
scf=(maxcycle=300,direct,vshift=200,tight,yqc) density=current
scrf=(solvent=2,2,2-Trifluoroethanol,smd)

Pointgroup= C1 Stoichiometry= C16H28B10BrCsO8RuSe C1[X(C16H28B10BrCsO8RuSe)] #Atoms= 66

Charge = 0 Multiplicity = 1

SCF Energy= -6566.51713538

===

Optimization incomplete.

Atomic Type	Coordinates (Angstroms) X	Y	Z
B	2.206347	3.142405	1.043850
B	2.788760	4.283784	-0.200780
H	3.845521	4.844731	-0.134530
H	2.840826	2.897744	2.032580
B	1.374632	5.103727	-0.907860
B	2.138679	3.757055	-1.769730
B	0.438717	3.272979	1.084070
B	1.220374	1.863177	0.254410
H	-0.322863	3.114471	1.988110
H	2.598189	3.909124	-2.863660
H	1.314324	6.186757	-1.412000
C	-0.073424	2.787511	-0.446290
C	0.440609	3.869389	-1.629800
B	1.399361	4.735047	0.827370
B	-0.081180	4.479431	-0.116460
H	-1.136298	5.033263	-0.081420
H	1.434803	5.602347	1.653600
B	2.663696	2.539744	-0.573070
H	3.629434	1.869512	-0.797900
B	1.171955	2.303608	-1.483730
H	0.903263	1.540358	-2.365460
C	-1.385806	2.006304	-0.516840
O	-1.330269	0.845884	0.054080
O	-2.396555	2.504716	-1.055910
Cs	-2.784775	-1.873823	0.589360
C	-0.439761	4.124382	-2.841990
H	-0.045192	3.590891	-3.720050
H	-1.466791	3.786444	-2.637540
H	-0.451318	5.203832	-3.056970
Ru	0.462429	-0.002161	0.853390
O	2.000196	-1.168414	1.827350
O	-0.045369	0.874601	2.810430
C	1.219644	-2.150143	2.211580
C	0.774322	1.110639	3.726610
O	-0.032506	-2.047919	2.073350

O	2.002391	0.619986	3.780790
C	0.428544	2.047129	4.848980
H	0.989134	1.806218	5.763760
H	0.705987	3.067849	4.527090
H	-0.655286	2.032082	5.031060
C	1.865981	-3.404924	2.736140
H	2.865361	-3.200397	3.147910
H	1.224030	-3.885343	3.490170
H	1.972849	-4.101224	1.883910
Se	0.800507	-0.915451	-1.220690
C	2.652756	-1.357456	-1.568640
C	2.937534	-2.030917	-2.770550
C	3.684097	-0.934879	-0.716480
C	4.268694	-2.287870	-3.115410
H	2.118883	-2.364645	-3.415790
C	5.014586	-1.191882	-1.075220
H	3.440448	-0.412518	0.209150
C	5.307945	-1.866893	-2.269010
H	4.496372	-2.815171	-4.047440
H	5.823777	-0.858274	-0.417550
H	6.349094	-2.064725	-2.543960
O	-4.956791	-0.157857	-0.584130
C	-5.518219	0.773154	-1.172650
O	-4.929236	1.915293	-1.520890
C	-6.976549	0.741928	-1.586080
H	-7.435781	-0.208431	-1.281360
H	-7.517867	1.585099	-1.124930
H	-7.060949	0.864848	-2.679060
H	-3.950346	1.931890	-1.239390
Br	0.075621	-3.502200	-1.146170
H	2.134859	-0.007005	2.990130

Supporting Information: 030-Selenylated-Cluster-RuII-2HOAc-1OAc-CsBr-01-SP-TFE-PBE.log

----

Using Gaussian 16: ES64L-G16RevA.03 25-Dec-2016

==

# pbepbe/Def2QZVP/auto empiricaldispersion=gd3bj
scf=(maxcycle=300,direct,vshift=200,tight,yqc) density=current
scrf=(solvent=2,2,2-Trifluoroethanol,smd)

Pointgroup= C1 Stoichiometry= C16H28B10BrCsO8RuSe C1[X(C16H28B10BrCsO8RuSe)]
#Atoms= 66
Charge = 0 Multiplicity = 1

SCF Energy= -6566.53951663

==

Optimization incomplete.

Atomic Type	Coordinates (Angstroms) X	Y	Z
B	-3.698672	1.796026	-1.721181
B	-3.423234	3.441626	-1.080301
H	-4.316895	4.181165	-0.785071
H	-4.787252	1.322615	-1.878721
B	-1.930765	4.064088	-1.813681
B	-1.896364	3.423598	-0.151211
B	-2.362762	1.414467	-2.838761
B	-2.350191	0.805607	-1.170661
H	-2.346861	0.651037	-3.756731
H	-1.579125	4.133218	0.757909
H	-1.623996	5.201368	-2.017331
C	-0.946602	1.500939	-1.871151
C	-0.688934	3.019589	-1.280781
B	-3.030434	3.066267	-2.788881
B	-1.281453	2.824369	-2.900001
H	-0.513574	3.035070	-3.783621
H	-3.622914	3.531136	-3.718821
B	-3.002612	2.037857	-0.080471
H	-3.569932	1.729986	0.925119
B	-1.256502	1.750139	-0.179291
H	-0.388472	1.657570	0.770839
C	0.316659	0.664581	-2.193691
O	0.906349	0.810381	-3.261941
O	0.675710	-0.159819	-1.234331
C	0.747096	3.493661	-1.128511
H	0.775745	4.584921	-1.269231
H	1.135556	3.257222	-0.125451
H	1.390966	3.028372	-1.891551
Ru	-0.171370	-0.062230	0.711389
Se	-2.091559	-1.083132	-0.513641
C	-3.623949	-1.157554	0.678469
C	-4.911709	-1.206616	0.124549
C	-3.419299	-1.233824	2.061659
C	-6.018079	-1.304187	0.979249
H	-5.051949	-1.152466	-0.959321
C	-4.535958	-1.340185	2.904359
H	-2.406769	-1.189763	2.470489
C	-5.831358	-1.371457	2.368109
H	-7.027318	-1.327658	0.555689
H	-4.387058	-1.394185	3.987799

H	-6.697898	-1.448728	3.032609
O	1.470389	0.611402	2.077499
C	0.656019	0.469621	3.087789
C	1.138789	0.717002	4.489289
H	1.771160	-0.130208	4.808599
H	1.754898	1.630802	4.525639
H	0.287049	0.805231	5.178699
O	-0.537850	0.111430	2.839099
Cs	3.562731	-0.845626	-0.131901
Br	0.549073	-2.455279	1.163049
H	0.702732	-1.799059	-1.962791
O	0.507103	-2.634059	-2.481541
C	1.649104	-3.324448	-2.630531
C	1.419715	-4.640948	-3.338471
H	0.879106	-5.325549	-2.661671
H	0.789045	-4.497709	-4.230571
H	2.382986	-5.089377	-3.616981
O	2.746253	-2.937897	-2.223451
O	4.304118	2.014435	0.795069
C	3.764526	3.026475	1.244649
O	2.595556	3.016723	1.911749
C	4.315935	4.426075	1.088999
H	4.276154	4.969385	2.046709
H	5.347165	4.383587	0.713319
H	3.691414	4.987205	0.371359
H	2.253738	2.060253	1.964609

Supporting Information: 030-Selenylated-Cluster-RuII-2HOAc-1OAc-CsBr-02-SP-TFE-PBE.log

----

Using Gaussian 16: ES64L-G16RevA.03 25-Dec-2016

==

# pbepbe/Def2QZVP/auto empiricaldispersion=gd3bj
scf=(maxcycle=300,direct,vshift=200,tight,yqc) density=current
scrf=(solvent=2,2,2-Trifluoroethanol,smd)

Pointgroup= C1 Stoichiometry= C16H28B10BrCsO8RuSe C1[X(C16H28B10BrCsO8RuSe)]
#Atoms= 66
Charge = 0 Multiplicity = 1

SCF Energy= -6566.54217707

==

Optimization incomplete.

Atomic Coordinates (Angstroms)
Type X Y Z

B	-3.567128	2.883992	-0.150831
B	-3.795508	3.170463	1.600749
H	-4.850698	3.475183	2.076819
H	-4.453678	2.958243	-0.952561
B	-2.277658	3.838182	2.242579
B	-2.658289	2.115322	2.486029
B	-1.906298	3.375972	-0.569111
B	-2.300369	1.667112	-0.298081
H	-1.471248	3.732231	-1.622501
H	-2.771109	1.680482	3.594909
H	-2.126228	4.550992	3.190349
C	-0.900439	2.366591	0.385239
C	-1.106918	2.616951	1.997169
B	-2.832048	4.322142	0.622909
B	-1.122418	3.975121	0.907319
H	-0.165647	4.682301	0.915479
H	-3.178577	5.446072	0.401819
B	-3.471439	1.525512	1.022929
H	-4.262999	0.630963	1.053389
B	-1.763879	1.133672	1.282379
H	-1.293040	0.125601	1.970159
C	0.527981	1.959290	-0.057941
O	1.339262	2.828840	-0.394511
O	0.758451	0.680990	0.001479
C	0.112611	2.463991	2.891549
H	-0.083258	2.959741	3.854079
H	0.322851	1.397151	3.075399
H	0.994342	2.932460	2.426579
Ru	-0.687380	-0.724879	0.619169
Se	-1.895700	0.058912	-1.444941
C	-3.686300	-0.696988	-1.454271
C	-4.665890	-0.084497	-2.249841
C	-3.958751	-1.861297	-0.725811
C	-5.952330	-0.638686	-2.295421
H	-4.432749	0.823643	-2.814511
C	-5.248731	-2.409937	-0.788251
H	-3.183981	-2.314328	-0.102001
C	-6.243921	-1.801596	-1.566721
H	-6.726470	-0.158556	-2.902651
H	-5.473961	-3.316627	-0.217171
H	-7.249431	-2.232746	-1.605701
O	0.284109	-1.741429	2.324319
C	-0.717291	-2.557389	2.389479
C	-0.700032	-3.721599	3.343709
H	-0.239472	-4.591519	2.842059

H	-0.105591	-3.480979	4.238599
H	-1.726772	-3.996118	3.628949
O	-1.708731	-2.368268	1.602779
Cs	3.124680	-1.059531	1.030589
Br	0.377729	-2.488510	-0.932381
H	1.129610	-0.899030	-2.329191
O	1.276480	-0.066960	-2.886741
C	2.497280	0.409539	-2.672961
C	2.709691	1.781969	-3.251071
H	2.092101	1.936170	-4.147921
H	2.385341	2.501249	-2.474811
H	3.772881	1.955249	-3.471451
O	3.357440	-0.210741	-2.015641
O	5.708780	0.433848	0.316429
C	6.242301	0.997688	-0.643721
O	5.736111	1.009058	-1.883421
C	7.546391	1.761407	-0.554951
H	7.370832	2.825777	-0.787301
H	7.968351	1.668367	0.454859
H	8.262801	1.381547	-1.302201
H	4.842450	0.517428	-1.887021

Supporting Information: 030-Selenylated-Cluster-RuII-2HOAc-1OAc-CsBr-03-SP-TFE-PBE.log

---

Using Gaussian 16: ES64L-G16RevA.03 25-Dec-2016

==

# pbepbe/Def2QZVP/auto empiricaldispersion=gd3bj
scf=(maxcycle=300,direct,vshift=200,tight,yqc) density=current
scrf=(solvent=2,2,2-Trifluoroethanol,smd)

Pointgroup= C1 Stoichiometry= C16H28B10BrCsO8RuSe C1[X(C16H28B10BrCsO8RuSe)]
#Atoms= 66
Charge = 0 Multiplicity = 1

SCF Energy= -6566.53366562

==

Optimization incomplete.

Atomic Type	Coordinates (Angstroms) X	Y	Z
B	-4.298534	1.843901	0.120519
B	-4.146315	2.362252	1.825929
H	-5.078015	2.410599	2.576009
H	-5.331943	1.493459	-0.373021

B	-2.843968	3.571224	1.909869
B	-2.523814	1.889335	2.403429
B	-3.083246	2.736914	-0.829611
B	-2.774052	1.069945	-0.306881
H	-3.091956	3.009464	-1.992131
H	-2.183203	1.653956	3.525969
H	-2.709249	4.457285	2.701239
C	-1.575755	2.278467	-0.148451
C	-1.430576	2.748388	1.420219
B	-3.935117	3.554742	0.504829
B	-2.201938	3.809136	0.276369
H	-1.585810	4.766397	-0.068981
H	-4.696459	4.456570	0.306379
B	-3.432851	0.819393	1.318169
H	-3.825349	-0.254588	1.665179
B	-1.692072	1.028597	1.070699
H	-0.711920	0.387939	1.643679
C	-0.257815	2.253690	-0.967591
O	0.101423	3.278041	-1.561241
O	0.384148	1.126722	-0.921521
C	-0.047497	3.144641	1.909439
H	-0.139188	3.575800	2.917939
H	0.609805	2.259952	1.960159
H	0.403632	3.895362	1.242589
Ru	-0.261609	-0.509720	0.261309
Se	-2.159368	-0.513104	-1.391811
C	-3.510896	-1.774137	-0.792601
C	-4.818836	-1.643600	-1.282231
C	-3.151853	-2.822366	0.063629
C	-5.791914	-2.570462	-0.884331
H	-5.079528	-0.819361	-1.953401
C	-4.134531	-3.747808	0.446519
H	-2.129523	-2.897054	0.443269
C	-5.450452	-3.623021	-0.021831
H	-6.818434	-2.467264	-1.250671
H	-3.865360	-4.567278	1.120999
H	-6.212750	-4.346023	0.285899
O	1.404942	-0.841646	1.667039
C	0.777824	-1.862628	2.153169
C	1.403176	-2.724176	3.216709
H	1.835508	-3.616685	2.731569
H	2.201585	-2.175714	3.739199
H	0.636557	-3.059178	3.932539
O	-0.383795	-2.136160	1.687269
Cs	3.376360	0.332998	-0.642911
Br	0.833175	-2.157857	-1.386551

H	2.967380	4.696057	1.724819
O	2.715709	5.158937	0.890999
C	3.188581	4.368678	-0.111491
C	2.839290	4.893677	-1.473181
H	1.896731	4.398355	-1.781701
H	2.680487	5.981587	-1.463491
H	3.630660	4.623009	-2.187991
O	3.791053	3.322629	0.126329
O	4.437136	-2.472519	0.063369
C	4.040338	-3.527670	0.558099
O	2.791239	-4.007143	0.425599
C	4.896530	-4.437708	1.413809
H	4.784972	-5.487209	1.097519
H	5.948719	-4.128316	1.351429
H	4.562120	-4.376209	2.464699
H	2.239918	-3.369434	-0.135001

Supporting Information: 030-Selenylated-Cluster-RuII-2HOAc-1OAc-CsBr-04-SP-TFE-PBE.log

----

Using Gaussian 16: ES64L-G16RevA.03 25-Dec-2016

==

# pbepbe/Def2QZVP/auto empiricaldispersion=gd3bj
scf=(maxcycle=300,direct,vshift=200,tight,yqc) density=current
scrf=(solvent=2,2,2-Trifluoroethanol,smd)

Pointgroup= C1 Stoichiometry= C16H28B10BrCsO8RuSe C1[X(C16H28B10BrCsO8RuSe)]
#Atoms= 66
Charge = 0 Multiplicity = 1

SCF Energy= -6566.53712072

==

Optimization incomplete.

Atomic Type	Coordinates (Angstroms) X	Y	Z
B	-2.743191	3.272490	0.351230
B	-1.906261	3.542980	1.909750
H	-2.437391	4.056550	2.851590
H	-3.888521	3.561820	0.156400
B	-0.191221	3.842270	1.555910
B	-0.707051	2.232910	2.124810
B	-1.529981	3.401280	-0.949630
B	-2.040471	1.814260	-0.342810
H	-1.687791	3.691560	-2.097610

H	1.653549	-2.626310	1.190980
H	-0.271571	1.798670	3.150740
H	0.595909	4.473960	2.197240
C	-0.388351	2.165800	-0.606180
C	0.380639	2.407470	0.830240
B	-1.434901	4.488250	0.461200
B	0.037009	3.769460	-0.197680
H	0.957999	4.252250	-0.775620
H	-1.611241	5.667740	0.359480
B	-2.288821	1.889600	1.405890
H	-3.090561	1.186070	1.942340
B	-0.840571	1.119060	0.724500
H	-0.332491	-0.032800	1.156560
C	0.454249	1.467800	-1.701830
O	0.262469	0.187860	-1.797280
O	1.281479	2.120650	-2.355480
Cs	3.267419	-0.467940	-1.828100
C	1.830979	1.978240	0.947160
H	1.909899	0.880630	1.009490
H	2.416449	2.342120	0.088590
H	2.261399	2.401870	1.866750
Ru	-0.709321	-0.899070	-0.244270
O	3.353879	-1.009740	1.381840
O	1.070629	-2.293910	-0.269880
C	2.959639	-1.760250	2.304330
C	0.362549	-3.076380	-1.041340
O	2.052789	-2.700170	2.131920
O	-0.718451	-2.622600	-1.527460
C	0.804668	-4.489740	-1.300580
H	0.369658	-4.864180	-2.239100
H	1.903808	-4.569130	-1.322190
H	0.434588	-5.118870	-0.471010
C	3.450909	-1.647020	3.726230
H	2.731669	-1.029500	4.293950
H	3.486469	-2.635910	4.207120
H	4.435389	-1.158790	3.762530
Se	-2.712411	0.165340	-1.291390
C	-4.430131	0.058720	-0.379550
C	-5.427351	0.955931	-0.793250
C	-4.681171	-0.917789	0.590990
C	-6.696521	0.884941	-0.203170
H	-5.213661	1.709991	-1.557520
C	-5.958851	-0.979779	1.167950
H	-3.880881	-1.593710	0.909980
C	-6.963311	-0.083349	0.776470
H	-7.475841	1.589441	-0.511490

H	-6.161151	-1.733799	1.935660
H	-7.955831	-0.136329	1.235630
Br	-1.566931	-2.446500	1.595020
O	5.225479	1.229680	-0.185420
C	5.431129	1.638170	0.960620
O	4.912609	1.066060	2.059520
C	6.267929	2.854100	1.289140
H	6.958899	2.639229	2.120030
H	6.824589	3.177729	0.399290
H	5.606179	3.673940	1.619800
H	4.345409	0.270850	1.774040

Supporting Information: 030-Selenylated-Cluster-RuII-2HOAc-1OAc-CsBr-05-SP-TFE-PBE.log

---

Using Gaussian 16: ES64L-G16RevA.03 25-Dec-2016

==

# pbepbe/Def2QZVP/auto empiricaldispersion=gd3bj
scf=(maxcycle=300,direct,vshift=200,tight,yqc) density=current
scrf=(solvent=2,2,2-Trifluoroethanol,smd)

Pointgroup= C1 Stoichiometry= C16H28B10BrCsO8RuSe C1[X(C16H28B10BrCsO8RuSe)]
#Atoms= 66
Charge = 0 Multiplicity = 1

SCF Energy= -6566.54023515

==

Optimization incomplete.

Atomic Type	Coordinates (Angstroms) X	Y	Z
B	-3.791929	2.809230	-0.700071
B	-3.414879	3.757990	0.766529
H	-4.254169	4.318090	1.410729
H	-4.904090	2.662551	-1.119561
B	-1.806139	4.476939	0.544049
B	-1.989929	3.018720	1.551289
B	-2.403439	2.943080	-1.810611
B	-2.603610	1.511480	-0.780961
H	-2.382599	2.830800	-2.999261
H	1.833340	0.048268	2.513129
H	-1.689019	3.050169	2.708789
H	-1.370729	5.479799	1.028029
C	-1.081520	2.281659	-0.938721
C	-0.731359	3.156659	0.413889

B	-2.905179	4.355330	-0.845931
B	-1.186949	3.983719	-1.041761
H	-0.324099	4.549569	-1.633691
H	-3.361919	5.335740	-1.358191
B	-3.230240	1.993140	0.802589
H	-3.915970	1.252890	1.442939
B	-1.520040	1.577479	0.595179
H	-0.745440	0.868249	1.357219
C	0.134400	1.600529	-1.626671
O	0.352460	0.377149	-1.237001
O	0.822120	2.233939	-2.431591
Cs	2.891079	-1.285782	-1.189281
C	0.725961	3.267029	0.831829
H	1.007950	2.434728	1.495699
H	1.379951	3.266158	-0.054691
H	0.868551	4.215249	1.372169
Ru	-0.694541	-0.529771	0.348469
O	3.921050	0.296827	1.417539
O	0.848049	-0.990691	1.891669
C	3.519730	1.009738	2.357419
C	-0.073171	-1.554511	2.629669
O	2.349990	0.827888	2.954209
O	-1.265271	-1.540631	2.196669
C	0.299769	-2.207941	3.930969
H	-0.599001	-2.399691	4.534179
H	0.801938	-3.168791	3.720669
H	1.006779	-1.574572	4.492209
C	4.289620	2.187857	2.905499
H	3.849211	3.116867	2.500659
H	4.198890	2.233737	4.001549
H	5.346150	2.141337	2.606539
Se	-2.555751	-0.433360	-1.308601
C	-4.162591	-1.035060	-0.398211
C	-5.411221	-0.505309	-0.755131
C	-4.047581	-2.057250	0.553119
C	-6.563391	-0.988589	-0.119991
H	-5.484540	0.288171	-1.504501
C	-5.210231	-2.537169	1.173739
H	-3.063211	-2.458520	0.809419
C	-6.464341	-2.002999	0.844009
H	-7.539911	-0.568069	-0.381111
H	-5.129232	-3.331469	1.922969
H	-7.366801	-2.377629	1.337729
Br	-0.302191	-2.869201	-0.598771
O	5.868299	-0.418883	-1.248151
C	6.678940	0.283126	-0.640821

O	6.423660	0.852317	0.550019
C	8.068710	0.607846	-1.141231
H	8.822430	0.290086	-0.401361
H	8.248030	0.103996	-2.100401
H	8.177900	1.698546	-1.266171
H	5.480750	0.605437	0.826959

Supporting Information: 030-Selenylated-Cluster-RuII-2HOAc-1OAc-CsBr-06-SP-TFE-PBE.log

----

Using Gaussian 16: ES64L-G16RevA.03 25-Dec-2016

==

# pbepbe/Def2QZVP/auto empiricaldispersion=gd3bj
scf=(maxcycle=300,direct,vshift=200,tight,yqc) density=current
scrf=(solvent=2,2,2-Trifluoroethanol,smd)

Pointgroup= C1 Stoichiometry= C16H28B10BrCsO8RuSe C1[X(C16H28B10BrCsO8RuSe)]
#Atoms= 66
Charge = 0 Multiplicity = 1

SCF Energy= -6566.54640158

==

Optimization incomplete.

Atomic Type	Coordinates (Angstroms) X	Y	Z
B	-2.819270	3.404860	-0.699079
B	-2.366040	4.185560	0.843061
H	-3.138210	4.828430	1.494051
H	-3.919170	3.477869	-1.167839
B	-0.654420	4.652070	0.727251
B	-1.111770	3.183010	1.624011
B	-1.383190	3.387960	-1.749649
B	-1.842700	1.939190	-0.825639
H	-1.326540	3.348420	-2.941669
H	-1.266029	-3.284660	1.165371
H	-0.864690	3.093670	2.790531
H	-0.091950	5.541920	1.293471
C	-0.218830	2.476580	-0.883019
C	0.202890	3.185950	0.542651
B	-1.698880	4.797180	-0.704349
B	-0.055430	4.172380	-0.869229
H	0.910560	4.631260	-1.390349
H	-1.977720	5.867750	-1.160799
B	-2.459460	2.415430	0.761951

H	-3.280310	1.755099	1.325561
B	-0.824560	1.748030	0.590701
H	-0.262430	0.885200	1.409151
C	0.907440	1.672020	-1.582789
O	1.018210	0.443080	-1.168509
O	1.625360	2.213100	-2.427259
Cs	3.287661	-1.479330	-1.419739
C	1.635590	3.011690	1.015131
H	1.785290	2.004760	1.433041
H	2.339790	3.161070	0.181561
H	1.849420	3.755160	1.797331
Ru	-0.257759	-0.380280	0.276171
O	0.210381	-2.092710	-0.916699
O	-1.464279	-3.229930	2.199761
C	-0.394759	-3.222760	-1.026929
C	-1.750569	-2.005520	2.560101
O	-1.277999	-3.692770	-0.255619
O	-1.578969	-0.974070	1.864931
C	-2.322289	-1.862130	3.948251
H	-2.889379	-0.923550	4.023571
H	-2.955259	-2.725130	4.204121
H	-1.486199	-1.822430	4.669381
C	0.026021	-4.082880	-2.216459
H	0.651831	-4.921170	-1.861049
H	-0.869089	-4.525180	-2.681759
H	0.579561	-3.500580	-2.969849
Se	-2.057190	0.032780	-1.464129
C	-3.793649	-0.422571	-0.719349
C	-4.879910	0.459709	-0.806069
C	-3.946829	-1.705521	-0.172939
C	-6.126690	0.062739	-0.302229
H	-4.757410	1.452849	-1.244529
C	-5.200529	-2.091711	0.321161
H	-3.099219	-2.396470	-0.133409
C	-6.289139	-1.209051	0.265371
H	-6.972310	0.756119	-0.355679
H	-5.319909	-3.090471	0.754151
H	-7.264379	-1.513291	0.658821
Br	1.729291	-0.985870	1.861671
O	5.113740	0.282441	0.270321
C	5.104070	1.107061	1.185851
O	4.168170	1.155461	2.148541
H	3.474720	0.440590	1.977881
C	6.133480	2.203461	1.351801
H	6.484150	2.252861	2.395161
H	6.977650	2.030741	0.670631

H 5.670470 3.178001 1.116951

Supporting Information: 030-Selenylated-Cluster-RuII-2HOAc-1OAc-CsBr-07-SP-TFE-PBE.log

----

Using Gaussian 16: ES64L-G16RevA.03 25-Dec-2016

==

# pbepbe/Def2QZVP/auto empiricaldispersion=gd3bj
scf=(maxcycle=300,direct,vshift=200,tight,yqc) density=current
scrf=(solvent=2,2,2-Trifluoroethanol,smd)

Pointgroup= C1 Stoichiometry= C16H28B10BrCsO8RuSe C1[X(C16H28B10BrCsO8RuSe)]
#Atoms= 66
Charge = 0 Multiplicity = 1

SCF Energy= -6566.53787117

==

Optimization incomplete.

Atomic Type	X	Y	Z
B	5.037880	-0.322040	-0.620870
B	5.385210	-1.310900	0.832060
H	6.375690	-1.173450	1.489260
H	5.759330	0.547460	-1.016860
B	4.669790	-2.921561	0.578480
B	3.828060	-1.732281	1.600640
B	4.114320	-1.337951	-1.757570
B	3.281410	-0.172311	-0.709170
H	4.027160	-1.249981	-2.944860
H	3.617450	-1.975291	2.752620
H	5.019750	-3.963690	1.046870
C	2.689000	-1.760801	-0.908420
C	2.984640	-2.654501	0.440400
B	5.423450	-2.063570	-0.790830
B	3.907770	-2.941271	-1.019050
H	3.650220	-3.929421	-1.629250
H	6.425750	-2.475680	-1.297470
B	4.065410	-0.130541	0.875170
H	4.066280	0.864979	1.536030
B	2.522900	-0.973431	0.648240
H	1.569150	-0.731231	1.541700
C	1.354270	-2.060661	-1.629490
O	1.304700	-2.848181	-2.571460
O	0.339120	-1.421421	-1.112360

C	1.952330	-3.703111	0.824510
H	2.350900	-4.308681	1.652250
H	1.014150	-3.223031	1.146810
H	1.738840	-4.365621	-0.028470
Ru	0.482420	-0.048631	0.435570
O	-0.202970	1.569529	1.744150
C	-1.192140	1.809459	0.921450
O	-1.200880	1.131049	-0.167470
C	-2.286560	2.753979	1.276340
H	-1.935780	3.516189	1.987580
H	-2.715130	3.213639	0.373430
H	-3.090210	2.143569	1.742700
Se	1.918610	1.256989	-1.161220
C	2.845630	2.742859	-0.313850
C	3.892620	3.344969	-1.027470
C	2.453370	3.214769	0.944990
C	4.572589	4.429099	-0.455660
H	4.185040	2.963749	-2.010870
C	3.136759	4.306979	1.500500
H	1.635400	2.727719	1.482800
C	4.194559	4.911349	0.806170
H	5.399329	4.895870	-1.000460
H	2.840839	4.680259	2.486300
H	4.726759	5.759299	1.248580
Br	-4.405370	-0.242411	1.588320
Cs	-2.785170	-1.414051	-1.365180
O	-0.827170	-1.429371	1.464190
C	-1.233950	-1.338511	2.654980
O	-0.972670	-0.319011	3.449770
C	-2.079760	-2.412571	3.261590
H	-3.130350	-2.116601	3.063150
H	-1.879330	-3.381461	2.782100
H	-1.924190	-2.466831	4.349190
H	-0.582000	0.441619	2.906290
O	-4.687220	0.818899	-2.194650
C	-5.141180	1.857259	-1.706270
O	-5.285140	2.090149	-0.396920
C	-5.622590	3.030309	-2.538760
H	-5.326990	2.891609	-3.587900
H	-6.722610	3.098479	-2.476120
H	-5.216630	3.977379	-2.147600
H	-5.006380	1.269369	0.148540

Supporting Information: 030-Selenylated-Cluster-RuII-2HOAc-1OAc-CsBr-08-SP-TFE-PBE.log

----

Using Gaussian 16: ES64L-G16RevA.03 25-Dec-2016

==

# pbepbe/Def2QZVP/auto empiricaldispersion=gd3bj
scf=(maxcycle=300,direct,vshift=200,tight,yqc) density=current
scrf=(solvent=2,2,2-Trifluoroethanol,smd)

Pointgroup= C1 Stoichiometry= C16H28B10BrCsO8RuSe C1[X(C16H28B10BrCsO8RuSe)]
#Atoms= 66
Charge = 0 Multiplicity = 1

SCF Energy= -6566.54042745
==
Optimization incomplete.

Atomic Type	Coordinates (Angstroms) X	Y	Z
B	4.505179	-1.048103	-0.846520
B	4.983108	-1.781364	0.714140
H	6.089818	-1.692375	1.161480
H	5.250959	-0.407784	-1.530590
B	4.028287	-3.265333	0.930160
B	3.554368	-1.776513	1.786510
B	3.254448	-2.087533	-1.575090
B	2.803939	-0.612552	-0.696800
H	2.960568	-2.215192	-2.725440
H	3.533358	-1.757923	2.982600
H	4.313166	-4.243653	1.555490
C	1.968298	-2.082152	-0.439280
C	2.400847	-2.749392	1.000190
B	4.613467	-2.828624	-0.692110
B	2.976047	-3.458822	-0.481190
H	2.479406	-4.487452	-0.813900
H	5.436227	-3.495514	-1.249300
B	3.865719	-0.404403	0.705490
H	4.132560	0.683527	1.121160
B	2.205929	-0.988152	0.906670
H	1.354139	-0.647431	1.834340
C	0.488028	-2.279200	-0.853660
O	0.159697	-3.277260	-1.506650
O	-0.315461	-1.351970	-0.424450
C	1.335697	-3.517821	1.765180
H	1.824246	-4.162901	2.510700
H	0.658177	-2.820770	2.286280
H	0.750556	-4.151061	1.079850
Ru	0.322510	0.244740	0.802100

Se	1.599750	0.902989	-1.261550
C	2.808381	2.359038	-0.817260
C	3.889972	2.614557	-1.673080
C	2.555922	3.161618	0.301970
C	4.750133	3.682946	-1.385220
H	4.068781	1.977937	-2.545240
C	3.420233	4.233367	0.572510
H	1.714592	2.936059	0.962340
C	4.515133	4.493306	-0.264290
H	5.604833	3.880446	-2.040240
H	3.235403	4.863208	1.448910
H	5.188064	5.328006	-0.043150
O	-0.891310	-0.003529	2.630610
C	-0.402350	1.102940	3.088100
C	-0.913919	1.696141	4.373190
H	-1.788589	2.333281	4.150600
H	-1.231830	0.902531	5.067020
H	-0.140179	2.324750	4.838800
O	0.503341	1.695550	2.404910
Cs	-3.127432	-1.463067	0.893880
Br	-1.550349	1.740041	-0.135960
H	-1.562950	0.746041	-2.135500
O	-1.368500	0.149911	-2.930120
C	-2.235301	-0.861448	-2.944000
C	-1.824392	-1.982399	-3.857740
H	-1.230412	-1.607649	-4.704700
H	-1.188072	-2.669669	-3.266610
H	-2.711022	-2.528598	-4.210110
O	-3.264691	-0.900677	-2.242950
O	-5.326740	0.507244	0.145910
C	-5.305229	1.443184	-0.658040
O	-4.650709	1.413564	-1.827690
H	-4.139730	0.545803	-1.895460
C	-5.987498	2.772735	-0.425430
H	-6.736778	2.676255	0.372450
H	-5.225508	3.510384	-0.116710
H	-6.451968	3.146645	-1.351340

Supporting Information: 030-Selenylated-Cluster-RuII-2HOAc-1OAc-CsBr-09-SP-TFE-PBE.log

----

Using Gaussian 16: ES64L-G16RevA.03 25-Dec-2016

==

# pbepbe/Def2QZVP/auto empiricaldispersion=gd3bj
scf=(maxcycle=300,direct,vshift=200,tight,yqc) density=current
scrf=(solvent=2,2,2-Trifluoroethanol,smd)

Pointgroup= C1 Stoichiometry= C16H28B10BrCsO8RuSe C1[X(C16H28B10BrCsO8RuSe)]
#Atoms= 66
Charge = 0 Multiplicity = 1

SCF Energy= -6566.52851419

===

Optimization incomplete.

Atomic Type	Coordinates (Angstroms) X	Y	Z
B	-3.942641	0.666307	-0.778050
B	-5.309381	-0.309644	-1.337130
H	-6.319531	0.171065	-1.761770
H	-3.935962	1.860717	-0.791460
B	-5.345380	-1.802494	-0.368120
B	-4.673070	-1.827794	-2.014840
B	-3.113041	-0.221393	0.529020
B	-2.471471	-0.251072	-1.108720
H	-2.777051	0.301018	1.667640
H	0.146197	2.777619	1.715850
H	-5.169529	-2.531964	-2.842620
H	-6.287829	-2.489275	-0.108140
C	-2.612560	-1.704552	-0.190350
C	-3.875959	-2.610043	-0.716850
B	-4.897031	-0.264404	0.409300
B	-4.010470	-1.743843	0.793570
H	-3.951269	-2.389543	1.794120
H	-5.596071	0.243456	1.237530
B	-3.787081	-0.304483	-2.284570
H	-3.678771	0.184957	-3.370610
B	-2.907220	-1.785053	-1.893070
H	-2.147019	-2.442352	-2.532200
C	-1.341989	-2.390832	0.359720
O	-0.677470	-1.653581	1.196430
O	-1.018159	-3.523981	-0.021910
Cs	2.202991	-2.311969	0.154890
C	-3.800568	-4.128743	-0.656760
H	-3.017538	-4.509763	-1.324920
H	-3.576858	-4.471183	0.363480
H	-4.779248	-4.528234	-0.963720
Ru	-1.081511	0.389039	1.449730
O	1.036569	0.583720	1.651460
O	-1.360271	0.691348	3.624030

C	1.693948	1.658400	1.552460
C	-1.432982	1.949248	3.456480
O	1.146277	2.860280	1.561790
O	-1.367432	2.405618	2.231210
C	-1.590143	2.908928	4.601400
H	-0.721273	3.588869	4.637520
H	-2.486803	3.531978	4.445220
H	-1.672782	2.362998	5.551580
C	3.168668	1.634831	1.334920
H	3.317538	1.488971	0.239020
H	3.634347	2.580622	1.645280
H	3.628749	0.774412	1.842320
Se	-0.584511	0.436279	-1.014520
C	-0.713342	2.262489	-1.649300
C	0.064967	2.588799	-2.769350
C	-1.466183	3.226878	-0.965460
C	0.053077	3.912259	-3.233240
H	0.706678	1.834160	-3.235280
C	-1.469664	4.543988	-1.445460
H	-2.023123	2.954708	-0.065610
C	-0.717524	4.884999	-2.580080
H	0.661616	4.181300	-4.102310
H	-2.057444	5.305918	-0.923250
H	-0.720055	5.916619	-2.946600
Br	2.992399	0.356261	-2.066800
O	5.025810	-1.316757	0.773950
C	5.949659	-0.623737	0.330040
O	5.858989	0.195063	-0.717840
C	7.343009	-0.615626	0.930030
H	7.363400	-1.228596	1.841650
H	8.066010	-1.015456	0.198450
H	7.654779	0.416934	1.158760
H	4.915209	0.169982	-1.124370

Supporting Information: 030-Selenylated-Cluster-RuII-2HOAc-1OAc-CsBr-10-SP-TFE-PBE.log

----

Using Gaussian 16: ES64L-G16RevA.03 25-Dec-2016

==

# pbepbe/Def2QZVP/auto empiricaldispersion=gd3bj
scf=(maxcycle=300,direct,vshift=200,tight,yqc) density=current
scrf=(solvent=2,2,2-Trifluoroethanol,smd)

Pointgroup= C1 Stoichiometry= C16H28B10BrCsO8RuSe C1[X(C16H28B10BrCsO8RuSe)]
#Atoms= 66
Charge = 0 Multiplicity = 1

SCF Energy= -6566.53332462

==

Optimization incomplete.

Atomic Type	Coordinates (Angstroms) X	Y	Z
B	-2.589271	2.003091	1.537751
B	-4.124331	2.875601	1.595621
H	-4.334901	3.762811	2.370561
H	-1.677031	2.248891	2.266751
B	-5.411221	1.738531	1.131411
B	-4.798581	2.923611	-0.048589
B	-2.903631	0.323711	1.023861
B	-2.316271	1.505211	-0.138279
H	-2.346232	-0.658199	1.666731
H	-1.640302	-2.317919	-2.091389
H	-5.547781	3.728731	-0.515289
H	-6.564151	1.765151	1.443491
C	-3.639931	0.476631	-0.531249
C	-5.061671	1.289701	-0.480919
B	-4.048381	1.158661	2.118301
B	-4.670331	0.163021	0.799931
H	-5.255682	-0.875499	0.799731
H	-4.200171	0.809591	3.253091
B	-3.038541	3.089641	0.182741
H	-2.458231	4.106721	-0.062359
B	-3.668161	2.096031	-1.132379
H	-3.617221	2.221831	-2.315229
C	-3.471742	-0.657599	-1.559529
O	-2.601192	-1.551749	-1.151609
O	-4.100312	-0.681319	-2.610929
Cs	2.914278	-1.222370	0.924851
C	-6.190541	0.916371	-1.431169
H	-5.915321	1.127011	-2.472499
H	-6.436221	-0.151919	-1.351759
H	-7.074111	1.510381	-1.151689
Ru	-1.287202	-1.255679	0.459241
O	0.205048	-2.385490	-0.554989
O	-1.639712	-3.014799	1.696021
C	0.188548	-2.831610	-1.739719
C	-0.647542	-2.706029	2.440881
O	-0.841822	-2.780849	-2.547309
O	-0.003232	-1.610950	2.188011
C	-0.218402	-3.603790	3.572931

H	0.410718	-4.422930	3.180311
H	0.357958	-3.037850	4.320601
H	-1.101392	-4.062529	4.044181
C	1.432288	-3.425110	-2.328349
H	1.973168	-4.016050	-1.572369
H	1.197738	-4.044590	-3.205169
H	2.074068	-2.570300	-2.635239
Se	-0.567291	0.785781	-0.858949
C	0.652129	1.845030	0.223831
C	1.453539	2.779820	-0.446579
C	0.787789	1.628340	1.603351
C	2.380779	3.535540	0.286251
H	1.380299	2.888000	-1.531799
C	1.719109	2.390580	2.327381
H	0.177389	0.866390	2.097611
C	2.512589	3.344160	1.669871
H	3.016939	4.255370	-0.237069
H	1.818929	2.239150	3.407861
H	3.239639	3.934120	2.237731
Br	2.669959	0.005660	-2.357559
O	5.101439	0.845740	0.587791
C	5.355999	1.835930	-0.105899
O	4.837439	2.086370	-1.309029
H	4.173019	1.352460	-1.579159
C	6.284769	2.944710	0.351601
H	6.988269	2.557359	1.102531
H	6.827189	3.389669	-0.496439
H	5.680459	3.742510	0.820241

Supporting Information: 030-Selenylated-Cluster-RuII-2HOAc-1OAc-CsBr-11-SP-TFE-PBE.log

----

Using Gaussian 16: ES64L-G16RevA.03 25-Dec-2016

==

# pbepbe/Def2QZVP/auto empiricaldispersion=gd3bj
scf=(maxcycle=300,direct,vshift=200,tight,yqc) density=current
scrf=(solvent=2,2,2-Trifluoroethanol,smd)

Pointgroup= C1 Stoichiometry= C16H28B10BrCsO8RuSe C1[X(C16H28B10BrCsO8RuSe)]
#Atoms= 66
Charge = 0 Multiplicity = 1

SCF Energy= -6566.50554790

==

Optimization incomplete.

Atomic Type	Coordinates (Angstroms) X	Y	Z
B	3.995850	-0.299271	-2.156270
B	5.331630	-1.150491	-1.354600
H	6.478160	-1.016382	-1.671920
H	4.136970	0.473019	-3.059770
B	4.715340	-2.720891	-0.799710
B	4.881540	-1.377951	0.350950
B	2.550720	-1.342431	-2.091260
B	2.740440	-0.007631	-0.941270
H	1.621350	-1.364590	-2.836770
H	-3.419509	0.880641	-2.015580
H	5.596150	-1.485762	1.303200
H	5.318019	-3.738701	-0.626940
C	2.206730	-1.556111	-0.422620
C	3.492510	-2.367861	0.332590
B	4.156580	-2.064151	-2.350060
B	3.006450	-2.843671	-1.252270
H	2.412719	-3.870271	-1.359350
H	4.432130	-2.591911	-3.388370
B	4.435880	0.122779	-0.473550
H	4.898551	1.180069	-0.157890
B	3.270530	-0.677981	0.611290
H	2.853330	-0.351181	1.676470
C	0.779100	-1.917070	0.051730
O	-0.224290	-1.159700	-0.269710
O	0.616050	-2.971640	0.687120
Cs	-3.281160	-1.708159	0.728870
C	3.259409	-3.312261	1.502140
H	2.727400	-2.803361	2.316880
H	2.675639	-4.188621	1.195890
H	4.248539	-3.630491	1.867560
Ru	-0.640589	0.641640	-1.089730
O	-4.935929	0.727191	-0.258110
O	-2.451810	-0.545019	-2.084080
C	-4.514919	1.779871	-0.737800
C	-1.669750	-0.693470	-3.108470
O	-3.701319	1.825431	-1.816610
O	-0.495810	-0.188910	-3.069010
C	-2.137670	-1.443789	-4.327100
H	-1.353840	-1.458110	-5.097610
H	-2.400950	-2.479189	-4.049760
H	-3.049370	-0.970709	-4.730980
C	-4.796589	3.152761	-0.181770
H	-3.931929	3.432021	0.447810

H	-4.889519	3.902031	-0.982920
H	-5.701579	3.130361	0.441120
Se	1.538181	1.599410	-1.086270
C	2.181581	2.631519	0.450790
C	2.939401	3.769509	0.135750
C	1.910141	2.293769	1.782900
C	3.444792	4.571169	1.169760
H	3.139731	4.021289	-0.910800
C	2.417871	3.102209	2.809260
H	1.314791	1.413070	2.019720
C	3.183521	4.237999	2.506340
H	4.042212	5.455999	0.927200
H	2.208021	2.840269	3.851500
H	3.575362	4.865609	3.313300
Br	-1.479269	1.595560	1.135840
O	-1.693030	-1.750110	3.330990
C	-0.481650	-1.579090	3.465180
O	0.179200	-0.506770	2.988130
H	-0.467710	0.076360	2.477720
C	0.437960	-2.566720	4.143660
H	-0.100330	-3.083370	4.951600
H	0.731499	-3.314340	3.385990
H	1.346260	-2.081640	4.531550

Supporting Information: 030-Selenylated-Cluster-RuII-2HOAc-1OAc-CsBr-12-SP-TFE-PBE.log

----

Using Gaussian 16: ES64L-G16RevA.03 25-Dec-2016

==

\# pbepbe/Def2QZVP/auto empiricaldispersion=gd3bj
scf=(maxcycle=300,direct,vshift=200,tight,yqc) density=current
scrf=(solvent=2,2,2-Trifluoroethanol,smd)

Pointgroup= C1 Stoichiometry= C16H28B10BrCsO8RuSe C1[X(C16H28B10BrCsO8RuSe)]
#Atoms= 66
Charge = 0 Multiplicity = 1

SCF Energy= -6566.53712478

==

Optimization incomplete.

Atomic Type	Coordinates (Angstroms) X	Y	Z
B	-2.743582	3.272299	0.351421
B	-1.906632	3.542889	1.909911

H	-2.437782	4.056339	2.851801
H	-3.888952	3.561518	0.156651
B	-0.191642	3.842390	1.556001
B	-0.707251	2.232960	2.124871
B	-1.530432	3.401269	-0.949479
B	-2.040701	1.814179	-0.342659
H	-1.688382	3.691579	-2.097439
H	1.653951	-2.626459	1.190651
H	-0.271671	1.798740	3.150781
H	0.595438	4.474140	2.197331
C	-0.388661	2.165940	-0.606149
C	0.380369	2.407660	0.830291
B	-1.435442	4.488219	0.461361
B	0.036538	3.769640	-0.197579
H	0.957428	4.252530	-0.775599
H	-1.611973	5.667689	0.359661
B	-2.288991	1.889459	1.406041
H	-3.090651	1.185848	1.942521
B	-0.840671	1.119099	0.724591
H	-0.332490	-0.032730	1.156611
C	0.453989	1.468080	-1.701849
O	0.262520	0.188080	-1.797129
O	1.280949	2.121071	-2.355679
Cs	3.267490	-0.467758	-1.828019
C	1.830759	1.978621	0.947111
H	1.909799	0.881041	1.009871
H	2.416039	2.342201	0.088281
H	2.261339	2.402671	1.866441
Ru	-0.709250	-0.899000	-0.244259
O	3.353580	-1.009308	1.381691
O	1.070801	-2.293740	-0.269979
C	2.960311	-1.760719	2.303881
C	0.362801	-3.076140	-1.041569
O	2.053661	-2.700809	2.131351
O	-0.718219	-2.622390	-1.527649
C	0.805022	-4.489460	-1.300979
H	0.370102	-4.863780	-2.239589
H	1.904172	-4.568789	-1.322519
H	0.434912	-5.118720	-0.471529
C	3.452621	-1.648488	3.725491
H	2.733440	-1.031979	4.294381
H	3.489211	-2.637808	4.205421
H	4.436820	-1.159658	3.761461
Se	-2.712490	0.165219	-1.291259
C	-4.430190	0.058408	-0.379389
C	-5.427511	0.955477	-0.793119

C	-4.681100	-0.918092	0.591211
C	-6.696671	0.884367	-0.203029
H	-5.213921	1.709527	-1.557429
C	-5.958760	-0.980193	1.168181
H	-3.880729	-1.593912	0.910201
C	-6.963330	-0.083894	0.776671
H	-7.476071	1.588766	-0.511369
H	-6.160969	-1.734193	1.935931
H	-7.955850	-0.136974	1.235841
Br	-1.566679	-2.446741	1.594851
O	5.225199	1.230282	-0.185309
C	5.430739	1.638703	0.960771
O	4.912099	1.066532	2.059601
C	6.267408	2.854683	1.289411
H	6.825298	3.177463	0.400011
H	5.605348	3.674963	1.618361
H	6.957198	2.640433	2.121431
H	4.344970	0.271322	1.774021

Supporting Information: 030-Selenylated-Cluster-RuII-2HOAc-1OAc-CsBr-13-SP-TFE-PBE.log

----

Using Gaussian 16: ES64L-G16RevA.03 25-Dec-2016

==

# pbepbe/Def2QZVP/auto empiricaldispersion=gd3bj
scf=(maxcycle=300,direct,vshift=200,tight,yqc) density=current
scrf=(solvent=2,2,2-Trifluoroethanol,smd)

Pointgroup= C1 Stoichiometry= C16H28B10BrCsO8RuSe C1[X(C16H28B10BrCsO8RuSe)]
#Atoms= 66
Charge = 0 Multiplicity = 1

SCF Energy= -6566.53661593

==

Optimization incomplete.

Atomic Type	Coordinates (Angstroms) X	Y	Z
B	3.058181	-2.185389	2.043969
B	2.773281	-1.212839	3.518889
H	3.626521	-0.996578	4.329899
H	4.120251	-2.658018	1.758779
B	1.088011	-1.472549	4.017549
B	1.528960	0.014611	3.140039
B	1.542851	-3.033109	1.649299

B	1.984471	-1.533869	0.807289
H	1.382412	-4.044979	1.035849
H	-1.339651	3.292059	0.172519
H	1.367490	1.080491	3.657819
H	0.619361	-1.395260	5.114809
C	0.401971	-1.776030	1.382279
C	0.136011	-0.870850	2.731319
B	2.021241	-2.831749	3.352469
B	0.335921	-2.598140	2.877429
H	-0.644068	-3.232440	3.109069
H	2.316032	-3.767719	4.037469
B	2.759240	-0.414969	1.937989
H	3.582430	0.363312	1.560829
B	1.085250	-0.152949	1.410429
H	0.570030	1.044550	1.096659
C	-0.786649	-1.943910	0.415159
O	-0.915679	-1.022630	-0.475231
O	-1.554929	-2.918651	0.579129
Cs	-3.790860	0.216938	0.003829
C	-1.261530	-0.327800	2.971329
H	-1.440860	0.594699	2.394979
H	-2.016409	-1.089501	2.714829
H	-1.371620	-0.090331	4.040069
Ru	0.404290	0.673260	-0.535971
O	-2.826281	2.492569	1.835859
O	-1.368911	1.973979	-0.913861
C	-2.194661	3.557739	1.872099
C	-1.080211	1.908570	-2.185541
O	-1.396392	3.993699	0.895749
O	-0.202240	1.069190	-2.559791
C	-1.742691	2.837379	-3.163611
H	-1.734431	2.402769	-4.174251
H	-2.771931	3.079929	-2.852851
H	-1.163902	3.778260	-3.184021
C	-2.217452	4.502319	3.053319
H	-1.299382	4.349310	3.648259
H	-2.218192	5.551589	2.719699
H	-3.090592	4.294169	3.687209
Se	2.083711	-1.093509	-1.156521
C	3.984180	-0.664718	-1.193631
C	4.873661	-1.750278	-1.156401
C	4.439980	0.651162	-1.328991
C	6.251391	-1.506007	-1.233611
H	4.497751	-2.773538	-1.056661
C	5.822480	0.878303	-1.410361
H	3.726899	1.481592	-1.335861

C	6.726460	-0.192127	-1.361571
H	6.951831	-2.346457	-1.192971
H	6.188919	1.905673	-1.505361
H	7.803410	-0.004416	-1.421441
Br	1.695859	2.859851	-0.659911
O	-4.573189	-2.425462	-1.202411
C	-4.135738	-3.462942	-1.714541
O	-2.965488	-4.022261	-1.404851
C	-4.877648	-4.250092	-2.774871
H	-5.828198	-3.755222	-3.016271
H	-4.257038	-4.337982	-3.682431
H	-5.070128	-5.275352	-2.416331
H	-2.499098	-3.484161	-0.677491

Supporting Information: 030-Selenylated-Cluster-RuII-2HOAc-1OAc-CsBr-14-SP-TFE-PBE.log

---

Using Gaussian 16: ES64L-G16RevA.03 25-Dec-2016

==

# pbepbe/Def2QZVP/auto empiricaldispersion=gd3bj
scf=(maxcycle=300,direct,vshift=200,tight,yqc) density=current
scrf=(solvent=2,2,2-Trifluoroethanol,smd)

Pointgroup= C1 Stoichiometry= C16H28B10BrCsO8RuSe C1[X(C16H28B10BrCsO8RuSe)]
#Atoms= 66
Charge = 0 Multiplicity = 1

SCF Energy= -6566.54060668

==

Optimization incomplete.

Atomic Type	Coordinates (Angstroms) X	Y	Z
B	-4.465040	1.614539	-0.091001
B	-4.248180	2.525989	1.431859
H	-5.128580	2.676999	2.228689
H	-5.491450	1.081329	-0.401541
B	-3.063910	3.814209	1.126839
B	-2.552800	2.324959	1.960159
B	-3.401840	2.347969	-1.321031
B	-2.909880	0.876479	-0.461411
H	-3.516030	2.341019	-2.509651
H	2.239650	1.113760	2.089869
H	-2.115540	2.379909	3.072219
H	-2.958381	4.868659	1.680119

C	-1.816490	2.172839	-0.688641
C	-1.611640	3.012959	0.718499
B	-4.235390	3.388699	-0.139481
B	-2.547590	3.710739	-0.565341
H	-2.047961	4.602639	-1.173591
H	-5.087581	4.159629	-0.473501
B	-3.428540	0.965979	1.228729
H	-3.694030	-0.022701	1.844979
B	-1.736530	1.249329	0.784189
H	-0.663500	0.813020	1.362169
C	-0.560210	2.048880	-1.592201
O	0.128890	0.960920	-1.400251
O	-0.270950	2.954570	-2.378601
Cs	3.121620	0.574940	-1.777591
C	-0.251020	3.633620	0.989699
H	0.386300	2.947730	1.569039
H	0.252730	3.888590	0.043949
H	-0.393521	4.558690	1.568889
Ru	-0.240180	-0.371640	0.184699
O	3.607030	2.409441	0.651989
O	1.575800	-0.259990	1.458819
C	3.266630	2.727370	1.791819
C	1.072731	-1.163860	2.253269
O	2.495890	1.963050	2.586479
O	-0.076799	-1.625650	1.963199
C	1.849211	-1.647820	3.444669
H	1.170091	-2.078180	4.194979
H	2.547331	-2.431880	3.102429
H	2.434800	-0.824240	3.884209
C	3.635269	4.037391	2.453179
H	2.749909	4.697780	2.470179
H	3.941040	3.876351	3.499129
H	4.437969	4.530201	1.887959
Se	-2.252799	-0.875701	-1.209391
C	-3.444929	-2.043721	-0.214021
C	-4.791519	-2.116931	-0.599611
C	-2.937119	-2.836021	0.822809
C	-5.651239	-2.985271	0.086749
H	-5.169499	-1.492501	-1.415111
C	-3.807389	-3.707231	1.495229
H	-1.886479	-2.754221	1.113029
C	-5.160009	-3.781161	1.132489
H	-6.706859	-3.037822	-0.198571
H	-3.421479	-4.326621	2.311489
H	-5.834049	-4.459451	1.665429
Br	0.867741	-2.265300	-1.142981

O	4.630331	-1.696979	-0.356121
C	4.467141	-2.501399	0.560389
O	3.306191	-3.130020	0.819539
C	5.544541	-2.891449	1.550349
H	5.567321	-3.983809	1.692299
H	6.521141	-2.529509	1.201169
H	5.321791	-2.437759	2.532519
H	2.601621	-2.814190	0.165549

Supporting Information: 030-Selenylated-Cluster-RuII-2HOAc-1OAc-CsBr-15-SP-TFE-PBE.log

----

Using Gaussian 16: ES64L-G16RevA.03 25-Dec-2016

==

# pbepbe/Def2QZVP/auto empiricaldispersion=gd3bj
scf=(maxcycle=300,direct,vshift=200,tight,yqc) density=current
scrf=(solvent=2,2,2-Trifluoroethanol,smd)

Pointgroup= C1 Stoichiometry= C16H28B10BrCsO8RuSe C1[X(C16H28B10BrCsO8RuSe)]
#Atoms= 66
Charge = 0 Multiplicity = 1

SCF Energy= -6566.53433197

==

Optimization incomplete.

Atomic Type	Coordinates (Angstroms) X	Y	Z
B	-4.472410	1.612548	0.456918
B	-4.538220	1.468198	2.241358
H	-5.549250	1.234427	2.836858
H	-5.424060	1.467077	-0.254462
B	-3.304601	2.557849	2.921988
B	-2.973220	0.813609	2.802448
B	-3.201901	2.795609	0.057298
B	-2.869510	1.052369	-0.027682
H	-3.085341	3.477419	-0.915482
H	-2.752479	0.176679	3.790098
H	-3.302371	3.090209	3.991618
C	-1.764320	2.126010	0.705098
C	-1.806210	1.967850	2.341968
B	-4.233171	3.061028	1.484948
B	-2.500061	3.385379	1.580688
H	-1.890162	4.401039	1.687208
H	-5.006772	3.971617	1.540758

B	-3.702859	0.224868	1.295358
H	-4.076378	-0.903702	1.175988
B	-1.956879	0.513449	1.366148
H	-1.181349	-0.499710	1.729358
C	-0.381061	2.405750	0.088358
O	-0.063741	3.577531	-0.167942
O	0.343970	1.350561	-0.094032
C	-0.499220	2.154340	3.096688
H	-0.707360	2.138250	4.177018
H	0.208160	1.344631	2.854188
H	-0.037241	3.120481	2.840998
Ru	-0.346309	-0.592040	0.258018
O	-0.368067	-2.757370	0.141518
C	0.525493	-2.721399	-0.815672
O	0.843792	-1.550099	-1.239152
C	1.192104	-3.950549	-1.312672
H	0.613254	-4.849209	-1.054972
H	1.363004	-3.886008	-2.398662
H	2.191454	-3.978788	-0.809082
Se	-2.053749	-0.053361	-1.515462
C	-3.483278	-1.368432	-1.636322
C	-4.625038	-1.012162	-2.369512
C	-3.358517	-2.632031	-1.045482
C	-5.671278	-1.936523	-2.494032
H	-4.704479	-0.020402	-2.825572
C	-4.410057	-3.550212	-1.186992
H	-2.460477	-2.887051	-0.476412
C	-5.563877	-3.205073	-1.905132
H	-6.570948	-1.661384	-3.053512
H	-4.324436	-4.539002	-0.724862
H	-6.382096	-3.924973	-2.006042
Br	4.090133	-2.899057	0.537358
Cs	3.396671	0.313293	-0.715532
O	1.285181	-0.687878	1.667168
C	1.524472	-1.629748	2.476508
O	0.836893	-2.753509	2.521538
C	2.641222	-1.527568	3.461868
H	3.516622	-1.979287	2.943018
H	2.855801	-0.476537	3.703298
H	2.420212	-2.110448	4.368208
H	0.270333	-2.834139	1.688188
O	3.424159	3.301753	-1.305732
C	3.088538	4.443053	-1.634382
O	1.870908	4.955682	-1.432462
C	4.010497	5.431703	-2.317262
H	5.001068	4.981404	-2.467672

H	3.584977	5.735783	-3.288482
H	4.103267	6.345353	-1.706322
H	1.283138	4.278192	-0.968572

Supporting Information: 030-Selenylated-Cluster-RuII-2HOAc-1OAc-CsBr-16-SP-TFE-PBE.log

----

Using Gaussian 16: ES64L-G16RevA.03 25-Dec-2016

==

# pbepbe/Def2QZVP/auto empiricaldispersion=gd3bj
scf=(maxcycle=300,direct,vshift=200,tight,yqc) density=current
scrf=(solvent=2,2,2-Trifluoroethanol,smd)

Pointgroup= C1 Stoichiometry= C16H28B10BrCsO8RuSe C1[X(C16H28B10BrCsO8RuSe)]
#Atoms= 66
Charge = 0 Multiplicity = 1

SCF Energy= -6566.53902584

==

Optimization incomplete.

Atomic Type	Coordinates (Angstroms) X	Y	Z
B	-4.585550	-0.837759	-0.441521
B	-4.655740	-0.877439	-2.229241
H	-5.604100	-0.493169	-2.850341
H	-5.468140	-0.408089	0.244439
B	-3.713661	-2.282219	-2.777691
B	-2.996860	-0.653089	-2.852771
B	-3.594841	-2.219819	0.093669
B	-2.893910	-0.593229	-0.011861
H	-3.614741	-2.798799	1.137739
H	-2.663140	-0.195969	-3.906841
H	-3.849451	-2.916419	-3.781971
C	-2.056891	-1.954219	-0.618041
C	-2.112601	-1.982269	-2.262291
B	-4.692811	-2.407279	-1.297381
B	-3.073051	-3.112159	-1.350361
H	-2.705821	-4.243699	-1.338521
H	-5.649061	-3.125319	-1.251731
B	-3.546300	0.249091	-1.426831
H	-3.667390	1.437681	-1.437251
B	-1.908020	-0.421699	-1.449051
H	-0.839500	0.137880	-1.952561
C	-0.738481	-2.470760	0.014179

O	-0.617111	-3.667320	0.295939
O	0.175699	-1.556400	0.153939
C	-0.901911	-2.537960	-2.994371
H	-1.188551	-2.782220	-4.028171
H	-0.089261	-1.792720	-3.015491
H	-0.541261	-3.454430	-2.501511
Ru	-0.083870	0.441580	-0.455291
Se	-1.835850	0.442260	1.356349
C	-2.848550	2.097871	1.253859
C	-4.093760	2.158291	1.896719
C	-2.308160	3.212011	0.600159
C	-4.821740	3.355451	1.862539
H	-4.497890	1.277371	2.405329
C	-3.044159	4.406461	0.581819
H	-1.342360	3.137980	0.093489
C	-4.297099	4.479681	1.207429
H	-5.800540	3.407001	2.350219
H	-2.633209	5.281731	0.068089
H	-4.867529	5.413781	1.184309
O	1.543320	0.726500	-1.913301
C	1.207000	1.969200	-2.037581
C	1.999610	2.903870	-2.911361
H	2.668521	3.501300	-2.267741
H	2.605530	2.337600	-3.634791
H	1.323031	3.595210	-3.437541
O	0.193030	2.391200	-1.378451
Cs	3.249149	-1.572111	-0.265831
Br	1.512700	1.210330	1.428359
H	0.993260	-0.693190	2.756029
O	0.541259	-1.464210	3.202179
C	1.311689	-2.556880	3.025999
C	0.614459	-3.817880	3.472619
H	-0.081121	-3.625250	4.303209
H	0.033079	-4.188690	2.608389
H	1.357489	-4.578130	3.753149
O	2.434029	-2.522990	2.519229
O	4.984660	0.956359	-0.463671
C	4.893460	2.177959	-0.577541
O	3.869840	2.908939	-0.096931
H	3.207780	2.297729	0.351589
C	5.904880	3.044649	-1.295841
H	6.138521	3.943699	-0.703611
H	6.818040	2.466919	-1.493201
H	5.478700	3.386909	-2.255701

Supporting Information: 030-Selenylated-Cluster-RuII-2HOAc-1OAc-CsBr-17-SP-TFE-PBE.log

----

Using Gaussian 16: ES64L-G16RevA.03 25-Dec-2016
==
pbepbe/Def2QZVP/auto empiricaldispersion=gd3bj scf=(maxcycle=300,direct,vshift=200,tight,yqc) density=current scrf=(solvent=2,2,2-Trifluoroethanol,smd)

Pointgroup= C1 Stoichiometry= C16H28B10BrCsO8RuSe C1[X(C16H28B10BrCsO8RuSe)] #Atoms= 66

Charge = 0 Multiplicity = 1

SCF Energy= -6566.52412702
==
Optimization incomplete.

Atomic Type	Coordinates (Angstroms) X	Y	Z
B	2.035244	3.141707	-0.549130
B	1.624925	4.677768	-1.323930
H	2.442406	5.510627	-1.587700
H	3.148193	2.836906	-0.243280
B	-0.007154	5.140339	-0.788690
B	0.227175	4.408859	-2.392800
B	0.653863	2.628919	0.454540
B	0.896972	1.926178	-1.138980
H	0.695713	2.228299	1.681480
H	2.394399	-1.148543	2.323540
H	-0.083824	5.020659	-3.370770
H	-0.473253	6.237040	-0.703680
C	-0.664767	2.480040	-0.639040
C	-1.046126	3.908980	-1.366950
B	1.101845	4.357698	0.363690
B	-0.603486	3.896940	0.320260
H	-1.482005	4.051901	1.111490
H	1.534286	4.946408	1.311690
B	1.486694	3.155268	-2.261490
H	2.198543	2.870817	-3.179550
B	-0.219767	2.696580	-2.293810
H	-0.859368	2.097450	-3.100990
C	-1.772618	1.472631	-0.287060
O	-1.456949	0.618131	0.623310
O	-2.867208	1.528642	-0.889590
Cs	-2.284662	-2.285008	-0.610520
C	-2.488675	4.239892	-1.722710

H	-2.865356	3.572202	-2.508420
H	-3.145405	4.146123	-0.846880
H	-2.512644	5.280482	-2.081190
Ru	0.481061	0.541029	1.455890
O	0.142579	-1.552641	1.696460
O	0.248171	0.847019	3.640050
C	1.032238	-2.428212	1.904400
C	1.491431	0.646378	3.810780
O	2.284558	-2.150233	2.214750
O	2.223071	0.433177	2.746010
C	2.134571	0.646017	5.167870
H	2.568170	-0.348443	5.372150
H	2.962382	1.374536	5.188720
H	1.395781	0.893648	5.942830
C	0.729646	-3.876331	1.733010
H	0.797826	-4.071462	0.628690
H	1.458835	-4.504742	2.262910
H	-0.295444	-4.100400	2.065980
Se	1.060670	-0.068362	-0.921480
C	2.974470	-0.351784	-1.055220
C	3.412089	-1.175234	-2.101950
C	3.860310	0.166795	-0.101360
C	4.783279	-1.447966	-2.211940
H	2.686758	-1.642144	-2.775210
C	5.227970	-0.115836	-0.227190
H	3.487041	0.767906	0.731070
C	5.689559	-0.915237	-1.283800
H	5.137038	-2.095506	-3.020330
H	5.930851	0.286263	0.509730
H	6.757969	-1.136618	-1.374280
Br	0.742176	-3.547941	-1.793260
O	-5.037571	-1.143255	0.071290
C	-5.652020	-0.154925	0.480770
O	-5.206759	1.103835	0.374200
C	-7.000480	-0.221663	1.165590
H	-7.343011	-1.263853	1.218800
H	-6.927090	0.201247	2.181870
H	-7.735889	0.388238	0.614540
H	-4.308059	1.103454	-0.090680

Supporting Information: 030-Selenylated-Cluster-RuII-2HOAc-1OAc-CsBr-18-SP-TFE-PBE.log

----

Using Gaussian 16: ES64L-G16RevA.03 25-Dec-2016

==

# pbepbe/Def2QZVP/auto empiricaldispersion=gd3bj
scf=(maxcycle=300,direct,vshift=200,tight,yqc) density=current
scrf=(solvent=2,2,2-Trifluoroethanol,smd)

Pointgroup= C1 Stoichiometry= C16H28B10BrCsO8RuSe C1[X(C16H28B10BrCsO8RuSe)]
#Atoms= 66
Charge = 0 Multiplicity = 1

SCF Energy= -6566.50575726

===

Optimization incomplete.

Atomic Type	Coordinates (Angstroms) X	Y	Z
B	4.302530	1.660948	0.879710
B	5.035230	2.456258	-0.532540
H	6.218260	2.525498	-0.703640
H	4.927380	1.128368	1.752230
B	3.960300	3.786099	-1.020790
B	3.923810	2.247839	-1.907650
B	2.774700	2.499919	1.255890
B	2.760050	0.968359	0.358890
H	2.211690	2.574249	2.307570
H	-2.090661	-2.144840	1.532880
H	4.181900	2.220128	-3.074600
H	4.243501	4.798608	-1.589710
C	1.789430	2.291939	-0.136120
C	2.484030	3.047729	-1.460100
B	4.189500	3.432898	0.705510
B	2.583050	3.819799	0.085840
H	1.895081	4.770759	0.268470
H	4.740661	4.205728	1.434620
B	4.138820	0.926698	-0.744450
H	4.652739	-0.106742	-1.055210
B	2.518730	1.316049	-1.358920
H	1.788869	0.705569	-2.072640
C	0.253750	2.380630	-0.017000
O	-0.434710	1.377570	0.381990
O	-0.275450	3.491920	-0.267660
Cs	-3.878101	-0.568779	-1.361850
C	1.580280	3.656639	-2.521200
H	0.834110	2.924489	-2.864900
H	1.051561	4.534619	-2.128200
H	2.208190	3.949099	-3.376720
Ru	-0.342521	-0.605300	0.760770
O	-2.801422	-3.486379	-0.200780

O	-2.727161	-0.454280	1.564550
C	-1.673482	-3.547570	0.269970
C	-2.161211	0.034830	2.625220
O	-1.305302	-2.754010	1.341030
O	-0.892991	-0.030460	2.777250
C	-2.985041	0.752271	3.664600
H	-2.490861	0.715950	4.646940
H	-3.064380	1.812041	3.359850
H	-3.999231	0.327361	3.726050
C	-0.548122	-4.418500	-0.207460
H	-0.014692	-3.847830	-0.990100
H	0.163768	-4.644670	0.599790
H	-0.953172	-5.339200	-0.651370
Se	1.963579	-0.640851	1.291680
C	3.077379	-2.013791	0.449920
C	4.154689	-2.477802	1.219980
C	2.825969	-2.530841	-0.828170
C	5.001958	-3.464632	0.694840
H	4.335899	-2.062492	2.216730
C	3.675698	-3.522071	-1.339760
H	1.979059	-2.156151	-1.410290
C	4.762218	-3.988192	-0.583720
H	5.848508	-3.824162	1.288900
H	3.488168	-3.926651	-2.340150
H	5.423188	-4.760062	-0.991300
Br	-0.340051	-1.132600	-1.775090
O	-3.717460	2.395321	-0.735800
C	-3.725480	2.989911	0.346940
O	-2.673520	3.587330	0.903300
H	-1.832970	3.465950	0.329650
C	-4.966060	3.084591	1.220040
H	-4.911000	3.933371	1.917660
H	-5.862300	3.169391	0.586520
H	-5.052210	2.156351	1.813670

Supporting Information: 030-Selenylated-Cluster-RuII-2HOAc-1OAc-CsBr-19-SP-TFE-PBE.log

----

Using Gaussian 16: ES64L-G16RevA.03 25-Dec-2016

==

# pbepbe/Def2QZVP/auto empiricaldispersion=gd3bj
scf=(maxcycle=300,direct,vshift=200,tight,yqc) density=current
scrf=(solvent=2,2,2-Trifluoroethanol,smd)

Pointgroup= C1 Stoichiometry= C16H28B10BrCsO8RuSe C1[X(C16H28B10BrCsO8RuSe)]
#Atoms= 66
Charge = 0 Multiplicity = 1

SCF Energy= -6566.50145859
==
Optimization incomplete.

Atomic Coordinates (Angstroms)

Type	X	Y	Z
B	4.736521	1.272680	0.431901
B	5.164241	2.322450	-0.937349
H	6.284311	2.445819	-1.341889
H	5.523991	0.606719	1.041061
B	4.033871	3.694300	-0.941629
B	3.783571	2.337800	-2.058889
B	3.340441	1.995760	1.269431
B	3.102321	0.653840	0.138021
H	3.017711	1.875680	2.413381
H	-2.506690	-2.025079	1.688941
H	3.786281	2.522750	-3.239869
H	4.205411	4.798690	-1.365879
C	2.072081	2.021310	0.108751
C	2.485351	3.016480	-1.185329
B	4.617651	3.044620	0.605601
B	2.920371	3.499410	0.417321
H	2.301141	4.387620	0.909461
H	5.322741	3.688830	1.327111
B	4.213541	0.837840	-1.222759
H	4.626801	-0.113440	-1.816309
B	2.504641	1.298770	-1.399379
H	1.631891	0.806110	-2.040459
C	0.610741	2.085950	0.606101
O	0.134171	1.112970	1.324311
O	-0.027299	3.136390	0.419321
Cs	-2.933949	1.635191	0.481631
C	1.409461	3.793830	-1.929519
H	0.566321	3.139590	-2.194679
H	1.030711	4.625420	-1.322169
H	1.859731	4.182870	-2.856089
Ru	0.120740	-0.901010	1.106121
O	-4.100470	-1.369819	0.125901
O	-1.841340	-0.706989	2.431371
C	-3.445900	-2.427379	0.069891
C	-1.052590	-0.885630	3.452701
O	-2.626560	-2.806549	1.050331
O	0.194120	-1.062770	3.251691

C	-1.612720	-0.882469	4.850601
H	-0.819720	-1.079230	5.585981
H	-2.077329	0.095831	5.063961
H	-2.403800	-1.646949	4.939891
C	-3.453330	-3.371929	-1.099959
H	-2.574820	-3.106299	-1.717479
H	-3.337170	-4.415309	-0.770449
H	-4.364590	-3.248449	-1.702099
Se	2.482730	-1.127500	0.858571
C	3.167720	-2.245560	-0.596339
C	4.444060	-2.793870	-0.396469
C	2.435950	-2.514920	-1.759779
C	5.007130	-3.604610	-1.392059
H	4.998040	-2.575420	0.522381
C	3.007950	-3.332920	-2.745529
H	1.438610	-2.084450	-1.890299
C	4.289770	-3.874090	-2.566929
H	6.006680	-4.026971	-1.246029
H	2.444910	-3.543870	-3.660929
H	4.729970	-4.509030	-3.342699
Br	-0.692520	-0.746000	-1.308329
O	-5.228379	1.321071	-1.551739
C	-5.793099	0.451721	-2.220409
O	-5.704150	-0.862669	-1.969579
H	-5.095540	-1.004549	-1.167619
C	-6.665549	0.742631	-3.422819
H	-6.759669	1.827791	-3.563969
H	-6.222349	0.287151	-4.324829
H	-7.662369	0.290421	-3.288859

Supporting Information: 030-Selenylated-Cluster-RuII-2HOAc-1OAc-CsBr-20-SP-TFE-PBE.log

----

Using Gaussian 16: ES64L-G16RevA.03 25-Dec-2016

==

# pbepbe/Def2QZVP/auto empiricaldispersion=gd3bj
scf=(maxcycle=300,direct,vshift=200,tight,yqc) density=current
scrf=(solvent=2,2,2-Trifluoroethanol,smd)

Pointgroup= C1 Stoichiometry= C16H28B10BrCsO8RuSe C1[X(C16H28B10BrCsO8RuSe)]
#Atoms= 66
Charge = 0 Multiplicity = 1

SCF Energy= -6566.53087366

==

Optimization incomplete.

Atomic Type	Coordinates (Angstroms) X	Y	Z
B	-4.447568	-0.445722	1.330799
B	-5.523628	-1.203252	0.136679
H	-6.701338	-0.991513	0.089019
H	-4.811519	0.334018	2.164139
B	-4.867767	-2.811582	-0.238891
B	-4.584198	-1.458032	-1.351211
B	-3.125788	-1.584291	1.687539
B	-2.860248	-0.239811	0.566709
H	-2.471298	-1.670051	2.683039
H	3.786301	0.596832	0.784439
H	-4.977858	-1.513572	-2.478871
H	-5.453207	-3.784782	-0.613031
C	-2.300398	-1.820331	0.198219
C	-3.330747	-2.535291	-0.929231
B	-4.777577	-2.196022	1.424769
B	-3.398297	-3.049361	0.718199
H	-2.936206	-4.114371	0.980189
H	-5.395267	-2.707282	2.313859
B	-4.321438	0.007848	-0.394331
H	-4.605859	1.090378	-0.814661
B	-2.932618	-0.861461	-1.090401
H	-2.188598	-0.546821	-1.963471
C	-0.822257	-2.297490	0.236769
O	0.023322	-1.574209	0.883539
O	-0.563417	-3.414120	-0.253151
Cs	2.560633	-2.734048	-0.554341
C	-2.782077	-3.485121	-1.984221
H	-1.903427	-3.052160	-2.482461
H	-2.490126	-4.444001	-1.538211
H	-3.575077	-3.645471	-2.731531
Ru	0.605681	0.435141	0.793629
O	4.657802	-0.518837	-1.102161
O	2.642702	-0.352978	1.531139
C	4.743961	0.690153	-0.884041
C	2.230592	-0.050718	2.730289
O	4.308831	1.279033	0.245669
O	1.051101	0.407481	2.871529
C	3.140052	-0.230698	3.916569
H	2.604982	-0.003678	4.849659
H	3.524582	-1.264378	3.948609
H	4.010931	0.441782	3.821159
C	5.321031	1.697583	-1.852321

H	4.500480	2.339153	-2.219261
H	6.048960	2.352443	-1.345541
H	5.795881	1.182013	-2.697851
Se	-1.617039	1.250350	1.122269
C	-2.261450	2.633689	-0.103401
C	-3.185150	3.547159	0.427539
C	-1.813200	2.748890	-1.425851
C	-3.680321	4.578359	-0.383451
H	-3.522130	3.443849	1.464149
C	-2.310610	3.787209	-2.226801
H	-1.092379	2.028340	-1.821811
C	-3.242911	4.699639	-1.710531
H	-4.407231	5.288088	0.025129
H	-1.966860	3.878750	-3.262691
H	-3.628531	5.506539	-2.342251
Br	0.659172	0.107901	-1.805191
O	1.462260	2.363641	0.911729
C	1.962250	3.116411	0.044889
O	1.958520	2.888151	-1.260251
H	1.529021	1.982191	-1.445641
C	2.669629	4.379242	0.448469
H	2.366379	4.672632	1.462819
H	3.756350	4.181242	0.441989
H	2.462879	5.188872	-0.268471

Supporting Information: 030-Selenylated-Cluster-RuII-2HOAc-1OAc-CsBr-21-SP-TFE-PBE.log

----

Using Gaussian 16: ES64L-G16RevA.03 25-Dec-2016

===

# pbepbe/Def2QZVP/auto empiricaldispersion=gd3bj
scf=(maxcycle=300,direct,vshift=200,tight,yqc) density=current
scrf=(solvent=2,2,2-Trifluoroethanol,smd)

Pointgroup= C1 Stoichiometry= C16H28B10BrCsO8RuSe C1[X(C16H28B10BrCsO8RuSe)]
#Atoms= 66
Charge = 0 Multiplicity = 1

SCF Energy= -6566.52687409

===

Optimization incomplete.

Atomic Coordinates (Angstroms)
Type X Y Z

B	-4.352100	-0.797812	1.199249

B	-5.271080	-1.798362	0.054729
H	-6.459430	-1.723623	-0.073111
H	-4.842191	0.028088	1.916149
B	-4.431159	-3.357962	-0.094331
B	-4.222430	-2.117642	-1.345631
B	-2.944360	-1.738091	1.752249
B	-2.753251	-0.512811	0.488999
H	-2.345670	-1.634270	2.780999
H	3.741129	1.082613	0.365869
H	-4.540210	-2.344522	-2.475651
H	-4.890088	-4.425302	-0.376951
C	-2.009400	-2.047620	0.342959
C	-2.890879	-2.994541	-0.737791
B	-4.505729	-2.551672	1.486149
B	-3.005829	-3.323821	0.953309
H	-2.454778	-4.295101	1.363629
H	-5.119879	-3.022072	2.399909
B	-4.170281	-0.531712	-0.560311
H	-4.538171	0.460008	-1.118071
B	-2.660610	-1.316631	-1.083631
H	-1.902390	-1.020490	-1.949641
C	-0.492820	-2.358409	0.508749
O	0.261960	-1.463409	1.045449
O	-0.116759	-3.511479	0.220269
Cs	2.848201	-2.567897	-0.361321
C	-2.180789	-3.988950	-1.644681
H	-1.325029	-3.515440	-2.146651
H	-1.817548	-4.853140	-1.074791
H	-2.903298	-4.319251	-2.407461
Ru	0.613249	0.564571	0.649279
O	4.622859	-0.281846	-1.322851
O	2.761259	0.107152	1.464599
C	4.385209	0.923183	-1.421611
C	2.347689	0.498542	2.618299
O	4.000778	1.692323	-0.385321
O	1.124479	0.891302	2.740299
C	3.248879	0.463843	3.824529
H	2.905258	1.179393	4.586549
H	3.222879	-0.547337	4.270039
H	4.289399	0.680243	3.535379
C	4.435618	1.699423	-2.714191
H	3.405488	1.717133	-3.115661
H	4.755598	2.739994	-2.551091
H	5.093338	1.196014	-3.436611
Se	-1.678252	1.132830	0.936969
C	-2.391432	2.392710	-0.380041

C	-3.560303	3.085899	-0.028421
C	-1.708182	2.677760	-1.569341
C	-4.065553	4.065529	-0.894541
H	-4.074133	2.855249	0.910709
C	-2.217613	3.669790	-2.422001
H	-0.802292	2.119280	-1.825811
C	-3.391993	4.361699	-2.089381
H	-4.982844	4.601968	-0.630331
H	-1.693163	3.894090	-3.357191
H	-3.783724	5.132619	-2.761121
Br	0.745379	0.007931	-1.848641
O	1.117998	2.537242	0.099779
C	1.009567	3.554541	0.819909
O	0.748357	3.520681	2.125759
H	0.744218	2.556271	2.420199
C	1.104446	4.926312	0.218979
H	1.898286	4.936522	-0.542971
H	0.148486	5.148791	-0.289631
H	1.291086	5.690272	0.986819

Supporting Information: 030-Selenylated-Cluster-RuII-2HOAc-1OAc-CsBr-22-SP-TFE-PBE.log

----

Using Gaussian 16: ES64L-G16RevA.03 25-Dec-2016

==

# pbepbe/Def2QZVP/auto empiricaldispersion=gd3bj
scf=(maxcycle=300,direct,vshift=200,tight,yqc) density=current
scrf=(solvent=2,2,2-Trifluoroethanol,smd)

Pointgroup= C1 Stoichiometry= C16H28B10BrCsO8RuSe C1[X(C16H28B10BrCsO8RuSe)]
#Atoms= 66

Charge = 0 Multiplicity = 1

SCF Energy= -6566.52412761

==

Optimization incomplete.

Atomic Type	Coordinates (Angstroms) X	Y	Z
B	2.035004	3.142168	-0.547850
B	1.624936	4.678178	-1.322840
H	2.442417	5.511307	-1.585770
H	3.147754	2.837606	-0.241020
B	-0.007743	5.140220	-0.789020
B	0.228236	4.408990	-2.393010

B	0.652844	2.628800	0.454480
B	0.897673	1.926349	-1.138920
H	0.694043	2.228319	1.681580
H	2.392809	-1.148093	2.324950
H	-0.082033	5.020841	-3.371180
H	-0.474292	6.236751	-0.704340
C	-0.664697	2.479701	-0.640360
C	-1.045835	3.908612	-1.368380
B	1.100416	4.357749	0.364270
B	-0.604725	3.896501	0.319220
H	-1.484015	4.050992	1.109690
H	1.531857	4.946428	1.312760
B	1.488034	3.155758	-2.260730
H	2.200814	2.871698	-3.178180
B	-0.218256	2.696551	-2.294660
H	-0.856937	2.097432	-3.102560
C	-1.772508	1.471893	-0.289420
O	-1.457459	0.617702	0.621450
O	-2.866508	1.527314	-0.893100
Cs	-2.284473	-2.286477	-0.611710
C	-2.488184	4.239034	-1.725400
H	-2.863945	3.571194	-2.511430
H	-3.145644	4.145024	-0.850130
H	-2.512223	5.279604	-2.083900
Ru	0.479791	0.541140	1.455760
O	0.141568	-1.552690	1.696120
O	0.245081	0.847210	3.639780
C	1.031347	-2.428061	1.904400
C	1.488231	0.646698	3.811490
O	2.283357	-2.149793	2.215770
O	2.220741	0.433528	2.747320
C	2.130331	0.646638	5.169080
H	2.565390	-0.347213	5.373200
H	2.956952	1.376497	5.190900
H	1.390581	0.892799	5.943590
C	0.729355	-3.876221	1.732290
H	0.798225	-4.070951	0.627940
H	1.458454	-4.504582	2.262390
H	-0.295835	-4.100769	2.064630
Se	1.061440	-0.068161	-0.921130
C	2.975380	-0.351323	-1.053370
C	3.413909	-1.174734	-2.099770
C	3.860440	0.167405	-0.098870
C	4.785218	-1.447256	-2.208730
H	2.689138	-1.641753	-2.773560
C	5.228240	-0.115026	-0.223680

H	3.486461	0.768496	0.733270
C	5.690729	-0.914377	-1.279930
H	5.139677	-2.094756	-3.016860
H	5.930511	0.287203	0.513750
H	6.759239	-1.135598	-1.369610
Br	0.743456	-3.547331	-1.794020
O	-5.035671	-1.143423	0.075110
C	-5.650390	-0.154222	0.482030
O	-5.205858	1.104457	0.371490
H	-4.307328	1.103186	-0.093720
C	-6.998450	-0.219610	1.167770
H	-7.340671	-1.261770	1.223770
H	-6.924590	0.205839	2.182950
H	-7.734329	0.388710	0.615610

Supporting Information: 040-Decarboxylated-Cluster-RuII-2HOAc-1OAc-CsBr-01-SP-TFE-PBE.log

----

Using Gaussian 16: ES64L-G16RevA.03 25-Dec-2016

==

# pbepbe/Def2QZVP/auto empiricaldispersion=gd3bj
scf=(maxcycle=300,direct,vshift=200,tight,yqc) density=current
scrf=(solvent=2,2,2-Trifluoroethanol,smd)

Pointgroup= C1 Stoichiometry= C15H28B10BrCsO6RuSe C1[X(C15H28B10BrCsO6RuSe)]
#Atoms= 63
Charge = 0 Multiplicity = 1

SCF Energy= -6378.02797185

==

Optimization incomplete.

Atomic Type	Coordinates (Angstroms) X	Y	Z
B	-2.471080	1.873950	2.562880
B	-1.965479	0.965570	4.000490
H	-2.542769	1.049830	5.046720
H	-3.415970	2.610640	2.541360
B	-0.195179	0.794110	3.972270
B	-1.240339	-0.586680	3.493100
B	-0.987080	2.228010	1.598520
B	-2.024889	0.895690	1.183680
H	-0.828220	3.143560	0.849010
H	-1.192809	-1.613330	4.105040
H	0.550831	0.712630	4.904110

C	-0.327839	0.702300	1.156010
C	0.152661	-0.128320	2.632780
B	-0.932170	2.295000	3.368690
B	0.410790	1.542450	2.439380
H	1.537250	1.946000	2.368340
H	-0.765070	3.325350	3.956300
B	-2.637379	0.069780	2.600670
H	-3.692859	-0.493640	2.604510
B	-1.213629	-0.579630	1.714350
H	-1.168799	-1.631730	0.972400
C	1.361561	-1.038940	2.565290
H	1.177511	-1.941350	3.168680
H	1.535171	-1.358180	1.528240
H	2.257511	-0.534360	2.960040
Ru	-0.414219	-0.662010	-0.437950
Se	-2.517969	0.627100	-0.751380
C	-3.956059	-0.668710	-0.535470
C	-5.196339	-0.232961	-0.046280
C	-3.750799	-2.003660	-0.909640
C	-6.241509	-1.156841	0.091540
H	-5.339489	0.813339	0.242440
C	-4.807589	-2.917001	-0.774020
H	-2.776019	-2.322060	-1.292080
C	-6.048119	-2.498201	-0.271680
H	-7.207229	-0.826061	0.487300
H	-4.655119	-3.962401	-1.062170
H	-6.866679	-3.217051	-0.163100
O	1.341161	-2.083680	-0.544820
C	0.695751	-2.808730	-1.418330
C	1.372381	-3.995980	-2.055680
H	2.025111	-3.638310	-2.872200
H	2.002661	-4.534210	-1.329800
H	0.620111	-4.673320	-2.485030
O	-0.482189	-2.468470	-1.737230
Cs	3.169951	0.990910	-0.218710
Br	0.519371	0.471270	-2.523730
H	0.113520	2.622880	-2.014340
O	-0.182290	3.566520	-1.811960
C	0.784470	4.191890	-1.122100
C	0.369800	5.585850	-0.707500
H	-0.235520	6.070600	-1.488750
H	-0.254740	5.511030	0.200950
H	1.259680	6.187090	-0.474220
O	1.870160	3.683020	-0.833350
O	4.454441	-1.582040	0.849030
C	4.310691	-2.788650	1.065700

O	3.216021	-3.483670	0.727110
C	5.344751	-3.653700	1.752770
H	5.632161	-4.493109	1.097530
H	6.229861	-3.054099	2.004370
H	4.917041	-4.093380	2.669720
H	2.555101	-2.868580	0.250040

Supporting Information: 040-Decarboxylated-Cluster-RuII-2HOAc-1OAc-CsBr-02-SP-TFE-PBE.log

----

Using Gaussian 16: ES64L-G16RevA.03 25-Dec-2016

==

# pbepbe/Def2QZVP/auto empiricaldispersion=gd3bj
scf=(maxcycle=300,direct,vshift=200,tight,yqc) density=current
scrf=(solvent=2,2,2-Trifluoroethanol,smd)

Pointgroup= C1 Stoichiometry= C15H28B10BrCsO6RuSe C1[X(C15H28B10BrCsO6RuSe)]
#Atoms= 63
Charge = 0 Multiplicity = 1

SCF Energy= -6378.02882335

==

Optimization incomplete.

Atomic Type	Coordinates (Angstroms) X	Y	Z
B	1.401570	3.589001	0.156810
B	0.994070	3.984641	-1.524020
H	1.307020	5.023411	-2.032710
H	2.017180	4.317471	0.882740
B	-0.570160	3.235261	-1.918970
B	0.959260	2.481361	-2.491130
B	0.087880	2.528371	0.791280
B	1.579590	1.850291	0.218750
H	-0.232430	2.409351	1.936470
H	1.158990	2.361671	-3.664660
H	-1.391500	3.623991	-2.698920
C	0.086120	1.118001	-0.201860
C	-0.328070	1.590291	-1.819700
B	-0.305030	3.879361	-0.285480
B	-1.116050	2.287541	-0.479450
H	-2.269010	2.044641	-0.300640
H	-0.923000	4.834171	0.093380
B	2.179240	2.677871	-1.203170
H	3.345650	2.748021	-1.461780

B	1.262170	1.142241	-1.362510
H	1.738449	-0.013799	-1.687590
C	-1.117131	0.619171	-2.680380
H	-0.955940	0.877291	-3.738950
H	-0.766951	-0.413399	-2.520500
H	-2.195781	0.702011	-2.468110
Ru	0.935459	-0.799319	-0.203960
Se	2.457889	0.431361	1.347250
C	4.205239	0.385940	0.487400
C	5.105720	1.435840	0.720950
C	4.556409	-0.706100	-0.318070
C	6.371240	1.400910	0.119060
H	4.814330	2.282360	1.351080
C	5.829529	-0.733120	-0.908060
H	3.838689	-1.515090	-0.487500
C	6.734009	0.317230	-0.694960
H	7.072440	2.225260	0.285140
H	6.111219	-1.581840	-1.540250
H	7.723429	0.292620	-1.163120
O	-0.100951	-2.235399	-1.573050
C	0.894629	-3.057609	-1.464640
C	0.842539	-4.394549	-2.166540
H	0.373189	-5.140979	-1.500330
H	0.246609	-4.327839	-3.090340
H	1.861029	-4.742639	-2.396160
O	1.892099	-2.737789	-0.742510
Cs	-2.903341	-1.846269	-0.418450
Br	-0.093671	-2.022689	1.795790
H	-1.163891	-0.384979	2.761250
O	-1.503041	0.437461	3.261390
C	-2.610400	0.907921	2.710620
C	-3.021930	2.253731	3.250860
H	-2.614550	2.419791	4.258160
H	-2.608470	3.028211	2.579500
H	-4.117450	2.352491	3.249690
O	-3.238311	0.314471	1.808510
O	-4.554281	0.576391	-1.295910
C	-4.912440	1.672791	-0.852420
O	-4.836070	2.013491	0.442470
C	-5.438140	2.796741	-1.713480
H	-4.590400	3.456631	-1.973810
H	-5.864080	2.393071	-2.642770
H	-6.180430	3.403841	-1.173420
H	-4.322780	1.295201	0.943530

Supporting Information: 040-Decarboxylated-Cluster-RuII-2HOAc-1OAc-CsBr-03-SP-TFE-PBE.log

----

Using Gaussian 16: ES64L-G16RevA.03 25-Dec-2016
==
# pbepbe/Def2QZVP/auto empiricaldispersion=gd3bj
scf=(maxcycle=300,direct,vshift=200,tight,yqc) density=current
scrf=(solvent=2,2,2-Trifluoroethanol,smd)

Pointgroup= C1 Stoichiometry= C15H28B10BrCsO6RuSe C1[X(C15H28B10BrCsO6RuSe)]
#Atoms= 63
Charge = 0 Multiplicity = 1

SCF Energy= -6378.02453613
==
Optimization incomplete.

Atomic Type	Coordinates (Angstroms) X	Y	Z
B	-3.288391	2.649687	0.277430
B	-3.360822	2.854307	2.037030
H	-4.269542	3.414177	2.580980
H	-4.136632	3.042297	-0.472410
B	-1.703802	3.039238	2.657260
B	-2.558321	1.466098	2.825450
B	-1.547611	2.657259	-0.196370
B	-2.415401	1.160338	-0.008570
H	-1.101622	2.977699	-1.256980
H	-2.768840	1.014408	3.913010
H	-1.344142	3.654299	3.618740
C	-0.826251	1.318769	0.602160
C	-0.934211	1.600129	2.342020
B	-2.123242	3.742288	1.080390
B	-0.561932	2.863329	1.261240
H	0.531168	3.352910	1.300840
H	-2.142173	4.931828	0.939330
B	-3.519861	1.205717	1.348360
H	-4.526360	0.559047	1.373220
B	-1.894960	0.452968	1.521890
H	-1.616609	-0.802202	1.622370
C	0.168979	1.069500	3.239660
H	-0.263650	0.819029	4.221030
H	0.614670	0.152150	2.821690
H	0.939349	1.841720	3.402860
Ru	-0.379209	-0.686591	0.228900
Se	-2.218160	-0.261822	-1.421670

C	-3.662179	-1.453243	-0.887230
C	-4.987029	-1.095294	-1.176910
C	-3.358958	-2.668453	-0.257660
C	-6.026949	-1.959894	-0.807940
H	-5.206050	-0.142154	-1.668840
C	-4.408508	-3.529043	0.098980
H	-2.317388	-2.929212	-0.042600
C	-5.738798	-3.176064	-0.170370
H	-7.064239	-1.678925	-1.017230
H	-4.180397	-4.479633	0.592450
H	-6.553407	-3.849225	0.115880
O	1.231761	-1.408810	1.557390
C	0.953252	-2.639930	1.258570
C	1.755892	-3.765929	1.862880
H	2.407543	-4.193469	1.081570
H	2.373492	-3.403849	2.698670
H	1.080663	-4.564330	2.210410
O	0.011192	-2.878331	0.434610
Cs	2.996899	1.174291	0.771610
Br	1.260901	-0.736210	-1.733000
H	1.020839	1.373600	-2.615970
O	0.788089	2.245240	-3.055730
C	1.424848	3.237450	-2.410010
C	1.027207	4.597760	-2.933750
H	0.087617	4.900260	-2.437010
H	1.805387	5.334661	-2.690250
H	0.839117	4.570610	-4.017790
O	2.214528	3.059411	-1.480540
O	4.708301	-1.272128	0.078570
C	4.591042	-2.455458	-0.240280
O	3.548182	-2.955728	-0.927260
C	5.590392	-3.534617	0.115860
H	5.780093	-4.192287	-0.747130
H	6.526162	-3.076586	0.464130
H	5.175183	-4.164477	0.922780
H	2.894322	-2.216579	-1.124870

Supporting Information: 040-Decarboxylated-Cluster-RuII-2HOAc-1OAc-CsBr-04-SP-TFE-PBE.log

----

Using Gaussian 16: ES64L-G16RevA.03 25-Dec-2016

==

# pbepbe/Def2QZVP/auto empiricaldispersion=gd3bj
scf=(maxcycle=300,direct,vshift=200,tight,yqc) density=current
scrf=(solvent=2,2,2-Trifluoroethanol,smd)

Pointgroup= C1 Stoichiometry= C15H28B10BrCsO6RuSe C1[X(C15H28B10BrCsO6RuSe)]
#Atoms= 63

Charge = 0 Multiplicity = 1

SCF Energy= -6378.03577344

==

Optimization incomplete.

Atomic Type	Coordinates (Angstroms) X	Y	Z
B	3.576701	-2.072930	-1.939061
B	4.493351	-2.996940	-0.721971
H	5.523401	-3.561090	-0.962691
H	3.915251	-1.950110	-3.082551
B	3.328301	-3.689600	0.426849
B	4.231951	-2.227510	0.860479
B	1.852821	-2.222740	-1.518271
B	2.734981	-0.777640	-1.065311
H	0.956481	-2.167640	-2.305271
H	4.956361	-2.221960	1.813449
H	3.443801	-4.680970	1.087919
C	1.635251	-1.462320	0.025929
C	2.544481	-2.354320	1.161579
B	2.912551	-3.609790	-1.290551
B	1.687661	-3.185690	-0.053091
H	0.718821	-3.811860	0.263449
H	2.784061	-4.602060	-1.950911
B	4.384181	-1.213450	-0.595621
H	5.317821	-0.476330	-0.740281
B	3.149861	-0.851740	0.635409
H	3.125051	0.076580	1.378969
C	2.016201	-2.463740	2.582389
H	2.371721	-1.620750	3.193649
H	0.915931	-2.462650	2.576529
H	2.367491	-3.405090	3.032649
Ru	0.140391	-0.033690	0.199799
Se	1.693901	0.911980	-1.534371
C	2.764181	2.243240	-0.608061
C	4.118271	2.394760	-0.944761
C	2.159281	3.073830	0.345129
C	4.880941	3.377230	-0.298821
H	4.578601	1.741100	-1.691421
C	2.931671	4.058360	0.978499
H	1.101831	2.941150	0.592479
C	4.289701	4.209850	0.663179

H	5.941211	3.488180	-0.548841
H	2.464521	4.706690	1.727469
H	4.888611	4.975980	1.166489
O	-0.891059	-0.721690	2.091289
C	-0.059239	0.028000	2.743829
C	-0.183939	0.197070	4.236819
H	-0.899859	1.008730	4.461579
H	-0.551649	-0.731200	4.702959
H	0.788541	0.477860	4.668649
O	0.853801	0.635740	2.093919
Cs	-3.850999	-0.325230	1.405269
O	-0.905669	-0.747590	-1.524611
C	-1.579539	-1.796790	-1.435241
O	-1.669989	-2.467120	-0.277821
H	-0.988989	-2.000740	0.324259
C	-2.334689	-2.383610	-2.586071
H	-2.073879	-3.448620	-2.700921
H	-3.420429	-2.306050	-2.404231
H	-2.081309	-1.833790	-3.502941
Br	-1.519689	2.079030	0.231459
O	-4.265469	0.048950	-1.576931
C	-3.912379	0.690300	-2.576721
O	-2.930689	1.588690	-2.598161
H	-2.494209	1.661560	-1.681211
C	-4.545699	0.512880	-3.941491
H	-5.483579	-0.052550	-3.851931
H	-4.727389	1.489520	-4.417381
H	-3.850049	-0.041330	-4.596181

Supporting Information: 040-Decarboxylated-Cluster-RuII-2HOAc-1OAc-CsBr-05-SP-TFE-PBE.log

----

Using Gaussian 16: ES64L-G16RevA.03 25-Dec-2016

==

# pbepbe/Def2QZVP/auto empiricaldispersion=gd3bj
scf=(maxcycle=300,direct,vshift=200,tight,yqc) density=current
scrf=(solvent=2,2,2-Trifluoroethanol,smd)

Pointgroup= C1 Stoichiometry= C15H28B10BrCsO6RuSe C1[X(C15H28B10BrCsO6RuSe)]
#Atoms= 63
Charge = 0 Multiplicity = 1

SCF Energy= -6378.02882399

==

Optimization incomplete.

Atomic Type	Coordinates (Angstroms) X	Y	Z
B	1.401682	3.589049	0.156839
B	0.994183	3.984699	-1.524001
H	1.307183	5.023469	-2.032661
H	2.017283	4.317539	0.882749
B	-0.570078	3.235391	-1.918931
B	0.959312	2.481429	-2.491101
B	0.087942	2.528440	0.791299
B	1.579641	1.850339	0.218769
H	-0.232338	2.409430	1.936489
H	1.159052	2.361749	-3.664641
H	-1.391388	3.624161	-2.698891
C	0.086141	1.118100	-0.201881
C	-0.328039	1.590400	-1.819651
B	-0.304917	3.879470	-0.285441
B	-1.115999	2.287691	-0.479411
H	-2.268959	2.044842	-0.300581
H	-0.922847	4.834301	0.093429
B	2.179312	2.677899	-1.203141
H	3.345722	2.748018	-1.461761
B	1.262221	1.142299	-1.362491
H	1.738470	-0.013761	-1.687591
C	-1.117110	0.619301	-2.680351
H	-0.956260	0.877741	-3.738901
H	-0.766671	-0.413229	-2.520811
H	-2.195720	0.701852	-2.467781
Ru	0.935399	-0.799271	-0.204031
Se	2.457810	0.431308	1.347239
C	4.205200	0.385847	0.487469
C	5.105711	1.435706	0.721079
C	4.556359	-0.706183	-0.318031
C	6.371251	1.400745	0.119229
H	4.814331	2.282227	1.351219
C	5.829499	-0.733234	-0.907971
H	3.838609	-1.515133	-0.487511
C	6.734010	0.317075	-0.694801
H	7.072481	2.225065	0.285359
H	6.111179	-1.581954	-1.540181
H	7.723450	0.292444	-1.162931
O	-0.100992	-2.235350	-1.573211
C	0.894578	-3.057560	-1.464711
C	0.842537	-4.394550	-2.166521
H	0.374996	-5.141390	-1.499501
H	0.245117	-4.328410	-3.089391

H	1.860976	-4.741751	-2.397721
O	1.892018	-2.737731	-0.742551
Cs	-2.903462	-1.846308	-0.418421
Br	-0.093702	-2.022830	1.795629
H	-1.163821	-0.385099	2.761139
O	-1.502880	0.437391	3.261249
C	-2.610260	0.907872	2.710549
C	-3.021679	2.253742	3.250699
H	-2.613758	2.420122	4.257729
H	-2.608778	3.028132	2.578899
H	-4.117218	2.352313	3.250149
O	-3.238310	0.314363	1.808579
O	-4.553950	0.576664	-1.295901
C	-4.912259	1.672964	-0.852281
O	-4.836029	2.013454	0.442679
C	-5.437998	2.796994	-1.713191
H	-4.590288	3.456984	-1.973391
H	-5.863858	2.393445	-2.642571
H	-6.180358	3.403955	-1.173071
H	-4.322739	1.295143	0.943679

Supporting Information: 040-Decarboxylated-Cluster-RuII-2HOAc-1OAc-CsBr-06-SP-TFE-PBE.log

----

Using Gaussian 16: ES64L-G16RevA.03 25-Dec-2016

==

# pbepbe/Def2QZVP/auto empiricaldispersion=gd3bj
scf=(maxcycle=300,direct,vshift=200,tight,yqc) density=current
scrf=(solvent=2,2,2-Trifluoroethanol,smd)

Pointgroup= C1 Stoichiometry= C15H28B10BrCsO6RuSe C1[X(C15H28B10BrCsO6RuSe)]
#Atoms= 63
Charge = 0 Multiplicity = 1

SCF Energy= -6378.02916096

==

Optimization incomplete.

Atomic Type	Coordinates (Angstroms) X	Y	Z
B	-2.894681	3.248289	-1.195660
B	-3.271302	4.159859	0.289150
H	-4.037842	5.081079	0.319030
H	-3.372661	3.489599	-2.268300
B	-1.821152	4.155450	1.314540

B	-3.149361	3.037489	1.666040
B	-1.204131	2.700070	-1.057650
B	-2.511210	1.591069	-0.691210
H	-0.473891	2.498040	-1.983550
H	-3.721751	3.084569	2.716190
H	-1.487792	4.966420	2.129560
C	-1.124170	1.611720	0.283460
C	-1.514211	2.508230	1.686860
B	-1.644762	4.292280	-0.442090
B	-0.547931	3.229050	0.489030
H	0.608859	3.410931	0.732880
H	-1.224872	5.292100	-0.953240
B	-3.818091	2.463399	0.119150
H	-4.967631	2.139308	0.024670
B	-2.684230	1.451429	1.046990
H	-2.932490	0.440969	1.622760
C	-0.830151	2.125740	2.988930
H	-1.420790	1.371230	3.529430
H	0.169370	1.714301	2.780860
H	-0.721021	3.018710	3.623800
Ru	-0.276910	-0.280830	-0.040230
Se	-2.260570	-0.219240	-1.588450
C	-3.665299	-1.230761	-0.703610
C	-4.999429	-0.820862	-0.852770
C	-3.338859	-2.384371	0.021920
C	-6.018599	-1.565642	-0.243830
H	-5.240580	0.079988	-1.424710
C	-4.369528	-3.124061	0.620420
H	-2.292968	-2.692540	0.118680
C	-5.705498	-2.717062	0.494000
H	-7.059279	-1.240953	-0.346650
H	-4.119738	-4.024121	1.192310
H	-6.503958	-3.296432	0.969240
O	1.161251	-0.449649	1.682370
C	0.183631	-0.941399	2.373330
C	0.405481	-1.473689	3.765370
H	0.663221	-2.547079	3.713010
H	1.230651	-0.937179	4.260160
H	-0.517159	-1.379900	4.358430
O	-0.974319	-0.983790	1.837510
Cs	3.541141	-1.773678	0.266030
O	0.781600	0.240391	-1.840160
C	1.722770	1.053791	-1.741460
O	2.022600	1.584512	-0.544220
H	1.241880	1.239201	0.068030
C	2.585770	1.482792	-2.885030

H	2.460149	2.566332	-3.057080
H	3.649260	1.301932	-2.648860
H	2.298510	0.931982	-3.790860
Br	0.328142	-2.794489	-0.639360
O	5.165360	0.764083	-0.386310
C	5.384949	1.956053	-0.146210
O	4.419719	2.881913	-0.038550
H	3.536879	2.430952	-0.180420
C	6.760539	2.546964	0.057260
H	6.828149	3.008034	1.057090
H	7.525870	1.766664	-0.049390
H	6.937489	3.349684	-0.678130

Supporting Information: 040-Decarboxylated-Cluster-RuII-2HOAc-1OAc-CsBr-CO2-01-SP-TFE-PBE.log

----

Using Gaussian 16: ES64L-G16RevA.03 25-Dec-2016

==

# pbepbe/Def2QZVP/auto empiricaldispersion=gd3bj
scf=(maxcycle=300,direct,vshift=200,tight,yqc) density=current
scrf=(solvent=2,2,2-Trifluoroethanol,smd)

Pointgroup= C1 Stoichiometry= C16H28B10BrCsO8RuSe C1[X(C16H28B10BrCsO8RuSe)] #Atoms= 66

Charge = 0 Multiplicity = 1

SCF Energy= -6566.51951246

==

Optimization incomplete.

Atomic Type	Coordinates (Angstroms) X	Y	Z
B	1.973585	-2.537514	2.246071
B	1.601687	-1.771113	3.802741
H	2.077946	-2.152734	4.833501
H	2.722984	-3.464805	2.125731
B	-0.075172	-1.183590	3.761571
B	1.284510	-0.030703	3.544091
B	0.511666	-2.373871	1.201311
B	1.851978	-1.278234	1.038531
H	0.188674	-3.101871	0.313171
H	1.437611	0.870107	4.315911
H	-0.841272	-1.075399	4.674151
C	0.251449	-0.686351	1.000551

C	-0.118710	-0.005770	2.586381
B	0.331695	-2.702381	2.933241
B	-0.738373	-1.514149	2.111521
H	-1.920673	-1.616717	1.951051
H	-0.106947	-3.742800	3.333291
B	2.547668	-0.852675	2.588281
H	3.702049	-0.563217	2.711071
B	1.368380	0.244977	1.789261
H	1.620942	1.381407	1.235381
C	-3.306717	-3.855744	0.865791
O	-4.074215	-2.955133	0.818111
O	-2.572059	-4.771006	0.961911
C	-1.090428	1.155412	2.633441
H	-0.677667	1.968931	3.249751
H	-1.251777	1.552502	1.622031
H	-2.051019	0.836683	3.067901
Ru	0.755441	0.863228	-0.329579
Se	2.515558	-0.830355	-0.810589
C	4.201230	0.025882	-0.342549
C	5.272448	-0.772640	0.084431
C	4.337672	1.414272	-0.476239
C	6.494500	-0.165512	0.405071
H	5.148247	-1.855779	0.184401
C	5.568813	2.008860	-0.159559
H	3.489883	2.018633	-0.813839
C	6.643442	1.224318	0.283541
H	7.329618	-0.781633	0.753851
H	5.683605	3.093300	-0.259209
H	7.599373	1.695286	0.534461
O	-0.593155	2.661231	-0.208689
C	0.253266	3.347509	-0.925369
C	-0.085792	4.753510	-1.350909
H	-0.706232	4.708231	-2.264019
H	-0.662571	5.279161	-0.573529
H	0.833829	5.308538	-1.586939
O	1.335355	2.790467	-1.280619
Cs	-3.143630	-0.065445	-0.553529
Br	-0.283479	0.376400	-2.610119
H	-0.256203	-1.847440	-2.542739
O	-0.116455	-2.848920	-2.562639
C	-1.145926	-3.457108	-1.961509
C	-0.956189	-4.954559	-1.885239
H	-0.338140	-5.321820	-2.717629
H	-0.439069	-5.192669	-0.938779
H	-1.933740	-5.456997	-1.871009
O	-2.125605	-2.862856	-1.495629

O	-3.860046	2.580597	0.821361
C	-3.518464	3.675756	1.277471
O	-2.278783	4.173404	1.177931
C	-4.449902	4.609798	2.019331
H	-4.520440	5.573058	1.486151
H	-5.447033	4.157459	2.105381
H	-4.046662	4.826527	3.022831
H	-1.694854	3.527713	0.642601

Supporting Information: 040-Decarboxylated-Cluster-RuII-2HOAc-1OAc-CsBr-CO2-02-SP-TFE-PBE.log

----

Using Gaussian 16: ES64L-G16RevA.03 25-Dec-2016

==

# pbepbe/Def2QZVP/auto empiricaldispersion=gd3bj
scf=(maxcycle=300,direct,vshift=200,tight,yqc) density=current
scrf=(solvent=2,2,2-Trifluoroethanol,smd)

Pointgroup= C1 Stoichiometry= C16H28B10BrCsO8RuSe C1[X(C16H28B10BrCsO8RuSe)] #Atoms= 66

Charge = 0 Multiplicity = 1

SCF Energy= -6566.52024777

==

Optimization incomplete.

Atomic Type	Coordinates (Angstroms) X	Y	Z
B	0.831607	3.537421	-0.255920
B	0.576207	3.708821	-2.002870
H	0.713556	4.754971	-2.570150
H	1.163536	4.440411	0.458470
B	-0.715473	2.590640	-2.501750
B	1.007148	2.178101	-2.814180
B	-0.288352	2.249240	0.327890
B	1.383588	1.897181	0.000530
H	-0.730792	2.147990	1.432780
H	1.388028	2.023521	-3.937630
H	-1.494133	2.723889	-3.400060
C	0.159959	0.810530	-0.499180
C	-0.128012	1.061840	-2.217600
B	-0.819763	3.387900	-0.919610
B	-1.222792	1.636260	-1.045820
H	-2.311162	1.140359	-0.985470

H	-1.666034	4.210009	-0.712920
B	1.966687	2.737942	-1.420270
H	3.112567	3.058162	-1.547310
B	1.436218	1.023321	-1.531080
H	2.181809	-0.016498	-1.689550
C	-4.231633	3.440958	-0.148370
O	-4.529673	3.167367	-1.257990
O	-3.942983	3.738218	0.956560
C	-0.542551	-0.123510	-3.070650
H	-0.191411	0.043780	-4.101000
H	-0.082470	-1.054720	-2.701780
H	-1.641261	-0.211931	-3.102450
Ru	1.398190	-0.852939	-0.217260
Se	2.424729	0.813472	1.342270
C	4.225088	1.109903	0.662130
C	4.850078	2.343293	0.897560
C	4.887259	0.078013	-0.017220
C	6.152417	2.553564	0.423460
H	4.318037	3.138733	1.429300
C	6.193949	0.298104	-0.479370
H	4.381270	-0.877837	-0.188250
C	6.824678	1.532155	-0.264600
H	6.639797	3.519655	0.590510
H	6.717839	-0.502295	-1.012380
H	7.842048	1.699455	-0.632900
O	0.779671	-2.565069	-1.484010
C	1.904241	-3.164908	-1.242230
C	2.150202	-4.545908	-1.799840
H	1.768652	-5.299539	-1.087570
H	1.625972	-4.678429	-2.759260
H	3.229582	-4.717338	-1.928360
O	2.770971	-2.588698	-0.509680
Cs	-2.193490	-2.265281	-0.503500
Br	0.517020	-2.136849	1.810630
H	-0.702691	-0.604060	2.754630
O	-1.169661	0.127790	3.290630
C	-2.347931	0.414879	2.761230
C	-3.050442	1.563308	3.437480
H	-2.639182	1.749409	4.439270
H	-2.911433	2.467908	2.819810
H	-4.132282	1.366128	3.490440
O	-2.823771	-0.193242	1.779250
O	-4.924460	-0.899523	-0.734450
C	-5.525261	0.082917	-0.292680
O	-5.104091	0.799627	0.764010
C	-6.799331	0.635416	-0.888630

H	-6.558282	1.554646	-1.450290
H	-7.242271	-0.100604	-1.573420
H	-7.516202	0.908715	-0.098040
H	-4.237631	0.397748	1.118220

Supporting Information: 040-Decarboxylated-Cluster-RuII-2HOAc-1OAc-CsBr-CO2-03-SP-TFE-PBE.log

----

Using Gaussian 16: ES64L-G16RevA.03 25-Dec-2016

==

# pbepbe/Def2QZVP/auto empiricaldispersion=gd3bj
scf=(maxcycle=300,direct,vshift=200,tight,yqc) density=current
scrf=(solvent=2,2,2-Trifluoroethanol,smd)

Pointgroup= C1 Stoichiometry= C16H28B10BrCsO8RuSe C1[X(C16H28B10BrCsO8RuSe)]
#Atoms= 66

Charge = 0 Multiplicity = 1

SCF Energy= -6566.51410750

==

Optimization incomplete.

Atomic Type	Coordinates (Angstroms) X	Y	Z
B	2.637360	3.214930	-0.245040
B	2.622150	3.464680	-2.000420
H	3.335960	4.260290	-2.541270
H	3.359740	3.808680	0.504280
B	0.967210	3.204710	-2.597850
B	2.212580	1.925520	-2.811330
B	0.967810	2.740580	0.247640
B	2.205600	1.539510	0.016210
H	0.471620	2.909640	1.320620
H	2.519390	1.568590	-3.911070
H	0.437490	3.716730	-3.540840
C	0.623280	1.272110	-0.573560
C	0.621690	1.604970	-2.306190
B	1.206290	3.965860	-1.010060
B	-0.059100	2.699940	-1.194040
H	-1.243910	2.865030	-1.221630
H	0.903180	5.112600	-0.841750
B	3.233810	1.908680	-1.351260
H	4.377370	1.560230	-1.401220
B	1.871330	0.746290	-1.523630

H	1.942270	-0.535440	-1.650440
C	-3.516660	4.119699	-0.236900
O	-3.989740	3.251529	-0.888990
O	-3.073080	5.026709	0.368670
C	-0.315690	0.813740	-3.200210
H	0.147700	0.708360	-4.193750
H	-0.488430	-0.197420	-2.797230
H	-1.269460	1.351650	-3.331510
Ru	0.745190	-0.787630	-0.242620
Se	2.425290	0.091080	1.398930
C	4.130990	-0.650220	0.823830
C	5.312200	0.050460	1.108470
C	4.161000	-1.889930	0.170250
C	6.542680	-0.490220	0.710140
H	5.270440	1.017750	1.619380
C	5.399860	-2.424360	-0.215710
H	3.226850	-2.421000	-0.040430
C	6.587400	-1.726680	0.048500
H	7.467090	0.059330	0.915540
H	5.432110	-3.391660	-0.728000
H	7.550220	-2.146350	-0.260520
O	-0.628800	-1.896420	-1.570140
C	-0.019890	-3.009960	-1.303310
C	-0.495570	-4.300750	-1.923430
H	-1.003540	-4.898590	-1.147180
H	-1.193860	-4.104920	-2.751140
H	0.366300	-4.883840	-2.285650
O	0.965590	-2.997290	-0.495510
Cs	-3.037360	0.085719	-0.694660
Br	-0.787890	-1.327310	1.732540
H	-1.098300	0.727890	2.664770
O	-1.092250	1.601790	3.161320
C	-1.920190	2.461379	2.551430
C	-1.869760	3.836889	3.172180
H	-1.133370	4.440650	2.612270
H	-2.847630	4.329679	3.078060
H	-1.551390	3.790710	4.223640
O	-2.620450	2.167229	1.575960
O	-3.994520	-2.756101	-0.020450
C	-3.550590	-3.869131	0.261230
O	-2.391200	-4.079771	0.911170
C	-4.227100	-5.172421	-0.104270
H	-4.206040	-5.875281	0.743570
H	-5.261660	-4.980221	-0.419680
H	-3.678500	-5.647231	-0.937220
H	-1.960450	-3.193871	1.115250

Supporting Information: 042-Decarboxylated-Cluster-RuIV-H-1HOAc-2OAc-CsBr-01-SP-TFE-PBE.log

----

Using Gaussian 16: ES64L-G16RevA.03 25-Dec-2016

==

# pbepbe/Def2QZVP/auto empiricaldispersion=gd3bj
scf=(maxcycle=300,direct,vshift=200,tight,yqc) density=current
scrf=(solvent=2,2,2-Trifluoroethanol,smd)

Pointgroup= C1 Stoichiometry= C15H28B10BrCsO6RuSe C1[X(C15H28B10BrCsO6RuSe)]
#Atoms= 63

Charge = 0 Multiplicity = 1

SCF Energy= -6378.02736625

==

Optimization incomplete.

Atomic Type	Coordinates (Angstroms) X	Y	Z
B	-2.582018	3.438632	-1.028539
B	-3.107837	4.220292	0.483951
H	-3.826857	5.178383	0.513141
H	-2.900727	3.809172	-2.122689
B	-1.805137	4.025002	1.679851
B	-3.226098	2.966152	1.741851
B	-0.953938	2.777701	-0.734919
B	-2.351119	1.722462	-0.653239
H	-0.119588	2.595671	-1.567929
H	-3.928638	2.948153	2.709971
H	-1.549977	4.728591	2.613171
C	-1.135939	1.580261	0.500301
C	-1.633468	2.333861	1.913371
B	-1.394377	4.324881	-0.016549
B	-0.475778	3.117361	0.926381
H	0.650282	3.166690	1.318021
H	-0.864446	5.341301	-0.363889
B	-3.714448	2.588873	0.067351
H	-4.856268	2.350653	-0.203939
B	-2.776509	1.421772	1.023761
H	-3.139250	0.373882	1.450091
C	-1.137579	1.779241	3.238631
H	-1.817349	0.999672	3.612001
H	-0.132389	1.348641	3.110701

H	-1.081768	2.594291	3.976341
Ru	-0.250180	-0.314209	0.151751
Se	-2.026000	0.002192	-1.691609
C	-3.595930	-0.988027	-1.112019
C	-4.860040	-0.459807	-1.421259
C	-3.467201	-2.222597	-0.462269
C	-6.009980	-1.170516	-1.053239
H	-4.948299	0.503454	-1.932059
C	-4.627322	-2.923217	-0.100229
H	-2.475781	-2.627118	-0.238099
C	-5.895751	-2.401426	-0.390099
H	-6.996460	-0.755805	-1.284359
H	-4.531422	-3.885097	0.414241
H	-6.796212	-2.952825	-0.100709
O	0.883220	-0.910810	1.963111
C	-0.217251	-1.340809	2.482521
C	-0.223911	-2.112599	3.771651
H	-0.127422	-3.189399	3.544331
H	0.620589	-1.811210	4.410691
H	-1.176821	-1.960439	4.301261
O	-1.299800	-1.107589	1.842081
Cs	3.449839	-1.592042	0.386951
O	0.920160	0.168710	-1.558529
C	1.936131	0.954589	-1.470619
O	2.378521	1.419149	-0.371339
H	0.707751	0.893540	0.500881
C	2.628391	1.293539	-2.777399
H	2.596602	2.386229	-2.927419
H	3.690901	0.999288	-2.722539
H	2.141641	0.790389	-3.623949
Br	0.173609	-2.796580	-0.568839
O	5.460121	0.519237	-0.542639
C	5.656881	1.742477	-0.530789
O	4.690372	2.658198	-0.466309
H	3.775152	2.182968	-0.431969
C	7.035952	2.366196	-0.594659
H	7.201862	3.007516	0.287311
H	7.804311	1.582366	-0.639289
H	7.112692	3.016526	-1.482399

Supporting Information: 050-Product-Catalyst-Complex-1HOAc-2OAc-CsBr-01-SP-TFE-PBE.log

----

Using Gaussian 16: ES64L-G16RevA.03 25-Dec-2016

==

# pbepbe/Def2QZVP/auto empiricaldispersion=gd3bj
scf=(maxcycle=300,direct,vshift=200,tight,yqc) density=current
scrf=(solvent=2,2,2-Trifluoroethanol,smd)

Pointgroup= C1 Stoichiometry= C15H28B10BrCsO6RuSe C1[X(C15H28B10BrCsO6RuSe)]
#Atoms= 63
Charge = 0 Multiplicity = 1

SCF Energy= -6378.05033093
==
Optimization incomplete.

Atomic Type	Coordinates (Angstroms) X	Y	Z
B	4.366331	1.196361	0.477458
B	4.446300	2.176231	-1.017142
H	5.390330	2.161051	-1.753742
H	5.235221	0.447931	0.821098
B	3.541660	3.681701	-0.731342
B	2.782250	2.352450	-1.647692
B	3.413370	2.109911	1.671068
B	2.661201	0.812920	0.730888
H	3.437930	2.047681	2.864728
H	2.451870	2.545340	-2.781212
H	3.705559	4.744361	-1.254102
C	1.886770	2.297220	0.919748
C	1.933720	3.198770	-0.432572
B	4.521630	2.979741	0.583678
B	2.923720	3.645650	0.928018
H	2.581039	4.608750	1.543438
H	5.498450	3.538121	0.992398
B	3.306901	0.818681	-0.924392
H	3.410481	-0.192319	-1.551152
B	1.671320	1.422410	-0.595402
H	0.428790	1.501590	-1.070822
C	0.753120	4.103140	-0.751662
H	0.862519	4.471240	-1.783372
H	-0.205980	3.561319	-0.672762
H	0.732679	4.971030	-0.074532
Ru	0.015821	-0.034450	-0.522812
Se	1.535401	-0.729030	1.373388
C	2.715582	-2.138530	0.733858
C	3.930382	-2.337649	1.407858
C	2.332852	-2.960980	-0.332422
C	4.785442	-3.366459	0.990438
H	4.212911	-1.688469	2.242558

C	3.195882	-3.990629	-0.736272
H	1.383162	-2.788180	-0.843062
C	4.418932	-4.193809	-0.081532
H	5.739582	-3.518529	1.505408
H	2.906062	-4.634430	-1.573292
H	5.088473	-4.997049	-0.405932
O	-1.111829	0.151999	-2.447852
C	-0.217039	-0.587221	-3.010352
C	-0.348959	-1.002011	-4.453532
H	-0.921408	-1.945201	-4.513842
H	-0.884199	-0.231821	-5.030542
H	0.646381	-1.179340	-4.888342
O	0.774891	-0.981350	-2.305732
Cs	-3.864439	0.198658	-1.159562
Br	-1.307118	-2.196891	-0.044242
H	0.963210	2.347160	1.503228
O	-0.884669	0.940979	1.145458
C	-1.696240	1.953329	1.009458
C	-2.151680	2.565959	2.334818
H	-1.603940	2.144129	3.191508
H	-2.018140	3.660309	2.301768
H	-3.229810	2.368548	2.465928
O	-2.109270	2.424789	-0.075582
O	-4.116639	-0.250972	1.860178
C	-3.514839	-0.592642	2.883048
O	-2.370939	-1.284241	2.898628
H	-2.066839	-1.454451	1.952018
C	-3.979539	-0.244692	4.282048
H	-3.389329	0.612628	4.651678
H	-5.041519	0.037488	4.264578
H	-3.812039	-1.083762	4.975108

Supporting Information: 050-Product-Catalyst-Complex-1HOAc-2OAc-CsBr-02-SP-TFE-PBE.log

----

Using Gaussian 16: ES64L-G16RevA.03 25-Dec-2016

==

# pbepbe/Def2QZVP/auto empiricaldispersion=gd3bj
scf=(maxcycle=300,direct,vshift=200,tight,yqc) density=current
scrf=(solvent=2,2,2-Trifluoroethanol,smd)

Pointgroup= C1 Stoichiometry= C15H28B10BrCsO6RuSe C1[X(C15H28B10BrCsO6RuSe)]
#Atoms= 63
Charge = 0 Multiplicity = 1

SCF Energy= -6378.05034435

==

Optimization incomplete.

Atomic Type	Coordinates (Angstroms) X	Y	Z
B	4.128158	1.978513	0.150921
B	3.891097	2.964943	-1.326119
H	4.764937	3.159423	-2.121369
H	5.161879	1.439224	0.422451
B	2.694886	4.232612	-0.956659
B	2.194318	2.775781	-1.851539
B	3.078238	2.657972	1.417831
B	2.567199	1.227792	0.501481
H	3.196818	2.592252	2.605641
H	1.752837	2.892641	-2.956139
H	2.587325	5.308052	-1.467459
C	1.508148	2.509681	0.771091
C	1.259847	3.396681	-0.563369
B	3.892887	3.756483	0.280471
B	2.213426	4.052431	0.737971
H	1.707386	4.912431	1.392671
H	4.749656	4.513783	0.634571
B	3.084799	1.391432	-1.190399
H	3.369109	0.446982	-1.862899
B	1.384368	1.621961	-0.746199
H	0.178249	1.396130	-1.255249
Cs	-3.345589	-2.067973	0.367141
C	-0.131203	3.976769	-0.771589
H	-0.891243	3.339929	-0.289659
H	-0.196174	4.995509	-0.360229
H	-0.349713	4.016409	-1.849669
Ru	0.039300	-0.117530	-0.545119
O	-0.956951	1.029569	0.959761
O	0.897661	-1.440400	-2.005959
C	-1.445991	0.479908	2.045121
C	0.354272	-2.467170	-1.465769
O	-1.495260	-0.741032	2.289011
O	-0.383108	-2.286391	-0.422109
C	0.633533	-3.848430	-1.996319
H	0.739663	-3.821120	-3.091699
H	1.587933	-4.205009	-1.567529
H	-0.161697	-4.551901	-1.706019
C	-1.997791	1.490678	3.052951
H	-3.075831	1.301957	3.194001
H	-1.506411	1.338608	4.028731

H	-1.853632	2.531878	2.724251
Se	1.788420	-0.473069	1.253531
C	3.210511	-1.735368	0.821501
C	3.883552	-2.295107	1.919341
C	3.543251	-2.106788	-0.489819
C	4.907702	-3.227827	1.701881
H	3.610651	-1.999728	2.937561
C	4.568432	-3.042527	-0.694589
H	3.001351	-1.678198	-1.334789
C	5.251223	-3.602866	0.395171
H	5.436583	-3.659956	2.557671
H	4.835172	-3.329107	-1.717469
H	6.051103	-4.331096	0.226501
H	0.614038	2.351710	1.383691
Br	-1.973220	0.153718	-2.160569
O	-4.529681	0.768436	0.702871
C	-4.318932	1.965416	0.492141
O	-3.463642	2.435767	-0.425729
H	-3.008472	1.657887	-0.886779
C	-4.986093	3.084896	1.264011
H	-5.290013	3.901225	0.589871
H	-5.854362	2.694865	1.812931
H	-4.264313	3.504416	1.986841

Supporting Information: 050-Product-Catalyst-Complex-1HOAc-2OAc-CsBr-03-SP-TFE-PBE.log

----

Using Gaussian 16: ES64L-G16RevA.03 25-Dec-2016

==

\# pbepbe/Def2QZVP/auto empiricaldispersion=gd3bj
scf=(maxcycle=300,direct,vshift=200,tight,yqc) density=current
scrf=(solvent=2,2,2-Trifluoroethanol,smd)

Pointgroup= C1 Stoichiometry= C15H28B10BrCsO6RuSe C1[X(C15H28B10BrCsO6RuSe)]
\#Atoms= 63
Charge = 0 Multiplicity = 1

SCF Energy= -6378.02459373

==

Optimization incomplete.

Atomic Type	Coordinates (Angstroms) X	Y	Z
B	-2.720228	3.215479	-1.230471
B	-3.168218	4.213479	0.176239

H	-4.021558	5.052490	0.137009
H	-3.233978	3.314219	-2.307511
B	-1.713648	4.438179	1.175239
B	-2.931168	3.233719	1.640779
B	-0.989508	2.833959	-1.091651
B	-2.202339	1.625639	-0.623311
H	-0.221199	2.584198	-1.968971
H	-3.490658	3.317629	2.693849
H	-1.450188	5.338199	1.916629
C	-0.854479	1.951558	0.366259
C	-1.260968	2.866039	1.680259
B	-1.573078	4.436229	-0.598991
B	-0.365348	3.594818	0.388309
H	0.785962	3.824168	0.588809
H	-1.259238	5.419929	-1.204151
B	-3.556449	2.463709	0.161139
H	-4.669259	2.028840	0.111779
B	-2.349999	1.632559	1.157619
H	-2.452459	0.663029	1.836109
C	-0.505779	2.596208	2.973029
H	-1.210189	2.292429	3.761899
H	0.229061	1.787258	2.826699
H	0.026912	3.502908	3.297019
Ru	-0.231680	-0.568842	0.095229
Se	-2.067290	-0.181021	-1.510231
C	-3.694150	-0.990900	-0.801101
C	-4.934100	-0.537480	-1.276961
C	-3.609040	-2.050171	0.110939
C	-6.110260	-1.137290	-0.806531
H	-4.981899	0.282830	-2.000411
C	-4.793870	-2.647890	0.567029
H	-2.631450	-2.395431	0.457669
C	-6.040970	-2.192360	0.115859
H	-7.080980	-0.779229	-1.164821
H	-4.736031	-3.475940	1.281359
H	-6.961010	-2.661559	0.479519
O	1.073670	-0.917452	1.881399
C	0.029380	-1.305632	2.531089
C	0.141780	-1.831972	3.939209
H	0.297239	-2.925382	3.907979
H	0.999180	-1.374242	4.457099
H	-0.788890	-1.638802	4.494459
O	-1.108130	-1.247001	1.945489
Cs	3.415630	-1.705283	0.098269
O	0.951401	0.014648	-1.578871
C	1.856331	0.916738	-1.431811

O	2.159521	1.436747	-0.306981
H	0.088321	1.335718	0.522399
C	2.604051	1.332947	-2.687851
H	2.510311	2.423967	-2.823541
H	3.679231	1.110097	-2.567881
H	2.210411	0.815117	-3.573631
Br	0.059599	-2.962872	-0.530641
O	5.285021	0.657556	-0.433141
C	5.408971	1.879696	-0.261121
O	4.394551	2.734407	-0.168431
H	3.492671	2.216837	-0.251531
C	6.755671	2.566066	-0.141321
H	6.821832	3.104366	0.819199
H	7.565951	1.827705	-0.214451
H	6.864272	3.320056	-0.939271

17. Transition State Solvation Single Point Energies

Supporting Information: 015-Cs-Cluster-RuII-1HOAc-2OAc-Cymene-Deprotonation-TS-01-SP-TFE-PBE.log

----

Using Gaussian 16: ES64L-G16RevA.03 25-Dec-2016

==

# pbepbe/Def2QZVP/auto empiricaldispersion=gd3bj
scf=(maxcycle=300,direct,vshift=200,tight,yqc) density=current
scrf=(solvent=2,2,2-Trifluoroethanol,smd)

Pointgroup= C1 Stoichiometry= C20H37B10CsO8Ru C1[X(C20H37B10CsO8Ru)] #Atoms= 77
Charge = 0 Multiplicity = 1

SCF Energy= -1749.08437739

==

Optimization incomplete.

Atomic Type	Coordinates (Angstroms) X	Y	Z
B	3.922467	-1.387757	0.668511
B	5.118177	-1.502459	-0.647739
H	6.055645	-2.246351	-0.617189
H	3.999716	-2.041807	1.672241
B	5.253349	0.096861	-1.412939
B	4.274607	-1.166818	-2.180319
B	3.335320	0.282394	0.706501
B	2.320747	-1.003234	-0.039479
H	2.929061	0.925945	1.626181
H	1.144235	-2.138722	-0.118539
H	4.535956	-1.554368	-3.280429
H	6.183270	0.570769	-1.995509
C	2.586540	0.513805	-0.798829
C	3.701060	0.435783	-2.048369
B	5.040589	-0.019509	0.345401
B	4.154071	1.204423	-0.578679
H	4.230123	2.393563	-0.582639
H	5.898010	0.320100	1.108841
B	3.455065	-2.097766	-0.901119
H	3.200963	-3.253836	-1.058389
B	2.571748	-0.856525	-1.808109
H	1.695208	-0.935023	-2.612369
C	1.372102	1.437248	-0.820799

O	0.375171	1.004449	-0.120569
O	1.392674	2.525227	-1.433119
Cs	-2.760269	1.026165	-1.313159
C	3.519681	1.309604	-3.279289
H	3.115420	0.714594	-4.112079
H	2.836453	2.142025	-3.055989
H	4.499162	1.715212	-3.574849
Ru	0.313568	-0.904121	0.818081
O	-0.697973	-1.319579	-0.969989
C	-0.399145	-2.456209	-1.535949
O	0.542734	-3.192001	-1.130939
C	-1.245896	-2.829588	-2.730259
H	-0.935745	-2.210148	-3.591739
H	-1.088738	-3.886518	-2.987989
H	-2.313895	-2.612566	-2.524569
C	1.128327	-1.077312	2.926961
C	0.716005	-2.351711	2.418111
C	0.224479	0.018420	2.936211
H	2.172918	-0.922664	3.211421
C	-0.632105	-2.573209	1.979311
H	1.452584	-3.155143	2.320931
C	-1.145831	-0.221338	2.524931
C	-1.578753	-1.487417	2.096021
H	-1.852840	0.612303	2.504241
H	-2.603834	-1.595866	1.682491
C	0.666482	1.366329	3.479261
H	1.755682	1.443047	3.307071
C	-0.007396	2.572150	2.806541
H	0.450705	3.505099	3.176041
H	0.108704	2.541090	1.712931
H	-1.085316	2.621672	3.046071
C	0.423892	1.373679	5.005631
H	0.954620	0.546268	5.506531
H	0.777293	2.324889	5.439371
H	-0.653079	1.275201	5.230821
C	-1.078908	-3.920008	1.480051
H	-1.446739	-4.524498	2.330051
H	-1.908168	-3.815967	0.764071
H	-0.255639	-4.461590	0.991031
O	-4.086314	-1.674763	-1.770359
C	-4.168944	-1.985293	-0.540019
O	-3.994913	-1.193703	0.451341
C	-4.508597	-3.459732	-0.229519
H	-5.484997	-3.712980	-0.678219
H	-3.767848	-4.129913	-0.703529
H	-4.549397	-3.659542	0.853571

O	-1.896204	3.944693	-0.837839
C	-1.144333	4.695042	-0.212359
O	0.190707	4.579400	-0.192129
H	0.474046	3.778819	-0.745149
C	-1.621241	5.846973	0.647821
H	-1.054369	6.763472	0.417181
H	-2.695710	6.013555	0.491091
H	-1.437901	5.611002	1.710981

Supporting Information: 015-Cs-Cluster-RuII-1HOAc-2OAc-PhSeBr-01-Deprotonation-TS-01-SP-TFE-PBE.log

----

Using Gaussian 16: ES64L-G16RevA.03 25-Dec-2016

===

# pbepbe/Def2QZVP/auto empiricaldispersion=gd3bj
scf=(maxcycle=300,direct,vshift=200,tight,yqc) density=current
scrf=(solvent=2,2,2-Trifluoroethanol,smd)

Pointgroup= C1 Stoichiometry= C16H28B10BrCsO8RuSe C1[X(C16H28B10BrCsO8RuSe)] #Atoms= 66

Charge = 0 Multiplicity = 1

SCF Energy= -6566.50452081

===

Optimization incomplete.

Atomic Type	Coordinates (Angstroms) X	Y	Z
B	-2.509270	2.598900	0.037309
B	-2.345760	4.287190	0.587699
H	-3.275920	4.940810	0.965499
H	-3.575230	2.061270	-0.007011
B	-1.013950	5.044210	-0.311241
B	-0.745910	4.459340	1.343209
B	-1.267840	2.334170	-1.193691
B	-1.018150	1.668630	0.471709
H	-1.275710	1.604070	-2.145021
H	-1.201770	0.455530	1.845129
H	-0.400170	5.213000	2.205899
H	-0.848759	6.199470	-0.573221
C	0.204150	2.564510	-0.401161
C	0.380540	4.150620	0.105849
B	-2.090190	3.897570	-1.134381
B	-0.343640	3.840930	-1.420511

H	0.302920	4.076980	-2.393591
H	-2.823820	4.243210	-2.015771
B	-1.676590	2.956300	1.568129
H	-2.119310	2.696250	2.645569
B	0.059440	2.882640	1.257649
H	0.985910	2.599300	1.955379
C	1.394160	1.718919	-0.827931
O	1.282960	0.473619	-0.498131
O	2.361520	2.218139	-1.443441
Cs	3.372230	-1.342681	0.891689
C	1.761030	4.787339	0.121969
H	2.175920	4.774159	1.141379
H	2.436800	4.247529	-0.557441
H	1.673131	5.832659	-0.211291
Ru	-0.286770	-0.387670	0.617649
O	0.920320	-0.036520	2.389839
O	-1.382730	-1.865340	1.705919
C	0.166260	0.291090	3.385769
C	-0.518311	-2.804590	1.513279
O	-1.076850	0.524710	3.271569
O	0.564410	-2.536730	0.884809
C	-0.833011	-4.196560	1.989279
H	0.089679	-4.737880	2.250029
H	-1.522591	-4.170920	2.846459
H	-1.325551	-4.730410	1.156439
C	0.811350	0.456570	4.744909
H	1.033320	1.527220	4.901279
H	0.117810	0.138910	5.537839
H	1.752890	-0.109421	4.813569
Se	-1.484730	-0.855830	-1.353391
Br	-1.705401	-3.350290	-1.597031
C	-3.365820	-0.549060	-1.045531
C	-4.111560	-0.048710	-2.124081
C	-3.947820	-0.760310	0.213959
C	-5.462380	0.270470	-1.929551
H	-3.635150	0.109210	-3.096631
C	-5.302280	-0.454170	0.389229
H	-3.341180	-1.149770	1.035379
C	-6.055840	0.067830	-0.675181
H	-6.048410	0.680890	-2.757921
H	-5.767640	-0.608890	1.367969
H	-7.110340	0.320810	-0.524711
O	5.047580	-0.192561	-1.319801
C	5.071520	0.228309	-2.482711
O	4.189280	1.084769	-2.995261
C	6.121410	-0.177381	-3.497671

H	6.814610	-0.905401	-3.054491
H	5.637900	-0.610751	-4.389221
H	6.680410	0.711649	-3.835361
H	3.512350	1.374509	-2.290301

Supporting Information: 015-Cs-Cluster-RuII-1HOAc-2OAc-PhSeBr-01-Deprotonation-TS-02-SP-TFE-PBE.log

----

Using Gaussian 16: ES64L-G16RevA.03 25-Dec-2016

===

# pbepbe/Def2QZVP/auto empiricaldispersion=gd3bj
scf=(maxcycle=300,direct,vshift=200,tight,yqc) density=current
scrf=(solvent=2,2,2-Trifluoroethanol,smd)

Pointgroup= C1 Stoichiometry= C16H28B10BrCsO8RuSe C1[X(C16H28B10BrCsO8RuSe)]
#Atoms= 66
Charge = 0 Multiplicity = 1

SCF Energy= -6566.50231912

===

Optimization incomplete.

Atomic Type	Coordinates (Angstroms) X	Y	Z
B	-2.653919	2.454011	0.160901
B	-2.613268	4.171201	0.650811
H	-3.577538	4.720991	1.101511
H	-3.655189	1.806451	0.230291
B	-1.422508	5.034530	-0.369649
B	-0.992288	4.521900	1.275201
B	-1.474279	2.280051	-1.143439
B	-1.084279	1.649570	0.506131
H	-1.477609	1.507911	-2.062929
H	-1.140360	0.461380	1.909721
H	-0.686748	5.221120	2.197071
H	-1.514308	6.194001	-0.655009
C	0.020111	2.678570	-0.465539
B	-2.446339	3.750151	-1.068359
B	-0.719438	3.847680	-1.488819
H	-0.219608	4.032360	-2.557219
H	-3.287028	3.964621	-1.894739
B	-1.746299	2.937311	1.599731
H	-1.970499	2.654631	2.736351
C	1.263811	1.889010	-0.864929

O	1.283980	0.657290	-0.446639
O	2.164391	2.435919	-1.527409
Cs	3.441300	-1.229611	0.710981
Ru	-0.235970	-0.337960	0.621961
O	0.950520	-0.195520	2.450571
O	-1.280821	-1.895150	1.655611
C	0.181630	0.077930	3.448741
C	-0.390311	-2.799660	1.417851
O	-1.029430	0.445200	3.314131
O	0.666909	-2.480820	0.771421
C	-0.644941	-4.212900	1.867481
H	0.302138	-4.725530	2.096731
H	-1.316641	-4.232570	2.738901
H	-1.134022	-4.746830	1.032811
C	0.771030	0.009840	4.840691
H	1.294550	0.958640	5.057001
H	-0.022010	-0.124920	5.589891
H	1.506070	-0.807041	4.912201
Se	-1.399600	-0.811190	-1.389259
Br	-1.473571	-3.296439	-1.684519
C	-3.302970	-0.640409	-1.098219
C	-4.065450	-0.124499	-2.156789
C	-3.886570	-0.982419	0.130781
C	-5.439480	0.078482	-1.969599
H	-3.587530	0.137551	-3.105659
C	-5.263990	-0.794468	0.295901
H	-3.264580	-1.378999	0.937311
C	-6.037170	-0.257058	-0.746089
H	-6.040560	0.501732	-2.780419
H	-5.732400	-1.053028	1.250961
H	-7.110410	-0.095877	-0.602419
O	5.060930	0.257378	-1.320429
C	5.127250	0.765978	-2.446779
O	4.200571	1.562658	-2.973199
C	6.294410	0.554448	-3.390359
H	6.986290	-0.191813	-2.976119
H	5.932660	0.231288	-4.380319
H	6.828501	1.508738	-3.538289
H	3.448651	1.742919	-2.307969
C	-0.124949	3.064660	1.111981
C	1.043931	2.828770	2.056311
H	0.653431	2.776040	3.083761
H	1.553871	1.882689	1.832581
H	1.760531	3.661199	1.994641
B	0.166112	4.338410	-0.051909
H	1.261002	4.809660	-0.051829

Supporting Information: 017-Cs-Cluster-RuII-1HOAc-2OAc-PhSeBr-Decarboxylation-TS-01-SP-TFE-PBE.log

----

Using Gaussian 16: ES64L-G16RevA.03 25-Dec-2016

==

# pbepbe/Def2QZVP/auto empiricaldispersion=gd3bj
scf=(maxcycle=300,direct,vshift=200,tight,yqc) density=current
scrf=(solvent=2,2,2-Trifluoroethanol,smd)

Pointgroup= C1 Stoichiometry= C16H28B10BrCsO8RuSe C1[X(C16H28B10BrCsO8RuSe)]
#Atoms= 66
Charge = 0 Multiplicity = 1

SCF Energy= -6566.43720965

==

Optimization incomplete.

Atomic Type	X	Y	Z
B	3.934942	1.189770	2.286250
B	5.460820	0.490976	1.707100
H	6.537901	0.854373	2.086100
H	3.864895	2.076170	3.090250
B	5.218709	-0.016014	0.021860
B	5.159676	-1.215413	1.318470
B	2.780602	1.107703	0.957180
B	2.683329	-0.089087	2.243980
H	1.938974	1.932355	0.766440
H	1.715549	-0.176244	2.941670
H	5.923693	-2.135795	1.328380
H	6.029039	-0.116026	-0.851910
C	2.515657	-0.579056	0.605620
C	3.992926	-1.214030	0.059530
B	4.462363	1.471049	0.597080
B	3.545730	0.356411	-0.457390
H	3.222330	0.508762	-1.593040
H	4.772276	2.548098	0.173750
B	4.358248	-0.480121	2.725070
H	4.613247	-0.829112	3.842070
B	3.463665	-1.575229	1.668890
H	3.089612	-2.675278	1.917350
C	4.040973	-2.297380	-1.009840
H	3.918140	-3.295420	-0.570560

H	3.250073	-2.130878	-1.753710
H	5.023683	-2.231173	-1.502420
Ru	0.388069	-0.046200	-0.806200
C	0.550403	-2.289791	-2.778320
O	-0.567978	-2.670718	-2.384150
O	1.200506	-1.235413	-2.345030
C	1.323591	-3.078343	-3.831050
H	2.004392	-2.433405	-4.407450
H	1.931889	-3.841395	-3.312480
H	0.624149	-3.598751	-4.503140
Cs	-2.793537	-2.285412	-0.458190
O	0.605403	1.385209	-2.340730
C	-0.577508	1.133212	-2.759360
C	-1.140456	1.751764	-4.000670
H	-2.231277	1.614497	-4.040880
H	-0.888513	2.823843	-4.022670
H	-0.684117	1.273802	-4.885030
O	-1.264620	0.292804	-2.045670
C	1.108654	-1.884952	0.315170
O	1.539991	-2.969804	-0.001020
O	-0.015485	-1.475049	0.790930
Se	-0.390417	1.475772	0.866330
Br	-2.902338	1.177478	1.124280
C	-0.526692	3.219532	0.018500
C	0.405690	4.192230	0.413240
C	-1.499201	3.507205	-0.951260
C	0.386534	5.451530	-0.204030
H	1.146120	3.972088	1.187730
C	-1.517888	4.773715	-1.548580
H	-2.245403	2.752947	-1.213930
C	-0.570565	5.743752	-1.184950
H	1.119076	6.207158	0.096230
H	-2.281147	5.004077	-2.299530
H	-0.587413	6.730972	-1.657240
O	-2.669237	-2.186022	2.622560
C	-2.165865	-1.413364	3.438900
O	-0.907293	-0.942407	3.346440
H	-0.515394	-1.258338	2.482940
C	-2.888043	-0.844592	4.636860
H	-3.767415	-1.459559	4.873070
H	-2.218393	-0.770433	5.507590
H	-3.220420	0.177539	4.381150

Supporting Information: 025-Cs-Deprotonated-Cluster-RuII-2HOAc-1OAc-PhSeBr-03-Selenylation-TS-02-SP-TFE-PBE.log

----

Using Gaussian 16: ES64L-G16RevA.03 25-Dec-2016

==
 # pbepbe/Def2QZVP/auto empiricaldispersion=gd3bj
 scf=(maxcycle=300,direct,vshift=200,tight,yqc) density=current
 scrf=(solvent=2,2,2-Trifluoroethanol,smd)

 Pointgroup= C1   Stoichiometry= C16H28B10BrCsO8RuSe   C1[X(C16H28B10BrCsO8RuSe)]
#Atoms= 66
 Charge = 0   Multiplicity = 1

 SCF Energy= -6566.48009815
==
 Optimization incomplete.

     Atomic   Coordinates (Angstroms)
      Type   X   Y   Z

B	2.399850	2.955560	0.533551
B	2.453329	4.421040	-0.482499
H	3.444119	5.075470	-0.636019
H	3.346000	2.535890	1.135901
B	0.860209	5.194440	-0.414849
B	1.298179	4.190130	-1.815679
B	0.759480	2.821390	1.236461
B	1.212030	1.826080	-0.173949
H	0.393350	2.446860	2.306761
H	1.695470	-2.141390	2.235431
H	1.327369	4.655480	-2.916729
H	0.587169	6.350150	-0.555609
C	-0.286980	2.689850	-0.132589
C	-0.241741	4.070980	-1.074759
B	1.525419	4.427620	1.040591
B	-0.189451	4.221480	0.634031
H	-1.171001	4.619849	1.179611
H	1.829159	5.067580	2.005581
B	2.256140	2.807310	-1.232099
H	3.080410	2.295670	-1.932439
B	0.527770	2.610020	-1.623659
H	-0.012760	2.043030	-2.523909
C	-1.583190	1.870219	-0.005739
O	-1.431750	0.654869	0.394981
O	-2.671260	2.410639	-0.304199
Cs	-2.669360	-1.810001	-0.950799
C	-1.479921	4.545569	-1.819749
H	-1.848231	3.771329	-2.507699

H	-2.288221	4.798469	-1.121729
H	-1.200141	5.437549	-2.401639
Ru	0.416060	-0.068970	1.165501
O	-0.591980	-1.876080	1.646611
O	0.319910	0.414060	3.402391
C	-0.001239	-2.961150	1.902301
C	1.474180	-0.083490	3.531241
O	1.273741	-3.070430	2.216001
O	2.058980	-0.586030	2.464141
C	2.219950	-0.104560	4.837441
H	2.592070	-1.121250	5.048381
H	3.099540	0.559200	4.767431
H	1.569550	0.238170	5.654511
C	-0.730449	-4.271320	1.800351
H	-0.544199	-4.667580	0.784561
H	-0.341089	-4.996640	2.530201
H	-1.810889	-4.126171	1.949601
Se	1.181730	-0.304270	-1.010229
C	3.097970	-0.635970	-1.091539
C	3.602270	-1.127700	-2.306229
C	3.947700	-0.338600	-0.017869
C	4.981330	-1.327540	-2.442269
H	2.916990	-1.371960	-3.123339
C	5.327710	-0.536940	-0.169179
H	3.533630	0.035870	0.920551
C	5.844890	-1.029419	-1.376219
H	5.381990	-1.716640	-3.383929
H	5.999910	-0.301809	0.662621
H	6.923390	-1.180949	-1.488379
Br	0.629851	-3.033130	-1.634499
O	-5.167090	-0.188121	-0.492879
C	-5.720240	0.711889	0.149511
O	-5.129670	1.846919	0.523761
H	-4.170320	1.883219	0.183011
C	-7.165130	0.650058	0.602551
H	-7.608000	-0.315212	0.321451
H	-7.226700	0.791308	1.694541
H	-7.738500	1.470788	0.139071

Supporting Information: 035-Selenylated-Cluster-RuII-1OAc-2HOAc-Cymene-CsBr-Decarboxylation-TS-01-SP-TFE-PBE.log

----

Using Gaussian 16: ES64L-G16RevA.03 25-Dec-2016

==

# pbepbe/Def2QZVP/auto empiricaldispersion=gd3bj
scf=(maxcycle=300,direct,vshift=200,tight,yqc) density=current
scrf=(solvent=2,2,2-Trifluoroethanol,smd)

Pointgroup= C1 Stoichiometry= C26H42B10BrCsO8RuSe C1[X(C26H42B10BrCsO8RuSe)]
#Atoms= 90
Charge = 0 Multiplicity = 1

SCF Energy= -6955.64369032
==
Optimization incomplete.

Atomic Type	Coordinates (Angstroms) X	Y	Z
B	0.253629	-0.562980	3.577030
B	-0.874410	-1.583531	4.509620
H	-0.984510	-1.504381	5.698820
H	0.960989	0.260830	4.071690
B	-1.016689	-3.155881	3.692360
B	-2.302540	-1.939462	3.500900
B	0.773330	-1.505920	2.150560
B	-0.493341	-0.303721	1.997130
H	1.781110	-1.420099	1.525060
H	-3.426030	-2.170042	3.840710
H	-1.264858	-4.221261	4.173800
C	-0.657110	-1.855491	1.246460
C	-1.699459	-2.829001	2.172420
B	0.550091	-2.318160	3.714770
B	-0.010469	-3.111390	2.221990
H	0.408952	-4.087760	1.685290
H	1.482141	-2.781819	4.307320
B	-1.522871	-0.332691	3.426840
H	-2.100171	0.640488	3.809320
B	-2.022200	-1.164142	1.932140
H	-3.066820	-0.824712	1.288280
C	-0.113629	-2.290280	-0.520510
O	-0.541089	-3.391611	-0.838880
O	0.645040	-1.405600	-0.904590
C	-2.572709	-3.838552	1.450320
H	-2.639448	-4.753302	2.058800
H	-3.586799	-3.435042	1.306260
H	-2.127438	-4.081442	0.474610
Ru	-2.324001	-0.553722	-0.357670
Se	-0.509061	1.040369	0.493950
C	-1.514652	2.552509	1.191100
C	-0.960013	3.814419	0.931050

C	-2.773392	2.427478	1.794310
C	-1.674444	4.962359	1.301180
H	0.019287	3.890570	0.454220
C	-3.474323	3.583138	2.169200
H	-3.216342	1.443318	1.956790
C	-2.927204	4.850838	1.921600
H	-1.242914	5.948249	1.101580
H	-4.452543	3.487567	2.651910
H	-3.478924	5.751198	2.210000
C	-4.247721	0.153307	-1.078400
C	-4.307180	-1.267313	-1.342980
C	-3.261821	1.001118	-1.686130
H	-5.008521	0.609417	-0.436740
C	-3.305080	-1.902132	-2.106980
H	-5.117590	-1.854413	-0.903550
C	-2.177201	0.342068	-2.366100
C	-2.180920	-1.077022	-2.500910
H	-1.300901	0.951869	-2.642810
H	-1.302410	-1.563081	-2.935480
C	-3.320069	-3.370452	-2.483190
H	-2.342439	-3.781462	-2.169810
C	-3.413679	-3.486202	-4.023000
H	-2.594309	-2.946672	-4.526930
H	-3.355808	-4.546302	-4.322900
H	-4.371149	-3.076643	-4.391150
C	-4.438398	-4.180873	-1.817140
H	-4.394508	-4.123213	-0.717150
H	-5.437239	-3.838864	-2.144360
H	-4.346448	-5.242703	-2.099020
C	-3.294632	2.494958	-1.582380
H	-3.855343	2.889337	-2.452150
H	-3.796803	2.839557	-0.665280
H	-2.261093	2.885148	-1.632060
Br	2.059128	2.345431	1.674750
Cs	2.760539	0.942051	-1.653620
O	-0.163262	2.474890	-2.071910
C	0.444297	3.427210	-2.696770
O	1.583607	3.340201	-3.226350
C	-0.309274	4.767089	-2.800630
H	-1.187464	4.644349	-3.460640
H	-0.690884	5.070149	-1.809670
H	0.335856	5.558600	-3.211060
O	4.255570	-0.657858	0.840330
C	4.317030	-0.811028	2.075990
C	4.937520	-2.019347	2.737600
H	5.628681	-2.529817	2.051930

H	5.451370	-1.733217	3.667780
H	4.125931	-2.718698	3.008930
O	3.806269	0.044462	2.946470
H	3.287809	0.784382	2.459360
O	4.697990	-1.408048	-2.425400
C	5.221541	-2.410937	-1.938850
O	5.306001	-2.650447	-0.619970
H	4.882530	-1.873297	-0.119050
C	5.844591	-3.523807	-2.756380
H	5.332232	-4.477847	-2.545480
H	5.768981	-3.288307	-3.826530
H	6.902791	-3.655176	-2.474020

Supporting Information: 035-Selenylated-Cluster-RuII-2HOAc-1OAc-CsBr-Decarboxylation-TS-01-SP-TFE-PBE.log

----

Using Gaussian 16: ES64L-G16RevA.03 25-Dec-2016

==

# pbepbe/Def2QZVP/auto empiricaldispersion=gd3bj
scf=(maxcycle=300,direct,vshift=200,tight,yqc) density=current
scrf=(solvent=2,2,2-Trifluoroethanol,smd)

Pointgroup= C1 Stoichiometry= C16H28B10BrCsO8RuSe C1[X(C16H28B10BrCsO8RuSe)]
#Atoms= 66
Charge = 0 Multiplicity = 1

SCF Energy= -6566.48398529

==

Optimization incomplete.

Atomic Type	Coordinates (Angstroms) X	Y	Z
B	-3.685901	-0.265781	2.716170
B	-3.557391	-2.021561	2.998870
H	-4.480050	-2.684972	3.376890
H	-4.699061	0.354378	2.873040
B	-1.902121	-2.374211	3.550850
B	-2.362320	-2.700721	1.863270
B	-2.092661	0.460279	3.070650
B	-2.554181	0.115899	1.418610
H	-1.859292	1.570699	3.433230
H	-2.303380	-3.808481	1.415330
H	-1.533910	-3.265571	4.257660
C	-0.981811	-0.276881	1.963410

C	-0.883991	-1.947651	2.262950
B	-2.696231	-0.876521	4.072750
B	-0.981371	-0.843981	3.577050
H	-0.008601	-0.681780	4.246020
H	-2.979881	-0.701851	5.222880
B	-3.459621	-1.398281	1.337180
H	-4.281721	-1.587872	0.490030
B	-1.733581	-1.351581	0.893860
H	-1.406211	-1.710671	-0.317630
C	0.521109	0.848070	1.801850
O	1.562229	0.204190	1.950320
O	0.111418	1.995480	1.717980
C	0.441370	-2.648780	2.019130
H	0.658030	-3.311390	2.871330
H	0.399150	-3.252350	1.100410
H	1.239069	-1.896820	1.930560
Ru	-0.526341	-0.232061	-0.593080
Se	-2.378841	1.368959	-0.162690
C	-3.942331	0.751839	-1.144280
C	-5.211621	1.128108	-0.680080
C	-3.791411	-0.007521	-2.311240
C	-6.349281	0.712168	-1.384740
H	-5.312852	1.719428	0.235700
C	-4.938141	-0.410892	-3.012300
H	-2.793461	-0.290111	-2.656830
C	-6.213881	-0.056962	-2.550400
H	-7.343201	0.988488	-1.018300
H	-4.829361	-1.009112	-3.923040
H	-7.104711	-0.380332	-3.098330
O	1.094849	-1.622680	-1.358030
C	0.495409	-1.593820	-2.502540
C	1.029159	-2.329030	-3.698580
H	1.810889	-1.714680	-4.180150
H	1.483210	-3.284050	-3.389240
H	0.227640	-2.504950	-4.430820
O	-0.574151	-0.892311	-2.585560
Cs	3.791109	0.273520	-0.121240
Br	0.800158	1.715620	-1.604170
H	1.287778	3.402520	-0.056160
O	1.539468	4.266430	0.385080
C	2.812058	4.194020	0.795560
C	3.215818	5.427270	1.575620
H	2.828687	6.340070	1.095890
H	2.772358	5.373660	2.585490
H	4.309937	5.472601	1.665920
O	3.562918	3.238680	0.584560

O	4.125610	-2.809649	-0.291190
C	3.509460	-3.874290	-0.358700
O	2.225550	-3.969690	-0.750860
C	4.093480	-5.222069	0.000440
H	3.911440	-5.949189	-0.807780
H	5.170730	-5.125789	0.191380
H	3.593010	-5.611460	0.903980
H	1.878250	-3.037420	-0.947480

Supporting Information: 035-Selenylated-Cluster-RuII-2HOAc-1OAc-CsBr-Decarboxylation-TS-02-SP-TFE-PBE.log

----

Using Gaussian 16: ES64L-G16RevA.03 25-Dec-2016

==

# pbepbe/Def2QZVP/auto empiricaldispersion=gd3bj
scf=(maxcycle=300,direct,vshift=200,tight,yqc) density=current
scrf=(solvent=2,2,2-Trifluoroethanol,smd)

Pointgroup= C1 Stoichiometry= C16H28B10BrCsO8RuSe C1[X(C16H28B10BrCsO8RuSe)]
#Atoms= 66
Charge = 0 Multiplicity = 1

SCF Energy= -6566.48497212

==

Optimization incomplete.

Atomic Type	Coordinates (Angstroms) X	Y	Z
B	3.467040	3.118519	0.218910
B	3.674430	3.451469	-1.521150
H	4.640870	4.003229	-1.963970
H	4.280310	3.410969	1.048940
B	2.064020	3.743009	-2.225640
B	2.863030	2.166009	-2.452430
B	1.716790	3.174639	0.575870
B	2.514350	1.639329	0.331610
H	1.190450	3.439429	1.611650
H	3.121740	1.770769	-3.551670
H	1.789740	4.417069	-3.174420
C	0.993670	1.936249	-0.406060
C	1.209980	2.290059	-2.036550
B	2.420630	4.322359	-0.585290
B	0.849400	3.560599	-0.932640
H	-0.233020	4.056129	-0.985400

H	2.465070	5.494779	-0.345490
B	3.728330	1.771299	-0.944190
H	4.703610	1.079539	-0.953060
B	2.135570	1.050509	-1.279950
H	2.094240	-0.181021	-1.716180
C	-0.726660	1.518739	0.325280
O	-1.562700	1.524179	-0.574930
O	-0.603010	1.483069	1.537780
C	0.140920	1.856789	-3.027250
H	0.414560	2.243209	-4.021290
H	0.079870	0.757309	-3.068770
H	-0.839480	2.251729	-2.730340
Ru	0.974930	-0.651971	-0.478720
Se	2.335960	0.001249	1.507130
C	4.135980	-0.691591	1.248790
C	5.196000	-0.062851	1.918830
C	4.350510	-1.818551	0.445480
C	6.497020	-0.558641	1.759140
H	5.011500	0.819369	2.540250
C	5.656810	-2.310281	0.300490
H	3.510590	-2.293091	-0.069200
C	6.728460	-1.681991	0.950930
H	7.330850	-0.062521	2.266260
H	5.834210	-3.188141	-0.329630
H	7.745990	-2.066751	0.828390
O	-0.161040	-1.568781	-2.197790
C	0.590350	-2.599861	-2.061560
C	0.414460	-3.839961	-2.894370
H	-0.279390	-4.528281	-2.379060
H	-0.006650	-3.584431	-3.878790
H	1.377030	-4.359931	-3.014930
O	1.505250	-2.563651	-1.153900
Cs	-3.183150	-0.964331	-1.187110
Br	-0.554050	-1.995121	1.104740
H	-1.613670	-0.766821	2.612700
O	-2.054420	-0.245711	3.360010
C	-3.172100	0.306319	2.917370
C	-3.741490	1.325389	3.872130
H	-3.530090	1.047589	4.915400
H	-3.235750	2.287149	3.672020
H	-4.821570	1.455719	3.713610
O	-3.685050	0.030969	1.814100
O	-6.002650	0.138119	-0.656260
C	-6.564130	0.870879	0.163070
O	-6.040400	1.211199	1.347920
C	-7.926260	1.494599	-0.056290

H	-7.845400	2.594369	-0.023570
H	-8.331580	1.179439	-1.027260
H	-8.613990	1.197349	0.753170
H	-5.131560	0.761799	1.459530

Supporting Information: 035-Selenylated-Cluster-RuII-2HOAc-1OAc-CsBr-Decarboxylation-TS-03-SP-TFE-PBE.log

----

Using Gaussian 16: ES64L-G16RevA.03 25-Dec-2016

==

pbepbe/Def2QZVP/auto empiricaldispersion=gd3bj scf=(maxcycle=300,direct,vshift=200,tight,yqc) density=current scrf=(solvent=2,2,2-Trifluoroethanol,smd)

Pointgroup= C1 Stoichiometry= C16H28B10BrCsO8RuSe C1[X(C16H28B10BrCsO8RuSe)] #Atoms= 66

Charge = 0 Multiplicity = 1

SCF Energy= -6566.48756899

==

Optimization incomplete.

Atomic Type	Coordinates (Angstroms) X	Y	Z
B	-4.326777	-2.070495	0.148990
B	-4.626097	-2.254876	-1.597620
H	-5.724297	-2.392167	-2.055220
H	-5.196997	-2.058056	0.972460
B	-3.236336	-3.112544	-2.311710
B	-3.382768	-1.343644	-2.494470
B	-2.726976	-2.787094	0.504710
B	-2.892498	-1.060494	0.306140
H	-2.350256	-3.257853	1.532400
H	-3.464118	-0.853774	-3.582820
H	-3.227305	-3.817564	-3.277300
C	-1.581427	-1.886722	-0.444890
C	-1.902827	-2.096443	-2.088080
B	-3.799255	-3.556405	-0.686930
B	-2.053375	-3.433213	-1.013020
H	-1.240164	-4.301452	-1.082470
H	-4.282984	-4.631845	-0.479690
B	-4.049578	-0.691185	-0.974640
H	-4.692029	0.317294	-0.963210
B	-2.299238	-0.621343	-1.293790

H	-1.745610	0.504157	-1.641280
C	0.218453	-2.252020	0.248960
O	0.970104	-2.504100	-0.678990
O	0.097883	-2.324681	1.465340
C	-0.746367	-2.074141	-3.074860
H	-1.164897	-2.146272	-4.090940
H	-0.176468	-1.136491	-2.982730
H	-0.067626	-2.918501	-2.896410
Ru	-0.545200	0.361699	-0.368860
Se	-2.122249	0.321437	1.560230
C	-3.446391	1.716486	1.261110
C	-4.698951	1.594174	1.880410
C	-3.128662	2.832246	0.476230
C	-5.658632	2.597113	1.687070
H	-4.931490	0.713944	2.488350
C	-4.095453	3.833555	0.297570
H	-2.144872	2.908717	0.004250
C	-5.358043	3.716544	0.896390
H	-6.644042	2.499982	2.154050
H	-3.857154	4.707715	-0.317410
H	-6.109974	4.498133	0.747600
O	0.900970	0.740900	-2.046620
C	0.696059	1.995270	-1.869340
C	1.335518	3.039791	-2.742050
H	1.196597	4.038721	-2.303820
H	2.409918	2.822452	-2.852930
H	0.875618	3.015090	-3.745530
O	-0.108602	2.344839	-0.924240
Cs	3.529712	-0.847677	-1.061300
Br	1.323380	0.718831	1.360440
H	1.507993	-2.335329	2.499260
O	2.151673	-2.360778	3.271230
C	3.408143	-2.293577	2.818740
C	4.407843	-2.243266	3.956280
H	4.367042	-1.246546	4.429870
H	4.155284	-2.983466	4.732120
H	5.422464	-2.420205	3.573350
O	3.722923	-2.258217	1.626600
O	4.107989	2.189734	-0.865770
C	3.793767	3.280853	-0.380770
O	2.756967	3.476362	0.443540
C	4.520876	4.575084	-0.681010
H	4.726455	5.129614	0.248590
H	5.456456	4.364165	-1.216710
H	3.879285	5.219374	-1.307690
H	2.297768	2.594002	0.634380

Supporting Information: 035-Selenylated-Cluster-RuII-2HOAc-1OAc-CsBr-Decarboxylation-TS-04-SP-TFE-PBE.log

----

Using Gaussian 16: ES64L-G16RevA.03 25-Dec-2016

==

pbepbe/Def2QZVP/auto empiricaldispersion=gd3bj scf=(maxcycle=300,direct,vshift=200,tight,yqc) density=current scrf=(solvent=2,2,2-Trifluoroethanol,smd)

Pointgroup= C1 Stoichiometry= C16H28B10BrCsO8RuSe C1[X(C16H28B10BrCsO8RuSe)] #Atoms= 66

Charge = 0 Multiplicity = 1

SCF Energy= -6566.42655868

==

Optimization incomplete.

Atomic Type	Coordinates (Angstroms) X	Y	Z
B	3.568728	1.393333	2.542029
B	2.308489	1.194421	3.785829
H	2.233298	1.895161	4.753939
H	4.420468	2.233233	2.583069
B	1.883730	-0.530469	3.868599
B	0.838809	0.598150	2.982069
B	3.918160	-0.204477	1.846439
B	2.868149	0.906772	0.971329
H	4.940150	-0.582466	1.365769
H	2.897680	-0.284238	-2.133771
H	-0.306521	0.739499	3.300939
H	1.454121	-1.146870	4.797239
C	2.435791	-0.760239	1.130869
C	1.272601	-0.919350	2.327809
B	3.575020	-0.050747	3.587659
B	2.877831	-1.398138	2.670089
H	3.113232	-2.561548	2.748909
H	4.424450	-0.279067	4.398949
B	1.876448	1.787171	2.159859
H	1.480027	2.895671	1.932309
B	1.199519	0.435600	1.250099
H	0.361699	0.506939	0.411879
C	2.923062	-2.118908	-0.046301
O	2.298283	-3.192469	0.242069

O	3.894892	-1.787177	-0.705431
C	0.260162	-2.015281	2.069859
H	-0.569018	-1.962242	2.798879
H	-0.417288	-1.808701	1.163159
H	0.704283	-3.018650	2.078779
Ru	0.703242	-1.899250	-0.383541
O	1.233392	-2.135400	-2.370161
O	-1.033679	-0.863852	-1.117931
C	2.008432	-1.651659	-3.228991
C	-1.604788	-2.003903	-1.324661
O	2.829461	-0.631618	-3.081371
O	-0.890057	-3.047642	-1.060031
C	-3.020938	-2.125194	-1.777961
H	-3.647068	-2.192445	-0.868771
H	-3.322649	-1.229644	-2.342231
H	-3.159217	-3.037434	-2.377761
C	2.055332	-2.270939	-4.598741
H	2.151281	-1.491129	-5.370141
H	2.949873	-2.915938	-4.661221
H	1.160853	-2.885140	-4.768171
Se	3.388418	1.588492	-0.831661
C	1.821937	2.648621	-1.256871
C	1.674506	3.935721	-0.709071
C	0.873298	2.171350	-2.179531
C	0.578485	4.733910	-1.071561
H	2.422366	4.310231	-0.004651
C	-0.213033	2.979199	-2.554101
H	0.981769	1.172570	-2.609341
C	-0.364494	4.261529	-2.000281
H	0.476704	5.737489	-0.645131
H	-0.935883	2.605118	-3.287771
H	-1.201015	4.898738	-2.307841
Br	-3.188969	-1.255844	1.894259
Cs	-2.263662	1.834677	0.550109
O	-4.832011	0.975614	-0.823651
C	-5.749060	0.150273	-0.737591
O	-5.785249	-0.867687	0.124239
H	-4.934869	-0.891686	0.702249
C	-6.976780	0.175092	-1.627431
H	-7.892800	0.175301	-1.013801
H	-6.955001	1.062372	-2.275001
H	-7.006199	-0.737098	-2.247901

Supporting Information: 035-Selenylated-Cluster-RuII-2HOAc-1OAc-CsBr-Decarboxylation-TS-05-SP-TFE-PBE.log

----

Using Gaussian 16: ES64L-G16RevA.03 25-Dec-2016

==
 # pbepbe/Def2QZVP/auto empiricaldispersion=gd3bj
 scf=(maxcycle=300,direct,vshift=200,tight,yqc) density=current
 scrf=(solvent=2,2,2-Trifluoroethanol,smd)

 Pointgroup= C1 Stoichiometry= C16H28B10BrCsO8RuSe C1[X(C16H28B10BrCsO8RuSe)]
#Atoms= 66
 Charge = 0 Multiplicity = 1

 SCF Energy= -6566.48497002
==
 Optimization incomplete.

Atomic Type	Coordinates (Angstroms) X	Y	Z
B	4.811910	-1.003241	-1.238051
B	5.307050	-1.942831	0.192929
H	6.449550	-2.046221	0.534559
H	5.579950	-0.404601	-1.935381
B	4.183390	-3.309511	0.373729
B	4.001570	-1.873391	1.410239
B	3.351410	-1.777901	-1.922621
B	3.205320	-0.375361	-0.889801
H	2.971090	-1.761971	-3.051821
H	4.086030	-1.960981	2.599869
H	4.396440	-4.379141	0.861959
C	2.151910	-1.712711	-0.671761
C	2.659610	-2.628331	0.671479
B	4.657060	-2.780271	-1.250951
B	2.957080	-3.206831	-0.919191
H	2.325080	-4.140821	-1.300471
H	5.309120	-3.490771	-1.959901
B	4.385800	-0.441161	0.418999
H	4.818690	0.556029	0.915519
B	2.692670	-0.902881	0.713269
H	1.987000	-0.205671	1.602239
C	0.491750	-1.866241	-1.444211
O	-0.148210	-2.804951	-0.965341
O	0.416430	-1.073321	-2.384931
C	1.618760	-3.363661	1.498109
H	1.954730	-4.401701	1.643719
H	1.505360	-2.884511	2.481699
H	0.653750	-3.360411	0.972279

Ru	0.748430	0.083369	0.429409
O	-0.082050	1.760479	1.375169
C	-1.071710	1.832409	0.512529
O	-1.020020	0.978689	-0.435581
C	-2.183790	2.799719	0.695709
H	-1.861240	3.663279	1.296169
H	-2.578430	3.121139	-0.280121
H	-2.994250	2.253679	1.228629
Se	2.125630	1.285309	-1.287421
C	3.282550	2.559959	-0.378541
C	4.466050	2.953009	-1.021241
C	2.918660	3.097339	0.863009
C	5.310710	3.879169	-0.394301
H	4.738480	2.522989	-1.990371
C	3.768600	4.029579	1.477069
H	1.987160	2.784289	1.342819
C	4.963600	4.417179	0.853939
H	6.243510	4.177289	-0.883441
H	3.493900	4.450039	2.449999
H	5.625960	5.139779	1.340899
Br	-4.192920	-0.040901	1.763809
Cs	-2.976070	-1.368991	-1.313351
O	-0.527240	-1.171941	1.626299
C	-0.942720	-0.952181	2.792779
O	-0.727600	0.174639	3.454359
C	-1.740450	-1.976191	3.534059
H	-2.804810	-1.738501	3.331909
H	-1.514440	-2.984541	3.159489
H	-1.562510	-1.901631	4.617279
H	-0.382590	0.862039	2.807049
O	-4.980360	0.848089	-1.957931
C	-5.552940	1.818799	-1.454641
O	-5.640330	2.060679	-0.142381
H	-5.193650	1.309519	0.389789
C	-6.262990	2.883319	-2.268911
H	-6.019750	2.764839	-3.333861
H	-7.353840	2.784089	-2.131371
H	-5.983400	3.890789	-1.920411

Supporting Information: 035-Selenylated-Cluster-RuII-2HOAc-1OAc-CsBr-Decarboxylation-TS-06-SP-TFE-PBE.log

----

Using Gaussian 16: ES64L-G16RevA.03 25-Dec-2016

==

# pbepbe/Def2QZVP/auto empiricaldispersion=gd3bj
scf=(maxcycle=300,direct,vshift=200,tight,yqc) density=current
scrf=(solvent=2,2,2-Trifluoroethanol,smd)

Pointgroup= C1 Stoichiometry= C16H28B10BrCsO8RuSe C1[X(C16H28B10BrCsO8RuSe)]
#Atoms= 66
Charge = 0 Multiplicity = 1

SCF Energy= -6566.47187340
==
Optimization incomplete.

Atomic Type	Coordinates (Angstroms) X	Y	Z
B	-1.394611	3.474579	-1.486669
B	-1.786841	4.735939	-0.291169
H	-2.415311	5.719159	-0.561049
H	-1.727221	3.516339	-2.635959
B	-0.470811	4.777140	0.899731
B	-1.985941	3.952629	1.289241
B	0.161479	2.742720	-1.021189
B	-1.369891	1.913819	-0.642419
H	0.959589	2.229360	-1.740369
H	-2.648691	4.300519	2.222291
H	-0.091841	5.689710	1.573541
C	-0.059191	2.033600	0.540641
C	-0.465451	3.228340	1.641101
B	-0.098921	4.492700	-0.814019
B	0.732269	3.563110	0.432611
H	1.862209	3.636800	0.788601
H	0.516189	5.288950	-1.462029
B	-2.564341	3.133809	-0.172729
H	-3.731811	2.961179	-0.367019
B	-1.712741	2.215639	1.090711
H	-2.145271	1.431749	1.870091
C	1.221200	0.895610	1.139861
O	0.900060	0.088460	2.103731
O	2.363550	1.298110	0.841051
C	0.000789	3.039200	3.077411
H	-0.633181	3.645059	3.742511
H	-0.074831	1.980290	3.365801
H	1.046769	3.363310	3.186981
Ru	-0.232480	-0.463840	0.306061
O	-1.460330	-3.158951	-0.919219
C	-0.309170	-3.295270	-0.433759
O	0.246690	-2.466750	0.407591

C	0.561190	-4.473270	-0.828329
H	-0.053279	-5.295800	-1.221579
H	1.250020	-4.127120	-1.620269
H	1.164691	-4.826390	0.024181
Se	-1.721820	0.124569	-1.574369
C	-3.557970	-0.356021	-1.134939
C	-4.088380	-1.368791	-1.953639
C	-4.322560	0.178259	-0.087289
C	-5.381080	-1.852481	-1.714529
H	-3.482700	-1.792011	-2.761119
C	-5.621580	-0.301611	0.133891
H	-3.915440	0.954609	0.559211
C	-6.152530	-1.318291	-0.672719
H	-5.785170	-2.647871	-2.348889
H	-6.220450	0.127749	0.944631
H	-7.165240	-1.691982	-0.491439
O	-1.724980	-0.746871	1.813301
C	-2.638720	-1.604931	1.914501
O	-2.934200	-2.549131	1.057511
C	-3.544320	-1.571151	3.120431
H	-3.161950	-0.862551	3.867231
H	-4.551290	-1.259141	2.792501
H	-3.636760	-2.579101	3.556041
H	-2.314280	-2.587461	0.214551
Cs	3.384540	-1.693990	1.157871
Br	1.455470	-0.594060	-1.663659
O	5.095740	0.168730	-0.556889
C	4.923470	1.121140	-1.324559
O	3.880189	1.955510	-1.283909
H	3.235659	1.656990	-0.567269
C	5.873049	1.467211	-2.453429
H	5.423120	1.150341	-3.410909
H	6.032309	2.555651	-2.512129
H	6.829550	0.944311	-2.314939

Supporting Information: 035-Selenylated-Cluster-RuII-2HOAc-1OAc-CsBr-Decarboxylation-TS-08-SP-TFE-PBE.log

----

Using Gaussian 16: ES64L-G16RevA.03 25-Dec-2016

==

# pbepbe/Def2QZVP/auto empiricaldispersion=gd3bj
scf=(maxcycle=300,direct,vshift=200,tight,yqc) density=current
scrf=(solvent=2,2,2-Trifluoroethanol,smd)

Pointgroup= C1 Stoichiometry= C16H28B10BrCsO8RuSe C1[X(C16H28B10BrCsO8RuSe)]
#Atoms= 66
Charge = 0 Multiplicity = 1

SCF Energy= -6566.48383585
==

Optimization incomplete.

Atomic Type	Coordinates (Angstroms) X	Y	Z
B	4.733149	1.981322	0.788100
B	4.857239	2.756032	-0.815020
H	5.905169	3.065713	-1.303920
H	5.682319	1.709393	1.465990
B	3.414068	3.770352	-1.054310
B	3.516039	2.174622	-1.842710
B	3.187339	2.498822	1.523060
B	3.307039	0.948862	0.724320
H	2.914929	2.583792	2.680010
H	3.480539	2.081182	-3.034560
H	3.315508	4.759292	-1.718340
C	1.945369	1.948731	0.440570
C	2.094969	2.705021	-1.059800
B	4.140858	3.650632	0.561200
B	2.369588	3.606511	0.382110
H	1.555188	4.416641	0.696470
H	4.652428	4.603562	1.073980
B	4.330969	1.064352	-0.707190
H	4.959420	0.127092	-1.101650
B	2.558479	1.051391	-0.860210
H	2.089630	-0.006439	-1.543060
C	0.350799	1.823721	1.391740
O	-0.542191	2.470620	0.842220
O	0.580029	1.226831	2.438990
C	0.835039	3.000821	-1.856790
H	1.070638	3.770891	-2.607270
H	0.474609	2.092371	-2.363680
H	0.050808	3.367990	-1.180280
Ru	0.917840	-0.260729	-0.339040
Se	2.638950	-0.831139	1.418900
C	3.956170	-1.973998	0.556320
C	5.241730	-2.038998	1.114380
C	3.607821	-2.743298	-0.561530
C	6.202261	-2.870827	0.523520
H	5.497230	-1.429938	1.987480
C	4.576591	-3.579498	-1.137650

H	2.600641	-2.679639	-0.983320
C	5.870721	-3.641167	-0.601440
H	7.212091	-2.914147	0.943810
H	4.314731	-4.181508	-2.013910
H	6.623201	-4.290587	-1.059810
O	-0.870930	-0.918020	0.655960
C	-1.185500	-1.615300	1.657140
O	-2.376910	-2.162080	1.745560
C	-0.293270	-1.876870	2.833720
H	0.516541	-2.561699	2.528590
H	0.166540	-0.929230	3.153930
H	-0.863929	-2.338470	3.651070
H	-2.918450	-2.054801	0.860960
O	-0.543490	-0.046050	-1.985260
C	-0.410440	-1.305210	-2.209770
C	-1.248960	-2.044130	-3.200310
H	-2.224310	-2.262250	-2.718480
H	-1.431740	-1.418610	-4.088240
H	-0.768909	-2.991720	-3.486090
O	0.469450	-1.930269	-1.492300
Cs	-3.051471	1.395849	-0.562510
Br	-4.132600	-2.005061	-0.822400
O	-5.791001	1.119538	0.748290
C	-6.671660	0.342358	1.128040
O	-6.671890	-0.976052	0.900860
H	-5.852590	-1.231072	0.360180
C	-7.895291	0.775847	1.909320
H	-7.973780	0.197807	2.845010
H	-7.835691	1.849658	2.132950
H	-8.806601	0.567107	1.323320

Supporting Information: 035-Selenylated-Cluster-RuII-2HOAc-1OAc-CsBr-Decarboxylation-TS-09-SP-TFE-PBE.log

----

Using Gaussian 16: ES64L-G16RevA.03 25-Dec-2016

==

\# pbepbe/Def2QZVP/auto empiricaldispersion=gd3bj
scf=(maxcycle=300,direct,vshift=200,tight,yqc) density=current
scrf=(solvent=2,2,2-Trifluoroethanol,smd)

Pointgroup= C1 Stoichiometry= C16H28B10BrCsO8RuSe C1[X(C16H28B10BrCsO8RuSe)]
#Atoms= 66
Charge = 0 Multiplicity = 1

SCF Energy= -6566.49018193

==

Optimization incomplete.

Atomic Type	Coordinates (Angstroms) X	Y	Z
B	2.424041	1.691928	-2.917680
B	4.013771	2.009797	-2.180760
H	4.694252	2.944856	-2.493380
H	1.929382	2.394679	-3.755280
B	4.755050	0.446166	-1.776800
B	3.994621	1.421447	-0.494140
B	2.175799	-0.077541	-2.934490
B	1.460960	0.899399	-1.677420
H	1.508099	-0.706611	-3.697300
H	4.638241	1.814297	0.433250
H	5.918780	0.178045	-1.716710
C	2.214459	-0.576821	-1.270360
C	3.741249	-0.262933	-0.615040
B	3.788710	0.592807	-3.255020
B	3.610729	-0.853243	-2.220630
H	3.953818	-1.974173	-2.438150
H	4.297650	0.488697	-4.334470
B	2.549721	2.194538	-1.190770
H	2.136952	3.234369	-0.770960
B	2.387360	0.718738	-0.195060
H	1.952910	0.934879	1.049400
C	1.319377	-2.326981	-1.156050
O	1.994407	-3.106201	-0.516590
O	0.345598	-2.235530	-1.897340
C	4.300208	-1.214853	0.429500
H	5.342348	-1.453804	0.167010
H	4.275069	-0.744993	1.423230
H	3.704328	-2.137473	0.455160
Ru	0.754089	-0.289680	0.847430
Se	-0.459470	0.811771	-1.055340
C	-0.829118	2.689191	-0.718150
C	-1.427738	3.050792	0.497010
C	-0.575227	3.647471	-1.710550
C	-1.761027	4.394622	0.723910
H	-1.621919	2.288682	1.254710
C	-0.908336	4.987881	-1.470320
H	-0.098988	3.357091	-2.651830
C	-1.501056	5.364002	-0.255740
H	-2.213226	4.682312	1.679240
H	-0.695796	5.740821	-2.236170

H	-1.754255	6.412882	-0.071540
O	1.872598	-1.490561	2.215050
C	1.555578	-1.759721	3.398290
C	2.284577	-2.839701	4.148320
H	3.239987	-3.062062	3.653920
H	2.444487	-2.545732	5.197310
H	1.661586	-3.751701	4.142600
O	0.584218	-1.174240	4.073170
Cs	-2.631842	-2.157787	-2.021850
Br	-1.207452	-1.971678	1.261670
H	0.138729	-0.481460	3.462910
O	-0.128840	0.719571	2.420370
C	0.432011	1.842250	2.901950
O	1.464122	2.366359	2.485250
C	-0.371638	2.397391	4.071420
H	-1.453648	2.326642	3.874600
H	-0.161339	1.798761	4.976140
H	-0.077718	3.439631	4.260790
O	-4.040101	-0.051186	-0.338510
C	-4.042190	0.676574	0.658110
O	-3.250950	0.496603	1.726800
H	-2.631021	-0.288697	1.562320
C	-4.911059	1.906535	0.799750
H	-4.302048	2.794504	0.550340
H	-5.758089	1.850385	0.101930
H	-5.267029	2.025025	1.834940

Supporting Information: 035-Selenylated-Cluster-RuII-2HOAc-1OAc-CsBr-Decarboxylation-TS-10-SP-TFE-PBE.log

----

Using Gaussian 16: ES64L-G16RevA.03 25-Dec-2016

==

# pbepbe/Def2QZVP/auto empiricaldispersion=gd3bj
scf=(maxcycle=300,direct,vshift=200,tight,yqc) density=current
scrf=(solvent=2,2,2-Trifluoroethanol,smd)

Pointgroup= C1 Stoichiometry= C16H28B10BrCsO8RuSe C1[X(C16H28B10BrCsO8RuSe)] #Atoms= 66

Charge = 0 Multiplicity = 1

SCF Energy= -6566.48711731

==

Optimization incomplete.

Atomic Coordinates (Angstroms)

Type	X	Y	Z
B	-2.736320	3.353921	-1.175540
B	-2.212659	4.365171	0.192810
H	-2.887289	5.251651	0.633520
H	-3.794560	3.478171	-1.726790
B	-0.446819	4.532010	0.110070
B	-1.172640	3.387300	1.273100
B	-1.270710	2.860830	-2.078590
B	-2.001790	1.778591	-0.925150
H	-1.197960	2.576990	-3.235050
H	-0.996070	3.523360	2.447070
H	0.223821	5.439290	0.506140
C	-0.303210	1.959250	-0.944190
C	0.175720	2.970840	0.313050
B	-1.383329	4.495540	-1.392060
B	0.146980	3.579410	-1.283910
H	1.188010	3.802300	-1.821380
H	-1.446249	5.461060	-2.098170
B	-2.592510	2.653331	0.484520
H	-3.519560	2.269111	1.132920
B	-1.044260	1.769780	0.547630
H	-0.951880	0.770630	1.443930
C	0.994660	0.828680	-2.167190
O	2.129340	1.078359	-1.804920
O	0.276239	0.324410	-2.996720
C	1.543740	2.759200	0.933910
H	2.031370	3.737679	1.063370
H	1.453200	2.267650	1.911820
H	2.160300	2.133229	0.274880
Ru	-0.397351	-0.271610	0.203670
Se	-2.383971	-0.153269	-1.370300
C	-4.012681	-0.520799	-0.382770
C	-4.184761	-1.820169	0.122540
C	-5.042980	0.425841	-0.277220
C	-5.380161	-2.157739	0.772100
H	-3.389411	-2.562159	0.012240
C	-6.232221	0.079742	0.379730
H	-4.920530	1.426761	-0.698570
C	-6.401541	-1.206528	0.911310
H	-5.512001	-3.170739	1.167610
H	-7.029560	0.824002	0.473460
H	-7.331681	-1.470648	1.424240
O	0.307669	-1.936410	-1.037740
C	-0.161281	-3.104520	-1.040730
O	-0.977132	-3.597010	-0.139940

C	0.210678	-4.067530	-2.142910
H	-0.708002	-4.465240	-2.605310
H	0.810998	-3.561560	-2.912400
H	0.760368	-4.929820	-1.727070
H	-1.138761	-2.869060	0.626470
Cs	3.605429	-1.489251	-1.115630
Br	1.682299	-0.630200	1.753300
O	-1.239121	-1.701400	1.423190
C	-1.831401	-1.390379	2.586420
O	-1.915521	-0.250349	3.033780
C	-2.435621	-2.602469	3.279260
H	-2.435511	-2.435429	4.366240
H	-3.483281	-2.708559	2.946130
H	-1.896162	-3.532769	3.040860
O	5.316429	0.288859	0.641170
C	5.240520	1.182739	1.487960
O	4.165380	1.405209	2.257960
H	3.435410	0.747939	2.019120
C	6.347580	2.173538	1.774560
H	7.257070	1.888368	1.228760
H	6.029350	3.182038	1.458280
H	6.549710	2.222618	2.856910

Supporting Information: 035-Selenylated-Cluster-RuII-2HOAc-1OAc-CsBr-Decarboxylation-TS-11-SP-TFE-PBE.log

----

Using Gaussian 16: ES64L-G16RevA.03 25-Dec-2016

==

# pbepbe/Def2QZVP/auto empiricaldispersion=gd3bj
scf=(maxcycle=300,direct,vshift=200,tight,yqc) density=current
scrf=(solvent=2,2,2-Trifluoroethanol,smd)

Pointgroup= C1 Stoichiometry= C16H28B10BrCsO8RuSe C1[X(C16H28B10BrCsO8RuSe)]
#Atoms= 66
Charge = 0 Multiplicity = 1

SCF Energy= -6566.48756964

==

Optimization incomplete.

Atomic Type	Coordinates (Angstroms) X	Y	Z
B	-4.326799	-2.070462	0.149040
B	-4.626109	-2.254882	-1.597560

H	-5.724309	-2.392182	-2.055170
H	-5.197029	-2.057992	0.972510
B	-3.236358	-3.112581	-2.311630
B	-3.382769	-1.343691	-2.494440
B	-2.727009	-2.787071	0.504790
B	-2.892519	-1.060471	0.306170
H	-2.350298	-3.257801	1.532480
H	-3.464119	-0.853841	-3.582800
H	-3.227328	-3.817621	-3.277200
C	-1.581439	-1.886720	-0.444840
C	-1.902839	-2.096481	-2.088030
B	-3.799288	-3.556392	-0.686850
B	-2.053408	-3.433221	-1.012930
H	-1.240198	-4.301470	-1.082350
H	-4.283028	-4.631832	-0.479580
B	-4.049580	-0.691182	-0.974630
H	-4.692030	0.317298	-0.963220
B	-2.299250	-0.621351	-1.293770
H	-1.745630	0.504129	-1.641300
C	0.218401	-2.252040	0.249090
O	0.970081	-2.504139	-0.678830
O	0.097811	-2.324620	1.465470
C	-0.746379	-2.074220	-3.074790
H	-1.164879	-2.146630	-4.090870
H	-0.176609	-1.136470	-2.982850
H	-0.067518	-2.918450	-2.896160
Ru	-0.545210	0.361700	-0.368880
Se	-2.122250	0.321499	1.560210
C	-3.446381	1.716539	1.261060
C	-4.698951	1.594248	1.880340
C	-3.128641	2.832289	0.476150
C	-5.658621	2.597188	1.686980
H	-4.931500	0.714028	2.488310
C	-4.095432	3.833588	0.297460
H	-2.144851	2.908739	0.004180
C	-5.358022	3.716598	0.896280
H	-6.644041	2.500067	2.153960
H	-3.857122	4.707748	-0.317530
H	-6.109952	4.498197	0.747470
O	0.900960	0.740851	-2.046650
C	0.696059	1.995221	-1.869400
C	1.335519	3.039721	-2.742150
H	1.196728	4.038641	-2.303880
H	2.409889	2.822291	-2.853160
H	0.875499	3.015091	-3.745580
O	-0.108601	2.344830	-0.924310

Cs	3.529741	-0.847818	-1.061300
Br	1.323400	0.718841	1.360380
O	2.756898	3.476312	0.443180
C	3.793839	3.280732	-0.380930
C	4.521078	4.574932	-0.681010
H	3.878608	5.220532	-1.305400
H	4.728868	5.128013	0.248980
H	5.455508	4.364163	-1.218770
O	4.108179	2.189552	-0.865720
H	2.297759	2.593961	0.634140
O	3.722831	-2.258048	1.626750
C	3.408041	-2.293318	2.818890
O	2.151571	-2.360459	3.271380
H	1.507901	-2.335069	2.499390
C	4.407731	-2.242878	3.956430
H	4.154902	-2.982608	4.732610
H	5.422301	-2.420347	3.573590
H	4.367321	-1.245908	4.429530

Supporting Information: 035-Selenylated-Cluster-RuII-2HOAc-1OAc-CsBr-Decarboxylation-TS-12-SP-TFE-PBE.log

----

Using Gaussian 16: ES64L-G16RevA.03 25-Dec-2016

==

# pbepbe/Def2QZVP/auto empiricaldispersion=gd3bj
scf=(maxcycle=300,direct,vshift=200,tight,yqc) density=current
scrf=(solvent=2,2,2-Trifluoroethanol,smd)

Pointgroup= C1 Stoichiometry= C16H28B10BrCsO8RuSe C1[X(C16H28B10BrCsO8RuSe)]
#Atoms= 66
Charge = 0 Multiplicity = 1

SCF Energy= -6566.48756922

==

Optimization incomplete.

Atomic Type	Coordinates (Angstroms) X	Y	Z
B	-4.326808	-2.070493	0.149010
B	-4.626128	-2.254893	-1.597590
H	-5.724338	-2.392204	-2.055190
H	-5.197028	-2.058053	0.972490
B	-3.236377	-3.112562	-2.311670
B	-3.382809	-1.343662	-2.494460

B	-2.727008	-2.787082	0.504740
B	-2.892549	-1.060482	0.306150
H	-2.350287	-3.257832	1.532430
H	-3.464169	-0.853802	-3.582810
H	-3.227347	-3.817582	-3.277260
C	-1.581458	-1.886701	-0.444880
C	-1.902868	-2.096451	-2.088070
B	-3.799277	-3.556402	-0.686890
B	-2.053397	-3.433201	-1.012990
H	-1.240187	-4.301441	-1.082430
H	-4.283006	-4.631853	-0.479640
B	-4.049619	-0.691193	-0.974630
H	-4.692080	0.317277	-0.963210
B	-2.299279	-0.621352	-1.293780
H	-1.745640	0.504119	-1.641270
C	0.218502	-2.252040	0.249120
O	0.970172	-2.504129	-0.678810
O	0.097852	-2.324690	1.465480
C	-0.746408	-2.074171	-3.074840
H	-1.164878	-2.147041	-4.090890
H	-0.176909	-1.136220	-2.983200
H	-0.067298	-2.918120	-2.895910
Ru	-0.545210	0.361670	-0.368850
Se	-2.122260	0.321449	1.560220
C	-3.446371	1.716508	1.261080
C	-4.698940	1.594237	1.880370
C	-3.128621	2.832258	0.476190
C	-5.658601	2.597186	1.687010
H	-4.931500	0.714007	2.488330
C	-4.095392	3.833577	0.297510
H	-2.144821	2.908709	0.004210
C	-5.357982	3.716596	0.896320
H	-6.644021	2.500076	2.153990
H	-3.857073	4.707737	-0.317480
H	-6.109902	4.498206	0.747520
O	0.900970	0.740861	-2.046610
C	0.696049	1.995230	-1.869360
C	1.335449	3.039741	-2.742140
H	1.196898	4.038631	-2.303700
H	2.409759	2.822182	-2.853440
H	0.875169	3.015311	-3.745450
O	-0.108611	2.344820	-0.924280
Cs	3.529761	-0.847788	-1.061320
Br	1.323400	0.718831	1.360420
H	1.507952	-2.335029	2.499510
O	2.151652	-2.360379	3.271460

C	3.408102	-2.293258	2.818900
C	4.407852	-2.242797	3.956390
H	4.367281	-1.245927	4.429650
H	4.155192	-2.982707	4.732460
H	5.422432	-2.420047	3.573460
O	3.722822	-2.258048	1.626740
O	4.108069	2.189583	-0.865810
C	3.793748	3.280742	-0.380960
O	2.756898	3.476272	0.443280
H	2.297759	2.593911	0.634210
C	4.520878	4.574963	-0.681190
H	4.727437	5.128893	0.248560
H	5.455948	4.364114	-1.217800
H	3.878877	5.219782	-1.306890

Supporting Information: 035-Selenylated-Cluster-RuII-2HOAc-1OAc-CsBr-Decarboxylation-TS-13-SP-TFE-PBE.log

----

Using Gaussian 16: ES64L-G16RevA.03 25-Dec-2016

===

# pbepbe/Def2QZVP/auto empiricaldispersion=gd3bj
scf=(maxcycle=300,direct,vshift=200,tight,yqc) density=current
scrf=(solvent=2,2,2-Trifluoroethanol,smd)

Pointgroup= C1 Stoichiometry= C16H28B10BrCsO8RuSe C1[X(C16H28B10BrCsO8RuSe)]
#Atoms= 66
Charge = 0 Multiplicity = 1

SCF Energy= -6566.48386088

===

Optimization incomplete.

Atomic Type	Coordinates (Angstroms) X	Y	Z
B	-2.538447	3.428192	-0.517461
B	-2.036127	4.125992	1.043109
H	-2.676766	4.962352	1.612939
H	-3.552367	3.726673	-1.081651
B	-0.258457	4.175651	1.081059
B	-1.130028	2.891431	1.965429
B	-1.056058	3.008001	-1.426051
B	-1.916129	1.783172	-0.541031
H	-0.925218	2.946321	-2.609491
H	-1.014288	2.795341	3.152729

H	0.443494	4.942640	1.671619
C	-0.213678	1.841981	-0.435951
C	0.252472	2.568060	1.015539
B	-1.106046	4.487691	-0.444721
B	0.351693	3.459090	-0.443851
H	1.434763	3.713699	-0.873771
H	-1.059016	5.569561	-0.956271
B	-2.541558	2.423992	0.979529
H	-3.536238	1.990103	1.481569
B	-1.061559	1.428571	0.958129
H	-1.137220	0.245861	1.642729
C	1.079631	0.893150	-1.802921
O	2.200691	0.897599	-1.318241
O	0.395271	0.690230	-2.771201
C	1.577632	2.166149	1.640079
H	2.353852	2.900479	1.378929
H	1.470712	2.137959	2.735409
H	1.876631	1.169559	1.289929
Ru	-0.502050	-0.530709	0.277069
Se	-2.469780	-0.002078	-1.290491
C	-4.225740	-0.074536	-0.448701
C	-5.255489	0.641444	-1.079131
C	-4.465230	-0.809016	0.718679
C	-6.539529	0.636155	-0.517441
H	-5.050579	1.214824	-1.988851
C	-5.756810	-0.812625	1.266949
H	-3.652541	-1.367657	1.190909
C	-6.791700	-0.090854	0.655499
H	-7.342009	1.205096	-0.998081
H	-5.948621	-1.382295	2.182289
H	-7.795500	-0.093334	1.092439
Br	-1.402932	-2.675408	1.299909
O	0.404329	-1.767450	-1.296171
C	-0.197492	-2.540359	-2.078741
O	-1.311622	-3.183219	-1.786431
H	-1.520382	-3.022638	-0.796671
C	0.327138	-2.769580	-3.470331
H	0.395949	-1.789260	-3.971691
H	1.345958	-3.189981	-3.421051
H	-0.322233	-3.448749	-4.039151
O	1.311629	-0.896011	1.237519
C	1.656389	-1.117551	2.492759
C	0.649709	-0.842380	3.598139
H	1.119359	-1.020340	4.576039
H	0.286840	0.198940	3.547449
H	-0.229711	-1.496779	3.480009

O	2.809589	-1.517242	2.753919
Cs	3.823989	-1.639032	-0.195661
O	5.363371	1.015536	-0.396401
C	5.210902	2.236616	-0.350521
O	4.059072	2.858797	-0.668191
H	3.382362	2.166288	-0.916371
C	6.276783	3.218736	0.077589
H	5.975413	3.702186	1.022949
H	7.231802	2.695845	0.220699
H	6.386533	4.018036	-0.673461

Supporting Information: 035-Selenylated-Cluster-RuII-2HOAc-1OAc-CsBr-Decarboxylation-TS-14-SP-TFE-PBE.log

----

Using Gaussian 16: ES64L-G16RevA.03 25-Dec-2016

==

# pbepbe/Def2QZVP/auto empiricaldispersion=gd3bj
scf=(maxcycle=300,direct,vshift=200,tight,yqc) density=current
scrf=(solvent=2,2,2-Trifluoroethanol,smd)

Pointgroup= C1 Stoichiometry= C16H28B10BrCsO8RuSe C1[X(C16H28B10BrCsO8RuSe)] #Atoms= 66

Charge = 0 Multiplicity = 1

SCF Energy= -6566.49563369

==

Optimization incomplete.

Atomic Type	Coordinates (Angstroms) X	Y	Z
B	3.665911	-0.906730	-2.581660
B	4.606122	-2.160699	-1.737690
H	5.762792	-2.366839	-1.970670
H	4.127391	-0.178170	-3.413610
B	3.512652	-3.507730	-1.357400
B	3.968782	-2.348190	-0.077120
B	1.967771	-1.474370	-2.679700
B	2.453591	-0.354050	-1.440760
H	1.154501	-1.181661	-3.501290
H	4.548852	-2.721539	0.899790
H	3.785623	-4.665830	-1.238810
C	1.443911	-1.669311	-1.032460
C	2.362562	-2.883630	-0.287500
B	3.303342	-2.627260	-2.879190

B	1.865092	-3.076140	-1.913910
H	1.034332	-3.897831	-2.155250
H	3.502742	-3.168160	-3.928990
B	4.061861	-0.732350	-0.827690
H	4.785421	0.115271	-0.394940
B	2.604831	-1.219320	0.084740
H	2.375101	-0.707650	1.286040
C	-0.679829	-1.715001	-1.135010
O	-1.076508	-2.670572	-0.492850
O	-0.963979	-0.871632	-1.958090
C	1.701782	-3.793271	0.735170
H	0.613022	-3.792141	0.591850
H	2.089383	-4.813620	0.590560
H	1.925172	-3.457760	1.756810
Ru	0.766461	-0.243331	0.816680
Se	1.476910	1.345559	-0.987650
C	3.046060	2.421340	-0.559250
C	3.780240	2.920060	-1.646760
C	3.416180	2.703550	0.760260
C	4.920229	3.697871	-1.403570
H	3.478640	2.684340	-2.672540
C	4.555859	3.490161	0.988670
H	2.822980	2.312770	1.593210
C	5.309059	3.983481	-0.086010
H	5.506049	4.076991	-2.247260
H	4.854419	3.711831	2.018660
H	6.200329	4.591061	0.101240
Br	0.853110	1.240699	2.856480
O	-1.298289	0.340308	0.703310
C	-2.005560	1.441378	0.664070
O	-3.251710	1.355428	0.844010
C	-1.331700	2.755598	0.361650
H	-0.958970	2.751878	-0.676450
H	-2.038141	3.588058	0.489910
H	-0.464600	2.877409	1.032950
O	0.174161	-1.784361	2.217220
C	-0.964518	-2.078552	2.642820
C	-1.141548	-3.189382	3.644030
H	-1.874848	-3.922922	3.268630
H	-0.180068	-3.683001	3.837950
H	-1.540258	-2.774422	4.585450
O	-2.084939	-1.479052	2.279340
Cs	-4.068129	-1.208343	-0.724040
H	-1.832869	-0.692372	1.640290
O	-5.481840	1.391887	-1.437520
C	-5.531550	2.598327	-1.169310

O	-4.813330	3.189207	-0.210650
H	-4.210260	2.490147	0.239800
C	-6.425811	3.579886	-1.898630
H	-5.814751	4.373227	-2.361370
H	-7.007370	3.058586	-2.671050
H	-7.106121	4.073736	-1.184470

Supporting Information: 035-Selenylated-Cluster-RuII-2HOAc-1OAc-CsBr-Decarboxylation-TS-15-SP-TFE-PBE.log

----

Using Gaussian 16: ES64L-G16RevA.03 25-Dec-2016

==

# pbepbe/Def2QZVP/auto empiricaldispersion=gd3bj
scf=(maxcycle=300,direct,vshift=200,tight,yqc) density=current
scrf=(solvent=2,2,2-Trifluoroethanol,smd)

Pointgroup= C1 Stoichiometry= C16H28B10BrCsO8RuSe C1[X(C16H28B10BrCsO8RuSe)]
#Atoms= 66
Charge = 0 Multiplicity = 1

SCF Energy= -6566.48973704

==

Optimization incomplete.

Atomic Type	Coordinates (Angstroms) X	Y	Z
B	-4.731670	1.030899	-0.002050
B	-4.550980	2.186949	1.343920
H	-5.393120	2.343879	2.181070
H	-5.690020	0.325709	-0.143240
B	-3.588521	3.560850	0.761210
B	-2.818400	2.308770	1.765760
B	-3.848290	1.690910	-1.411490
B	-3.110330	0.474850	-0.394970
H	-4.058550	1.490760	-2.567470
H	-2.330760	2.588250	2.821950
H	-3.619021	4.694220	1.141050
C	-2.196230	1.823090	-0.905660
C	-2.061051	2.930130	0.369880
B	-4.744460	2.784129	-0.334390
B	-3.131961	3.253920	-0.937210
H	-2.830851	4.118340	-1.699900
H	-5.712011	3.374209	-0.721690
B	-3.521400	0.742820	1.298720

H	-3.588580	-0.164480	2.074370
B	-1.927500	1.231430	0.661780
H	-0.864480	0.677520	1.255310
C	-1.066750	1.766130	-2.348310
O	-0.285750	2.716080	-2.356460
O	-1.420220	0.814420	-3.044680
C	-0.826301	3.810050	0.468010
H	-0.308621	3.815940	-0.502290
H	-1.146761	4.830770	0.727410
H	-0.144411	3.429020	1.240940
Ru	-0.244720	0.138190	-0.245680
Se	-2.227650	-1.242600	-0.985540
C	-3.015239	-2.388260	0.382090
C	-4.325849	-2.833420	0.146380
C	-2.312579	-2.790680	1.524330
C	-4.945239	-3.675631	1.079580
H	-4.864999	-2.508851	-0.749520
C	-2.939469	-3.641500	2.447250
H	-1.293939	-2.433500	1.694710
C	-4.252659	-4.081940	2.229990
H	-5.972419	-4.012341	0.905790
H	-2.395499	-3.953380	3.345120
H	-4.738499	-4.740511	2.957140
Br	1.081710	-1.444729	1.214690
O	0.991890	-0.551679	-1.862670
C	1.450190	-1.753979	-2.209760
O	2.647320	-1.920129	-2.492780
C	0.442681	-2.880850	-2.264170
H	-0.429469	-2.586100	-2.868930
H	0.915241	-3.777559	-2.689600
H	0.085031	-3.103680	-1.243550
O	1.224310	1.669021	0.113910
C	2.186050	1.986701	-0.638350
C	2.935779	3.269351	-0.373160
H	3.185989	3.759941	-1.326220
H	2.329489	3.940951	0.251110
H	3.873649	3.036351	0.161200
O	2.633110	1.261371	-1.629170
H	1.934430	0.413001	-1.813510
Cs	4.528940	-1.066619	-0.290660
O	3.988090	1.002961	1.905190
C	3.237940	1.534971	2.731520
O	2.027500	1.070251	3.059540
H	1.808020	0.282071	2.471640
C	3.572100	2.811351	3.470420
H	3.225450	2.766871	4.514500

H	4.653889	2.999521	3.428570
H	3.044359	3.651601	2.984790

Supporting Information: 035-Selenylated-Cluster-RuII-2HOAc-1OAc-CsBr-Decarboxylation-TS-16-SP-TFE-PBE.log

----

Using Gaussian 16: ES64L-G16RevA.03 25-Dec-2016

==

# pbepbe/Def2QZVP/auto empiricaldispersion=gd3bj
scf=(maxcycle=300,direct,vshift=200,tight,yqc) density=current
scrf=(solvent=2,2,2-Trifluoroethanol,smd)

Pointgroup= C1 Stoichiometry= C16H28B10BrCsO8RuSe C1[X(C16H28B10BrCsO8RuSe)] #Atoms= 66
Charge = 0 Multiplicity = 1

SCF Energy= -6566.44176836

==

Optimization incomplete.

Atomic Type	Coordinates (Angstroms) X	Y	Z
B	-3.509359	2.420242	-1.729160
B	-2.932428	4.032262	-1.263620
H	-3.646718	4.993863	-1.212640
H	-4.644860	2.174493	-2.020770
B	-1.244838	4.174101	-1.784800
B	-1.616559	3.819281	-0.091000
B	-2.170110	1.556552	-2.526530
B	-2.543600	1.214992	-0.840180
H	-2.259891	0.704932	-3.358090
H	-1.806613	-3.017579	-0.135170
H	-1.256748	4.555611	0.781110
H	-0.625098	5.153971	-2.082010
C	-0.907510	1.406701	-1.373150
C	-0.389569	2.970410	-0.928000
B	-2.405909	3.303352	-2.808750
B	-0.771849	2.650531	-2.562480
H	0.123541	2.614270	-3.347250
H	-2.720429	3.719322	-3.887680
B	-3.007369	2.728082	-0.042860
H	-3.759239	2.715003	0.888720
B	-1.356600	2.075291	0.154640
H	-0.869520	1.673031	1.158630

C	0.523749	0.208430	-2.244710
O	1.588349	0.196939	-1.531090
O	0.225029	0.026870	-3.400150
Cs	4.042028	-1.085162	-0.313630
C	1.071071	3.177830	-0.566090
H	1.148451	3.645459	0.426290
H	1.595770	2.212809	-0.546820
H	1.550741	3.833669	-1.309640
Ru	0.205998	-0.932350	-0.350710
O	-0.561532	-2.157909	-1.954520
O	-0.704393	-2.383719	1.053920
C	-1.400973	-3.083309	-1.989220
C	0.390717	-3.058550	1.024310
O	-2.053843	-3.569238	-0.939840
O	1.319077	-2.625041	0.238130
C	0.583866	-4.284450	1.864480
H	0.644136	-3.987970	2.926140
H	-0.286854	-4.952040	1.756320
H	1.504246	-4.815321	1.579910
C	-1.778043	-3.717169	-3.298130
H	-1.910024	-4.804499	-3.183800
H	-2.745433	-3.293348	-3.622630
H	-1.019753	-3.487289	-4.058340
Se	-3.270011	-0.566298	-0.321840
C	-3.653241	-0.244837	1.547170
C	-4.993291	-0.077857	1.933910
C	-2.630921	-0.209188	2.509930
C	-5.310121	0.134594	3.283340
H	-5.780031	-0.098546	1.173980
C	-2.955261	0.015882	3.855340
H	-1.588761	-0.346359	2.212080
C	-4.291621	0.185473	4.245960
H	-6.355791	0.271414	3.578880
H	-2.152911	0.057632	4.600010
H	-4.539341	0.359683	5.298400
Br	1.265739	0.131539	1.738130
O	4.809810	1.621407	0.818960
C	4.407391	2.644737	1.377750
O	3.183451	2.780068	1.911260
H	2.653800	1.938759	1.750410
C	5.225422	3.907427	1.529570
H	6.266611	3.718006	1.235540
H	4.800822	4.697447	0.885870
H	5.179462	4.275957	2.567040

Supporting Information: 035-Selenylated-Cluster-RuII-2HOAc-1OAc-CsBr-Decarboxylation-TS-17-SP-TFE-PBE.log

----
Using Gaussian 16: ES64L-G16RevA.03 25-Dec-2016
==
# pbepbe/Def2QZVP/auto empiricaldispersion=gd3bj
scf=(maxcycle=300,direct,vshift=200,tight,yqc) density=current
scrf=(solvent=2,2,2-Trifluoroethanol,smd)

Pointgroup= C1 Stoichiometry= C16H28B10BrCsO8RuSe C1[X(C16H28B10BrCsO8RuSe)]
#Atoms= 66
Charge = 0 Multiplicity = 1

SCF Energy= -6566.44176942
==
Optimization incomplete.

Atomic Type	Coordinates (Angstroms) X	Y	Z
B	-3.509303	2.420187	-1.729189
B	-2.932414	4.032227	-1.263679
H	-3.646724	4.993807	-1.212729
H	-4.644792	2.174396	-2.020809
B	-1.244834	4.174098	-1.784849
B	-1.616544	3.819308	-0.091049
B	-2.170022	1.556498	-2.526529
B	-2.543522	1.214977	-0.840179
H	-2.259771	0.704858	-3.358079
H	-1.806559	-3.017712	-0.135369
H	-1.256754	4.555658	0.781041
H	-0.625105	5.153969	-2.082079
C	-0.907442	1.406699	-1.373139
C	-0.389533	2.970439	-0.928019
B	-2.405863	3.303287	-2.808789
B	-0.771793	2.650509	-2.562499
H	0.123617	2.614249	-3.347249
H	-2.720383	3.719227	-3.887729
B	-3.007333	2.728067	-0.042889
H	-3.759213	2.715006	0.888681
B	-1.356542	2.075318	0.154631
H	-0.869472	1.673089	1.158631
C	0.523899	0.208420	-2.244639
O	1.588449	0.196950	-1.530939
O	0.225259	0.026909	-3.400109
Cs	4.042060	-1.085138	-0.313349

C	1.071107	3.177930	-0.566119
H	1.148477	3.645460	0.426301
H	1.595878	2.212930	-0.546959
H	1.550706	3.833860	-1.309629
Ru	0.206050	-0.932411	-0.350739
O	-0.561469	-2.157881	-1.954629
O	-0.704419	-2.383881	1.053731
C	-1.400918	-3.083262	-1.989429
C	0.390691	-3.058701	1.024181
O	-2.053798	-3.569292	-0.940099
O	1.319121	-2.625130	0.238111
C	0.583642	-4.284800	1.864121
H	0.641692	-3.988800	2.926031
H	-0.286227	-4.953211	1.754081
H	1.505013	-4.814640	1.580861
C	-1.778018	-3.716952	-3.298409
H	-1.910557	-4.804212	-3.184119
H	-2.745128	-3.292653	-3.623099
H	-1.019478	-3.487422	-4.058479
Se	-3.269850	-0.566323	-0.321799
C	-3.653121	-0.244843	1.547201
C	-4.993171	-0.077744	1.933901
C	-2.630831	-0.209273	2.509981
C	-5.310011	0.134755	3.283321
H	-5.779901	-0.098375	1.173961
C	-2.955171	0.015847	3.855381
H	-1.588670	-0.346542	2.212141
C	-4.291521	0.185556	4.245971
H	-6.355671	0.271665	3.578841
H	-2.152841	0.057518	4.600071
H	-4.539251	0.359806	5.298401
Br	1.265639	0.131370	1.738221
O	4.809288	1.621983	0.818321
C	4.406897	2.645062	1.377591
O	3.183127	2.780032	1.911581
H	2.653528	1.938721	1.750571
C	5.224726	3.907893	1.529301
H	6.266287	3.718224	1.236771
H	4.800986	4.697193	0.884121
H	5.177356	4.277563	2.566291

Supporting Information: 035-Selenylated-Cluster-RuII-2HOAc-1OAc-CsBr-Decarboxylation-TS-18-SP-TFE-PBE.log

----

Using Gaussian 16: ES64L-G16RevA.03 25-Dec-2016

==

\# pbepbe/Def2QZVP/auto empiricaldispersion=gd3bj

scf=(maxcycle=300,direct,vshift=200,tight,yqc) density=current
scrf=(solvent=2,2,2-Trifluoroethanol,smd)

Pointgroup= C1 Stoichiometry= C16H28B10BrCsO8RuSe C1[X(C16H28B10BrCsO8RuSe)]
#Atoms= 66
Charge = 0 Multiplicity = 1

SCF Energy= -6566.42591525
==
Optimization incomplete.

Atomic Type	Coordinates (Angstroms) X	Y	Z
B	-3.370152	1.372959	-2.365631
B	-2.196812	0.976639	-3.646251
H	-2.061103	1.642769	-4.632591
H	-4.103673	2.318809	-2.407971
B	-2.006211	-0.792330	-3.699541
B	-0.790332	0.204050	-2.881031
B	-3.903132	-0.141151	-1.621521
B	-2.687262	0.833979	-0.793531
H	-4.951842	-0.367482	-1.107871
H	0.337208	-0.528429	2.476479
H	0.351218	0.189561	-3.239461
H	-1.701801	-1.480650	-4.627351
C	-2.484261	-0.875591	-0.928001
C	-1.387301	-1.222610	-2.173601
B	-3.605012	-0.084051	-3.373091
B	-3.054621	-1.486831	-2.434441
H	-3.448121	-2.610361	-2.472131
H	-4.509082	-0.217452	-4.145791
B	-1.629543	1.538380	-2.052011
H	-1.077143	2.590360	-1.887731
B	-1.100262	0.133850	-1.130961
H	-0.203522	0.133830	-0.350881
C	-3.195891	-2.167611	0.258639
O	-2.534550	-3.244471	0.130819
O	-4.184431	-1.753432	0.809769
C	-0.513041	-2.434450	-1.913521
H	0.263789	-2.515399	-2.694581
H	0.242929	-2.273999	-1.055791
H	-1.084740	-3.369250	-1.852501
Ru	-0.804021	-2.100720	0.537789

O	-1.636091	-1.822930	2.404349
O	1.080389	-1.199279	1.223199
C	-1.340861	-0.972980	3.281639
C	1.528629	-2.401819	1.472559
O	-0.230862	-0.251540	3.278539
O	0.689380	-3.353369	1.287429
C	2.936269	-2.643368	1.893739
H	3.576129	-2.487328	1.005279
H	3.238469	-1.910658	2.659979
H	3.060790	-3.670308	2.265539
C	-2.292311	-0.696481	4.403639
H	-2.912102	0.169349	4.105509
H	-2.955681	-1.558801	4.555339
H	-1.752192	-0.440530	5.327229
Se	-3.010963	1.543829	1.049969
C	-1.657553	2.927800	1.081089
C	-1.767774	4.061270	0.253889
C	-0.582133	2.831100	1.983769
C	-0.793384	5.068950	0.303799
H	-2.616234	4.146159	-0.431221
C	0.381606	3.852521	2.046209
H	-0.499253	1.957790	2.636969
C	0.284866	4.969351	1.199529
H	-0.888435	5.944350	-0.347391
H	1.204576	3.776021	2.766259
H	1.030465	5.769911	1.255239
Br	2.905469	-1.492658	-1.759181
Cs	2.159477	1.862921	-0.922221
O	4.710158	1.098893	0.525689
C	5.514248	0.171943	0.671139
O	5.452849	-1.001927	0.036879
H	4.630839	-1.043337	-0.581971
C	6.707178	0.242484	1.604799
H	7.641228	0.127644	1.029109
H	6.713568	1.204134	2.136009
H	6.672938	-0.588936	2.328869

Supporting Information: 035-Selenylated-Cluster-RuII-2HOAc-1OAc-CsBr-Decarboxylation-TS-19-SP-TFE-PBE.log

```
------------------------------------------------------------------------
 Using  Gaussian 16:  ES64L-G16RevA.03 25-Dec-2016
========================================================================
 # pbepbe/Def2QZVP/auto empiricaldispersion=gd3bj
 scf=(maxcycle=300,direct,vshift=200,tight,yqc) density=current
 scrf=(solvent=2,2,2-Trifluoroethanol,smd)
------------------------------------------------------------------------
```

Pointgroup= C1 Stoichiometry= C16H28B10BrCsO8RuSe C1[X(C16H28B10BrCsO8RuSe)]
#Atoms= 66
Charge = 0 Multiplicity = 1

SCF Energy= -6566.48648147

===

Optimization incomplete.

Atomic Type	Coordinates (Angstroms) X	Y	Z
B	-4.520849	2.232800	-0.227731
B	-4.335509	2.985540	1.377439
H	-5.269239	3.348250	2.032699
H	-5.582069	2.026520	-0.743341
B	-2.814829	3.907810	1.384099
B	-2.882809	2.310660	2.167659
B	-3.085789	2.667660	-1.204101
B	-3.175670	1.112780	-0.409991
H	-3.002079	2.756940	-2.389501
H	-2.657239	2.190710	3.335919
H	-2.545309	4.879670	2.024999
C	-1.722399	2.020670	-0.351001
C	-1.575359	2.770270	1.169369
B	-3.796729	3.857870	-0.091431
B	-2.023339	3.702560	-0.202451
H	-1.224059	4.467310	-0.642071
H	-4.323419	4.847990	-0.509661
B	-3.934970	1.267240	1.174609
H	-4.545630	0.362970	1.662199
B	-2.164870	1.153440	1.033079
H	-1.548860	0.127560	1.621109
C	-0.332690	1.817309	-1.533251
O	0.669231	2.431039	-1.158591
O	-0.738790	1.209640	-2.524421
C	-0.188739	2.998889	1.745899
H	-0.114349	4.044509	2.081619
H	-0.019659	2.335779	2.607069
H	0.571871	2.807649	0.976079
Ru	-0.773730	-0.297040	0.138059
O	-0.402740	-2.290871	0.653259
C	0.280250	-2.517631	-0.446531
O	0.359510	-1.525441	-1.247181
C	0.926299	-3.834351	-0.688401

H	0.438229	-4.623471	-0.097281
H	0.903299	-4.077271	-1.762061
H	1.988229	-3.736571	-0.371721
Se	-2.819650	-0.684070	-1.262941
C	-4.111060	-1.708290	-0.226031
C	-5.467920	-1.590270	-0.563071
C	-3.690570	-2.568160	0.796919
C	-6.419200	-2.325709	0.156929
H	-5.782220	-0.912679	-1.363361
C	-4.651671	-3.305040	1.505459
H	-2.627600	-2.653130	1.039009
C	-6.012691	-3.182219	1.190969
H	-7.480670	-2.224879	-0.090781
H	-4.331221	-3.974530	2.310429
H	-6.758411	-3.754369	1.751789
Br	3.937810	-2.206431	0.380889
Cs	2.938210	0.393159	-1.830771
O	1.085640	0.260789	1.079999
C	1.590240	-0.264271	2.107009
O	1.103830	-1.345011	2.695029
C	2.789560	0.329389	2.775339
H	3.642690	-0.344501	2.580139
H	3.005540	1.323549	2.361789
H	2.629470	0.378999	3.864789
H	0.450280	-1.779841	2.068129
O	5.086820	1.455888	0.087199
C	5.925560	1.127298	0.931749
O	6.080490	-0.110082	1.414579
H	5.399440	-0.746162	0.987289
C	6.911521	2.097798	1.552759
H	7.944180	1.773908	1.339079
H	6.746701	3.107648	1.152819
H	6.797541	2.103458	2.649929

Supporting Information: 035-Selenylated-Cluster-RuII-2HOAc-1OAc-CsBr-Decarboxylation-TS-20-SP-TFE-PBE.log

----

Using Gaussian 16: ES64L-G16RevA.03 25-Dec-2016

==

# pbepbe/Def2QZVP/auto empiricaldispersion=gd3bj
scf=(maxcycle=300,direct,vshift=200,tight,yqc) density=current
scrf=(solvent=2,2,2-Trifluoroethanol,smd)

Pointgroup= C1 Stoichiometry= C16H28B10BrCsO8RuSe C1[X(C16H28B10BrCsO8RuSe)] #Atoms= 66

Charge = 0 Multiplicity = 1

SCF Energy= -6566.48078486

==

Optimization incomplete.

Atomic Type	Coordinates (Angstroms) X	Y	Z
B	-3.855764	2.734296	0.020490
B	-3.973234	2.924965	1.787590
H	-4.973455	3.293564	2.331810
H	-4.764504	2.942475	-0.731210
B	-2.378645	3.423147	2.399480
B	-2.904653	1.729007	2.573580
B	-2.164855	3.080538	-0.454500
B	-2.708283	1.431627	-0.263970
H	-1.764155	3.486818	-1.500470
H	-3.012472	1.229267	3.654330
H	-2.132616	4.078748	3.367580
C	-1.194393	1.909839	0.382880
C	-1.326863	2.140599	2.064240
B	-2.937976	4.031787	0.831280
B	-1.243785	3.501239	1.020880
H	-0.247676	4.154360	1.027700
H	-3.170957	5.195206	0.675250
B	-3.815692	1.292356	1.101260
H	-4.668511	0.455295	1.126810
B	-2.109292	0.818778	1.278830
H	-1.791800	-0.433962	1.562740
C	0.454767	1.793641	-0.550820
O	1.395387	2.019752	0.228230
O	0.232627	1.710140	-1.747430
C	-0.122043	1.862380	2.946730
H	0.037296	2.728330	3.607340
H	-0.301382	0.971060	3.566040
H	0.772307	1.704081	2.329590
Ru	-0.719740	-0.509281	0.192780
O	-0.711148	-2.596391	0.017260
C	0.208942	-2.577230	-0.920700
O	0.574841	-1.405379	-1.285810
C	0.806524	-3.829139	-1.439590
H	0.137035	-4.685560	-1.270970
H	1.054464	-3.725729	-2.507420
H	1.757994	-3.979118	-0.864460
Se	-2.410831	-0.047053	-1.604970

C	-4.021980	-1.068365	-1.216880
C	-5.242870	-0.589766	-1.715470
C	-3.951858	-2.266415	-0.494450
C	-6.416339	-1.312207	-1.459950
H	-5.281741	0.349164	-2.276920
C	-5.132757	-2.984326	-0.251750
H	-2.988018	-2.627823	-0.125550
C	-6.362568	-2.507977	-0.728200
H	-7.374360	-0.935769	-1.832340
H	-5.087376	-3.919166	0.316380
H	-7.281177	-3.069108	-0.530120
Br	3.580433	-3.297916	0.703420
Cs	3.567339	-0.198456	-0.904420
O	0.903030	-0.680139	1.600480
C	1.068801	-1.601699	2.444540
O	0.310042	-2.683449	2.512030
C	2.169221	-1.538987	3.450790
H	3.012302	-2.099816	2.988810
H	2.469110	-0.497827	3.636440
H	1.877102	-2.045888	4.382690
H	-0.213608	-2.755900	1.657970
O	4.410406	2.763045	-1.181590
C	4.048274	3.920005	-0.959410
O	2.886244	4.238704	-0.364850
H	2.388365	3.386623	-0.172320
C	4.849423	5.148886	-1.327370
H	4.978452	5.799536	-0.446730
H	5.828563	4.851577	-1.726140
H	4.302622	5.735405	-2.085550

Supporting Information: 035-Selenylated-Cluster-RuII-2HOAc-1OAc-CsBr-Decarboxylation-TS-21-SP-TFE-PBE.log

----

Using Gaussian 16: ES64L-G16RevA.03 25-Dec-2016

==

# pbepbe/Def2QZVP/auto empiricaldispersion=gd3bj
scf=(maxcycle=300,direct,vshift=200,tight,yqc) density=current
scrf=(solvent=2,2,2-Trifluoroethanol,smd)

Pointgroup= C1 Stoichiometry= C16H28B10BrCsO8RuSe C1[X(C16H28B10BrCsO8RuSe)] #Atoms= 66
Charge = 0 Multiplicity = 1

SCF Energy= -6566.48272790

==

Optimization incomplete.

Atomic Type	Coordinates (Angstroms) X	Y	Z
B	4.324941	1.853328	0.312560
B	4.467541	2.519278	-1.335370
H	5.523071	2.720298	-1.862550
H	5.266800	1.549988	0.987270
B	3.097061	3.620149	-1.607490
B	3.063331	1.980289	-2.298740
B	2.839971	2.524749	1.046740
B	2.830500	0.921489	0.346400
H	2.602941	2.700709	2.201030
H	2.989401	1.814059	-3.480660
H	3.049552	4.574689	-2.324700
C	1.533741	2.000110	0.033540
C	1.700691	2.660770	-1.526430
B	3.848861	3.543809	-0.002980
B	2.072301	3.616699	-0.146190
H	1.324552	4.499250	0.136240
H	4.436972	4.486318	0.442450
B	3.819030	0.880929	-1.114620
H	4.366740	-0.121562	-1.465440
B	2.048230	0.994189	-1.231040
H	1.409960	0.006940	-1.868750
C	0.003391	2.011090	0.987500
O	-0.889899	2.670861	0.445600
O	0.210310	1.443240	2.063790
C	0.450881	3.027030	-2.307870
H	0.554562	4.059170	-2.675880
H	0.327991	2.352950	-3.168240
H	-0.429379	2.957781	-1.653250
Ru	0.378410	-0.239040	-0.517120
O	-0.167821	-2.190140	-1.035720
C	-1.053781	-2.288939	-0.077860
O	-1.109001	-1.261459	0.697220
C	-1.965582	-3.446689	0.031640
H	-1.566512	-4.321699	-0.501630
H	-2.171682	-3.682789	1.087450
H	-2.925662	-3.115468	-0.449140
Se	2.129539	-0.765621	1.212800
C	3.421939	-1.992751	0.427690
C	4.711119	-2.029572	0.980140
C	3.061158	-2.841861	-0.626510
C	5.660068	-2.912472	0.447080

H	4.979459	-1.359322	1.803030
C	4.017238	-3.727392	-1.146400
H	2.049298	-2.803381	-1.038830
C	5.314698	-3.760882	-0.615460
H	6.672168	-2.933883	0.863520
H	3.744048	-4.390081	-1.974050
H	6.058398	-4.448973	-1.029440
Br	-4.436251	-1.442817	-1.505620
Cs	-3.561560	1.007342	0.844760
O	-1.216250	0.481121	-1.774730
C	-1.602360	-0.021339	-2.863890
O	-1.151531	-1.168629	-3.346410
C	-2.623480	0.660732	-3.712880
H	-3.596440	0.223582	-3.398060
H	-2.622259	1.744302	-3.526960
H	-2.460950	0.436532	-4.777740
H	-0.673131	-1.654429	-2.611890
O	-2.441610	0.107792	3.563530
C	-1.379530	-0.293199	4.037580
O	-0.586391	-1.194559	3.421750
H	-0.935381	-1.305149	2.494390
C	-0.788780	0.178541	5.342900
H	-1.573450	0.610161	5.979570
H	-0.265491	-0.636909	5.865520
H	-0.045100	0.961140	5.108400

Supporting Information: 035-Selenylated-Cluster-RuII-2HOAc-1OAc-CsBr-Decarboxylation-TS-22-SP-TFE-PBE.log

----

Using Gaussian 16: ES64L-G16RevA.03 25-Dec-2016

===

# pbepbe/Def2QZVP/auto empiricaldispersion=gd3bj
scf=(maxcycle=300,direct,vshift=200,tight,yqc) density=current
scrf=(solvent=2,2,2-Trifluoroethanol,smd)

Pointgroup= C1 Stoichiometry= C16H28B10BrCsO8RuSe C1[X(C16H28B10BrCsO8RuSe)]
#Atoms= 66
Charge = 0 Multiplicity = 1

SCF Energy= -6566.47188565

===

Optimization incomplete.

Atomic Coordinates (Angstroms)
Type X Y Z

B	-1.394957	3.474881	-1.486281
B	-1.785986	4.736281	-0.290371
H	-2.414405	5.719672	-0.559701
H	-1.728447	3.516761	-2.635311
B	-0.469016	4.777100	0.899459
B	-1.984056	3.952881	1.290209
B	0.161303	2.742610	-1.021971
B	-1.369988	1.914071	-0.642171
H	0.958742	2.228949	-1.741681
H	-2.646006	4.301002	2.223739
H	-0.089465	5.689590	1.573049
C	-0.058508	2.033580	0.540239
C	-0.463467	3.228160	1.640699
B	-0.098536	4.492660	-0.814601
B	0.733403	3.562919	0.431249
H	1.863623	3.636329	0.786389
H	0.516274	5.288790	-1.463041
B	-2.563827	3.134362	-0.171521
H	-3.731487	2.962142	-0.364961
B	-1.711708	2.215891	1.091139
H	-2.143888	1.432061	1.870749
C	1.221421	0.895369	1.139849
O	0.899711	0.088369	2.103569
O	2.363992	1.297608	0.841439
C	0.004323	3.038750	3.076459
H	-0.618237	3.657410	3.740539
H	-0.086098	1.982490	3.370139
H	1.055473	3.347389	3.181229
Ru	-0.232609	-0.463730	0.305729
O	-1.461241	-3.158829	-0.919471
C	-0.309891	-3.295120	-0.434461
O	0.246159	-2.466780	0.406929
C	0.560548	-4.472911	-0.829531
H	-0.053553	-5.294010	-1.226281
H	1.251918	-4.125501	-1.618691
H	1.161348	-4.828521	0.023879
Se	-1.722299	0.125001	-1.574281
C	-3.558389	-0.355348	-1.134441
C	-4.089310	-1.367867	-1.953141
C	-4.322519	0.178933	-0.086441
C	-5.382040	-1.851276	-1.713691
H	-3.483980	-1.791078	-2.760891
C	-5.621589	-0.300666	0.135079
H	-3.914998	0.955053	0.560069
C	-6.153040	-1.317076	-0.671551

H	-5.786531	-2.646466	-2.348061
H	-6.220089	0.128694	0.946079
H	-7.165770	-1.690565	-0.490011
O	-1.725130	-0.746639	1.813049
C	-2.638950	-1.604578	1.914439
O	-2.934651	-2.548808	1.057569
C	-3.544210	-1.570868	3.120629
H	-4.552290	-1.262717	2.792489
H	-3.633341	-2.578188	3.558439
H	-3.163610	-0.859638	3.865819
H	-2.314881	-2.587219	0.214509
Cs	3.383750	-1.695042	1.157509
Br	1.455330	-0.594001	-1.663961
O	5.096051	0.168146	-0.555831
C	4.924102	1.120607	-1.323521
O	3.880752	1.954897	-1.283341
H	3.236032	1.656428	-0.566831
C	5.874272	1.466996	-2.451811
H	6.829532	0.941465	-2.314801
H	5.423282	1.153836	-3.410001
H	6.036223	2.555196	-2.507751

Supporting Information: 035-Selenylated-Cluster-RuII-2HOAc-1OAc-CsBr-Decarboxylation-TS-24-SP-TFE-PBE.log

----

Using Gaussian 16: ES64L-G16RevA.03 25-Dec-2016

==

# pbepbe/Def2QZVP/auto empiricaldispersion=gd3bj
scf=(maxcycle=300,direct,vshift=200,tight,yqc) density=current
scrf=(solvent=2,2,2-Trifluoroethanol,smd)

Pointgroup= C1 Stoichiometry= C16H28B10BrCsO8RuSe C1[X(C16H28B10BrCsO8RuSe)] #Atoms= 66
Charge = 0 Multiplicity = 1

SCF Energy= -6566.48031955

==

Optimization incomplete.

Atomic Type	Coordinates (Angstroms) X	Y	Z
B	4.175081	1.859728	-0.651310
B	3.977902	3.624808	-0.764550
H	4.793252	4.344987	-1.267080

H	5.124451	1.258267	-1.068310
B	3.043742	4.159258	0.648570
B	2.242632	3.975659	-0.917330
B	3.345801	1.317188	0.840620
B	2.561481	1.154649	-0.716120
H	3.611310	0.357008	1.495050
H	1.712143	4.911709	-1.439570
H	3.056693	5.225938	1.189330
C	1.679251	1.717409	0.639370
C	1.516392	3.378929	0.525090
B	4.232802	2.853428	0.830400
B	2.659642	2.743929	1.640420
H	2.415772	2.815039	2.802030
H	5.224342	2.990557	1.488100
B	2.928031	2.552118	-1.733250
H	2.962681	2.460198	-2.926810
B	1.356011	2.445049	-0.901630
H	0.273751	2.348480	-1.384270
Cs	-3.828431	-1.544048	-0.575690
C	0.259052	4.042420	1.067120
H	-0.620238	3.762460	0.467990
H	0.085172	3.737820	2.107440
H	0.393003	5.133900	1.016240
Ru	0.011200	-0.207970	0.136020
Se	1.950520	-0.658951	-1.338310
C	3.294939	-1.819662	-0.532350
C	4.386399	-2.161742	-1.346130
C	3.153799	-2.373362	0.748820
C	5.353668	-3.056703	-0.866880
H	4.482549	-1.726582	-2.346190
C	4.121748	-3.277272	1.213580
H	2.323939	-2.068231	1.389040
C	5.219368	-3.620363	0.410200
H	6.210988	-3.315073	-1.496970
H	4.019748	-3.702312	2.217810
H	5.973088	-4.322433	0.781060
Br	-1.220880	0.202141	-2.087240
O	-1.777471	-1.063409	2.942950
C	-2.190380	-0.028189	2.353650
O	-1.852930	0.328831	1.147240
C	-3.171550	0.887212	3.056850
H	-2.628689	1.808122	3.332250
H	-3.994519	1.169352	2.379020
H	-3.561050	0.411602	3.967740
O	1.074550	-0.429840	2.023410
C	0.680090	0.780040	2.023440

O	0.152131	1.553540	2.803060
O	-0.540871	-2.220370	-0.071650
C	-0.333312	-3.110700	0.815680
O	-0.392712	-2.927630	2.096390
H	-0.849571	-1.978919	2.375440
C	0.069078	-4.487350	0.355850
H	1.174927	-4.513611	0.327430
H	-0.277213	-5.261390	1.056660
H	-0.295703	-4.677690	-0.664070
O	-4.608879	1.378503	-0.220990
C	-3.958699	2.422872	-0.364050
O	-2.745409	2.621302	0.156960
H	-2.417649	1.746391	0.568300
C	-4.440288	3.610093	-1.166560
H	-3.901428	3.626252	-2.130570
H	-5.519558	3.529053	-1.355960
H	-4.208397	4.553522	-0.647310

Supporting Information: 035-Selenylated-Cluster-RuII-2HOAc-1OAc-CsBr-Decarboxylation-TS-25-SP-TFE-PBE.log

----

Using Gaussian 16: ES64L-G16RevA.03 25-Dec-2016

==

# pbepbe/Def2QZVP/auto empiricaldispersion=gd3bj
scf=(maxcycle=300,direct,vshift=200,tight,yqc) density=current
scrf=(solvent=2,2,2-Trifluoroethanol,smd)

Pointgroup= C1 Stoichiometry= C16H28B10BrCsO8RuSe C1[X(C16H28B10BrCsO8RuSe)]
#Atoms= 66
Charge = 0 Multiplicity = 1

SCF Energy= -6566.48431806

==

Optimization incomplete.

Atomic Type	Coordinates (Angstroms) X	Y	Z
B	-4.732810	1.955020	-0.076231
B	-4.397780	2.877780	1.414489
H	-5.264100	3.257549	2.148189
H	-5.834820	1.645029	-0.428181
B	-2.948540	3.873620	1.136669
B	-2.817810	2.360690	2.070919
B	-3.460710	2.363020	-1.264721

B	-3.344900	0.897440	-0.320081
H	-3.528370	2.333680	-2.454131
H	-2.445120	2.377680	3.207229
H	-2.668020	4.917150	1.647449
C	-1.960670	1.885400	-0.530971
C	-1.679120	2.781080	0.870459
B	-4.104560	3.622500	-0.187431
B	-2.355800	3.551910	-0.513811
H	-1.666700	4.308640	-1.122661
H	-4.742850	4.536270	-0.623741
B	-3.919920	1.171040	1.324529
H	-4.408270	0.290750	1.969209
B	-2.177120	1.126650	0.971369
H	-1.541829	0.111950	1.590889
C	-0.705360	1.633950	-1.876211
O	0.299270	2.319210	-1.675821
O	-1.213330	0.935150	-2.748071
C	-0.245670	3.119360	1.245829
H	-0.261820	3.947790	1.970419
H	0.254340	2.248420	1.696889
H	0.307280	3.423900	0.346539
Ru	-0.752009	-0.264000	0.137199
Se	-2.899249	-0.956250	-1.001191
C	-3.909839	-1.979570	0.309389
C	-5.301529	-2.063521	0.153199
C	-3.252429	-2.648050	1.350519
C	-6.049179	-2.809631	1.074069
H	-5.799189	-1.533251	-0.665131
C	-4.012139	-3.399750	2.260139
H	-2.166559	-2.570730	1.457439
C	-5.405799	-3.477841	2.126899
H	-7.137249	-2.865801	0.968009
H	-3.507519	-3.922030	3.079529
H	-5.992819	-4.060241	2.844029
O	0.696991	-1.030140	-1.257311
C	0.720411	-1.846040	-2.215381
O	1.846721	-2.417800	-2.582011
C	-0.468229	-2.234190	-3.042711
H	-1.141939	-2.864510	-2.437181
H	-1.019169	-1.324260	-3.326571
H	-0.149629	-2.798970	-3.929451
H	2.616941	-2.193460	-1.928471
O	1.111771	0.069670	1.264589
C	1.074491	-1.166350	1.615449
C	2.196211	-1.838330	2.334629
H	2.939511	-2.151040	1.572439

H	2.689691	-1.136520	3.023989
H	1.837671	-2.728470	2.872559
O	0.024371	-1.835890	1.253599
Cs	3.108810	1.421690	-0.836331
Br	4.364091	-1.914120	-0.782301
O	5.050840	1.227000	1.523819
C	5.544150	0.395740	2.289189
O	5.602551	-0.921890	2.046239
H	5.208061	-1.120660	1.135359
C	6.170440	0.744260	3.623639
H	7.257060	0.555460	3.582899
H	5.991140	1.802820	3.856019
H	5.761251	0.102690	4.421279

Supporting Information: 035-Selenylated-Cluster-RuII-2HOAc-1OAc-CsBr-Decarboxylation-TS-26-SP-TFE-PBE.log

----
Using Gaussian 16: ES64L-G16RevA.03 25-Dec-2016

==

\# pbepbe/Def2QZVP/auto empiricaldispersion=gd3bj
scf=(maxcycle=300,direct,vshift=200,tight,yqc) density=current
scrf=(solvent=2,2,2-Trifluoroethanol,smd)

Pointgroup= C1 Stoichiometry= C16H28B10BrCsO8RuSe C1[X(C16H28B10BrCsO8RuSe)] #Atoms= 66
Charge = 0 Multiplicity = 1

SCF Energy= -6566.48243957

==

Optimization incomplete.

Atomic Type	Coordinates (Angstroms) X	Y	Z
B	3.743558	2.812101	0.369451
B	3.455858	3.507431	-1.247539
H	4.293978	4.117201	-1.846489
H	4.787918	2.894642	0.949661
B	1.745088	3.992260	-1.349519
B	2.286949	2.477811	-2.123609
B	2.187968	2.835341	1.253421
B	2.733299	1.374291	0.471081
H	2.015508	2.885060	2.431481
H	2.172559	2.324361	-3.303989
H	1.270508	4.867490	-2.010749

C	1.098589	1.859800	0.309931
C	0.848249	2.565390	-1.204649
B	2.619048	4.186311	0.183461
B	0.949648	3.562680	0.190641
H	-0.044172	4.096290	0.573551
H	2.836967	5.275841	0.628361
B	3.522599	1.741411	-1.062219
H	4.381589	1.038571	-1.506789
B	1.842479	1.160370	-1.028559
H	1.643540	-0.033910	-1.591049
C	-0.304731	1.306969	1.542821
O	-1.400651	1.648819	1.084611
O	0.225979	0.915630	2.562341
C	-0.506001	2.403249	-1.875649
H	-0.549012	3.092569	-2.733019
H	-0.639531	1.371489	-2.234669
H	-1.321101	2.649029	-1.180709
Ru	0.701970	-0.510340	-0.241359
Se	2.708510	-0.470569	1.295351
C	4.179780	-1.246279	0.285691
C	5.488940	-0.907058	0.659021
C	3.929800	-2.153699	-0.752049
C	6.566010	-1.468628	-0.039739
H	5.665640	-0.195758	1.472271
C	5.018121	-2.716028	-1.437089
H	2.902301	-2.407889	-1.028029
C	6.331711	-2.372838	-1.086869
H	7.589920	-1.196247	0.235431
H	4.832691	-3.423858	-2.251579
H	7.175451	-2.810677	-1.629489
O	-0.756440	-1.514871	0.966051
C	-0.708049	-2.339741	1.923031
O	-1.693999	-3.163271	2.156681
C	0.441221	-2.448560	2.884321
H	1.312781	-2.874570	2.357641
H	0.717560	-1.444830	3.241031
H	0.174331	-3.103440	3.724931
H	-2.412569	-3.179821	1.372141
O	-0.898360	-0.851261	-1.730539
C	-0.458860	-2.053211	-1.868509
C	-1.176439	-3.083241	-2.673879
H	-2.018549	-3.459041	-2.050339
H	-1.594579	-2.633701	-3.588499
H	-0.507939	-3.920331	-2.923469
O	0.619551	-2.351330	-1.215449
Cs	-3.613110	0.014528	-0.377919

Br	-3.789379	-3.472302	-0.051779
O	-3.844732	3.136938	-0.382469
C	-3.598022	4.099238	0.348781
O	-2.647122	4.086499	1.300221
H	-2.188732	3.194529	1.269601
C	-4.310963	5.429888	0.280991
H	-5.144593	5.372687	-0.431619
H	-4.679463	5.719058	1.278931
H	-3.602083	6.212328	-0.040489

Supporting Information: 035-Selenylated-Cluster-RuII-2HOAc-1OAc-CsBr-Decarboxylation-TS-27-SP-TFE-PBE.log

----

Using Gaussian 16: ES64L-G16RevA.03 25-Dec-2016

==

# pbepbe/Def2QZVP/auto empiricaldispersion=gd3bj
scf=(maxcycle=300,direct,vshift=200,tight,yqc) density=current
scrf=(solvent=2,2,2-Trifluoroethanol,smd)

Pointgroup= C1 Stoichiometry= C16H28B10BrCsO8RuSe C1[X(C16H28B10BrCsO8RuSe)]
#Atoms= 66
Charge = 0 Multiplicity = 1

SCF Energy= -6566.48450224

==

Optimization incomplete.

Atomic Type	Coordinates (Angstroms) X	Y	Z
B	-0.251327	3.417231	1.668291
B	0.768404	4.586920	0.797991
H	1.189715	5.582800	1.313401
H	-0.569297	3.535541	2.819351
B	0.265914	4.594521	-0.906519
B	1.738963	3.712119	-0.420739
B	-1.360108	2.673362	0.479711
B	0.095451	1.846081	0.964401
H	-2.441748	2.221213	0.699551
H	2.803553	4.013948	-0.871719
H	0.336065	5.495800	-1.688969
C	-0.344219	1.883601	-0.693659
C	0.595393	3.040990	-1.495069
B	-0.959356	4.398302	0.357181
B	-1.041167	3.390702	-1.117579

H	-1.831737	3.442353	-2.008939
H	-1.789905	5.240802	0.544161
B	1.426632	2.976169	1.169931
H	2.316342	2.766099	1.939831
B	1.290632	1.994330	-0.318579
H	2.257401	1.144919	-0.591389
C	-1.517110	0.573702	-1.721059
O	-1.321530	0.664972	-2.907029
O	-2.314210	0.162333	-0.871459
C	0.951302	2.817250	-2.955409
H	0.796693	3.759700	-3.503079
H	2.004292	2.513499	-3.045499
H	0.319372	2.030210	-3.386659
Ru	1.085810	-0.148630	-0.790829
Se	0.148150	-0.091989	1.518541
C	1.558840	-0.145061	2.849791
C	2.455249	-1.223642	2.800051
C	1.623700	0.804699	3.879291
C	3.441668	-1.337102	3.790311
H	2.395948	-1.942851	1.977971
C	2.619960	0.683388	4.858541
H	0.916691	1.638890	3.909601
C	3.528669	-0.384163	4.816051
H	4.148318	-2.173333	3.754421
H	2.685341	1.431408	5.655251
H	4.305149	-0.472773	5.582431
O	1.580100	-0.132161	-2.874499
C	2.214199	-1.023311	-3.488129
C	2.229469	-1.037881	-4.991869
H	1.938420	-0.052671	-5.380999
H	3.220918	-1.332012	-5.369569
H	1.496408	-1.786861	-5.340529
O	2.882428	-2.008062	-2.919699
Cs	-2.908192	-2.291606	0.847161
Br	-0.019113	-2.462999	-1.205999
H	2.786368	-1.921382	-1.903959
O	2.759758	-1.315312	-0.396979
C	3.931489	-0.713863	-0.144629
O	4.133240	0.502107	-0.129069
C	5.032078	-1.718674	0.173971
H	5.113618	-1.801904	1.272491
H	4.817347	-2.717554	-0.235339
H	5.994478	-1.345195	-0.207369
O	-5.207211	-0.300214	0.715121
C	-5.520990	0.761436	0.168751
O	-4.738249	1.418725	-0.698949

H	-3.864149	0.927364	-0.785329
C	-6.827839	1.483927	0.407351
H	-7.302809	1.747138	-0.551749
H	-7.500170	0.856218	1.007621
H	-6.631758	2.429757	0.941461

Supporting Information: 035-Selenylated-Cluster-RuII-2HOAc-1OAc-CsBr-Decarboxylation-TS-28-SP-TFE-PBE.log

----

Using Gaussian 16: ES64L-G16RevA.03 25-Dec-2016

==

# pbepbe/Def2QZVP/auto empiricaldispersion=gd3bj
scf=(maxcycle=300,direct,vshift=200,tight,yqc) density=current
scrf=(solvent=2,2,2-Trifluoroethanol,smd)

Pointgroup= C1 Stoichiometry= C16H28B10BrCsO8RuSe C1[X(C16H28B10BrCsO8RuSe)]
#Atoms= 66
Charge = 0 Multiplicity = 1

SCF Energy= -6566.48633701

==

Optimization incomplete.

Atomic Type	Coordinates (Angstroms) X	Y	Z
B	-2.441686	3.668451	-0.693649
B	-2.450555	4.307041	0.963741
H	-3.231945	5.139072	1.327811
H	-3.216236	4.005482	-1.545029
B	-0.779085	4.327400	1.559261
B	-1.894327	3.028171	2.091701
B	-0.743736	3.236230	-1.095339
B	-1.870427	2.017681	-0.575569
H	-0.257796	3.200319	-2.184809
H	-2.167187	2.903271	3.248131
H	-0.298805	5.067729	2.366081
C	-0.283427	2.022269	0.069011
C	-0.286197	2.718289	1.612101
B	-1.090135	4.692410	-0.142859
B	0.285324	3.628569	0.291501
H	1.452664	3.871148	0.231141
H	-0.882444	5.788220	-0.579689
B	-2.929917	2.611232	0.695071
H	-4.036637	2.195163	0.868941

B	-1.503908	1.615960	1.096311
H	-1.583729	0.409090	1.600411
C	1.604652	1.078328	-1.017039
O	2.495292	1.194797	-0.192949
O	1.222942	0.994208	-2.146169
C	0.760083	2.271258	2.615651
H	1.138854	3.162868	3.140081
H	0.336092	1.566389	3.343241
H	1.588452	1.766548	2.101091
Ru	-0.536049	-0.245600	0.338171
Se	-1.973059	0.335661	-1.679519
C	-3.774750	-0.316938	-1.384059
C	-3.970281	-1.707927	-1.396849
C	-4.869269	0.554733	-1.281529
C	-5.266351	-2.225546	-1.266369
H	-3.115621	-2.381108	-1.505749
C	-6.160549	0.026374	-1.142569
H	-4.719068	1.636583	-1.307179
C	-6.362100	-1.360965	-1.127319
H	-5.417592	-3.310486	-1.275399
H	-7.012059	0.707945	-1.048169
H	-7.372301	-1.767894	-1.018009
O	0.634989	-1.540641	-0.987029
C	0.261568	-2.640471	-1.481049
O	-0.766932	-3.342560	-1.088049
C	1.040658	-3.237672	-2.631439
H	0.373178	-3.335741	-3.504489
H	1.887939	-2.592323	-2.905039
H	1.387217	-4.253992	-2.377169
H	-1.222382	-2.851050	-0.214569
Cs	3.694999	-1.686454	0.371011
Br	0.907760	-1.055882	2.356451
O	-1.609251	-1.995469	0.751461
C	-2.551321	-2.051579	1.701871
O	-2.879670	-1.093308	2.396551
C	-3.212482	-3.418008	1.806651
H	-3.642602	-3.538398	2.811241
H	-4.032222	-3.471197	1.068321
H	-2.503663	-4.235569	1.598581
O	5.563411	0.246804	-1.068259
C	5.724532	1.427344	-1.389979
O	4.794643	2.380535	-1.221529
H	3.976942	1.956666	-0.826219
C	6.983152	1.973883	-2.023209
H	7.745962	1.186102	-2.082749
H	6.756813	2.350753	-3.035219

H 7.363173 2.828313 -1.439109

Supporting Information: 035-Selenylated-Cluster-RuII-2HOAc-1OAc-CsBr-Decarboxylation-TS-29-SP-TFE-PBE.log

----

Using Gaussian 16: ES64L-G16RevA.03 25-Dec-2016

==

pbepbe/Def2QZVP/auto empiricaldispersion=gd3bj scf=(maxcycle=300,direct,vshift=200,tight,yqc) density=current scrf=(solvent=2,2,2-Trifluoroethanol,smd)

Pointgroup= C1 Stoichiometry= C16H28B10BrCsO8RuSe C1[X(C16H28B10BrCsO8RuSe)] #Atoms= 66

Charge = 0 Multiplicity = 1

SCF Energy= -6566.48600641

==

Optimization incomplete.

Atomic Type	Coordinates (Angstroms) X	Y	Z
B	-3.676849	2.903811	-0.285450
B	-3.424229	3.557371	1.353180
H	-4.285659	4.120882	1.965250
H	-4.719459	2.972382	-0.871650
B	-1.726899	4.088540	1.484290
B	-2.235440	2.537361	2.202850
B	-2.120089	3.004171	-1.155530
B	-2.623000	1.497291	-0.430770
H	-1.944529	3.097810	-2.329750
H	-2.123840	2.351291	3.379300
H	-1.281148	4.955720	2.176170
C	-1.001080	2.033010	-0.241280
C	-0.790869	2.692840	1.299960
B	-2.599499	4.307361	-0.047380
B	-0.913289	3.734110	-0.066340
H	0.069071	4.310069	-0.419060
H	-2.850168	5.404001	-0.457270
B	-3.433230	1.794851	1.110310
H	-4.273670	1.052892	1.526350
B	-1.733100	1.268210	1.074230
H	-1.419631	0.128550	1.627110
C	0.494110	1.642659	-1.412420
O	1.551630	1.822679	-0.789370

O	0.053760	1.478919	-2.525990
C	0.557040	2.544509	1.987250
H	0.438901	2.854059	3.037540
H	0.893250	1.496939	1.958710
H	1.313401	3.181839	1.509270
Ru	-0.598141	-0.395090	0.175430
Se	-2.571501	-0.303669	-1.346780
C	-4.071201	-1.113048	-0.406100
C	-5.371571	-0.741358	-0.778460
C	-3.846222	-2.073949	0.587810
C	-6.464442	-1.325197	-0.123570
H	-5.529681	0.013292	-1.555650
C	-4.949292	-2.657338	1.229880
H	-2.824492	-2.351099	0.862300
C	-6.254552	-2.282927	0.880100
H	-7.481521	-1.027347	-0.398110
H	-4.782533	-3.406768	2.010710
H	-7.110462	-2.737257	1.389440
O	1.082529	-0.813461	1.611500
C	0.530678	-1.948811	1.840080
C	1.084808	-2.918321	2.846980
H	0.917588	-2.528091	3.866180
H	0.590757	-3.895541	2.746540
H	2.170898	-3.027312	2.696980
O	-0.547702	-2.232920	1.192370
Cs	3.674229	-0.338192	-0.145090
Br	0.643298	-1.676371	-1.676780
O	1.505937	-4.466471	-0.276390
C	2.734867	-4.418862	0.252890
C	3.172236	-5.772452	0.772760
H	2.551656	-6.051072	1.642470
H	3.019706	-6.548632	0.005510
H	4.227786	-5.735463	1.074610
O	3.422747	-3.396862	0.336440
H	1.248937	-3.552671	-0.630350
O	4.593590	2.610017	0.176740
C	4.177701	3.766667	0.087730
O	2.951501	4.092428	-0.359300
H	2.455541	3.247188	-0.594190
C	4.979662	4.992457	0.465200
H	5.967372	4.693456	0.840880
H	5.095182	5.650617	-0.412500
H	4.442902	5.573267	1.234070

Supporting Information: 035-Selenylated-Cluster-RuII-2HOAc-1OAc-CsBr-Decarboxylation-TS-30-SP-TFE-PBE.log

----

Using Gaussian 16: ES64L-G16RevA.03 25-Dec-2016

==

# pbepbe/Def2QZVP/auto empiricaldispersion=gd3bj
scf=(maxcycle=300,direct,vshift=200,tight,yqc) density=current
scrf=(solvent=2,2,2-Trifluoroethanol,smd)

Pointgroup= C1 Stoichiometry= C16H28B10BrCsO8RuSe C1[X(C16H28B10BrCsO8RuSe)]
#Atoms= 66
Charge = 0 Multiplicity = 1

SCF Energy= -6566.48502260

==

Optimization incomplete.

Atomic Type	Coordinates (Angstroms) X	Y	Z
B	3.104661	2.882480	1.455360
B	3.006082	4.002040	0.073170
H	3.831352	4.843979	-0.137500
H	4.001881	2.888699	2.249350
B	1.283942	4.321360	-0.262890
B	2.196311	3.175550	-1.281890
B	1.433981	2.483590	1.950770
B	2.336211	1.383550	0.939440
H	1.050471	2.157390	3.030500
H	2.320321	3.377240	-2.454410
H	0.799882	5.303320	-0.743330
C	0.696241	1.732860	0.566470
C	0.618721	2.835290	-0.714200
B	1.829772	4.131950	1.416910
B	0.307711	3.369130	0.892050
H	-0.811829	3.663701	1.176430
H	1.792082	5.054080	2.179620
B	3.316491	2.275700	-0.226430
H	4.342641	1.830839	-0.649570
B	1.765051	1.552060	-0.720130
H	1.739091	0.631030	-1.638860
C	-0.900179	0.793161	1.204640
O	-1.837789	1.066851	0.445710
O	-0.639309	0.319111	2.286080
C	-0.539419	2.744490	-1.694690
H	-0.280349	3.340390	-2.583930
H	-0.708419	1.700681	-2.000250

H	-1.460779	3.145341	-1.250720
Ru	0.823620	-0.450130	-0.606270
Se	2.480490	-0.607910	1.246290
C	4.237750	-0.861371	0.447630
C	5.367120	-0.482841	1.188480
C	4.360910	-1.442381	-0.820720
C	6.641600	-0.666941	0.635330
H	5.252441	-0.027211	2.177320
C	5.643110	-1.628781	-1.359760
H	3.467300	-1.732561	-1.380360
C	6.780590	-1.239441	-0.638080
H	7.526540	-0.358692	1.201380
H	5.748980	-2.078631	-2.352520
H	7.777570	-1.381752	-1.067270
O	-0.537650	-0.651570	-2.394730
C	0.257450	-1.564850	-2.819150
C	-0.011210	-2.336620	-4.083040
H	-0.561260	-3.262070	-3.834030
H	-0.618100	-1.737300	-4.778830
H	0.936410	-2.629840	-4.560390
O	1.299190	-1.838890	-2.112950
Cs	-3.445930	-1.072959	-1.039610
Br	-0.389230	-2.476180	0.407400
H	-1.534880	-2.118959	2.378550
O	-2.029360	-2.159919	3.251350
C	-3.326730	-1.911989	3.029950
C	-4.109130	-1.842239	4.324310
H	-3.805680	-2.648599	5.010640
H	-3.887670	-0.883959	4.826260
H	-5.186330	-1.898038	4.115060
O	-3.826700	-1.742649	1.915360
O	-4.670349	1.771991	-1.078900
C	-4.539099	2.867831	-0.531940
O	-3.553539	3.162831	0.336450
H	-2.960939	2.356641	0.440670
C	-5.459798	4.047562	-0.752630
H	-6.257329	3.773892	-1.456280
H	-5.897818	4.368882	0.207280
H	-4.886958	4.903642	-1.147460

Supporting Information: 035-Selenylated-Cluster-RuII-2HOAc-1OAc-CsBr-Decarboxylation-TS-31-SP-TFE-PBE.log

----

Using Gaussian 16: ES64L-G16RevA.03 25-Dec-2016

==

# pbepbe/Def2QZVP/auto empiricaldispersion=gd3bj
scf=(maxcycle=300,direct,vshift=200,tight,yqc) density=current

scrf=(solvent=2,2,2-Trifluoroethanol,smd)

Pointgroup= C1 Stoichiometry= C16H28B10BrCsO8RuSe C1[X(C16H28B10BrCsO8RuSe)]
#Atoms= 66
Charge = 0 Multiplicity = 1

SCF Energy= -6566.48497596
==
Optimization incomplete.

Atomic Coordinates (Angstroms)
Type X Y Z

B	3.468102	3.117888	0.219780
B	3.675633	3.451168	-1.520200
H	4.642233	4.002767	-1.962870
H	4.281413	3.409948	1.049900
B	2.065323	3.743279	-2.224690
B	2.863922	2.166118	-2.451790
B	1.717852	3.174399	0.576690
B	2.515002	1.638929	0.332120
H	1.191543	3.439089	1.612510
H	3.122562	1.771058	-3.551110
H	1.791253	4.417619	-3.173340
C	0.994442	1.936430	-0.405530
C	1.210892	2.290519	-2.035930
B	2.422033	4.322189	-0.584200
B	0.850623	3.560930	-0.931770
H	-0.231677	4.056740	-0.984450
H	2.466784	5.494549	-0.344150
B	3.729062	1.770858	-0.943610
H	4.704161	1.078847	-0.952590
B	2.136141	1.050539	-1.279580
H	2.094561	-0.180911	-1.716060
C	-0.725878	1.519080	0.325600
O	-1.561978	1.525101	-0.574570
O	-0.602239	1.482860	1.538090
C	0.141752	1.857730	-3.026760
H	0.415752	2.243930	-4.020790
H	0.080151	0.758280	-3.068210
H	-0.838478	2.253211	-2.730040
Ru	0.975040	-0.651820	-0.478770
Se	2.336141	0.000639	1.507290
C	4.136000	-0.692602	1.248860

C	5.196151	-0.064263	1.919060
C	4.350280	-1.819432	0.445310
C	6.497060	-0.560354	1.759300
H	5.011861	0.817857	2.540670
C	5.656449	-2.311463	0.300250
H	3.510249	-2.293652	-0.069500
C	6.728240	-1.683584	0.950850
H	7.330991	-0.064534	2.266550
H	5.833659	-3.189233	-0.330060
H	7.745679	-2.068564	0.828270
O	-0.161070	-1.568000	-2.198070
C	0.590029	-2.599310	-2.061980
C	0.413848	-3.839210	-2.895020
H	-0.280432	-4.527320	-2.380000
H	-0.006901	-3.583370	-3.879500
H	1.376228	-4.359571	-3.015400
O	1.504879	-2.563511	-1.154250
Cs	-3.182990	-0.962788	-1.187840
Br	-0.554510	-1.994700	1.104330
H	-1.614010	-0.765689	2.612020
O	-2.055299	-0.245469	3.359650
C	-3.173169	0.306302	2.917190
C	-3.743969	1.323482	3.873130
H	-3.532569	1.044562	4.916100
H	-3.239388	2.286152	3.674460
H	-4.824159	1.452743	3.714450
O	-3.685299	0.032182	1.813240
O	-6.003569	0.136653	-0.656090
C	-6.565419	0.869244	0.163130
O	-6.041489	1.210673	1.347580
H	-5.132239	0.762073	1.459050
C	-7.928309	1.491415	-0.055880
H	-7.848828	2.591265	-0.022730
H	-8.333379	1.176075	-1.026900
H	-8.615599	1.192985	0.753540

Supporting Information: 035-Selenylated-Cluster-RuII-2HOAc-1OAc-CsBr-Decarboxylation-TS-32-SP-TFE-PBE.log

----

Using Gaussian 16: ES64L-G16RevA.03 25-Dec-2016

==

# pbepbe/Def2QZVP/auto empiricaldispersion=gd3bj
scf=(maxcycle=300,direct,vshift=200,tight,yqc) density=current
scrf=(solvent=2,2,2-Trifluoroethanol,smd)

Pointgroup= C1 Stoichiometry= C16H28B10BrCsO8RuSe C1[X(C16H28B10BrCsO8RuSe)] #Atoms= 66

Charge = 0   Multiplicity = 1

SCF Energy= -6566.48713719

===

Optimization incomplete.

Atomic Type	Coordinates (Angstroms) X	Y	Z
B	4.088349	2.305151	-0.273010
B	3.714049	2.921431	-1.904360
H	4.563269	3.231611	-2.689760
H	5.204249	2.139671	0.130230
B	2.194589	3.838551	-1.810950
B	2.185919	2.180661	-2.463550
B	2.771099	2.820911	0.819490
B	2.776279	1.210421	0.150750
H	2.831699	3.009861	1.994650
H	1.830619	1.973190	-3.587580
H	1.843439	4.753720	-2.495570
C	1.322749	2.096640	0.180360
C	1.002429	2.721800	-1.358500
B	3.338939	3.913121	-0.460540
B	1.591779	3.760730	-0.129090
H	0.839899	4.565990	0.326040
H	3.898069	4.937261	-0.190830
B	3.357539	1.226891	-1.516390
H	3.923760	0.286151	-1.989770
B	1.614109	1.105930	-1.168230
H	1.033960	-0.053040	-1.646240
C	0.055019	2.039830	1.674510
O	-1.000211	2.561520	1.339220
O	0.669779	1.589700	2.624540
C	-0.441761	2.943090	-1.776200
H	-0.583601	4.006450	-2.022240
H	-0.681001	2.342450	-2.665420
H	-1.116321	2.667370	-0.956080
Ru	0.342580	-0.262620	-0.123730
Se	2.540200	-0.511429	1.174470
C	3.937730	-1.515959	0.260060
C	5.258220	-1.263099	0.664110
C	3.654150	-2.460839	-0.733100
C	6.309840	-1.953389	0.046410
H	5.464320	-0.518439	1.439580

C	4.715180	-3.151349	-1.338260
H	2.619360	-2.648439	-1.031770
C	6.039760	-2.898329	-0.954650
H	7.341970	-1.748098	0.348360
H	4.499061	-3.887839	-2.119290
H	6.862990	-3.436248	-1.435670
Br	0.020730	-2.669590	-0.829290
O	-0.764700	-0.730070	1.710560
C	-0.440320	-1.576390	2.576380
O	0.199900	-2.701190	2.316540
H	0.286570	-2.788020	1.302920
C	-0.753280	-1.334170	4.026680
H	-0.334640	-0.349960	4.297460
H	-1.846590	-1.288580	4.170190
H	-0.336610	-2.124040	4.666260
O	-1.557340	0.247170	-0.814110
C	-2.221380	0.126370	-1.921960
C	-1.560040	-0.468570	-3.149020
H	-2.243330	-0.426120	-4.009040
H	-0.630750	0.078160	-3.386860
H	-1.265890	-1.511330	-2.945500
O	-3.419240	0.536039	-1.961360
Cs	-3.695461	1.032269	1.180530
O	-5.725370	-0.828631	-0.164970
C	-6.034660	-1.346841	-1.243530
O	-5.417480	-1.116081	-2.405790
H	-4.643210	-0.463101	-2.263670
C	-7.172580	-2.336692	-1.397460
H	-6.775020	-3.313961	-1.720830
H	-7.703760	-2.451592	-0.442760
H	-7.870330	-1.997622	-2.181110

Supporting Information: 035-Selenylated-Cluster-RuII-2HOAc-1OAc-CsBr-Decarboxylation-TS-33-SP-TFE-PBE.log

----

Using Gaussian 16: ES64L-G16RevA.03 25-Dec-2016

==

# pbepbe/Def2QZVP/auto empiricaldispersion=gd3bj
scf=(maxcycle=300,direct,vshift=200,tight,yqc) density=current
scrf=(solvent=2,2,2-Trifluoroethanol,smd)

Pointgroup= C1 Stoichiometry= C16H28B10BrCsO8RuSe C1[X(C16H28B10BrCsO8RuSe)]
#Atoms= 66
Charge = 0 Multiplicity = 1

SCF Energy= -6566.48865974

==

Optimization incomplete.

Atomic Type	Coordinates (Angstroms) X	Y	Z
B	-4.844290	0.390444	0.294111
B	-5.316981	-0.599206	-1.113799
H	-6.243461	-0.319985	-1.819629
H	-5.404950	1.401314	0.608841
B	-4.939882	-2.296006	-0.746309
B	-3.852182	-1.360257	-1.799829
B	-4.148351	-0.706627	1.521751
B	-3.100830	0.208643	0.460581
H	-4.119281	-0.561567	2.703971
H	-3.654642	-1.700387	-2.929629
H	-5.481113	-3.276316	-1.165139
C	-2.769992	-1.437808	0.764541
C	-3.253742	-2.374497	-0.565249
B	-5.533911	-1.236566	0.544981
B	-4.196602	-2.370767	0.875361
H	-4.185703	-3.355027	1.546691
H	-6.603312	-1.428735	1.048801
B	-3.790040	0.300883	-1.160789
H	-3.573140	1.232363	-1.877799
B	-2.473701	-0.845578	-0.800459
H	-1.357331	-0.627659	-1.553279
C	-1.573342	-1.974798	2.011061
O	-1.210693	-3.137949	1.853531
O	-1.461711	-1.049258	2.822201
C	-2.504683	-3.658068	-0.880799
H	-1.848703	-3.914998	-0.036369
H	-3.240324	-4.461357	-1.040209
H	-1.892703	-3.532318	-1.784129
Ru	-0.415791	-0.649339	-0.155319
Se	-1.507280	1.346232	0.973401
C	-1.902109	2.852372	-0.201839
C	-2.782868	3.818472	0.309711
C	-1.327789	3.002661	-1.469869
C	-3.104647	4.939183	-0.468089
H	-3.226918	3.687193	1.301891
C	-1.649818	4.132642	-2.236269
H	-0.644839	2.242381	-1.856779
C	-2.537187	5.098622	-1.740899
H	-3.802037	5.686963	-0.076709

H	-1.206658	4.250401	-3.230769
H	-2.788947	5.975122	-2.346619
Br	1.244320	0.434990	-1.719139
O	1.214319	-0.813240	1.307351
C	1.939969	-0.084591	2.185931
O	3.174039	-0.143032	2.174911
C	1.138680	0.732190	3.160851
H	0.234700	0.182110	3.465501
H	1.771460	0.983029	4.024791
H	0.816681	1.666850	2.670991
O	0.254868	-2.513840	-0.941009
C	1.380227	-3.060910	-0.712439
C	1.613596	-4.420270	-1.340499
H	1.261206	-5.193920	-0.635109
H	1.043626	-4.521950	-2.275629
H	2.686306	-4.591321	-1.520559
O	2.316778	-2.573731	0.028531
H	1.820618	-1.592961	0.747091
Cs	4.660019	-0.608223	-0.510599
O	4.042671	2.334388	-0.033009
C	3.193741	2.820838	0.715961
O	1.867581	2.721379	0.502461
H	1.720971	2.122759	-0.293779
C	3.510922	3.541968	2.004091
H	4.491182	4.033938	1.927261
H	2.728912	4.268919	2.268761
H	3.563761	2.781198	2.804011

Supporting Information: 035-Selenylated-Cluster-RuII-2HOAc-1OAc-CsBr-Decarboxylation-TS-34-SP-TFE-PBE.log

----

Using Gaussian 16: ES64L-G16RevA.03 25-Dec-2016

==

# pbepbe/Def2QZVP/auto empiricaldispersion=gd3bj
scf=(maxcycle=300,direct,vshift=200,tight,yqc) density=current
scrf=(solvent=2,2,2-Trifluoroethanol,smd)

Pointgroup= C1 Stoichiometry= C16H28B10BrCsO8RuSe C1[X(C16H28B10BrCsO8RuSe)] #Atoms= 66

Charge = 0 Multiplicity = 1

SCF Energy= -6566.44380772

==

Optimization incomplete.

Atomic Type	Coordinates (Angstroms) X	Y	Z
B	3.070737	3.338971	0.508810
B	2.678357	4.258051	-0.957640
H	3.376026	5.131771	-1.389790
H	4.053067	3.514352	1.171070
B	0.912967	4.354160	-1.078370
B	1.801917	3.173261	-2.055670
B	1.544727	2.853121	1.287800
B	2.427238	1.690361	0.305060
H	1.363878	2.655140	2.450370
H	1.717349	-0.541969	2.545600
H	1.739747	3.205181	-3.250070
H	0.239856	5.193340	-1.602310
C	0.701798	1.846400	0.171960
C	0.364708	2.741040	-1.226700
B	1.688887	4.456461	0.517330
B	0.212967	3.498860	0.304820
H	-0.885603	3.746919	0.691110
H	1.648806	5.461561	1.168100
B	3.131338	2.534171	-1.082830
H	4.139648	2.128052	-1.585310
B	1.631268	1.588971	-1.267940
H	1.492429	0.601921	-1.907590
C	-0.890512	1.144749	1.144160
O	-1.792581	0.764589	0.282730
O	-0.942462	1.641869	2.254930
Cs	-3.760230	-1.481332	-0.807050
C	-0.896202	2.447379	-2.025470
H	-0.808033	2.956429	-2.997970
H	-1.001872	1.365889	-2.196400
H	-1.787403	2.826159	-1.504640
Ru	-0.287701	-0.806670	0.472050
O	-0.650281	-1.032641	2.594030
O	0.951350	-2.523840	0.703870
C	0.091489	-0.786620	3.572310
C	-0.080059	-3.279210	0.690320
O	1.381129	-0.534580	3.507010
O	-1.232870	-2.689141	0.596940
C	0.024851	-4.773310	0.737170
H	0.074472	-5.161860	-0.295760
H	0.946362	-5.068880	1.260770
H	-0.856678	-5.208911	1.232550
C	-0.481131	-0.731351	4.959880
H	0.263919	-1.028330	5.712370

H	-0.775161	0.316519	5.150870
H	-1.380530	-1.359901	5.023410
Se	3.323329	0.131601	1.166480
C	4.075289	-0.784718	-0.362480
C	5.466009	-0.690407	-0.538640
C	3.294370	-1.527299	-1.260030
C	6.075310	-1.335977	-1.623870
H	6.062229	-0.100907	0.164180
C	3.911200	-2.159348	-2.349080
H	2.214520	-1.604929	-1.124730
C	5.298430	-2.068868	-2.532760
H	7.158560	-1.255237	-1.762350
H	3.293760	-2.722119	-3.057450
H	5.774260	-2.565277	-3.385080
Br	-0.382180	-1.242030	-2.046130
O	-5.183202	1.179677	-0.447120
C	-4.906082	2.337177	-0.120180
O	-3.708942	2.713298	0.354490
H	-3.096292	1.915208	0.376200
C	-5.867313	3.500367	-0.206980
H	-5.463403	4.269247	-0.887200
H	-6.844073	3.154576	-0.570790
H	-5.977293	3.973627	0.783010

Supporting Information: 035-Selenylated-Cluster-RuII-2HOAc-1OAc-CsBr-Decarboxylation-TS-35-SP-TFE-PBE.log

----

Using Gaussian 16: ES64L-G16RevA.03 25-Dec-2016

==

# pbepbe/Def2QZVP/auto empiricaldispersion=gd3bj
scf=(maxcycle=300,direct,vshift=200,tight,yqc) density=current
scrf=(solvent=2,2,2-Trifluoroethanol,smd)

Pointgroup= C1 Stoichiometry= C16H28B10BrCsO8RuSe C1[X(C16H28B10BrCsO8RuSe)]
#Atoms= 66
Charge = 0 Multiplicity = 1

SCF Energy= -6566.44380926

==

Optimization incomplete.

Atomic Type	Coordinates (Angstroms) X	Y	Z
B	3.070639	3.339101	0.508541

B	2.678219	4.258100	-0.957949
H	3.375859	5.131831	-1.390139
H	4.052979	3.514541	1.170771
B	0.912829	4.354160	-1.078649
B	1.801799	3.173240	-2.055909
B	1.544670	2.853240	1.287571
B	2.427200	1.690470	0.304861
H	1.363840	2.655300	2.450151
H	1.717241	-0.540970	2.545701
H	1.739599	3.205110	-3.250309
H	0.239679	5.193290	-1.602619
C	0.701740	1.846450	0.171791
C	0.364610	2.741010	-1.226909
B	1.688769	4.456550	0.517021
B	0.212869	3.498900	0.304581
H	-0.885701	3.746949	0.690881
H	1.648659	5.461680	1.167751
B	3.131250	2.534231	-1.083079
H	4.139560	2.128111	-1.585539
B	1.631200	1.588980	-1.268109
H	1.492360	0.601910	-1.907739
C	-0.890520	1.144789	1.144041
O	-1.792600	0.764549	0.282651
O	-0.942470	1.641969	2.254781
Cs	-3.760179	-1.481612	-0.806729
C	-0.896310	2.447279	-2.025649
H	-0.808150	2.956269	-2.998179
H	-1.001970	1.365789	-2.196499
H	-1.787500	2.826099	-1.504829
Ru	-0.287629	-0.806651	0.472041
O	-0.650149	-1.032581	2.594041
O	0.951551	-2.523690	0.703891
C	0.091411	-0.785950	3.572331
C	-0.079788	-3.279161	0.690421
O	1.380931	-0.533330	3.507071
O	-1.232649	-2.689201	0.597021
C	0.025242	-4.773240	0.737411
H	0.073002	-5.162050	-0.295509
H	0.947652	-5.068680	1.259501
H	-0.855448	-5.208731	1.234421
C	-0.481299	-0.730631	4.959851
H	0.263361	-1.028790	5.712281
H	-0.774060	0.317509	5.151321
H	-1.381409	-1.358171	5.023021
Se	3.323351	0.131821	1.166421
C	4.075281	-0.784759	-0.362389

C	5.466071	-0.690899	-0.538269
C	3.294321	-1.527119	-1.260089
C	6.075391	-1.336698	-1.623349
H	6.062331	-0.101568	0.164651
C	3.911161	-2.159389	-2.348989
H	2.214411	-1.604410	-1.124999
C	5.298461	-2.069359	-2.532389
H	7.158691	-1.256318	-1.761609
H	3.293691	-2.721989	-3.057469
H	5.774311	-2.565959	-3.384589
Br	-0.382229	-1.242081	-2.046119
O	-5.183220	1.179428	-0.447309
C	-4.906180	2.336968	-0.120439
O	-3.709040	2.713208	0.354171
H	-3.096350	1.915148	0.375941
C	-5.867521	3.500068	-0.207249
H	-5.463571	4.269118	-0.887259
H	-6.844170	3.154217	-0.571289
H	-5.977761	3.973137	0.782811

Supporting Information: 035-Selenylated-Cluster-RuII-2HOAc-1OAc-Decarboxylation-TS-01-SP-TFE-PBE.log

----

Using Gaussian 16: ES64L-G16RevA.03 25-Dec-2016

==

# pbepbe/Def2QZVP/auto empiricaldispersion=gd3bj
scf=(maxcycle=300,direct,vshift=200,tight,yqc) density=current
scrf=(solvent=2,2,2-Trifluoroethanol,smd)

Pointgroup= C1 Stoichiometry= C16H28B10O8RuSe C1[X(C16H28B10O8RuSe)] #Atoms= 64
Charge = 0 Multiplicity = 1

SCF Energy= -3972.34630426

==

Optimization incomplete.

Atomic Type	Coordinates (Angstroms) X	Y	Z
B	-3.468061	1.980688	-0.503771
B	-3.391371	2.812318	1.073399
H	-4.369711	3.114528	1.693789
H	-4.493801	1.657858	-1.031751
B	-1.959711	3.865578	1.072169
B	-1.902191	2.300518	1.919279

B	-2.049231	2.504178	-1.466061
B	-2.026111	0.980058	-0.605661
H	-1.940661	2.547168	-2.653181
H	-1.696091	2.250499	3.096579
H	-1.792942	4.879778	1.682149
C	-0.663461	2.026729	-0.554881
C	-0.618241	2.832839	0.926439
B	-2.898611	3.670108	-0.420761
B	-1.119261	3.675289	-0.487601
H	-0.376012	4.485949	-0.942071
H	-3.505512	4.590318	-0.887671
B	-2.835951	1.132468	0.952679
H	-3.377960	0.199618	1.466689
B	-1.058281	1.164099	0.845059
H	-0.394310	0.215489	1.525349
C	0.786589	1.805129	-1.597831
O	1.670359	2.610970	-1.331441
O	0.528489	0.924869	-2.434631
C	0.730859	3.206489	1.517629
H	0.692848	4.256019	1.847109
H	0.957679	2.569229	2.385029
H	1.513479	3.090460	0.753419
Ru	0.434050	-0.294111	0.119729
O	0.901390	-2.198781	0.881289
C	1.647111	-2.504950	-0.151541
O	1.653900	-1.642750	-1.093781
C	2.468641	-3.748050	-0.172861
H	2.125721	-4.463320	0.588659
H	2.437511	-4.204510	-1.174871
H	3.512071	-3.445210	0.034449
Se	-1.523670	-0.842411	-1.338811
C	-2.816520	-1.877922	-0.314241
C	-4.149850	-1.885862	-0.751121
C	-2.417709	-2.629232	0.799279
C	-5.099969	-2.635683	-0.044441
H	-4.449360	-1.293042	-1.621341
C	-3.375339	-3.382702	1.494399
H	-1.373489	-2.612471	1.124169
C	-4.714359	-3.383972	1.077709
H	-6.144539	-2.631233	-0.371861
H	-3.071409	-3.965762	2.370029
H	-5.459089	-3.967573	1.628069
O	2.206230	0.496170	1.046179
C	2.839090	0.060420	2.034099
O	2.505080	-1.032000	2.711459
C	4.070539	0.752190	2.534229

H	4.936430	0.207411	2.118229
H	4.091009	1.791100	2.177379
H	4.125049	0.710160	3.632809
H	1.786490	-1.511900	2.199769
H	3.130880	-0.932520	-1.766051
O	3.952480	-0.402800	-1.971991
C	4.780770	-0.497879	-0.921441
O	4.586970	-1.233700	0.051499
C	5.965970	0.430941	-1.059171
H	6.351170	0.425901	-2.090871
H	5.635409	1.462141	-0.841201
H	6.755730	0.143531	-0.351231

Supporting Information: 035-Selenylated-Cluster-RuII-2HOAc-1OAc-Decarboxylation-TS-02-SP-TFE-PBE.log

----

Using Gaussian 16: ES64L-G16RevA.03 25-Dec-2016

==

# pbepbe/Def2QZVP/auto empiricaldispersion=gd3bj
scf=(maxcycle=300,direct,vshift=200,tight,yqc) density=current
scrf=(solvent=2,2,2-Trifluoroethanol,smd)

Pointgroup= C1 Stoichiometry= C16H28B10O8RuSe C1[X(C16H28B10O8RuSe)] #Atoms= 64
Charge = 0 Multiplicity = 1

SCF Energy= -3972.34348519

==

Optimization incomplete.

Atomic Type	Coordinates (Angstroms) X	Y	Z
B	-1.455722	-3.524409	-0.124591
B	-2.380022	-3.457229	1.402149
H	-2.421482	-4.371769	2.173689
H	-0.814752	-4.476370	-0.466071
B	-3.799661	-2.422718	1.144589
B	-2.378761	-1.770859	1.996259
B	-2.302571	-2.493349	-1.317521
B	-0.905911	-1.881660	-0.449181
H	-2.298811	-2.585069	-2.506451
H	-2.467711	-1.380759	3.123839
H	-4.856441	-2.472108	1.700739
C	-2.313251	-0.906029	-0.641941
C	-3.192751	-0.872778	0.795119

B	-3.239922	-3.479518	-0.167621
B	-3.758591	-1.816938	-0.528491
H	-4.715721	-1.425568	-1.117041
H	-3.911072	-4.396348	-0.544591
B	-0.931501	-2.454540	1.219649
H	0.071229	-2.623610	1.844489
B	-1.464150	-0.802039	0.817679
H	-0.801020	0.208260	1.379819
C	-2.453280	0.334691	-1.893161
O	-3.491400	0.984092	-1.802201
O	-1.488400	0.214191	-2.669151
C	-3.981650	0.377982	1.144479
H	-4.985680	0.078152	1.481399
H	-3.484150	0.930302	1.955279
H	-4.068170	1.018292	0.253869
Ru	-0.458720	0.955240	-0.140001
O	1.215321	2.124419	0.475549
C	1.264001	2.718929	-0.715221
O	0.392721	2.338800	-1.545121
C	2.298752	3.752579	-1.011781
H	3.154401	3.237468	-1.482561
H	1.895492	4.491869	-1.720201
H	2.643432	4.245309	-0.089601
Se	0.728129	-0.954230	-1.228051
C	2.036439	-1.725081	-0.006791
C	2.800909	-2.798991	-0.486831
C	2.236419	-1.229111	1.287749
C	3.757248	-3.394552	0.348479
H	2.644698	-3.174271	-1.503231
C	3.199969	-1.822092	2.115379
H	1.632410	-0.390111	1.645309
C	3.956679	-2.908122	1.649779
H	4.346268	-4.240342	-0.020411
H	3.358289	-1.431442	3.125429
H	4.702918	-3.375002	2.300589
O	-1.802769	2.518251	0.493649
C	-1.610878	3.412131	1.345409
O	-0.456478	3.606250	1.977399
C	-2.705408	4.360481	1.741639
H	-2.415428	5.393131	1.483369
H	-3.635578	4.094152	1.223009
H	-2.851958	4.326241	2.834399
H	0.233041	2.992740	1.570549
H	2.932571	1.664699	0.906139
O	3.876011	1.378358	1.060789
C	4.308010	0.855408	-0.113941

O	3.653890	0.906108	-1.153181
C	5.651680	0.183837	0.025079
H	6.289960	0.708307	0.752669
H	5.483870	-0.842223	0.398559
H	6.142220	0.126177	-0.956461

Supporting Information: 041-Decarboxylated-Cluster-RuII-2HOAc-2OAc-CsBr-H-Migration-TS-01-SP-TFE-PBE.log

----

Using Gaussian 16: ES64L-G16RevA.03 25-Dec-2016

==

# pbepbe/Def2QZVP/auto empiricaldispersion=gd3bj
scf=(maxcycle=300,direct,vshift=200,tight,yqc) density=current
scrf=(solvent=2,2,2-Trifluoroethanol,smd)

Pointgroup= C1 Stoichiometry= C15H28B10BrCsO6RuSe C1[X(C15H28B10BrCsO6RuSe)]
#Atoms= 63
Charge = 0 Multiplicity = 1

SCF Energy= -6378.02366458

==

Optimization incomplete.

Atomic Type	Coordinates (Angstroms) X	Y	Z
B	-2.687610	3.324369	-1.216450
B	-3.068810	4.244589	0.260990
H	-3.794120	5.198779	0.272930
H	-3.120460	3.589439	-2.302410
B	-1.651430	4.169299	1.330830
B	-3.040600	3.113159	1.635480
B	-1.028950	2.700079	-1.029000
B	-2.394510	1.651319	-0.707560
H	-0.284480	2.459019	-1.932900
H	-3.642300	3.182249	2.667580
H	-1.309570	4.960739	2.160950
C	-1.048150	1.611779	0.313790
C	-1.430940	2.508479	1.702570
B	-1.416160	4.307509	-0.418990
B	-0.393960	3.195229	0.537970
H	0.760970	3.313939	0.816520
H	-0.937440	5.289619	-0.911580
B	-3.687690	2.576729	0.064500
H	-4.847120	2.305619	-0.066750

B	-2.633880	1.510579	1.022910
H	-2.944970	0.509459	1.582770
C	-0.799680	2.088259	3.019670
H	-1.436060	1.356269	3.538350
H	0.187130	1.637169	2.833730
H	-0.669750	2.972429	3.662660
Ru	-0.242430	-0.320371	0.024640
Se	-2.181440	-0.160551	-1.608830
C	-3.660810	-1.106611	-0.775730
C	-4.964810	-0.628741	-0.981720
C	-3.425720	-2.273931	-0.037270
C	-6.046030	-1.319461	-0.418320
H	-5.135150	0.282139	-1.563060
C	-4.517930	-2.957381	0.517470
H	-2.403470	-2.638171	0.104190
C	-5.824460	-2.483271	0.333070
H	-7.063130	-0.942401	-0.567060
H	-4.338970	-3.867171	1.100230
H	-6.671230	-3.019801	0.773550
O	1.110330	-0.642581	1.780020
C	0.078580	-1.046871	2.441880
C	0.211730	-1.636601	3.819600
H	0.346610	-2.729901	3.734700
H	1.084610	-1.213561	4.341160
H	-0.704610	-1.453561	4.401520
O	-1.065030	-0.950631	1.877690
Cs	3.482450	-1.746081	0.189690
O	0.834390	0.056169	-1.821430
C	1.775070	0.891299	-1.704060
O	2.025810	1.428309	-0.535640
H	1.007450	0.969849	0.076150
C	2.633300	1.283929	-2.871640
H	2.487850	2.357829	-3.082030
H	3.698760	1.133939	-2.625370
H	2.358890	0.697279	-3.759180
Br	0.210050	-2.865751	-0.513900
O	5.184080	0.750449	-0.386780
C	5.322870	1.957689	-0.154660
O	4.306550	2.825419	-0.083470
H	3.443520	2.324109	-0.238810
C	6.658890	2.626839	0.077660
H	6.674450	3.100549	1.073740
H	7.469340	1.889779	-0.000480
H	6.810010	3.431219	-0.661710

Supporting Information: 045-Decarboxylated-Cluster-RuII-2HOAc-1OAc-CsBr-01-Protonolysis-TS-01-SP-TFE-PBE.log

----
Using Gaussian 16: ES64L-G16RevA.03 25-Dec-2016
==
# pbepbe/Def2QZVP/auto empiricaldispersion=gd3bj
scf=(maxcycle=300,direct,vshift=200,tight,yqc) density=current
scrf=(solvent=2,2,2-Trifluoroethanol,smd)

Pointgroup= C1 Stoichiometry= C15H28B10BrCsO6RuSe C1[X(C15H28B10BrCsO6RuSe)]
#Atoms= 63
Charge = 0 Multiplicity = 1

SCF Energy= -6378.01944980
==
Optimization incomplete.

Atomic Type	Coordinates (Angstroms) X	Y	Z
B	3.351459	-1.737311	-2.332930
B	4.340988	-2.843401	-1.344530
H	5.374338	-3.316352	-1.723260
H	3.639859	-1.392191	-3.443250
B	3.242668	-3.776361	-0.302600
B	4.126879	-2.393511	0.364140
B	1.649929	-2.014920	-1.891890
B	2.521270	-0.644900	-1.212170
H	0.700489	-1.829929	-2.586830
H	4.887529	-2.542102	1.275140
H	3.407417	-4.867221	0.159210
C	1.516519	-1.581870	-0.219940
C	2.455869	-2.624760	0.697000
B	2.756668	-3.388421	-1.965130
B	1.578398	-3.264180	-0.638380
H	0.617748	-3.928239	-0.400730
H	2.625018	-4.240580	-2.795770
B	4.195629	-1.120141	-0.882780
H	5.098230	-0.334462	-0.918030
B	3.022310	-1.022931	0.442700
H	3.003160	-0.256961	1.348920
C	1.952448	-2.984340	2.085400
H	2.396509	-2.318600	2.840200
H	0.855998	-2.887029	2.127850
H	2.224858	-4.025530	2.315800
Ru	0.046740	-0.065289	0.380220

Se	1.503871	1.102020	-1.338230
C	2.747641	2.231879	-0.356770
C	4.003072	2.494449	-0.927490
C	2.370972	2.812790	0.861140
C	4.901772	3.333328	-0.254710
H	4.282681	2.038849	-1.882450
C	3.277992	3.654779	1.521720
H	1.386892	2.603100	1.288080
C	4.540862	3.913638	0.970420
H	5.886072	3.530948	-0.691460
H	2.990243	4.107679	2.476270
H	5.244913	4.568428	1.494080
O	-0.929400	-0.888368	2.220740
C	-0.007320	-0.336429	2.934710
C	-0.072260	-0.369049	4.439780
H	-0.660760	0.494891	4.797470
H	-0.563671	-1.291309	4.787770
H	0.939220	-0.291440	4.866610
O	0.951530	0.253700	2.330810
Cs	-3.734080	-0.767447	1.084890
O	-1.089460	-0.315908	-1.377660
C	-1.700261	-1.452128	-1.581310
O	-1.789391	-2.382158	-0.745740
H	0.158519	-1.682599	0.111690
C	-2.361641	-1.562738	-2.951380
H	-2.190491	-2.571968	-3.358130
H	-3.451301	-1.416577	-2.845470
H	-1.970110	-0.803138	-3.644640
Br	-1.319899	2.056082	0.808700
O	-4.321059	0.820494	-1.446390
C	-3.818319	1.528913	-2.324510
O	-2.665279	2.193513	-2.197690
H	-2.258729	1.991382	-1.300330
C	-4.428359	1.716484	-3.697290
H	-3.940369	1.017633	-4.400130
H	-5.502789	1.487344	-3.664150
H	-4.259218	2.737244	-4.072910

Supporting Information: 045-Decarboxylated-Cluster-RuII-2HOAc-1OAc-CsBr-01-Protonolysis-TS-02-SP-TFE-PBE.log

```
------------------------------------------------------------------------
Using  Gaussian 16:  ES64L-G16RevA.03 25-Dec-2016
========================================================================
# pbepbe/Def2QZVP/auto empiricaldispersion=gd3bj
scf=(maxcycle=300,direct,vshift=200,tight,yqc) density=current
scrf=(solvent=2,2,2-Trifluoroethanol,smd)
------------------------------------------------------------------------
```

Pointgroup= C1 Stoichiometry= C15H28B10BrCsO6RuSe C1[X(C15H28B10BrCsO6RuSe)]
#Atoms= 63
Charge = 0 Multiplicity = 1

SCF Energy= -6378.02395649

===

Optimization incomplete.

Atomic Type	Coordinates (Angstroms) X	Y	Z
B	-2.575010	3.389650	-1.101430
B	-3.040801	4.238520	0.396020
H	-3.782041	5.179269	0.414550
H	-2.959040	3.697900	-2.193520
B	-1.674721	4.136040	1.530150
B	-3.059770	3.047460	1.719480
B	-0.917480	2.783010	-0.868650
B	-2.287220	1.696980	-0.665920
H	-0.117720	2.574461	-1.727760
H	-3.708180	3.060559	2.724360
H	-1.385281	4.890370	2.411890
C	-1.002570	1.653180	0.437760
C	-1.446480	2.462520	1.837440
B	-1.361501	4.357120	-0.202940
B	-0.362640	3.235780	0.749270
H	0.781100	3.317721	1.074780
H	-0.876921	5.365110	-0.630310
B	-3.624140	2.573539	0.094740
H	-4.769150	2.286669	-0.104780
B	-2.615770	1.476830	1.056670
H	-2.926669	0.447760	1.559680
C	-0.863760	1.965950	3.150540
H	-1.544950	1.245210	3.626160
H	0.105610	1.473621	2.972740
H	-0.707890	2.819190	3.828040
Ru	-0.261769	-0.379960	0.127540
Se	-2.057629	-0.062540	-1.644840
C	-3.659939	-0.958561	-0.997200
C	-4.906919	-0.457821	-1.403800
C	-3.565329	-2.102311	-0.194130
C	-6.076969	-1.101451	-0.978760
H	-4.966129	0.433609	-2.035910
C	-4.744698	-2.739021	0.220580

H	-2.585689	-2.485290	0.104290
C	-5.997369	-2.241611	-0.165380
H	-7.051379	-0.707142	-1.284800
H	-4.676938	-3.630901	0.852280
H	-6.912998	-2.742162	0.165930
O	0.960701	-0.896949	1.915470
C	-0.113079	-1.302609	2.505660
C	-0.051769	-1.980489	3.848140
H	0.070642	-3.068149	3.698130
H	0.805341	-1.610049	4.431980
H	-0.989729	-1.817430	4.400590
O	-1.229169	-1.129140	1.905670
Cs	3.413931	-1.666138	0.244220
O	0.938711	0.138991	-1.541770
C	1.930950	0.951491	-1.416310
O	2.343620	1.402551	-0.301430
H	0.316670	1.074311	0.579980
C	2.635300	1.336141	-2.706590
H	2.593090	2.432251	-2.826490
H	3.700820	1.053202	-2.646150
H	2.166210	0.852071	-3.574240
Br	0.058192	-2.822919	-0.561400
O	5.423691	0.507562	-0.521420
C	5.609580	1.729782	-0.426460
O	4.637610	2.632682	-0.319180
H	3.717160	2.151152	-0.327230
C	6.986810	2.362743	-0.429230
H	7.129960	2.962683	0.485160
H	7.761050	1.586093	-0.495120
H	7.078750	3.054463	-1.283770

Supporting Information: 045-Decarboxylated-Cluster-RuII-2HOAc-1OAc-CsBr-01-Protonolysis-TS-03-SP-TFE-PBE.log

----

Using Gaussian 16: ES64L-G16RevA.03 25-Dec-2016

==

# pbepbe/Def2QZVP/auto empiricaldispersion=gd3bj
scf=(maxcycle=300,direct,vshift=200,tight,yqc) density=current
scrf=(solvent=2,2,2-Trifluoroethanol,smd)

Pointgroup= C1 Stoichiometry= C15H28B10BrCsO6RuSe C1[X(C15H28B10BrCsO6RuSe)] #Atoms= 63
Charge = 0 Multiplicity = 1

SCF Energy= -6378.01934560

==

Optimization incomplete.

Atomic Type	Coordinates (Angstroms) X	Y	Z
B	-2.991832	2.714500	-1.663061
B	-3.388082	3.867410	-0.363751
H	-4.257502	4.686799	-0.452971
H	-3.555612	2.671640	-2.719491
B	-1.886682	4.228850	0.520409
B	-3.051562	3.075290	1.193169
B	-1.244172	2.390340	-1.566331
B	-2.388152	1.247050	-0.880761
H	-0.546902	2.056320	-2.473401
H	-3.557972	3.278760	2.257489
H	-1.609863	5.220230	1.129489
C	-0.972422	1.642510	-0.027221
C	-1.362902	2.738680	1.186649
B	-1.838352	4.019700	-1.240801
B	-0.560122	3.317130	-0.222471
H	0.583338	3.628370	-0.114861
H	-1.577643	4.929380	-1.974661
B	-3.737032	2.124799	-0.148571
H	-4.836862	1.656099	-0.083441
B	-2.451782	1.451410	0.872259
H	-2.532052	0.562450	1.655469
C	-0.566762	2.697790	2.480109
H	-0.812692	1.790320	3.050509
H	0.513428	2.723600	2.270529
H	-0.826212	3.579590	3.085859
Ru	-0.021432	-0.339120	0.040899
Se	-2.045272	-0.672200	-1.420871
C	-3.436752	-1.506870	-0.342661
C	-4.769642	-1.325951	-0.743841
C	-3.115262	-2.295490	0.768909
C	-5.796832	-1.929551	-0.005711
H	-5.005912	-0.708201	-1.616091
C	-4.152991	-2.896841	1.496919
H	-2.070951	-2.434350	1.059719
C	-5.489791	-2.714771	1.115439
H	-6.838352	-1.781921	-0.309111
H	-3.907981	-3.511511	2.369439
H	-6.293991	-3.184821	1.690779
O	1.549248	-0.264720	1.636489
C	0.647368	-0.557930	2.529269

C	1.042178	-0.756400	3.967279
H	1.572668	-1.719220	4.072389
H	1.730048	0.043140	4.289909
H	0.150538	-0.771390	4.610519
O	-0.557152	-0.670270	2.140729
Cs	3.667028	-1.447919	-0.442591
O	0.891068	-0.189760	-1.855921
C	1.672768	0.835670	-2.072321
O	2.005338	1.685410	-1.214181
H	0.429268	1.252660	-0.059121
C	2.166038	0.946780	-3.510271
H	1.528788	1.680440	-4.035751
H	3.198438	1.332420	-3.534261
H	2.091378	-0.012440	-4.045561
Br	0.429209	-2.863510	-0.047121
O	4.530178	1.208231	0.747799
C	3.864548	2.203751	1.040749
O	2.733478	2.146390	1.768789
H	2.432818	1.185340	1.807269
C	4.187468	3.607691	0.597799
H	3.875878	4.352101	1.346249
H	5.259318	3.702091	0.373569
H	3.607488	3.787570	-0.325501

Supporting Information: 047-Decarboxylated-Cluster-RuII-2HOAc-1OAc-CsBr-01-Protonolysis-TS-01-SP-TFE-PBE.log

---

Using Gaussian 16: ES64L-G16RevA.03 25-Dec-2016

===

# pbepbe/Def2QZVP/auto empiricaldispersion=gd3bj
scf=(maxcycle=300,direct,vshift=200,tight,yqc) density=current
scrf=(solvent=2,2,2-Trifluoroethanol,smd)

Pointgroup= C1 Stoichiometry= C15H28B10BrCsO6RuSe C1[X(C15H28B10BrCsO6RuSe)] #Atoms= 63

Charge = 0 Multiplicity = 1

SCF Energy= -6377.99696774

===

Optimization incomplete.

Atomic Type	Coordinates (Angstroms) X	Y	Z
B	-3.204152	2.805999	0.853020

B	-2.704072	2.901829	2.563710
H	-3.435012	3.284809	3.431410
H	-4.299832	3.098618	0.466590
B	-0.964842	3.280340	2.608960
B	-1.512231	1.607900	2.886260
B	-1.752522	3.095269	-0.154510
B	-2.310301	1.462659	0.149540
H	-1.694132	3.524239	-1.266590
H	-1.283981	1.057770	3.923360
H	-0.362752	3.859110	3.463790
C	-0.640371	1.840280	0.274150
C	-0.184871	1.962910	1.870120
B	-1.991542	4.013959	1.354400
B	-0.379552	3.405790	0.936420
H	0.608998	3.993091	0.634410
H	-2.193063	5.193829	1.339110
B	-2.900961	1.307929	1.806240
H	-3.752900	0.519958	2.093740
B	-1.283710	0.714630	1.372540
H	-1.012440	-0.552600	1.519130
C	1.238239	1.561791	2.218630
H	1.312460	0.473421	2.365540
H	1.926549	1.887841	1.416210
H	1.525689	2.062631	3.156140
Ru	-0.467210	-0.663970	-0.156700
Se	-2.572150	0.040289	-1.261130
C	-4.048510	-0.896582	-0.404770
C	-5.318880	-0.302823	-0.435560
C	-3.843789	-2.151282	0.182070
C	-6.398740	-0.969883	0.158470
H	-5.459880	0.680407	-0.895670
C	-4.935409	-2.811662	0.766560
H	-2.846129	-2.598151	0.190960
C	-6.208099	-2.223193	0.759680
H	-7.389890	-0.505214	0.153140
H	-4.784848	-3.791172	1.232240
H	-7.053899	-2.741123	1.222940
O	1.326141	-1.745789	0.751660
C	0.632201	-2.831569	0.640360
C	1.179892	-4.170119	1.043260
H	1.847452	-4.541479	0.245420
H	1.771472	-4.078219	1.968490
H	0.363363	-4.893649	1.180650
O	-0.549839	-2.724010	0.158080
Cs	3.490000	-0.106738	-1.091250
Br	0.383181	-1.266339	-2.467530

H	0.265659	1.724701	-0.574660
O	1.118569	2.223681	-1.640310
C	2.125418	2.971292	-1.308200
C	2.362408	4.177722	-2.218600
H	2.173778	3.917422	-3.272870
H	1.638557	4.967391	-1.946610
H	3.379247	4.579572	-2.091900
O	2.888398	2.773362	-0.322710
O	4.593751	-1.235257	1.583370
C	4.155261	-1.459847	2.711770
O	2.858851	-1.730168	2.966000
C	4.989671	-1.451617	3.971920
H	4.822781	-2.374507	4.551170
H	6.052701	-1.348156	3.716440
H	4.681120	-0.607387	4.612560
H	2.353861	-1.704418	2.089650

18. ^{1}H, ^{13}C, ^{11}B and ^{19}F NMR data

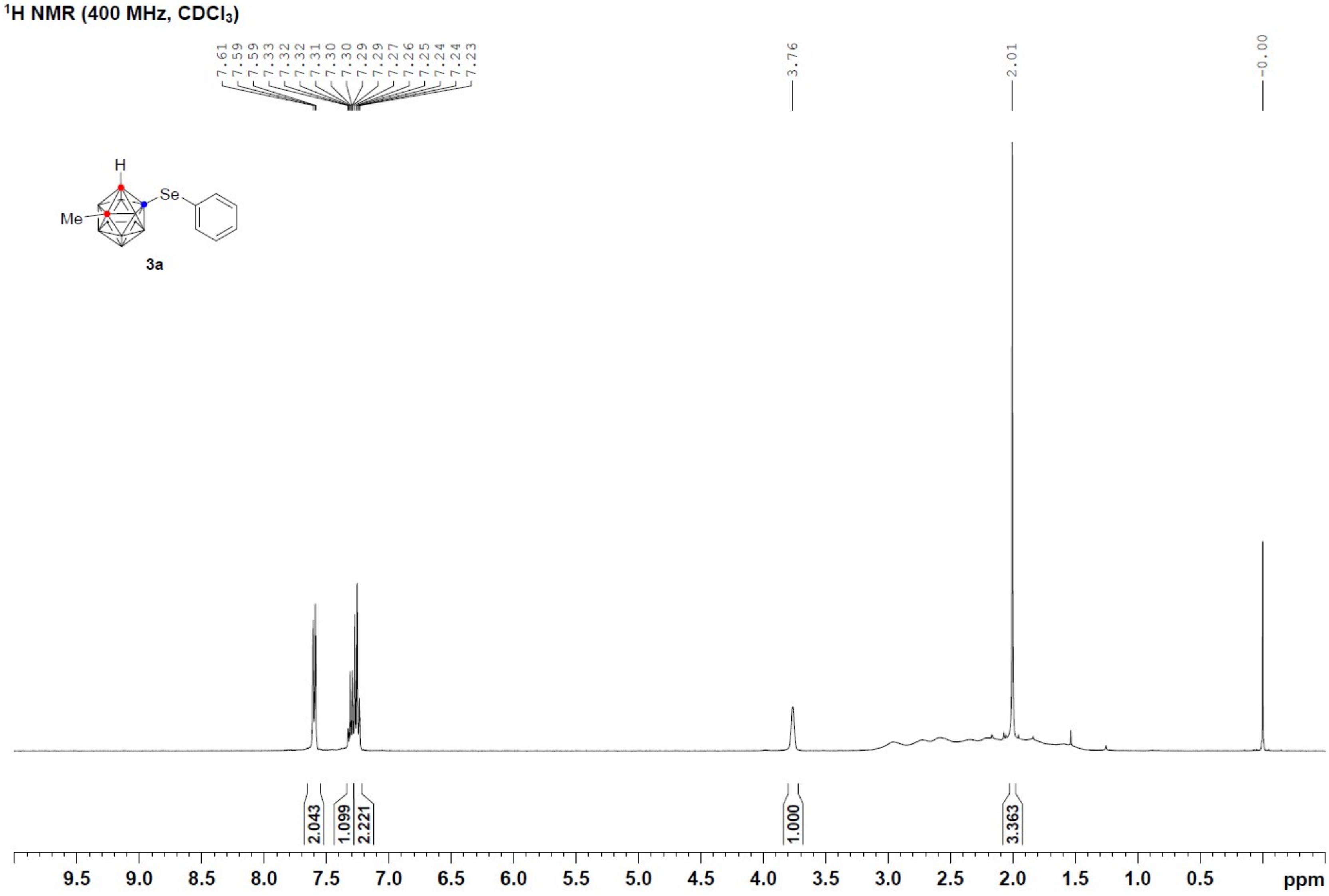

$^{13}C\{^1H\}$ NMR (100 MHz, $CDCl_3$)

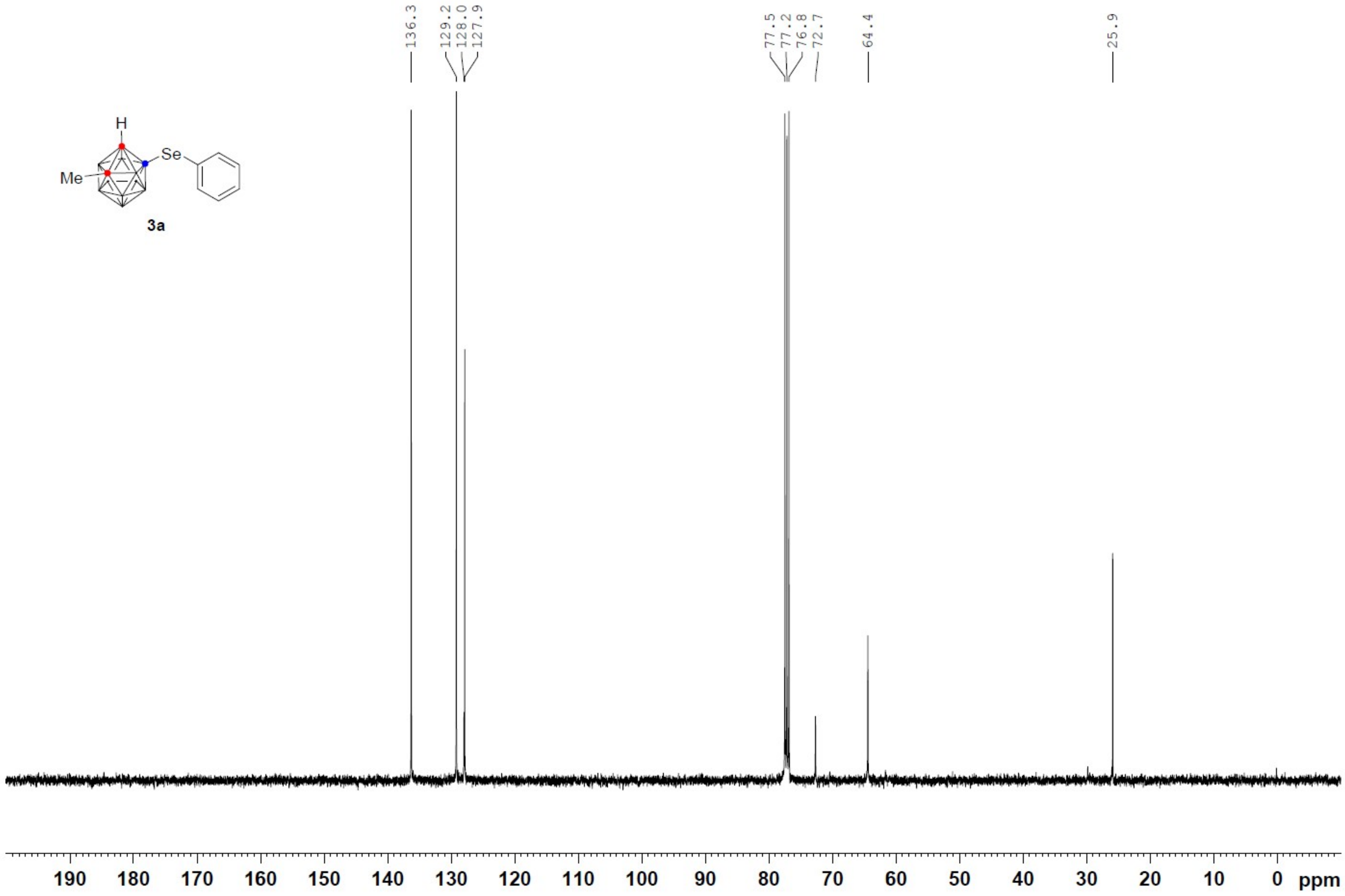

^{11}B NMR (128 MHz, $CDCl_3$)

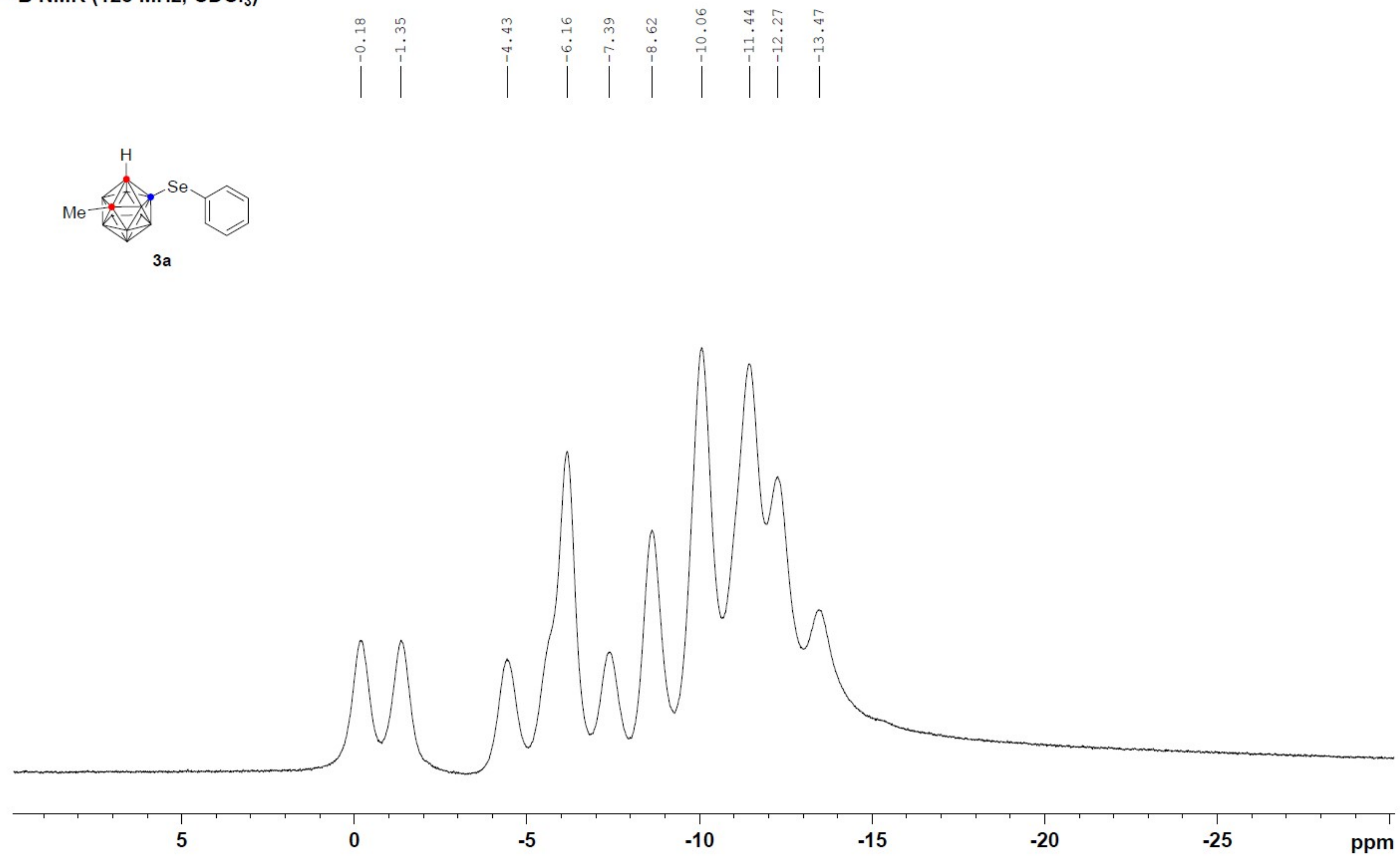

$^{11}B\{^1H\}$ NMR (128 MHz, $CDCl_3$)

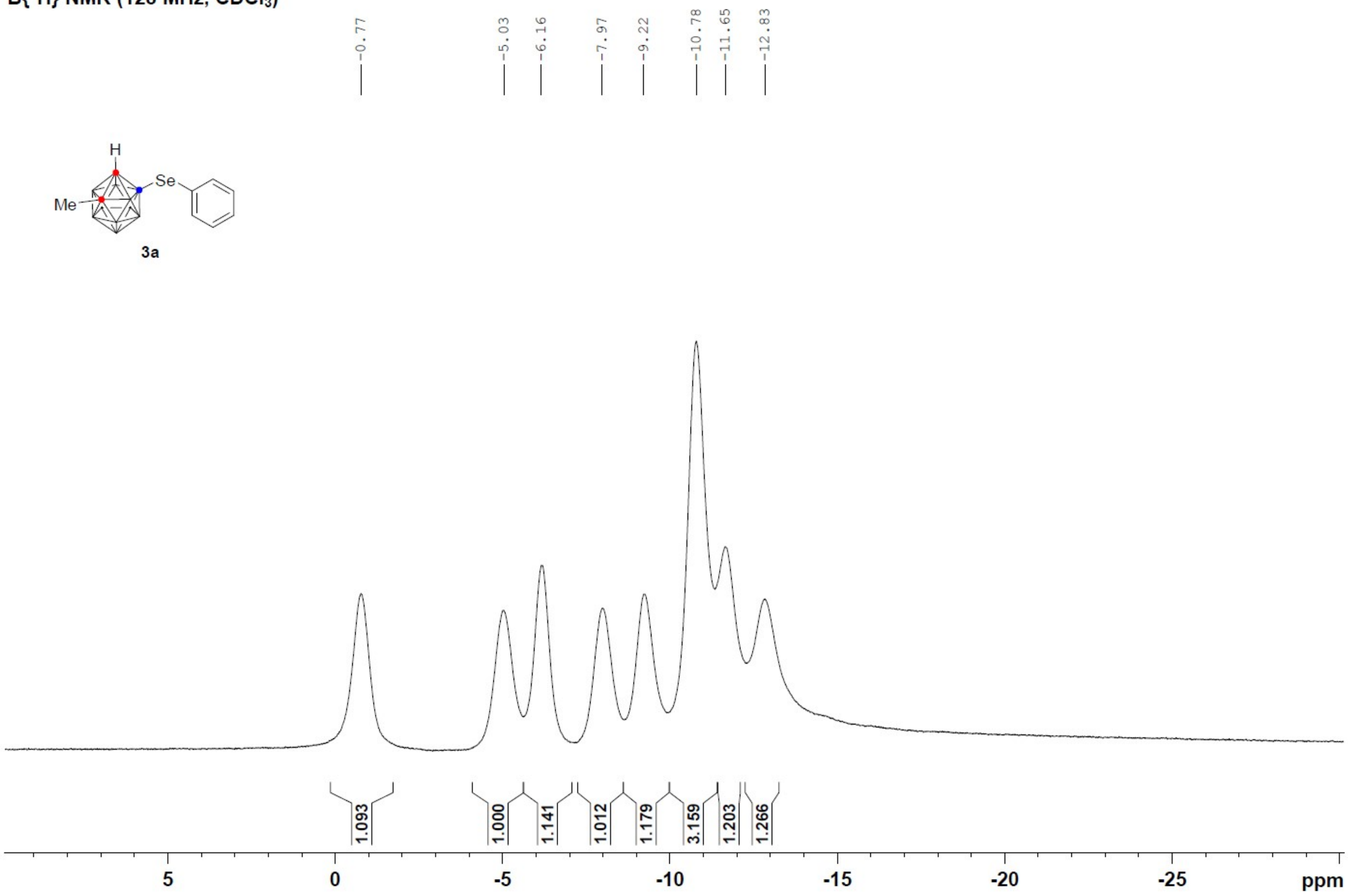

^1H NMR (400 MHz, $CDCl_3$)

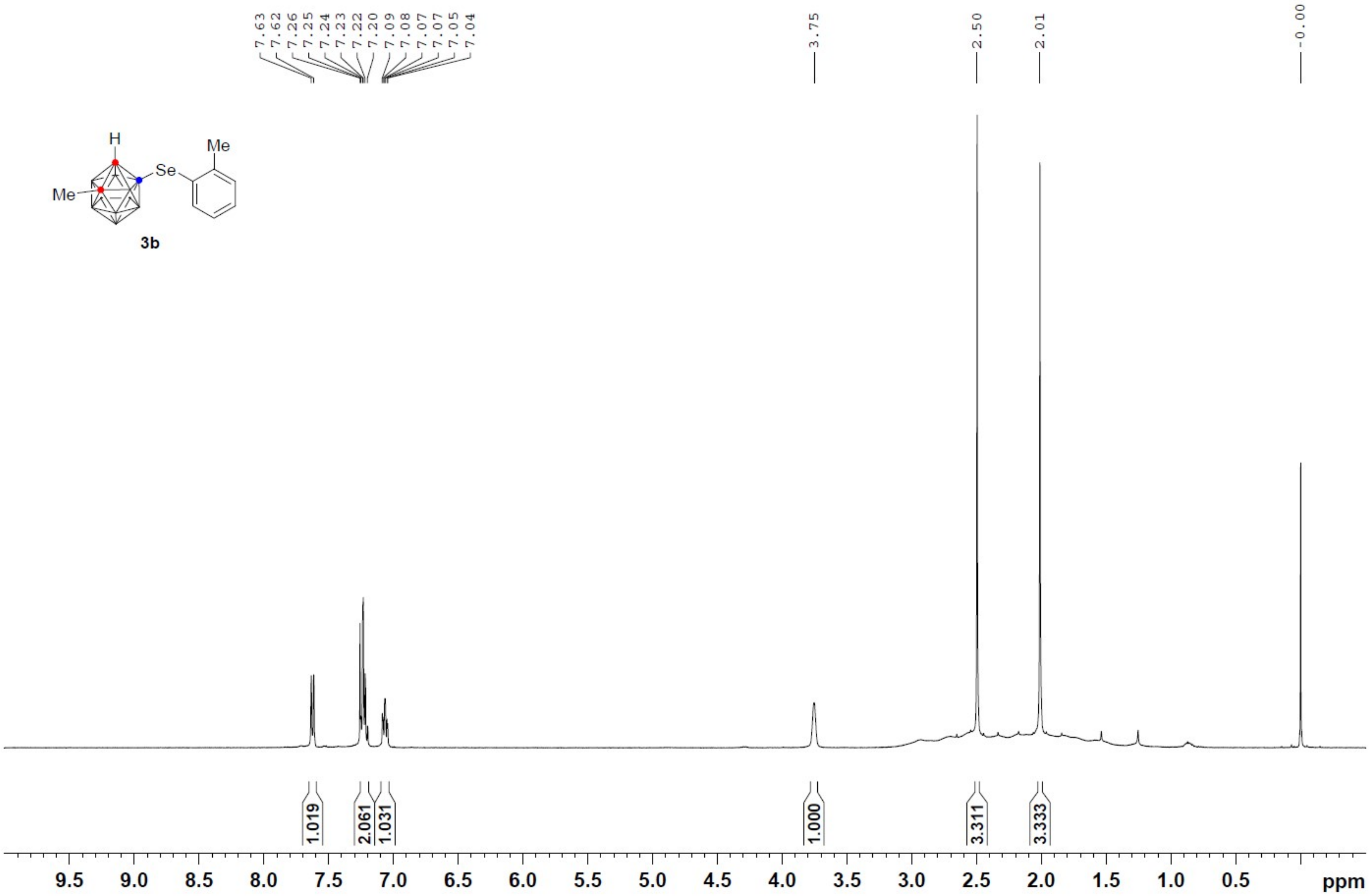

$^{13}C\{^1H\}$ NMR (100 MHz, CDCl3)

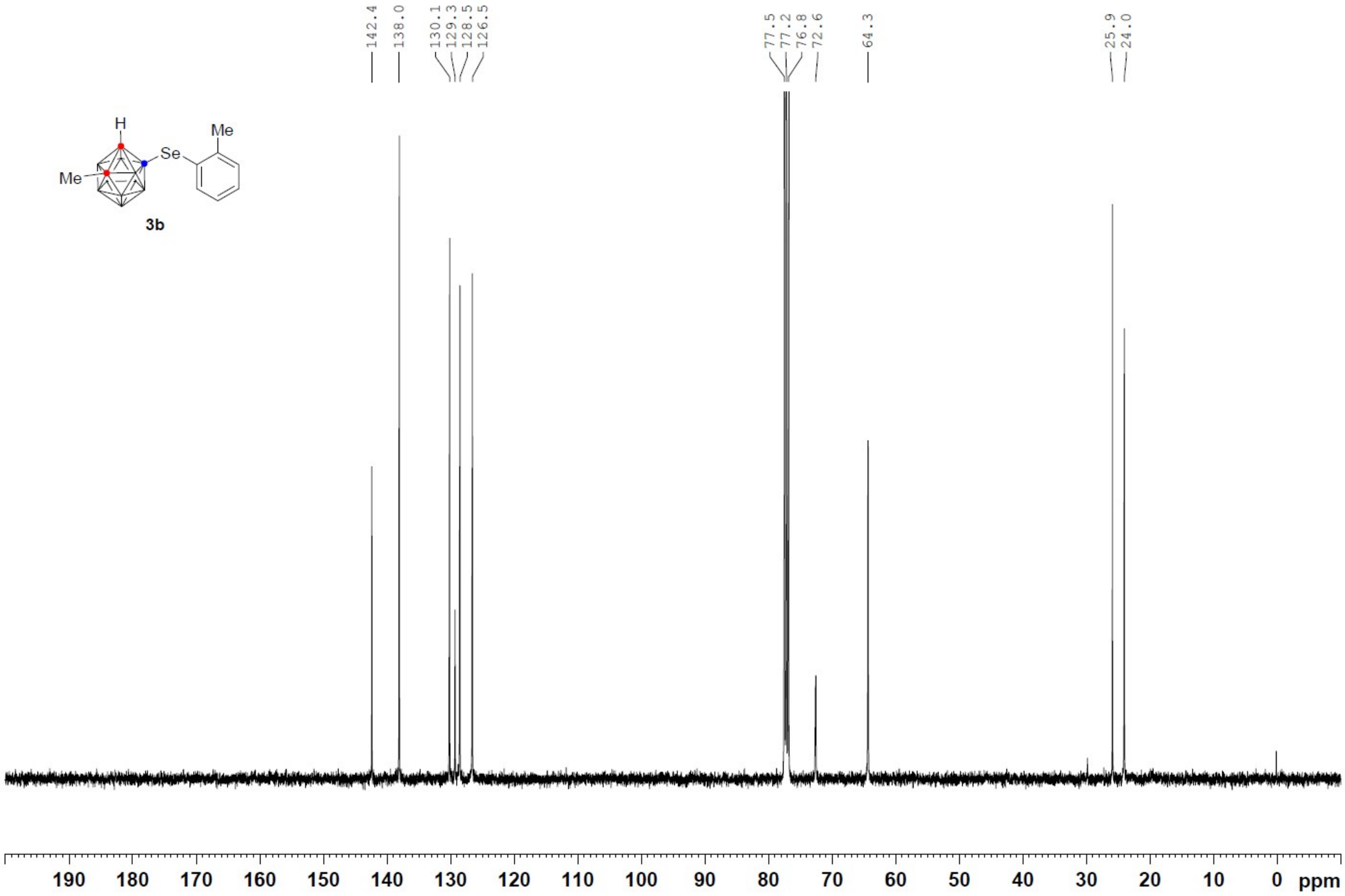

3b

^{11}B NMR (128 MHz, $CDCl_3$)

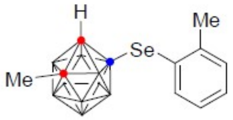

3b

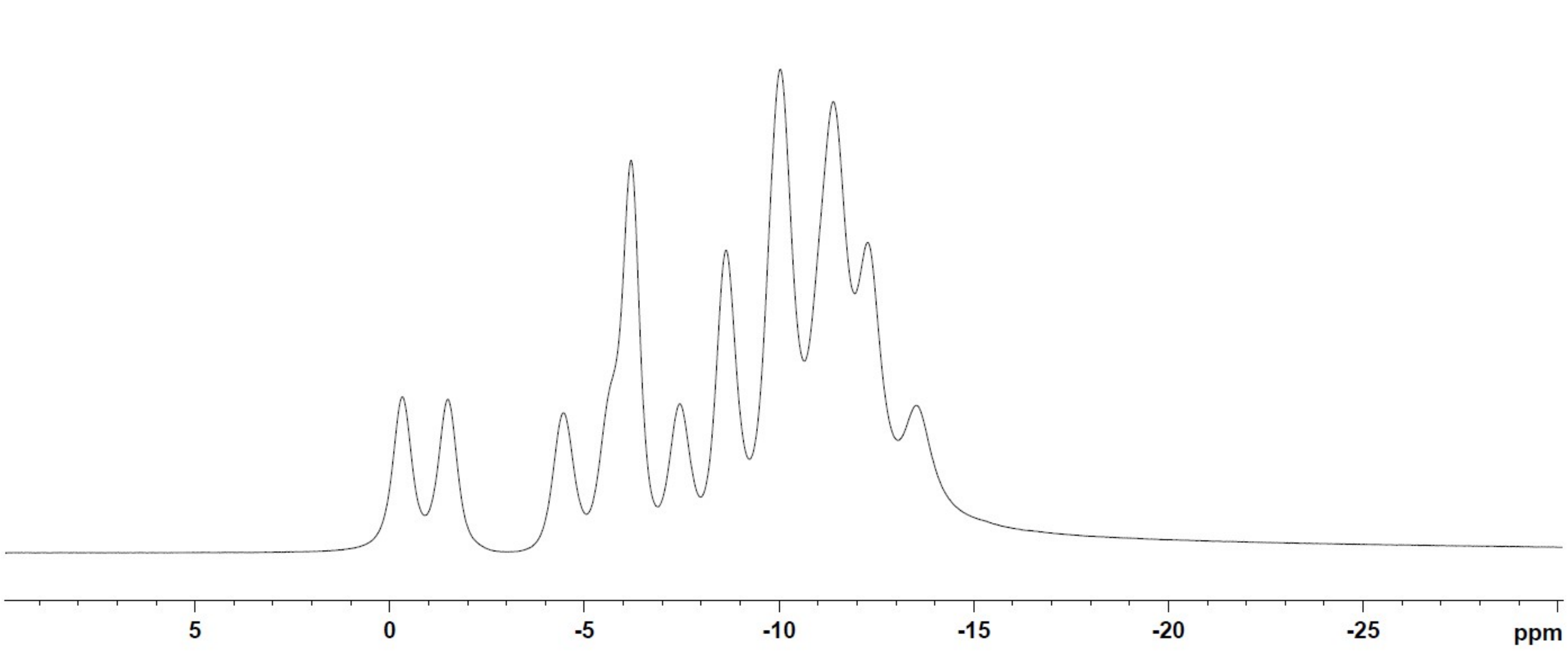

$^{11}B\{^1H\}$ NMR (128 MHz, $CDCl_3$)

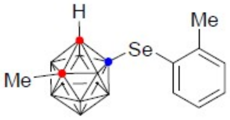

3b

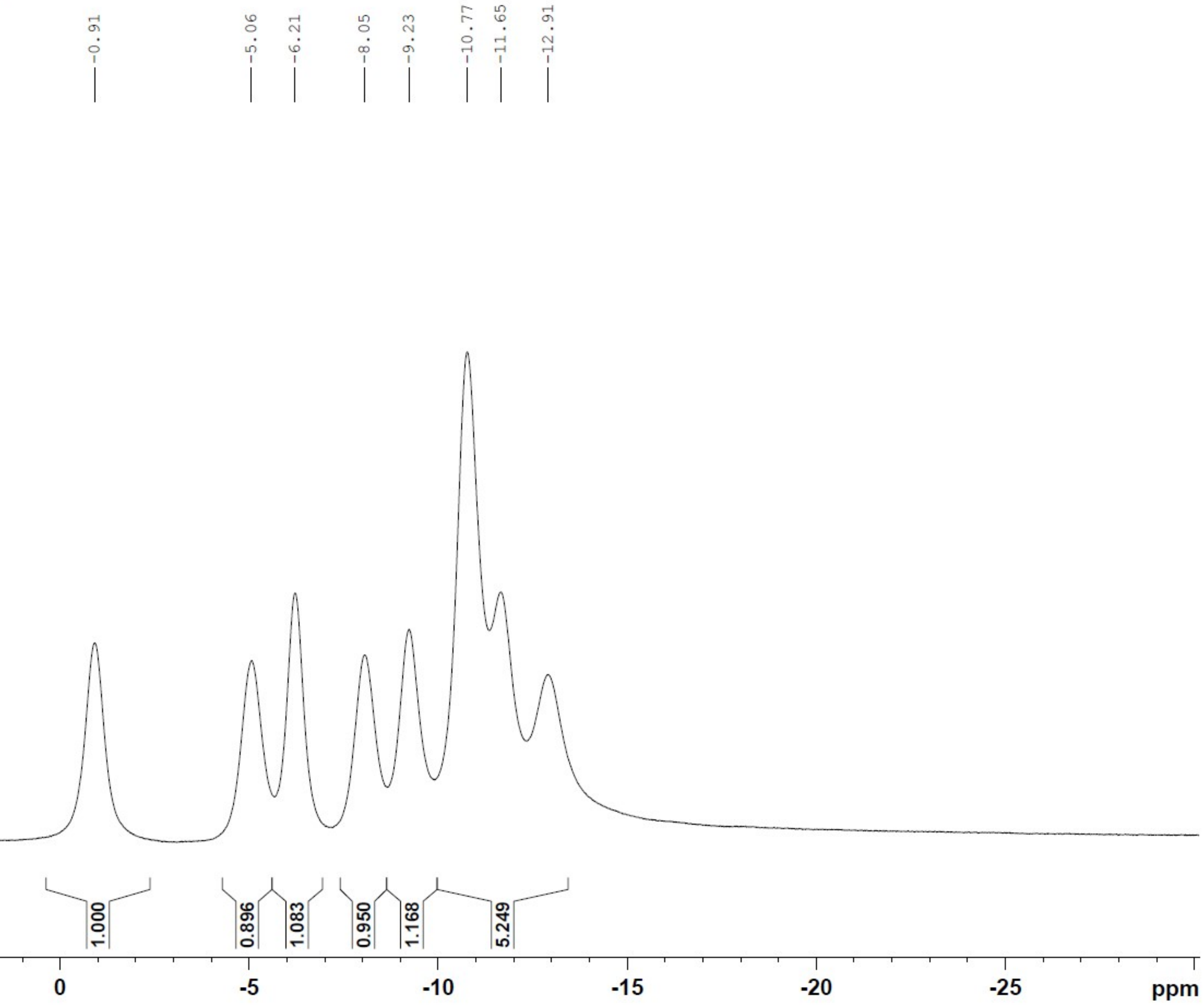

^{1}H NMR (400 MHz, $CDCl_3$)

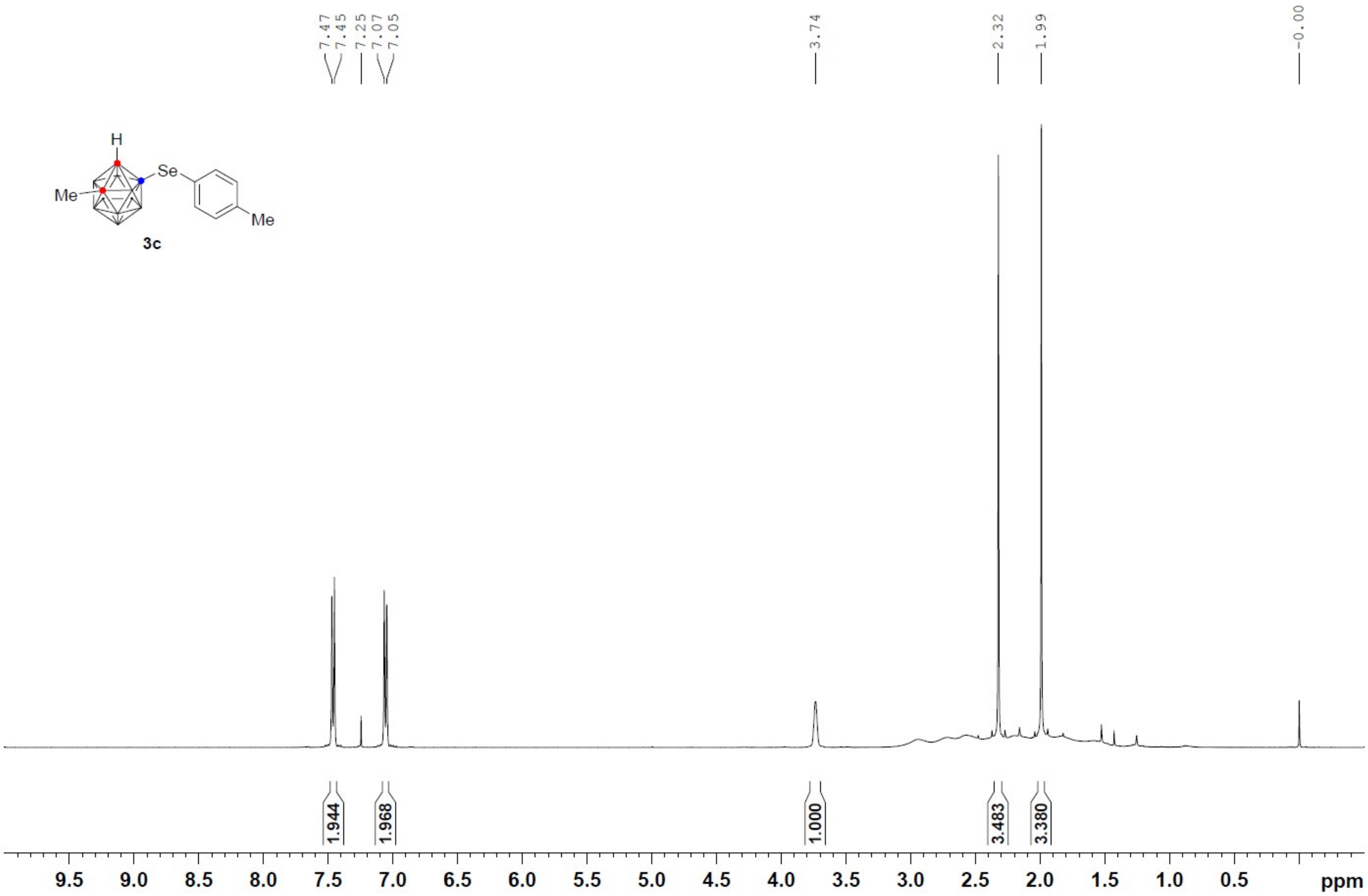

^{13}C NMR (100 MHz, $CDCl_3$)

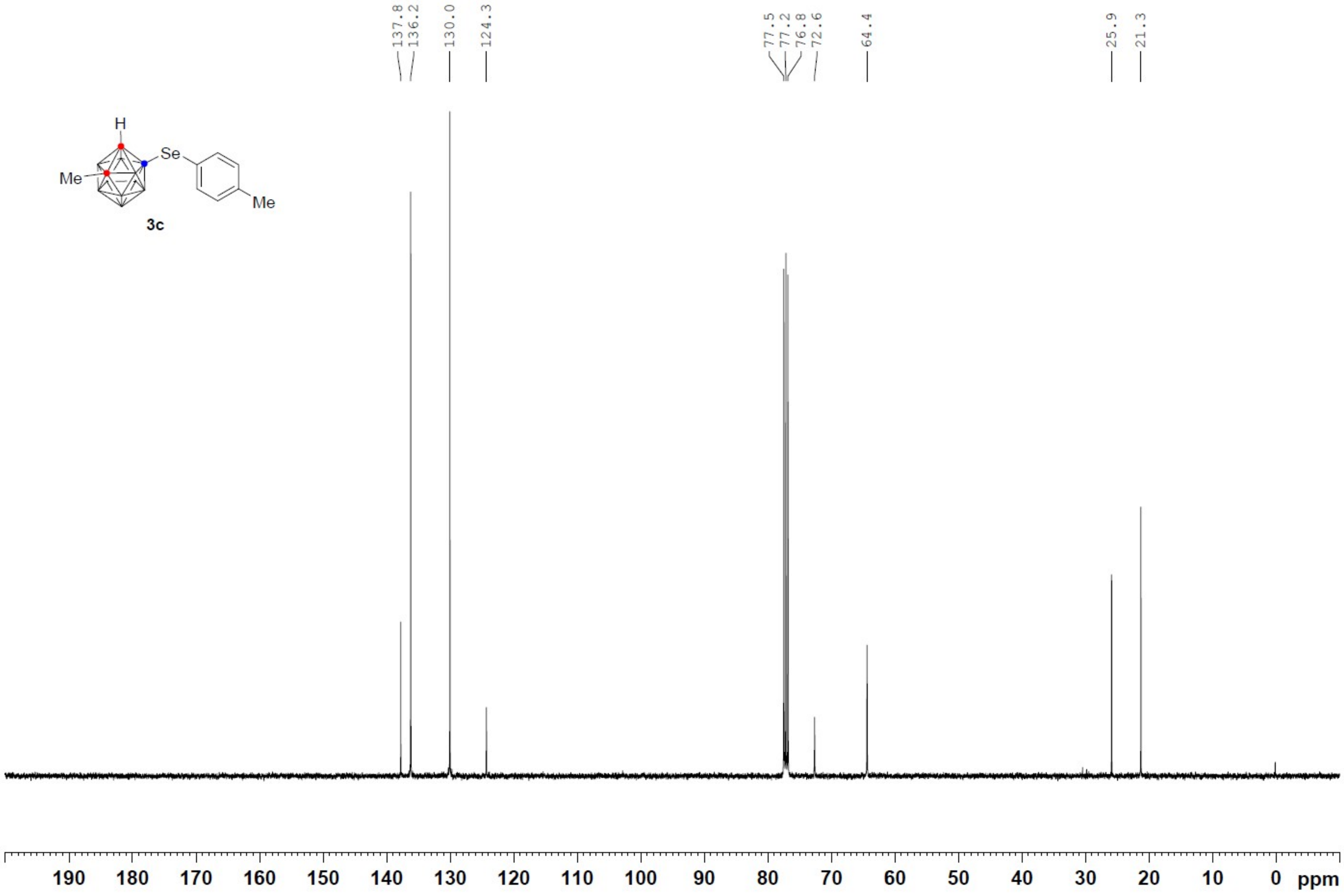

^{11}B NMR (128 MHz, $CDCl_3$)

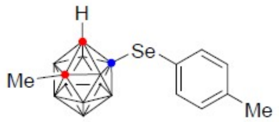

3c

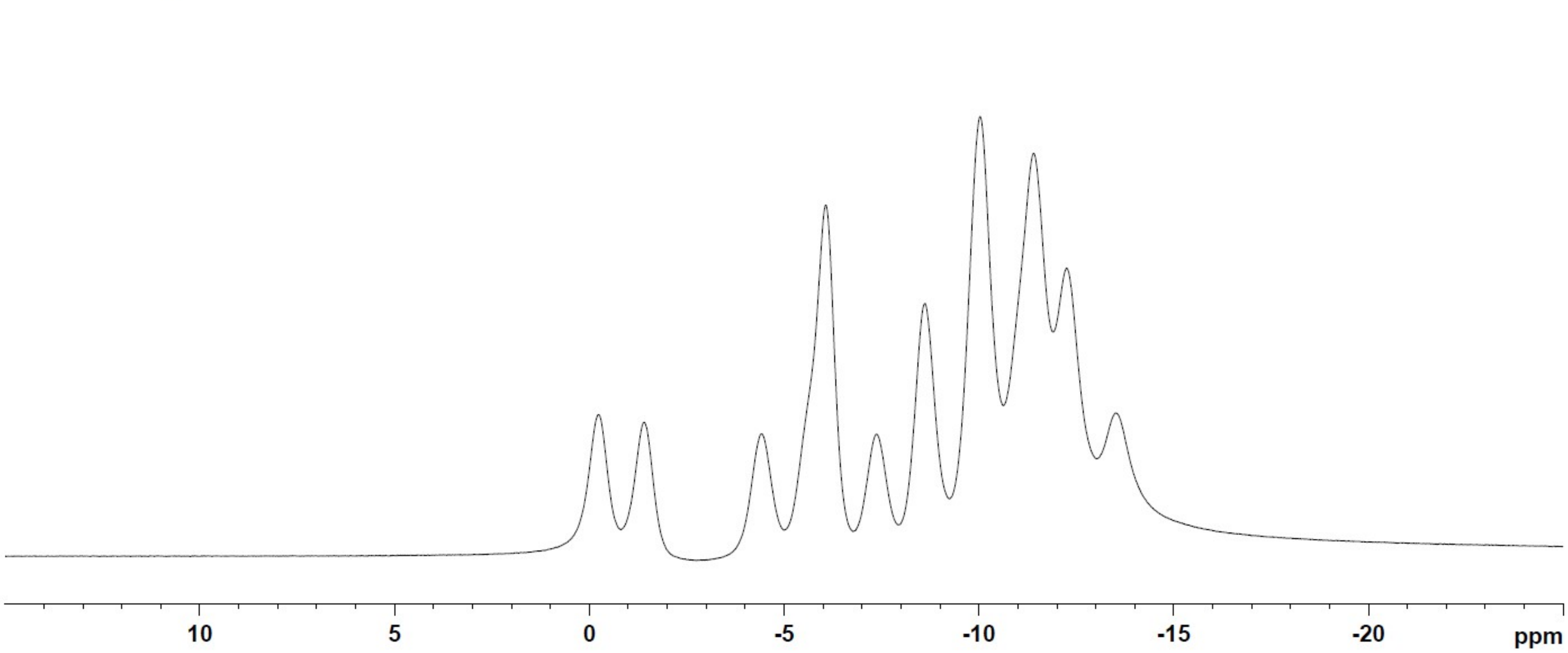

$^{11}B\{^1H\}$ NMR (128 MHz, $CDCl_3$)

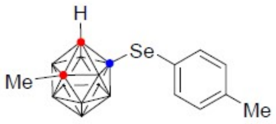

3c

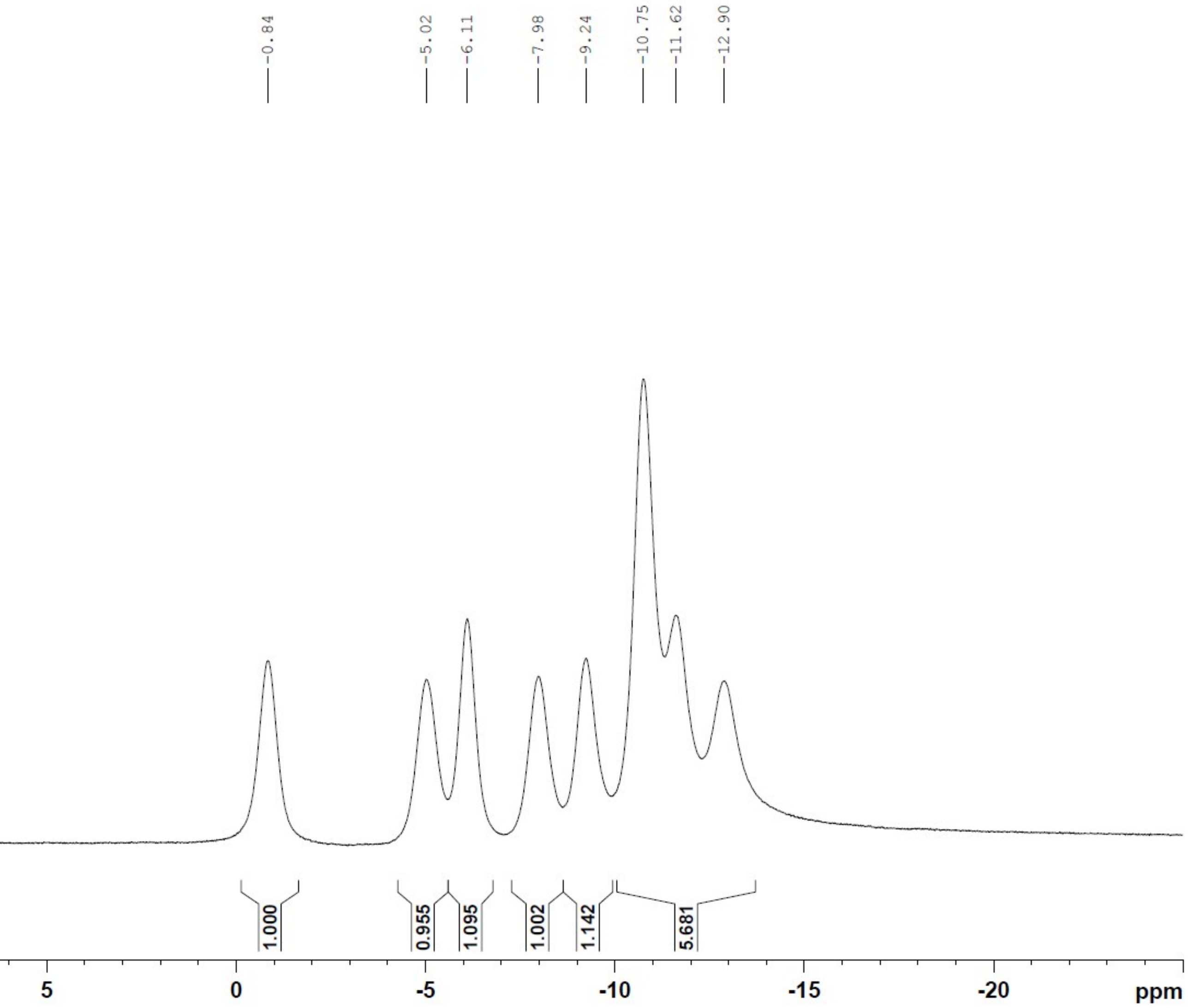

^1H NMR (400 MHz, $CDCl_3$)

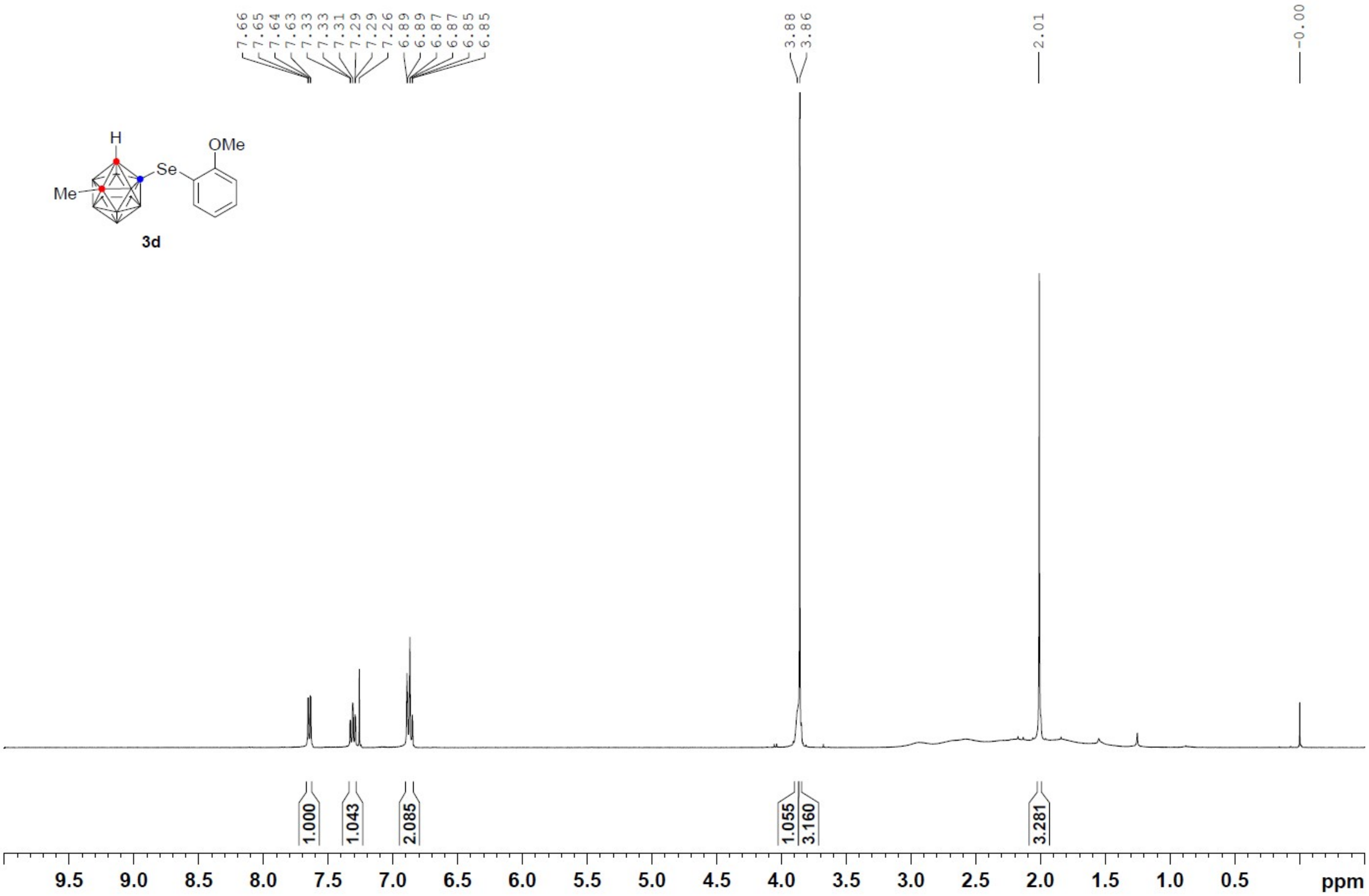

^{13}C NMR (100 MHz, $CDCl_3$)

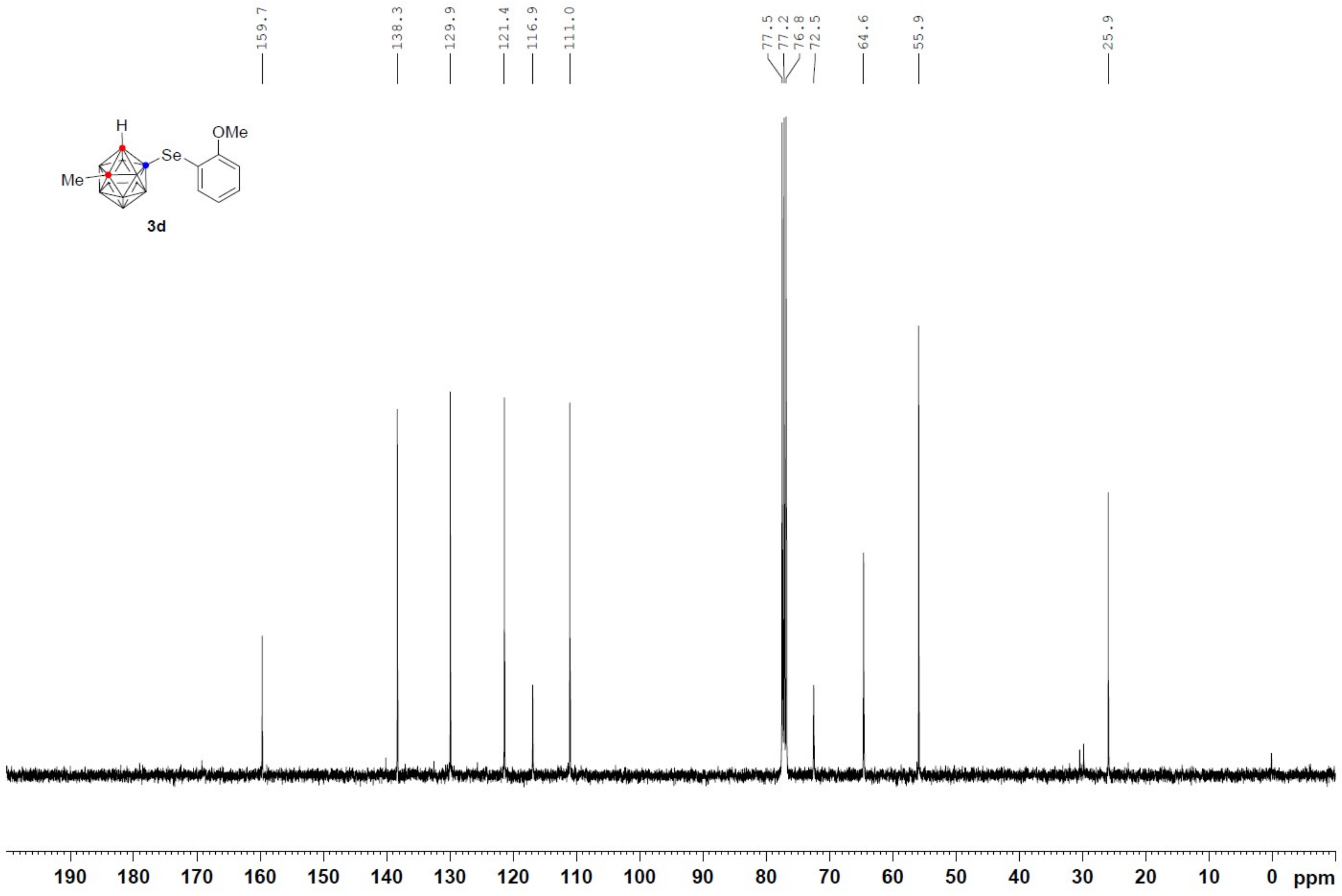

^{11}B NMR (128 MHz, $CDCl_3$)

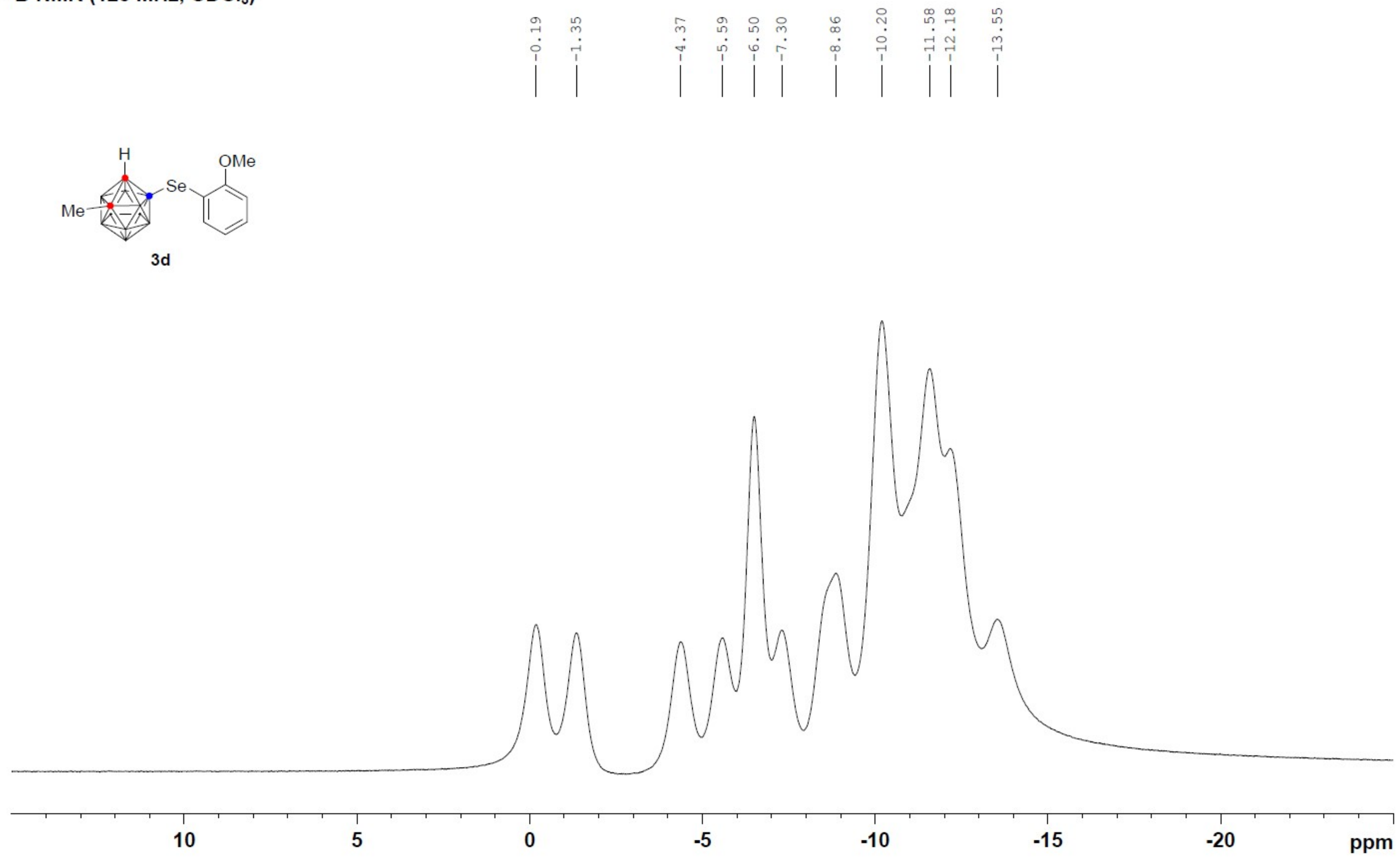

3d

$^{11}B\{^1H\}$ NMR (128 MHz, $CDCl_3$)

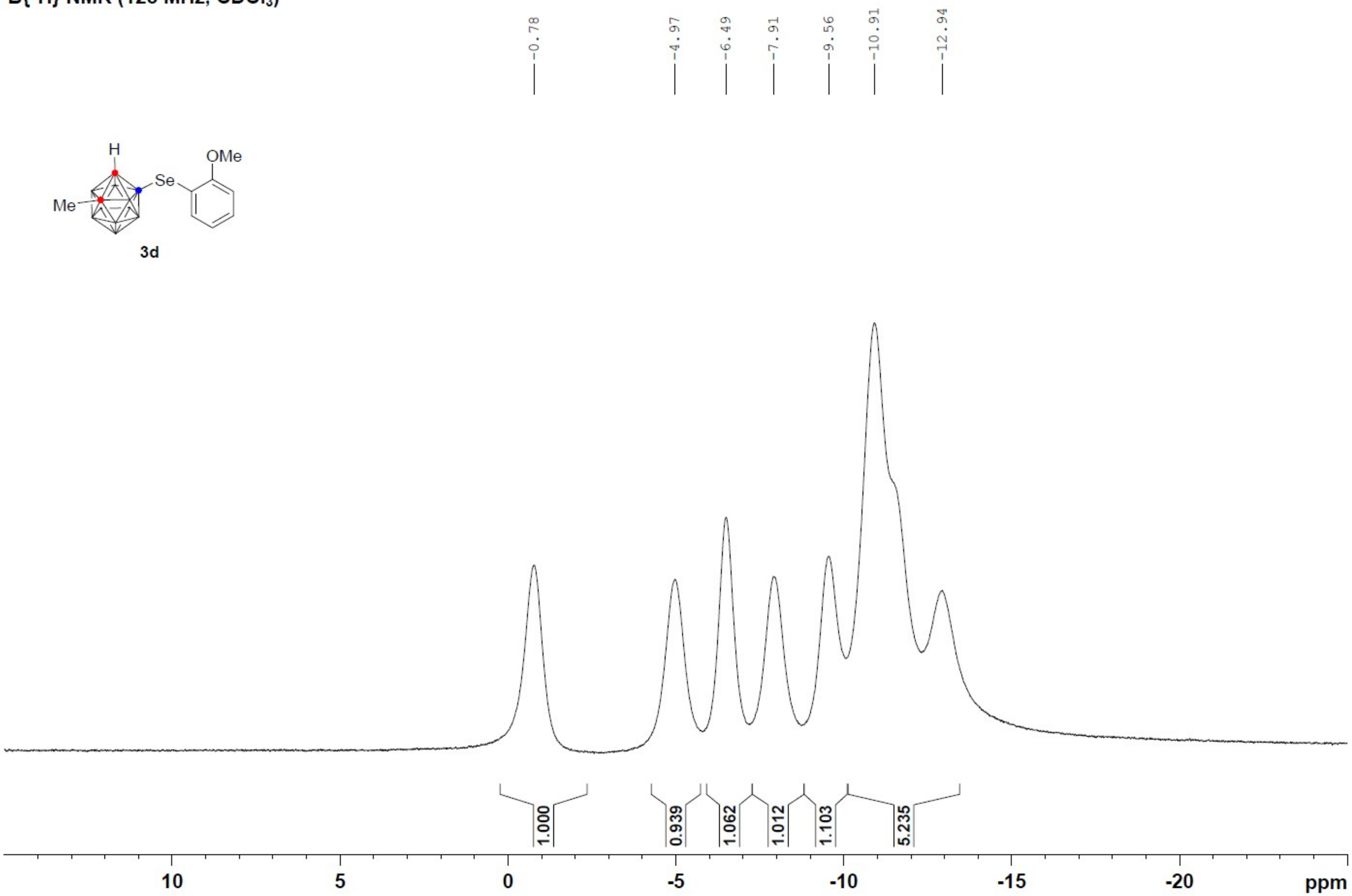

^{1}H NMR (400 MHz, $CDCl_3$)

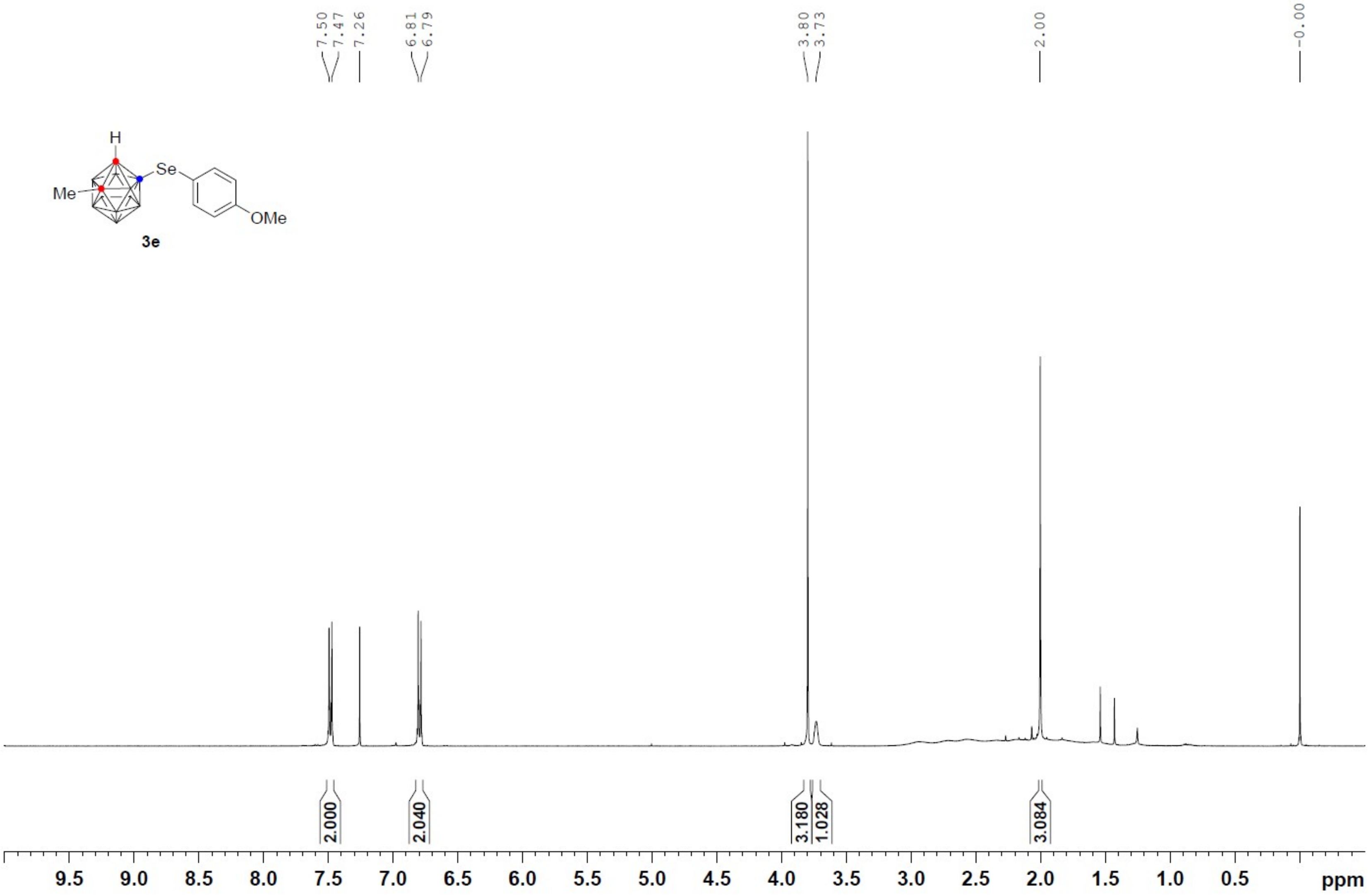

^{13}C NMR (100 MHz, $CDCl_3$)

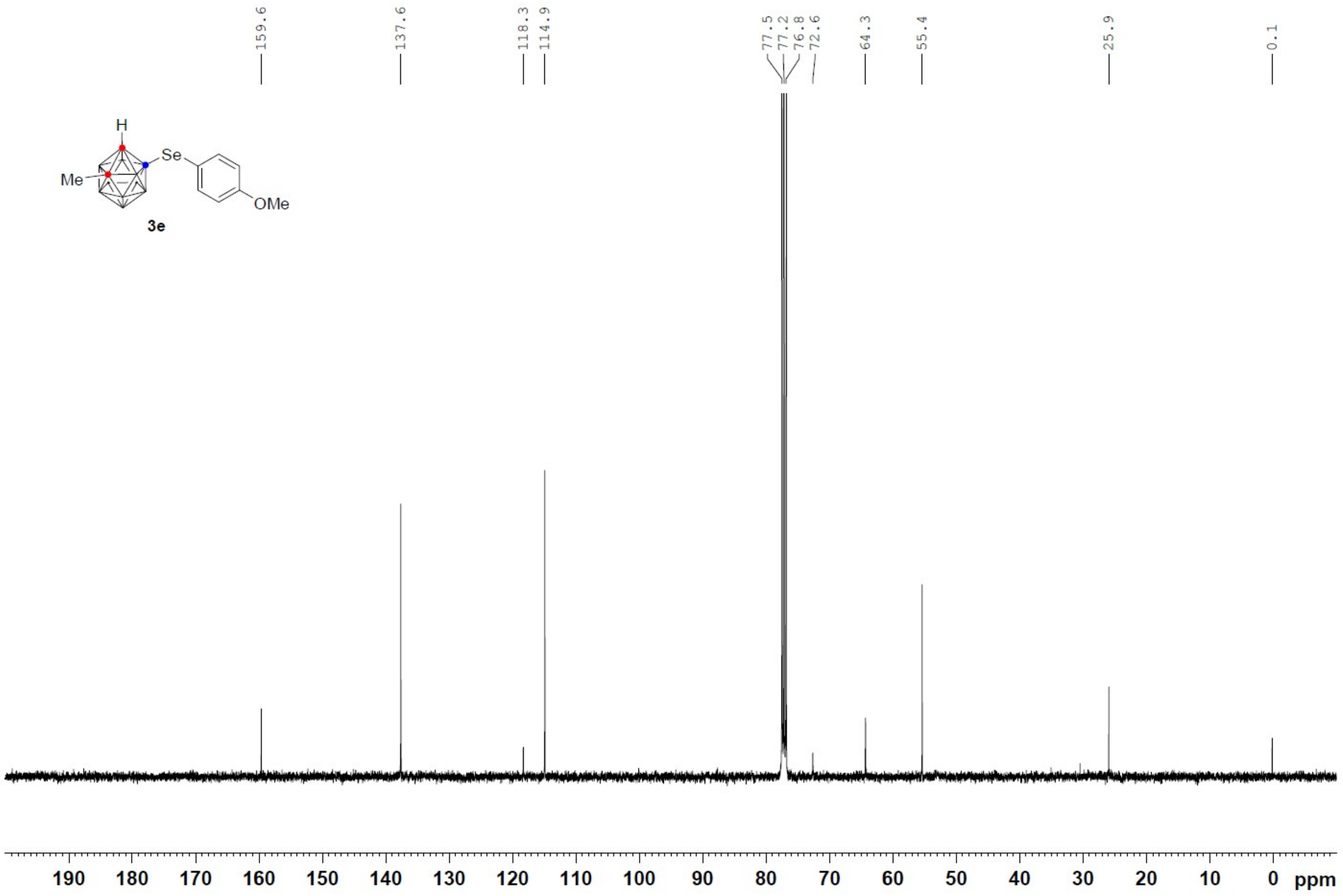

^{11}B NMR (128 MHz, $CDCl_3$)

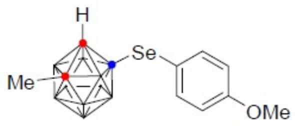

3e

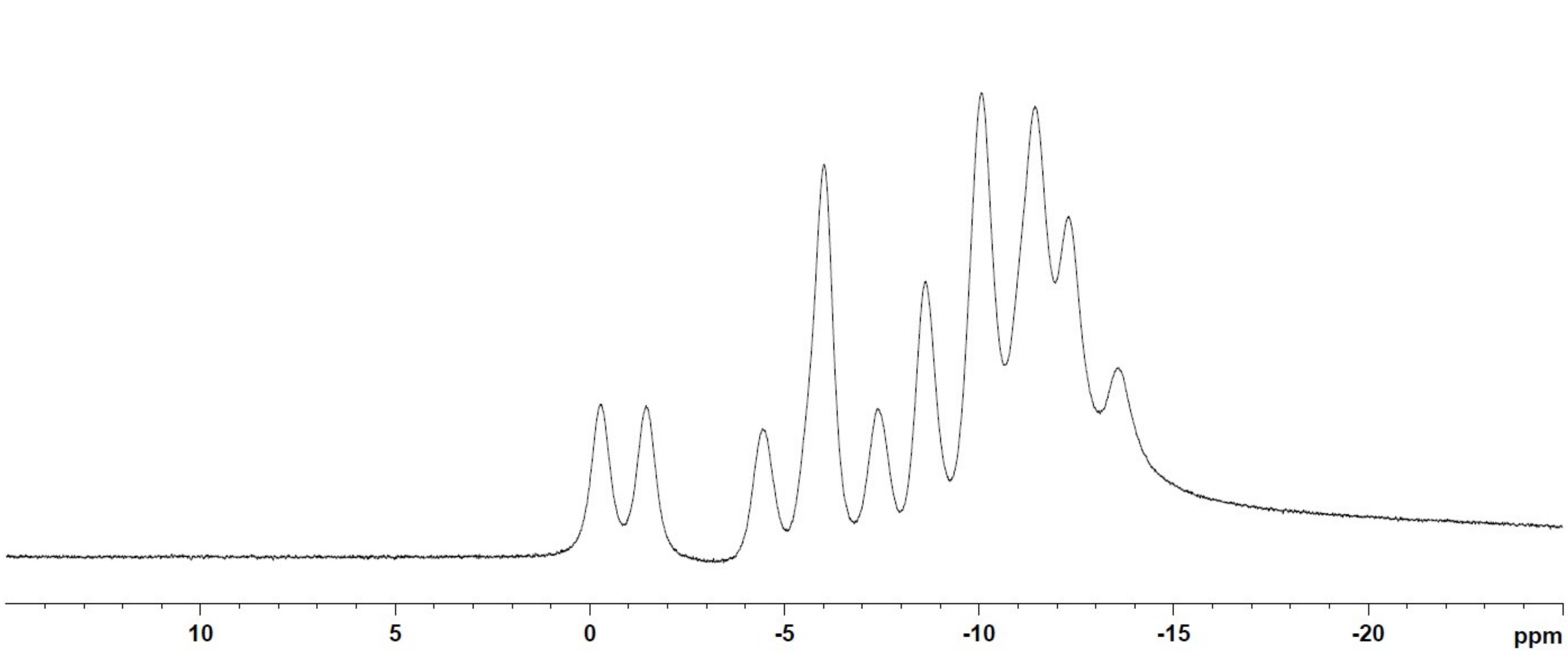

$^{11}B\{^1H\}$ NMR (128 MHz, $CDCl_3$)

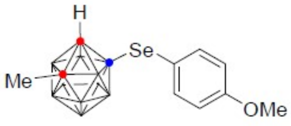

3e

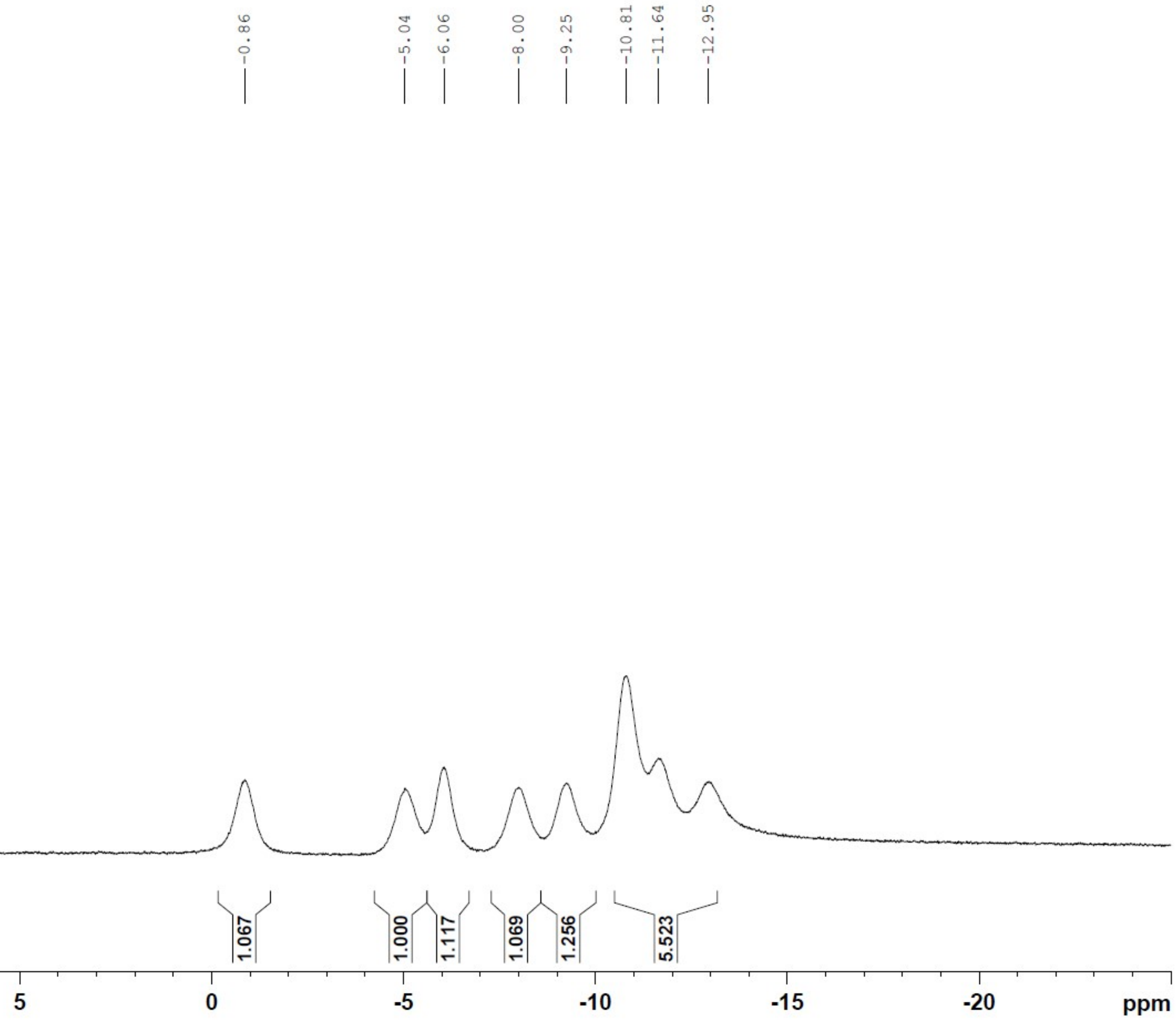

^{1}H NMR (400 MHz, $CDCl_3$)

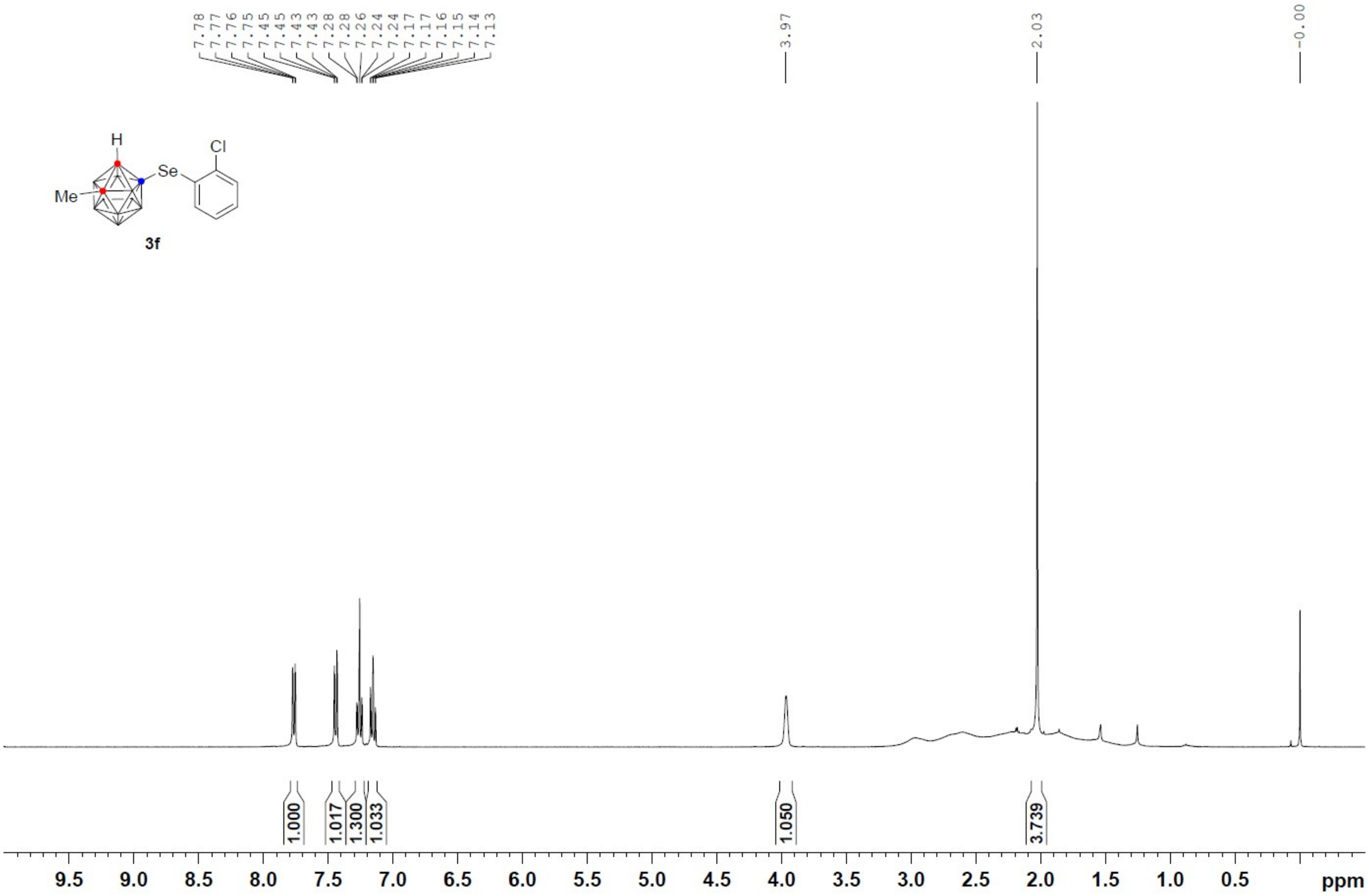

^{13}C NMR (100 MHz, $CDCl_3$)

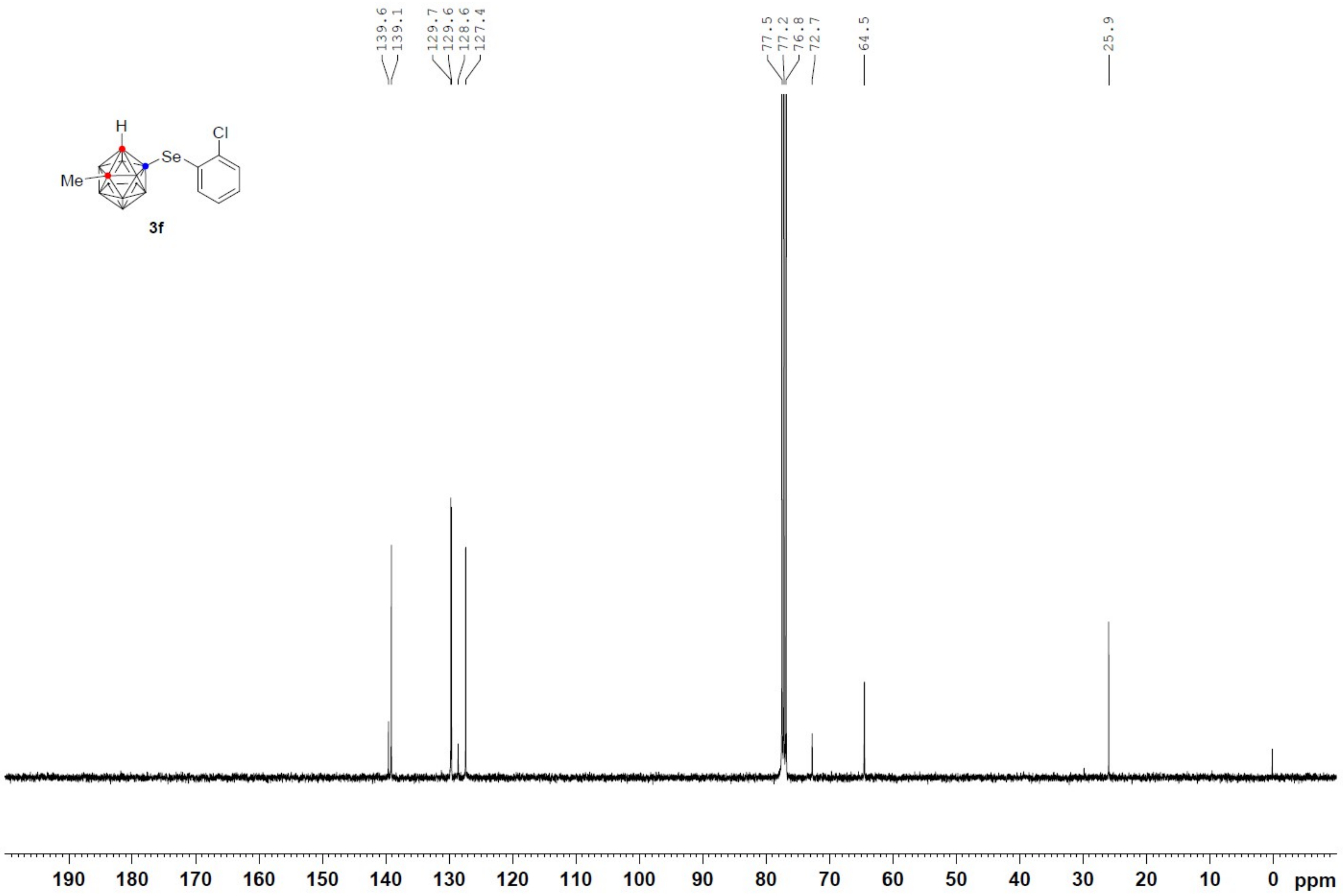

3f

^{11}B NMR (128 MHz, $CDCl_3$)

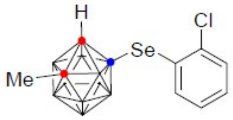

3f

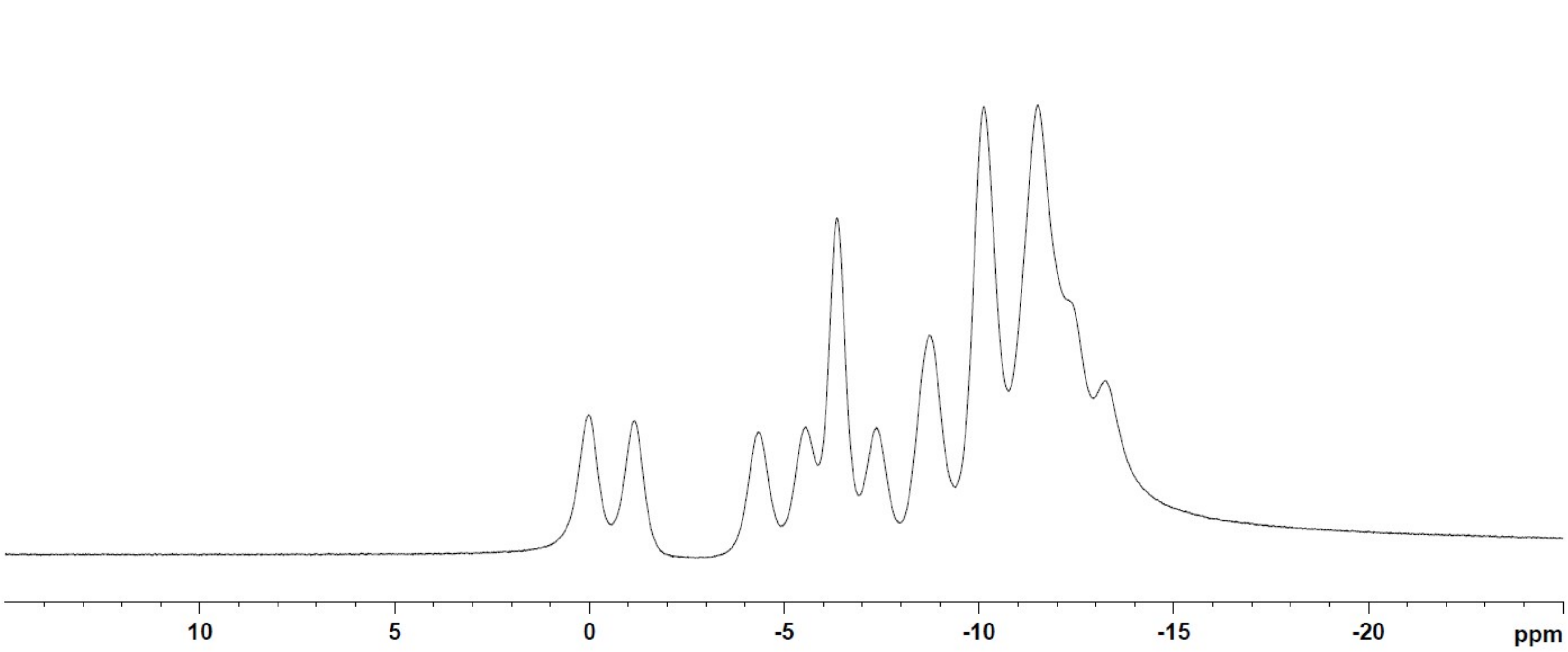

$^{11}B\{^{1}H\}$ NMR (128 MHz, $CDCl_3$)

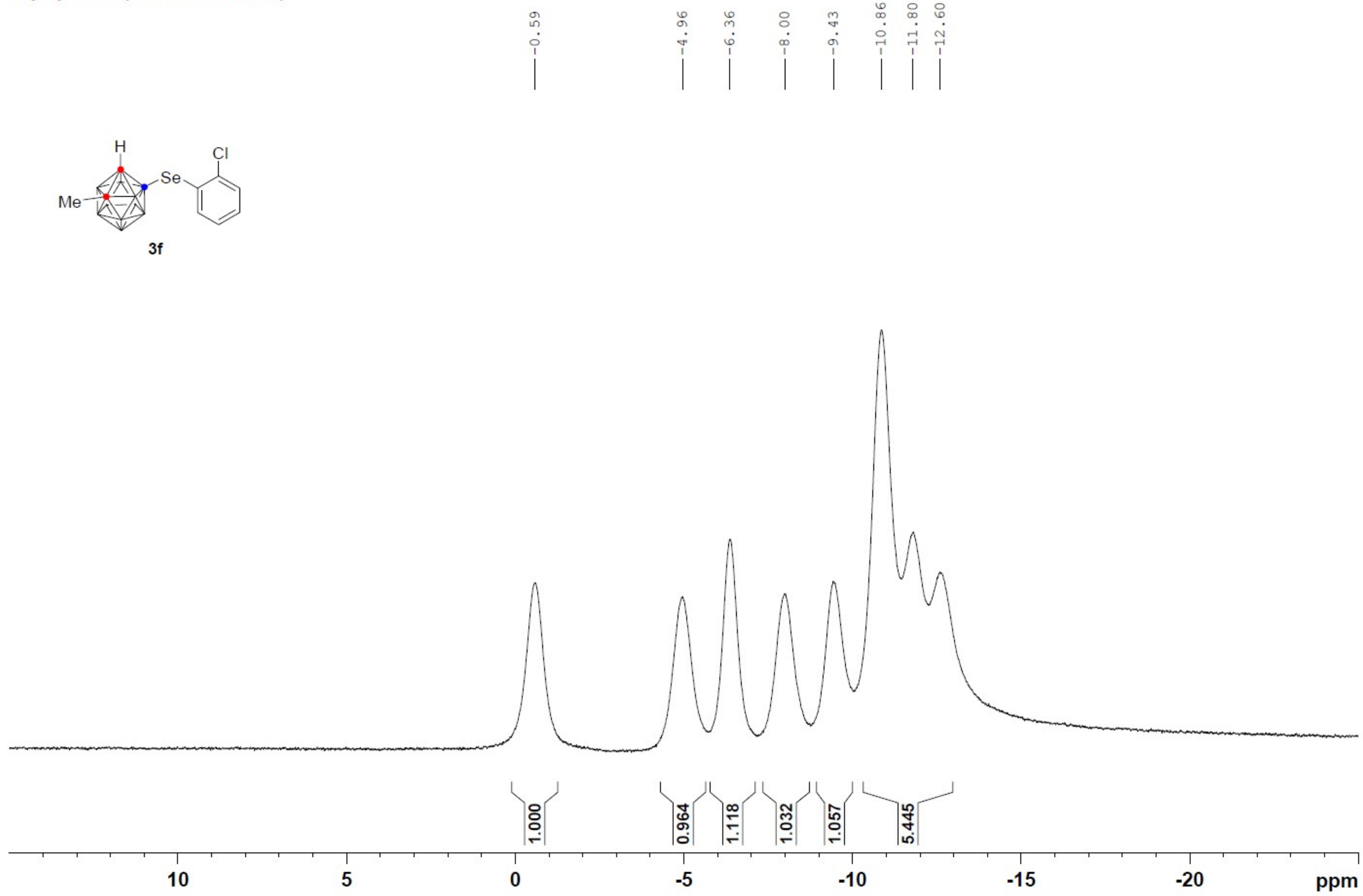

^1H NMR (400 MHz, $CDCl_3$)

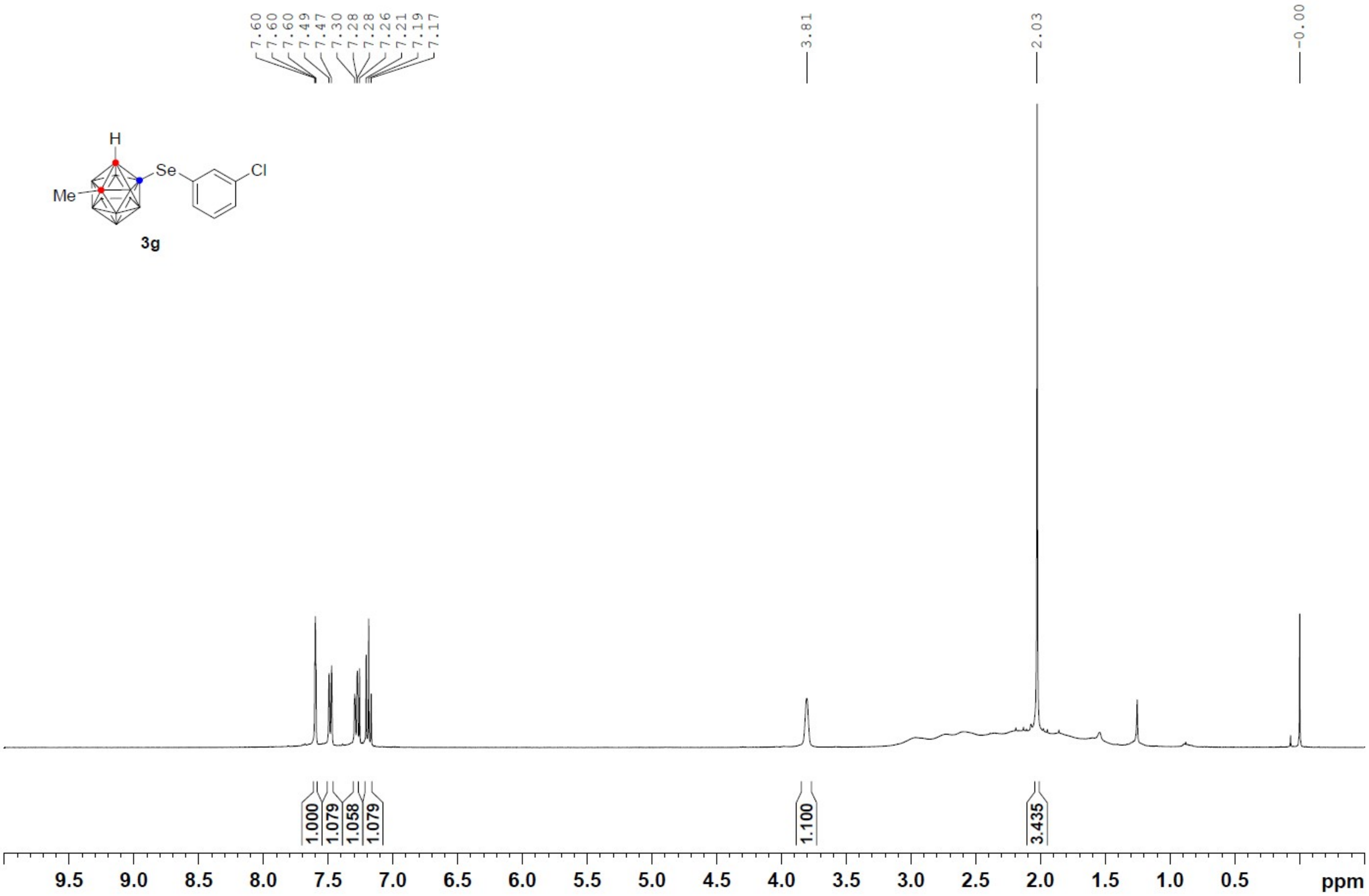

^{13}C NMR (100 MHz, $CDCl_3$)

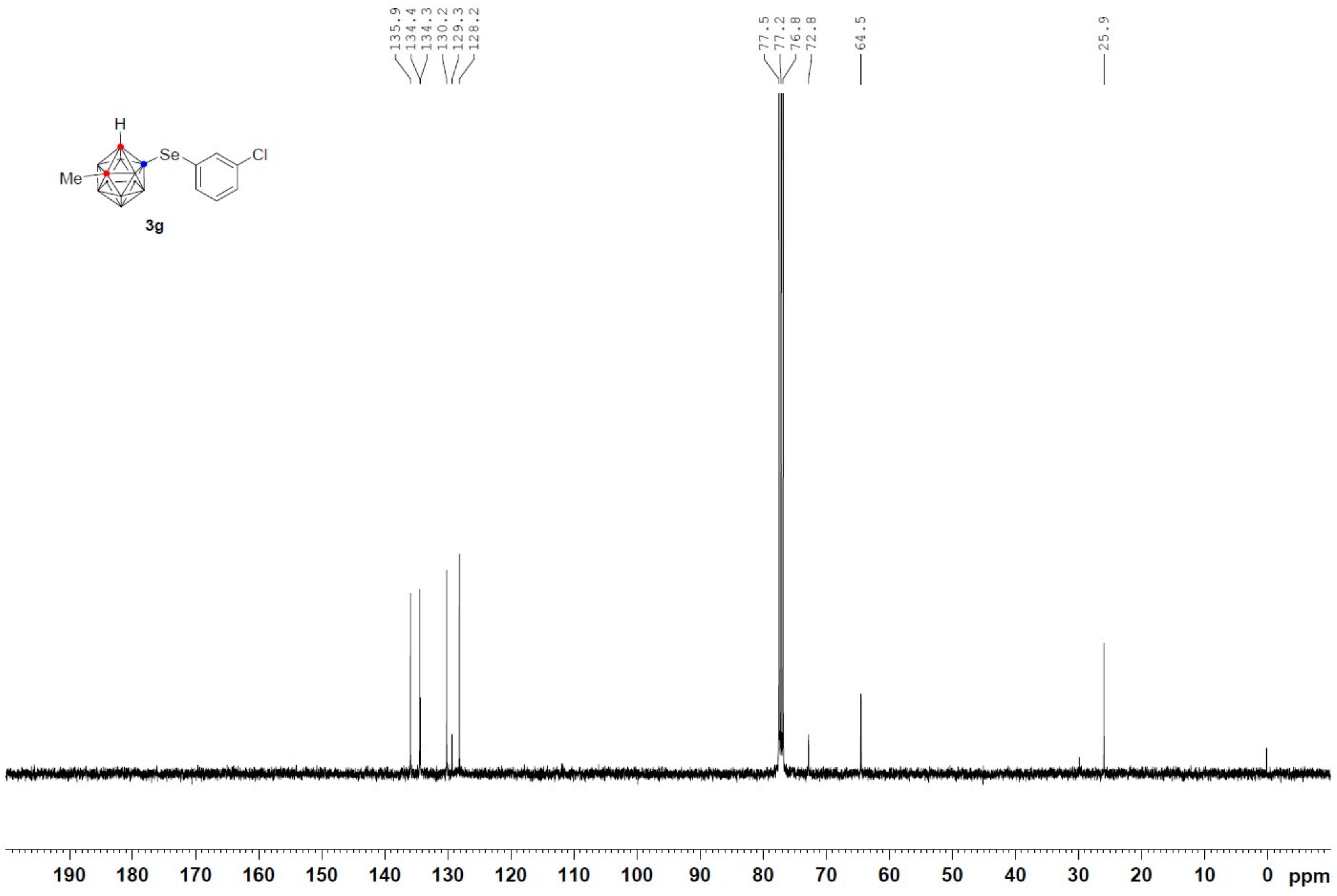

3g

^{11}B NMR (128 MHz, $CDCl_3$)

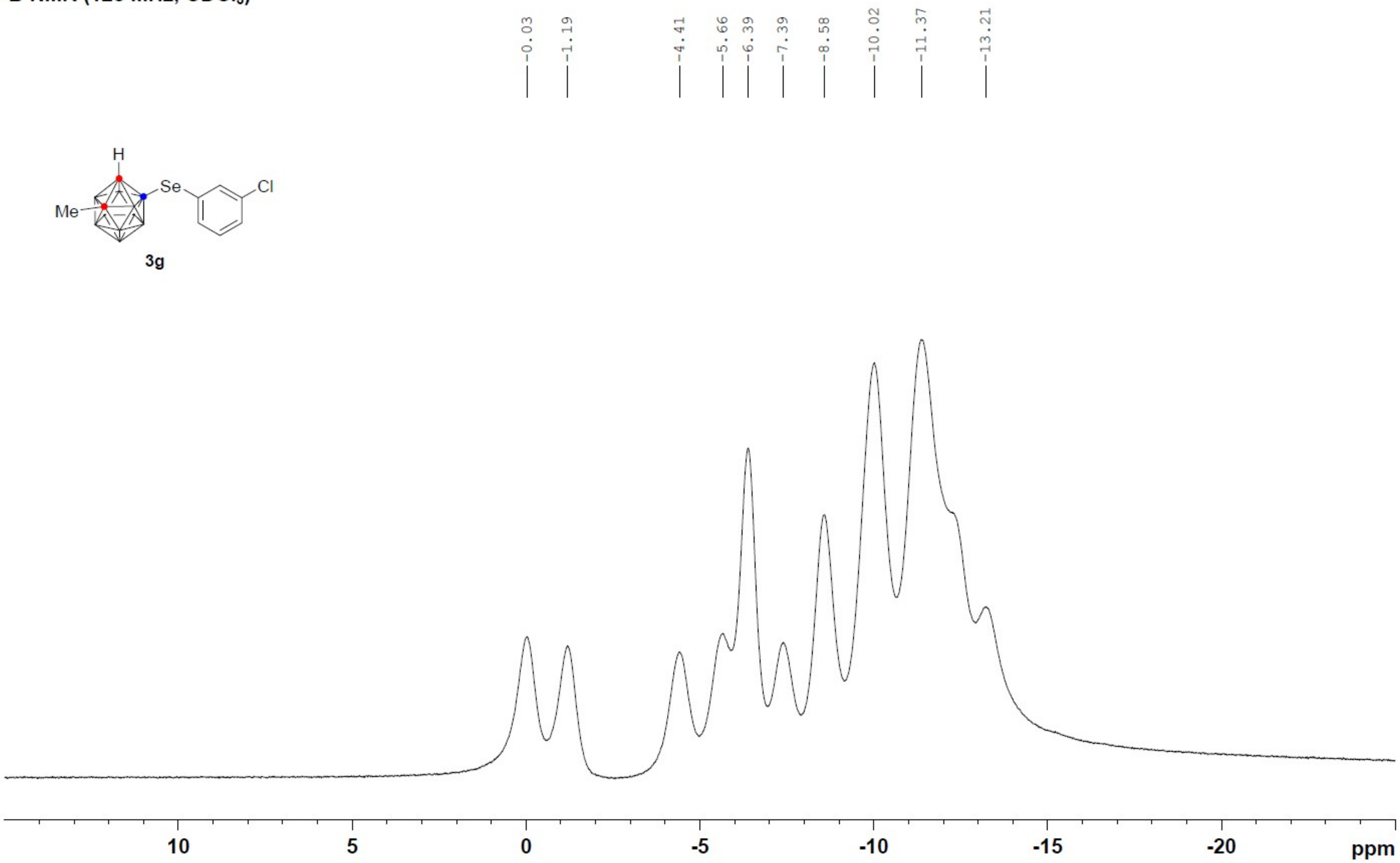

$^{11}B\{^{1}H\}$ NMR (128 MHz, $CDCl_3$)

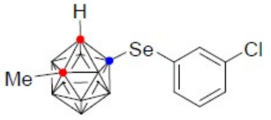

3g

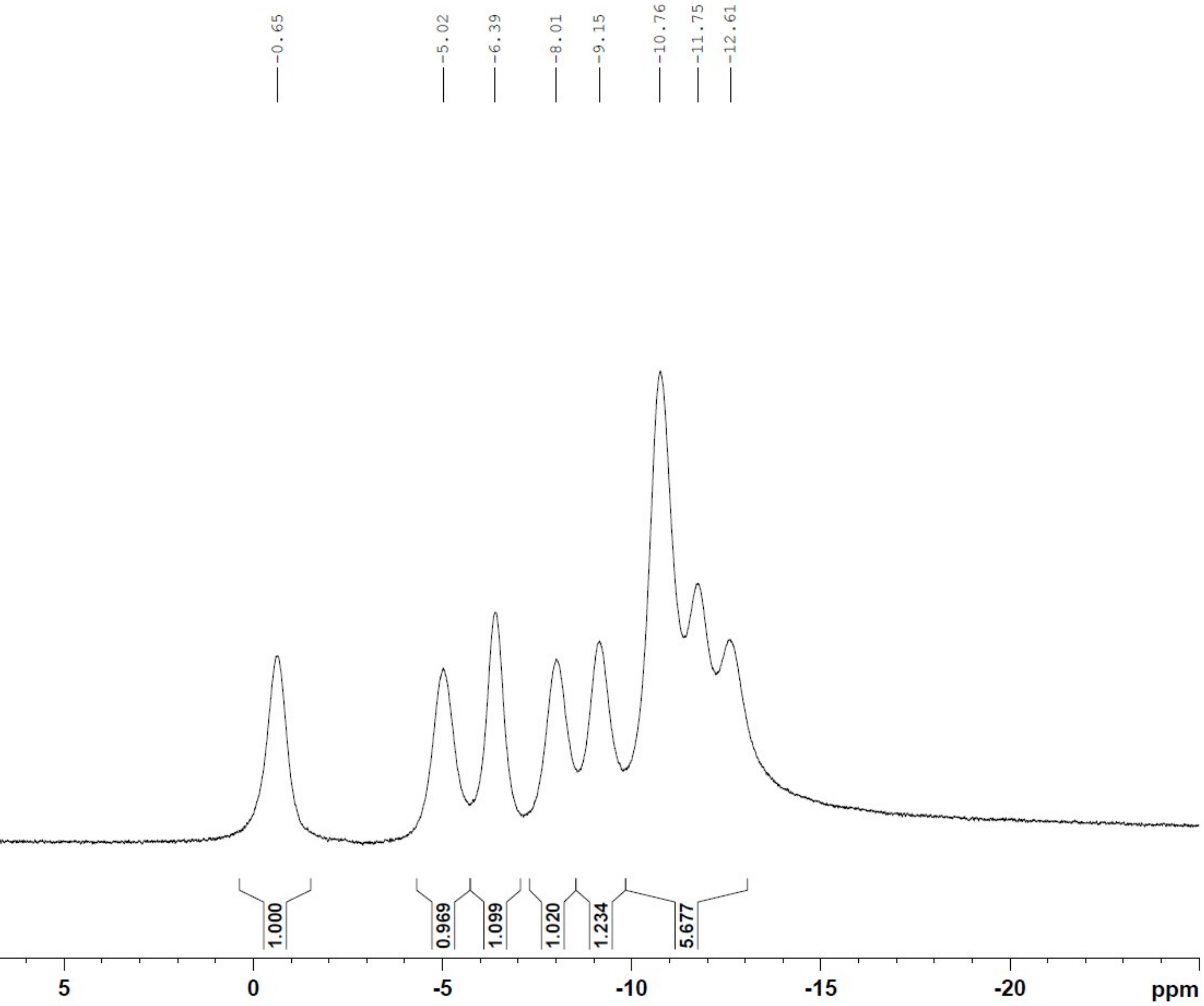

^{1}H NMR (400 MHz, $CDCl_3$)

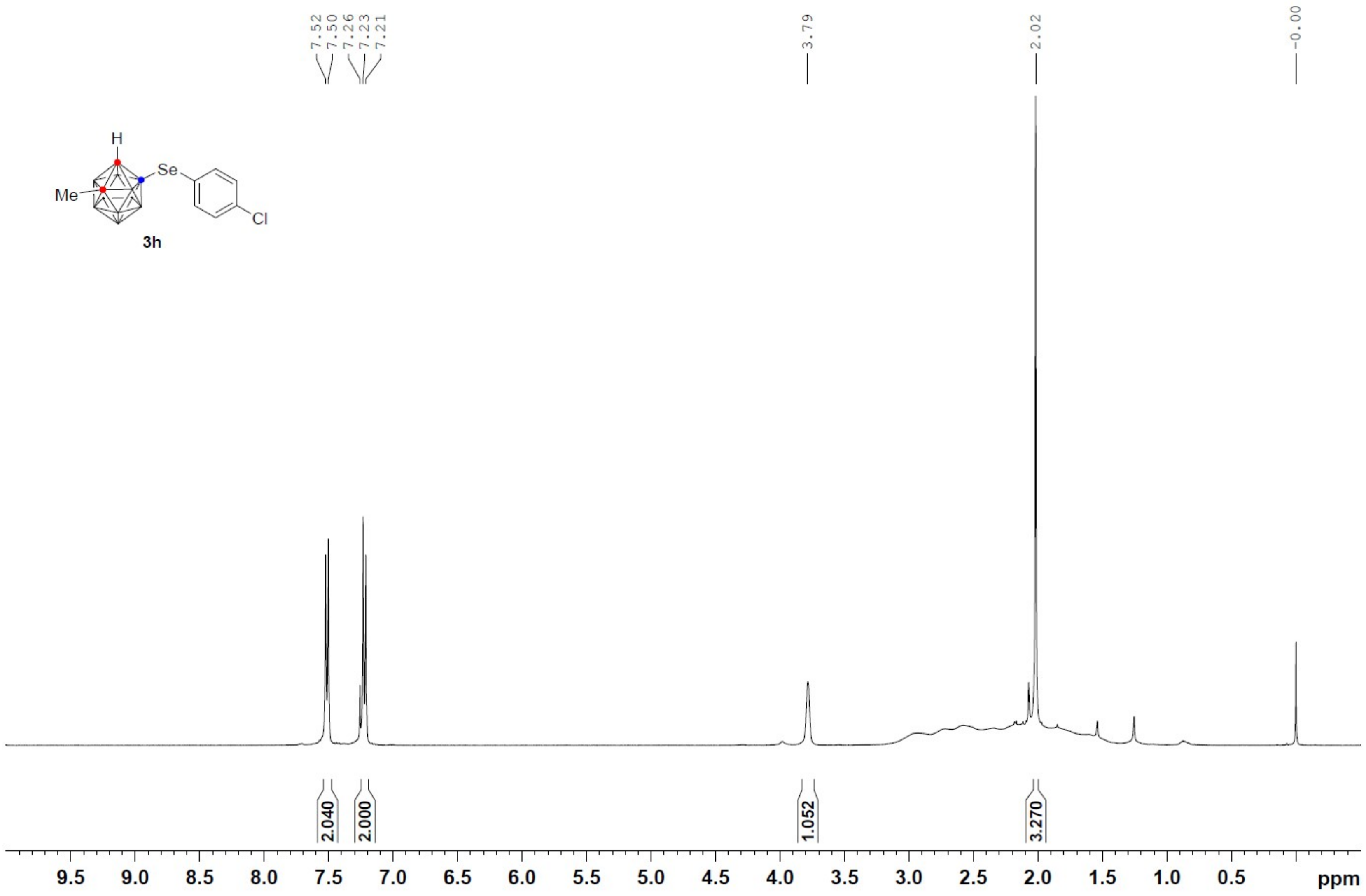

^{13}C NMR (100 MHz, $CDCl_3$)

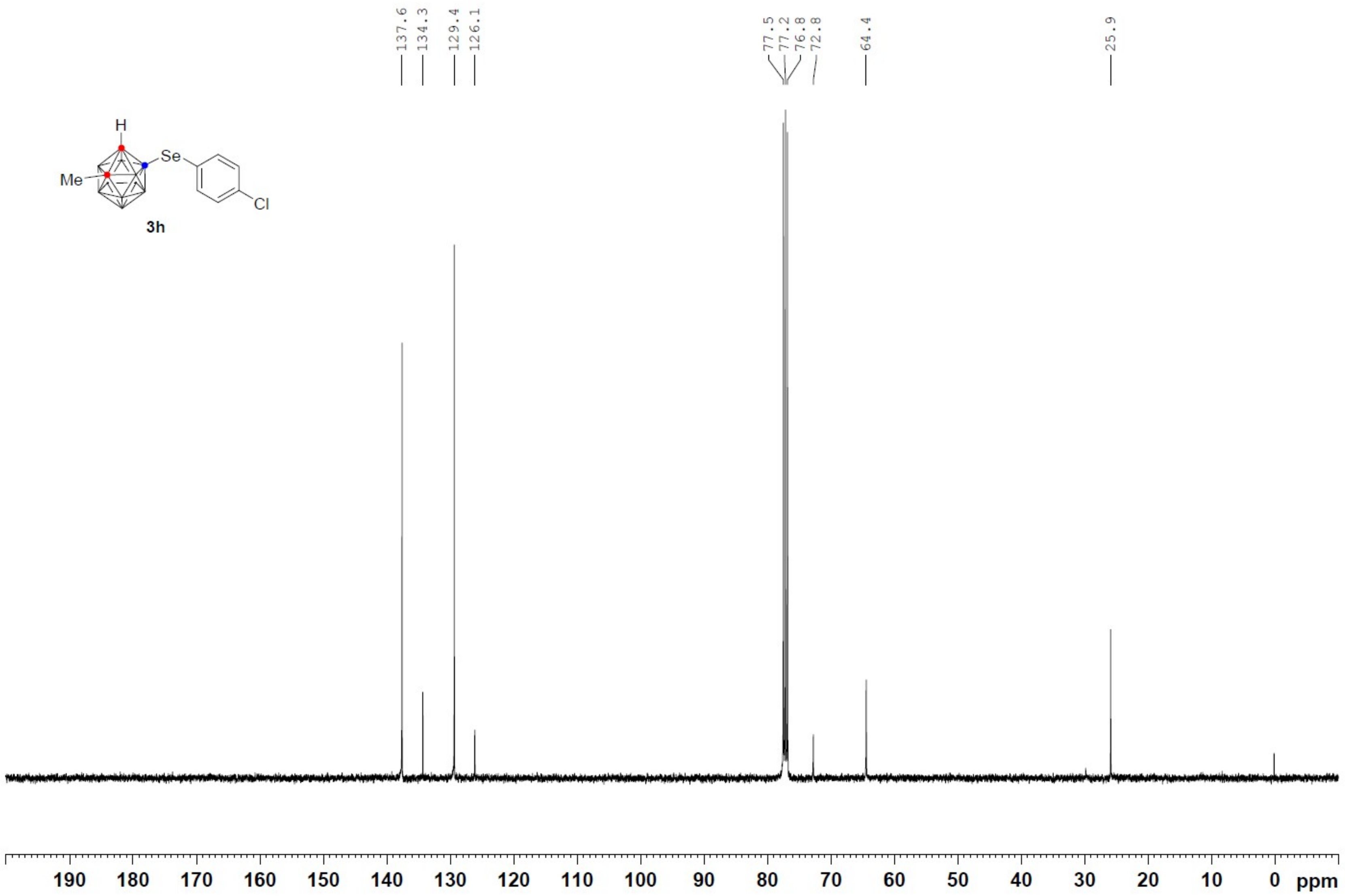

^{11}B NMR (128 MHz, CDCl$_3$)

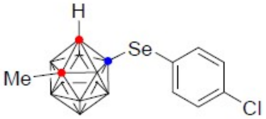

3h

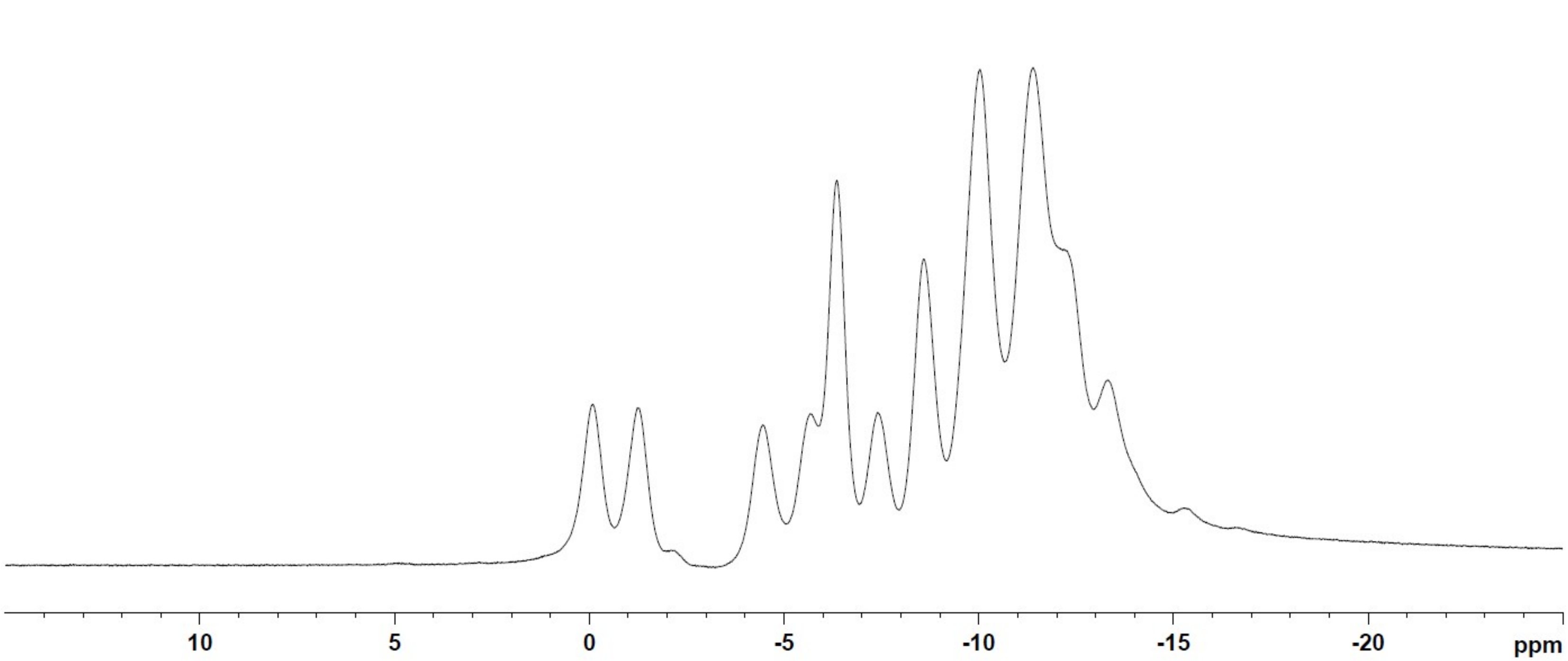

$^{11}B\{^1H\}$ NMR (128 MHz, $CDCl_3$)

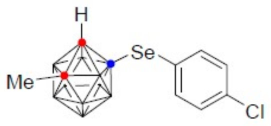

3h

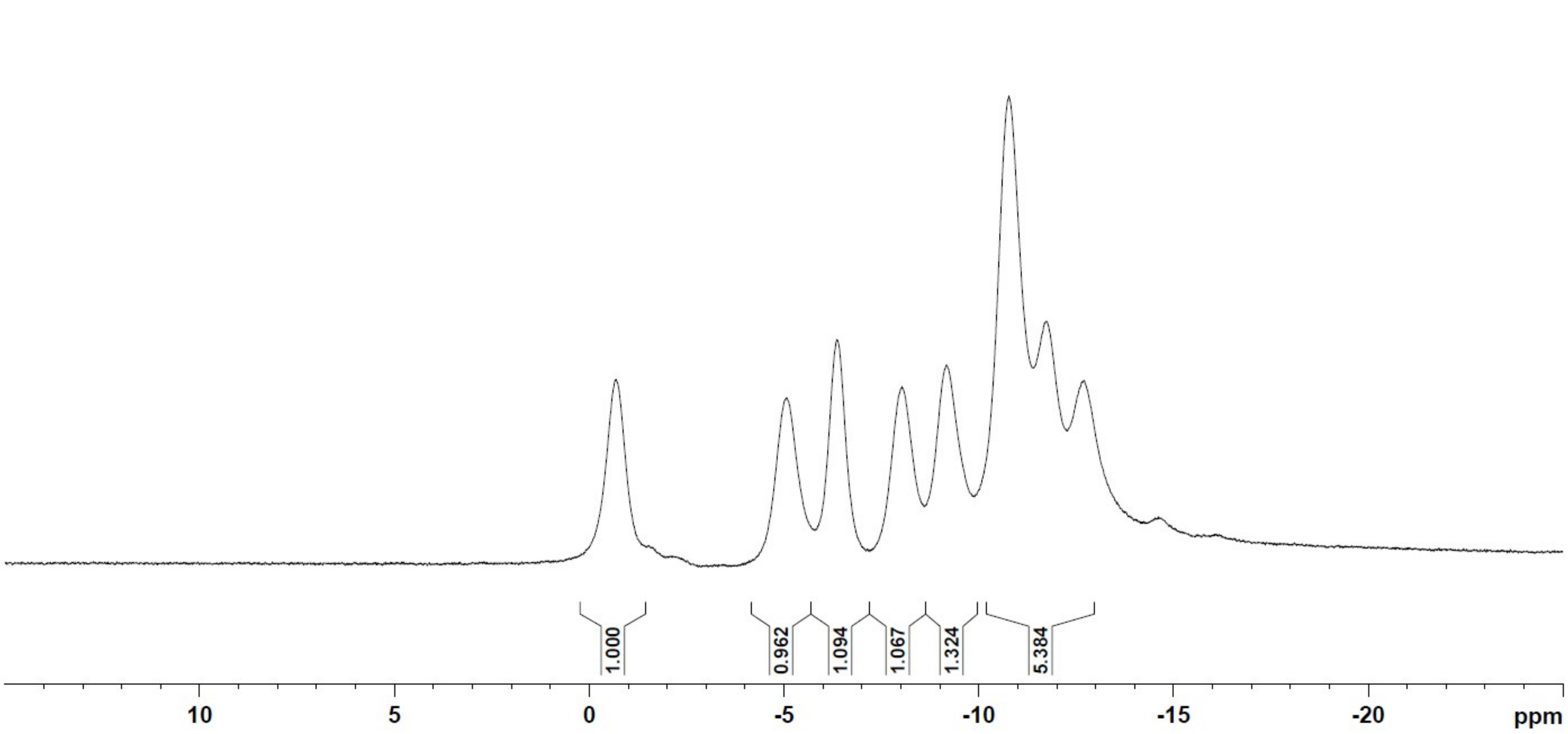

^{1}H NMR (400 MHz, $CDCl_3$)

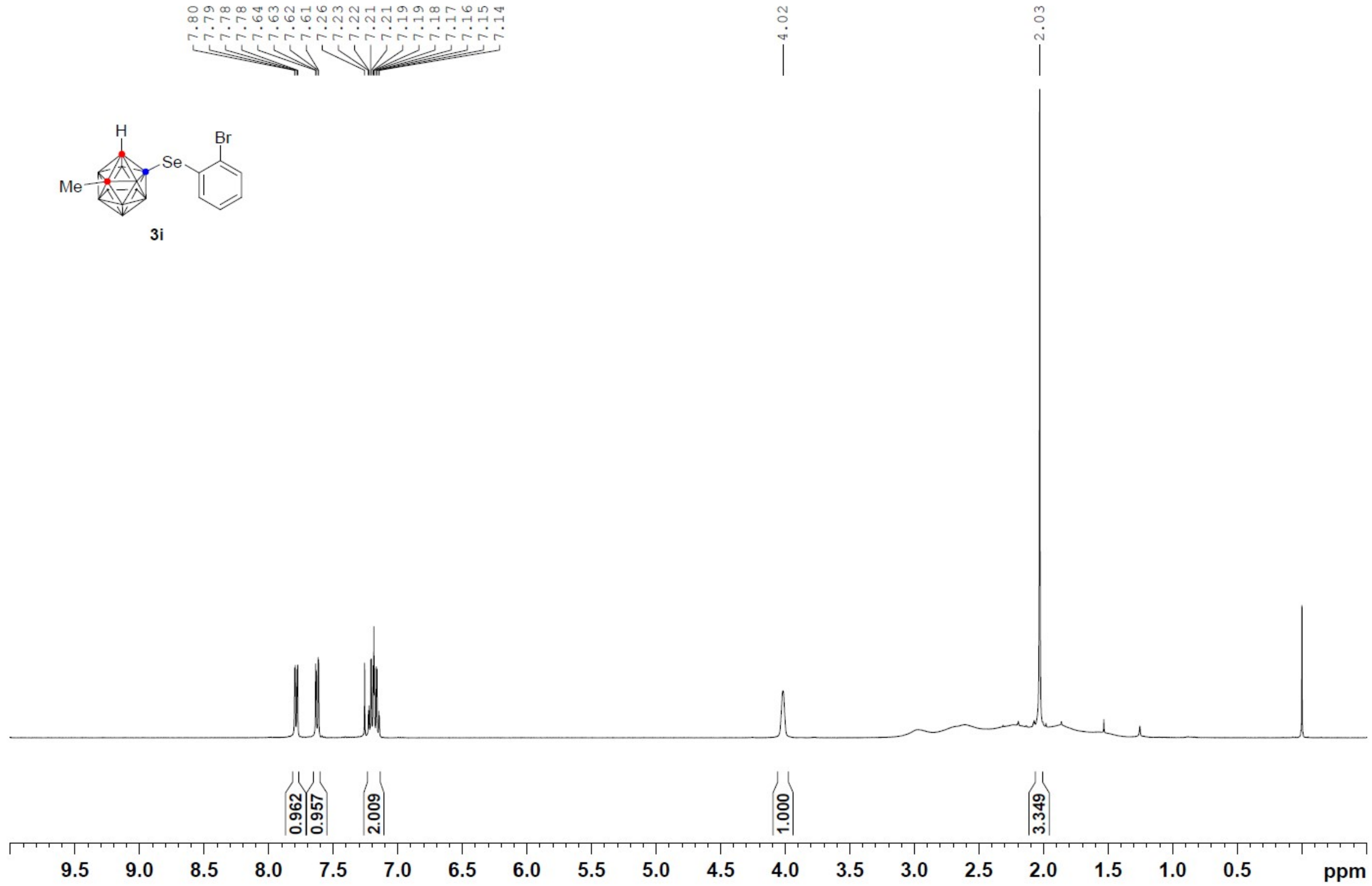

3i

^{13}C NMR (100 MHz, $CDCl_3$)

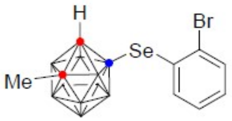

3i

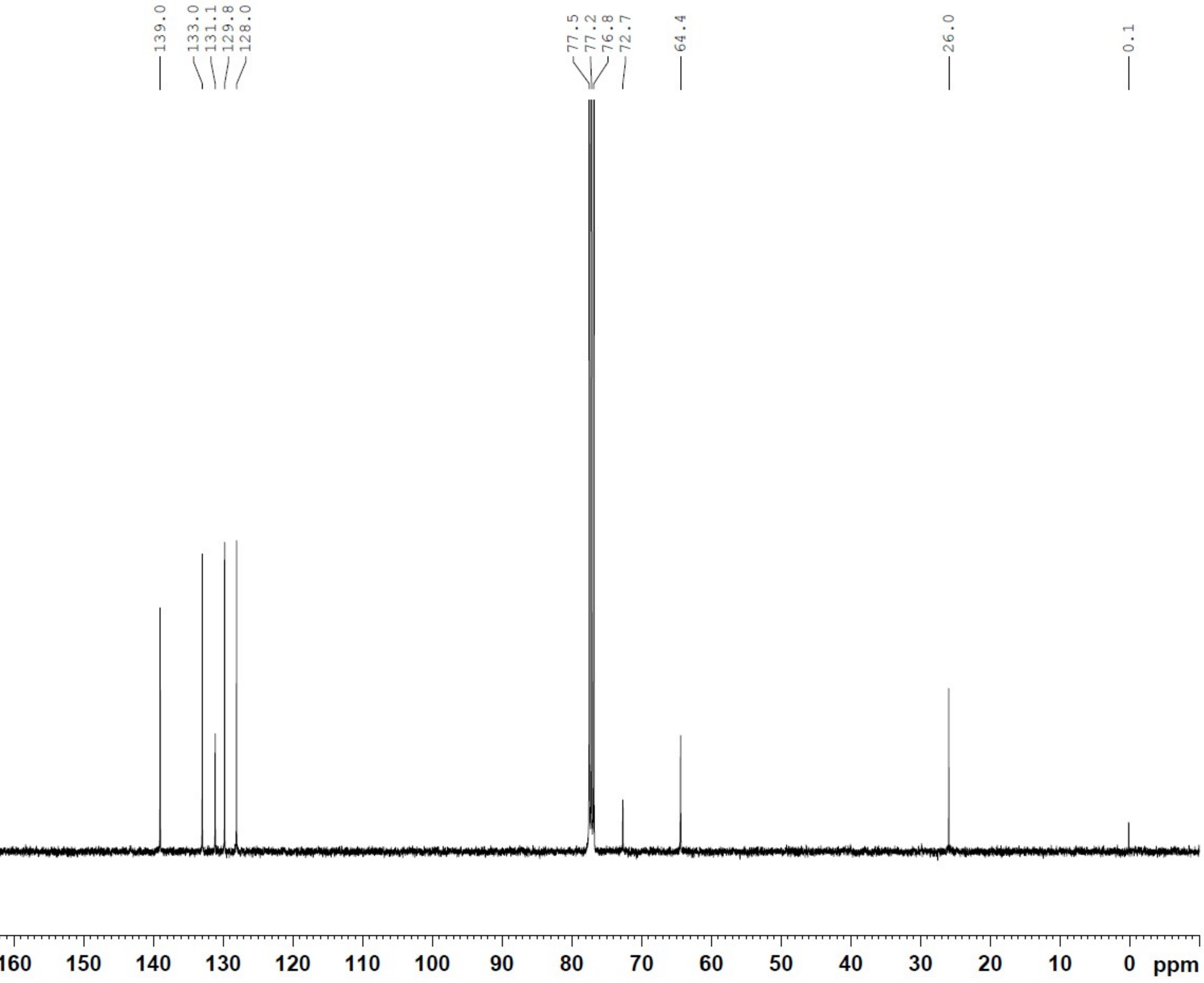

^{11}B NMR (128 MHz, $CDCl_3$)

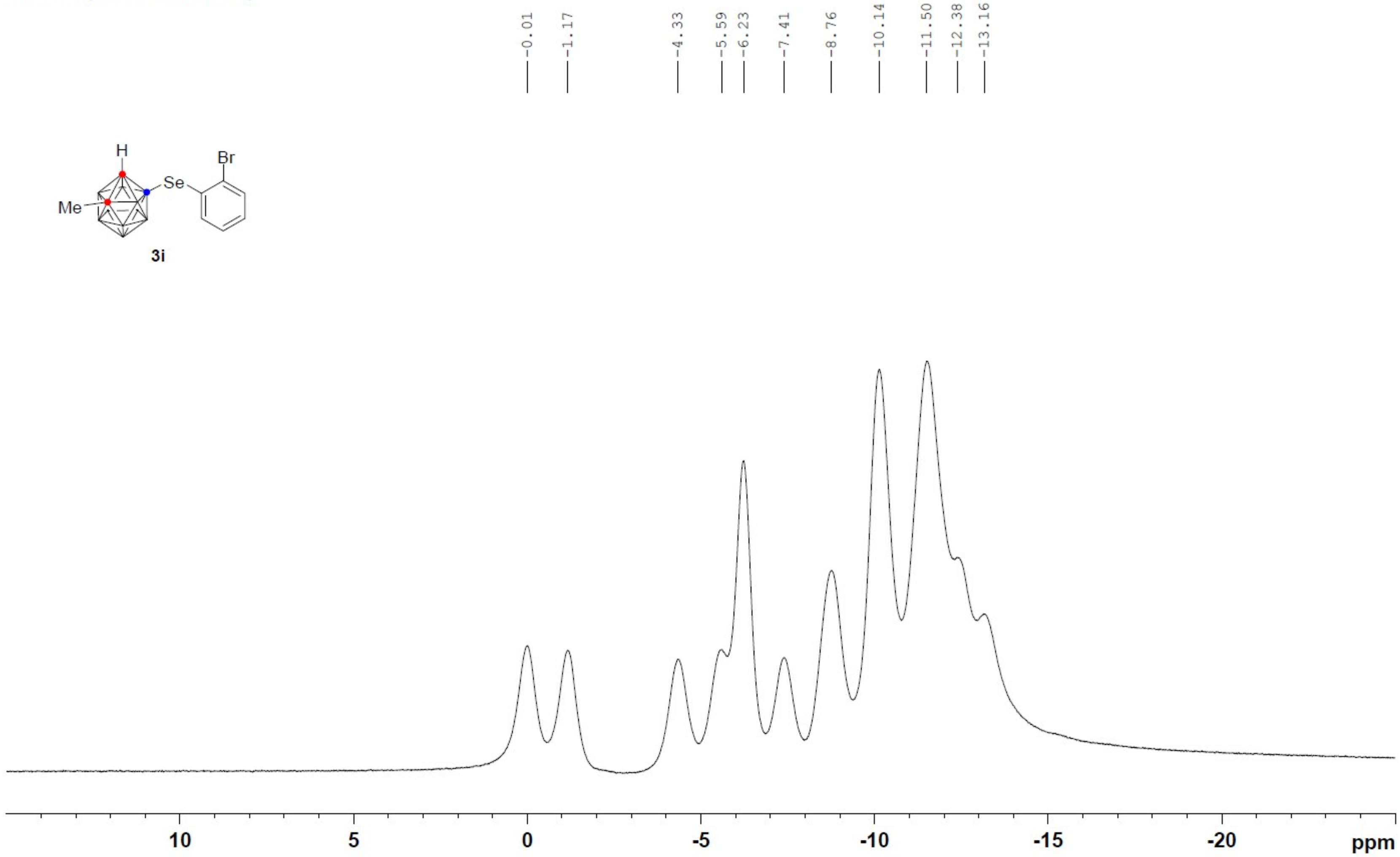

$^{11}B\{^1H\}$ NMR (128 MHz, $CDCl_3$)

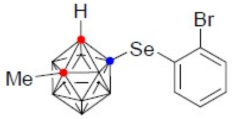

3i

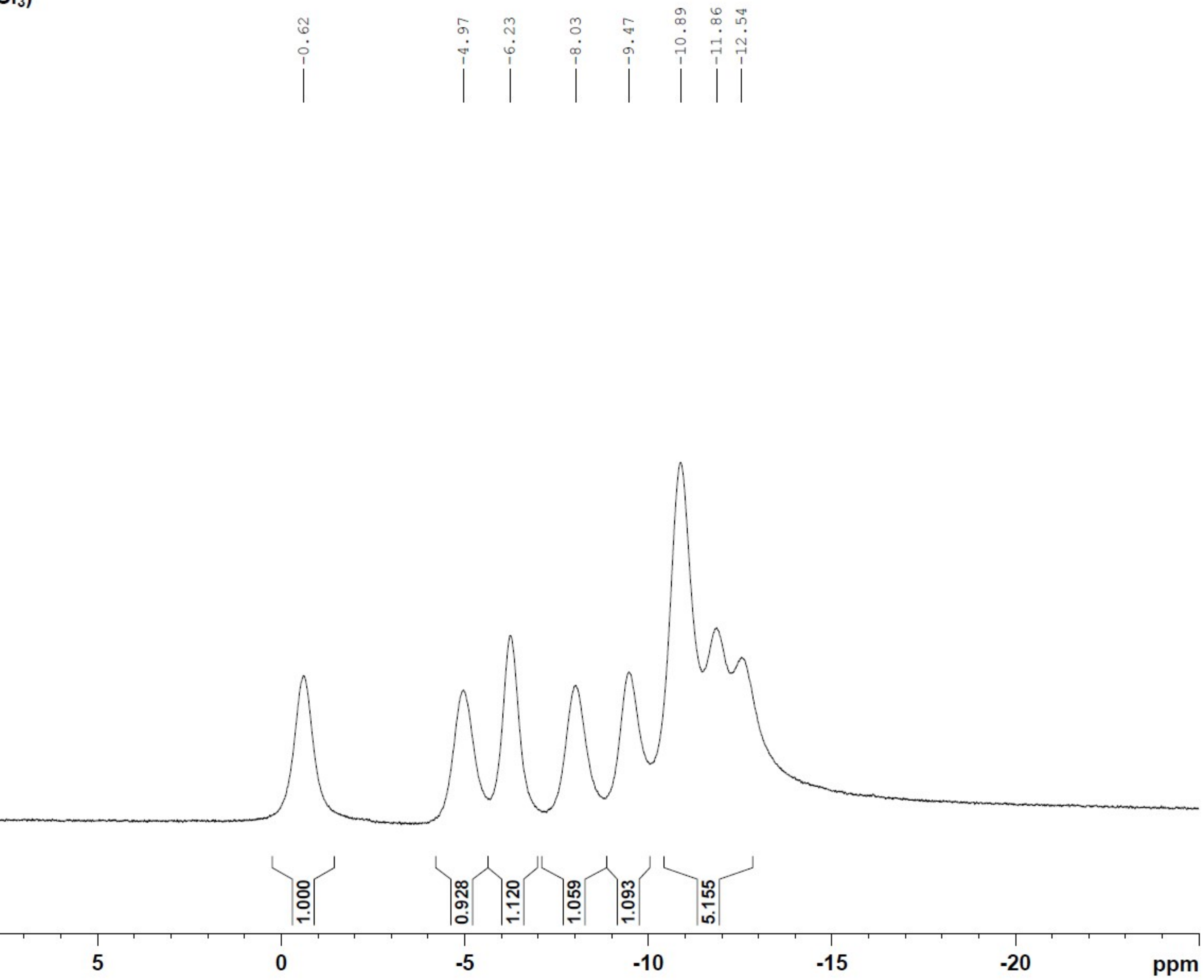

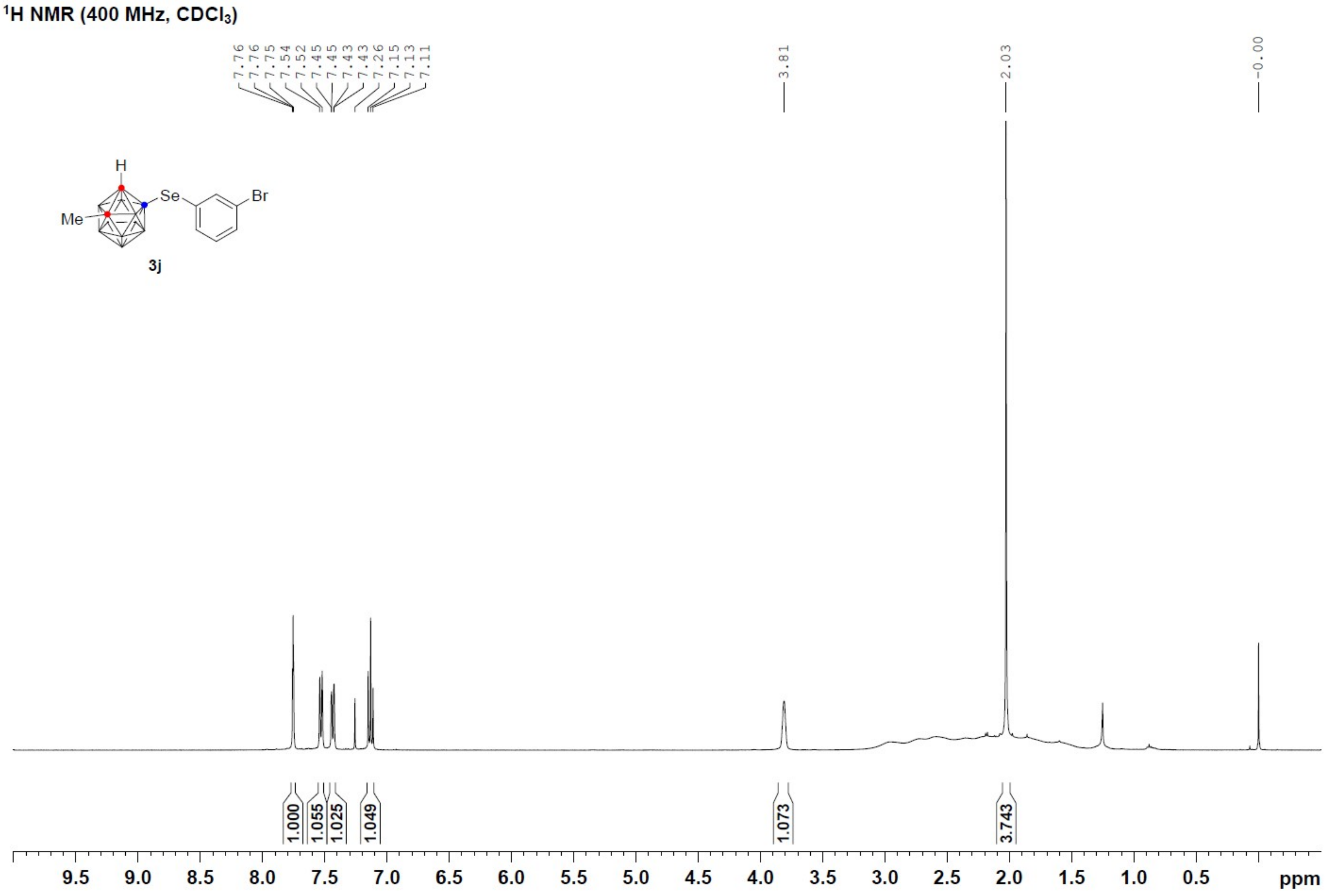

1H NMR (400 MHz, CDCl3)
7.76
7.76
7.75
7.54
7.52
7.45
7.45
7.43
7.43
7.26
7.15
7.13
7.11
3.81
2.03
-0.00
H
Se
Br
Me
3j
1.000
1.055
1.025
1.049
1.073
3.743
9.5
9.0
8.5
8.0
7.5
7.0
6.5
6.0
5.5
5.0
4.5
4.0
3.5
3.0
2.5
2.0
1.5
1.0
0.5
ppm

^{13}C NMR (100 MHz, $CDCl_3$)

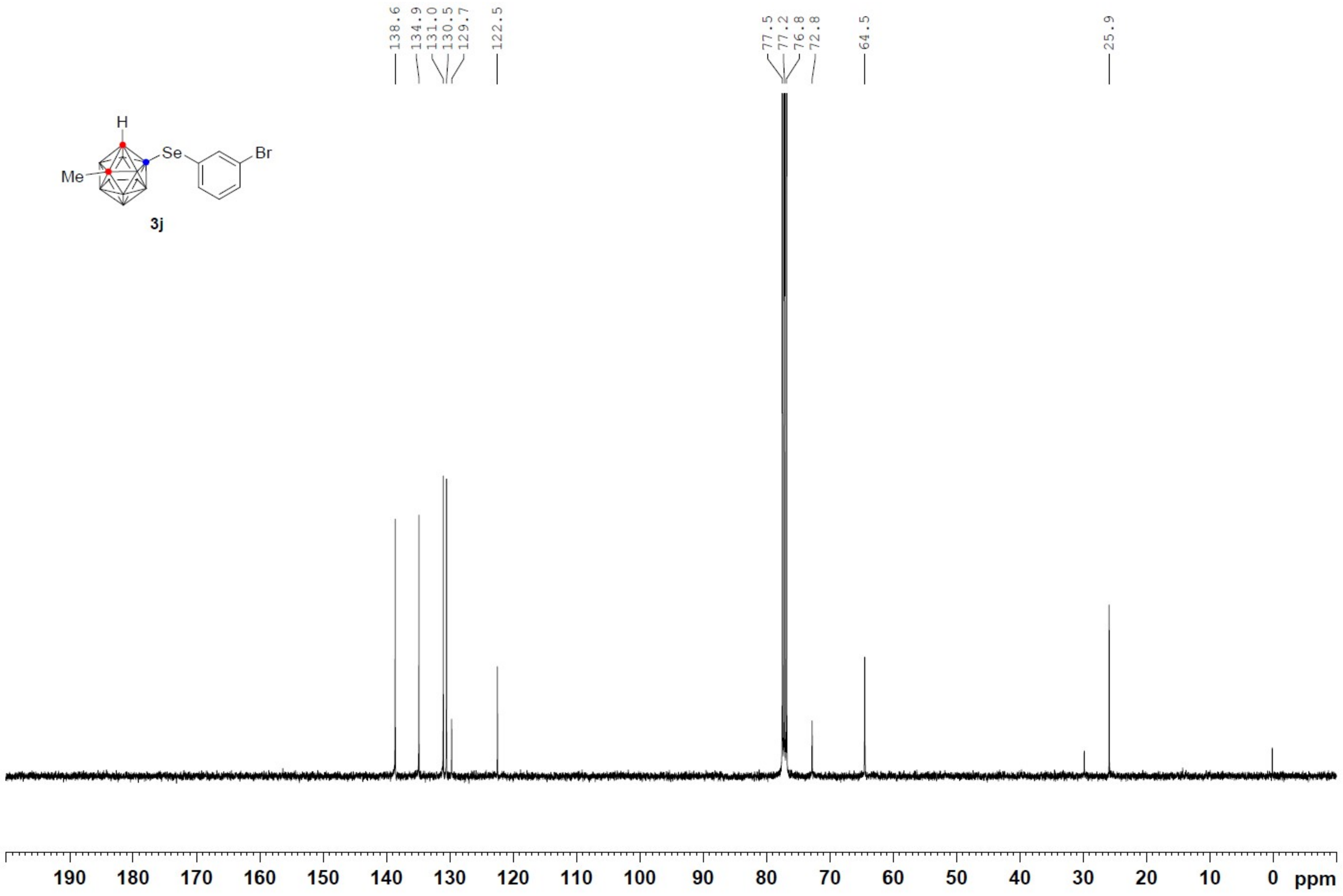

^{11}B NMR (128 MHz, $CDCl_3$)

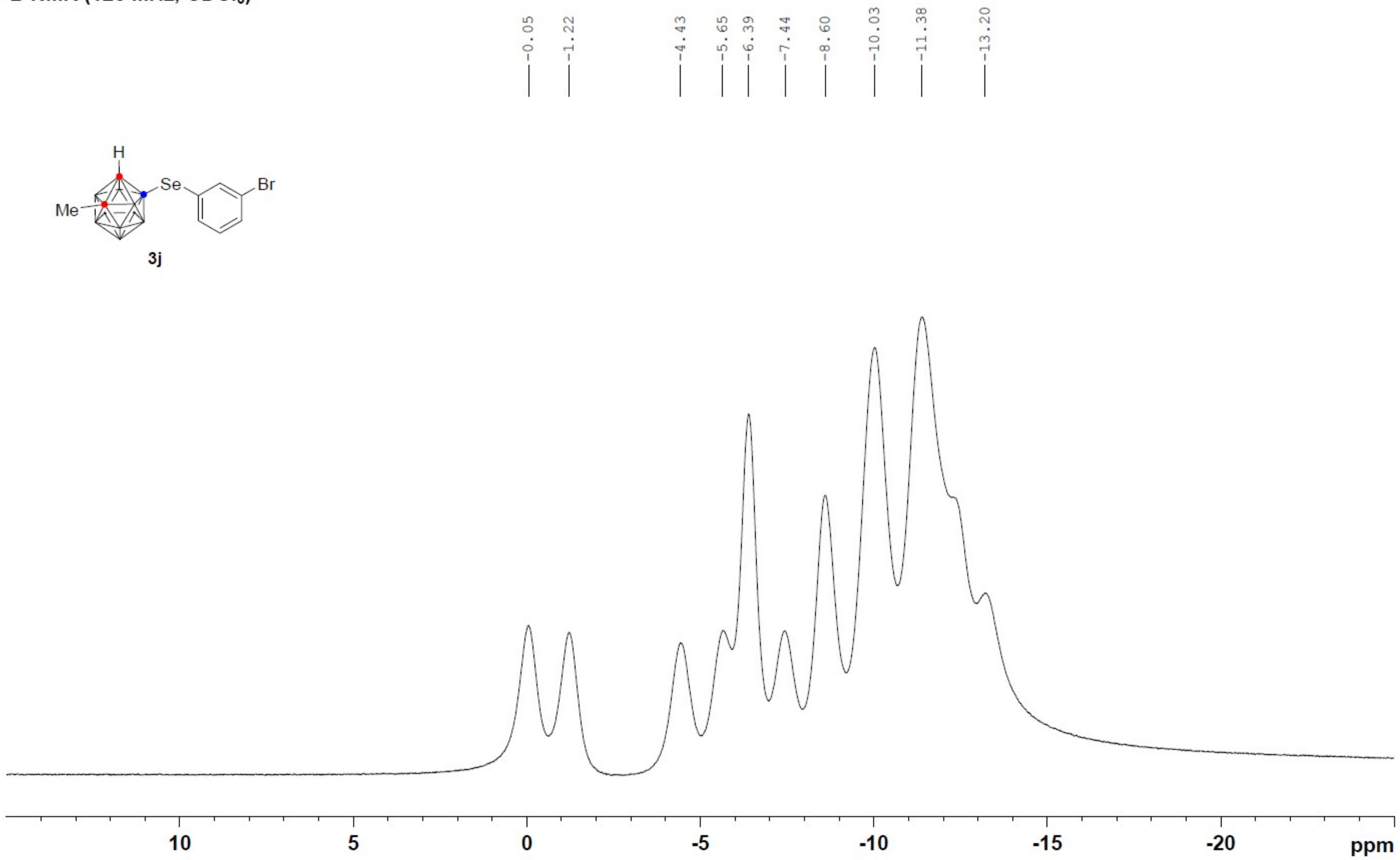

$^{11}B\{^1H\}$ NMR (128 MHz, $CDCl_3$)

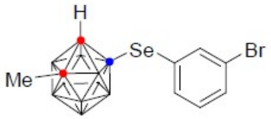

3j

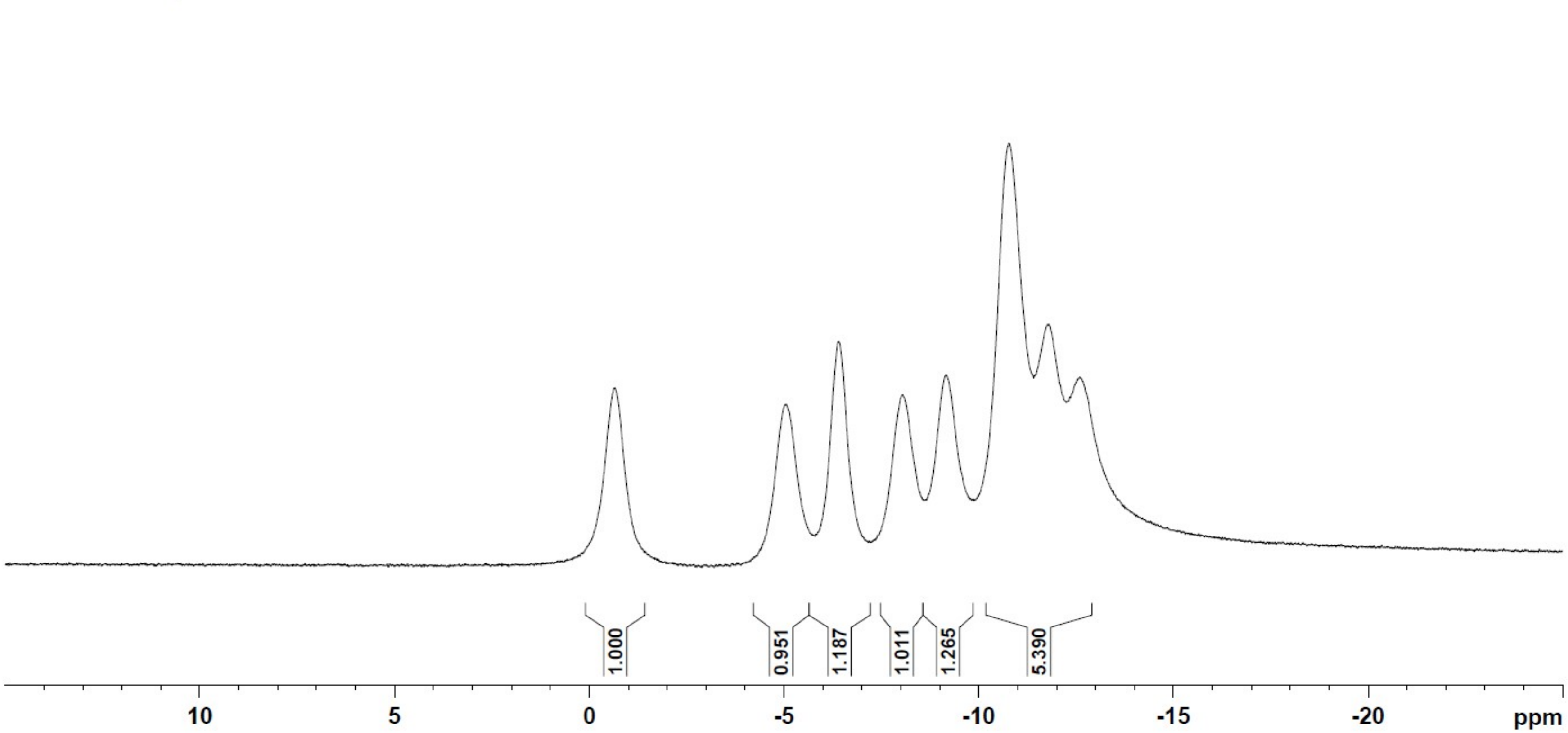

^1H NMR (400 MHz, $CDCl_3$)

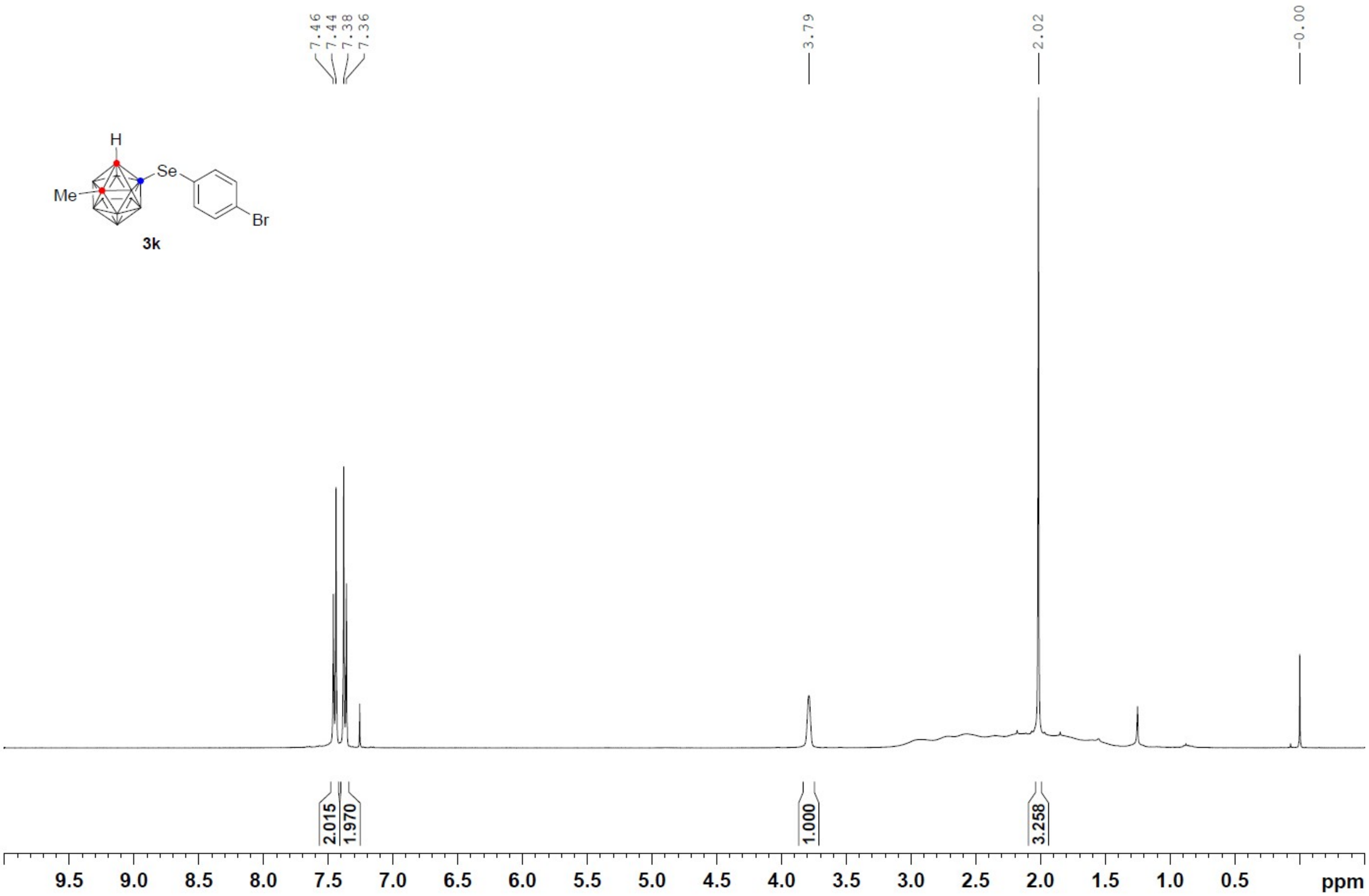

^{13}C NMR (100 MHz, $CDCl_3$)

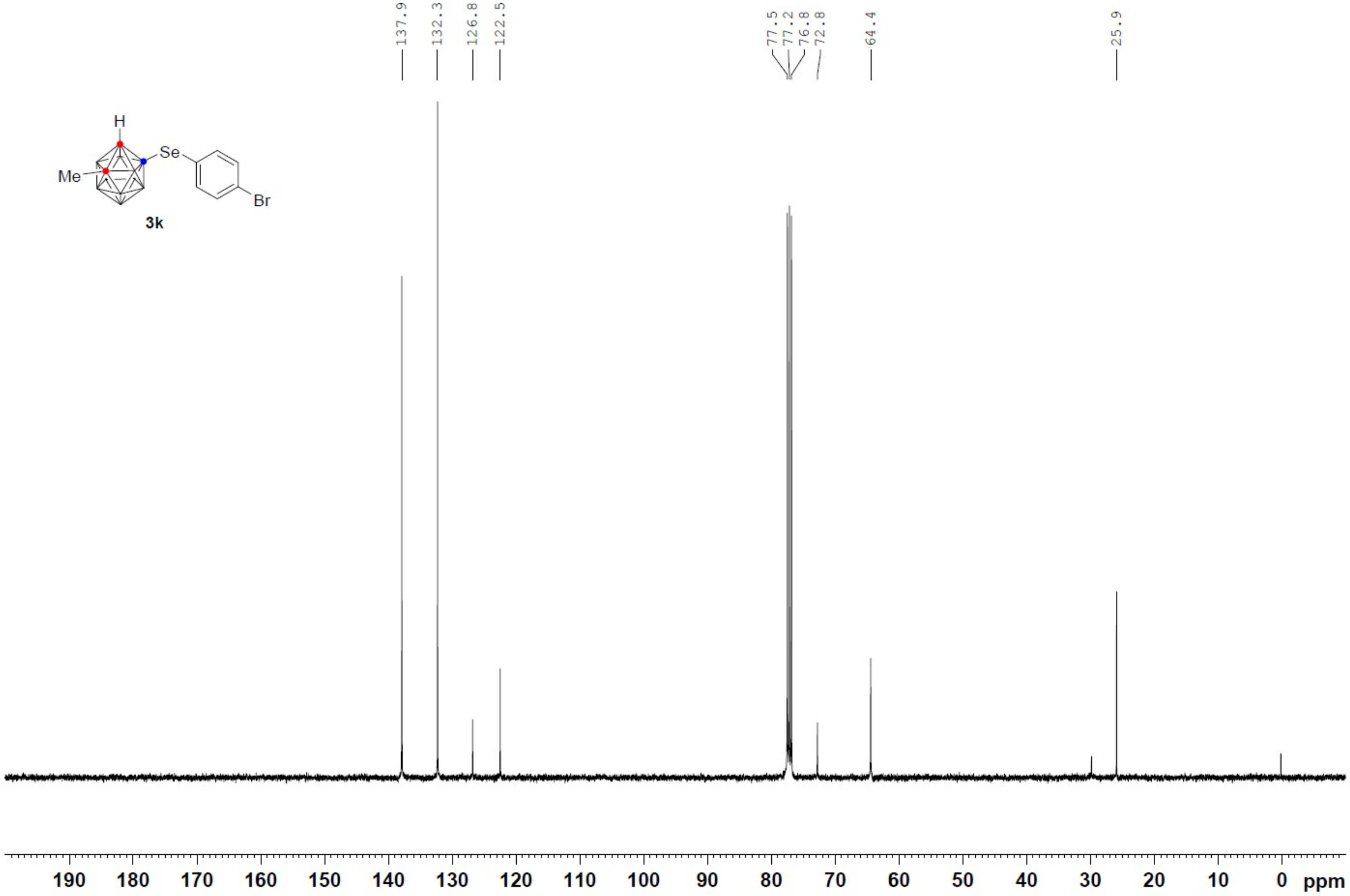

^{11}B NMR (128 MHz, $CDCl_3$)

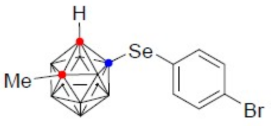

3k

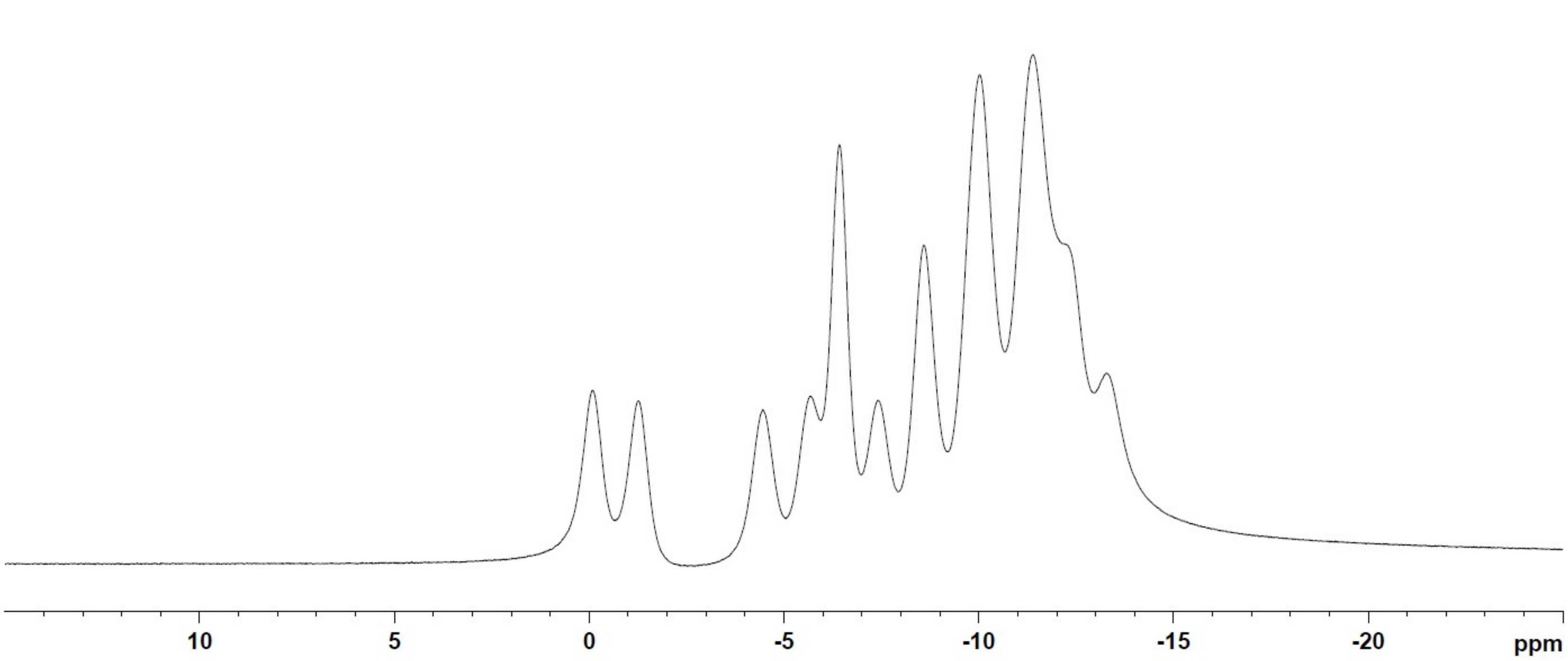

$^{11}B\{^1H\}$ NMR (128 MHz, $CDCl_3$)

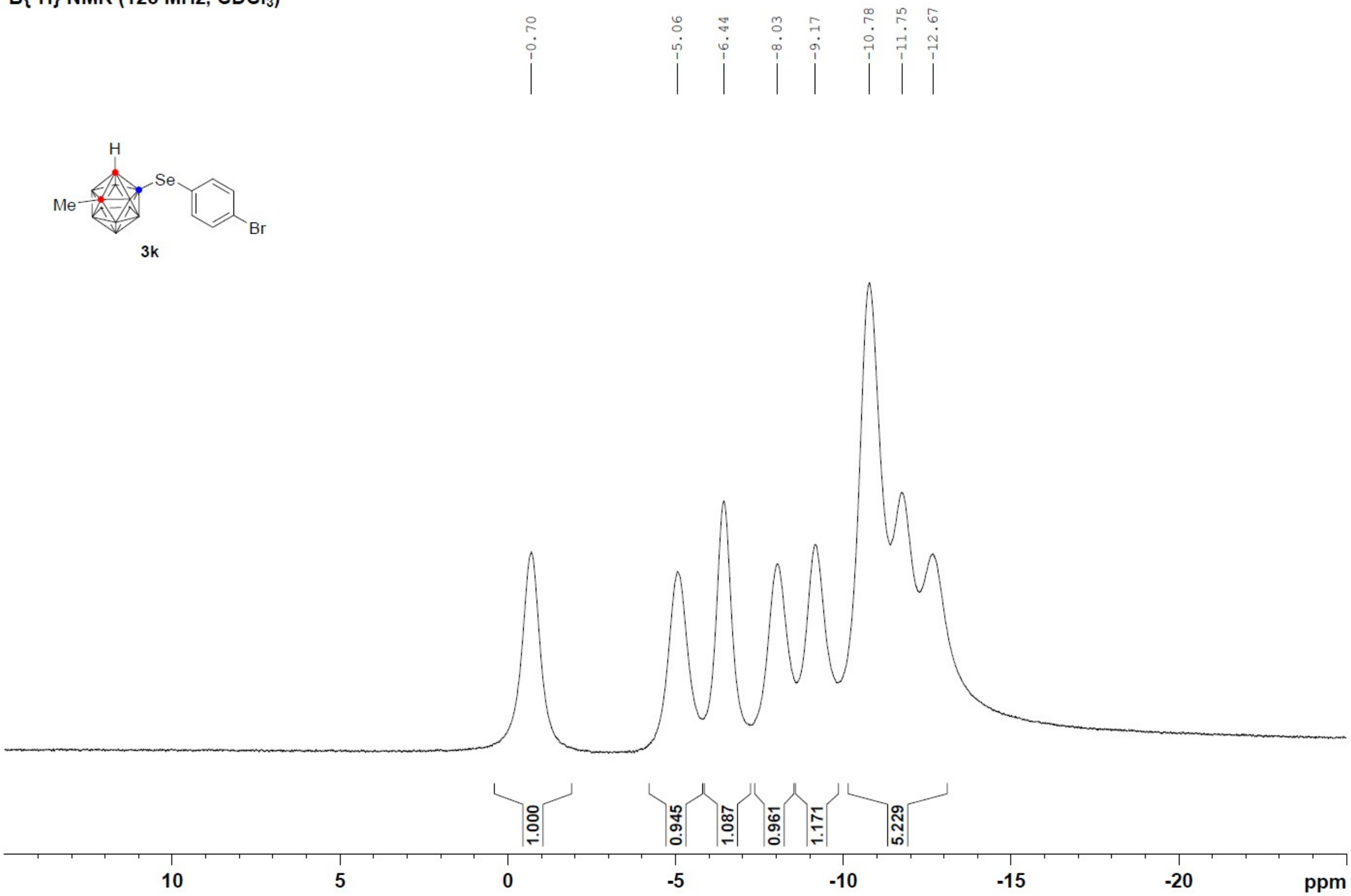

3k

^{1}H NMR (400 MHz, $CDCl_3$)

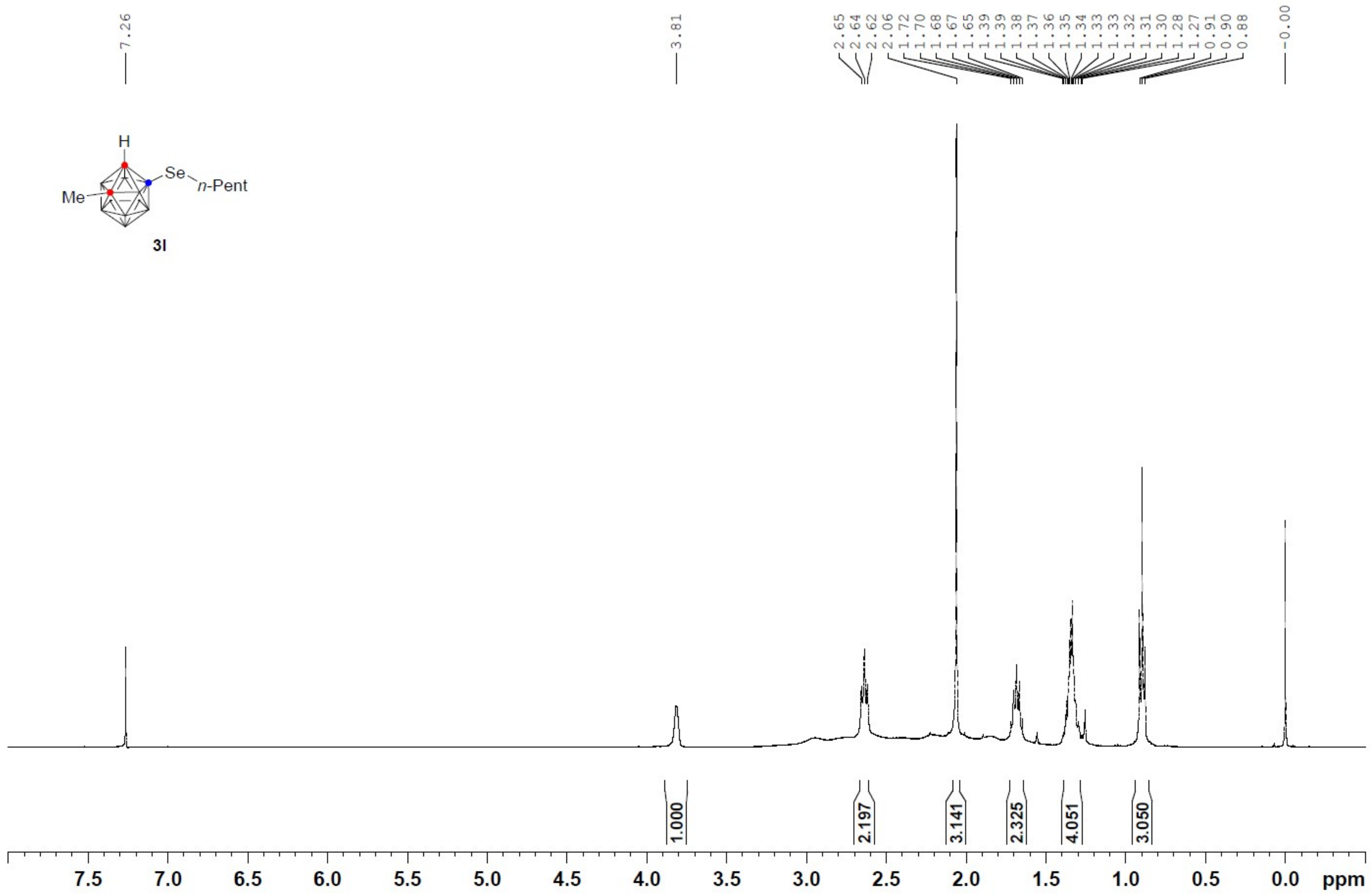

3l

$^{13}C\{^1H\}$ NMR (100 MHz, $CDCl_3$)

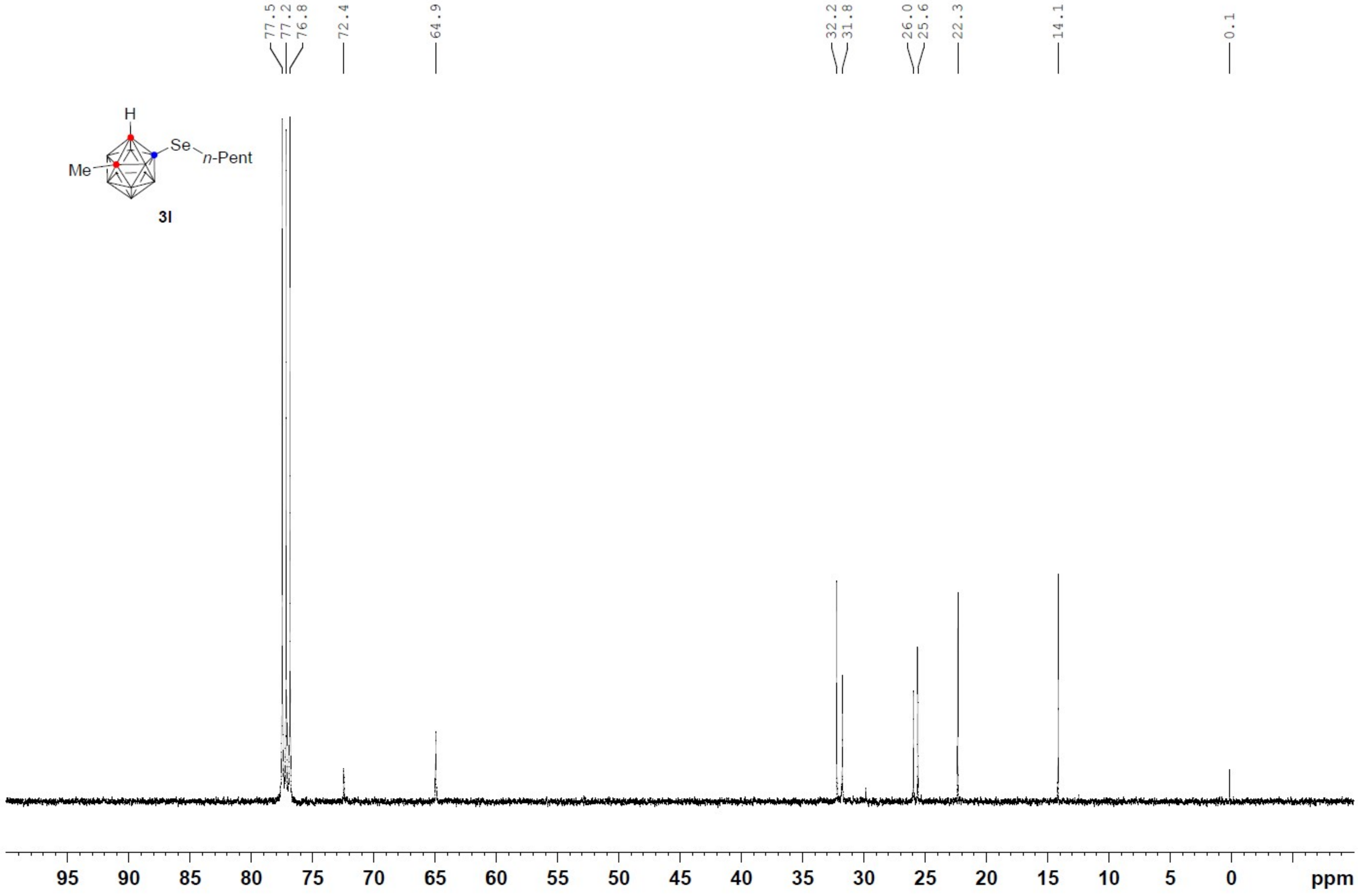

^{11}B NMR (128 MHz, $CDCl_3$)

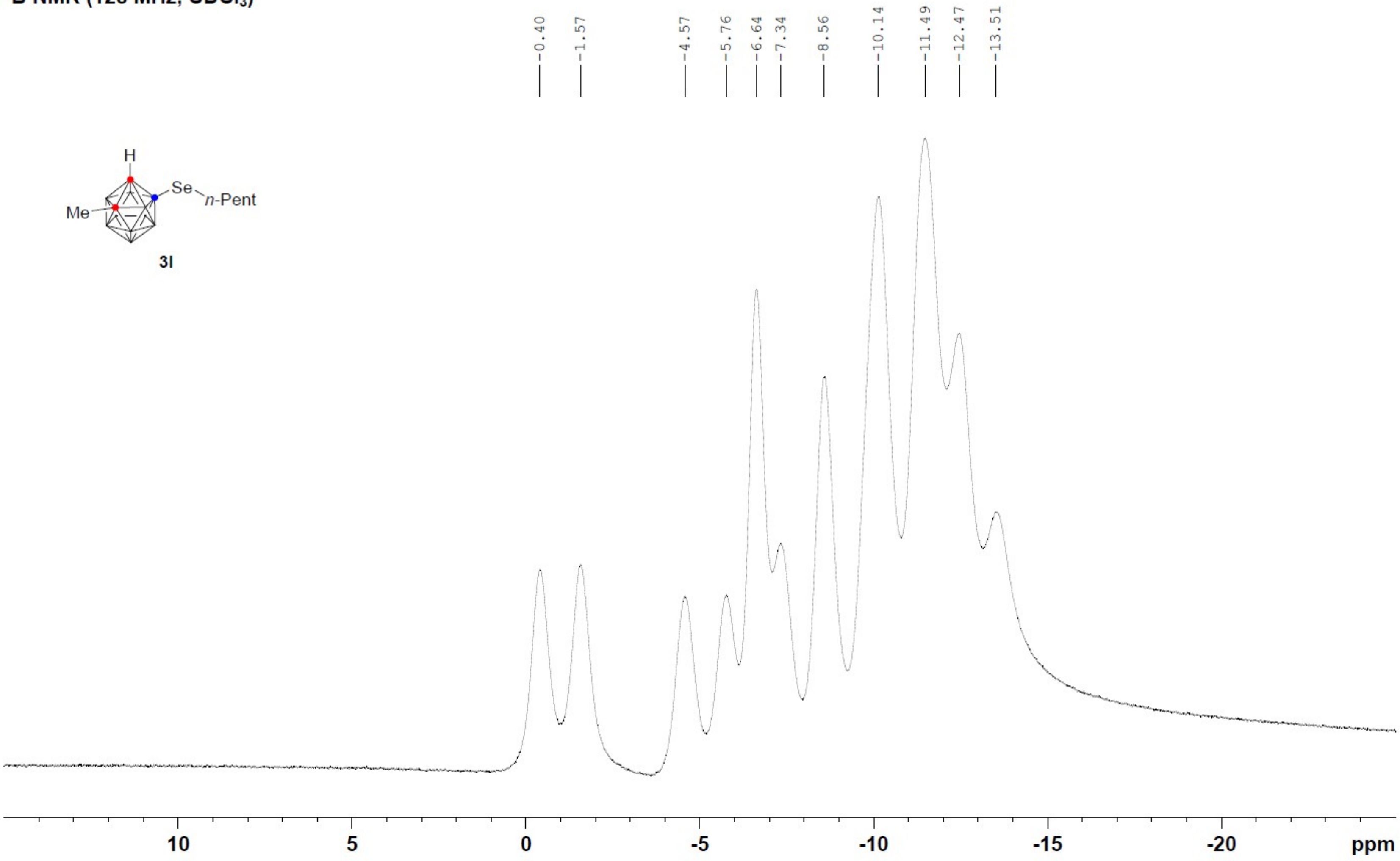

$^{11}B\{^1H\}$ NMR (128 MHz, $CDCl_3$)

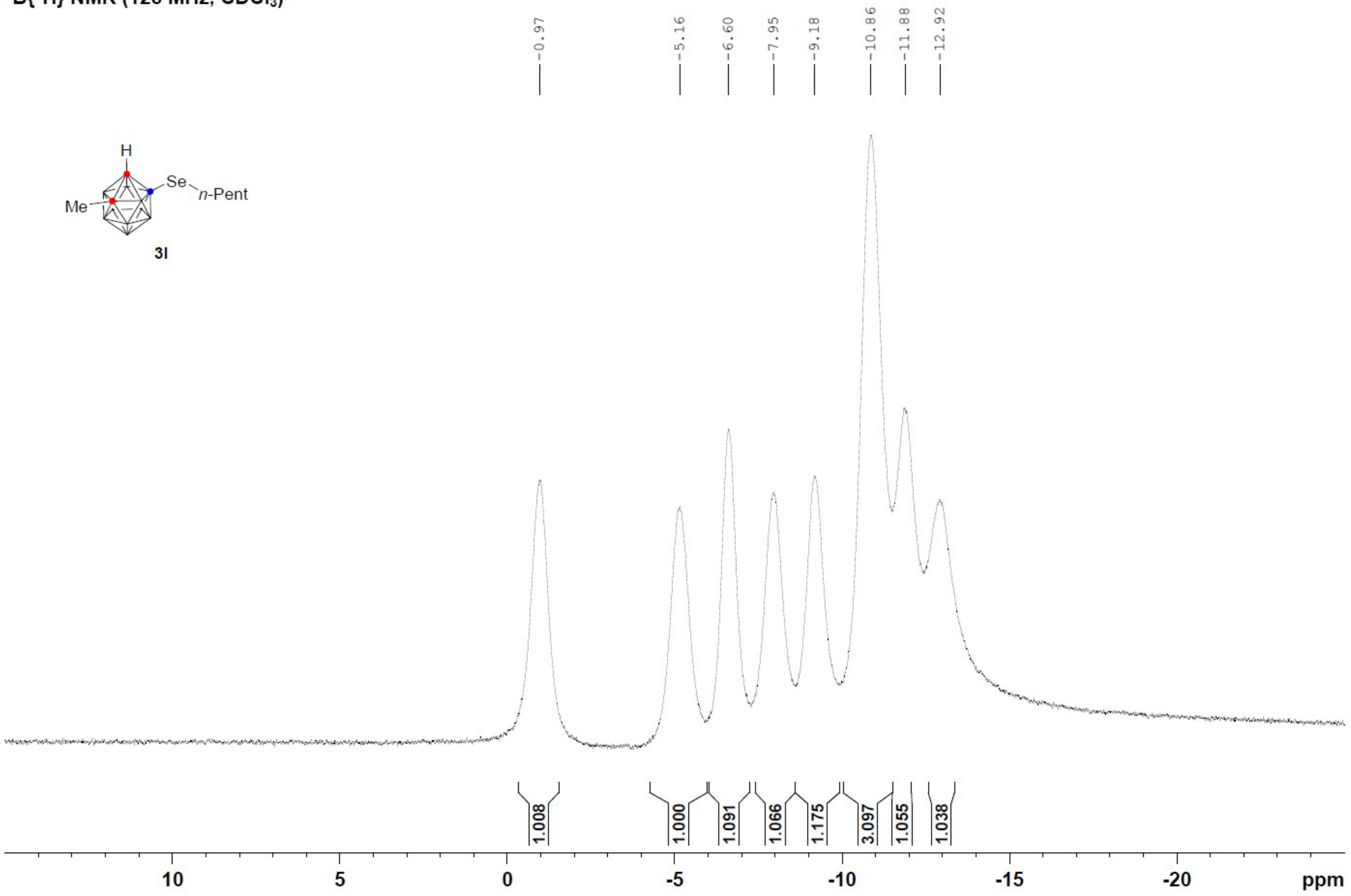

^{1}H NMR (400 MHz, $CDCl_3$)

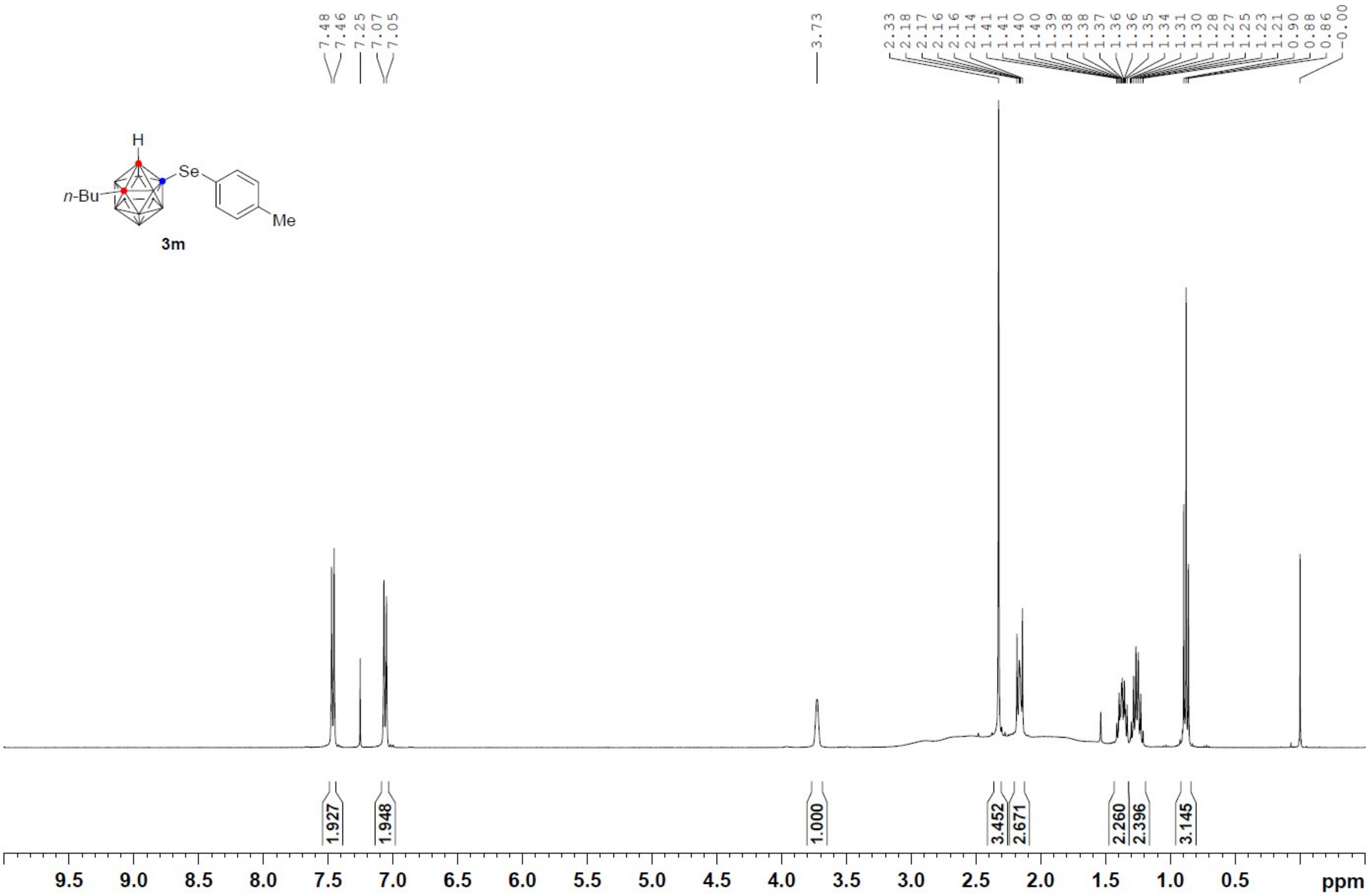

^{13}C NMR (100 MHz, $CDCl_3$)

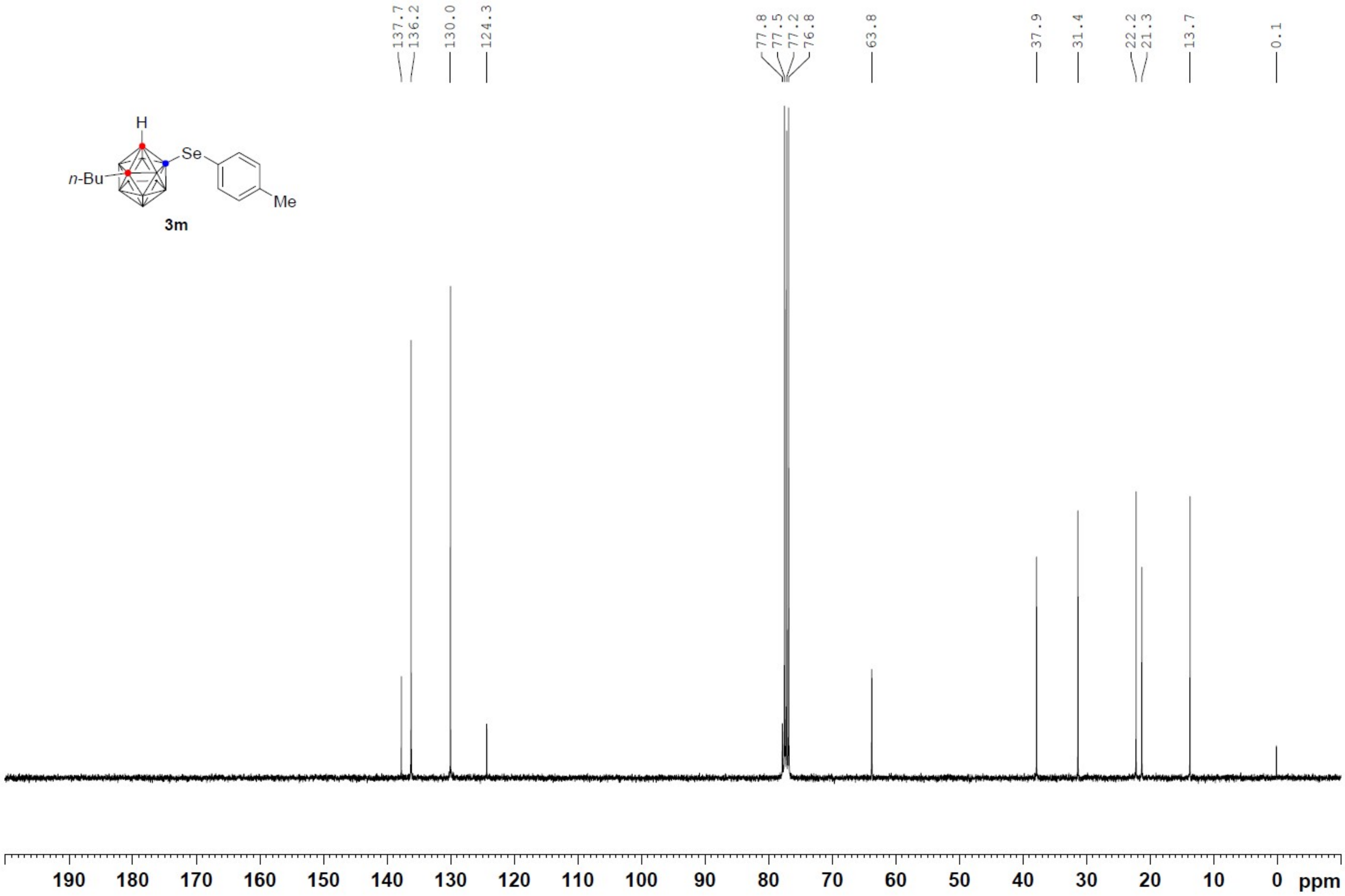

^{11}B NMR (128 MHz, $CDCl_3$)

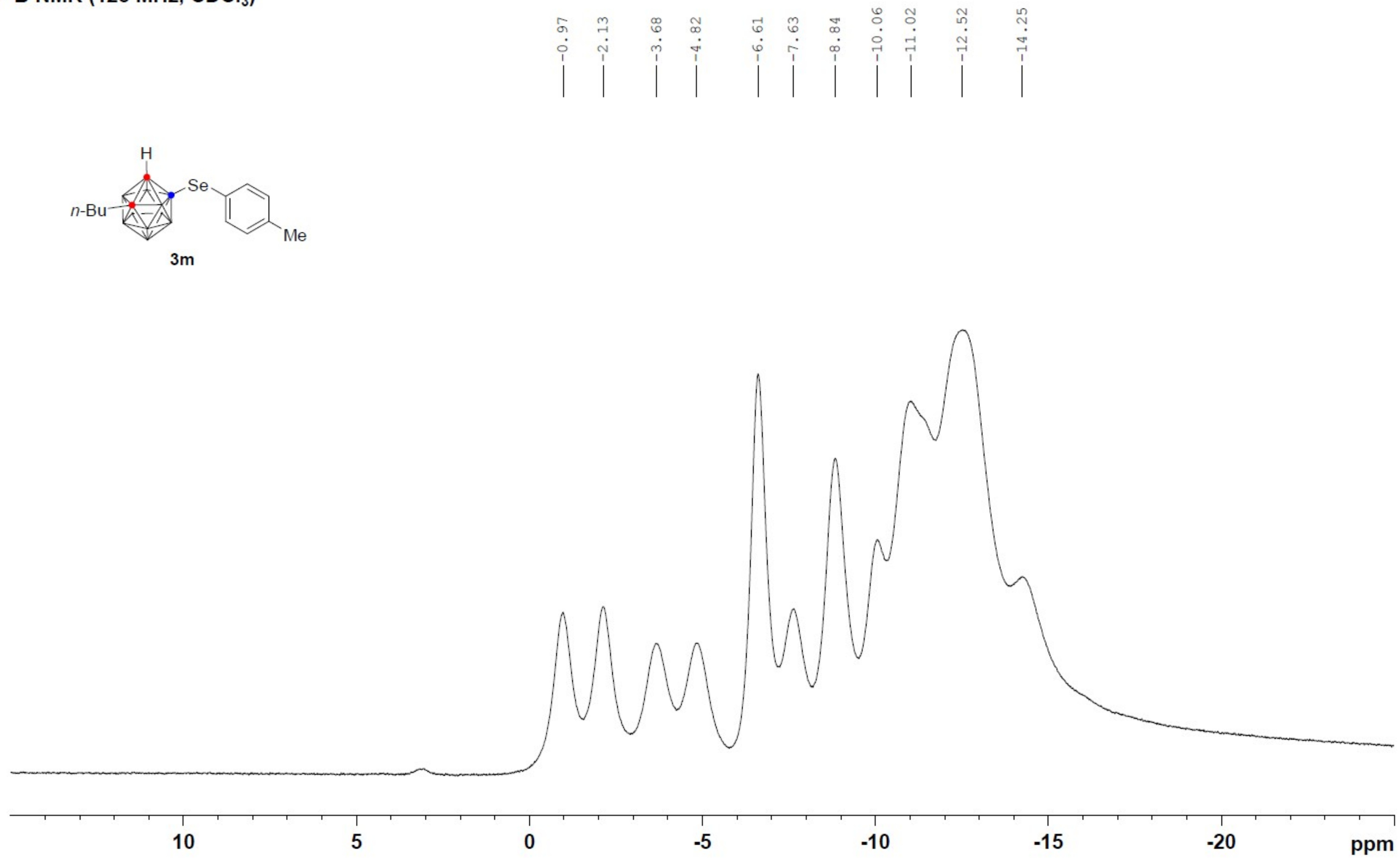

$^{11}B\{^1H\}$ NMR (128 MHz, $CDCl_3$)

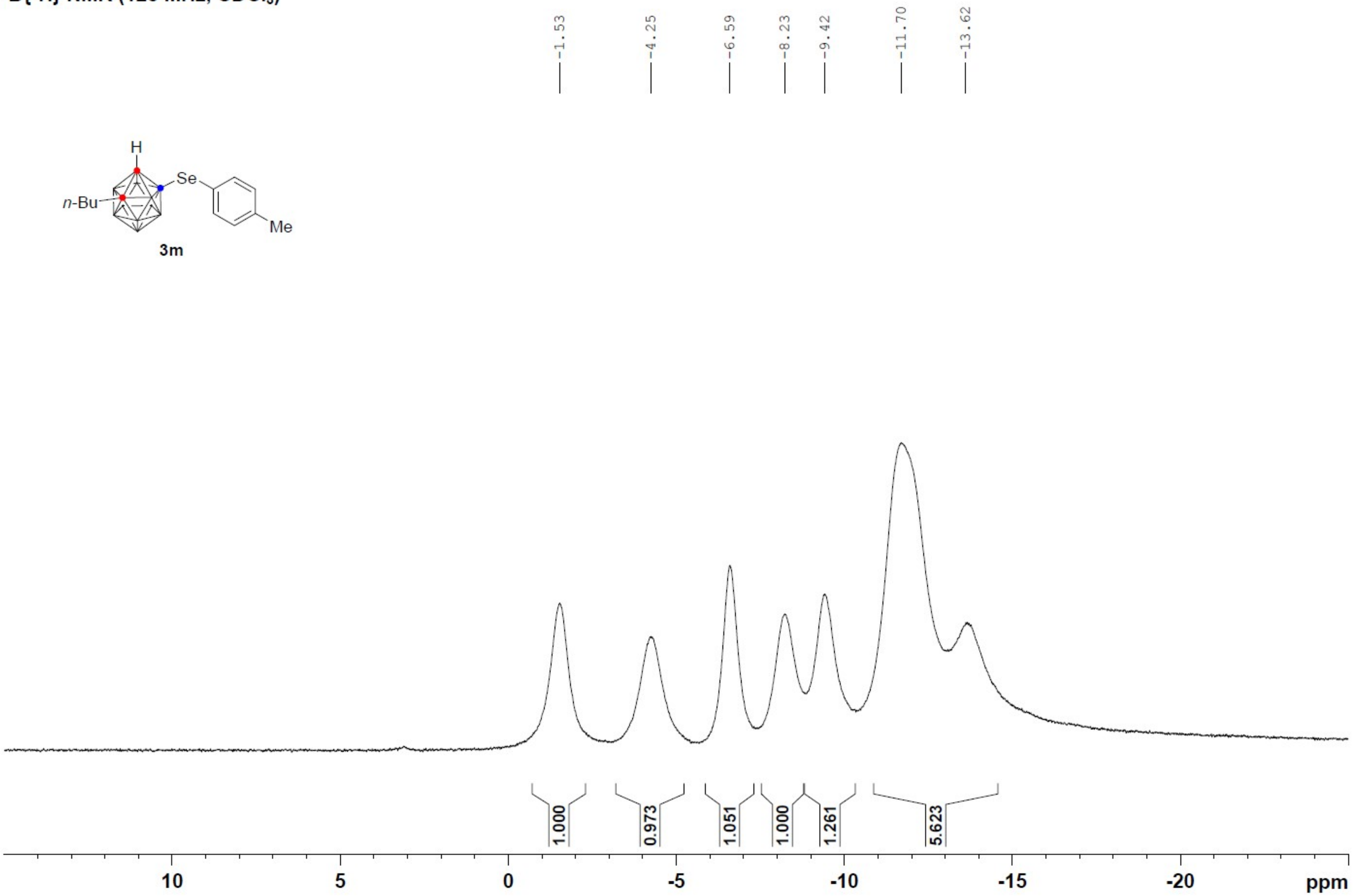

^1H NMR (400 MHz, $CDCl_3$)

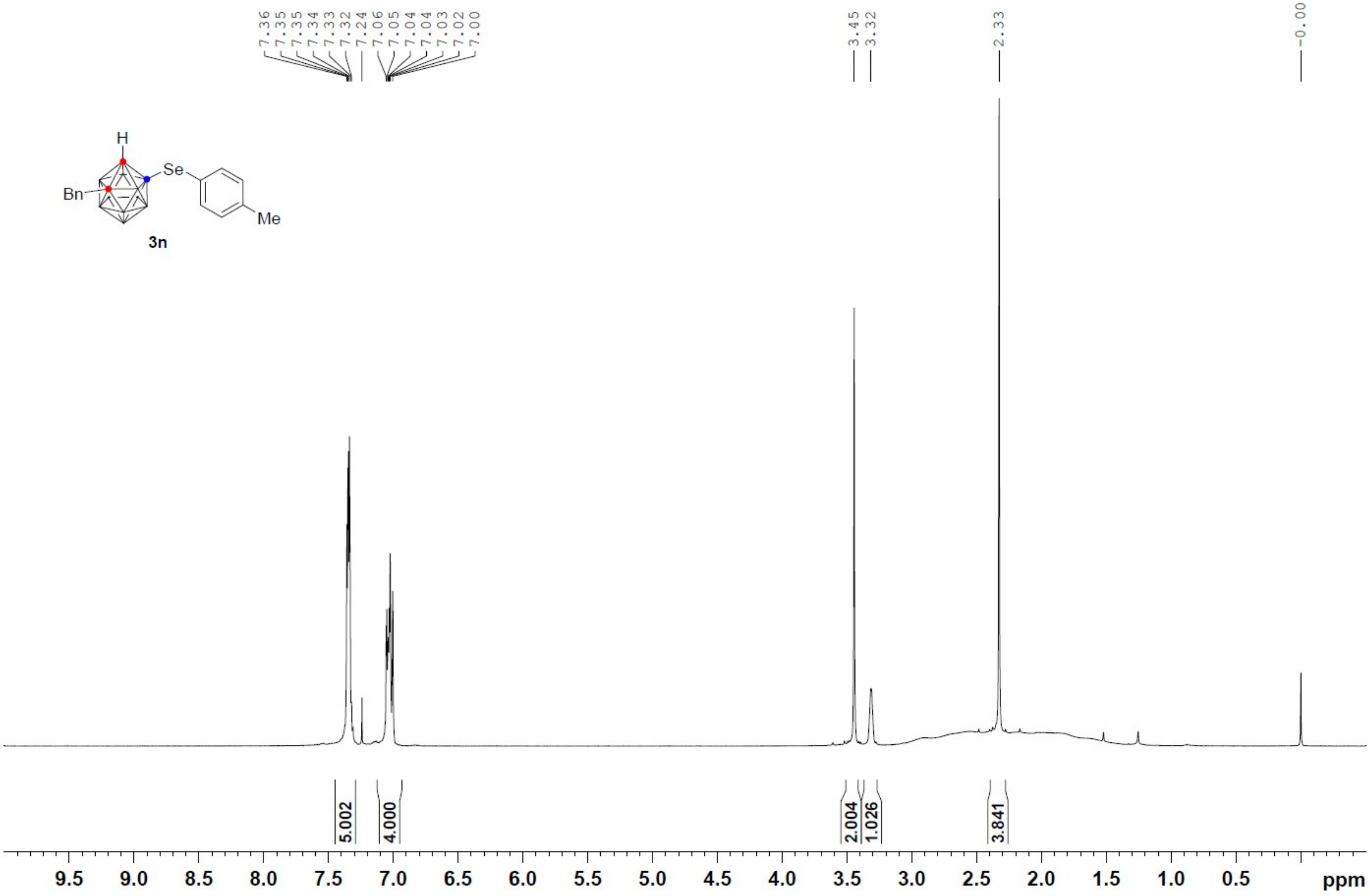

$^{13}C\{^{1}H\}$ NMR (100 MHz, $CDCl_3$)

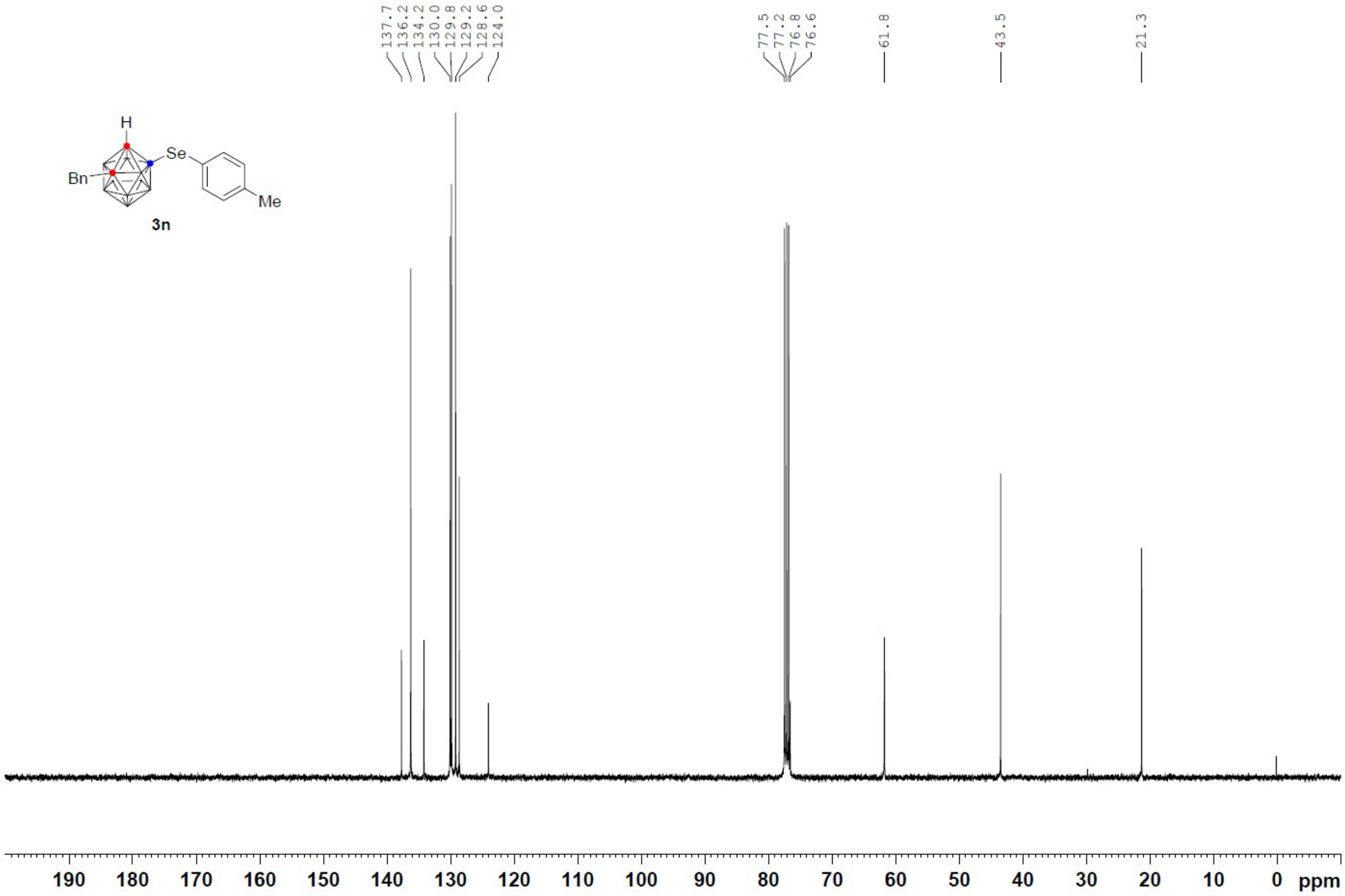

^{11}B NMR (128 MHz, $CDCl_3$)

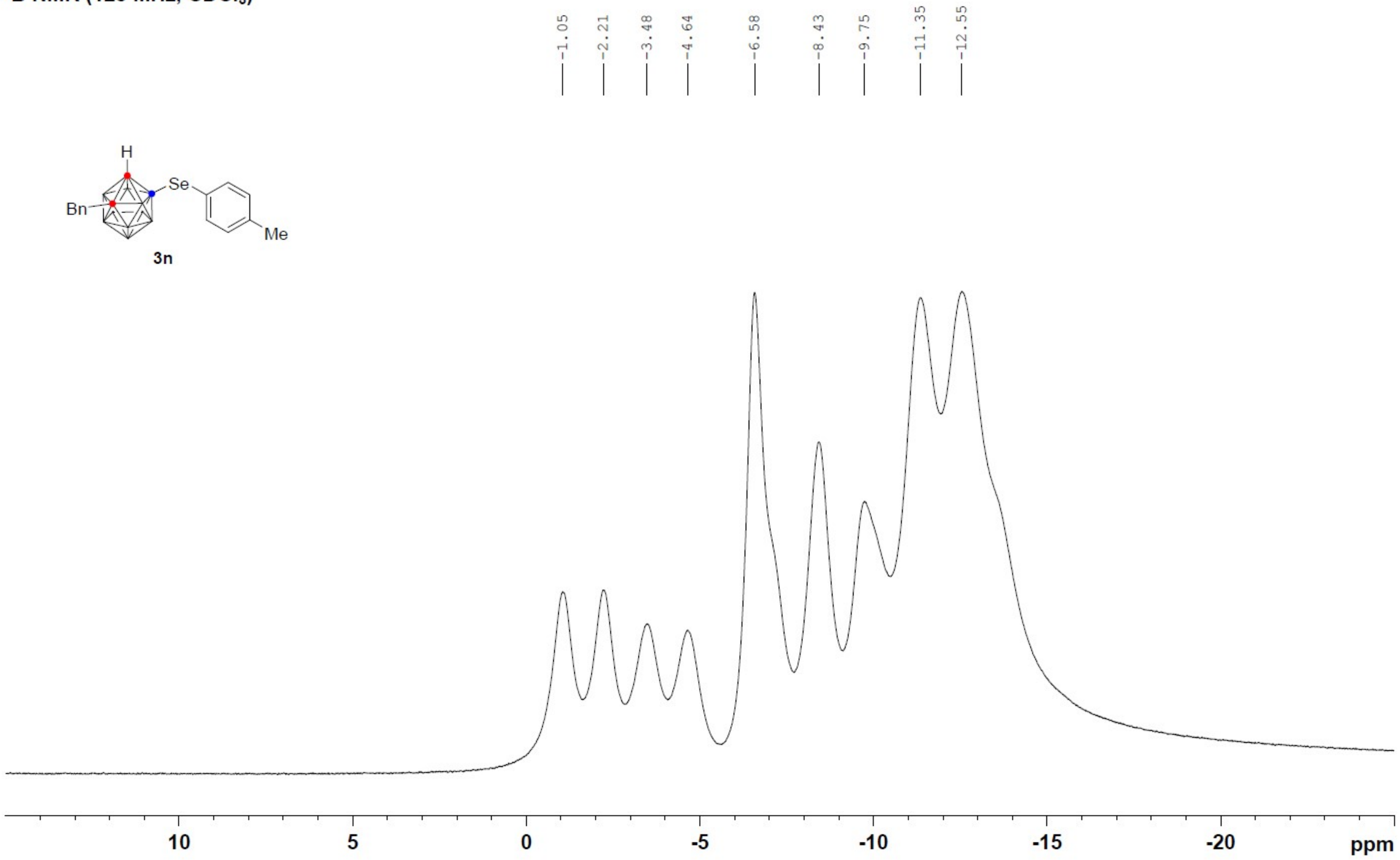

$^{11}B\{^1H\}$ NMR (128 MHz, $CDCl_3$)

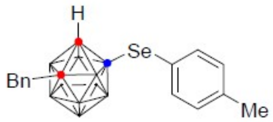

3n

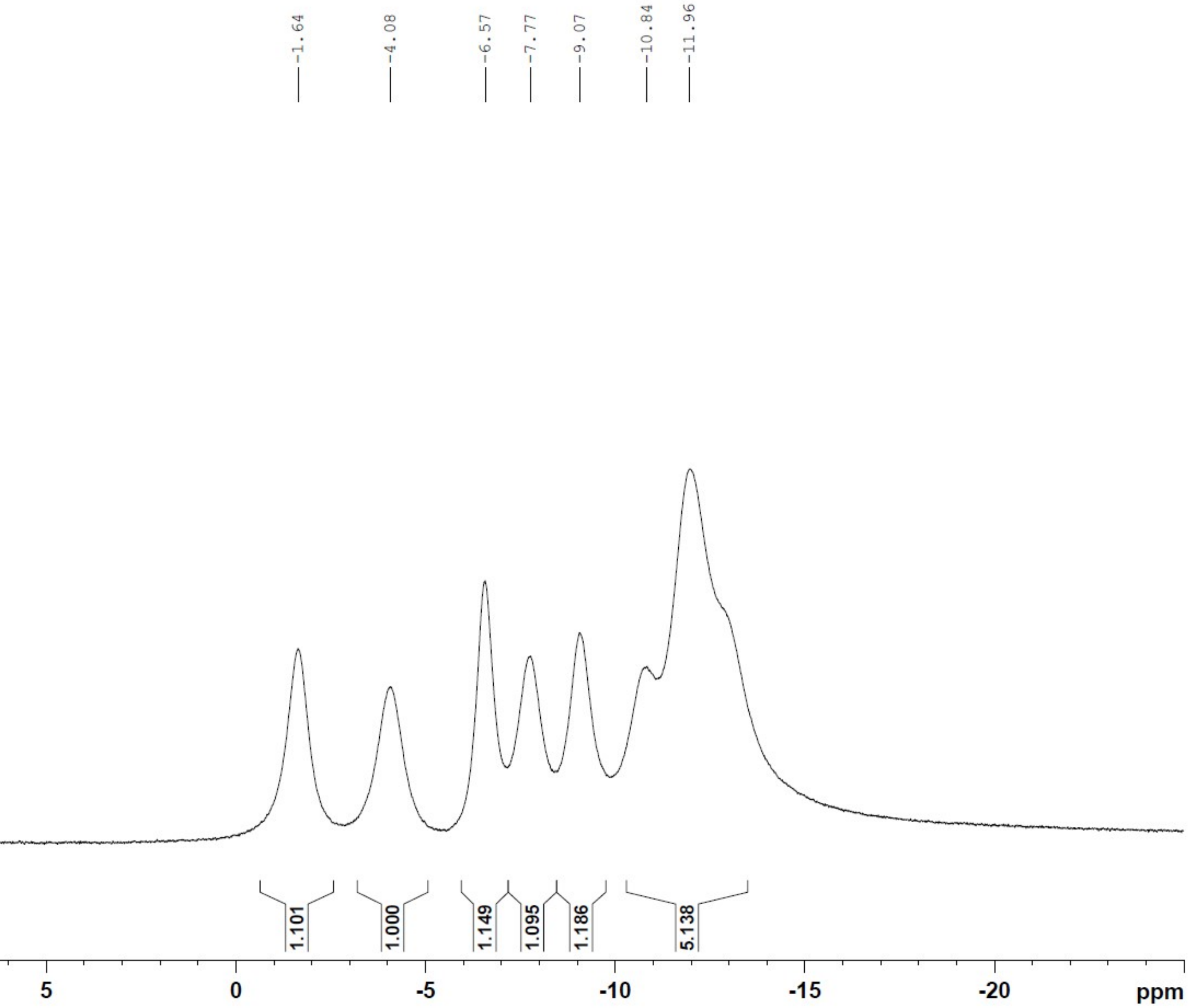

^1H NMR (400 MHz, $CDCl_3$)

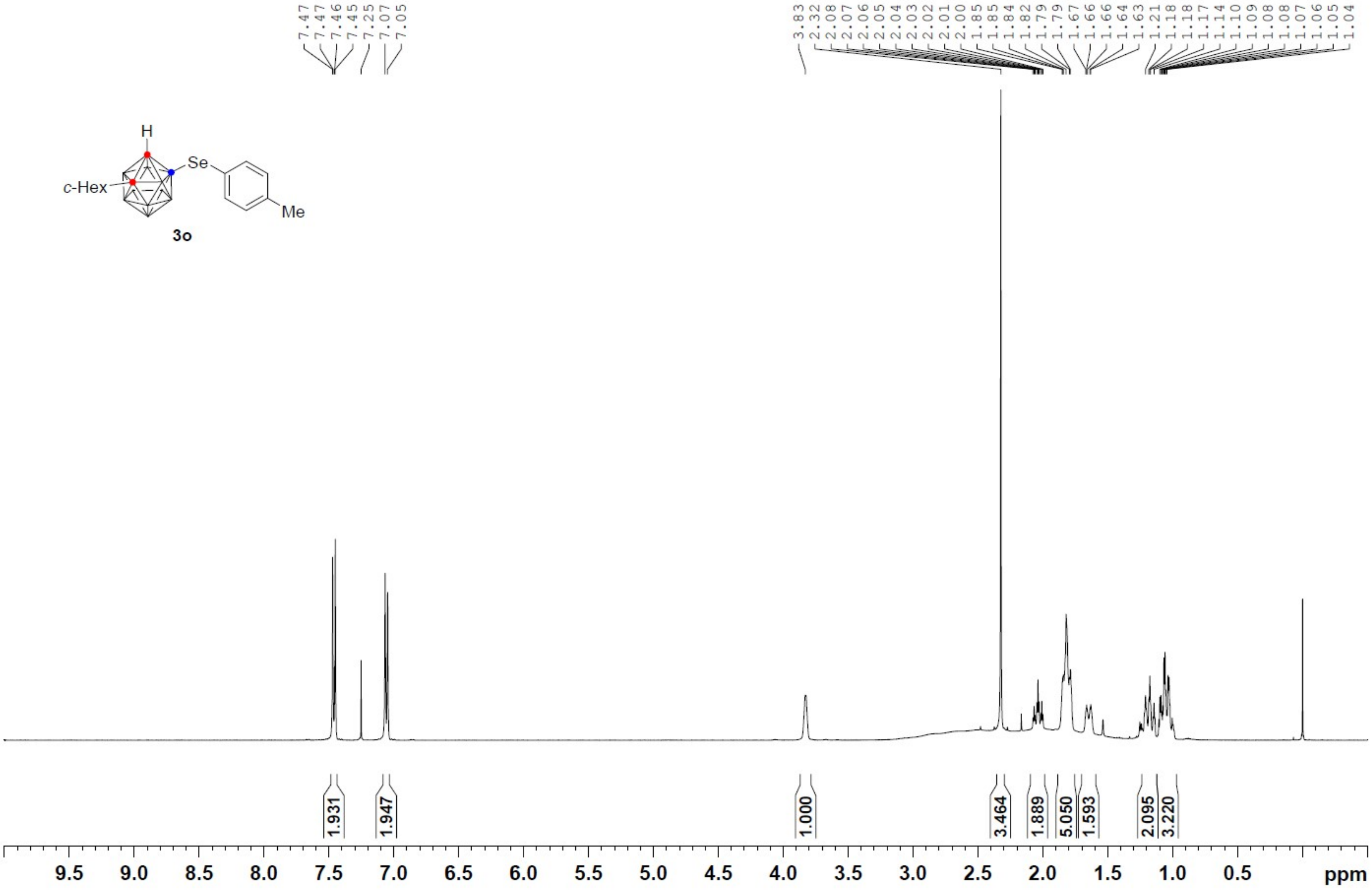

3o

^{13}C NMR (100 MHz, $CDCl_3$)

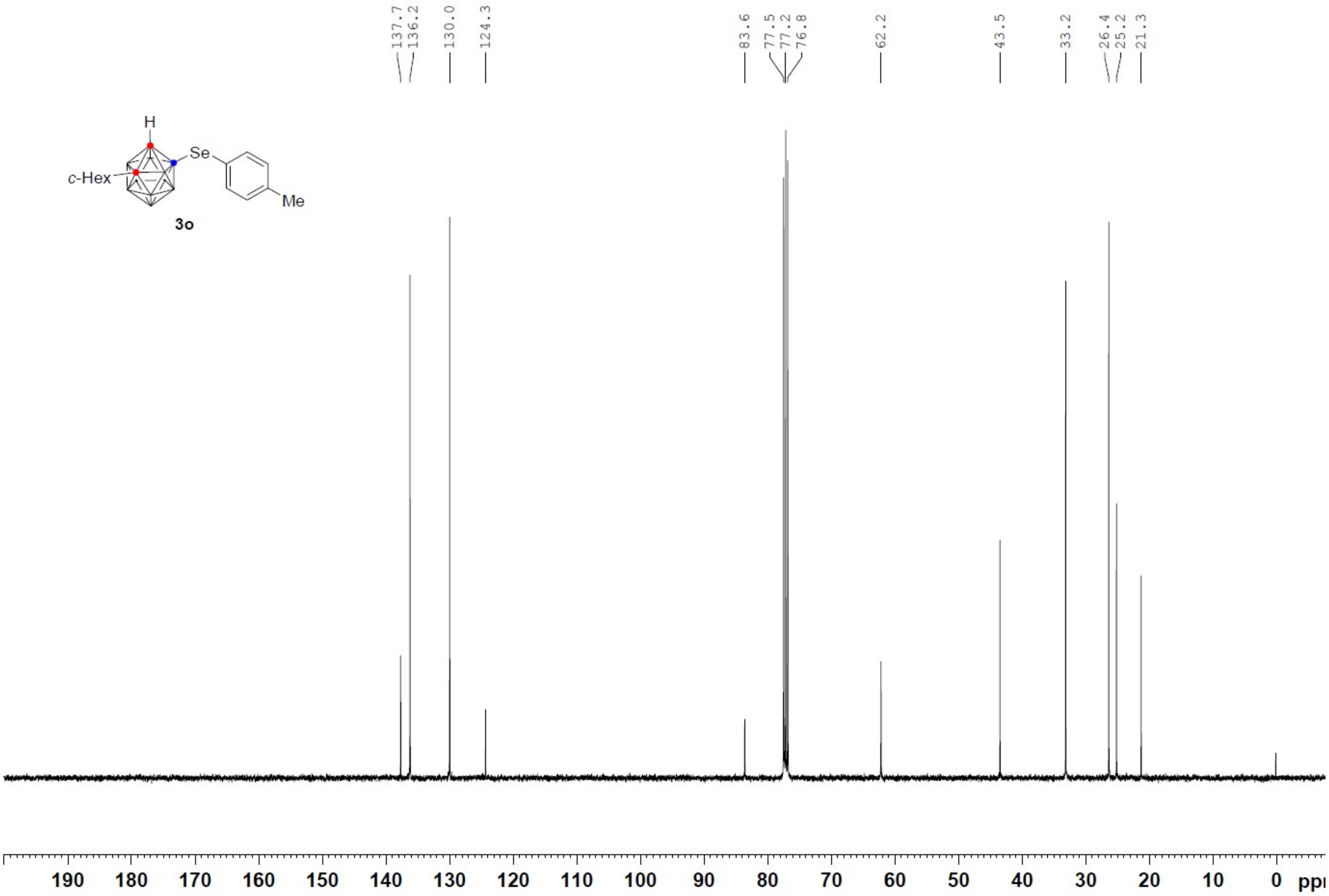

^{11}B NMR (128 MHz, $CDCl_3$)

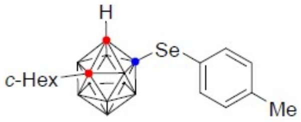

3o

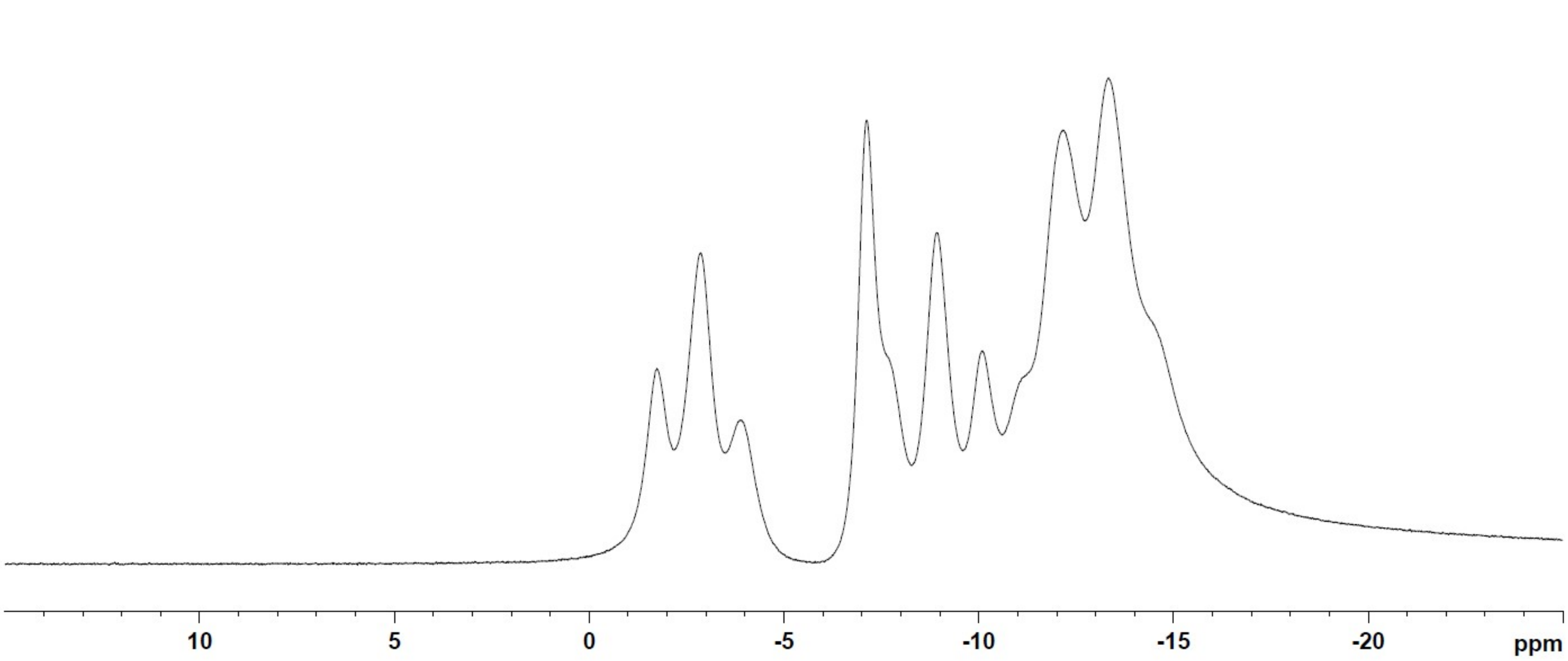

$^{11}B\{^{1}H\}$ NMR (128 MHz, $CDCl_3$)

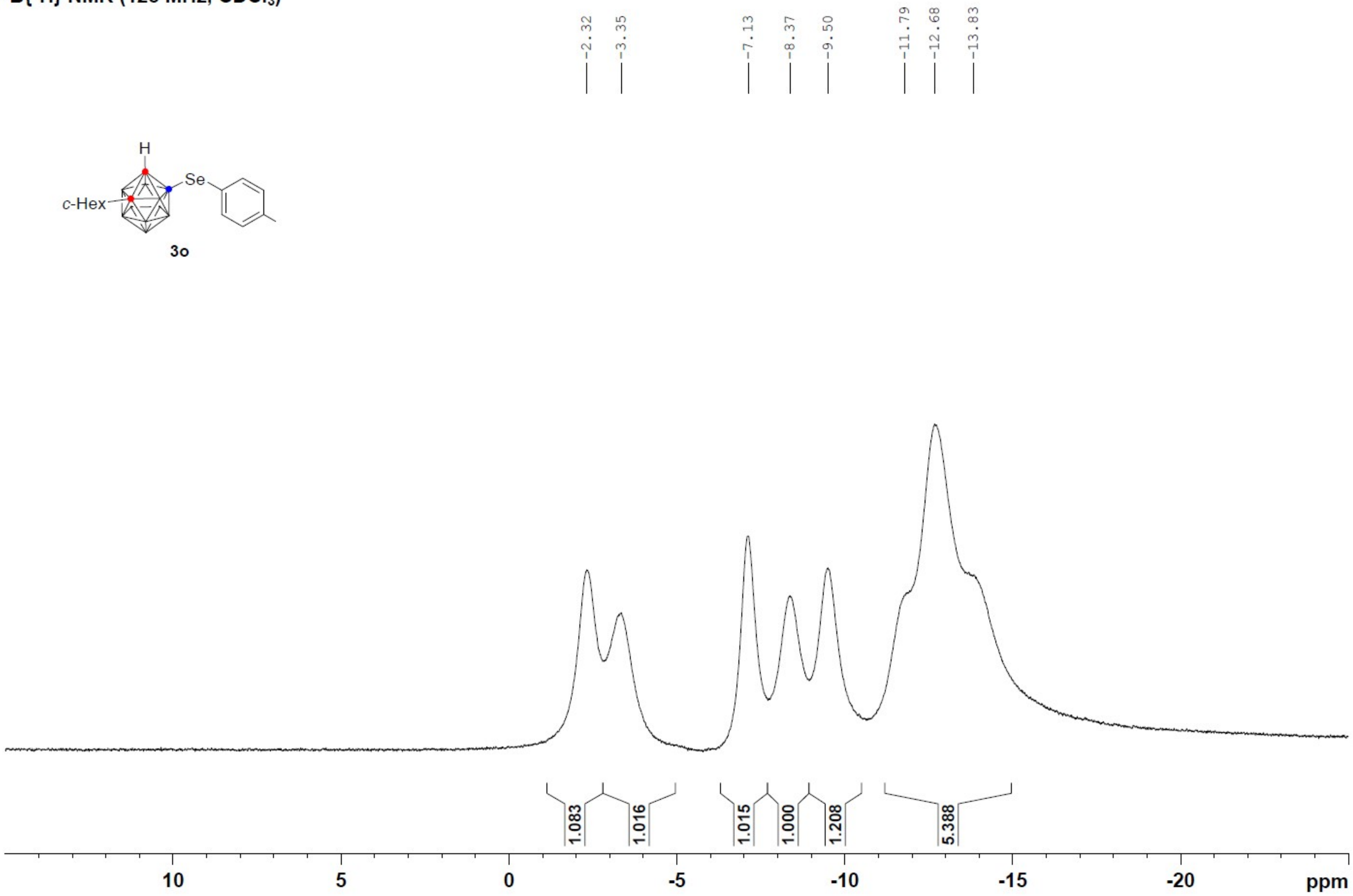

^{1}H NMR (400 MHz, $CDCl_3$)

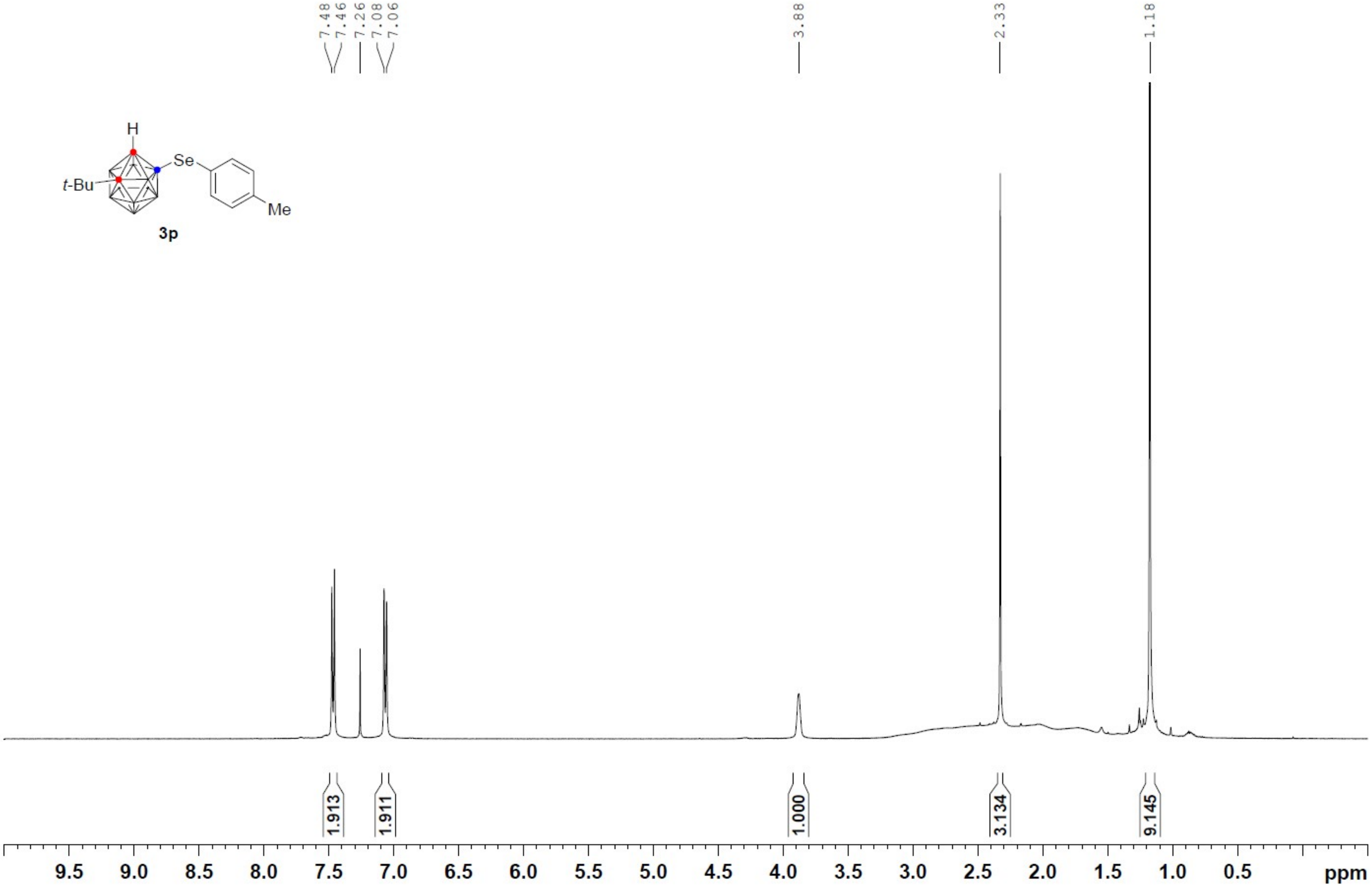

$^{13}C\{^{1}H\}$ NMR (100 MHz, $CDCl_3$)

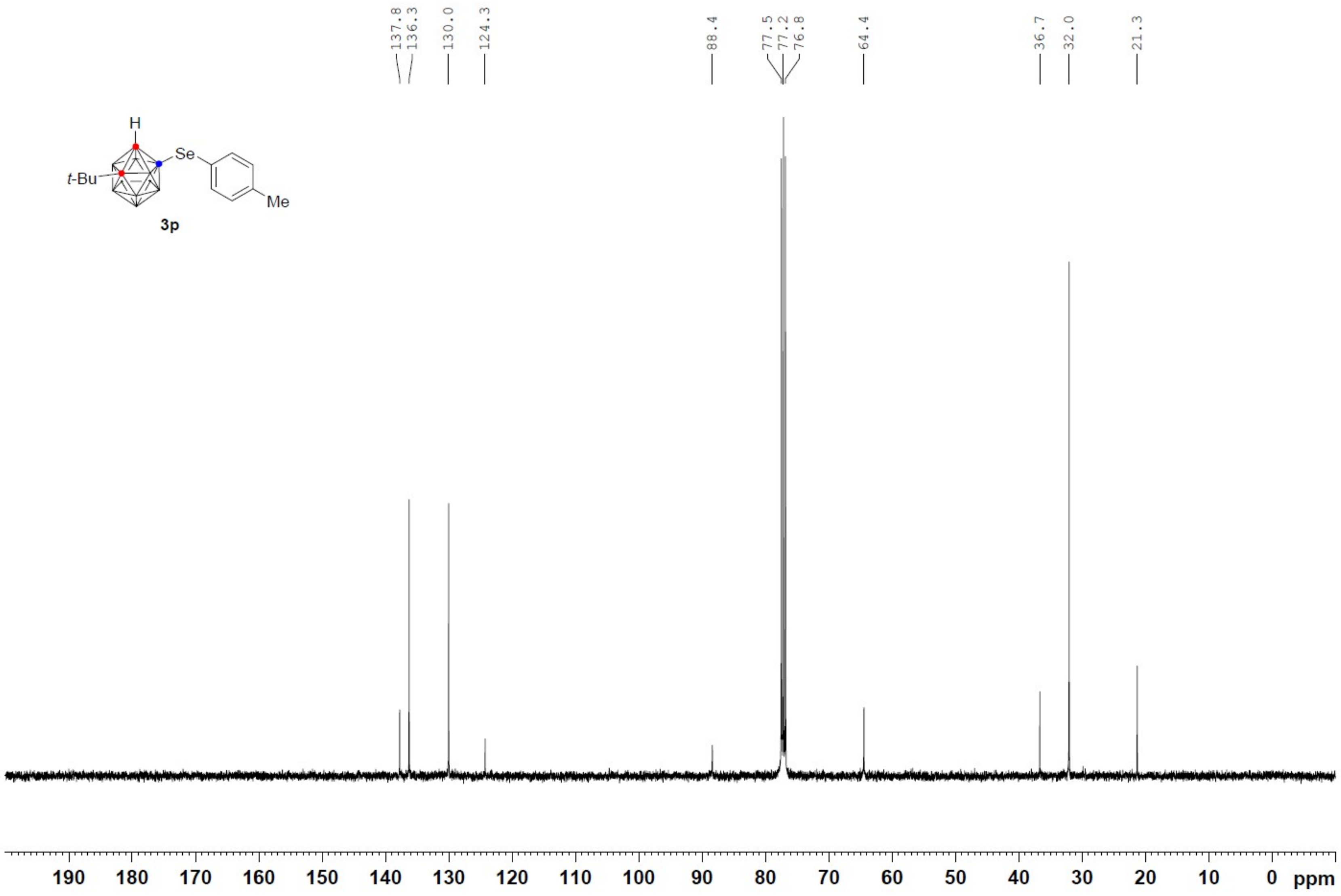

^{11}B NMR (128 MHz, $CDCl_3$)

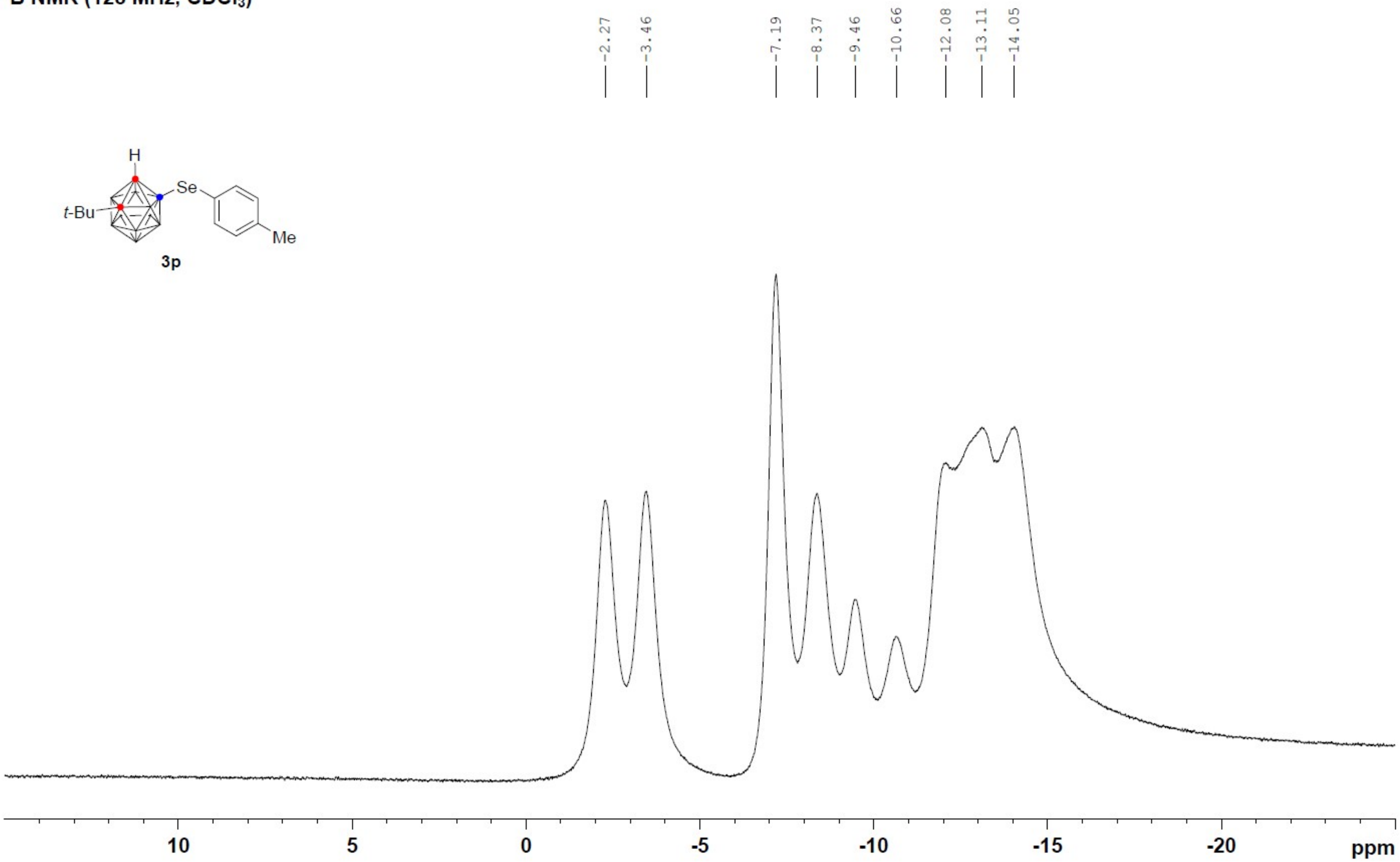

$^{11}B\{^1H\}$ NMR (128 MHz, $CDCl_3$)

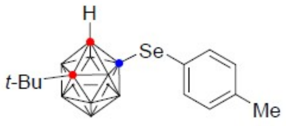

3p

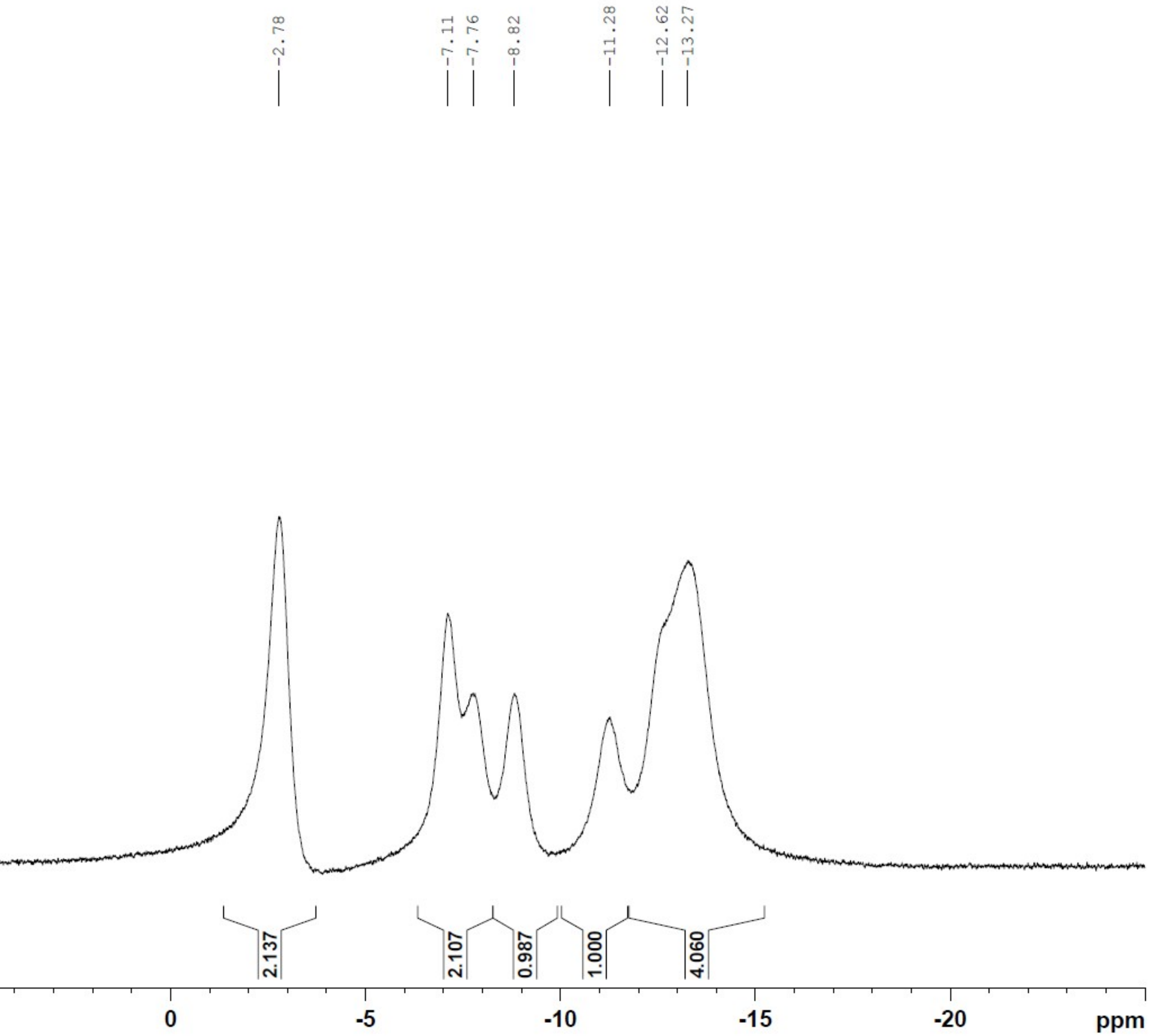

^1H NMR (400 MHz, $CDCl_3$)

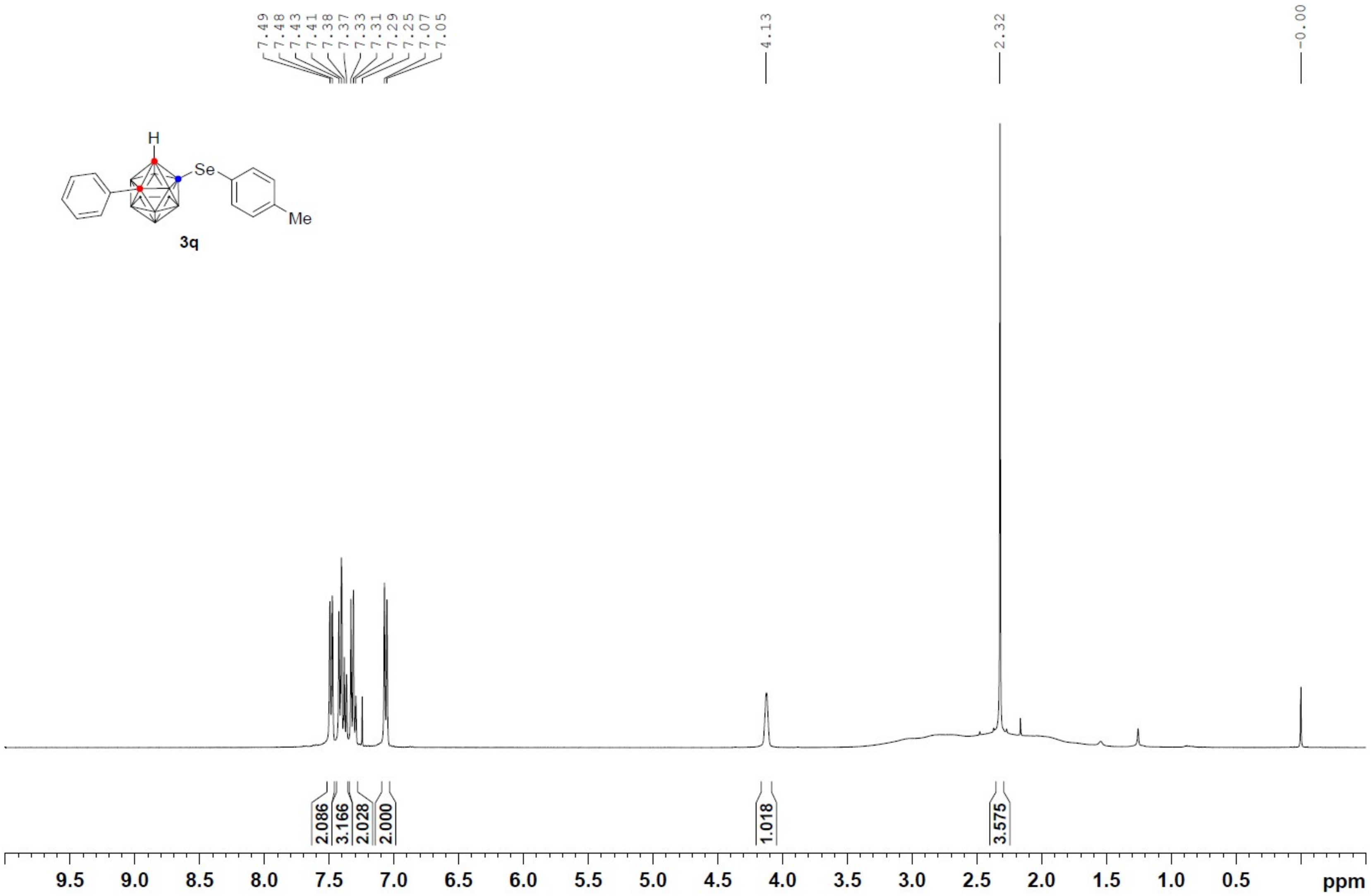

^{13}C NMR (100 MHz, $CDCl_3$)

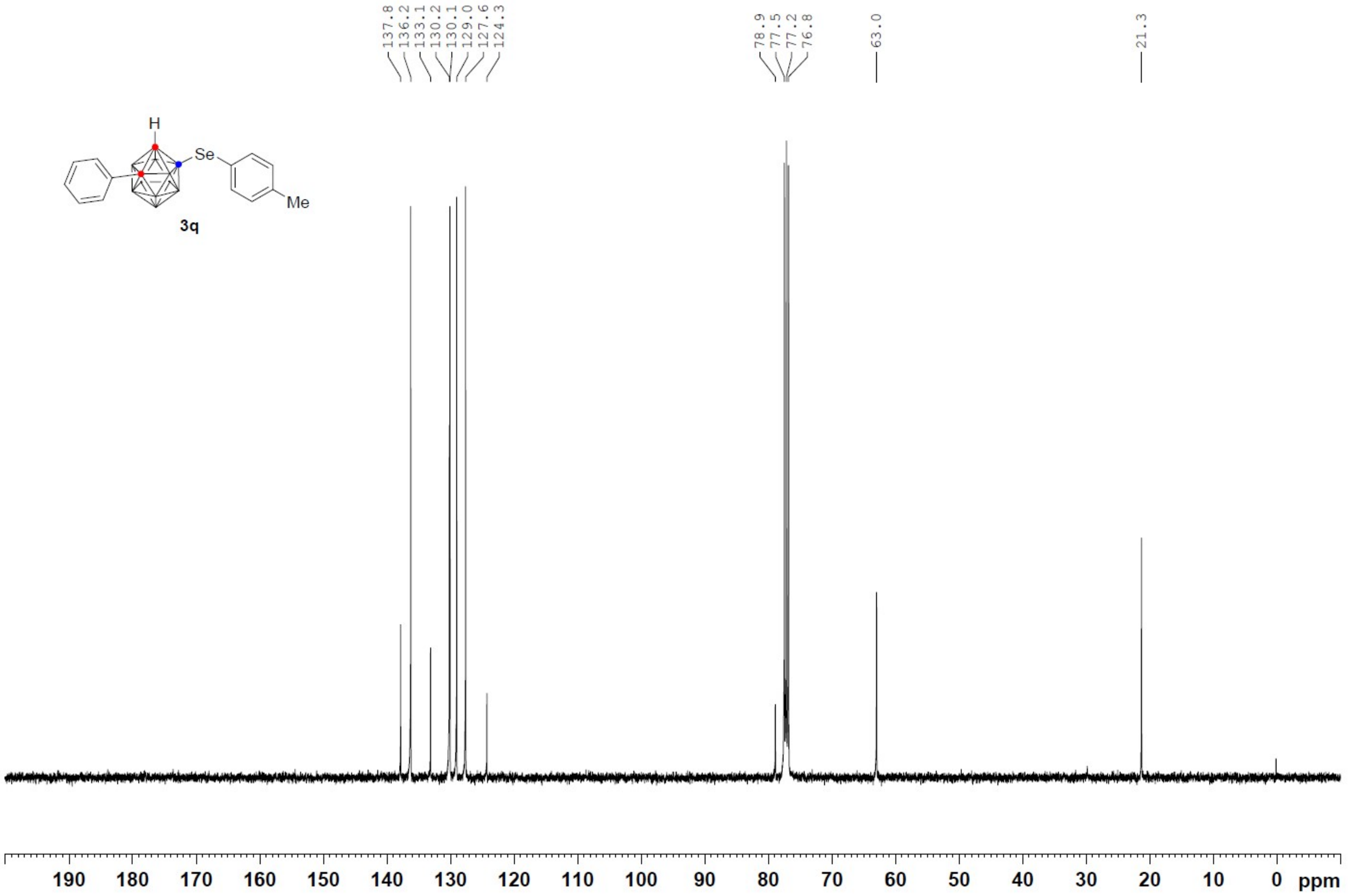

^{11}B NMR (128 MHz, $CDCl_3$)

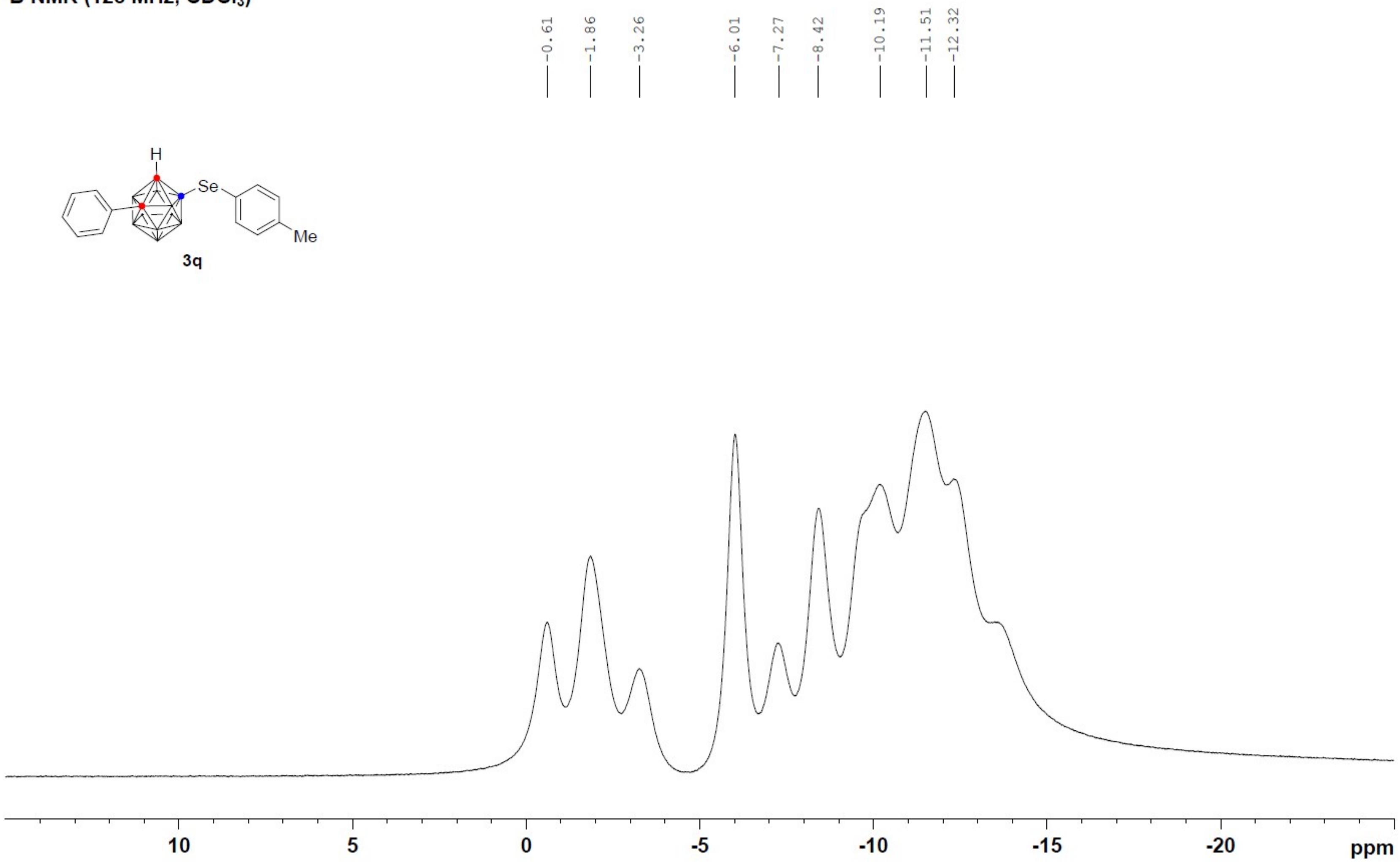

$^{11}B\{^{1}H\}$ NMR (128 MHz, $CDCl_3$)

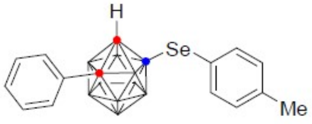

3q

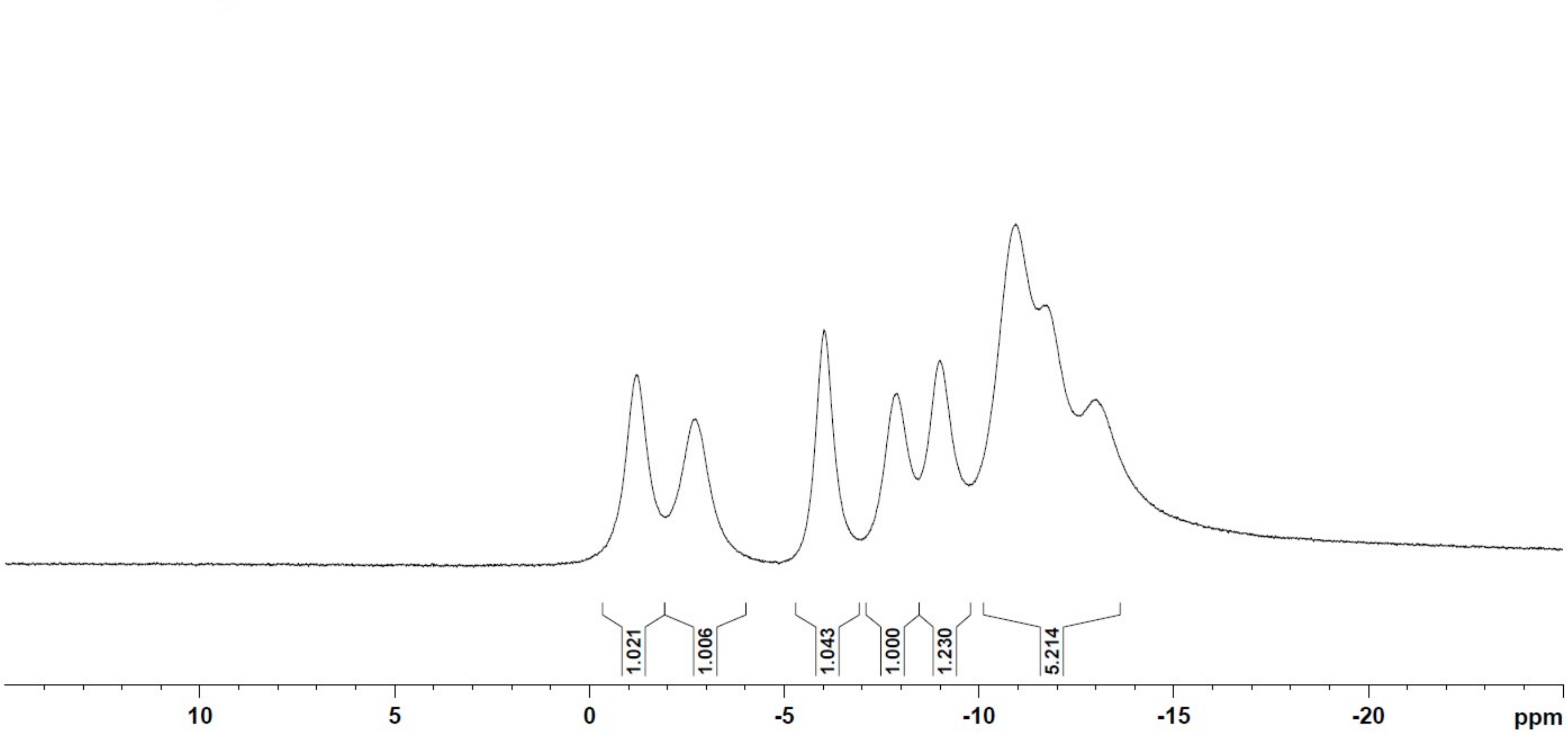

^1H NMR (400 MHz, $CDCl_3$)

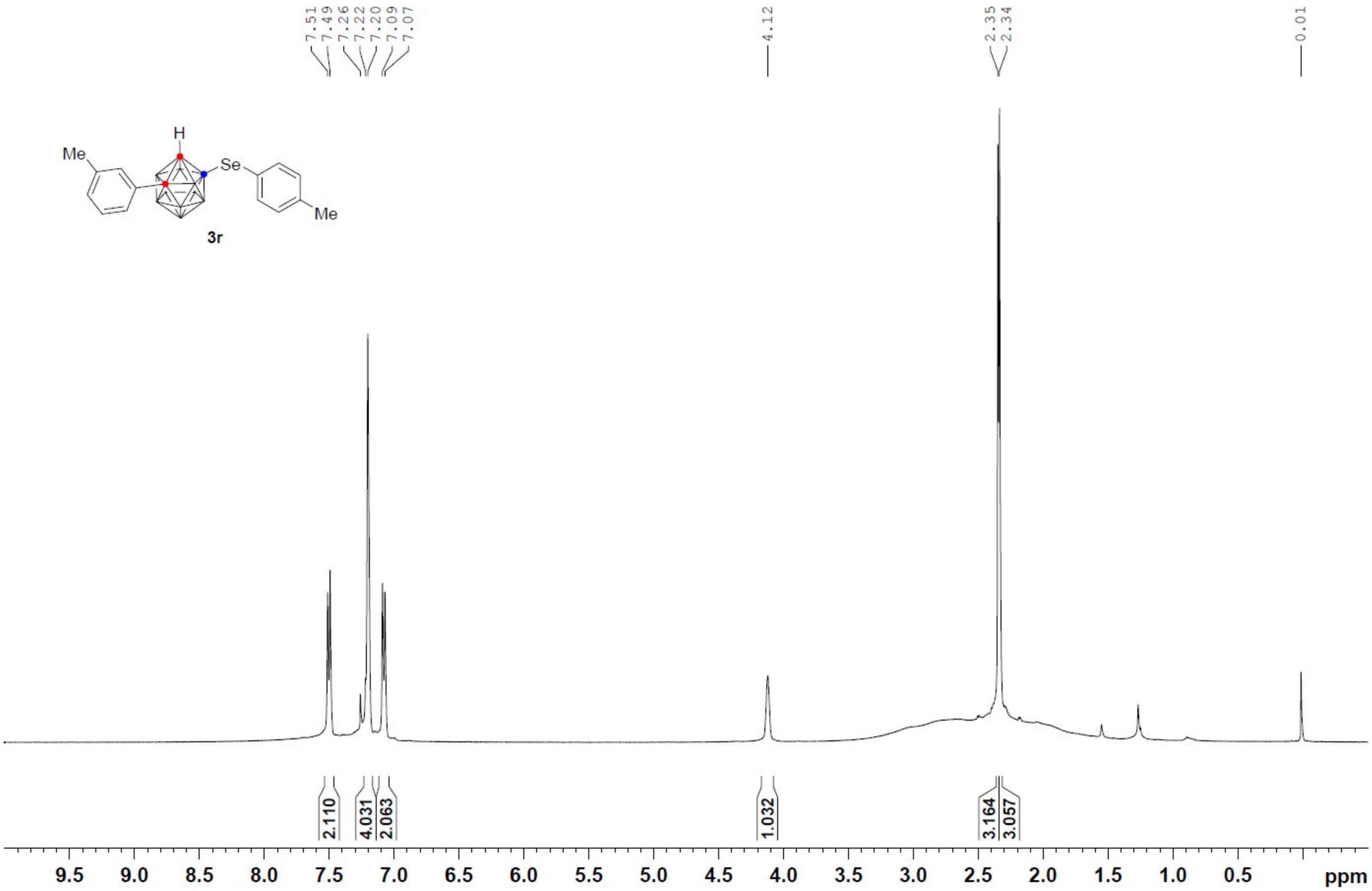

^{13}C NMR (100 MHz, $CDCl_3$)

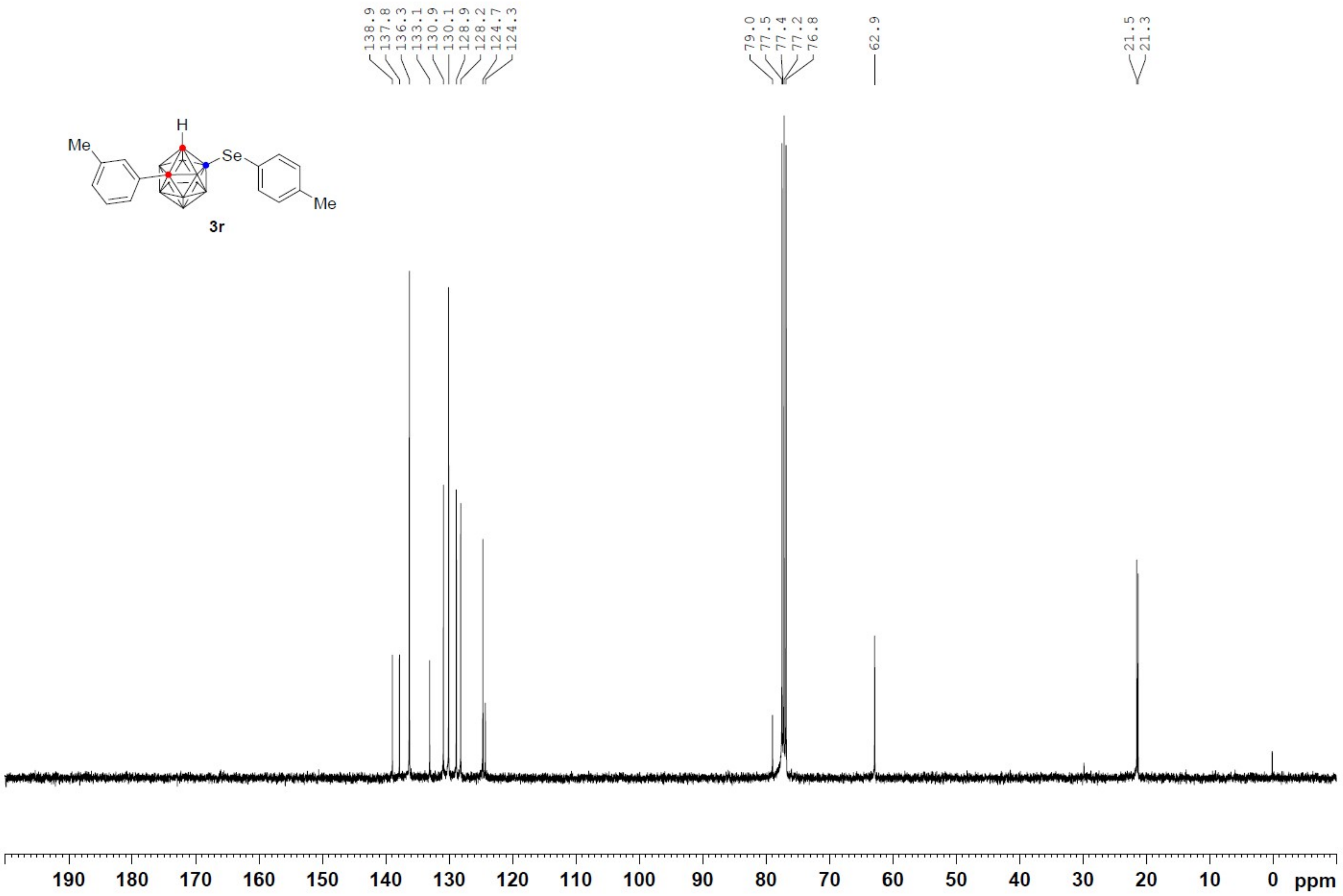

^{11}B NMR (128 MHz, $CDCl_3$)

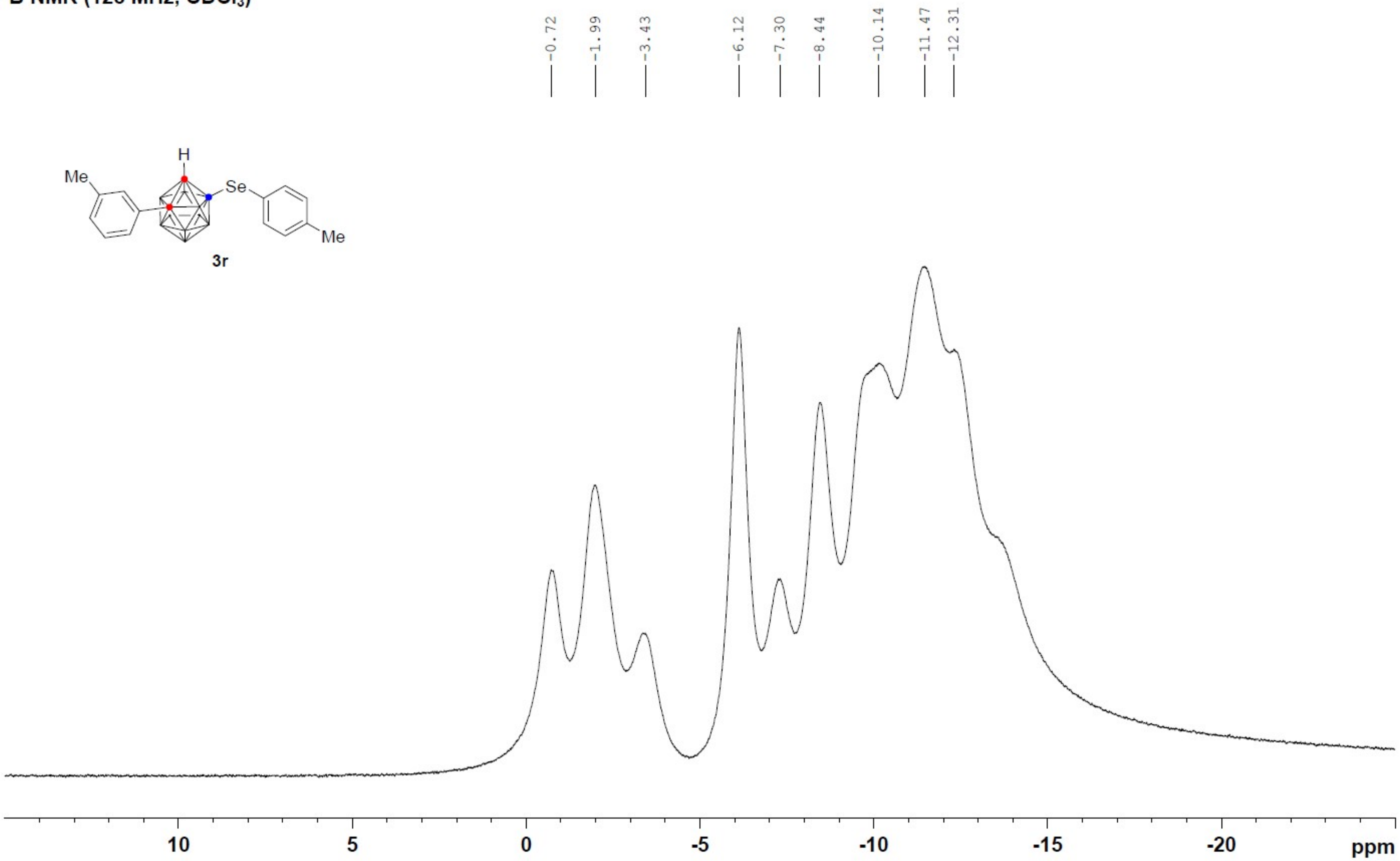

$^{11}B\{^1H\}$ NMR (128 MHz, $CDCl_3$)

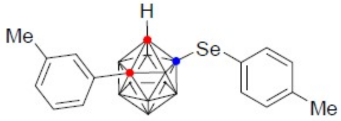

3r

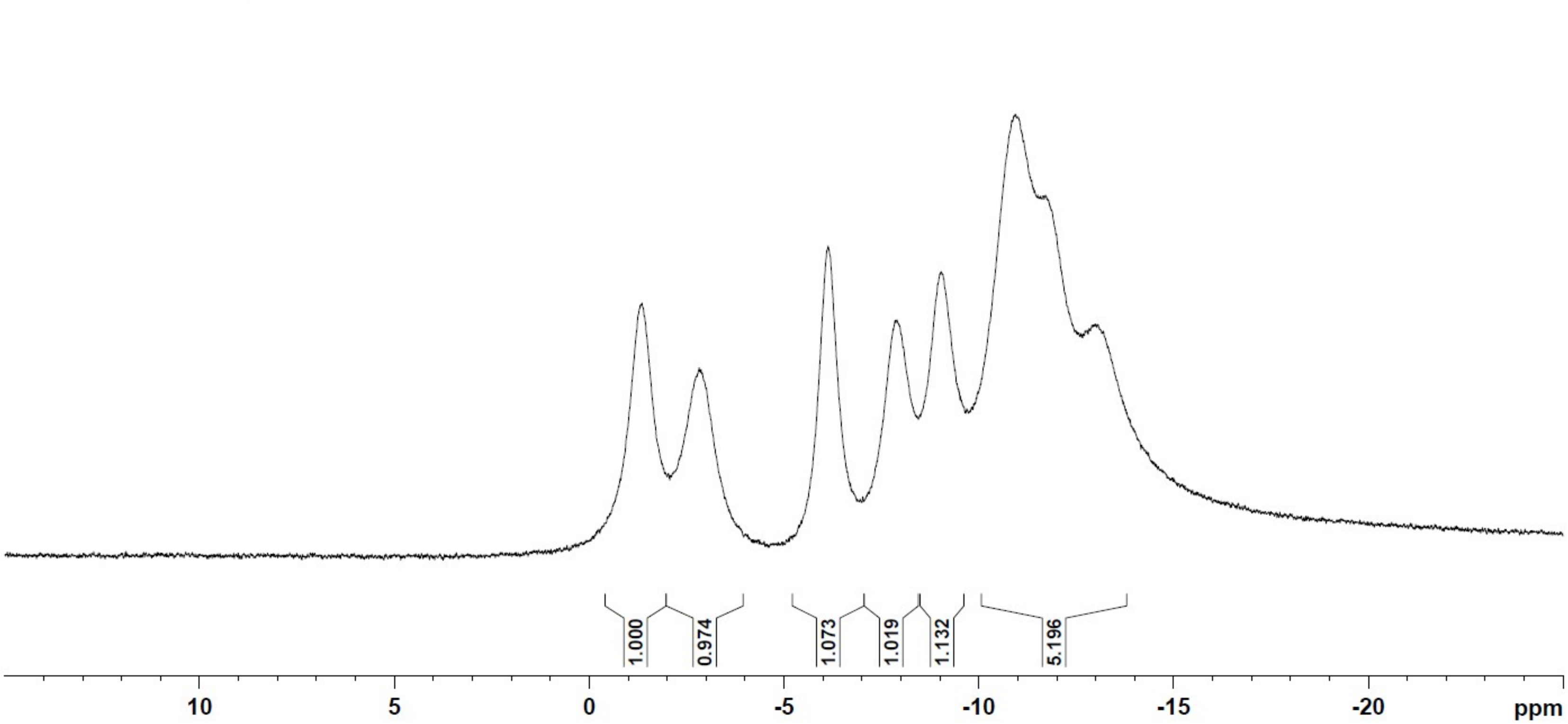

^{1}H NMR (400 MHz, $CDCl_3$)

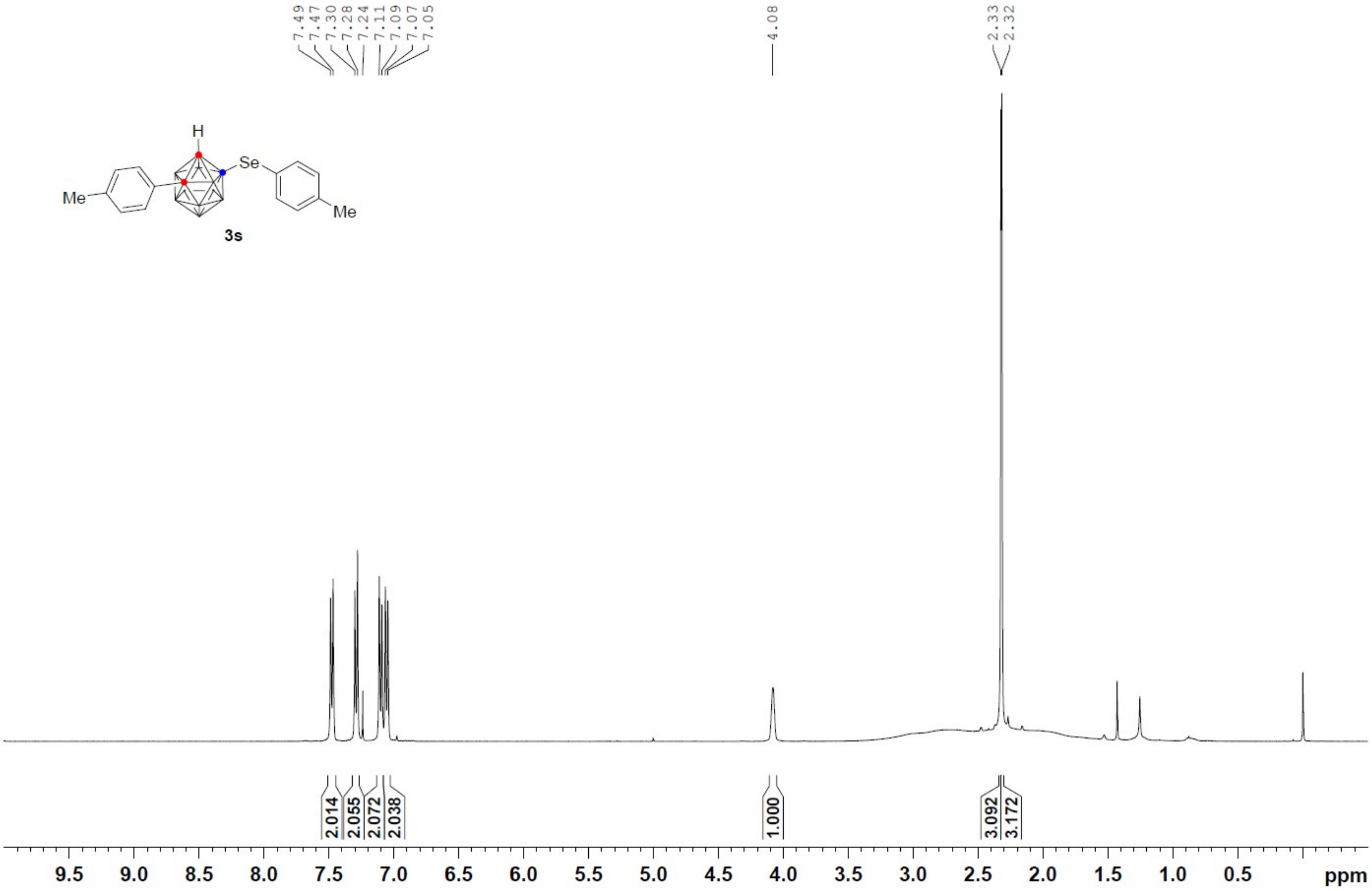

3s

^{13}C NMR (100 MHz, $CDCl_3$)

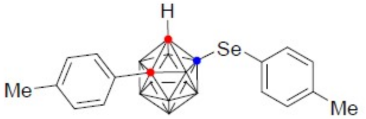

3s

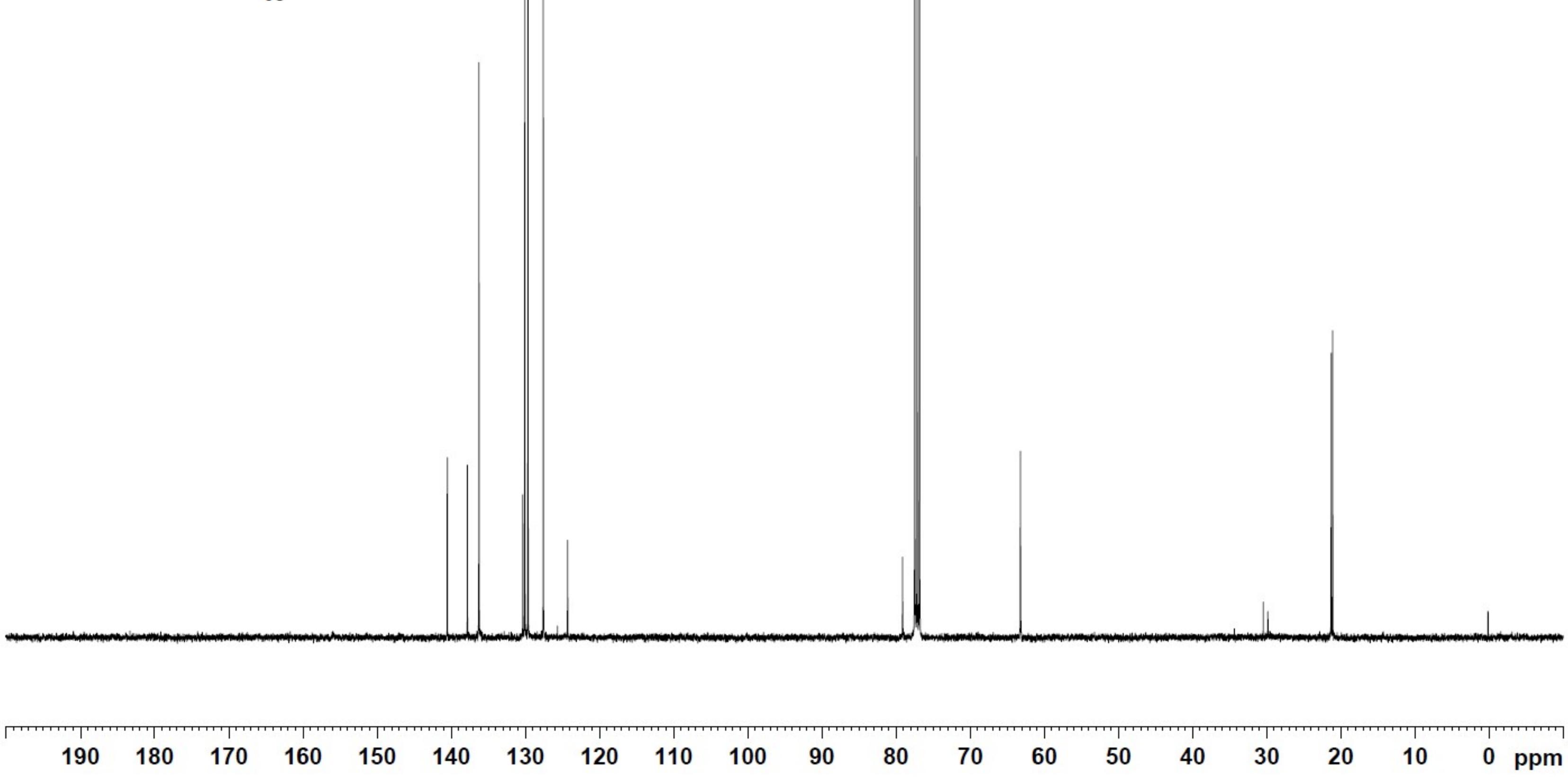

^{11}B NMR (128 MHz, $CDCl_3$)

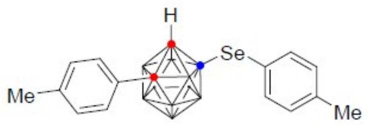

3s

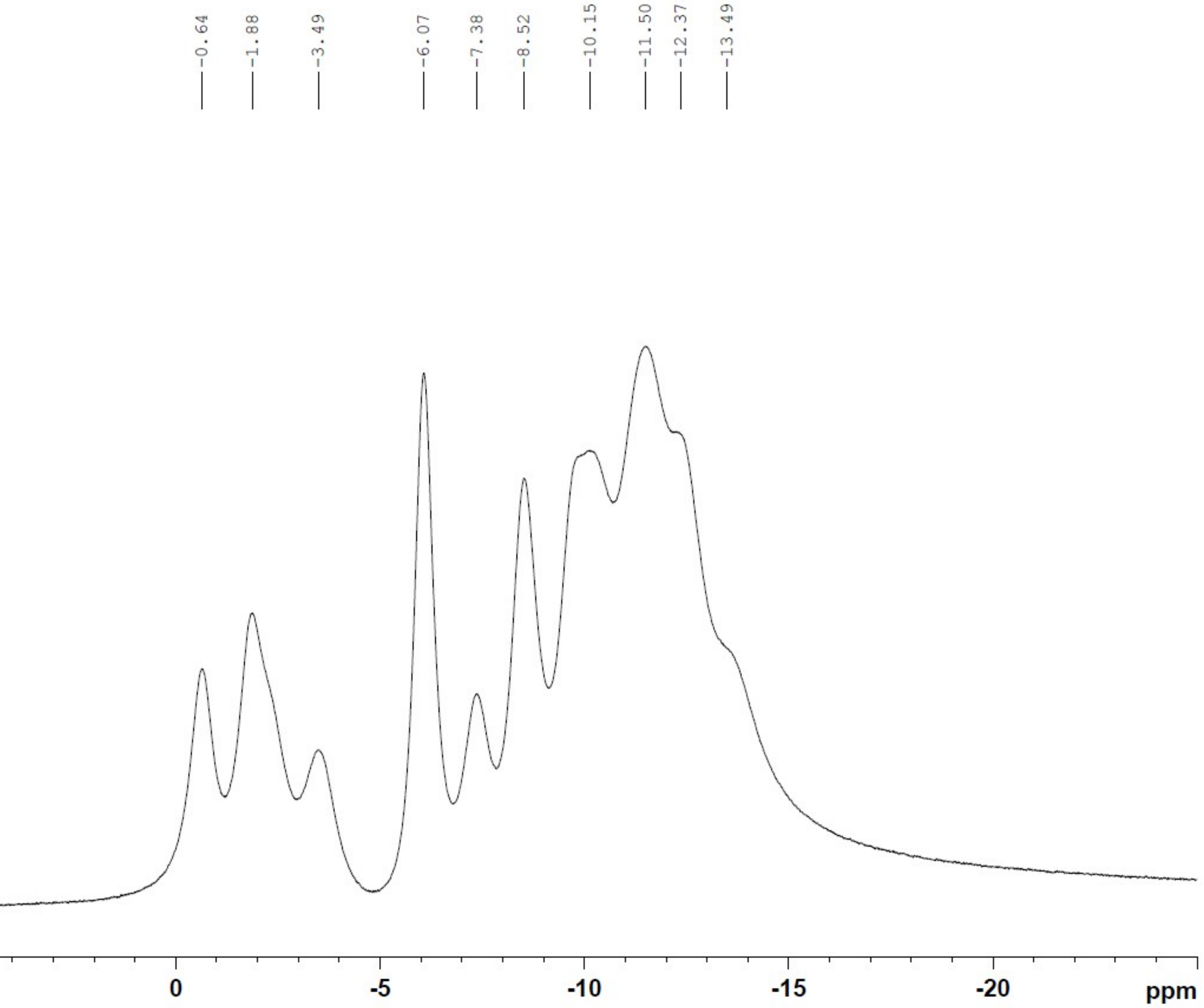

$^{11}B\{^{1}H\}$ NMR (128 MHz, $CDCl_3$)

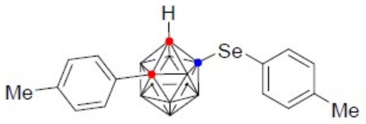

3s

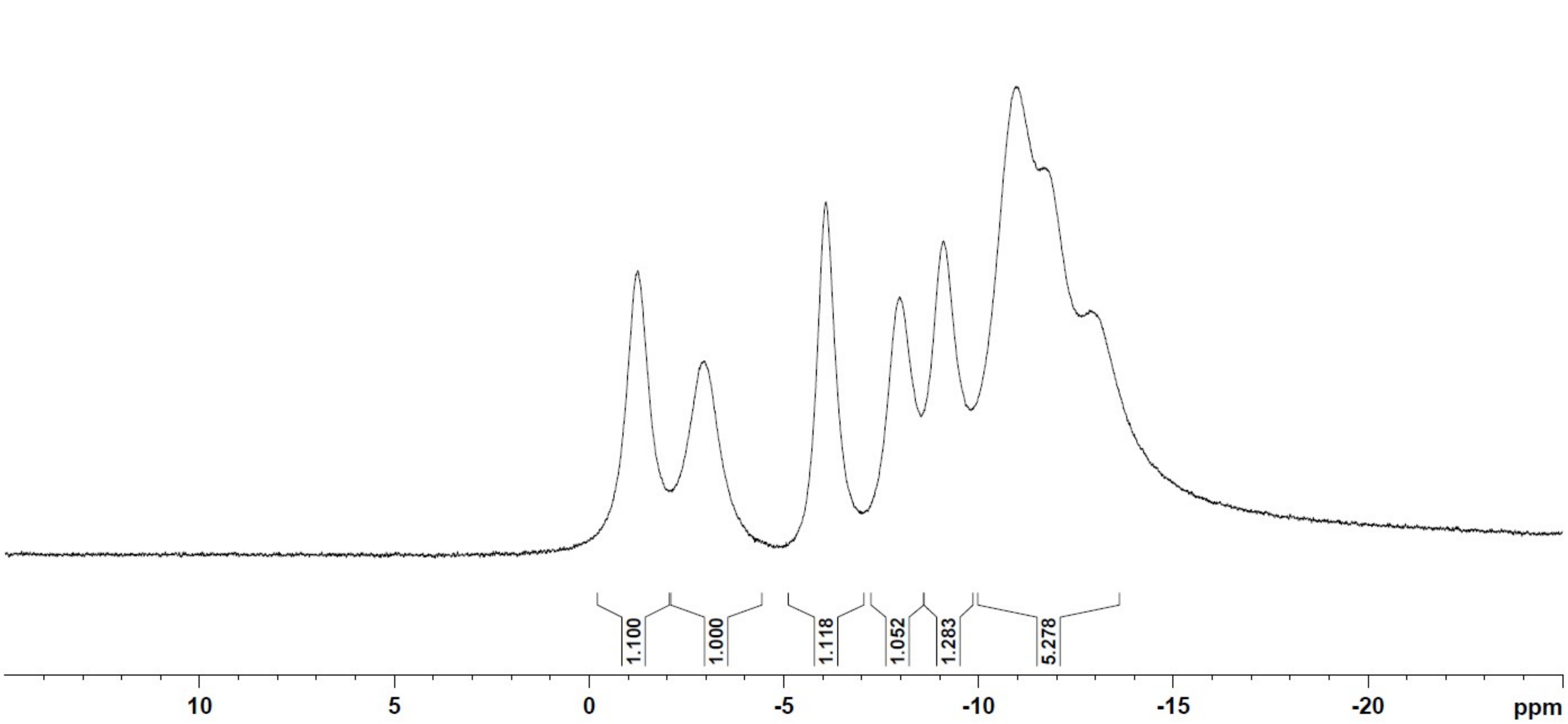

^{1}H NMR (400 MHz, $CDCl_3$)

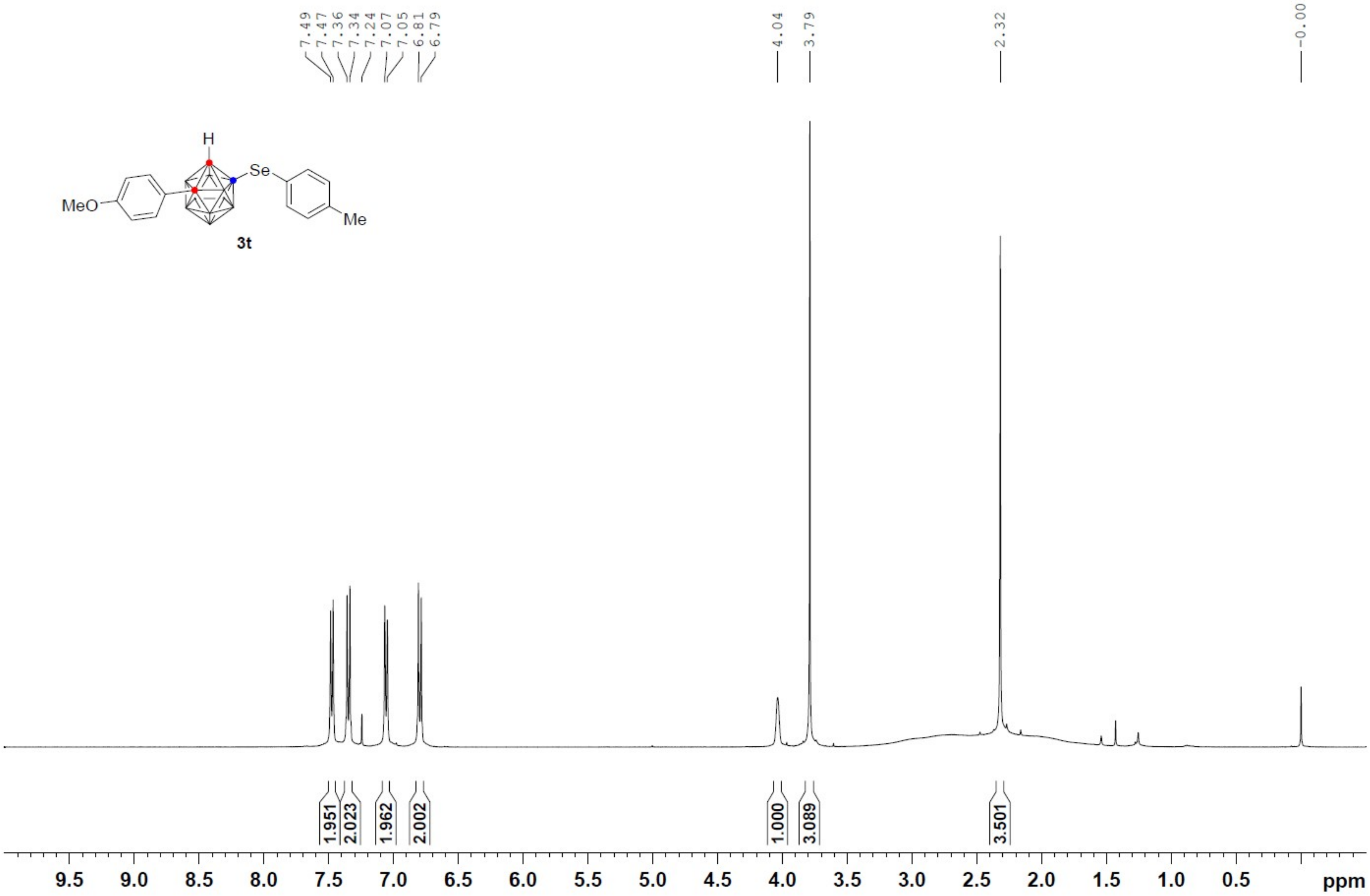

^{13}C NMR (100 MHz, $CDCl_3$)

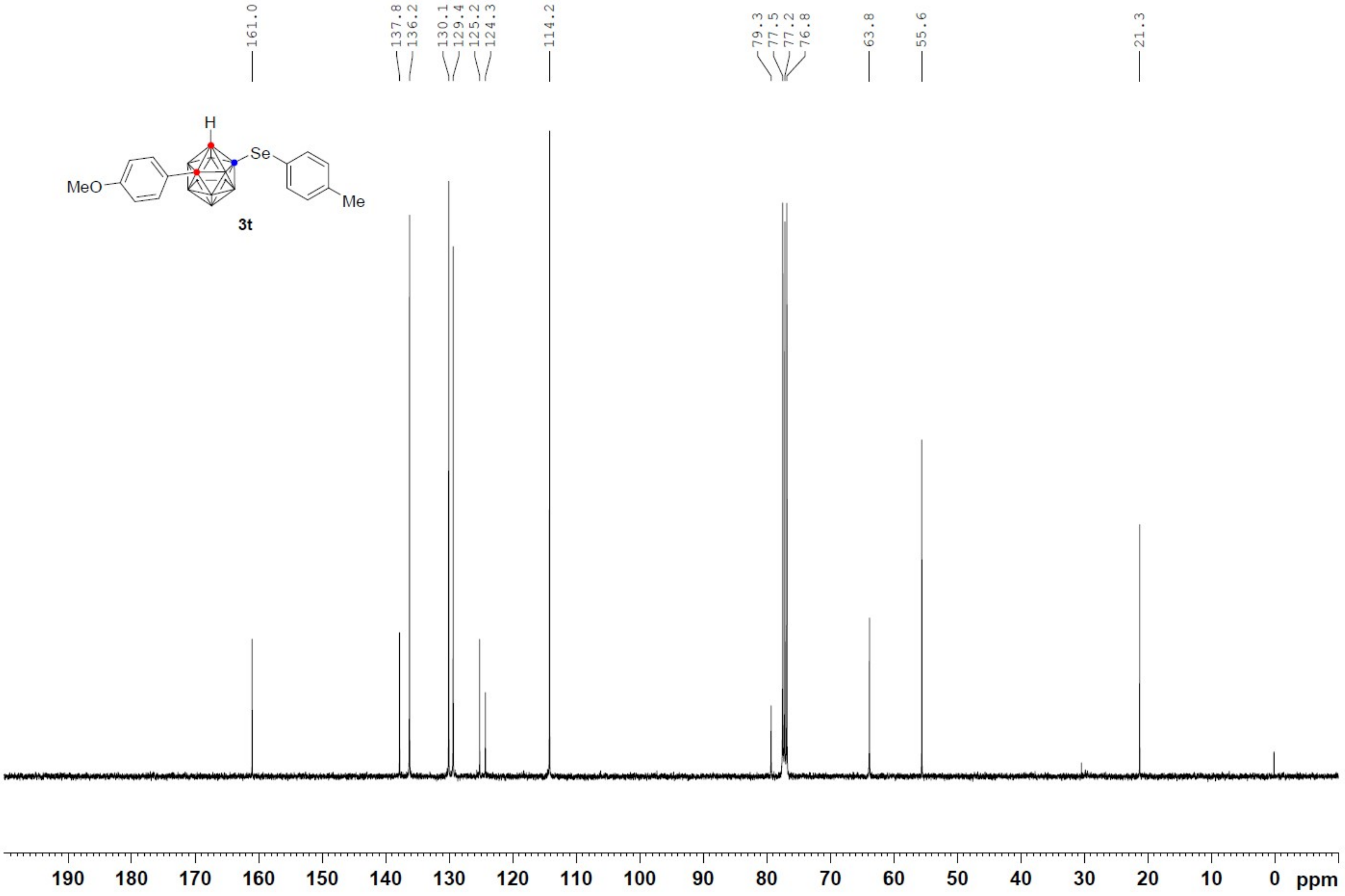

^{11}B NMR (128 MHz, $CDCl_3$)

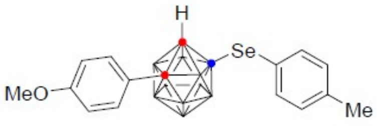

3t

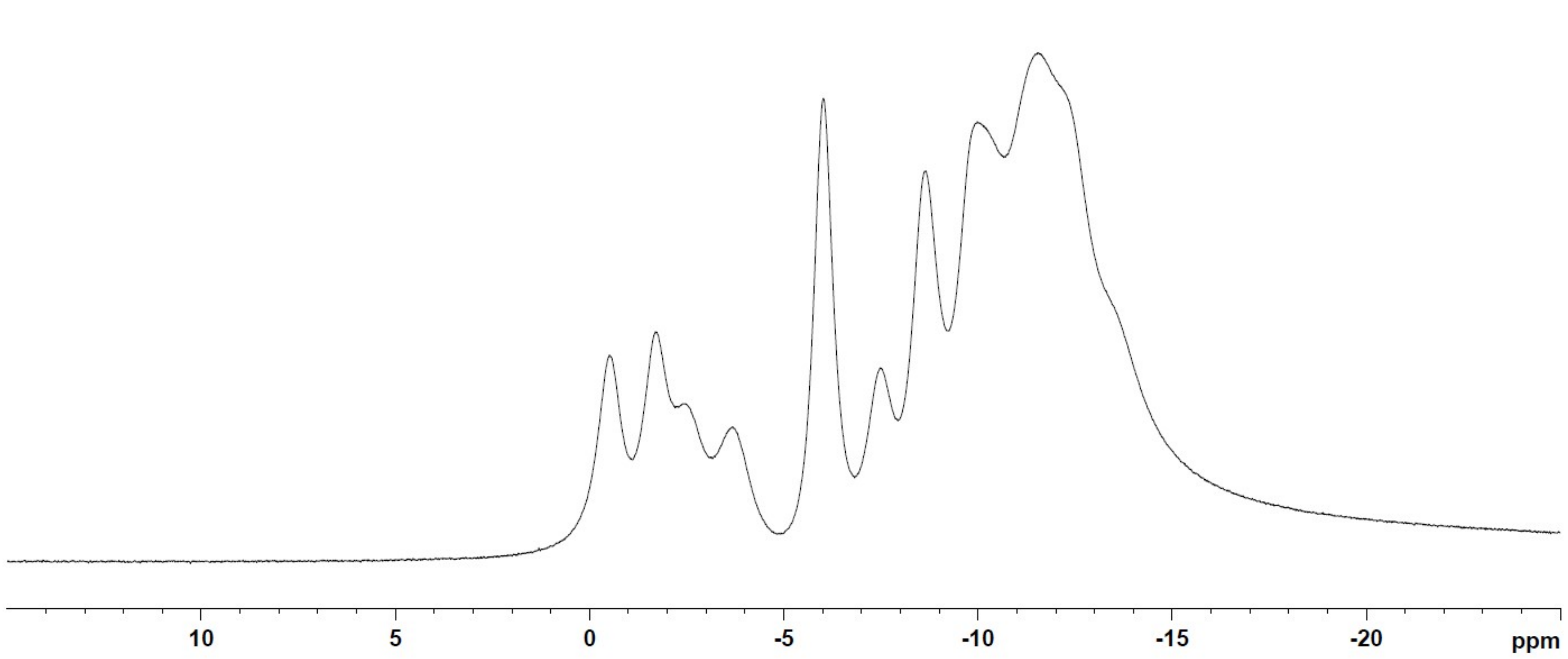

$^{11}B\{^1H\}$ NMR (128 MHz, $CDCl_3$)

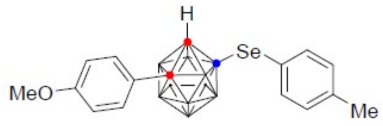

3t

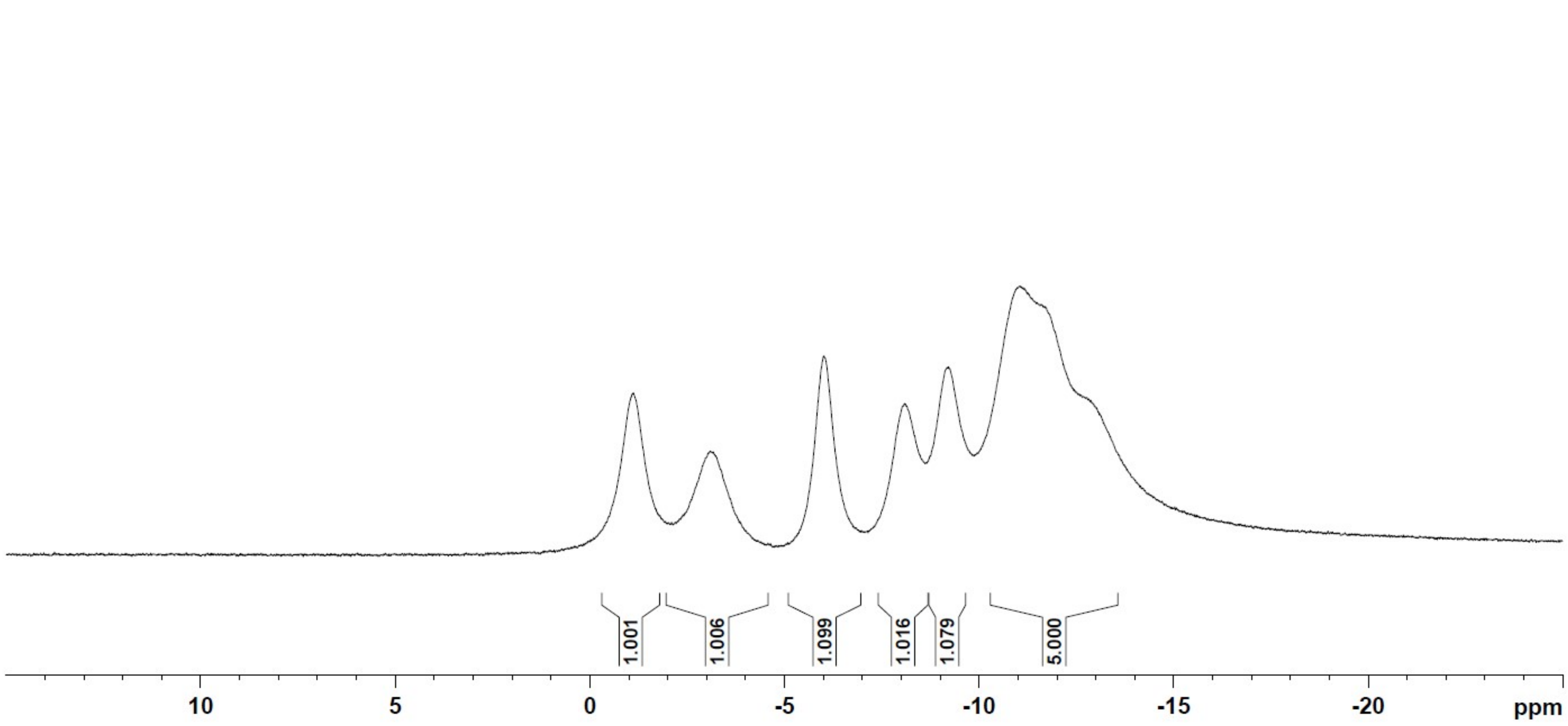

^{1}H NMR (400 MHz, CDCl$_3$)

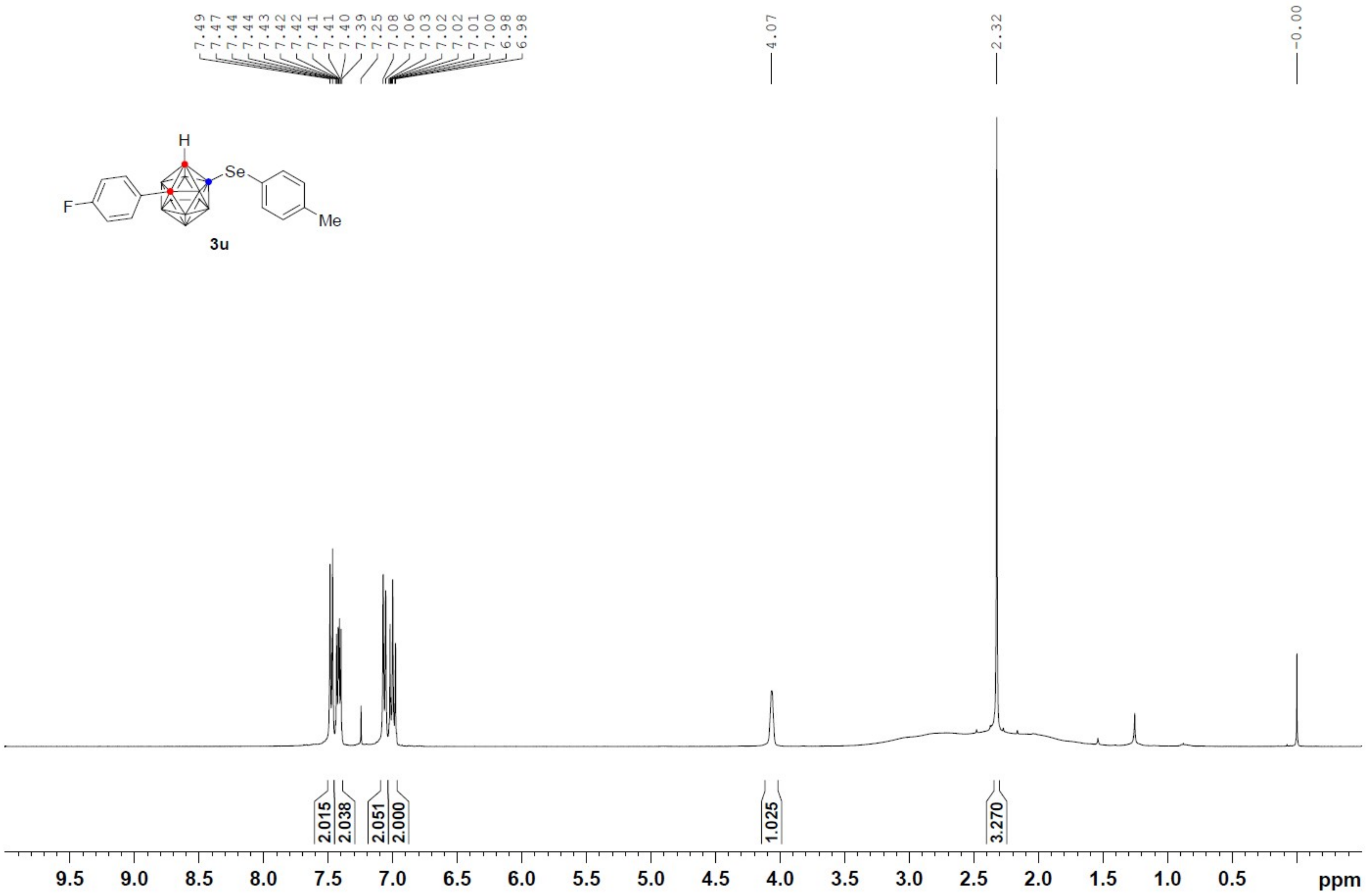

^{13}C NMR (100 MHz, $CDCl_3$)

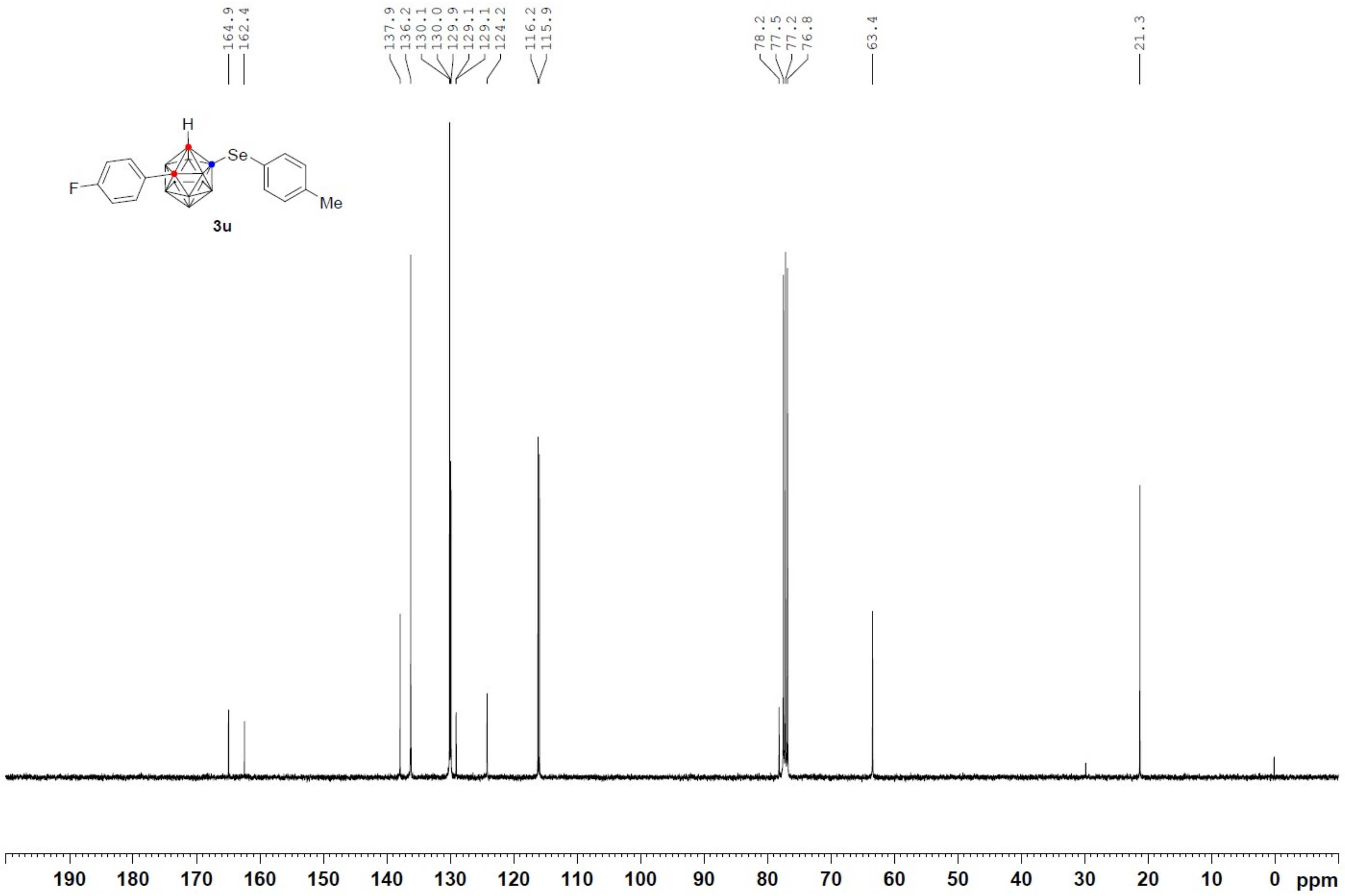

^{11}B NMR (128 MHz, $CDCl_3$)

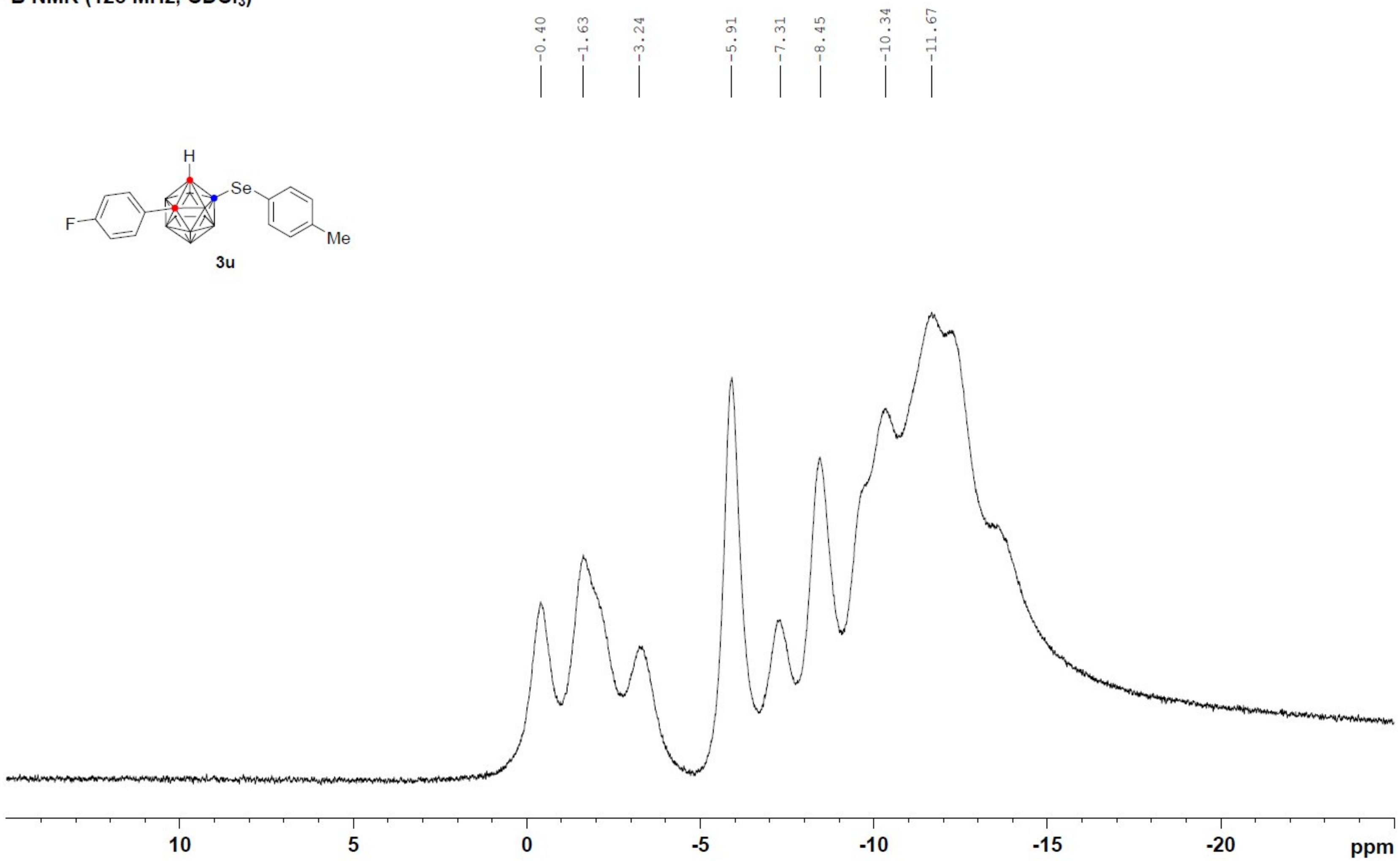

$^{11}B\{^{1}H\}$ NMR (128 MHz, $CDCl_3$)

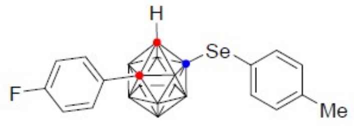

3u

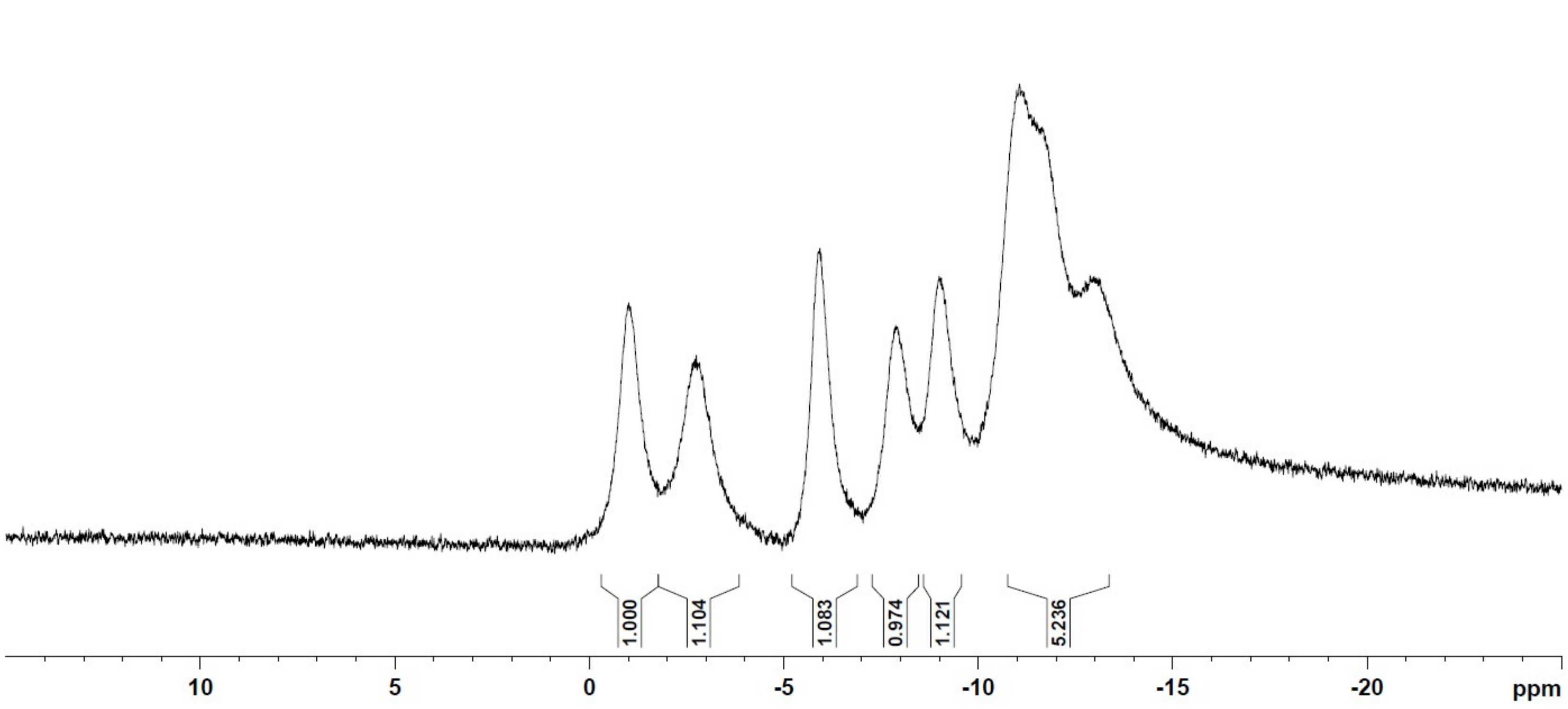

$^{19}F\{^1H\}$ NMR (377 MHz, $CDCl_3$)

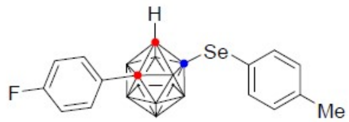

3u

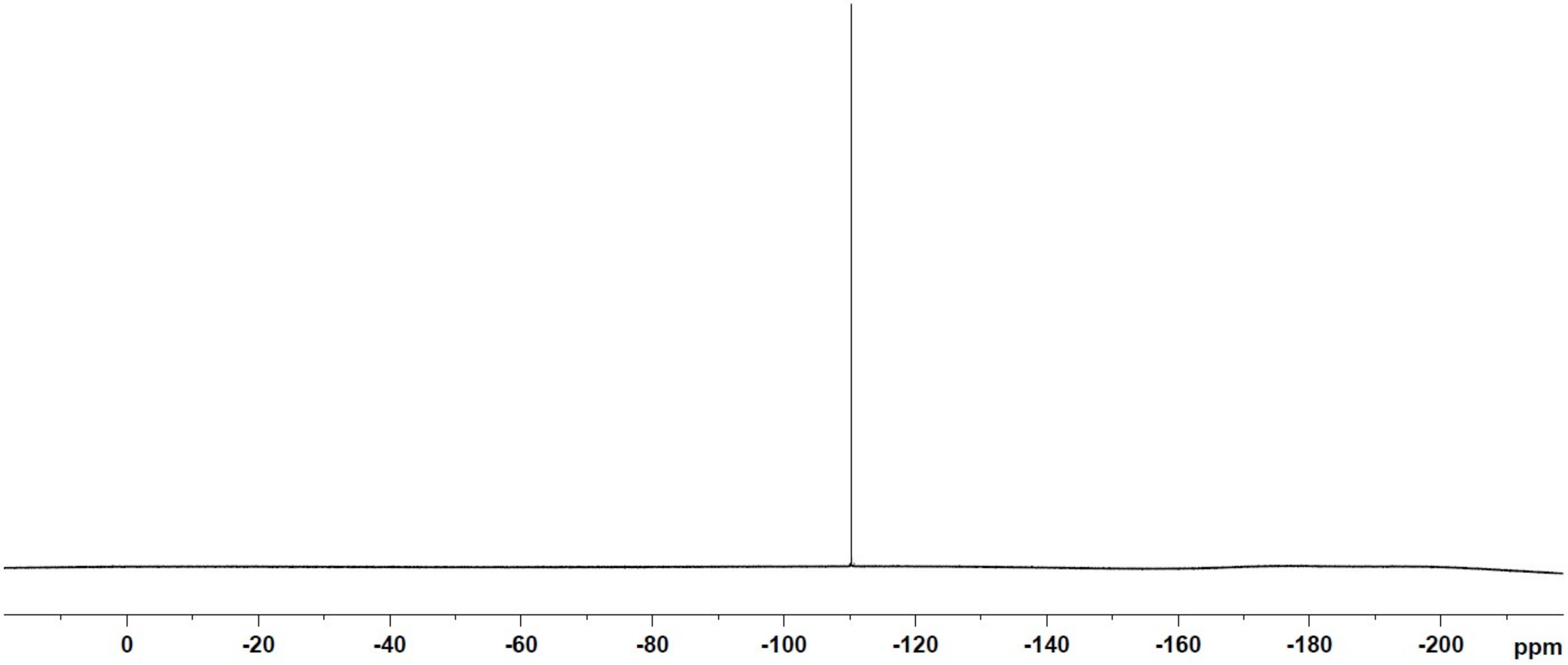

^{1}H NMR (400 MHz, CDCl$_3$)

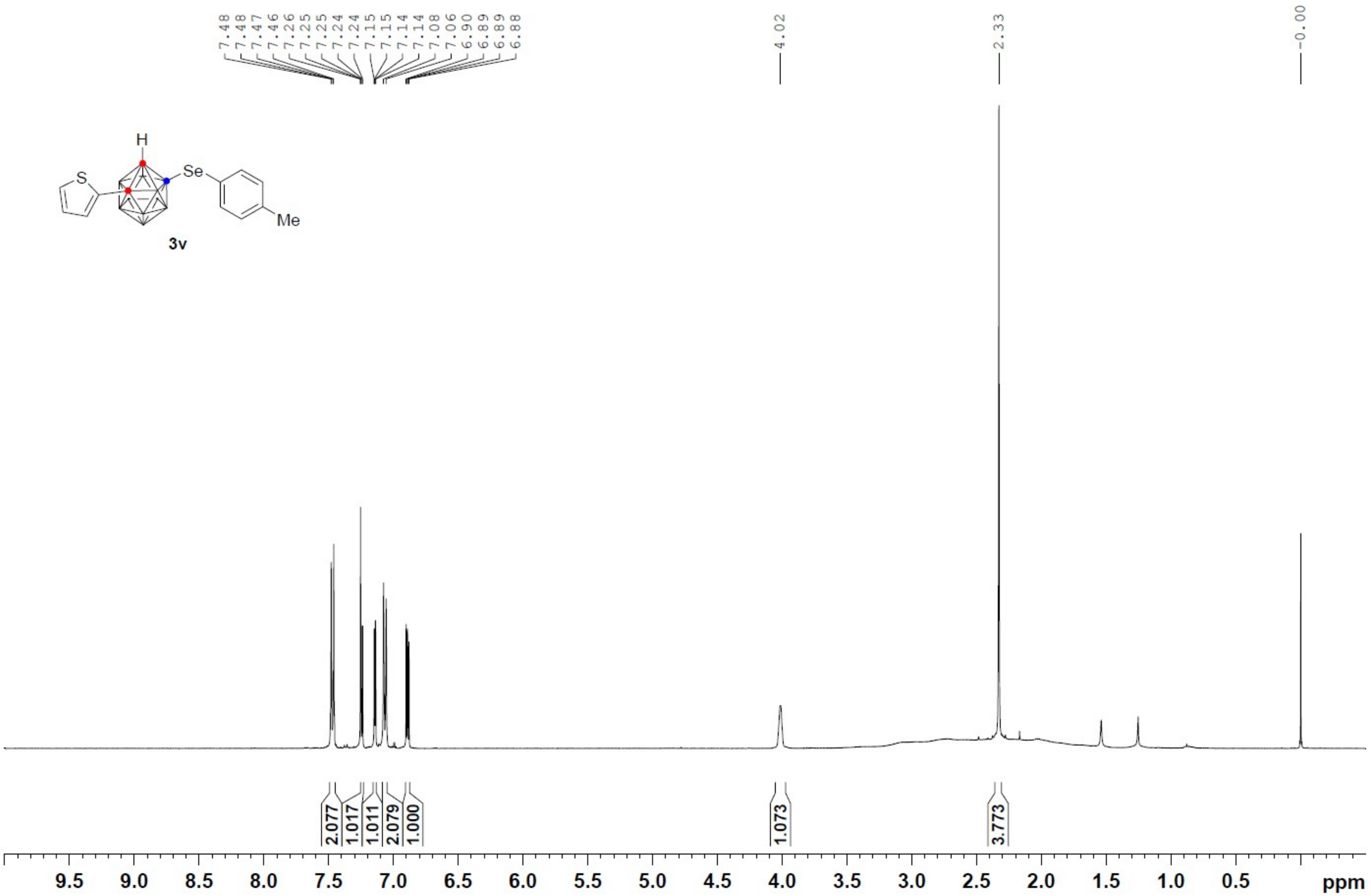

^{13}C NMR (100 MHz, CDCl$_3$)

3v

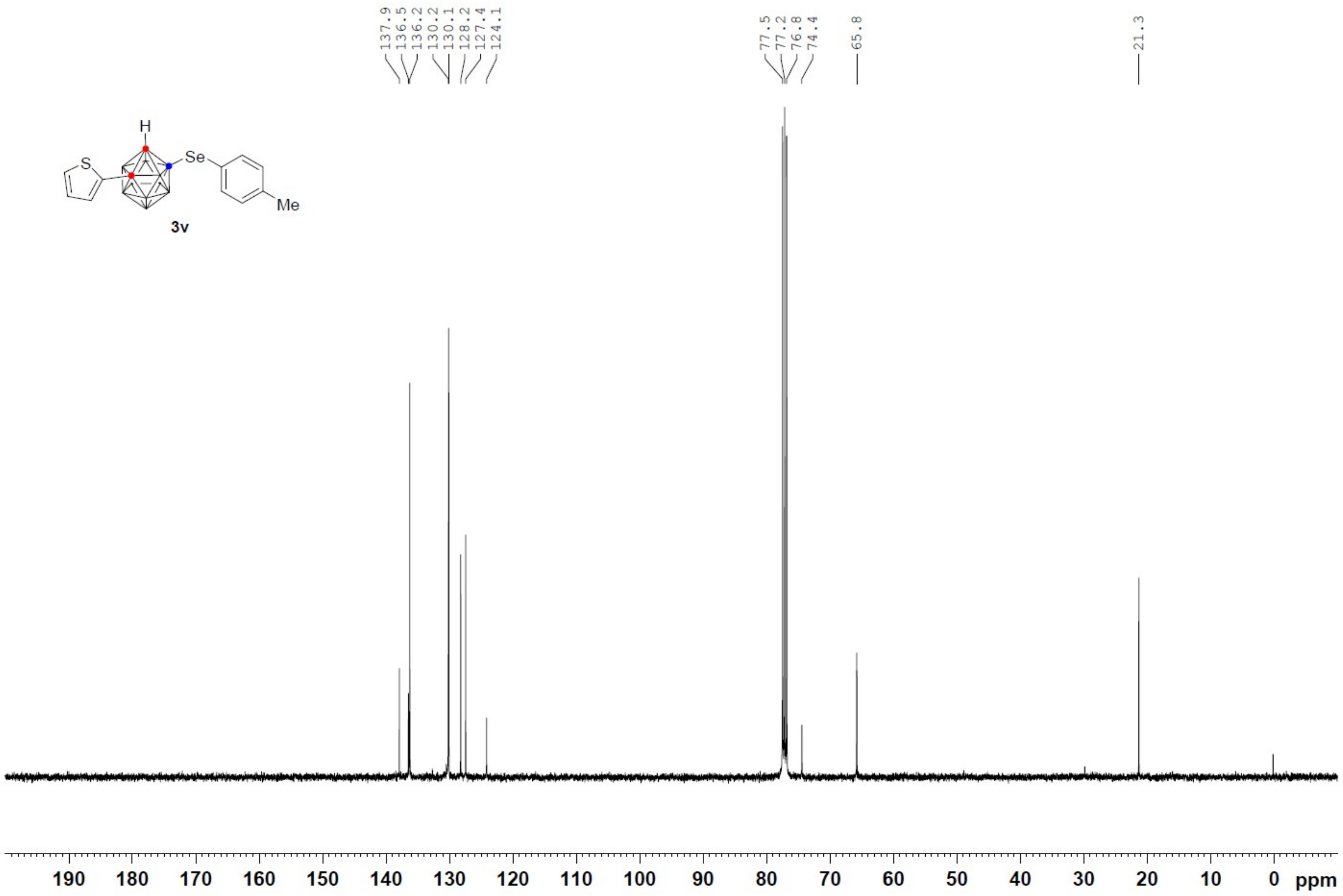

^{11}B NMR (128 MHz, $CDCl_3$)

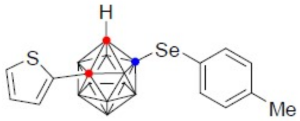

3v

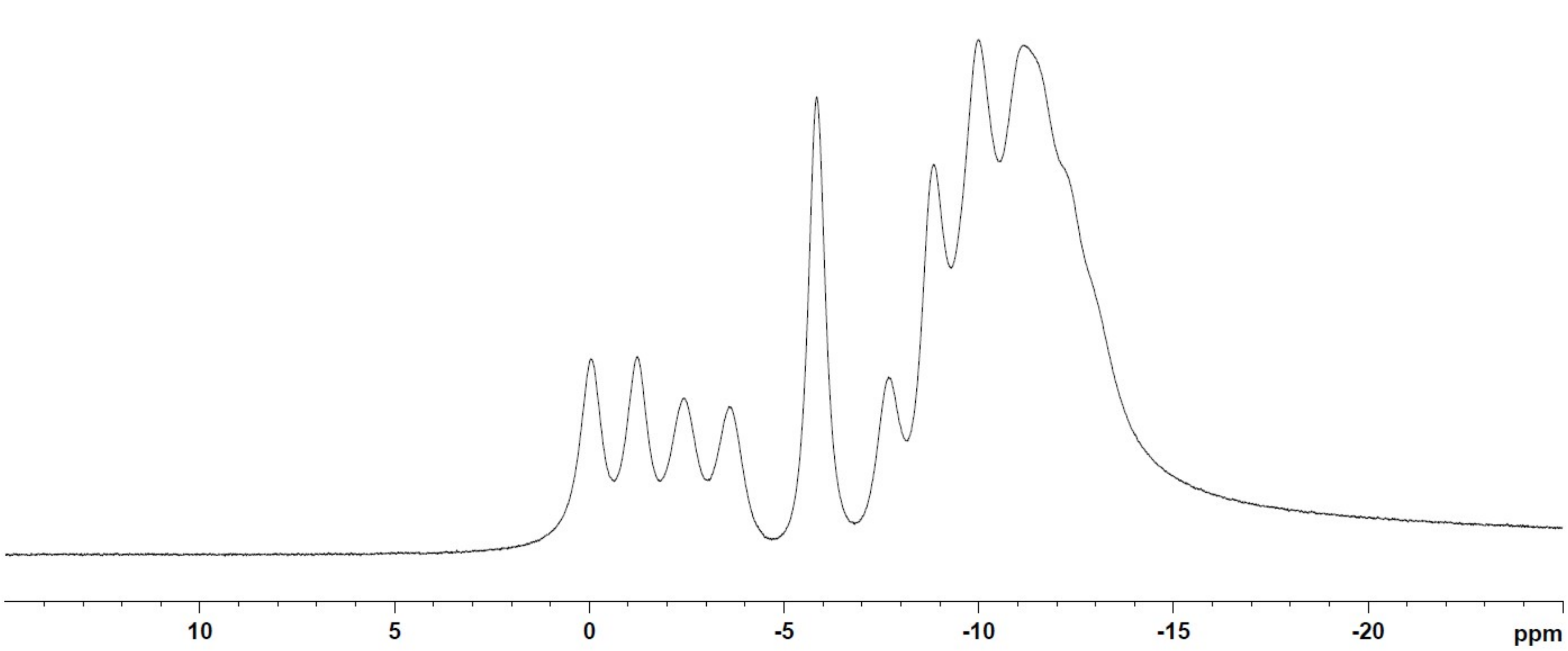

$^{11}B\{^{1}H\}$ NMR (128 MHz, $CDCl_3$)

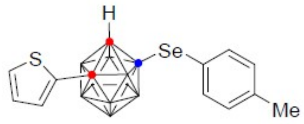

3v

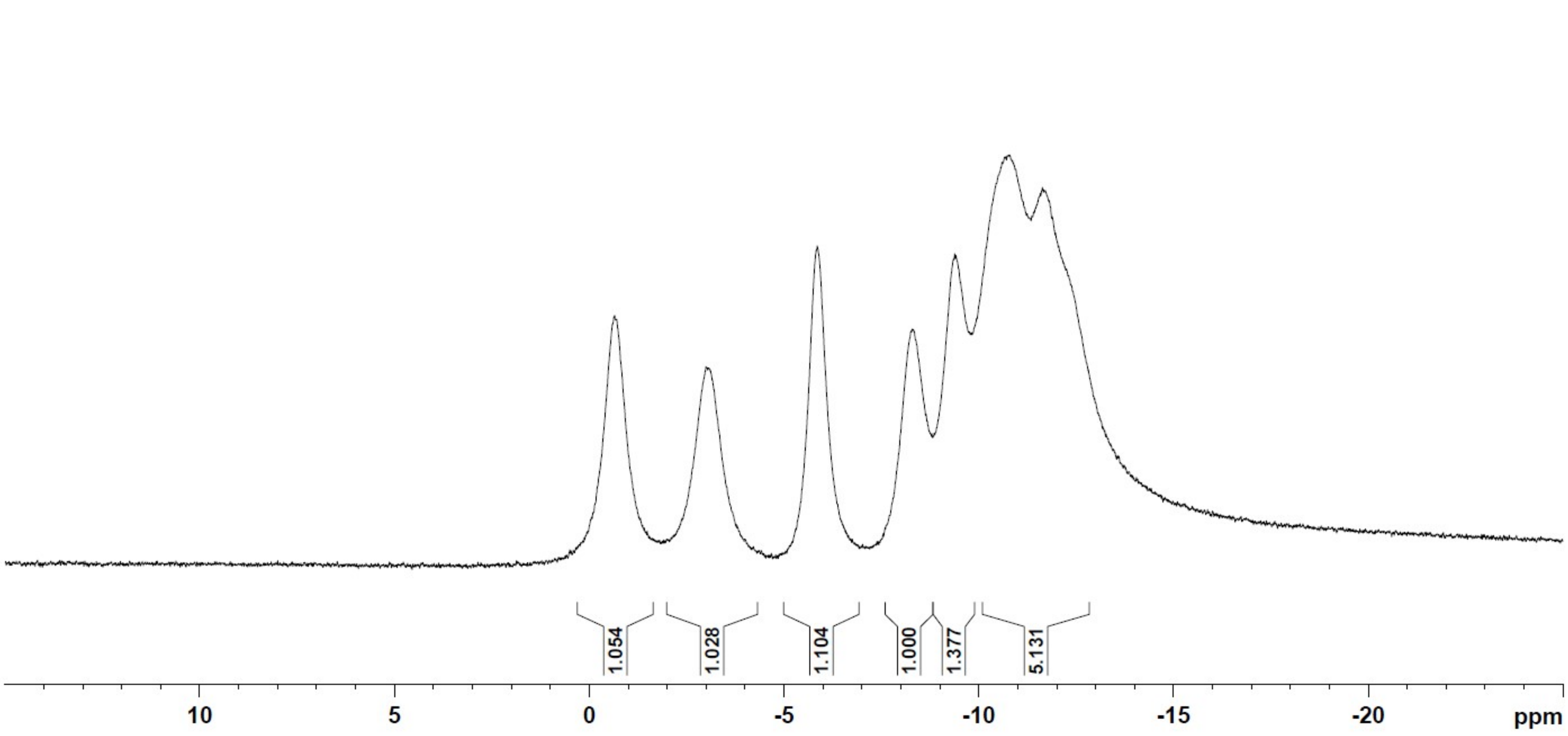

^1H NMR (400 MHz, $CDCl_3$)

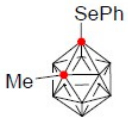

6a

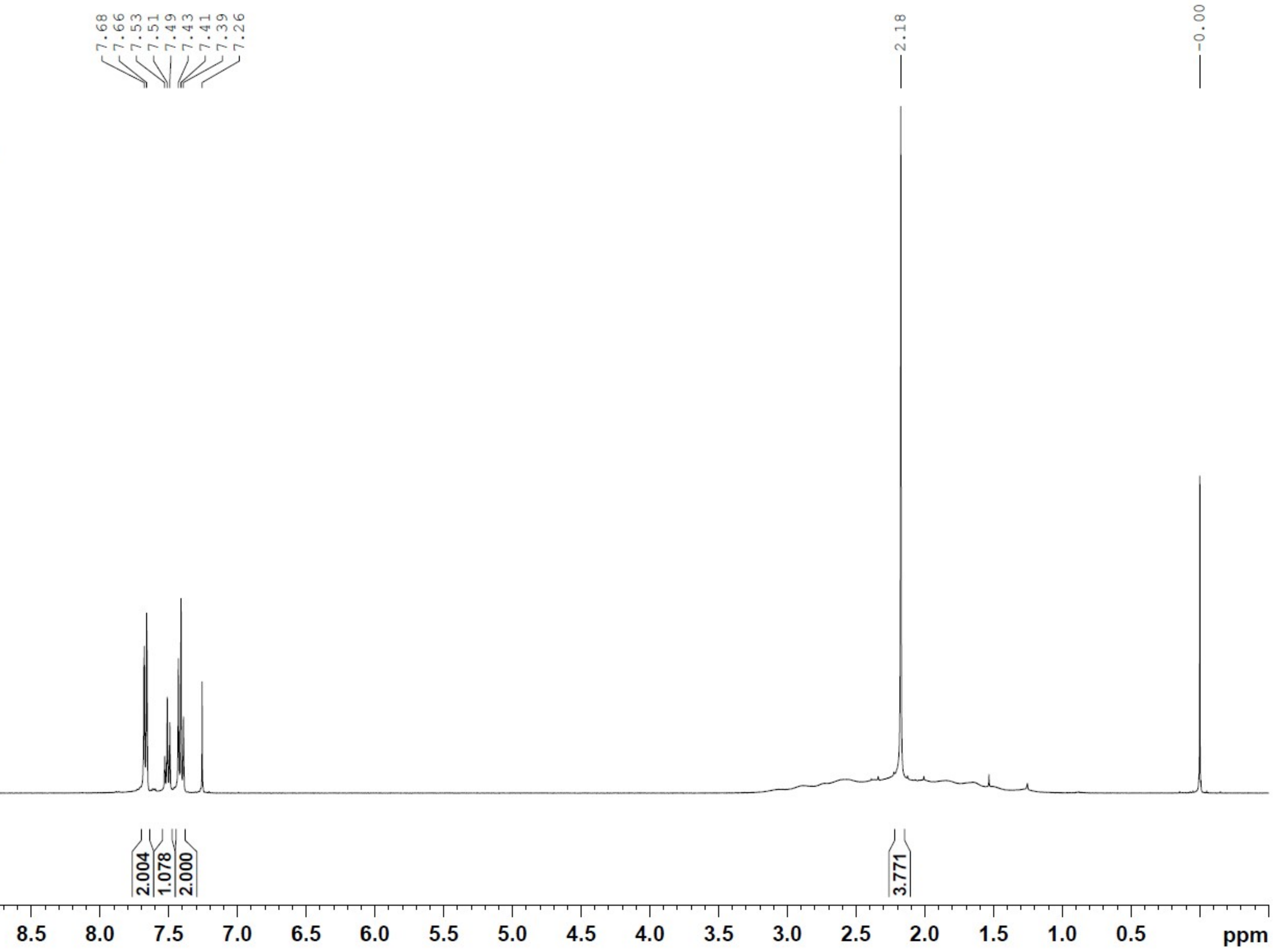

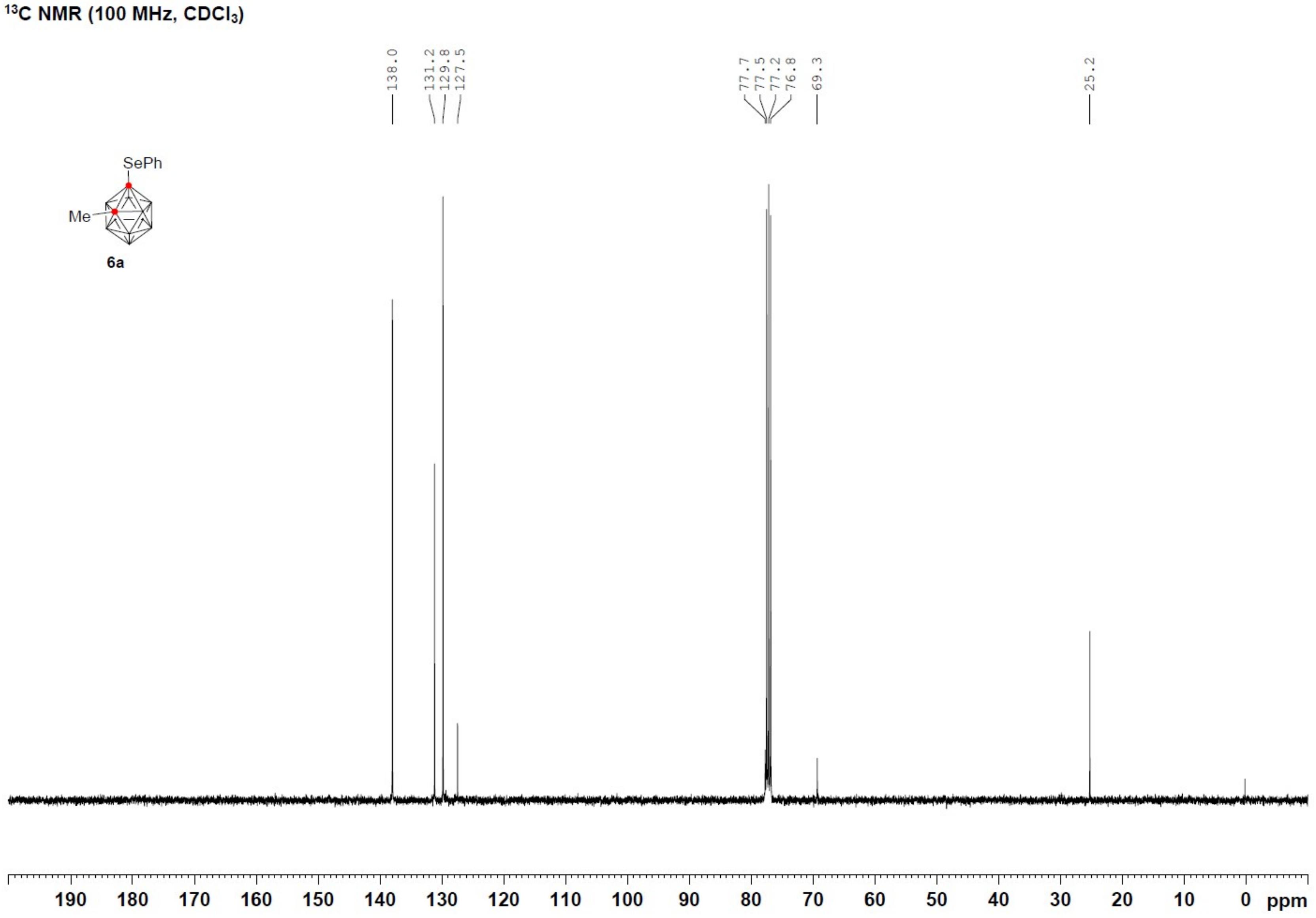

^{13}C NMR (100 MHz, CDCl$_3$)
138.0
131.2
129.8
127.5
77.7
77.5
77.2
76.8
69.3
25.2
SePh
Me
6a
190
180
170
160
150
140
130
120
110
100
90
80
70
60
50
40
30
20
10
0
ppm

^{11}B NMR (128 MHz, $CDCl_3$)

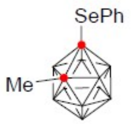

6a

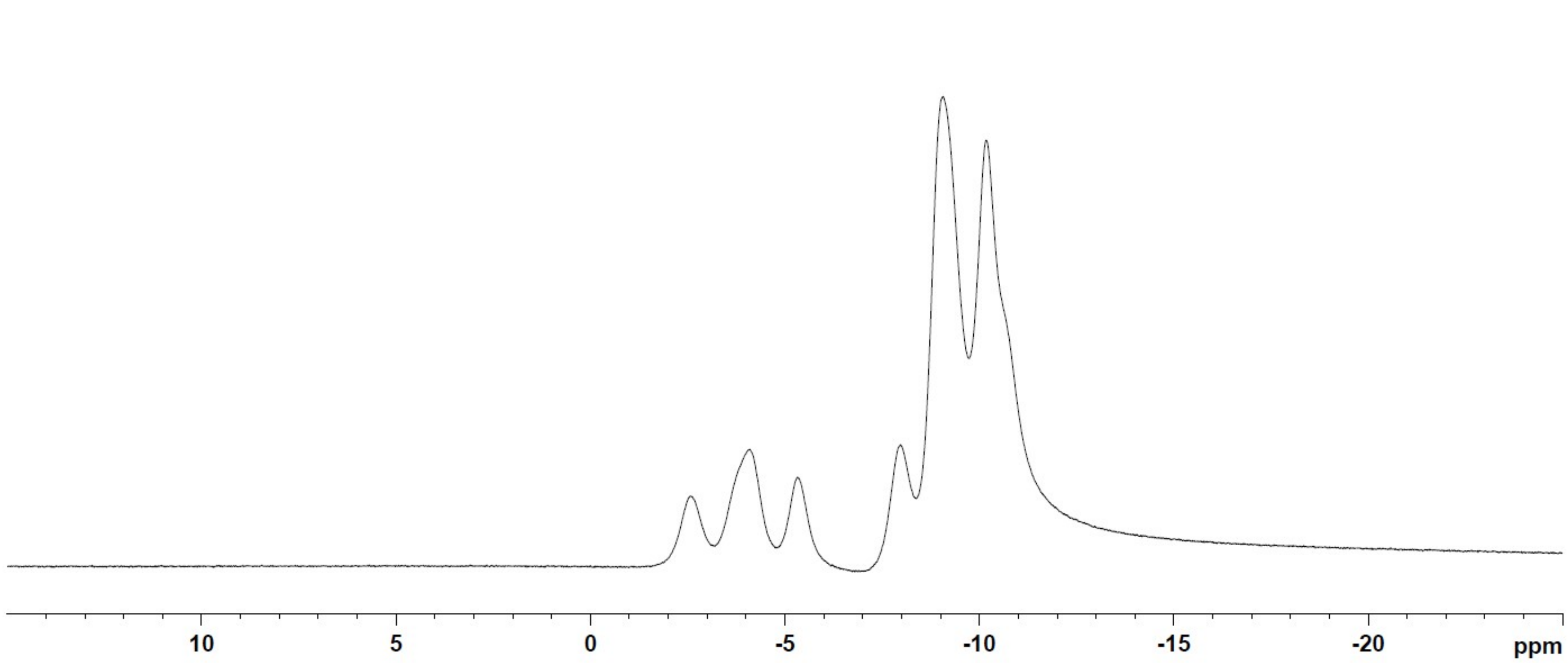

$^{11}B\{^1H\}$ NMR (128 MHz, $CDCl_3$)

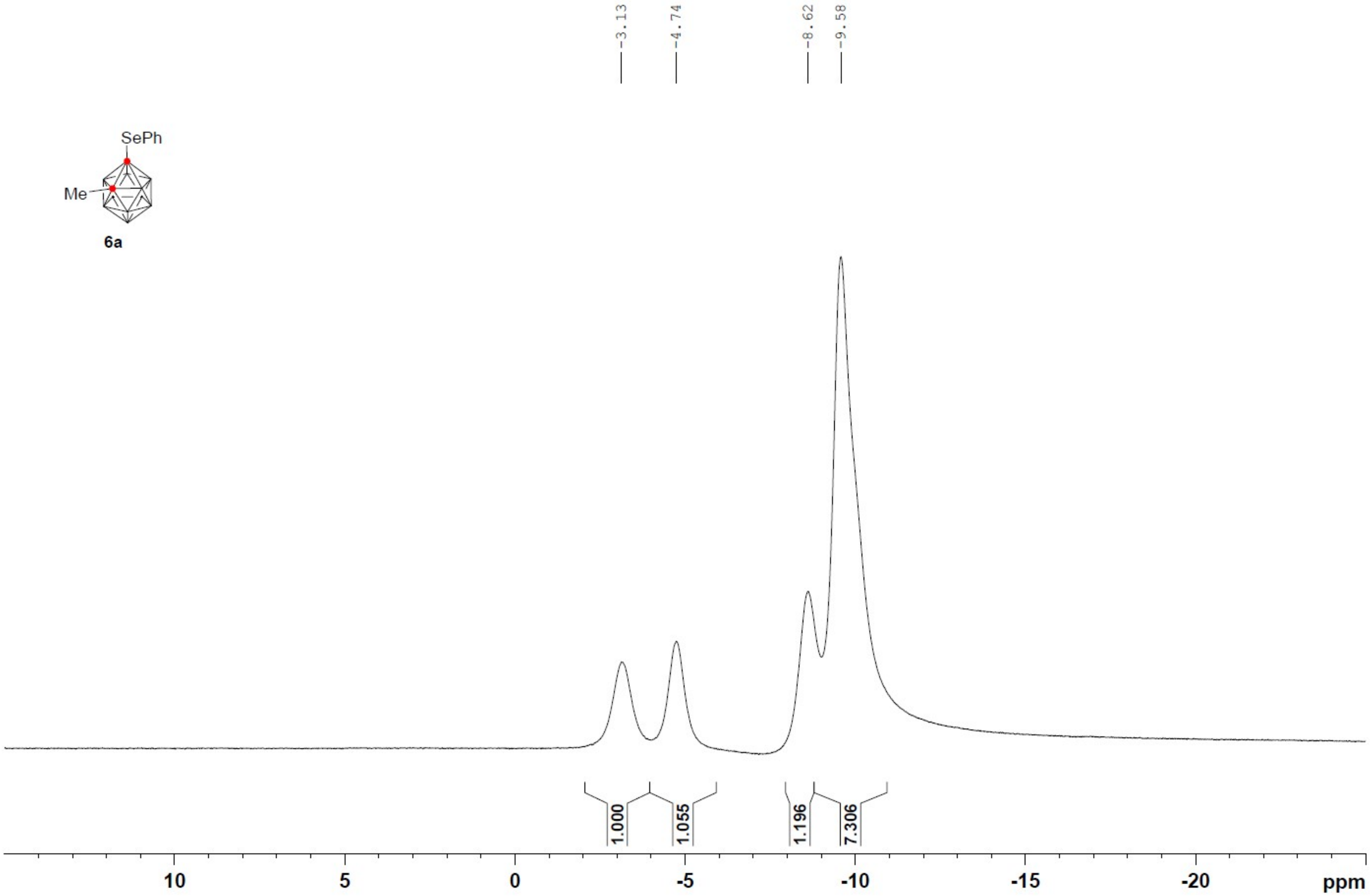

^{1}H NMR (400 MHz, $CDCl_3$)

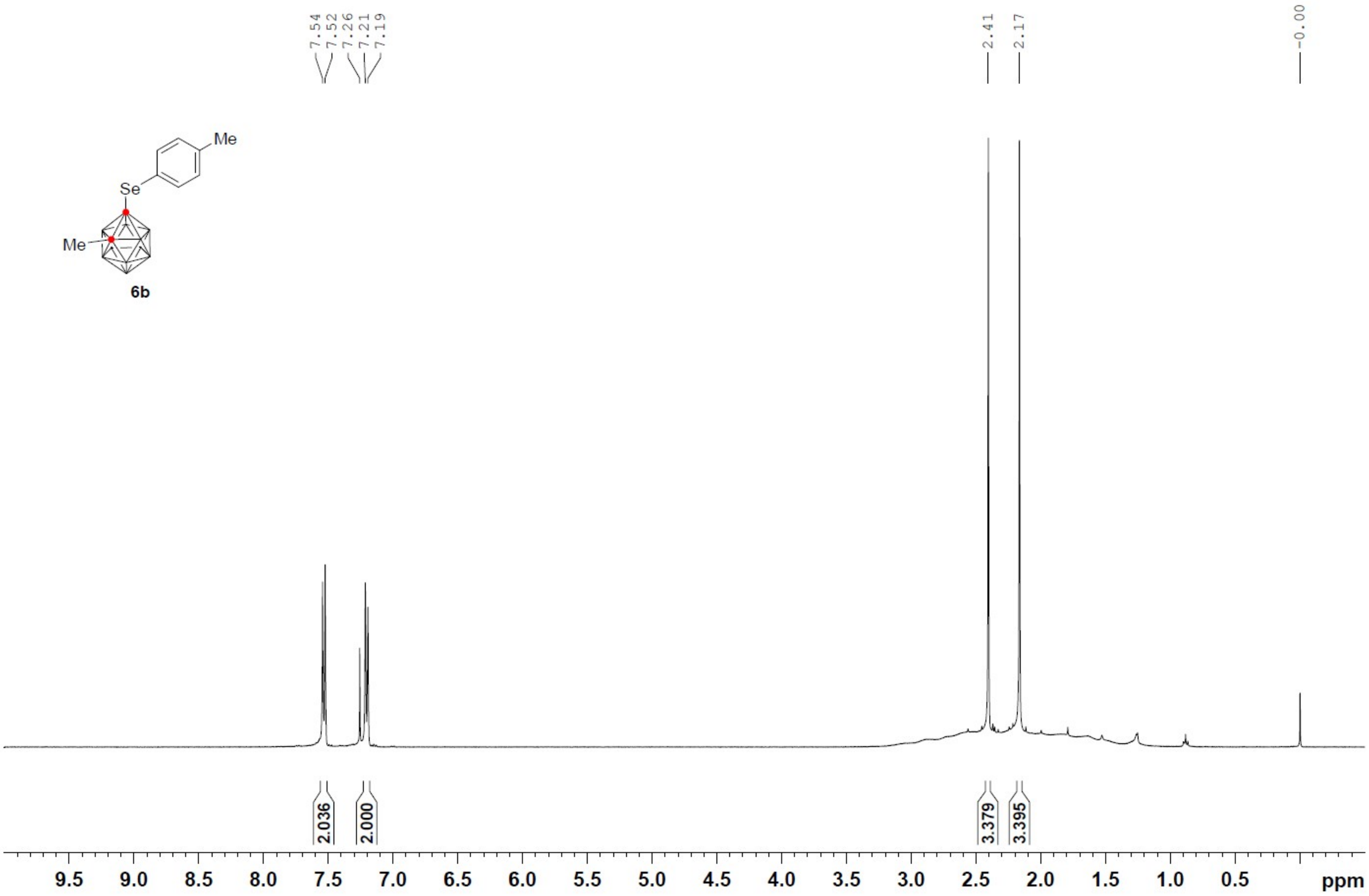

^{13}C NMR (100 MHz, $CDCl_3$)

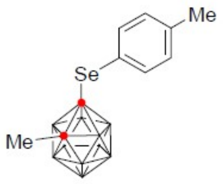

6b

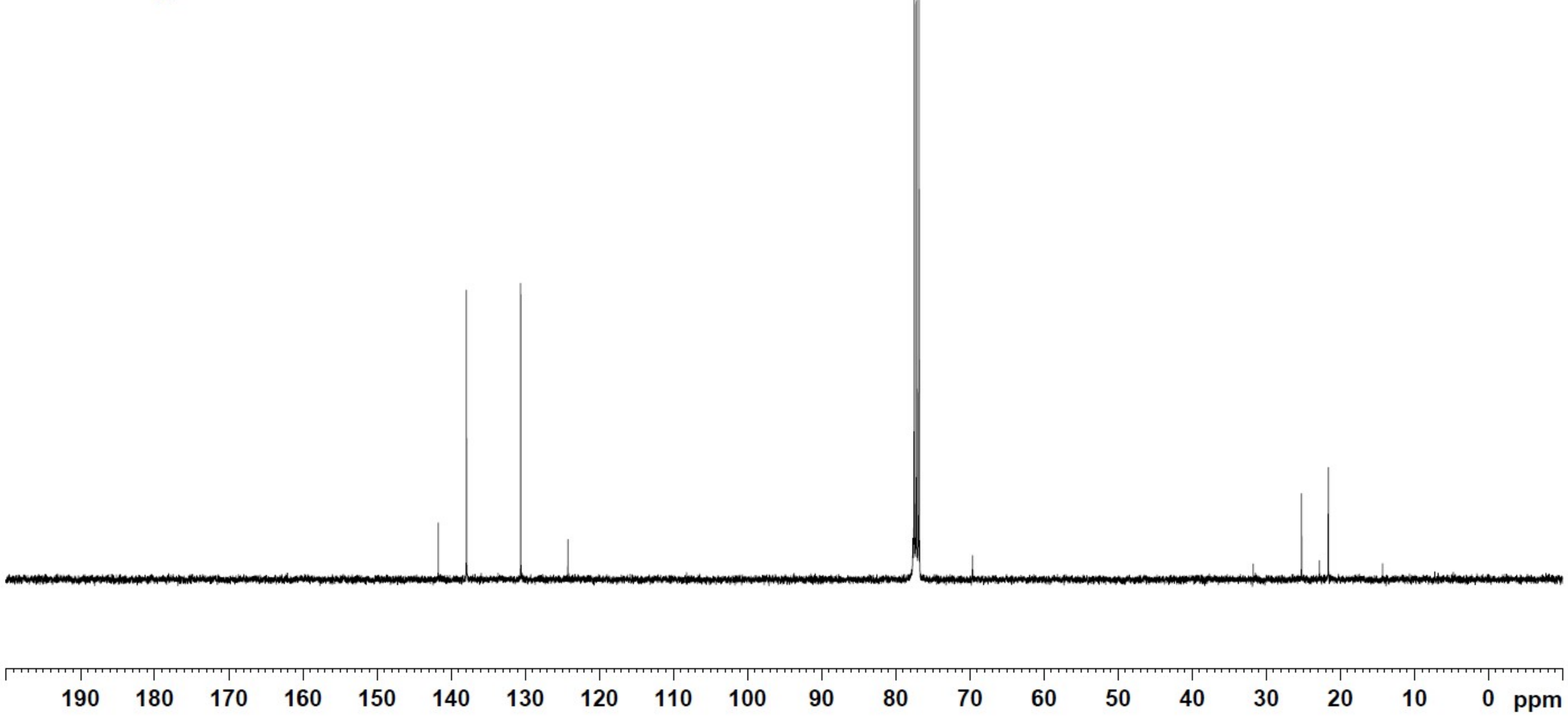

^{11}B NMR (128 MHz, $CDCl_3$)

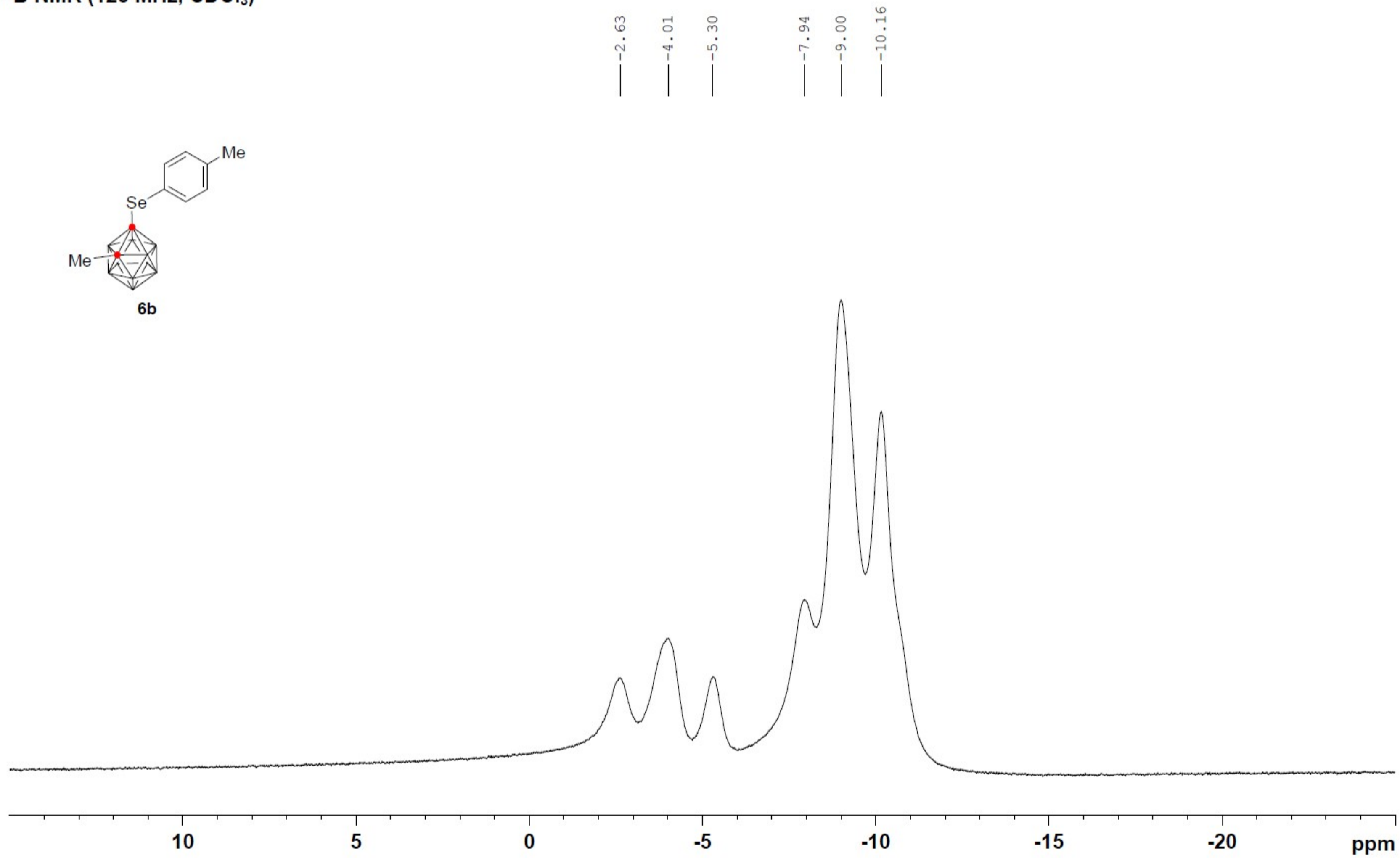

6b

$^{11}B\{^{1}H\}$ NMR (128 MHz, $CDCl_3$)

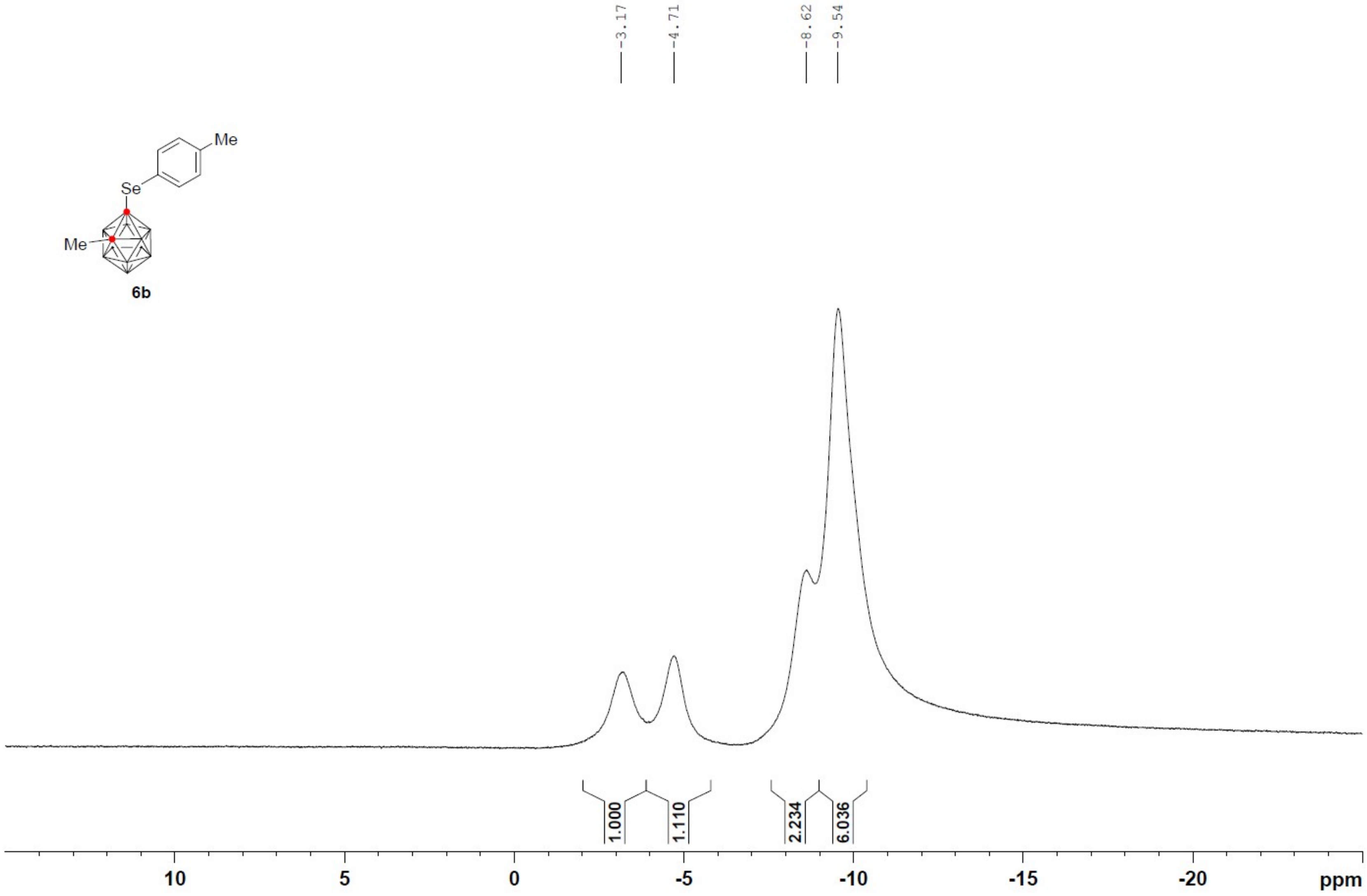

^1H NMR (400 MHz, $CDCl_3$)

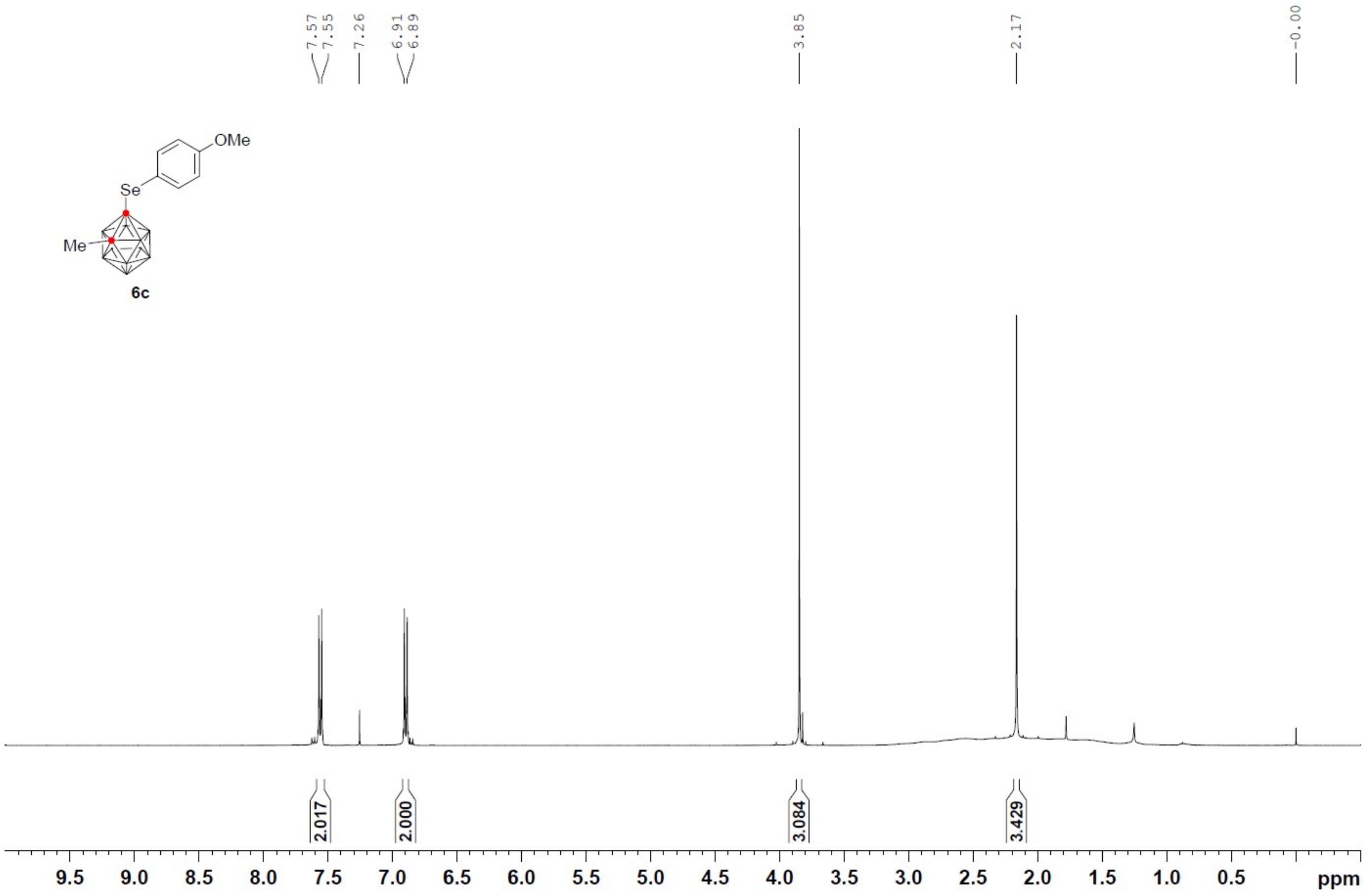

^{13}C NMR (100 MHz, $CDCl_3$)

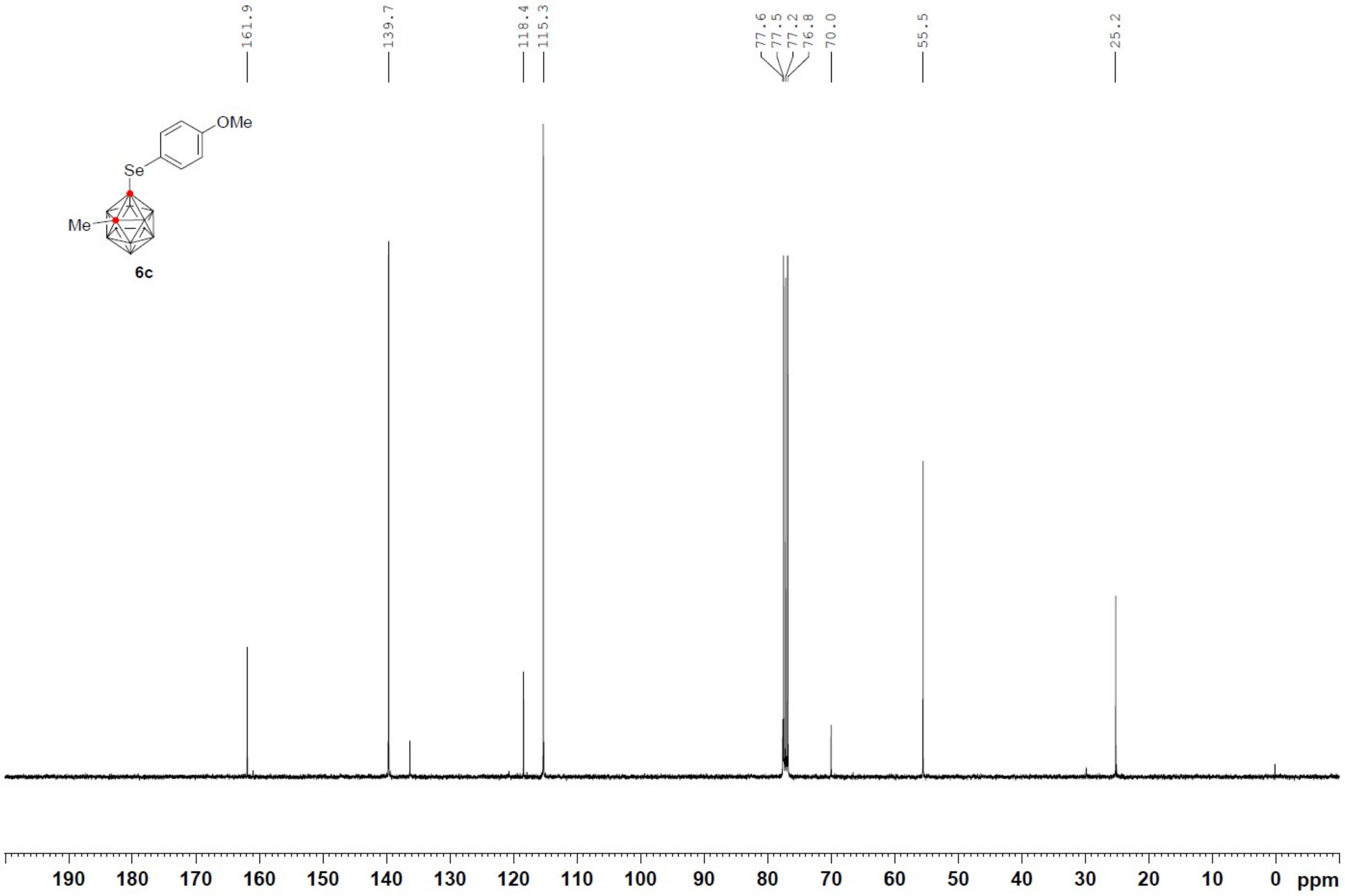

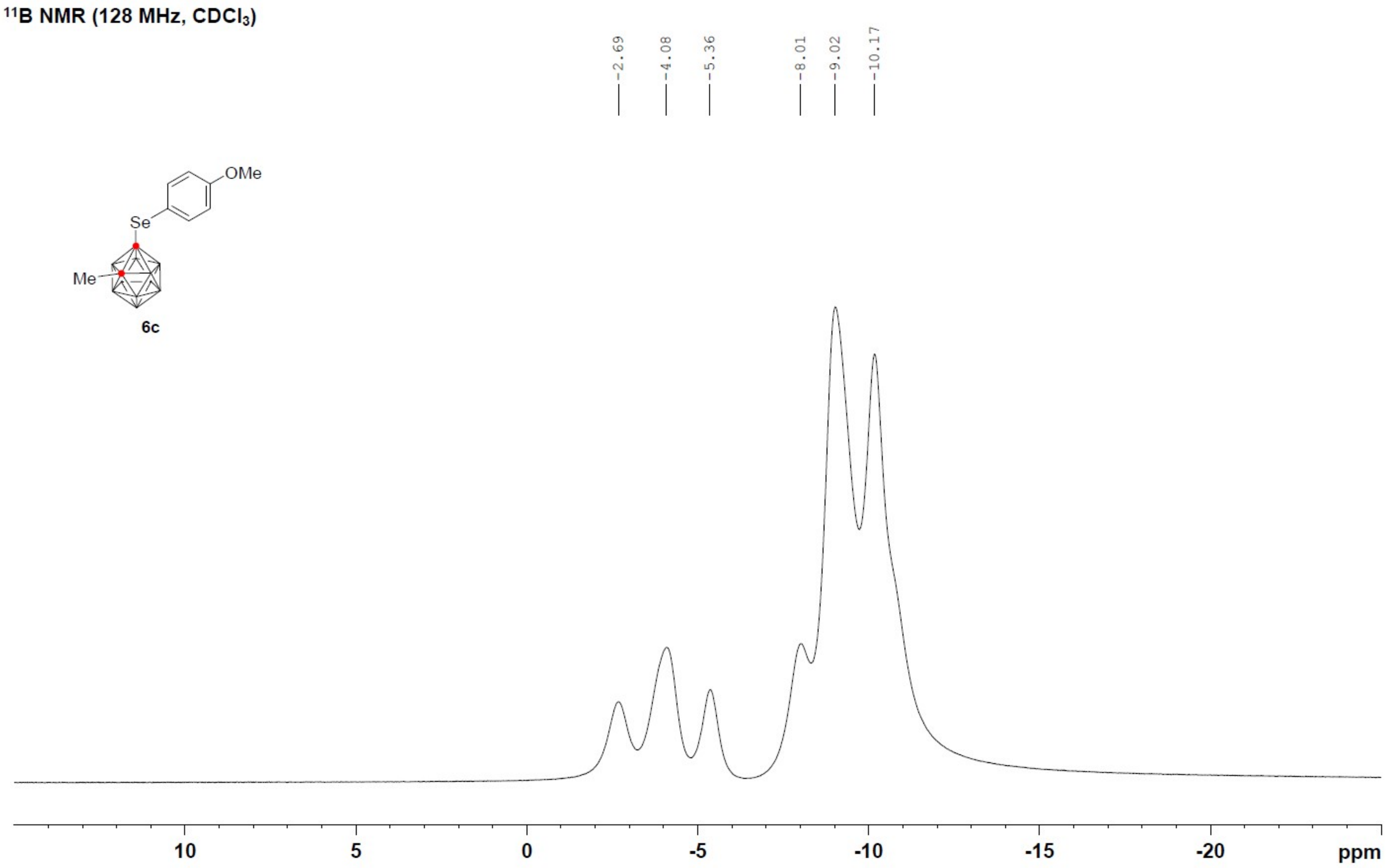

11B NMR (128 MHz, CDCl3)
-2.69
-4.08
-5.36
-8.01
-9.02
-10.17
OMe
Se
Me
6c
10
5
0
-5
-10
-15
-20
ppm

$^{11}B\{^1H\}$ NMR (128 MHz, $CDCl_3$)

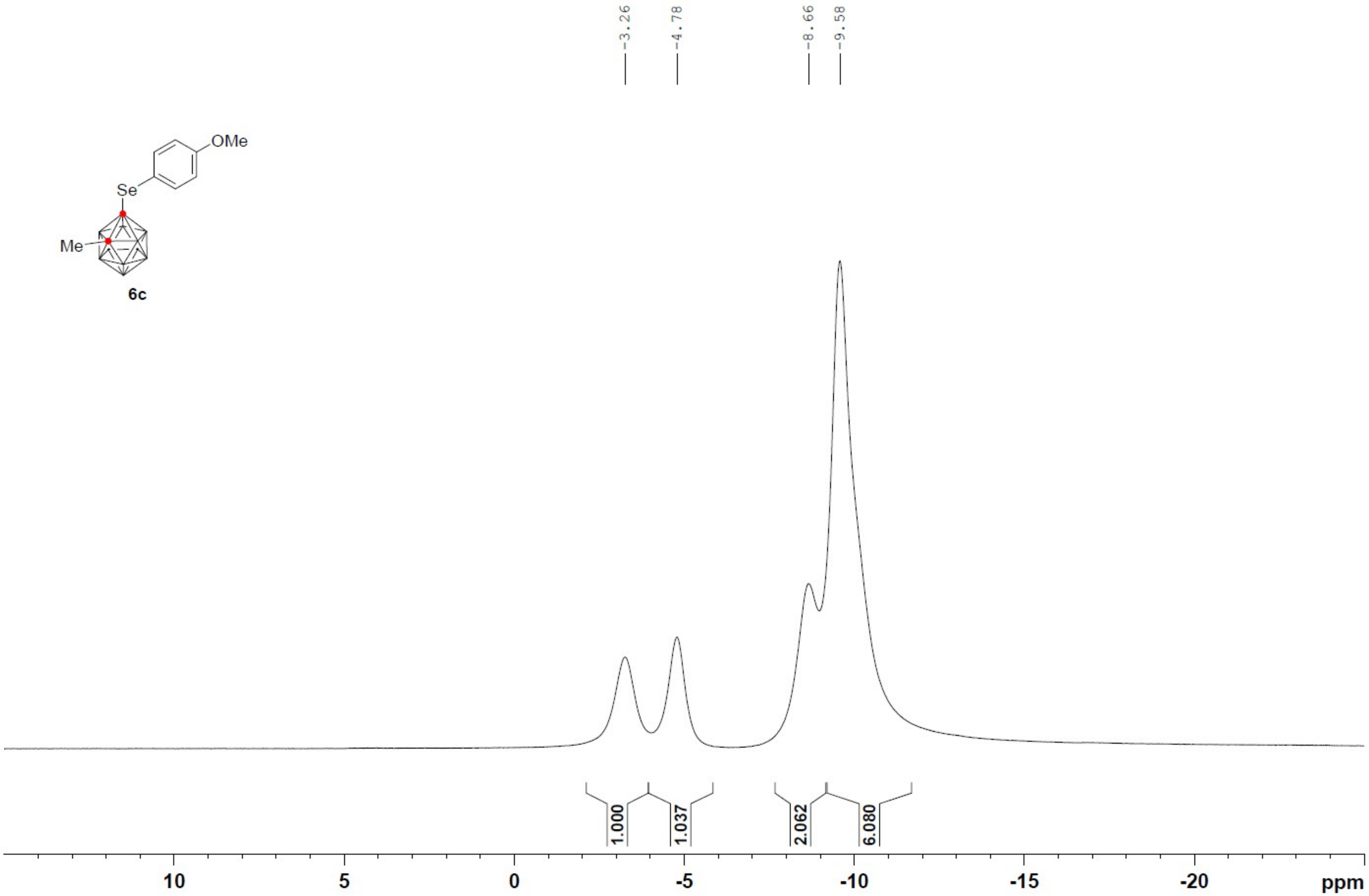

^{1}H NMR (400 MHz, $CDCl_3$)

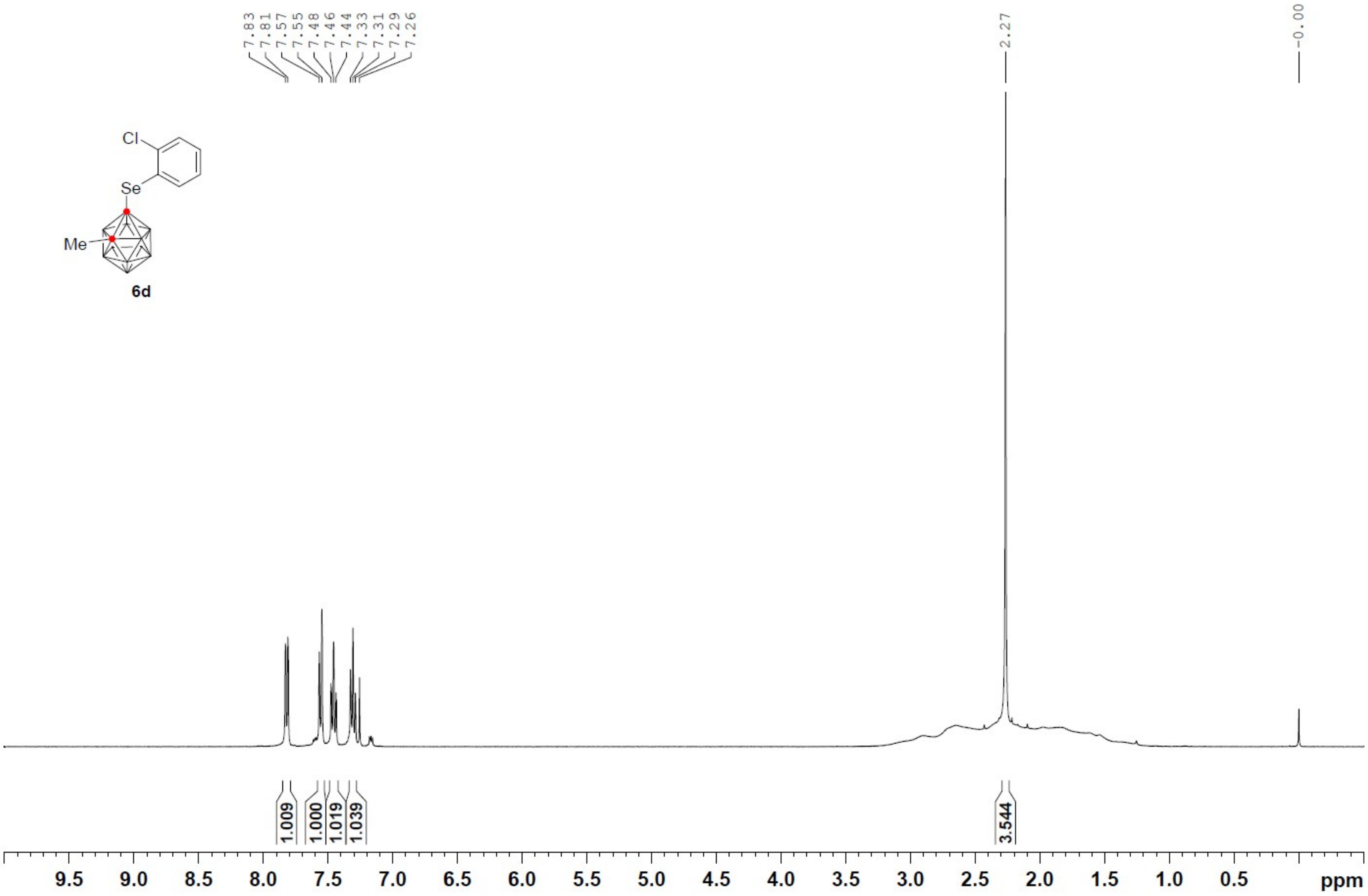

^{13}C NMR (100 MHz, $CDCl_3$)

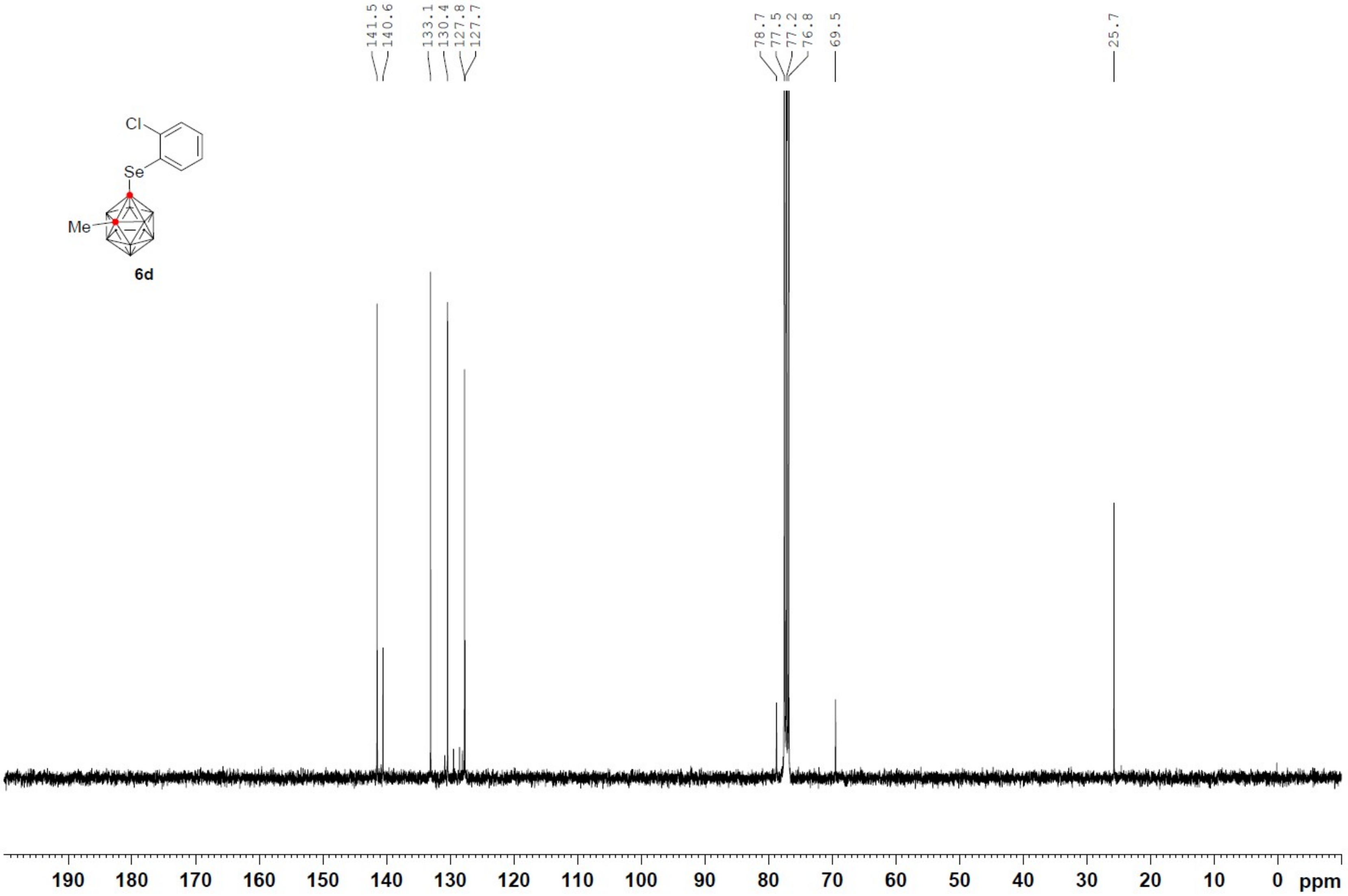

^{11}B NMR (128 MHz, $CDCl_3$)

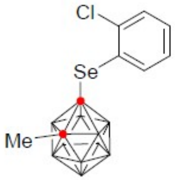

6d

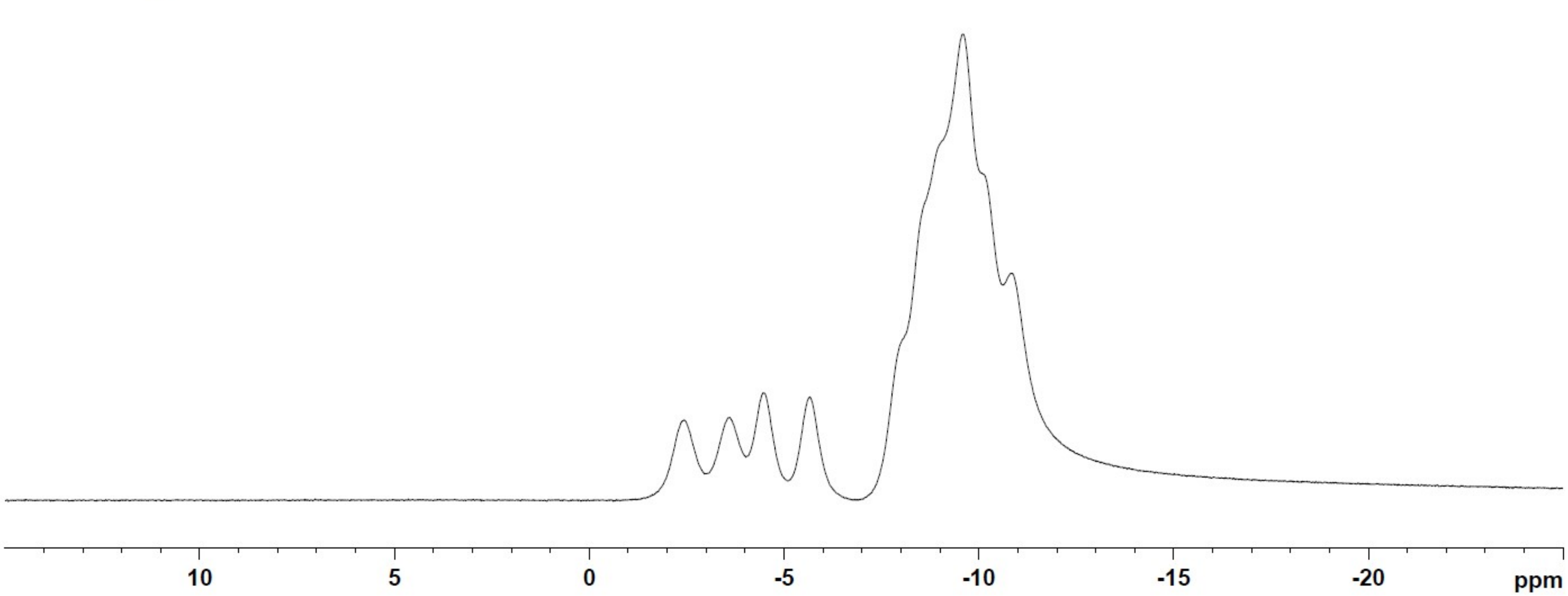

$^{11}B\{^{1}H\}$ NMR (128 MHz, $CDCl_3$)

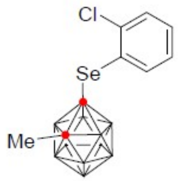

6d

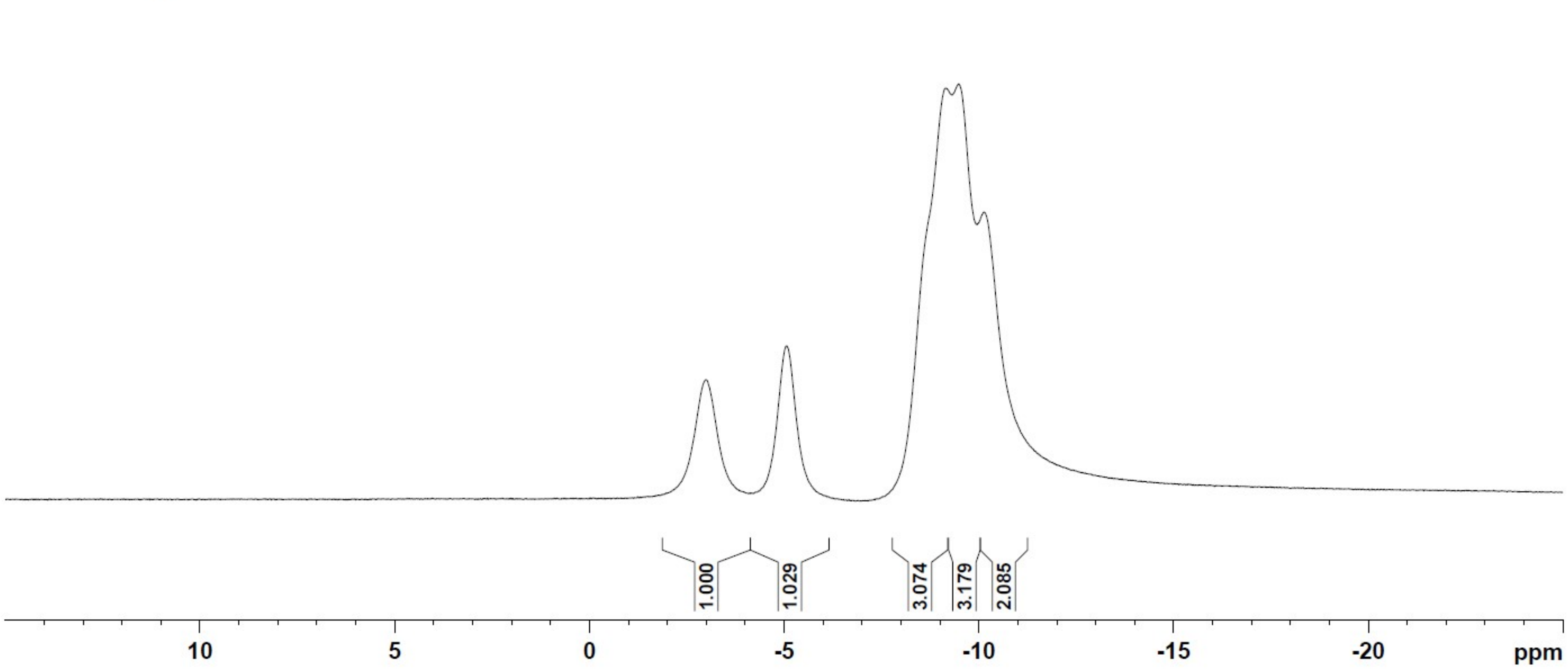

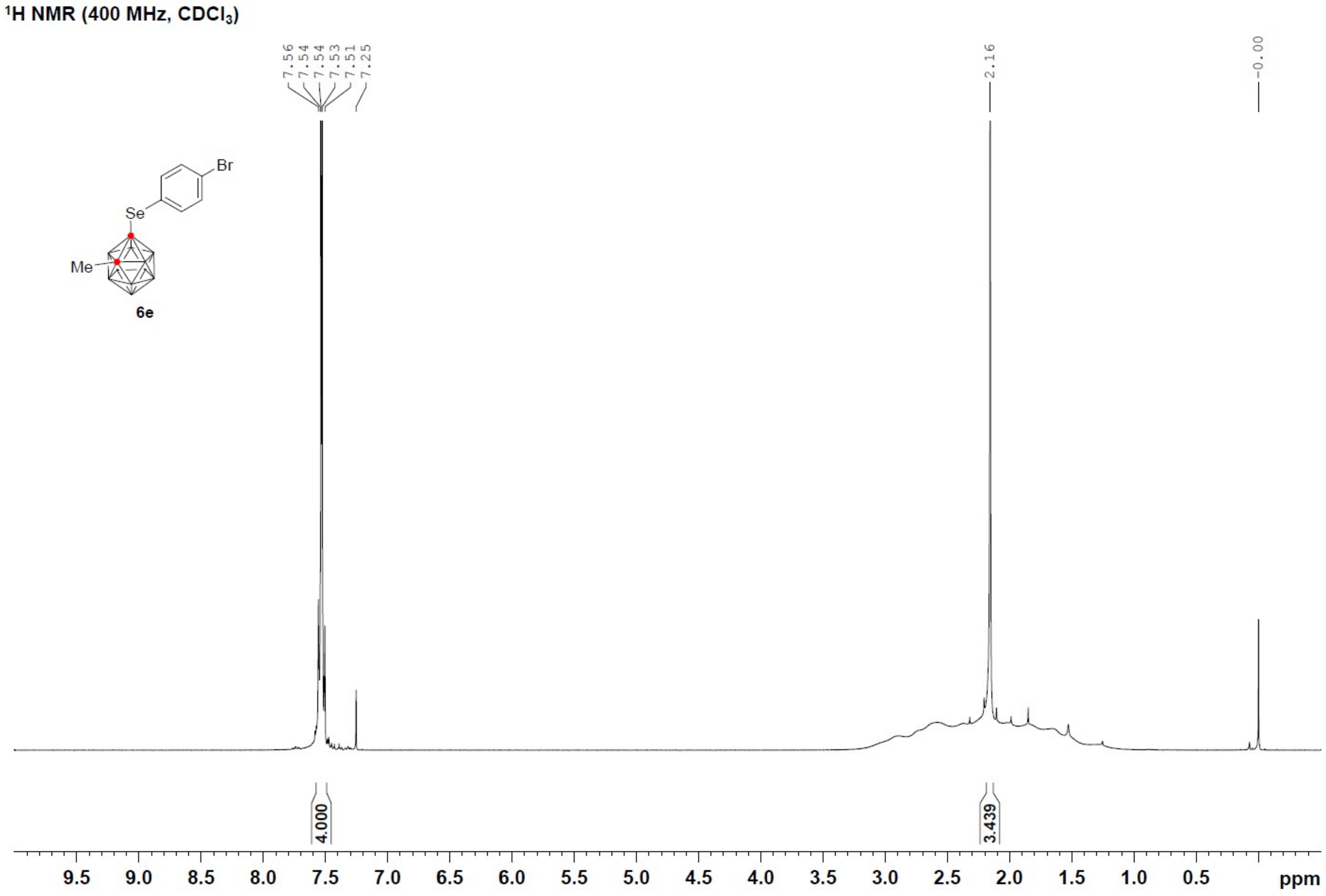

1H NMR (400 MHz, CDCl3)
7.56
7.54
7.54
7.53
7.51
7.25
2.16
-0.00
Br
Se
Me
6e
4.000
3.439
9.5
9.0
8.5
8.0
7.5
7.0
6.5
6.0
5.5
5.0
4.5
4.0
3.5
3.0
2.5
2.0
1.5
1.0
0.5
ppm

^{13}C NMR (100 MHz, $CDCl_3$)

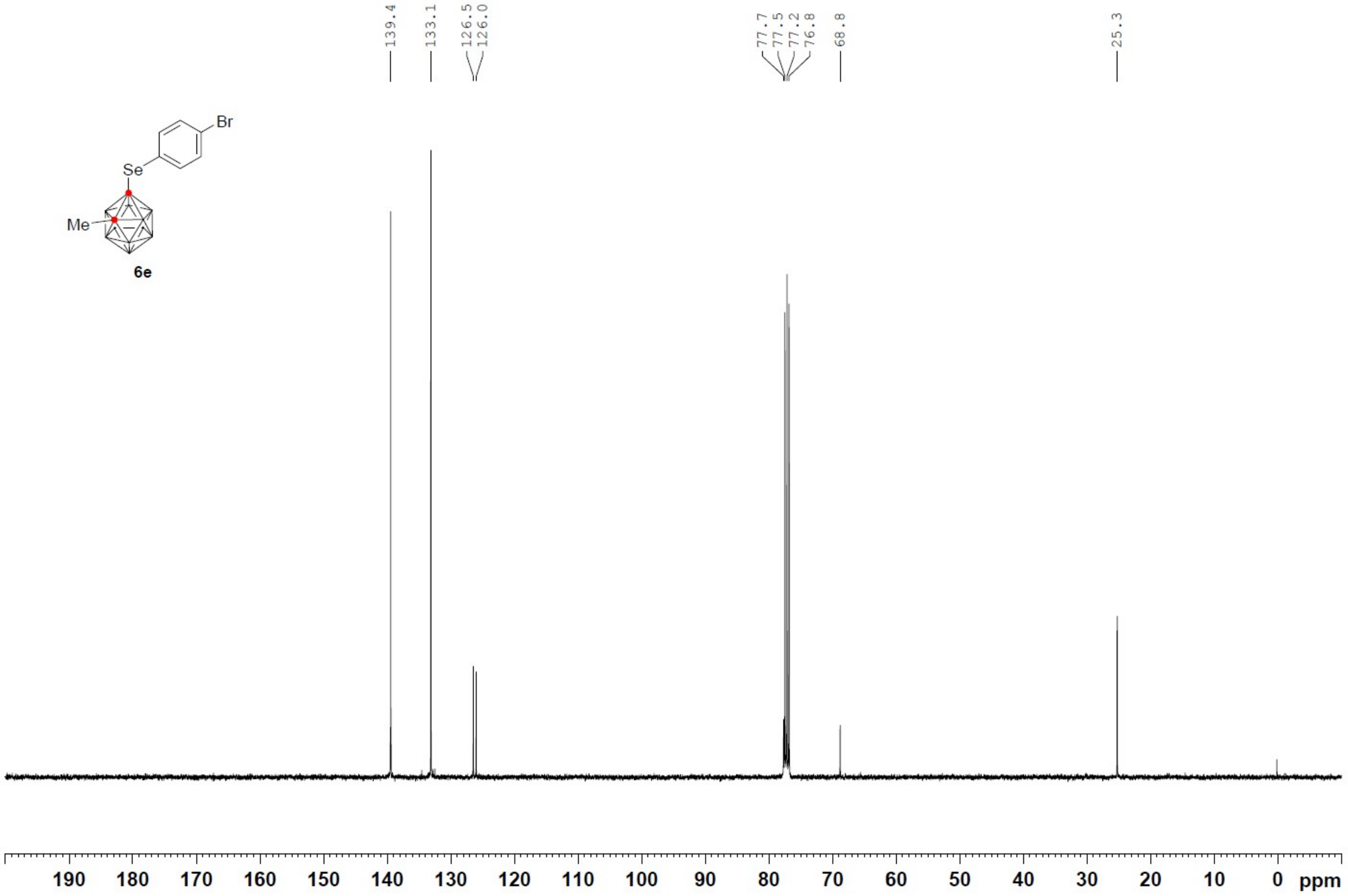

^{11}B NMR (128 MHz, $CDCl_3$)

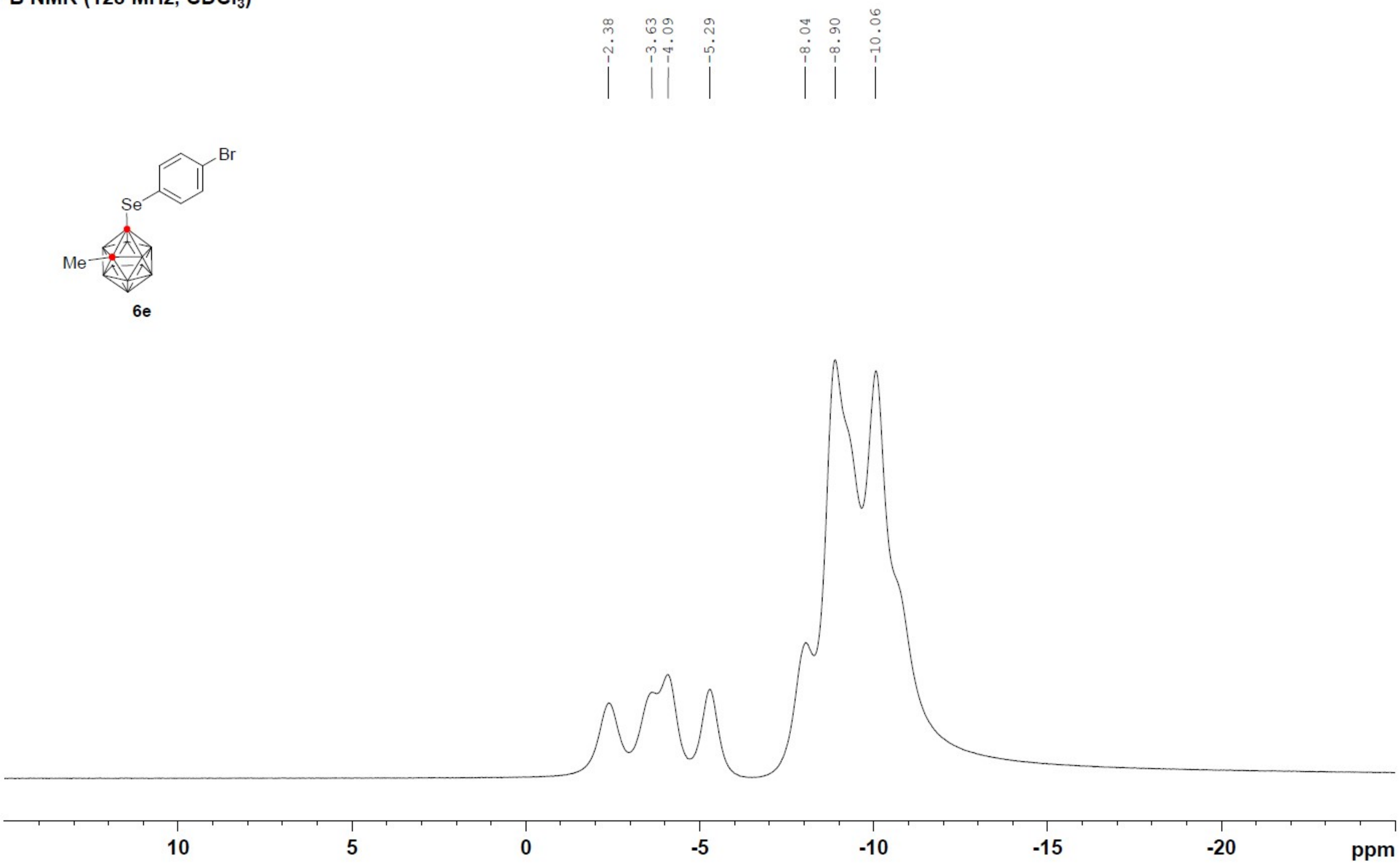

$^{11}B\{^{1}H\}$ NMR (128 MHz, $CDCl_3$)

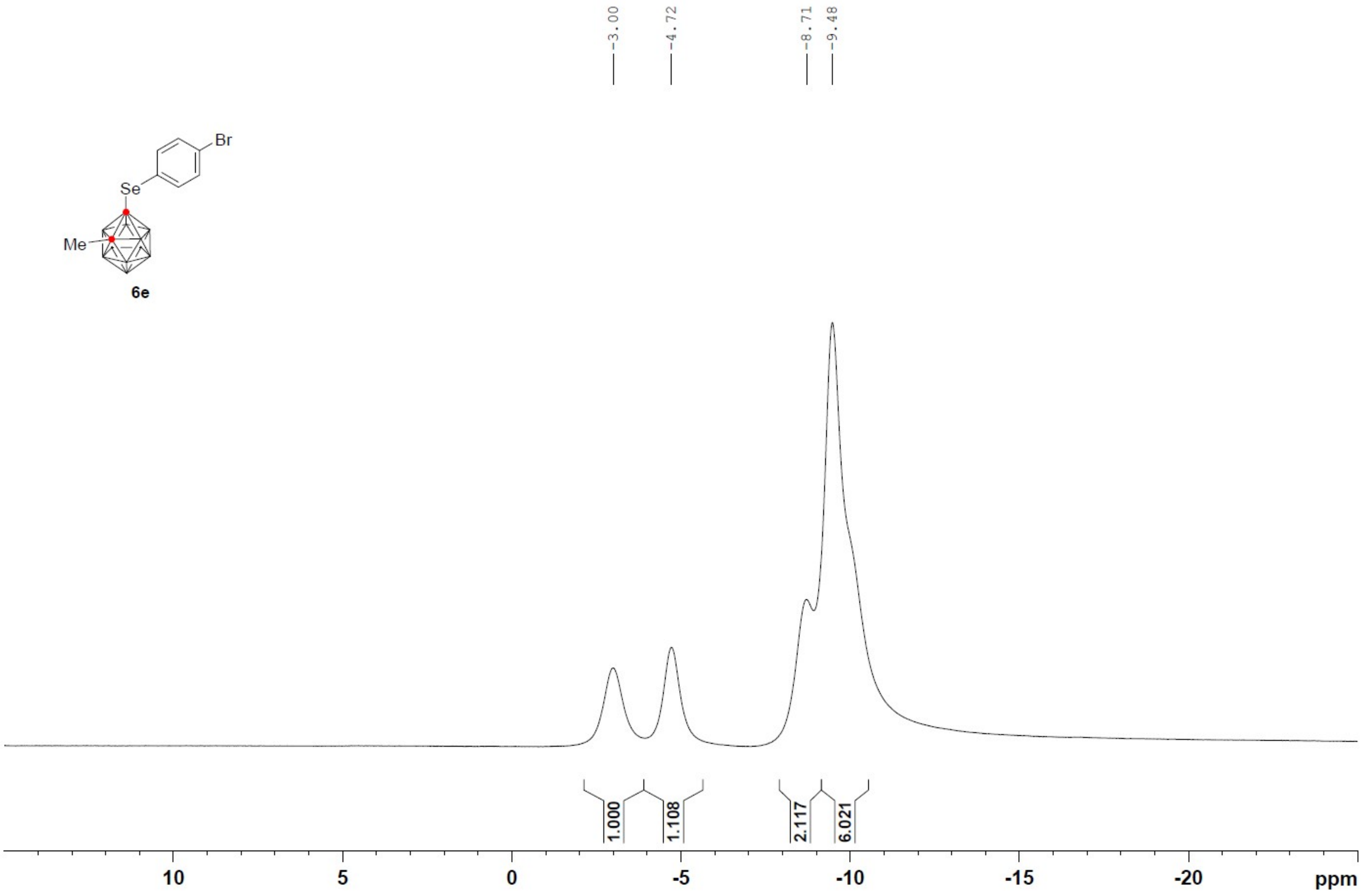

^{1}H NMR (400 MHz, $CDCl_3$)

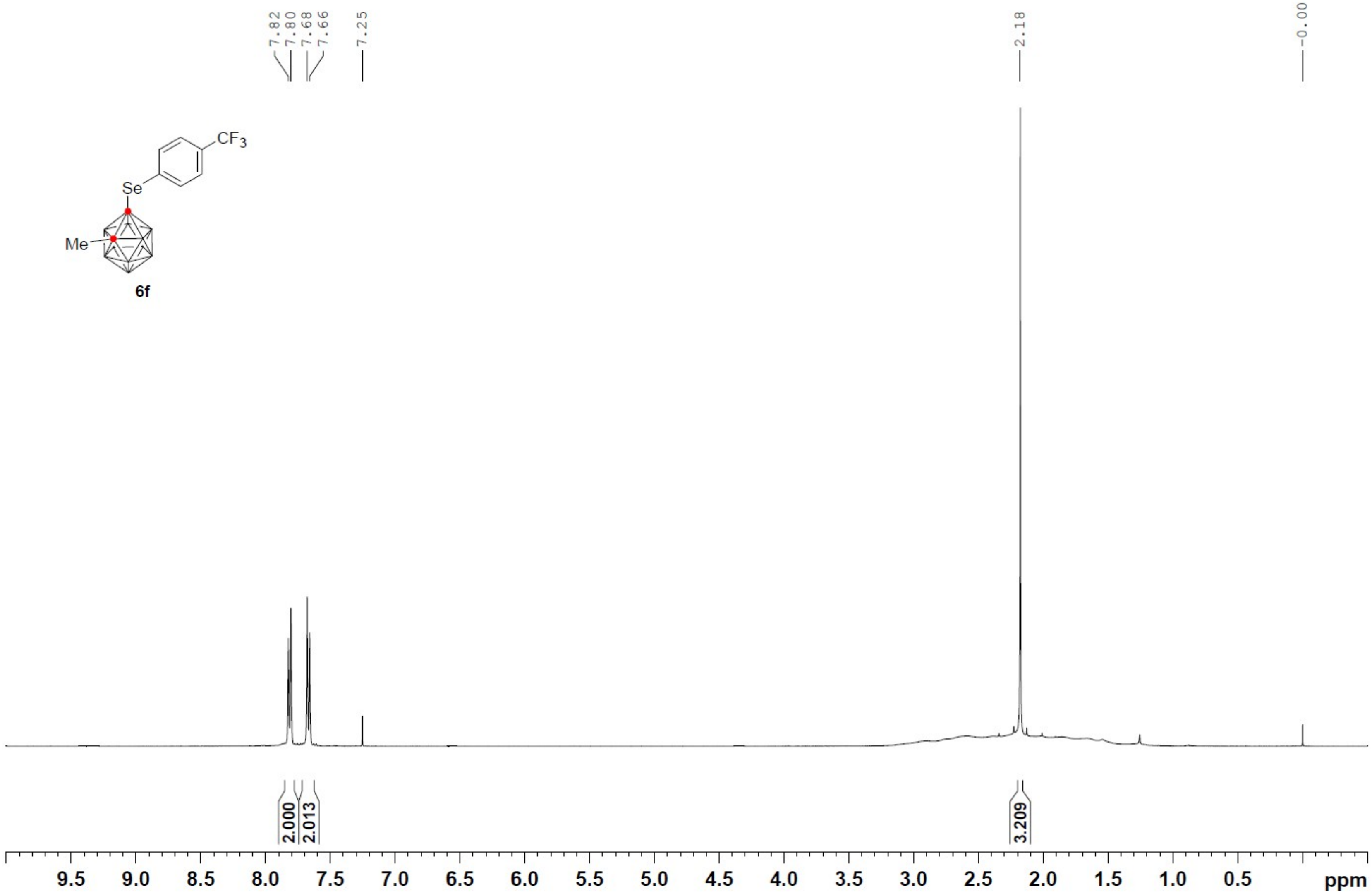

^{13}C NMR (100 MHz, $CDCl_3$)

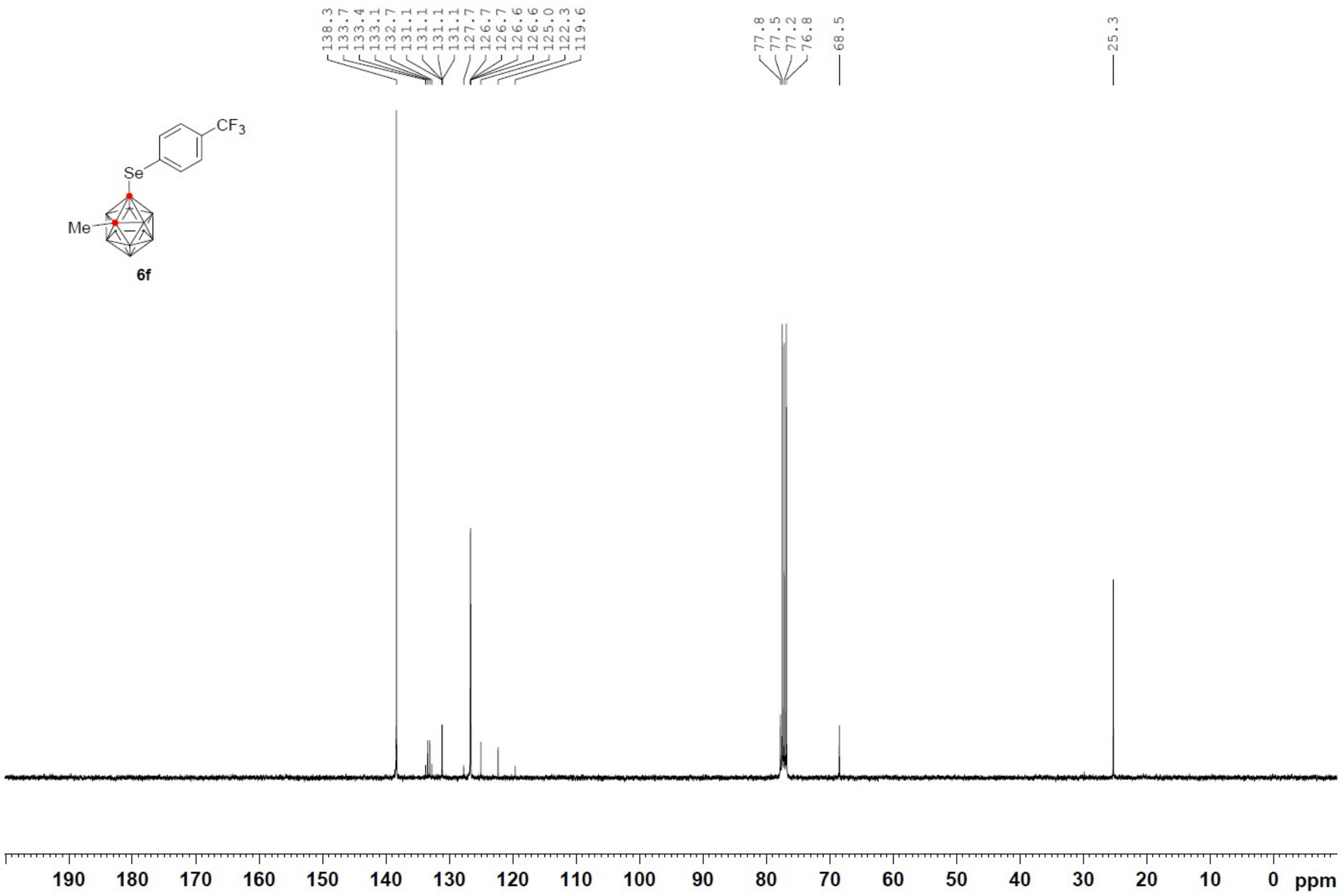

^{11}B NMR (128 MHz, $CDCl_3$)

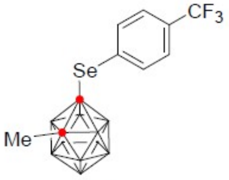

6f

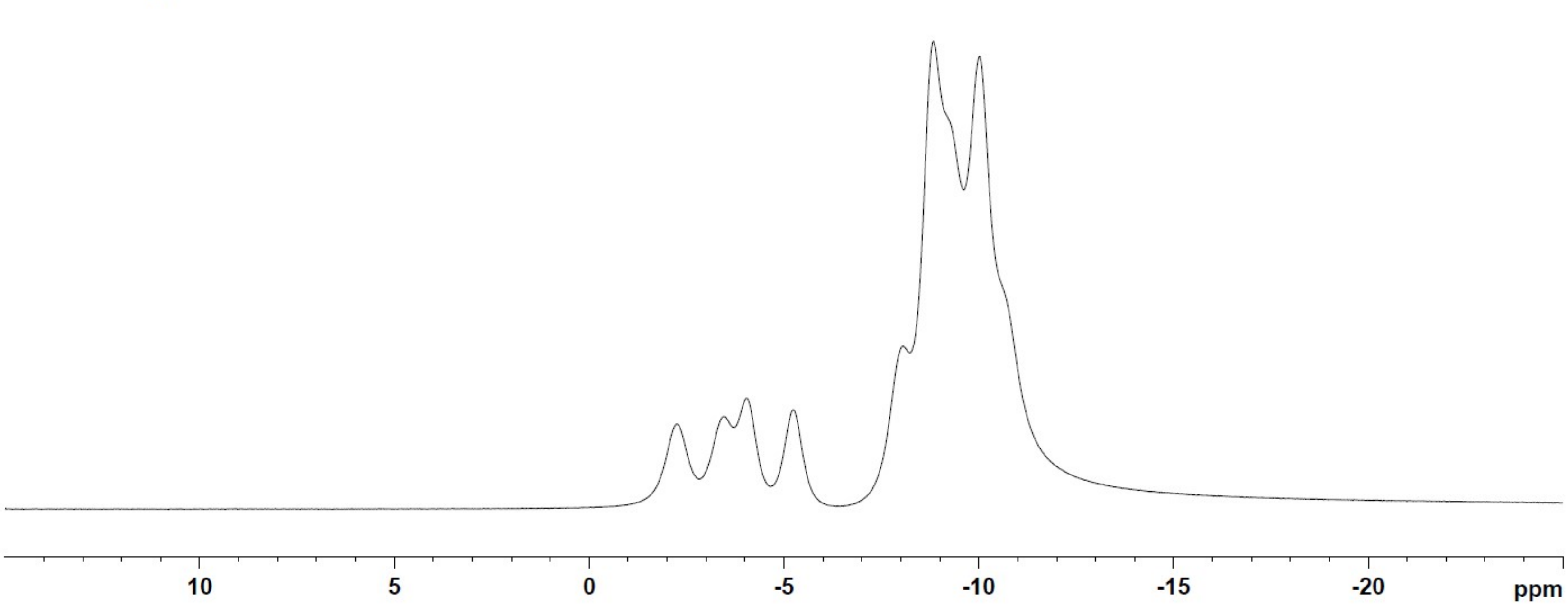

$^{11}B\{^1H\}$ NMR (128 MHz, $CDCl_3$)

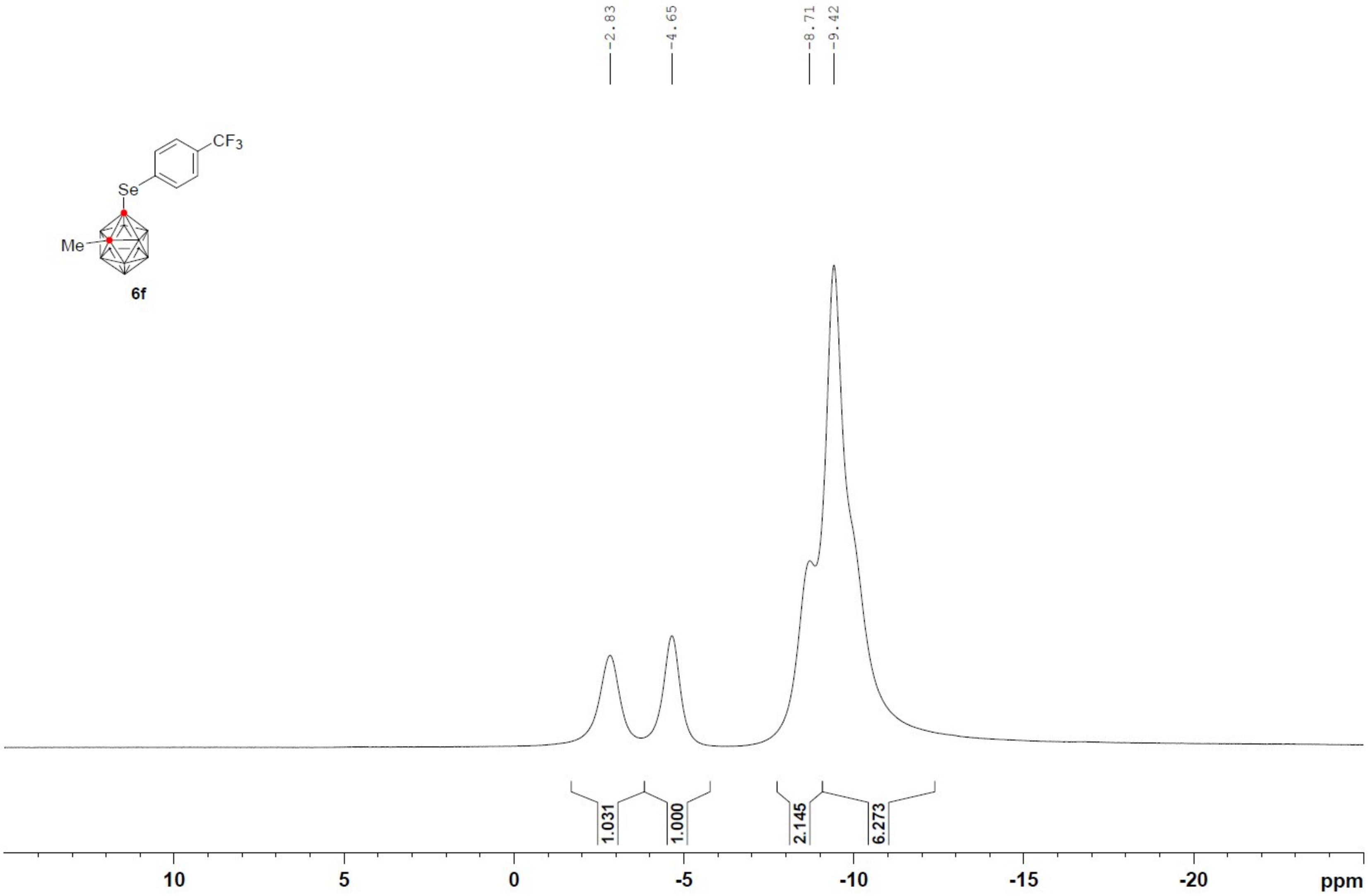

$^{19}F\{^1H\}$ NMR (377 MHz, $CDCl_3$)

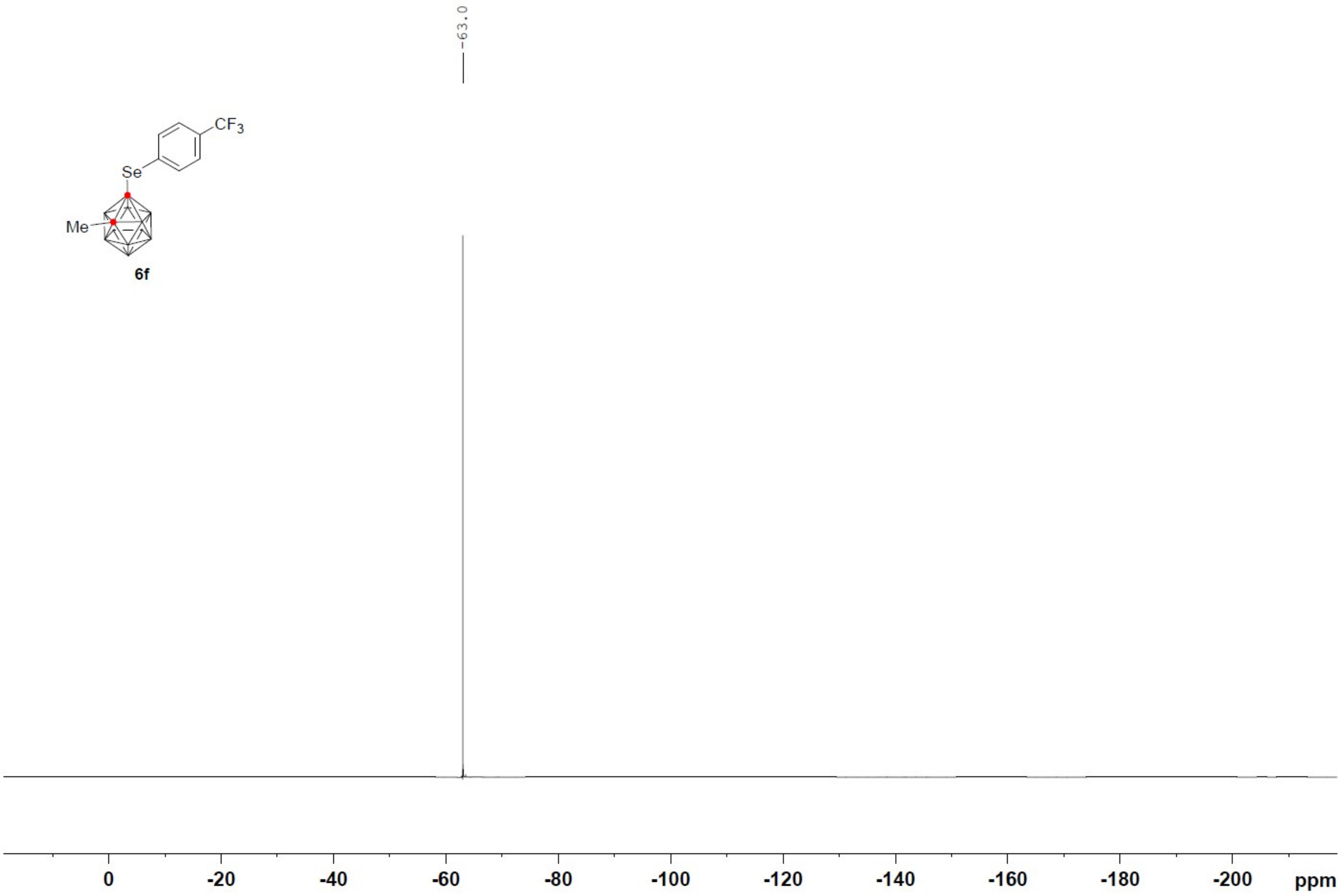

^{1}H NMR (400 MHz, $CDCl_3$)

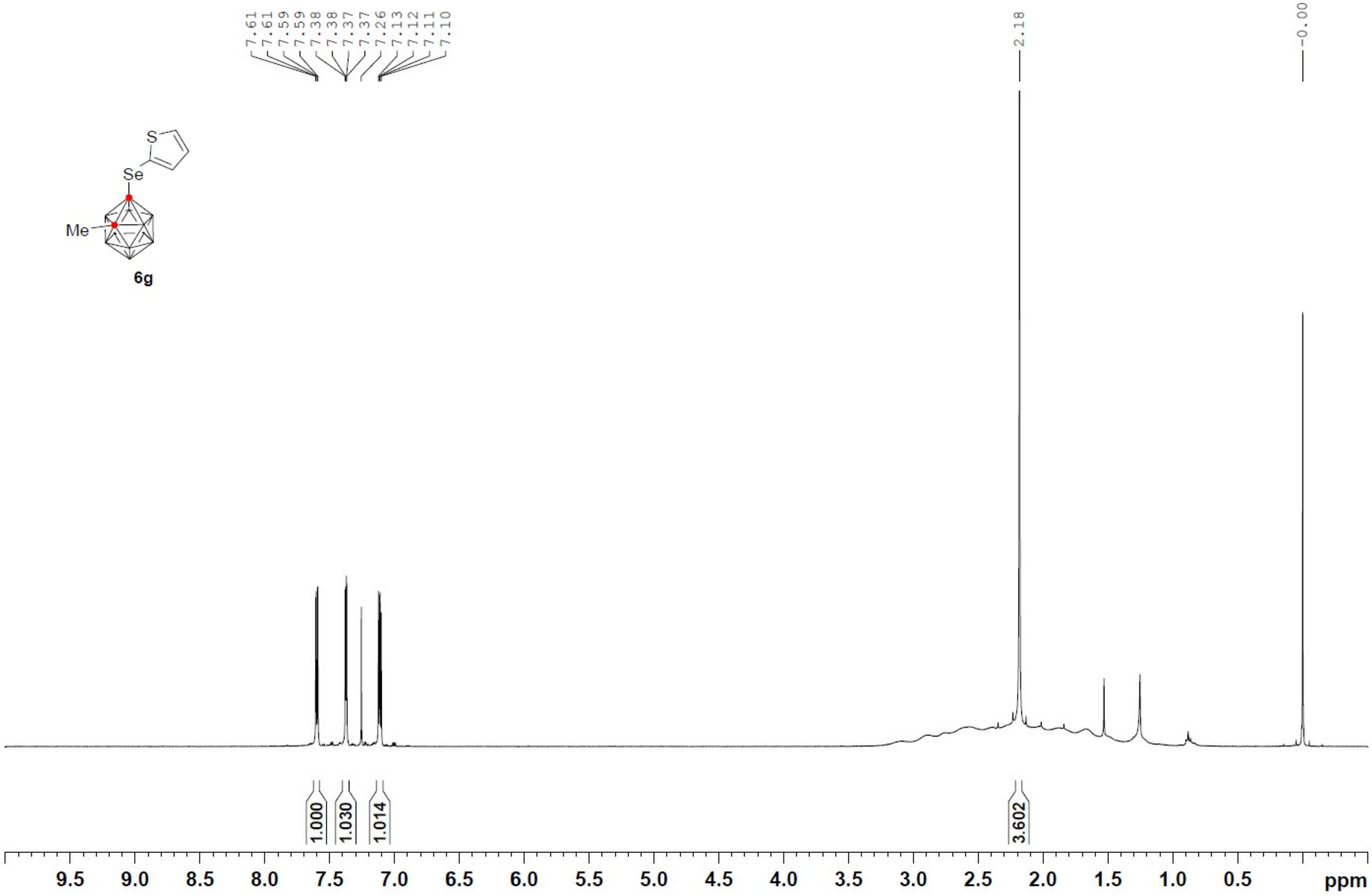

^{13}C NMR (100 MHz, $CDCl_3$)

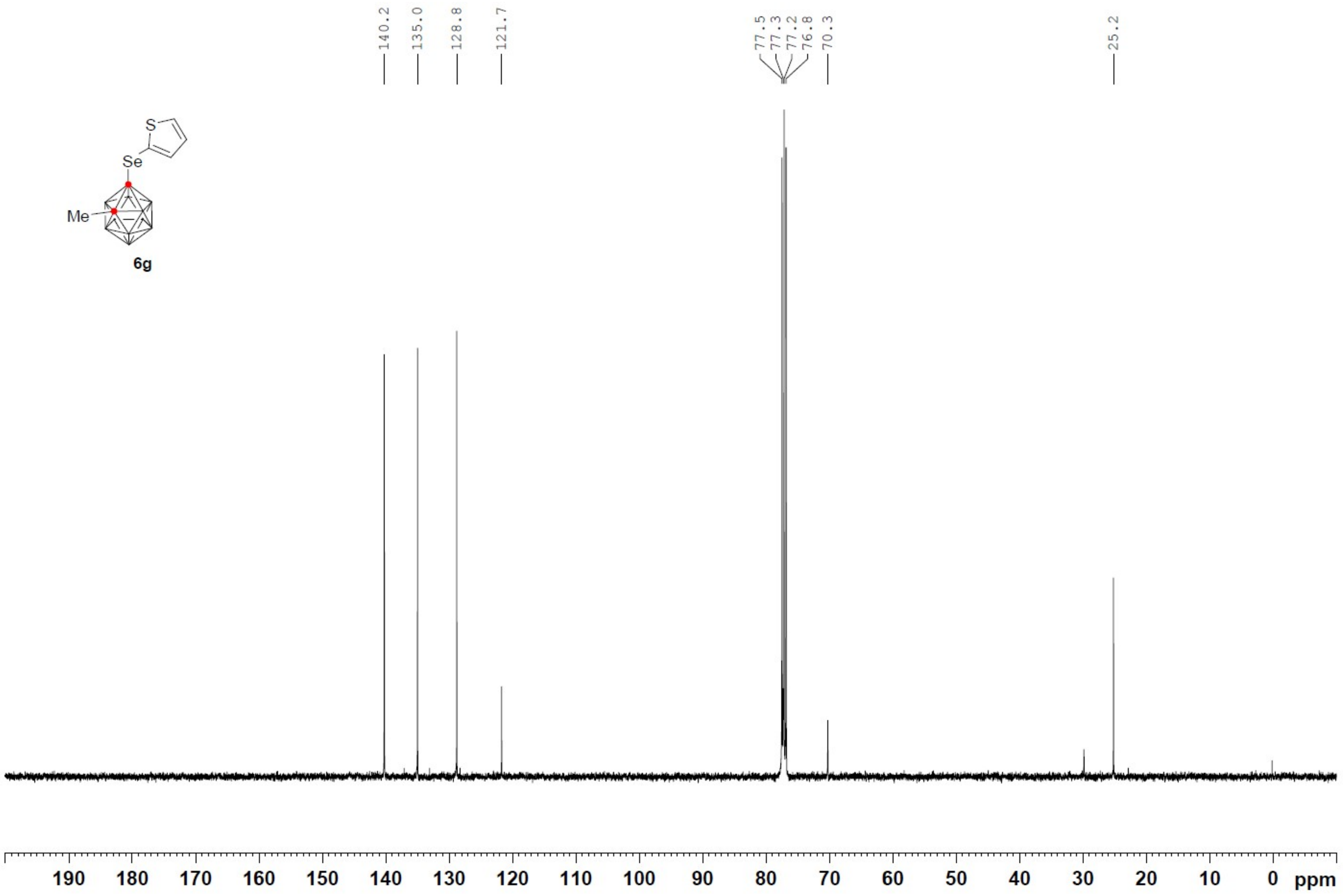

^{11}B NMR (128 MHz, $CDCl_3$)

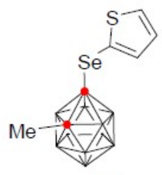

6g

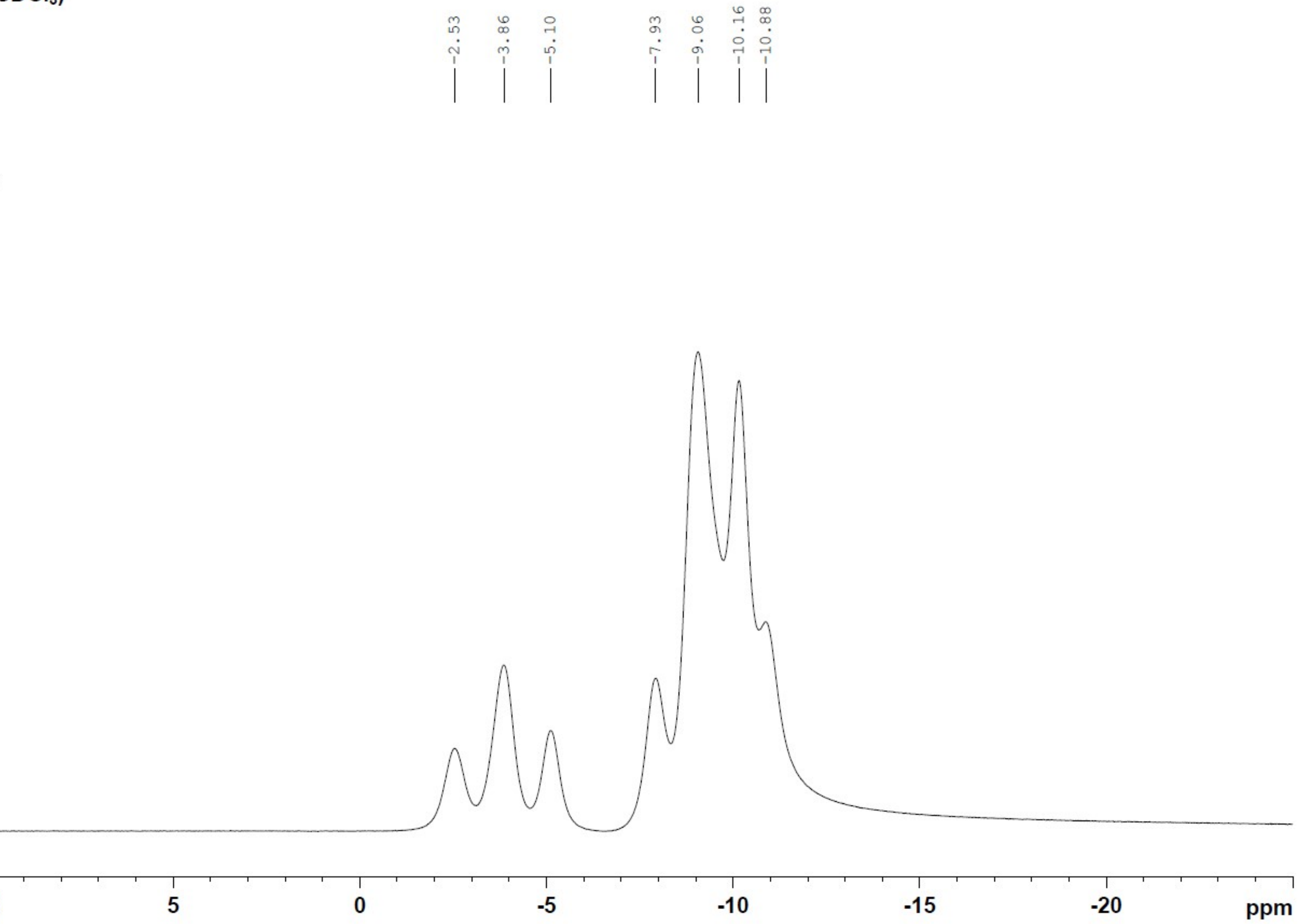

$^{11}B\{^{1}H\}$ NMR (128 MHz, $CDCl_3$)

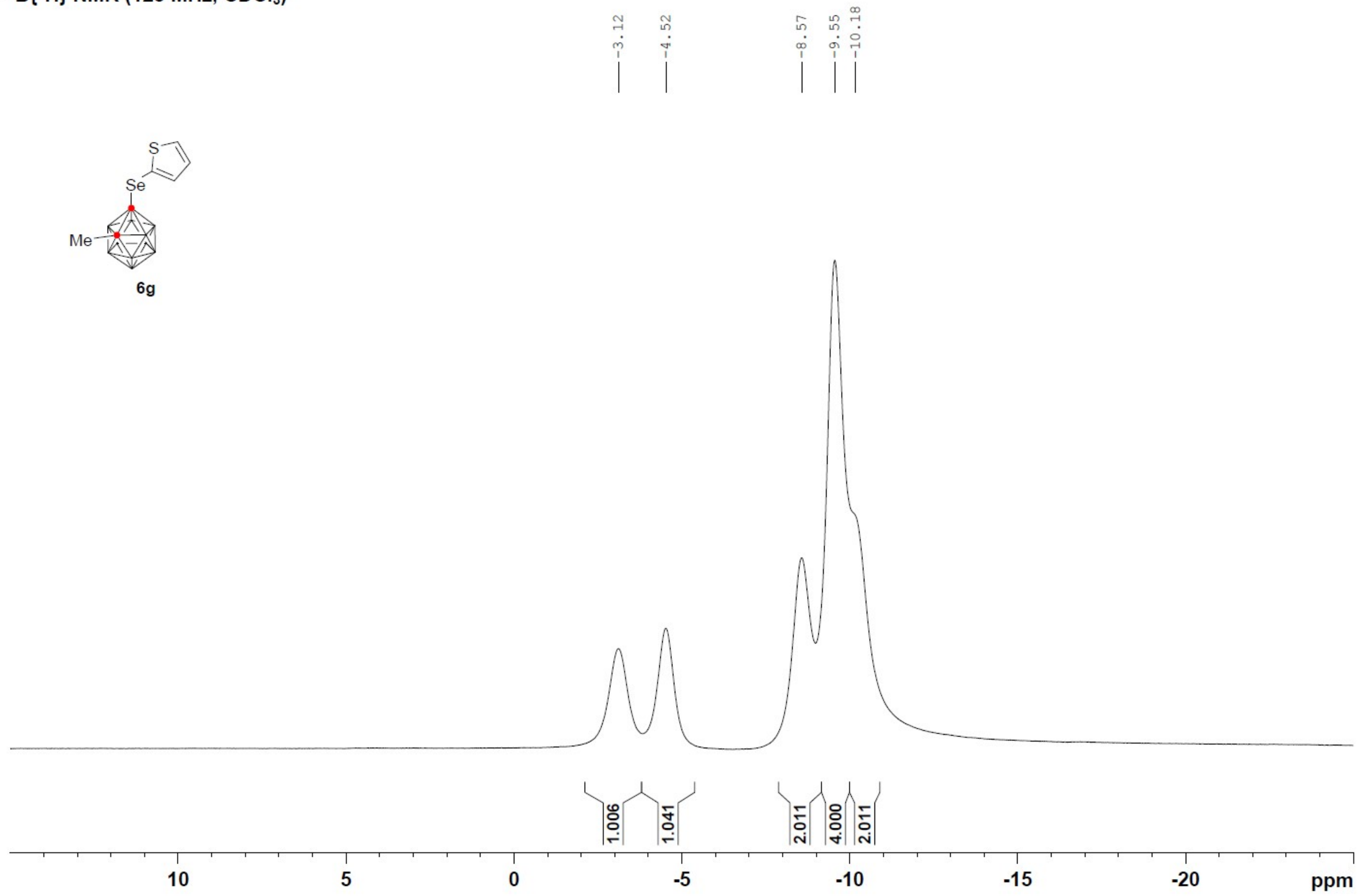

^{1}H NMR (400 MHz, $CDCl_3$)

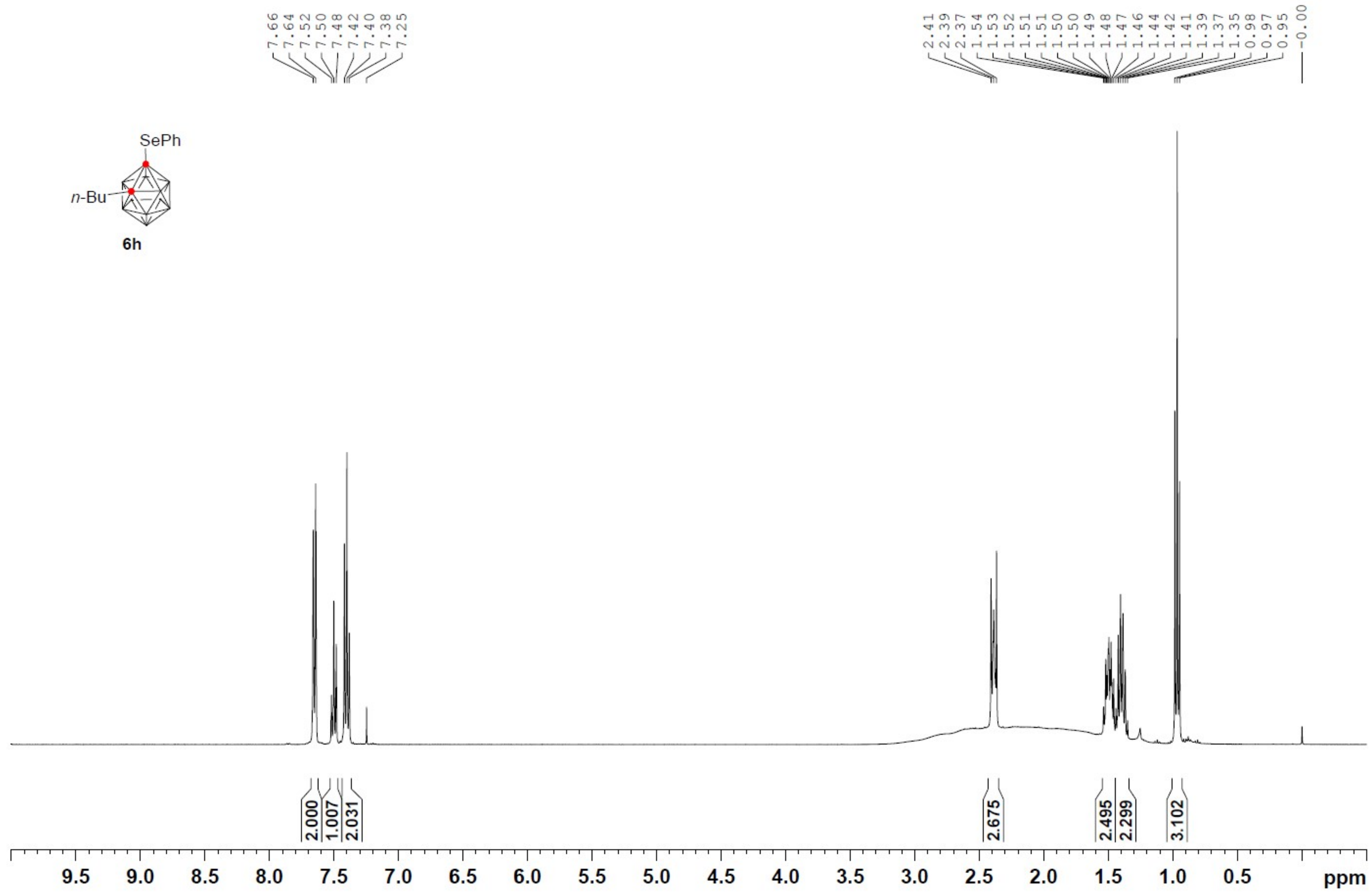

^{13}C NMR (100 MHz, $CDCl_3$)

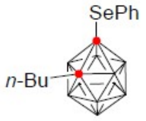

6h

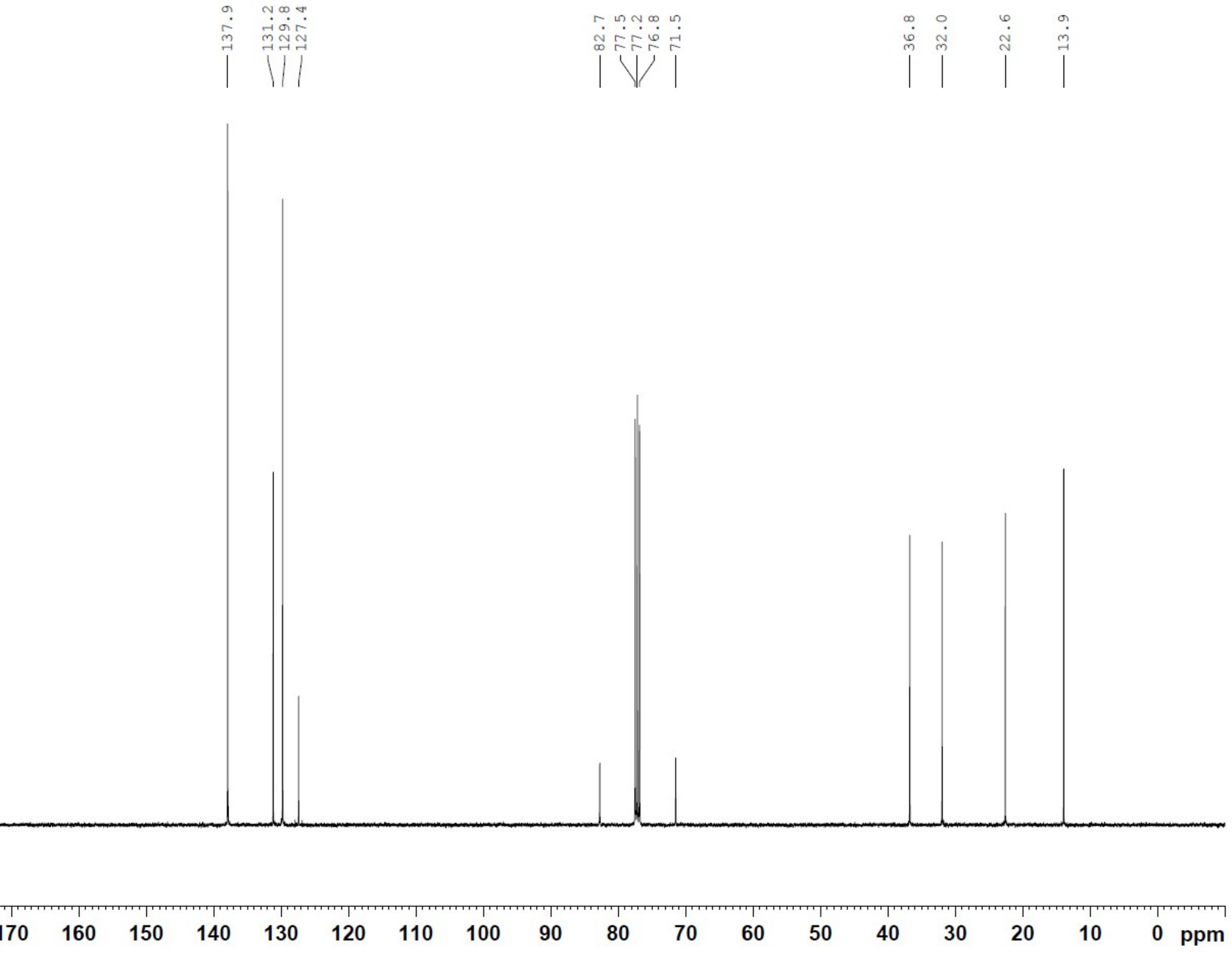

^{11}B NMR (128 MHz, $CDCl_3$)

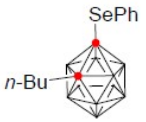

6h

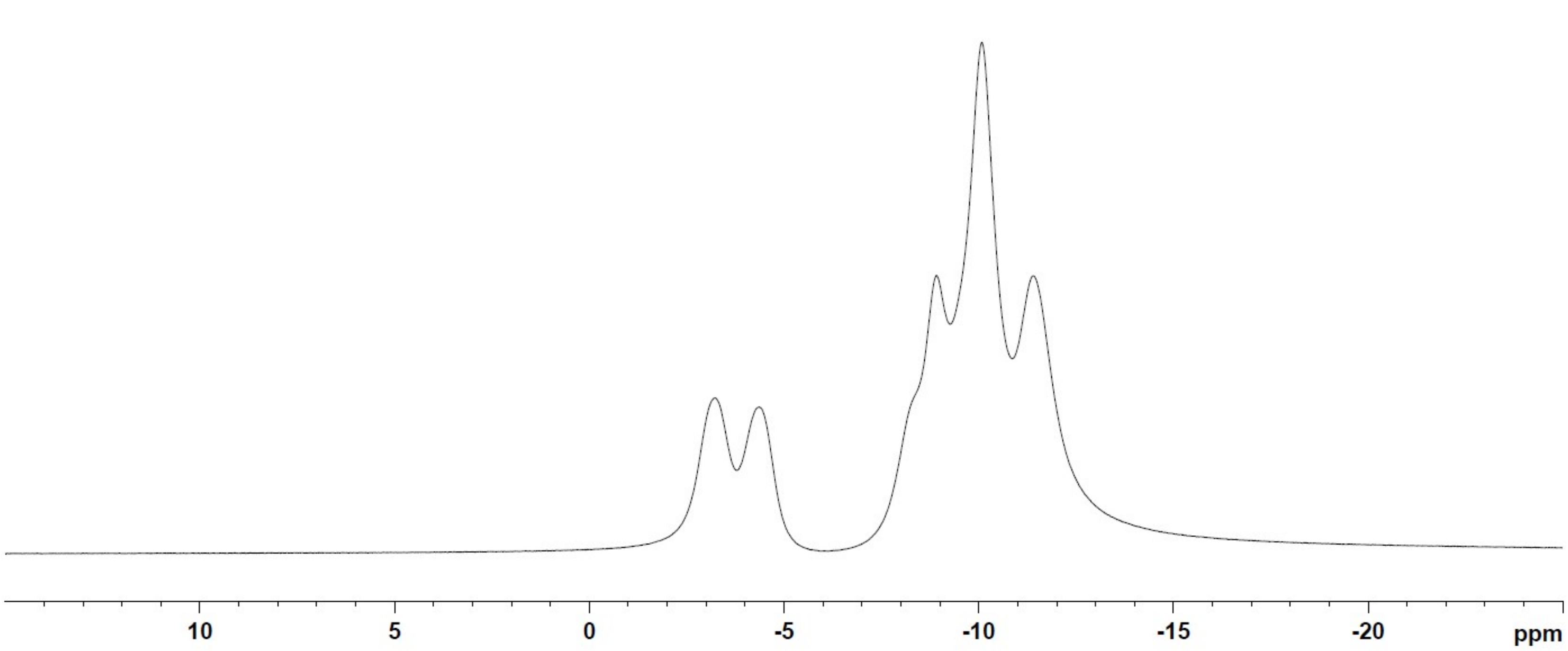

$^{11}B\{^{1}H\}$ NMR (128 MHz, $CDCl_3$)

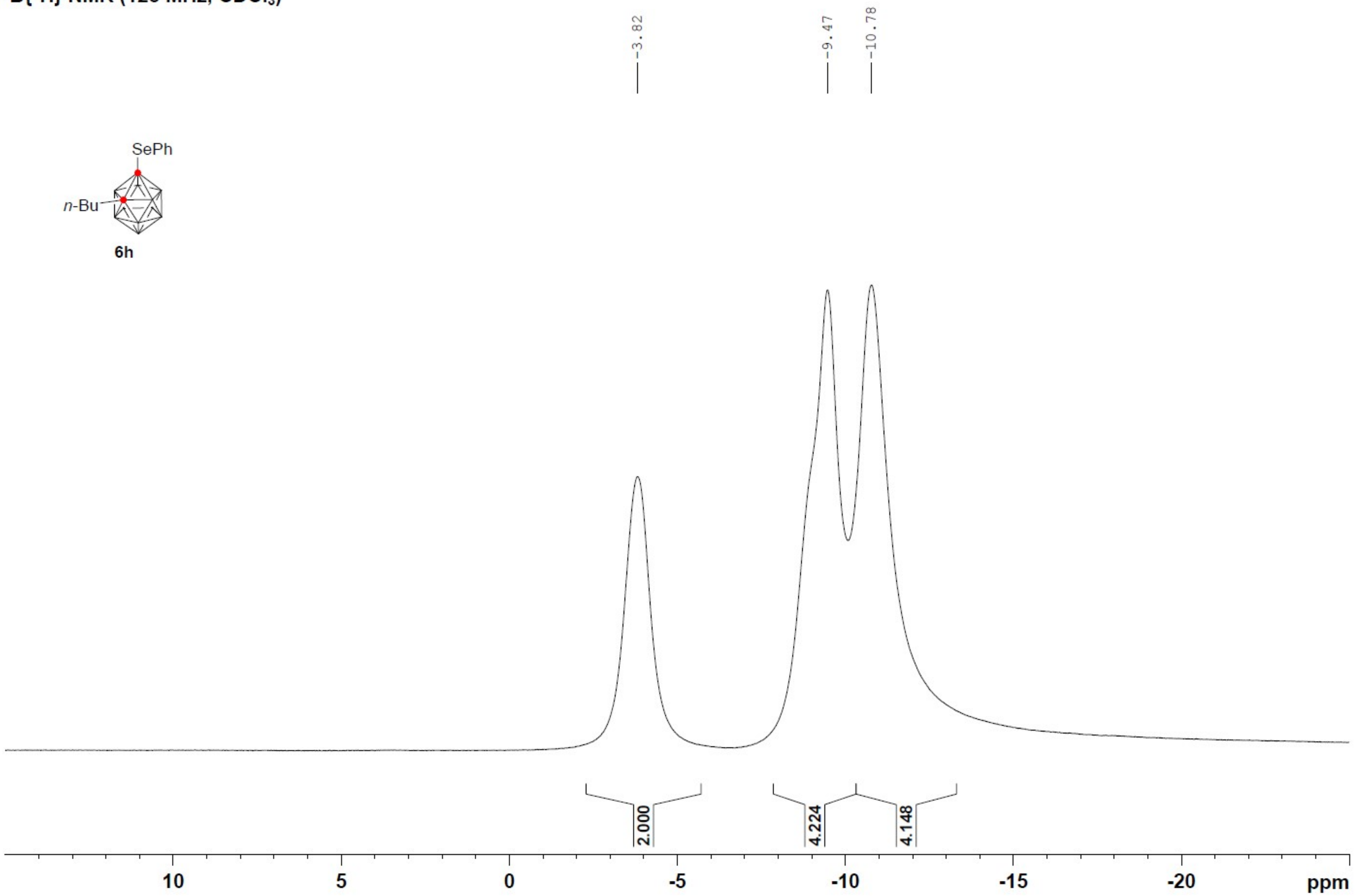

^{1}H NMR (400 MHz, $CDCl_3$)

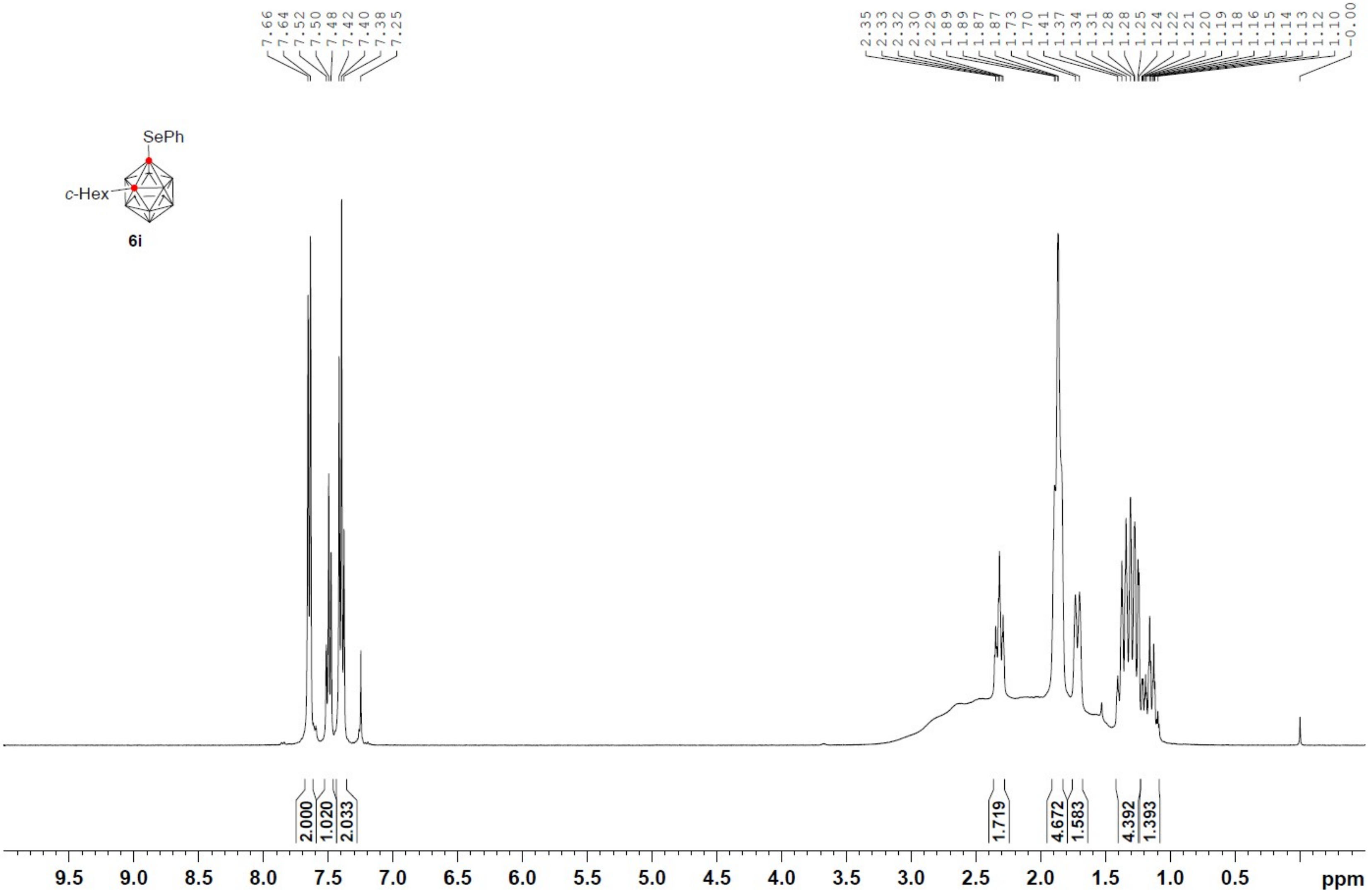

^{13}C NMR (100 MHz, $CDCl_3$)

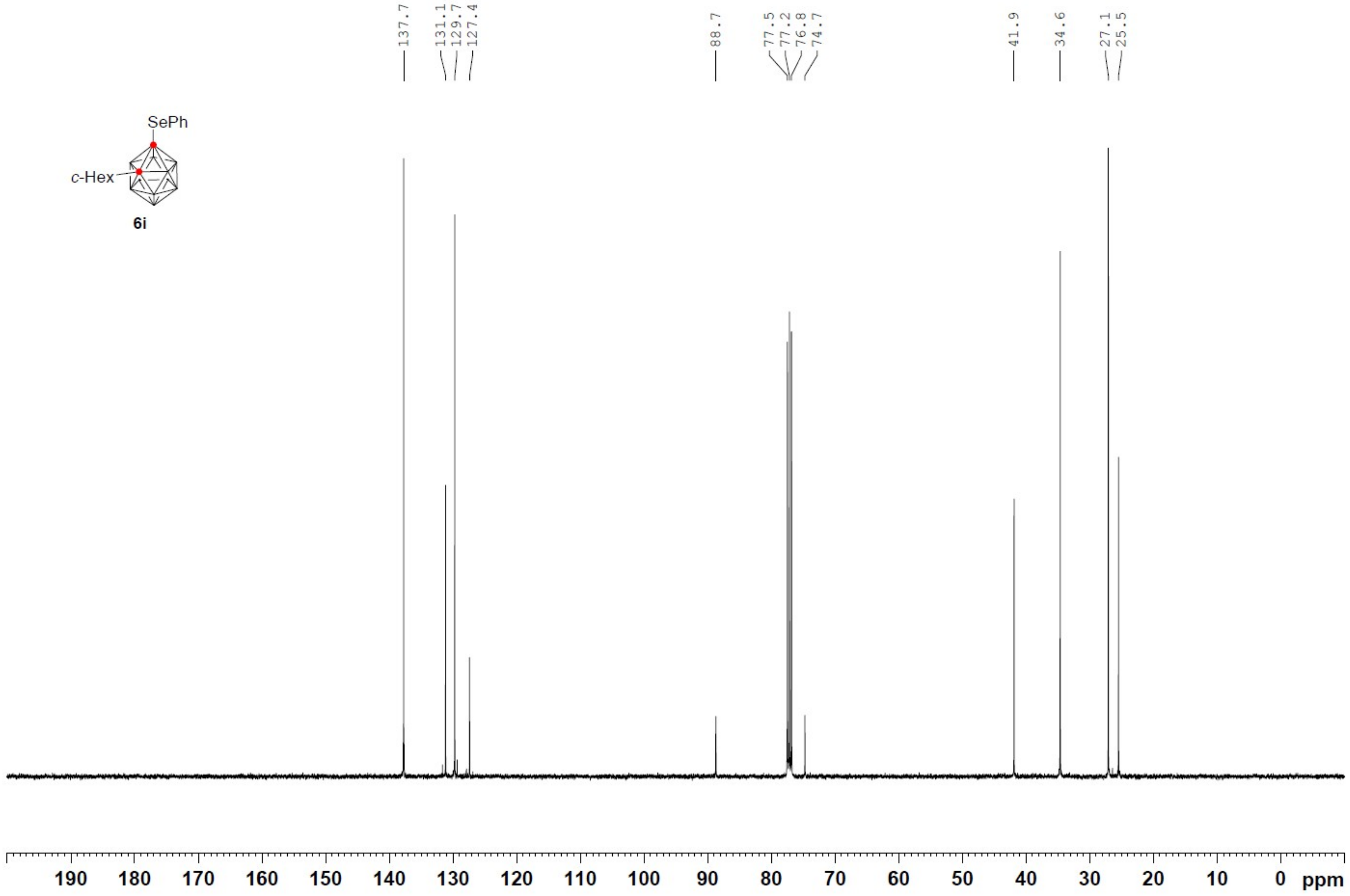

^{11}B NMR (128 MHz, $CDCl_3$)

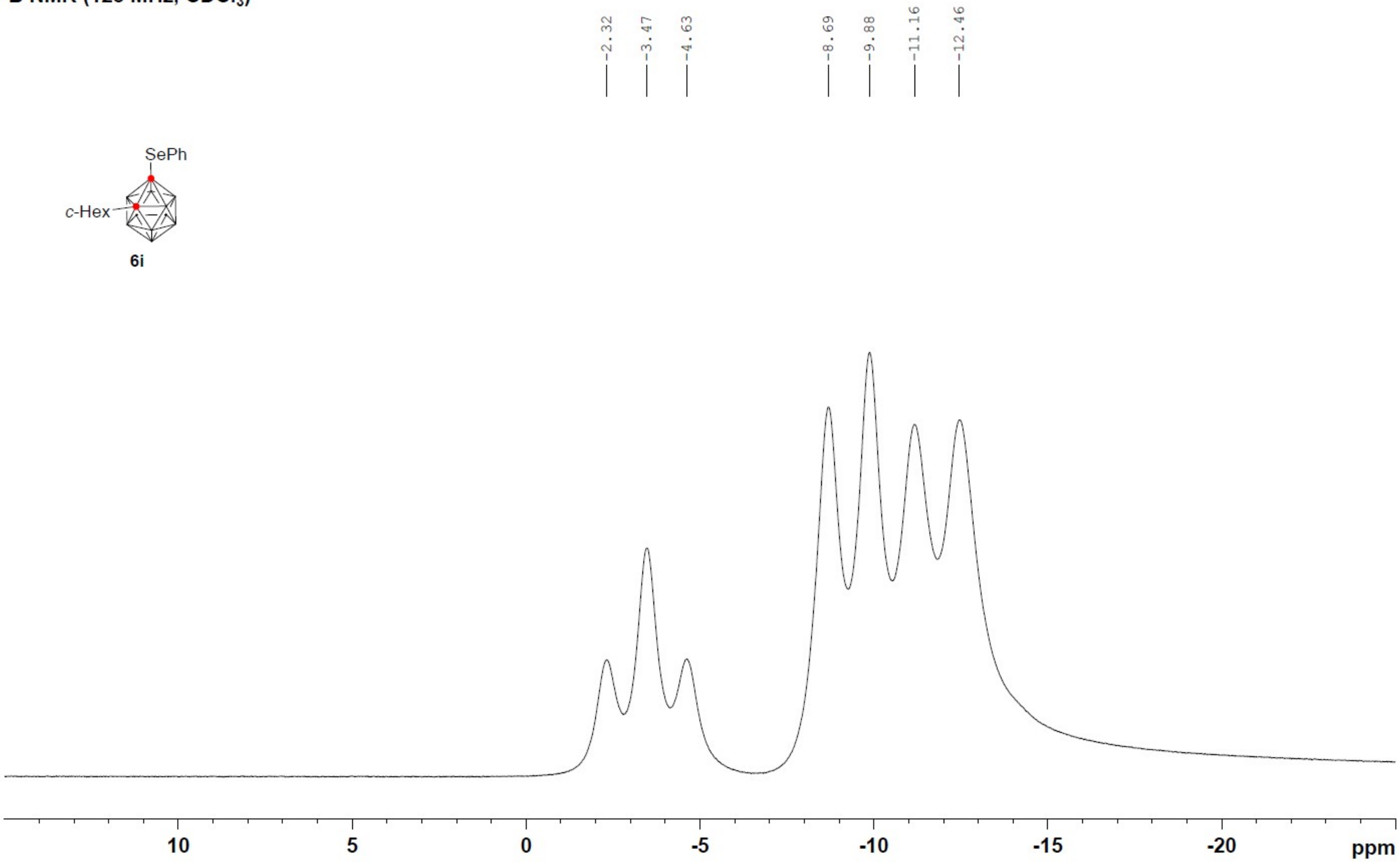

$^{11}B\{^1H\}$ NMR (128 MHz, $CDCl_3$)

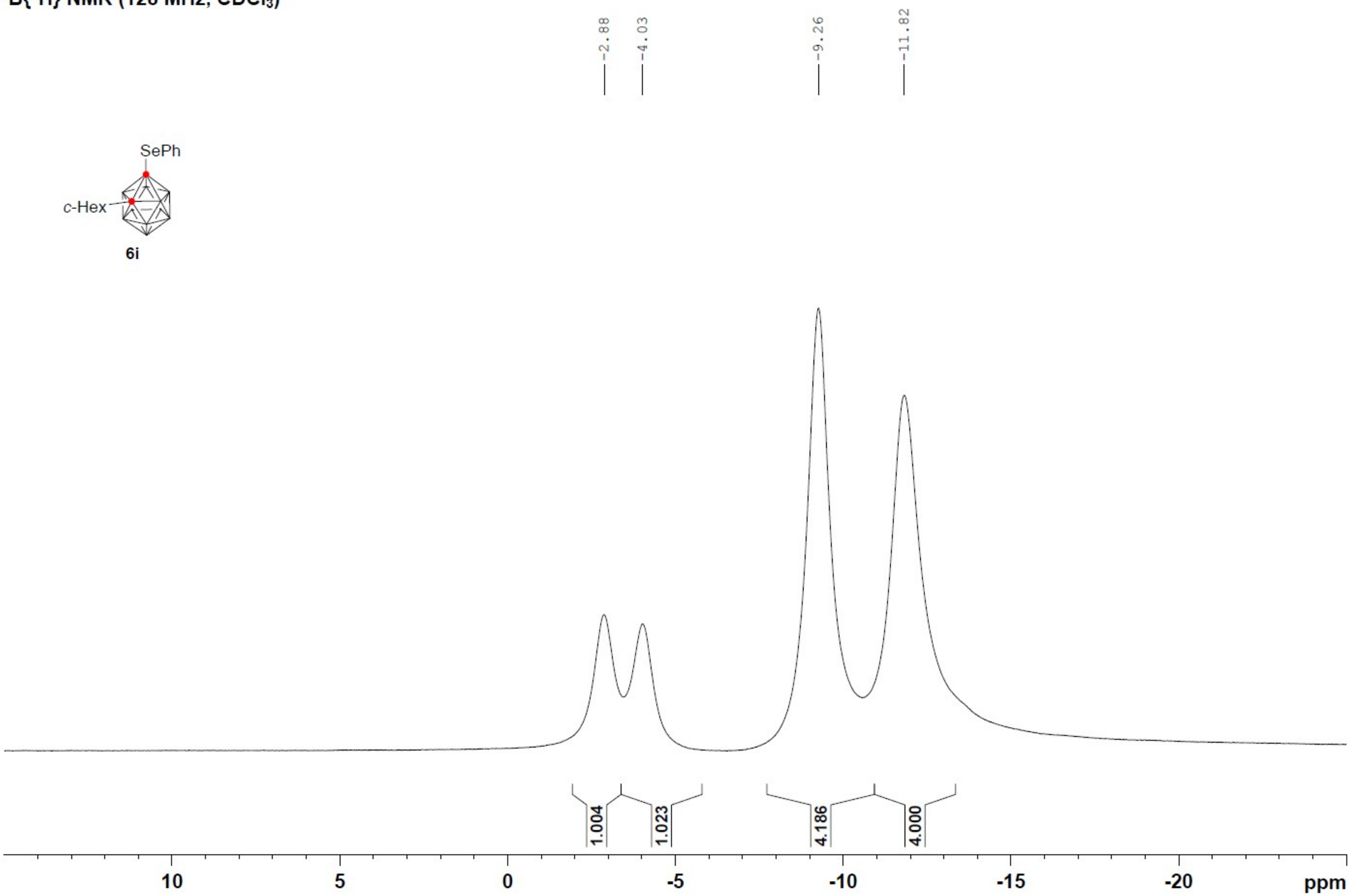

^1H NMR (400 MHz, $CDCl_3$)

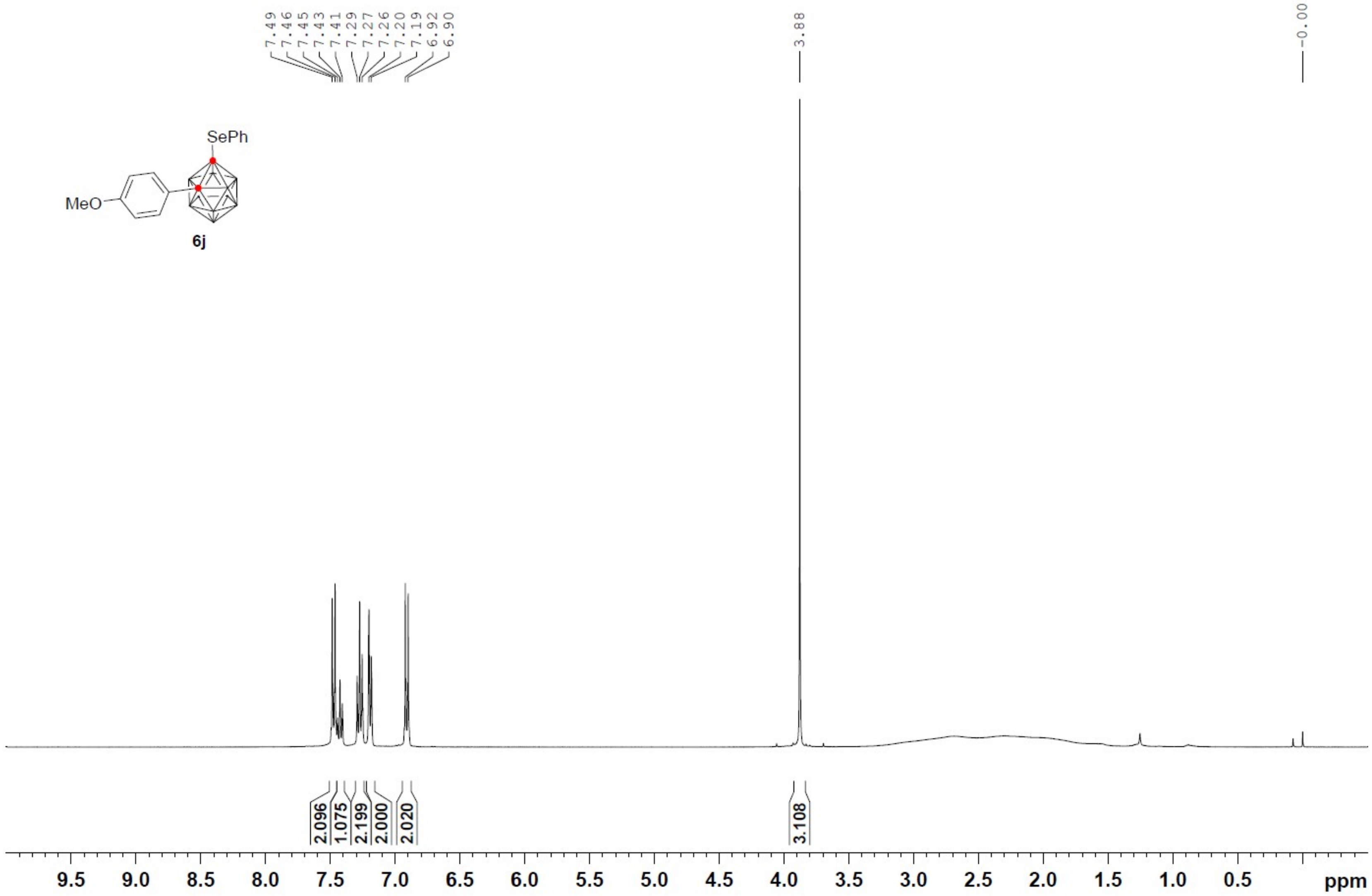

^{13}C NMR (100 MHz, $CDCl_3$)

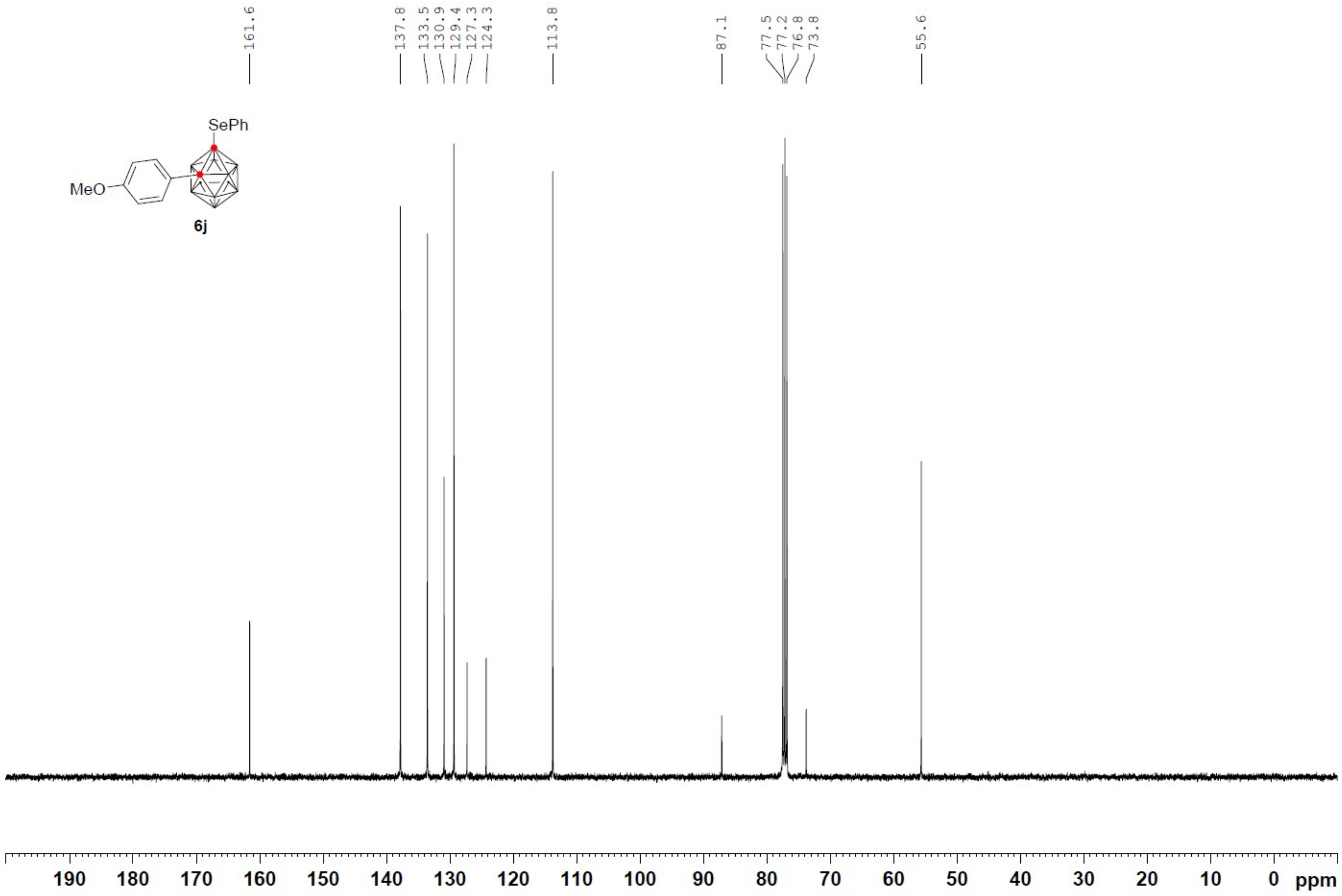

^{11}B NMR (128 MHz, $CDCl_3$)

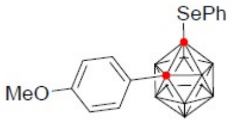

6j

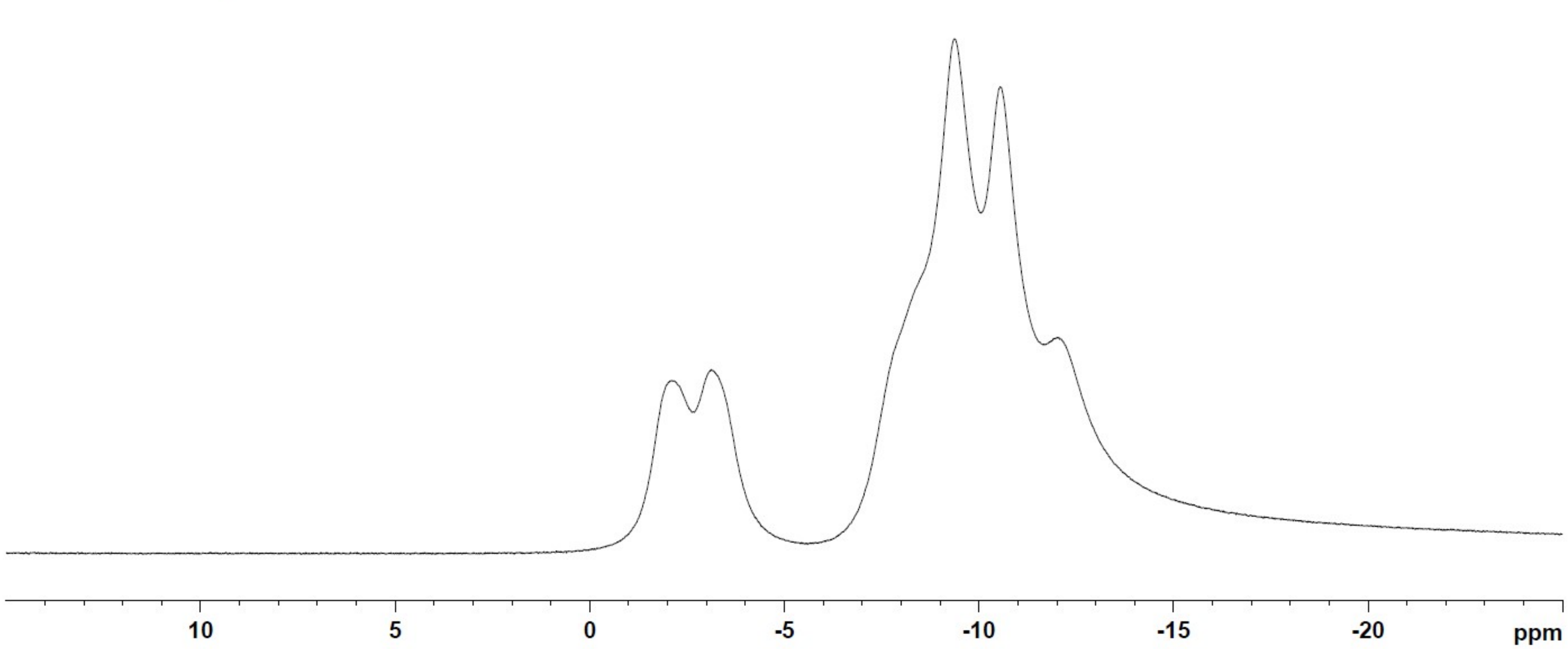

$^{11}B\{^1H\}$ NMR (128 MHz, $CDCl_3$)

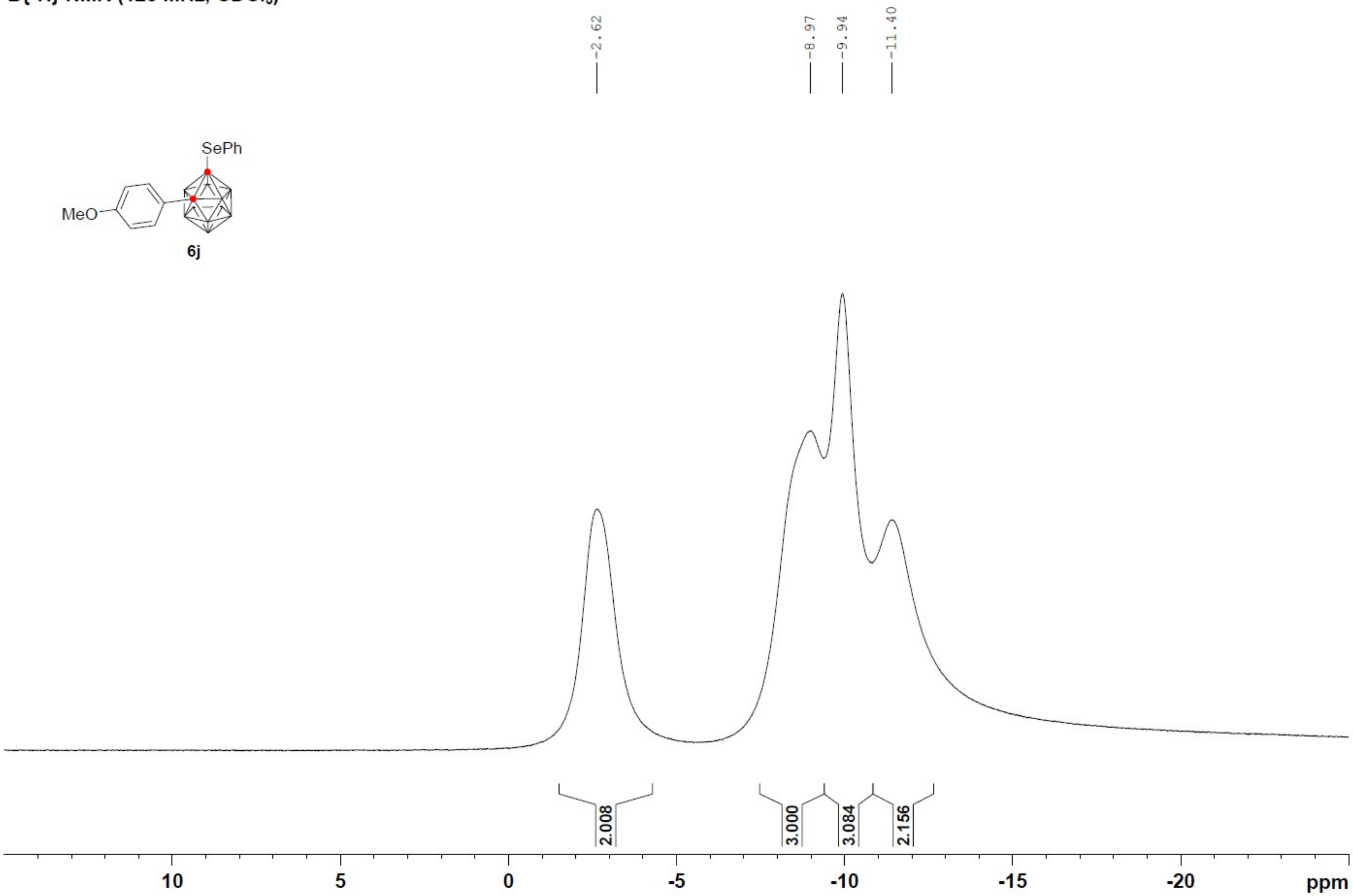

^{1}H NMR (400 MHz, $CDCl_3$)

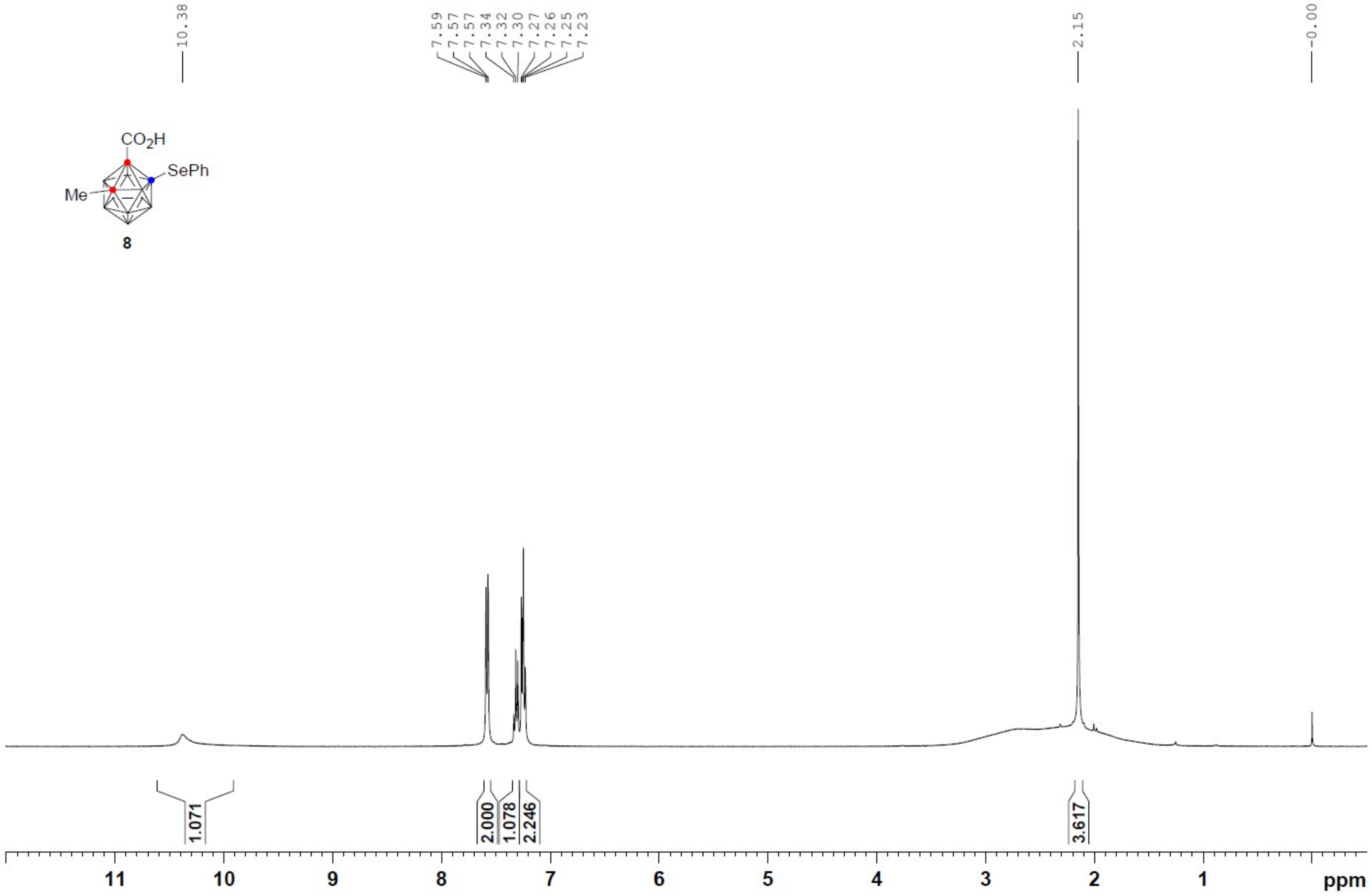

^{13}C NMR (100 MHz, CDCl$_3$)

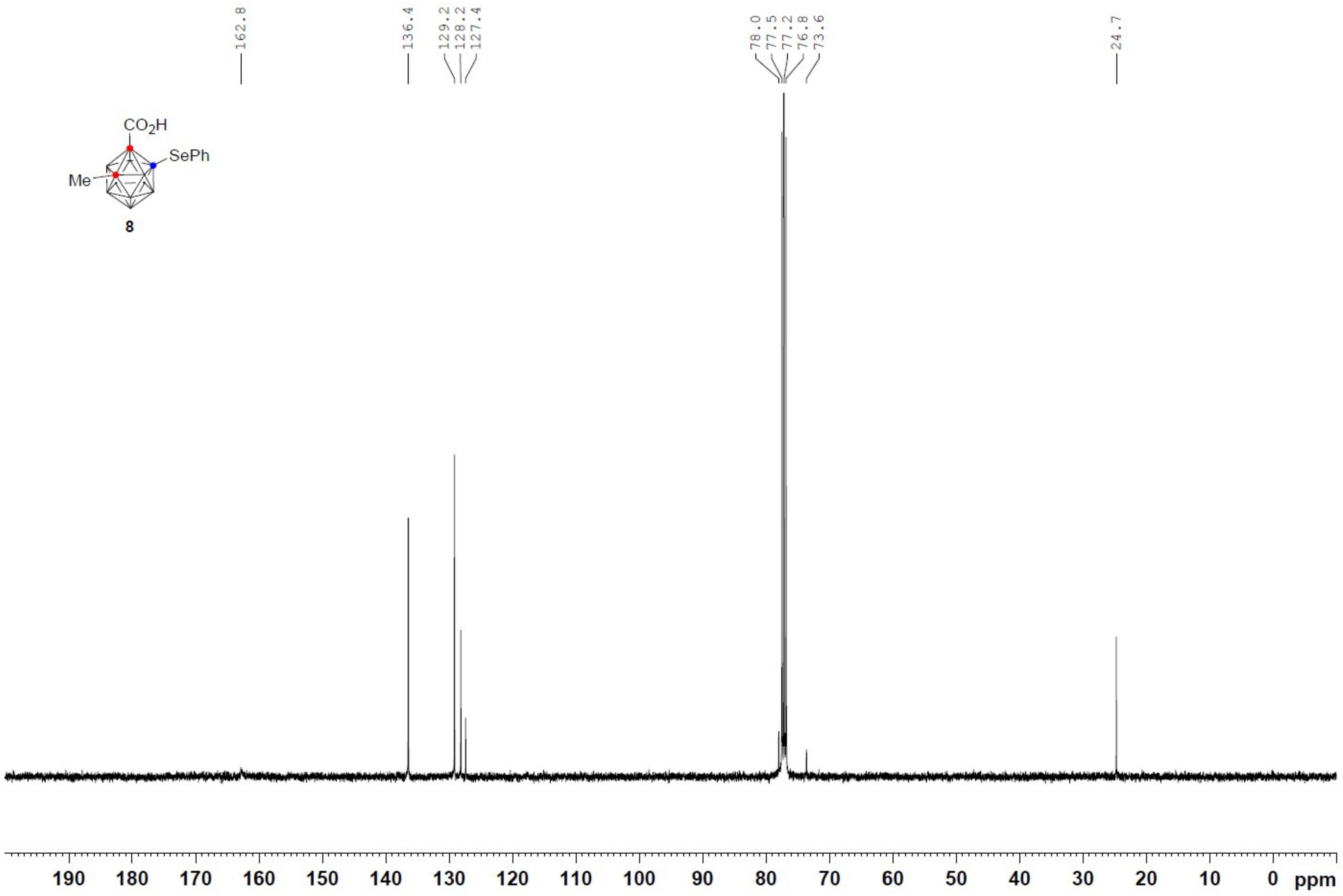

^{11}B NMR (128 MHz, $CDCl_3$)

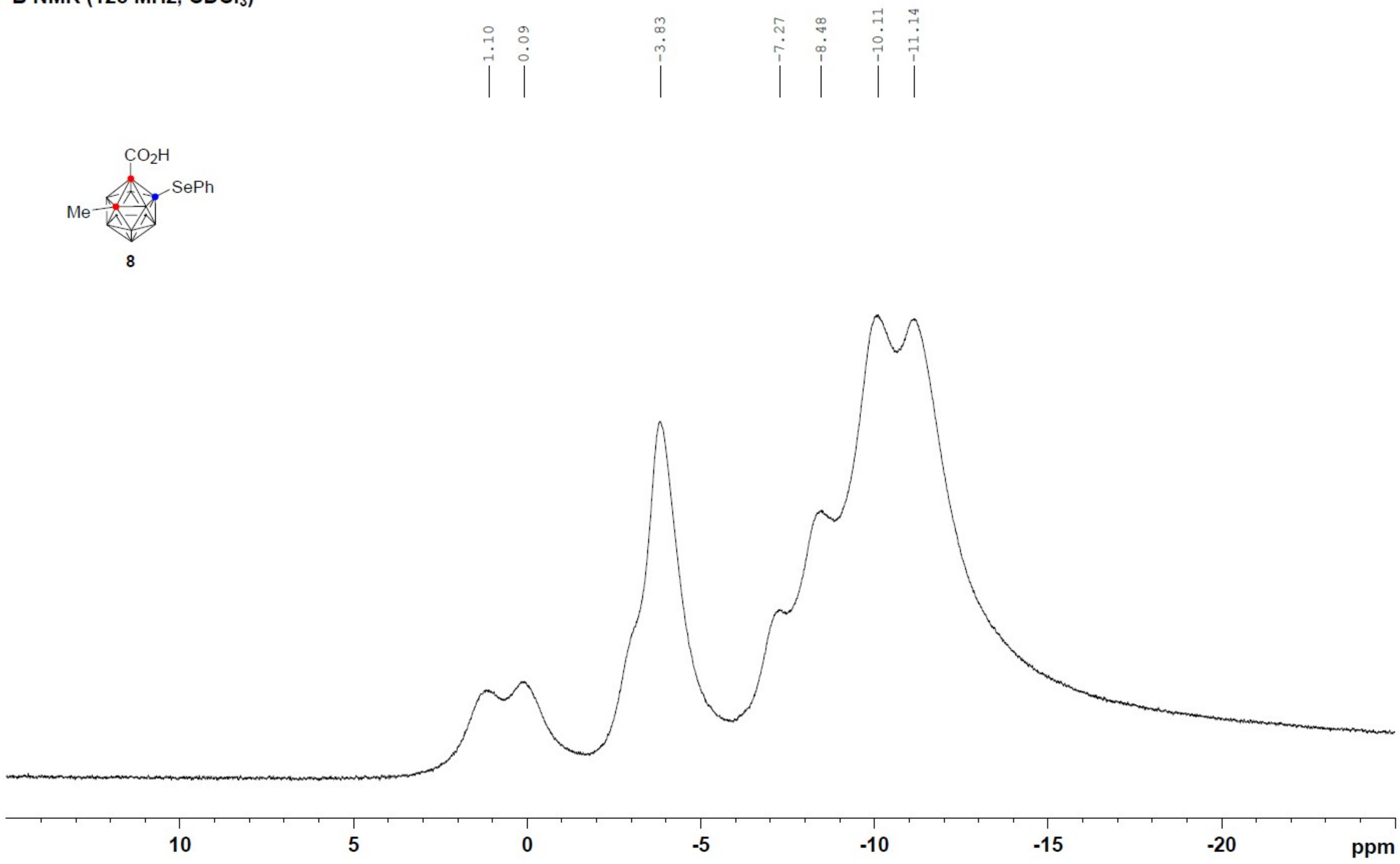

$^{11}B\{^1H\}$ NMR (128 MHz, $CDCl_3$)

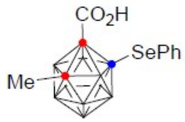

8

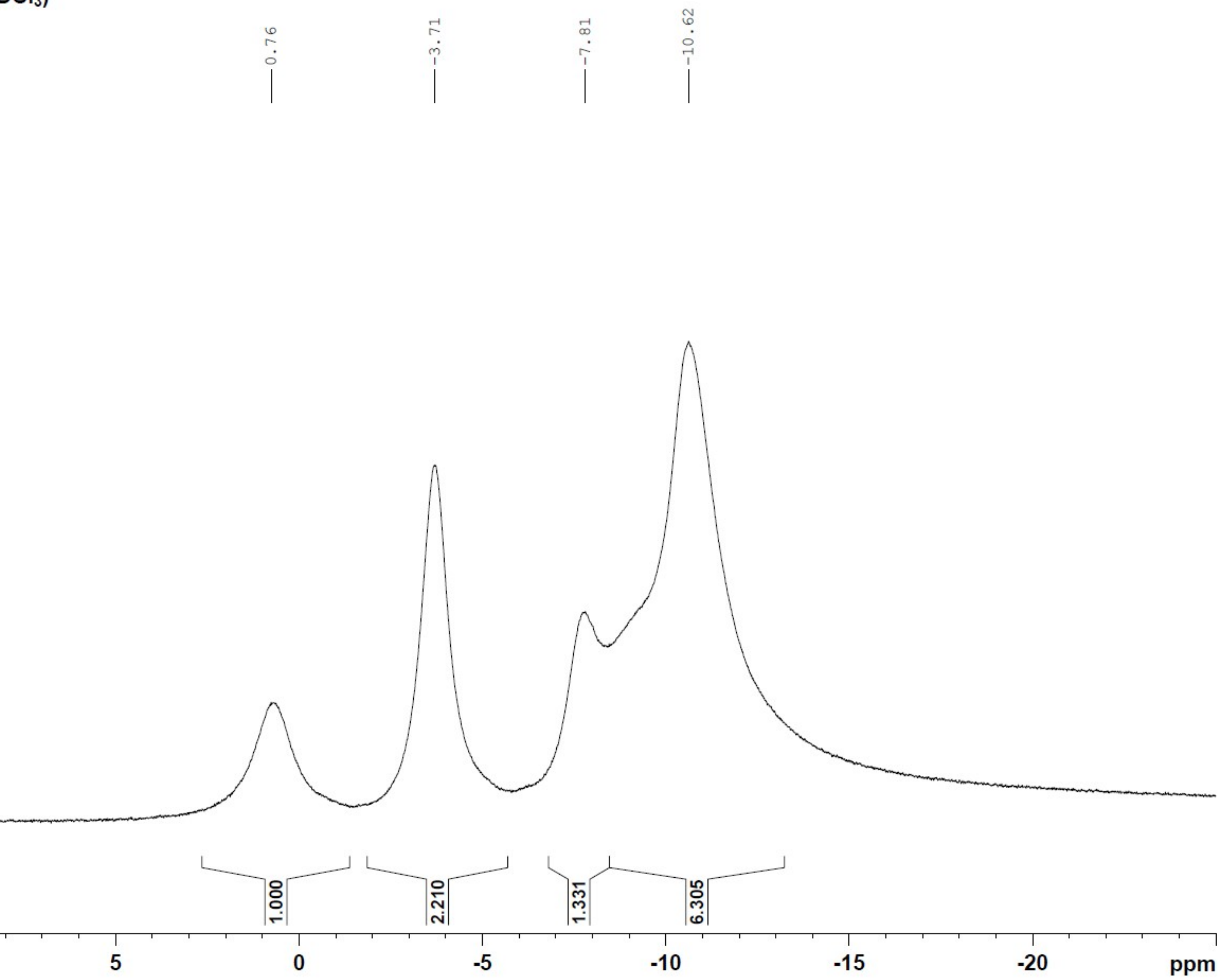

^1H NMR (400 MHz, $CDCl_3$)

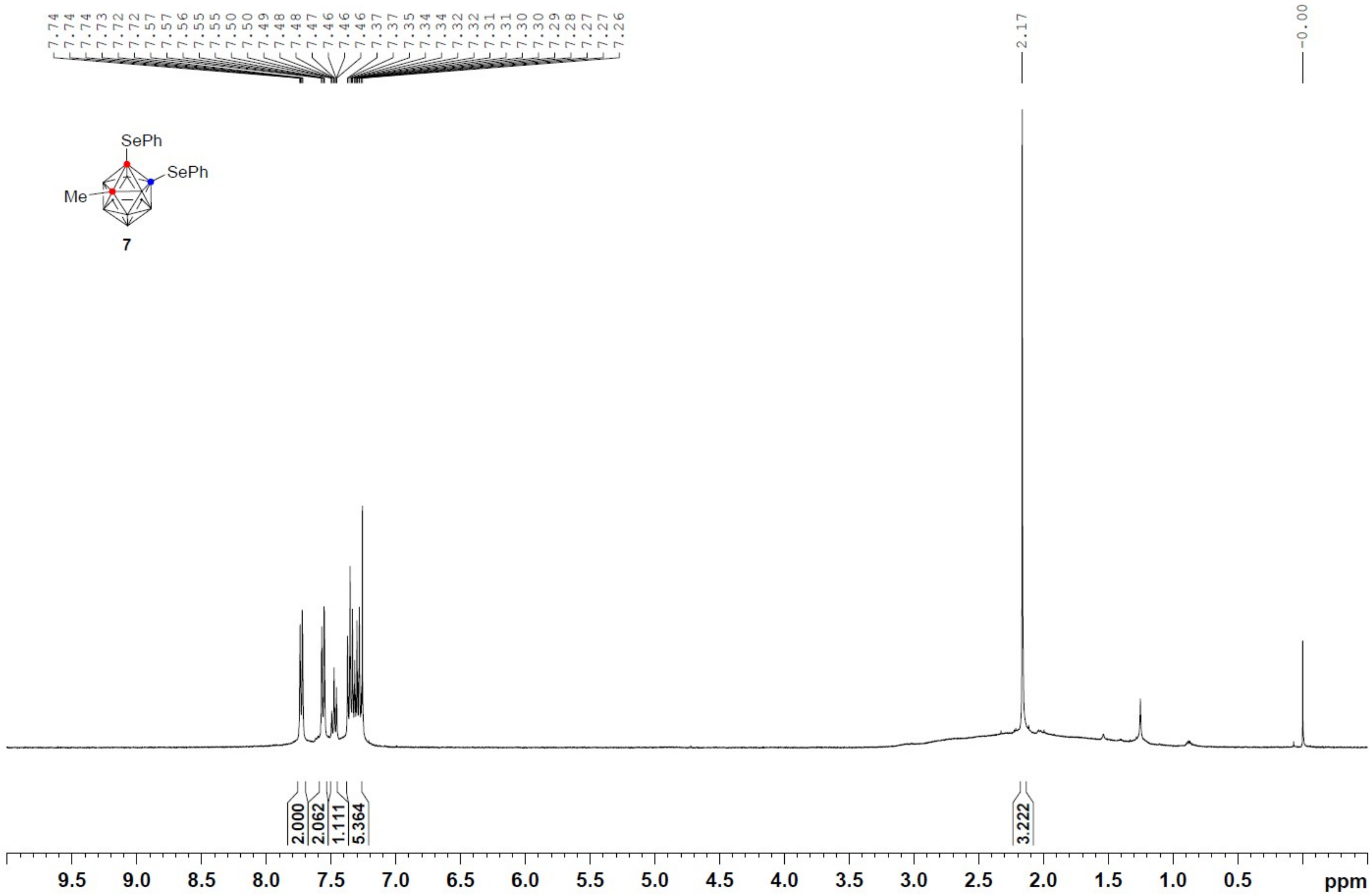

^{13}C NMR (100 MHz, $CDCl_3$)

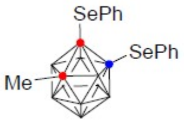

7

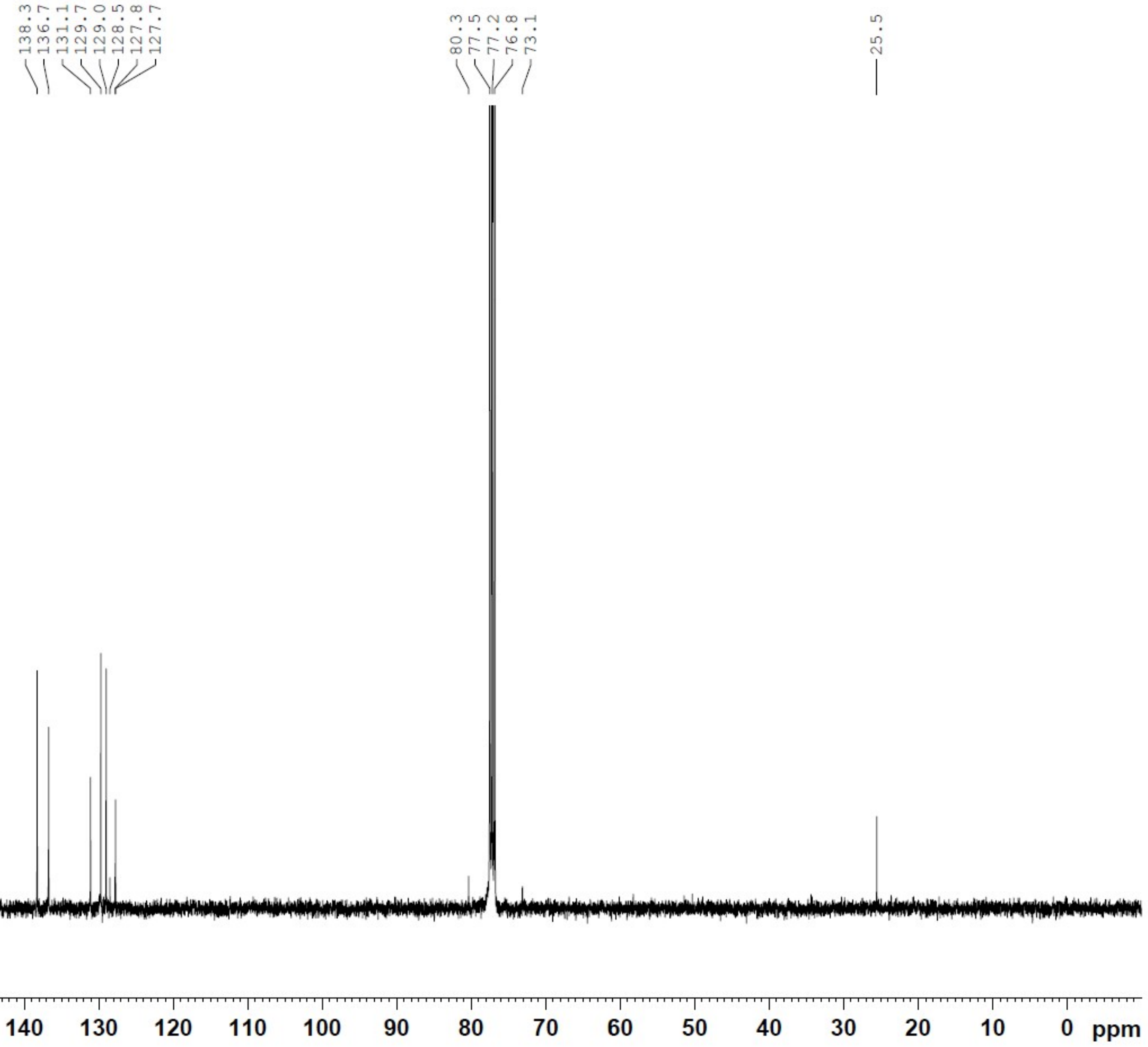

^{11}B NMR (128 MHz, $CDCl_3$)

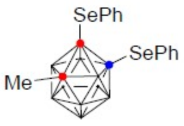

7

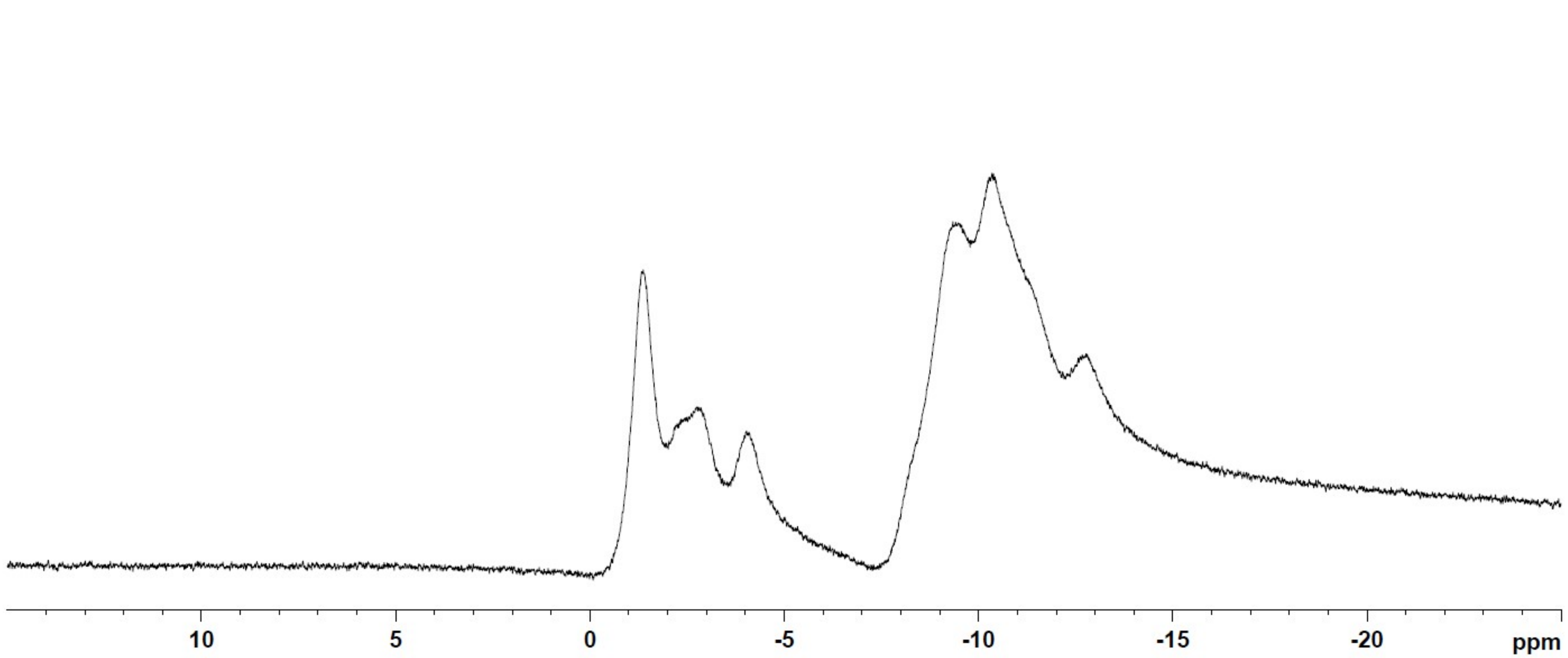

$^{11}B\{^1H\}$ NMR (128 MHz, $CDCl_3$)

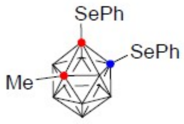

7

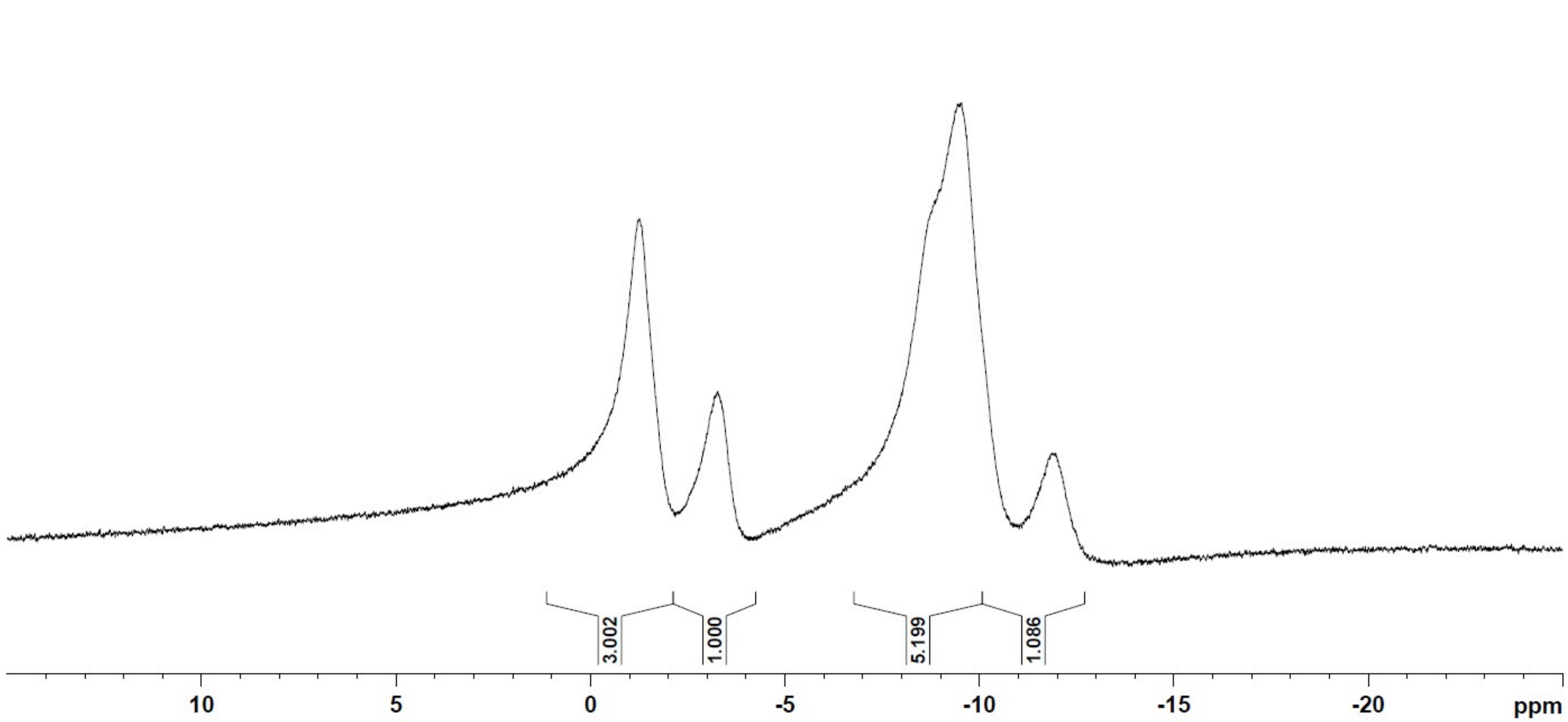

Supplement: SC-014-D2SC05590B-s001 [file SC-014-D2SC05590B-s001.pdf]
